# Secreted frizzled related protein is a target of PaxB and plays a role in aquiferous system development in the freshwater sponge, *Ephydatia muelleri*

**DOI:** 10.1371/journal.pone.0212005

**Published:** 2019-02-22

**Authors:** Chelsea Hall, Melanie Rodriguez, Josephine Garcia, Dora Posfai, Rachel DuMez, Erik Wictor, Omar A. Quintero, Malcolm S. Hill, Ajna S. Rivera, April L. Hill

**Affiliations:** 1 Department of Biology, University of Richmond, Richmond, Virginia, United States of America; 2 Department of Biological Sciences, University of the Pacific, Stockton, California, United States of America; 3 Department of Biology, Bates College, Lewiston, Maine, United States of America; University of Colorado Boulder, UNITED STATES

## Abstract

Canonical and non-canonical Wnt signaling, as well as the Pax/Six gene network, are involved in patterning the freshwater sponge aquiferous system. Using computational approaches to identify transcription factor binding motifs in a freshwater sponge genome, we located putative PaxB binding sites near a Secreted Frizzled Related Protein (SFRP) gene in *Ephydatia muelleri*. *EmSFRP* is expressed throughout development, but with highest levels in juvenile sponges. In situ hybridization and antibody staining show *EmSFRP* expression throughout the pinacoderm and choanoderm in a subpopulation of amoeboid cells that may be differentiating archeocytes. Knockdown of *EmSFRP* leads to ectopic oscula formation during development, suggesting that EmSFRP acts as an antagonist of Wnt signaling in *E*. *muelleri*. Our findings support a hypothesis that regulation of the Wnt pathway by the Pax/Six network as well as the role of Wnt signaling in body plan morphogenesis was established before sponges diverged from the rest of the metazoans.

## Introduction

While it is well established that the Kingdom Animalia is monophyletic, and that the Cnidaria and bilaterians are sister groups, the phylogenetic placement of the earliest branching, non-bilaterian animal lineages (i.e., the Porifera and Ctenophora) is currently a point of vigorous debate (e.g., [1–6]). Improved phylogenomic models support sponges as a sister group to all other animals [7], but interest in the branching order of the Porifera and Ctenophora remains because these animal groups provide insights into the last common ancestor to the animals. Regardless of the precise phylogenetic placement of sponges and ctenophores, our knowledge of all aspects of the biology of the extant metazoans will expand through detailed examination of these two poorly studied key animal lineages [8]. Members of the phylum Porifera, once thought to be simple animals lacking tissue-level organization, are now known to be more complex than previously understood, and share many features with the other animals (e.g., epithelia, sensory perception, cell adhesion, stem cells, gastrulation, gene and RNA regulatory networks; [8–13]).

A central feature of the sponge body plan is a branched water canal system, and this constitutes the main body axis of sponges [14]. Unidirectional water movement in the sponge aquiferous system begins at epithelial porocytes (i.e., ostia), moves through endopinacocyte lined canals, reaches filtering choanocytes in choanocyte chambers, and then leaves the sponge through excurrent canals and the osculum [15]. Despite the simple body axis (i.e., ostia to oscula), many complex aspects of metazoan organization, structures, and development are contained in the sponge body. For example, the epithelial lining of the oscula in some sponges, including the sponge studied here (*Ephydatia muelleri*), contain primary cilia involved in flow sensation and coordination of contractile behavior [16]. Studying the molecular, genetic, and developmental mechanisms that underlie the morphogenesis of the sponge aquiferous system will shed light on our understanding of sponge body plans and may provide clues about the development of more complex animal forms.

Data has begun to shed light on the role of two key developmental regulatory pathways in sponge body plan morphogenesis. Components of the Wnt signaling pathway, known widely for its role in body axis patterning, cell fate determination, stem cell maintenance and renewal, gastrulation, as well as cell proliferation and migration in animal development and evolution [17–18], have been described in both marine and freshwater sponges. In marine sponge species such as *Amphimedon queenslandica* and *Sycon ciliatum*, some Wnt genes are expressed at the poles of larvae [19–20]. In some adult sponge tissues, the osculum has been shown to exhibit Wnt expression in species such as *S*. *ciliatum* and *Halisarca dujardini* [21]. In the marine sponge *Oscarella lobularis*, one of the Wnts is expressed in newly forming ostia and activation of the Wnt pathway by inhibition of GSK3 leads to increased number of ostia in this species [22].

The Pax/Six pathway, a module of the PSED network with known functions in morphogenesis of sensory and other organs (e.g., eyes, kidneys, muscles, branchial arches; (reviewed in [23–24]) in bilaterian models, has also been elucidated in marine and freshwater sponges. The pathway has possible roles in morphogenesis of choanocyte chambers and putative larval sensory cells in *Sycon ciliatum* [25], and in choanosome and osculum development in adult tissues of *Chalinula loosanofi* [26].

Components of the Wnt and the Pax/Six networks have also been shown to be involved in formation of the aquiferous system of the sponge body plan in the emerging model freshwater sponge, *Ephydatia muelleri* [14,27–29]. Wnt ligands are expressed in subsets of amoeboid cells with filipodia in the mesohyl of the *Ephydatia* aquiferous system [29], and β-catenin nuclear staining also localizes to a subset of amoeboid cells in the mesohyl [29–30]. The cells expressing Wnts and β-catenin are possibly archeocytes (i.e., stem cells), but may be distinct populations [29]. Activation of Wnt signaling by inhibition of GSK3 results in sponges that form additional ectopic oscula and an irregularly branching canal system [14,29]. Wnt antagonists, on the other hand, inhibit osculum development and regeneration [29]. Furthermore, inhibition of a downstream module of the Wnt pathway, the Rho-associated protein kinase (ROCK), leads to loss of a functional aquiferous system and absence of an osculum [28]. PaxB and Six1/2, both expressed in endopinacodermal cells lining the canal system of *E*. *muelleri*, play roles in the specification, differentiation, or maintenance of the canal system since knockdown of either gene leads to canal system defects with missing epithelial cells in the lining of incurrent and excurrent canals. Further, PaxB was shown to regulate expression of Six1/2 in *E*. *muelleri* [27]. Earlier knockdown of these genes (i.e., before establishment of the aquiferous system) can also lead to branched or forked oscula (A. Hill, personal observation). These data imply that the role of both pathways in animal body plan morphogenesis may predate the divergence of animals from the last common ancestor.

Connections between the PSED and Wnt signaling pathways are known in several developmental contexts in animals. For example, canonical Wnt signaling represses *sine oculus/Six* and *eyes absent/Pax6* in developing visual systems of flies and vertebrates [31], and Wnt and Pax genes regulate several aspects of CNS development [32–33]. Here, we asked what downstream genes are under regulation of the transcription factor EmPaxB? We hypothesized that if connections between the Wnt pathway and the Pax/Six network were established before sponges diverged from the rest of the metazoans, we might find some Wnt pathway genes under EmPaxB regulation.

We describe a novel Secreted Frizzled Related Protein (SFRP) in the freshwater sponge, *Ephydatia muelleri* (*EmSFRP*). The gene was identified as part of a screen for PaxB binding motifs in the *E*. *muelleri* genome and we determined that knockdown of EmPaxB leads to lower levels of *EmSFRP* expression. We show that *EmSFRP* is expressed during *E*. *muelleri* development and specifically in amoeboid cells with filipodial-like projections in the choanosome and at the periphery of pinacodermal growth. dsRNA directed to *EmSFRP* leads to formation of additional oscula supporting a hypothesis that SFRP may act as an antagonist of Wnt signaling in *E*. *muelleri*.

## Materials and methods

### Collection and culturing of sponges

Gemmules were collected at the outflow of the dam at Bryan Park, Richmond, VA (37.5963° N, 77.4725° W) during late winter/early spring, and were stored in sampling water or cold Strekal’s media (SM) at 4°C in the dark [34]. Sponge gemmules were collected under Virginia Department of Game and Inland Fisheries Permit #047944 and these are not endangered or protected species. Gemmules were picked from host sponge tissue and washed once in cold 1% hydrogen peroxide solution and SM and 8–10 times in SM before hatching in petri dishes or 12-well plates in SM at room temperature in the dark. Media was replaced daily after sponge hatching.

### Identifying putative downstream targets of PaxB

**Fig 1:**
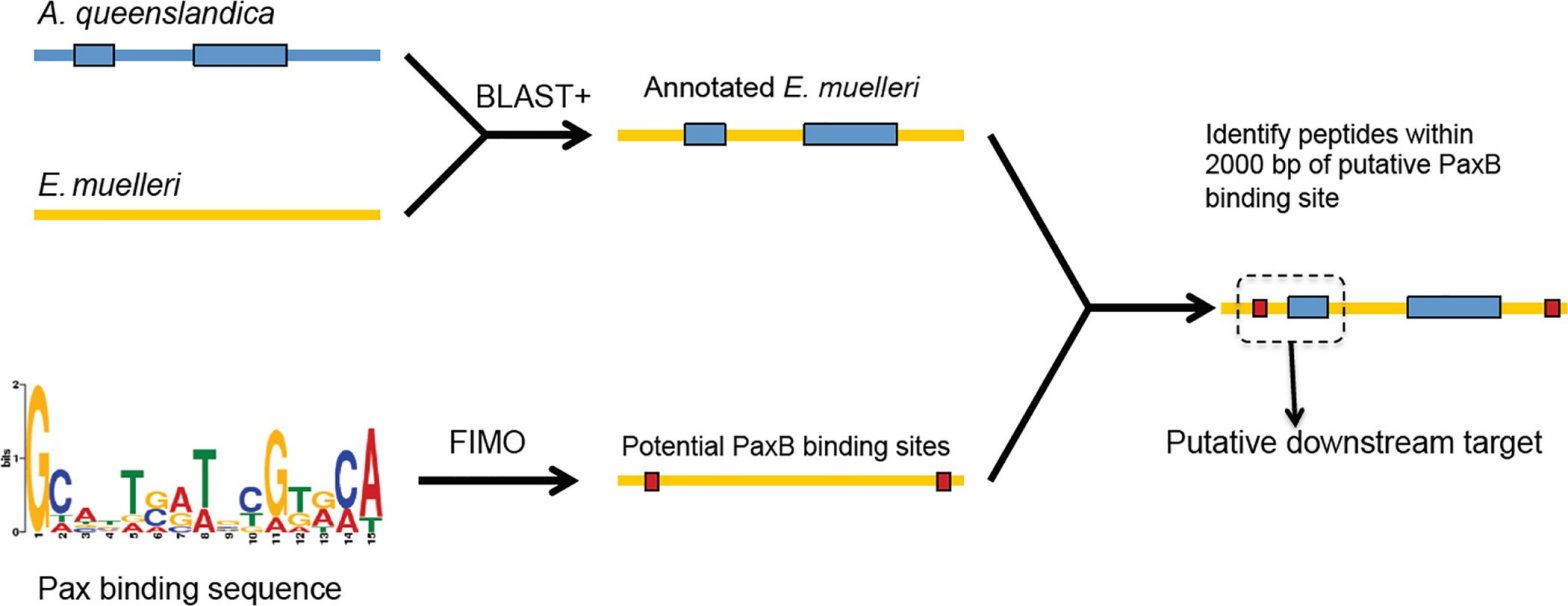
Schematic of computational approach used to identify potential downstream targets of EmPaxB. An *Ephydatia muelleri* low quality draft genome was “roughly annotated” against *Amphimedon queenslandica* predicted peptides database. Separately, potential sponge-specific binding sites of EmPaxB were identified in the *E*. *muelleri* genome using Find Individual Motif Occurrences (FIMO).

Putative downstream targets of EmPaxB were identified using a computational approach (Fig 1). Our previous study [27] found double stranded DNA sequences that bound to the *E*. *muelleri* PaxB paired-domain, as well as closely related sequence motifs that did not bind (S1 Table). Using these positive and negative binding sites, FIMO (MEME suite [35–36]) was used to generate a position specific probability matrix (PSPM) for genomic searches (MEME PSPM available in S1 File). A draft *E*. *muelleri* genome was scanned for this motif with FIMO using default parameters. FIMO estimated q-values (with a minimum false discovery rate at which a given motif is deemed significant) automatically from a uniformly sampled set of 10000 p-values, getting pi_0 = 0.9925. In parallel, the draft *E*. *muelleri* genome was roughly annotated with the *Amphimedon queenslandica* proteome. *E*. *muelleri* genomic scaffolds were searched to find regions that were enriched for FIMO hits within 2000bp of a protein-coding sequence using an in-house algorithm (compare.cpp and optparse.cpp scripts and FIMO genome scaffolds available in S2, S3 and S4 Files). Two of the top hits contained sequences with high similarity to frizzled-family proteins, SFRP.

### Phylogenetic analysis

To assess homology between our putative *E*. *muelleri* SFRP sequence (GenBank MG851821) and known SFRPs, phylogenetic analysis was performed with PhyML. *Ephydatia muelleri* sequences were used as bait in three types of blastp searches implemented in blast++ or UniRef [37–38] to find related sequences. 1) UniRef50 database (uniprot.org) was blasted to find the top 50 sequence-cluster hits. 2) The *Branchiostoma floridae*, *Capitella telata*, *Lottia gigantean*, *Danio rerio*, *Drosophila melanogaster*, *Nematostella victensis*, *Trichoplax adhaerens*, *Amphimedon queenslandica*, and *Monosiga leidyi* RefSeq databases downloaded from NCBI as well as *Mnemiopsis leidyi*, *Hydra vulgaris*, and *Acropora millepora* (Compagen) were blasted to find species-specific complements of SFRP-similar sequences. No *M*. *brevicolis* sequences were returned. 3) Available sponge transcriptomes including *E*. *fluviatilis*, *E*. *muelleri*, *Haliclona amboinensis*, *H*. *tubifora*, *Leucosolenia complecata*, *Sylissa carteri*, *Sycon ciliatum*, *Xestospongia testudinaria* (Compagen), *Aphrocallistes vastus* (Compagen), *Corticium candelabrum*, *Chondrilla nucula*, *Ircinia fasciculate*, *Petrosia ficiformis*, *Spongilla lacustris*, *Pseudospongosorites suberitoides*, *S*. *coactum* [39], and *Lubomirskia baicalensis* [40] were also blasted.

Sequences that were less than 80 amino acids long as well as *Mus musculus* ROR were removed and the remaining sequences were aligned with Muscle [41] implemented in SeaView for a PhyML tree [42] or with MAFFT using progressive alignment method L-Ins-1 for IQ-TREE [43] (raw and annotated alignments available in S5, S6, S7, S8 and S9 Files). Since the transmembrane domains of full length proteins carried a strong signal in the alignments, non- full length proteins were spuriously grouped together in the PhyML tree. For this reason, the aligned sequences were trimmed to contain only the CRD, as most sponge sequences were incomplete but contained this domain.

jModelTest suggested a Whelan and Goldman (WAG) model of substitution for the CRD-only sequences [44]. This model was used in PhyML [45], implemented in SeaView, using a Neighbor Joining Tree as a starting tree and the aLRT method of generating support values [46]. In parallel we ran a tree using a full-length alignment in IQ-TREE using abayes for support values [47]. ModelFinder [48] suggested a VT+I+G4+F model for the full-length sequences (a general matrix VT model with invariable sites, a discrete gamma model with 4 rate categories, and empirical amino acid frequencies from data).

### Domain analysis

Domain analysis was performed in Pfam [49] on full length sequences (note that most sponge transcriptome sequences lacked either a start, a stop, or both). Predicted N-glycosylation within the frizzled domain with NetNGlyc 1.0 [50] were identified. A protein was assessed as being a SFRP if it a) contained a Frizzled domain, b) Lacked a transmembrane domain or other protein domains (such as a Netrin domain) and b) grouped with other SFRP proteins in the phylogenetic analysis.

### RNA isolation and qRT-PCR analysis

Sponge tissue was harvested at developmental time points or after RNAi treatment by scraping sponges off of the bottom of the culture plate using the tip of a pipet and placing tissue into RNAlater (ThermoFisher) solution. In most cases, sponges were grown in 12-well plates at 2–3 sponges per well and tissue was pooled for ~12–24 sponges per time point or treatment. RNA was isolated from *E*. *muelleri* sponges using the RNeasy Mini Kit (Qiagen), limiting genomic DNA contamination through an additional on-column DNase I treatment. Equal amounts of cDNA (150μg) were synthesized from sponge mRNA using Superscript VILO reverse transcriptase master mix (ThermoFisher Scienctific). SYBR Green (ThermoFisher Scientific) chemistry and Chromo4 (BioRad Laboratories) were used for qPCR with *EmSFRP* gene specific primers (F: 5’-CAGCAAGACAAAAGTGGCG-3’; R: 5’-CGCTCTCACCCATTCACACA-3’) or *EmRXR* primer (F: 5’-TCCTGAAGGCTAGCTGGTGT-3’; R: 5’GGGACTCAAACCACAAGGAA-3’). *EmSFRP* levels were normalized to the housekeeping gene Ef1α (F: 5’-GCGGAGGTATCGACAAGCGT-3’; R: 5’-AGCGCAATCGGCCTGTGAG-3’) and/or GAPDH (5’-TCAAGGGAACAGTGGAGAC-3’; R: 5’-TGTCCTTGGTGGTGAAAACA-3’) and expression was quantitated as described [31]. Primers were designed and tested for specificity using CLC Workbench (Qiagen) or NCBI Primer-BLAST. Before use in qRT-PCR, all primers were tested for optimal Tm and PCR conditions, and checked to ensure a single band of the correct size was amplified. For qRT-PCR analysis, samples were assayed in duplicate or triplicate (technical replicates) with at least two biological replicates per experiment.

### In situ hybridization

In situ hybridization was carried out as described in Rivera et al. [27] with some modifications. Sponges were fixed with 4% paraformaldeyhyde in ¼ Holtfreter’s solution (HS) overnight followed by a dehydration series (25% ethanol in ¼ HS; 50% ethanol in ¼ HS, 75% ethanol in ¼ HS, 100% ethanol) and stored at -80°C until use. Prior to hybridization, tissues were rehydrated through an ethanol/PBT (phosphate-buffered saline with 0.1% Tween-20) series followed by a 10 min wash in PBT. Endogenous peroxidases were quenched with one wash for 30 min in 1% H_2_O_2_ in PBT. Tissue was treated with 1 μg/ml Proteinase K for 1 min at RT and immediately washed in 2 mg/ml glycine to stop the reaction. Tissue was washed once in PBT and then post-fixed for 30 min in 4% paraformaldehyde/phosphate buffered saline (PBS) at 4°C. Tissue was washed twice for 10 min in PBT prior to prehybridization. Tissue was prehybridized at 55–60°C for at least 2 h (up to overnight) in (50% formamide, 5X saline-sodium citrate (SSC) buffer at pH 4.5, 50 μg/ml heparin, 0.1% Tween-20, 10 mM dithothreitol (DTT), 1X Denhart’s and 100 μg/ml sheared salmon sperm DNA). Hybridization was carried out overnight with digoxygenin labeled anti-sense and sense RNA probes (10–0.5 ng/μl) at 55–60°C. Probes were recovered and used multiple times and results with lowest background hybridization were achieved with probes re-used multiple times. After hybridization and probe removal, tissues were washed for 10 min in hybridization buffer at 55°C followed by successive washes for 20 min each at 55°C in 75% post-hybridization buffer/25% 2X SSC; 50% post-hybridization buffer/50% 2X SSC; 25% post-hybridization buffer/75% 2X SSC where post-hybridization buffer contained 50% formamide, 5X SSC, and 0.1% Tween-20. Tissues were then washed three times in 2X SSC at 55°C and then for 10 min in at 1:1 solution of 2X SSC/maleic acid buffer before proceeding to blocking steps. Blocking and subsequent tissue processing was as described in Rivera et al. [27].

### Antibody design and immunoblot analysis

A novel *EmSFRP* antibody was designed using the CRD (cysteine rich domain) region from the mRNA sequence. *EmSFRP* antigen (peptides 349:366; DPVHVDSLRTRQSIPVPK) and the resulting antibody was generated by Thermo Scientific. The terminal bleeds from each rabbit were affinity purified and ELISA tested (Thermo Scientific). Antibody specificity was characterized using Western Blot of *EmSFRP* native protein lysate. Proteins from the lysates were separated by electrophoresis on 10% Mini-PROTEAN TGX Stain-Free gels (BioRad) and transferred to a nitrocellulose membrane. Following the transfer, the membrane was incubated in 5% milk/TBST block solution for 1 hour. The membrane was incubated with diluted 0.5 μg/mL *EmSFRP* antibody in 5% milk/TBST overnight at 4°C, gently rocking. The membrane was then washed with TBST (3 times, 10 min each) and incubated at 1:3000 dilution with goat anti-rabbit IgG H&L HRP-conjugated secondary antibody (abcam) for 1 hour at room temperature, gently rocking. The membrane was washed with TBST (3 times, 10 min each) and then incubated with Clarity Western ECL Substrate for 5 min and imaged with a ChemiDoc Imaging system. Antibody specificity was evaluated by incubating 0.5 μg/mL *EmSFRP* antibody diluted in 10% donkey serum/PBS with excess *EmSFRP* peptide at a 5:1 and 10:1 ratio overnight at 4°C, followed by Western blot analysis as described above using equal amounts of *Ephydatia* protein lysate in no peptide and plus peptide antibody incubations.

### Immunohistochemistry

*E*. *muelleri* sponges were grown from gemmules to juvenile stage in 35 mm petri dishes with a 14 mm #1.5 coverglass bottom (MatTek). The sponges were fixed in 4% paraformaldehyde/¼ Holtfreter’s Solution overnight at 4°C. The next day, the sponges were washed with ¼ Holtfreter’s Solution (3 times, 5 min each) and permeabilized with 0.1% Triton X-100/PBS for 2 min. Following the permeabilization, the sponges were washed with PBS (3 times, 5 min each). The *EmSFRP* antibody was diluted to 4.55 μg/mL in 10% donkey serum/PBS and incubated on the sponges overnight at 4°C, gently rocking. The next day, the sponges were washed with PBS (3 times, 10 min each) and incubated for one hour in the dark and at room temperature with 0.5 μg/mL diluted ALEXA488-conjugated donkey α-rabbit secondary antibody (Jackson Immunochemicals) in 10% donkey serum/PBS with 6.6 nM ALEXA568-Phalloidin (Life Technologies) and 1 μg/mL Hoescht 33342 (Life Technologies). Following the incubation, the sponges were washed with PBS (3 times, 10 min each) and imaged using an Olympus FV1200 laser scanning microscope using FluoView software.

### RNAi knockdown

RNAi by soaking in dsRNA was used to knockdown target gene expression of *EmSFRP* in *E*. *muelleri* tissues based on previously described methods [51–52]. dsRNA was synthesized (Target sequence at position 245 to 841 of mRNA; F: 5’-TGGCCAGCCGATGTTCTAAG-3’; R: 5’-TGATCACCGTTTTGGGAGGT-3’) using the T7 RiboMAX Express RNAi *in vitro* transcription system (Promega) and sponges were treated with 20 ng/μL over the course of 48 hours. Media and dsRNA was replaced daily. Soaking sponges in concentrations of >50 g/mL of dsRNA leads to potentially toxic effects. RNAi efficiency was validated post-RNAi treatment using qRT-PCR [27,51] and *EmSFRP* gene specific primers. Knockdown was also validated by Western Blot analysis as described above, using equal amounts of control and dsRNA treated sponge protein lysates and EmSFRP antibody.

After dsRNA treatment, sponges were washed in Strekal’s media, phenotypes were scored, and sponges were photographed on an Olympus SZX12 stereomicroscope and then either harvested for RNA or mounted for visualization on an Olympus BX61 microscope. Scoring of oscular phenotype was done by counting the number of primary and ectopic oscula every 24 hours over 2 days. Statistical comparison of oscula number between treated and control sponges was performed using Welch’s two sample t-test as implemented in R [53].

*EmPaxB* knockdown proceeded as described above with partial T7 Primers on the ends of *EmPaxB* amplicon (F: 5’-GGCGGGCTTTTCGTCAACGG-3’; R: 5’-TGGAACTGACGGAGGGAACG-3’). Sponges were treated with 10–20 ng/μL over the course of 48–96 hours, where media and dsRNA was replaced daily. Sponges pooled from at least 6 wells (with 2–4 sponges per well) were harvested for each treatment and stored in RNA later (Invitrogen) and frozen at -80°C after removal of the reagent. RNA isolation and qRT-PCR were as described above. Statistical comparison of relative gene expression values between treated and control sponges was performed using Welch’s two sample t-test as implemented in R [53].

## Results

### Downstream targets of PaxB

A computational approach was utilized to determine potential downstream targets of the EmPaxB transcription factor based on the consensus PaxB binding sites (S1 Table) determined through EMSA analysis [27]. PaxB binding sites identified using the FIMO algorithm were mapped onto the *Amphimedon queenslandica* genome and a draft *E*. *muelleri* genome sequence (Fig 1). Likelihood that downstream targets are regulated by EmPaxB were ranked by the compare algorithm based on the number of putative EmPaxB binding sites that were found within 2000 bp of a putative protein coding sequence. The FIMO program determined a cut off for inclusion at sites with a score higher than 10.6, a p-value less than or equal to 9.5 X 10^−5^, and a q value of less than 0.555. We chose five hits as the target number since that number of 15-nucleotide long sequence sites are unlikely to arise by chance in a scaffold. The program returned 138 predicted protein sequences where one protein had 10 possible binding sites nearby, one had nine, four had eight hits nearby, and eleven predicted proteins had seven sites. This was a total of 18 predicted protein sequences which we arbitrarily decided was a good number to focus on. This list of possible regulatory target genes found to have multiple (seven or greater) putative EmPaxB binding sites included several proteins whose roles have been documented in regulating developmental processes in other animals (S2 Table). There were an additional 23 predicted protein sequences with six possible binding sites nearby as well.

One of the top hits, retrieved several times, was a sequence containing a frizzled (fz) domain of the CRD_FZ superfamily. The SFRP target had 8 FIMO hits within 1000 bp of the predicted EmSFRP protein coding sequence. The CRD_FZ is a key domain in several cell surface receptors that are involved in cell signaling pathways, especially the Frizzled receptors that modulate Wnt protein activity in a variety of animal cell types. The CRD_FZ domain is also conserved across metazoans and found in a variety of other proteins including secreted frizzled related proteins (SFRPs), mouse type XVIII collagen (alpha chain), carboxypeptidase Z, some receptor tyrosine kinases, and serine protease corin.

### A novel secreted frizzled related protein in freshwater sponges

**Fig 2:**
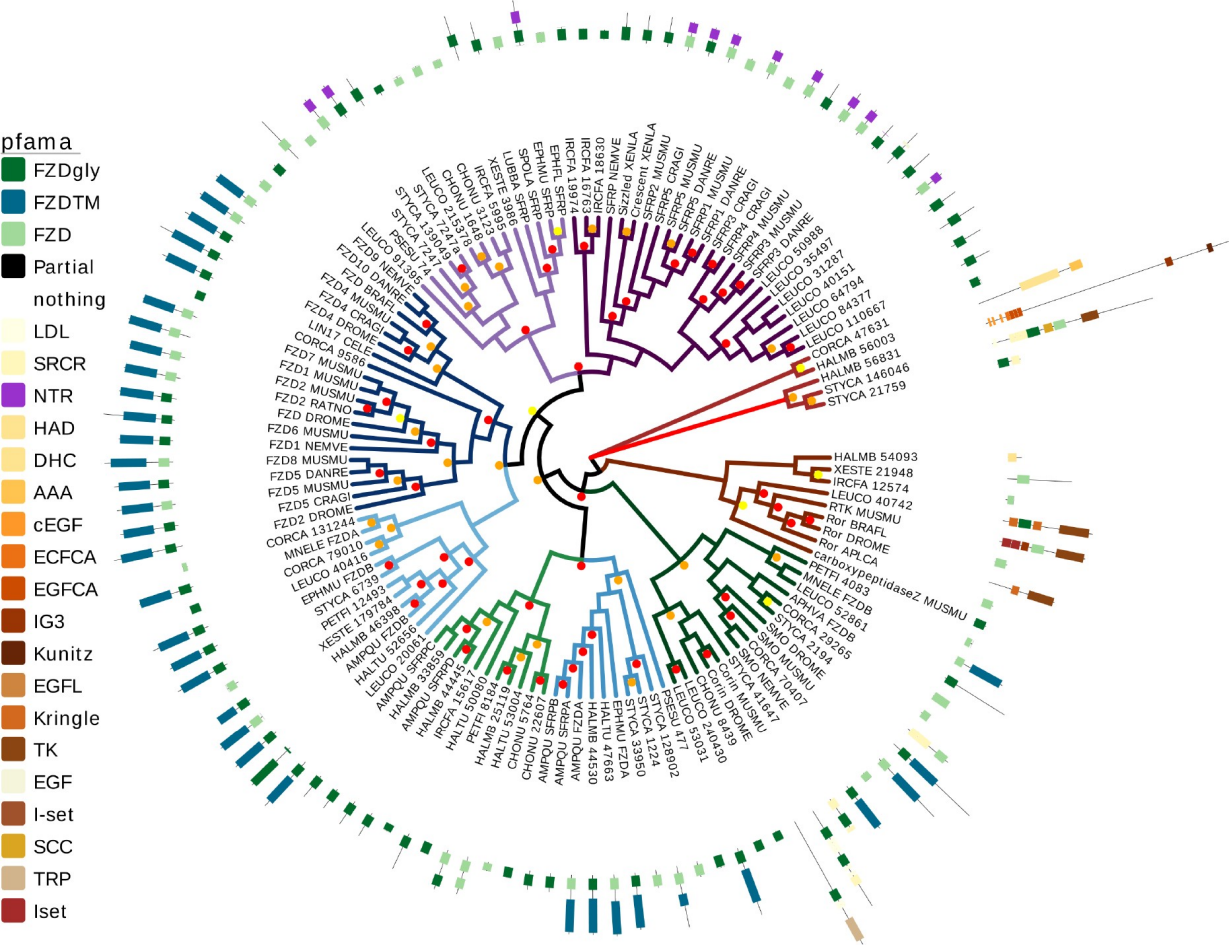
EmSFRP phylogenetic analysis. Tree of SFRP and Frizzled relationships from sponges and other metazoans based on PhyML. The two monophyletic Frizzled groups are shown with branches in blue. A monophyletic group of SFRPs (some with netrin domains) are designated with purple branches, including *Ephydatia muelleri* SFRP (denoted EPHMU SFRP in the phylogeny) and other sponge SFRPs in light purple. Additional SFRPs and other frizzled domain proteins without netrin domains are in green. Node support was run using aLRT and 90–100% supported nodes are designated with a red dot, 76–89% with an orange dot, 65–75% with yellow, and nodes with under 65% are not shown. Pfam domains are given in the key such that Frizzled domains with a predicted glycosylation site are dark green, Frizzled domains without a glycosylation site are light green, netrin domains are purple, and other domains are designated by beige/brown/brick red.

We conducted domain architecture and phylogenetic analysis of the fz domain containing sequence (Fig 2). The target gene contained a cysteine rich (CRD) frizzled domain with a predicted glycosylation site that may be important for Wnt-binding (reviewed in [54]) as well as a netrin-related domain which also may play a role in Wnt signal modulation [55]. Both domains are characteristic of a number of Secreted Frizzled Related proteins in other animals, and given that our putative PaxB fz domain containing target fell into a monophyletic clade with other SFRP genes, we characterize it as a SFRP. The closest related sequence was found be an SFRP from the freshwater sponges *Lubomirskia baicalensis* and *Spongilla lacustris*. We only find one SFRP-like sequence in both the *E*. *muelleri* and *E*. *fluviatilis* draft genomes. Alignment analysis showed that the CRD_FZ domain in *E*. *muelleri* was largely canonical with 10 Cysteines, and a basic (Lysine) residue following C6. The typical Proline 4 residues C-terminal to C9 is not present in EmSFRP, nor is it found in *A*. *queenslandica* SFRPC or FZD6, in *Nematostella vectensis* SFRP, or in *Mmemiopsis leidyi* FZDA (S6 File). In a PhyML tree using the CRD_FZ domain, these four, freshwater sponge SFRPs cluster with predicted SFRPs from seven other sponge species to form a monophyletic sister group to the eumetazoan SFRP clade. Together the sponge and eumetazoan SFRP sequences form a well-supported phylogenetic clade by PhyML analysis (Fig 2). IQtree analysis of full-length sequence using abayes to determine support also recovered a moderately-supported monophyletic clade containing the SFRPs from both *Ephydatia* species and all eumetazoan SFRPs (S1 and S2 Figs). Interestingly, both analyses grouped *A*. *queensladica* SFRPs with eumetazoan and *E*. *muelleri* FZs, suggesting they may be more closely related to FZs than canonical SFRPs.

### Expression of SFRP during E. muelleri development from gemmules

**Fig 3:**
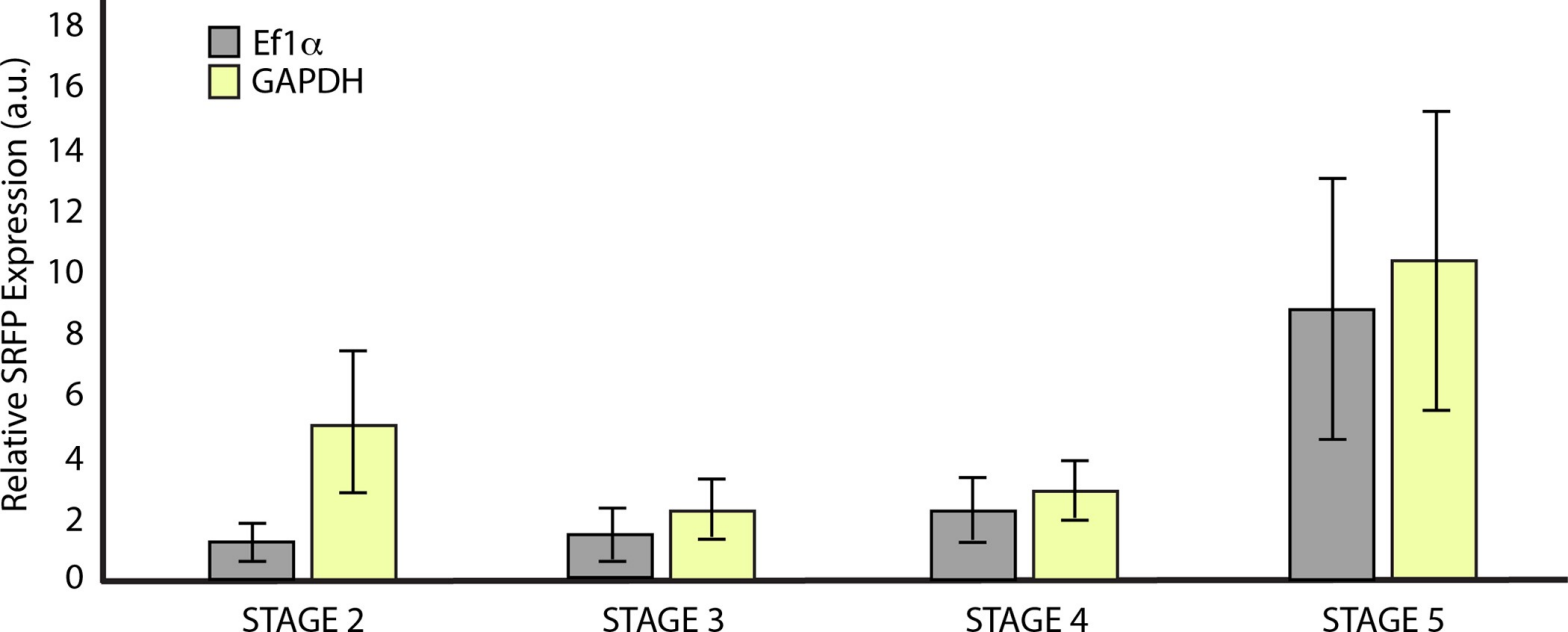
Relative expression levels of *EmSFRP* across asexual developmental stages of gemmulating *E*. *muelleri*. Relative *EmSFRP* expression (a.u. = arbitrary units) levels were normalized to Ef1α and GAPDH, averages (± SEM) are shown. A one-way ANOVA indicated no significant differences among stages (Ef1α: F_3,4_ = 5.012, p = 0.077; GAPDH: F_3,4_ = 3.33; p = 0.138). Developmental stage diagrams and pictures reprinted from [28] under a CCC license, with permission from Elsevier, original Copyright, 2016.

**Fig 4:**
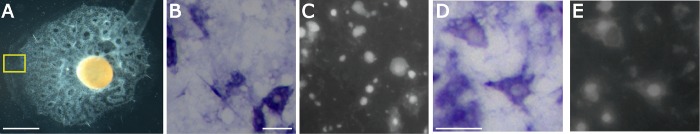
*EmSFRP* expression in amoeboid cells with filipodia in mesohyl at periphery of stage 5 sponge. A) Stage 5 juvenile sponge with functional aquiferous system. Scale: 1 mm. B&D) In situ hybridization of *EmSFRP* with staining in a subset of amoeboid cells with filipodia in the mesohyl of the pinacoderm. Multiple morphologies are shown, but all *EmSFRP* positive cells are amoeboid in shape. Region of sponge where photos were taken is shown in yellow box in A. C&E) Dapi staining of sections shown in B and D show locations of staining and not staining cells. Scales: 20 μM.

**Fig 5:**
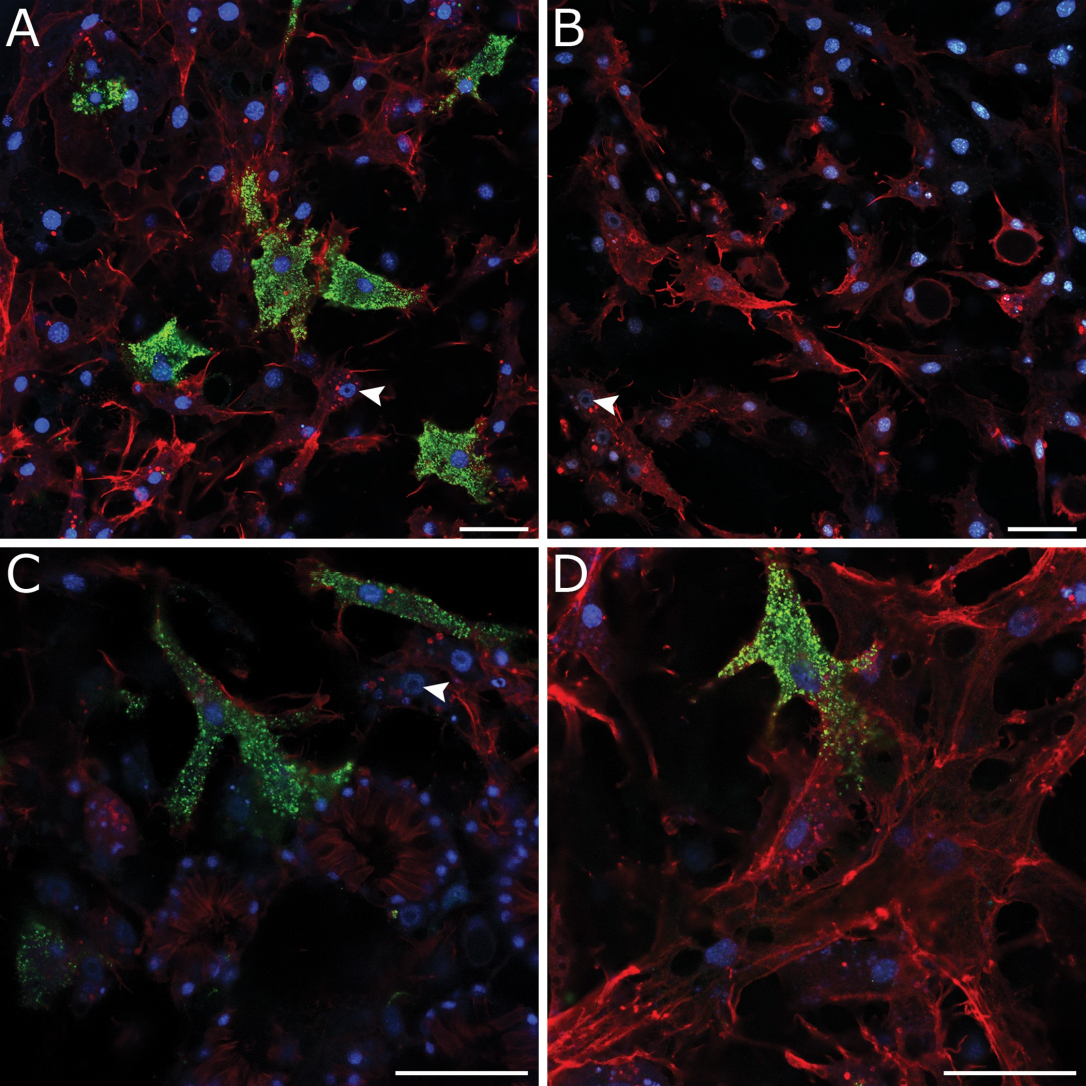
EmSFRP protein localization in amoeboid cells in mesohyl of pinacoderm and choanoderm. A) EmSFRP found in subset of amoeboid cells with filipodia in mesohyl. Other amoeboid cells containing nucleolated nuclei (white arrowhead) are shown. B) Antibody control shows no background staining. C) Closer magnification showing EmSFRP localized in amoeboid cell abutting choanocyte chamber (top center) and another next to amoeboid cells containing nucleolated nuclei (white arrowhead; likely archeocytes; top right). D) EmSFRP localization in amoeboid cell in basal pinacoderm next to other amoeboid and connective cells. Images show DNA in blue, anti-EmSFRP in green, and F-actin in red. Scales: 20 μm.

We assayed relative gene expression levels for *EmSFRP* across four developmental stages [28] to see if *EmSFRP* is turned on during development. *EmSFRP* is expressed after gemmule hatching and throughout early development of the sponge body. Expression seems to increase in Stage 5 sponges when the aquiferous system is functional (Fig 3). Given the level of expression at Stage 5, we performed in situ hybridization to localize expression in sponge tissue/cells and found that *EmSFRP* is expressed in a distinct population of filipodia possessing amoeboid cells in the mesohyl between the endopinacoderm and the basal pinadoderm layers (Fig 4 & S3 Fig). The cells expressing *EmSFRP* are morphologically similar to cells that express WntA in that same region (S3 Fig, also see [29]), but distinct from cells in that region that were shown to express *EmPaxB* (S3 Fig, also see [27]). To determine if EmSFRP protein is also present in these migrating cells, we raised a custom antibody against an 18 aa peptide in the CRD region of EmSFRP, avoiding the highly conserved FZD domain to reduce the chance of antibody binding to other frizzled related receptors. The resulting antibody was affinity purified and ELISA tested and its specificity further tested by Western Blot against whole-cell protein lysates where a ~55 kDa band of expected size was detected and a blocking peptide comprised of the amino acid sequence corresponding to the antibody epitope, bound specifically to the target antibody and prevented the antibody binding the EmSFRP epitope (S4 Fig). By immunostaining with the EmSFRP antibody, we found that EmSFRP localizes in the same population of amoeboid cells with filipodia in the mesohyl of the pinacoderm as we observed for *EmSFRP* mRNA. Additionally, confocal microscopy revealed that these EmSFRP staining cells are also found throughout the mesohyl of the choanoderm, often in direct contact with choanocyte chambers and other cells of the choanodermal region including amoeboid cells with nucleolated nuclei which are likely archeocytes (Fig 5 & S5 Fig).

### Knockdown of SFRP expression leads to ectopic oscula formation

**Fig 6:**
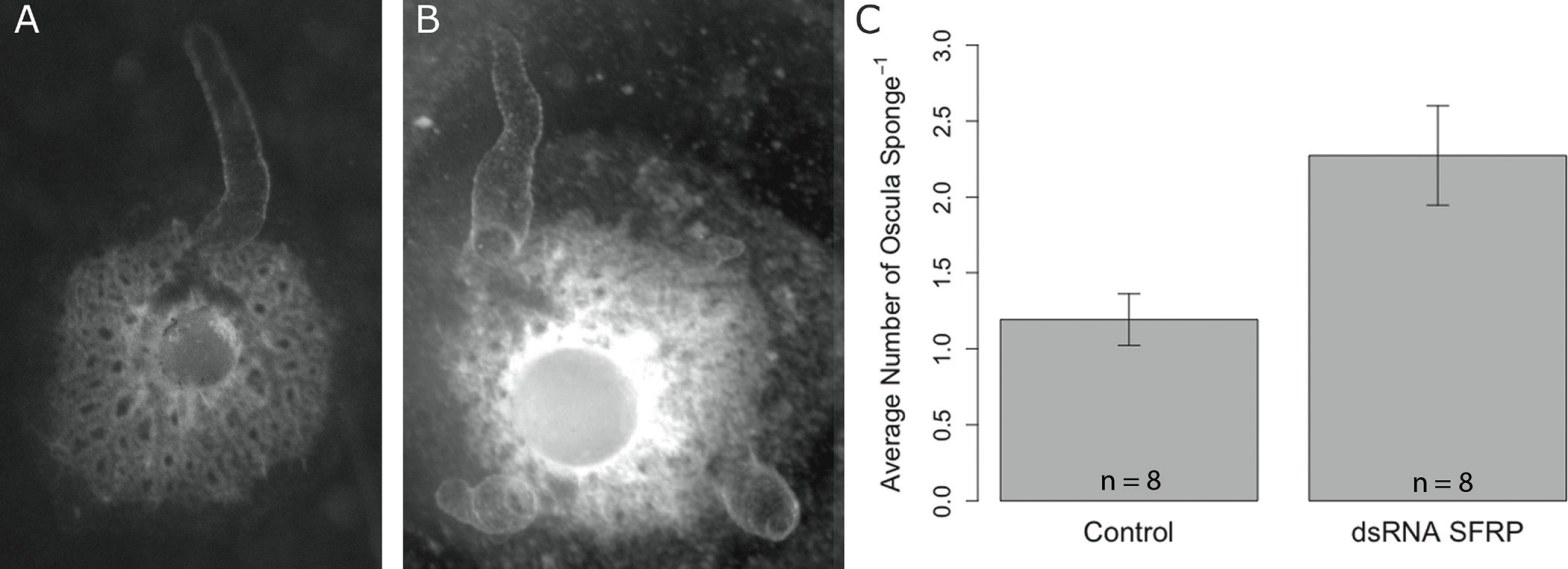
EmSFRP knockdown with dsRNA causes multiple oscula. A) Representative control sponge phenotype with a single primary osculum. B) Representative *EmSFRP* knockdown sponge exhibiting multiple oscula. C) Eight repeated trials show average oscula number (± SEM) per sponge in treated and control sponges. Welch’s two sample t-test indicated that the treatment group had significantly more oscula than the control (t_11.6_ = -2.403, p = 0.034).

To determine if *EmSFRP* plays a role in aquiferous system development as observed with other components of the Wnt signaling pathway in *E*. *muelleri*, we treated sponges hatched from gemmules with dsRNA directed to *EmSFRP* over the course of 48 hours. The average reduced expression of *EmSFRP* after RNAi treatment was only ~40%, which is within the expected range of 30–50% for RNAi in freshwater sponges [51]. Furthermore, Western Blot analysis confirmed a decrease in EmSFRP protein levels after dsRNA treatment (S6 Fig). Nonetheless, while the mRNA knockdown efficiency is moderate, the dsRNA treated sponges often developed multiple oscula and repeated experiments demonstrated that partial knockdown of *EmSFRP* led to significant differences in the number of oscula observed in treated compared to control sponges (Fig 6). This oscular phenotype is not observed with knockdown of other target genes (e.g., PaxB, Six ½ [27], MBD2 [28], silicatenin [29]), but is observed with knockdown of GSK3β [29]. Other than the development of multiple ectopic oscula and resulting canals, the RNAi treated sponges were healthy and did not have any other apparent phenotypic differences. The additional oscula, when fully formed, were functional in all cases observed.

### EmPaxB regulation of EmSFRP

**Fig 7:**
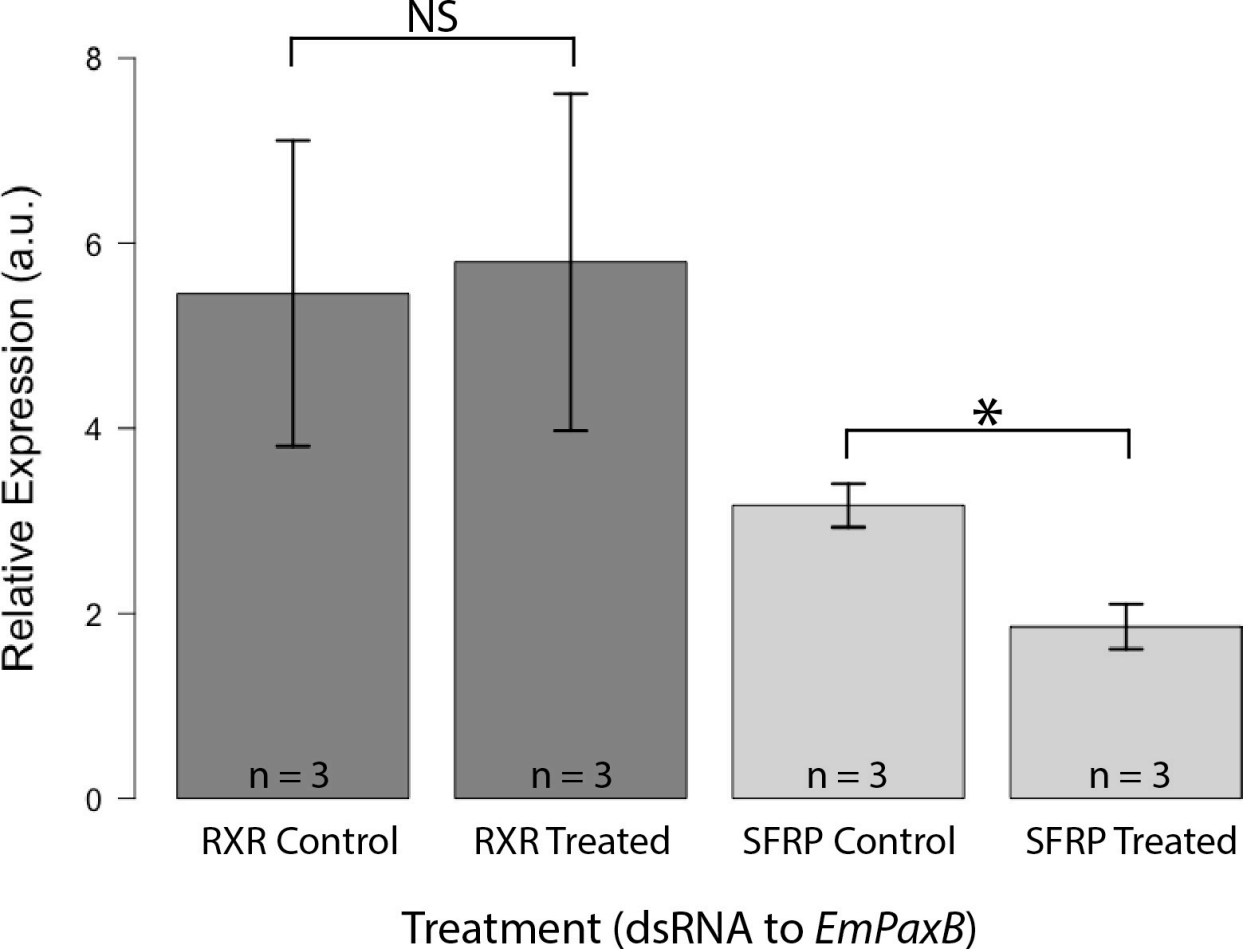
EmSFRP expression is decreased in sponges treated with dsRNA to EmPaxB. Relative expression (a.u. = arbitrary units) levels of *EmRXR* and *EmSFRP* were normalized to Ef1α, averages (± SEM) are shown. 48-hour control and treated sponges showed no significant difference in *EmRXR* expression when treated with dsRNA to *EmPaxB* (t_3.96_ = -0.138, p = 0.897). Whereas, control and treated sponges showed significant differences in *EmSFRP* expression when treated with dsRNA to *EmPaxB* (t_3.99_ = 3.9, p = 0.018).

To test the hypothesis that EmPaxB regulates expression of *EmSFRP*, sponges hatched from gemmules were treated with dsRNA for *EmPaxB* every 24 hours and were harvested after 48 and 96 hours. qRT-PCR analysis was used to determine if knockdown of *EmPaxB* expression would lead to decreased or increased expression of *EmSFRP*. Evaluation of relative gene expression levels after *EmPaxB* RNAi revealed that *EmSFRP* expression is decreased after knockdown in treated compared to control sponges after 48 hr of exposure to dsRNA (Fig 7). Another putative *EmPaxB* target gene, *EmRXR (retinoic acid receptor)*, did not show significant changes in expression after treatment with dsRNA to *EmPaxB* for the same time frame (Fig 7). Sponges treated for longer periods of time with dsRNA to EmPaxB (refreshed every 24 hours) continued to exhibit decrease expression of EmSFRP (S7 Fig). The average knockdown of *EmPaxB* for these experiments was approximately 60%. These results imply that EmPaxB may regulate expression of *EmSFRP* via the identified PaxB binding sites located upstream of the *EmSFRP* coding sequence in *E*. *muelleri*, though further investigation with EmPaxB protein would be necessary to validate this finding.

## Discussion

One of our overarching goals is to understand how conserved and uniquely metazoan gene regulatory networks function in early branching, non-bilaterian animals. This understanding may lead to insights into how these molecular pathways have contributed to the diversity of morphologies observed in animals. Our search for putative sponge-specific PaxB binding sites in the *Ephydatia muelleri* genome was originally focused on investigating downstream targets of the Pax/Six network. While that search revealed a host of possible PaxB targets that included genes with enzymatic function, membrane receptors, secreted proteins, and cell signaling components, we were interested to find that one PaxB target contained a putative frizzled domain and might be a member of the Wnt signaling pathway (S1 Table). This was of distinct interest because we hypothesized that a connection between the PaxB pathway to the Wnt pathway in sponges might represent a conserved module in animal development and evolution. For example, it is known that Pax6 regulates expression of SFRP2 and Wnt7b in mouse nervous system development [56] and Pax2 regulates SFRP2 in embryonic kidney cells [57]. Further, earlier work indicated a role for both the Pax/Six gene network [27] and the Wnt signaling pathway [14] in establishing the freshwater sponge aquiferous system. Thus, the finding that a secreted frizzled related protein contained eight possible upstream PaxB binding sites directed our efforts to examining the role of this protein in freshwater sponge development.

*EmSFRP* is expressed in juvenile sponges in a subpopulation of amoeboid cells in the mesohyl both at the periphery of the sponge and within the choanoderm. The EmSFRP staining cells possess cytoplasmic extensions that project in multiple directions suggesting that these cells are actively moving throughout the mesohyl. The morphology and locations of the cells suggested that they could be archeocytes (stem cells), however, we do not routinely observe that these cells contain a single large nucleolus and phagosomes (hallmark feature of archeocyte stem cells) as some of their neighboring amoeboid cells possess (See Fig 5, white arrowheads, and S5 Fig). However, some of those features could be obscured by EmSFRP staining as it is clear that some of the EmSFRP expressing cells have inclusions that could be phagosomes (see S5 Fig). Furthermore, given that nucleolus size can be an indicator for ribosome biogenesis and that nucleolar size can reduce during cell fate specification (e.g., [58]), it could be that the EmSFRP positive cells are, indeed, archeocytes in a state of differentiation. Sponge stem cells (archeocytes) in the closely related freshwater sponge *Ephydatia fluviatilis* possess a large nucleus containing a single large nucleolus and many phagosomes, as well as express homologs to *Piwi* and *Musashi* [59,60]. Thus, further studies looking for co-expression of Piwi and/or *Musashi* with *EmSFRP* are necessary to determine if *EmSFRP* staining cells are archeocytes. The alternative hypothesis that *EmSFRP* positive cells are differentiating archeocytes also requires further analysis as there are no studies tracing archeocyte cell fate with molecular markers in this species.

A recent study, Windsor et al. [29] showed that *E*. *muelleri* WntA, WntB, and WntC are expressed in subpopulations of amoeboid cells with filipodia in the freshwater sponge mesohyl and we confirm those results for *EmWntA* (S2 Fig). Furthermore, β-catenin, another component of the Wnt signaling pathway, was recently shown to be expressed in amoeboid cells of the mesohyl in *E*. *muelleri* [29–30], though Windsor et al. described the β-catenin expressing cells as lacking thin filopodial-like projections seen in cells labelled by their Wnt probes [29]. In both cases, the amoeboid cells expressing Wnt and β-catenin are described by the authors as archeocytes (actively migrating cells in the mesohyl), and in Schippers and Nichols [30], these cells are referred to as a stem cell lineage. It is difficult, however, to determine if Wnt or β-catenin expressing cells in either of those studies contain the single large nucleolus that defines archeocytes. It was further suggested that the expression of Wnt pathway members (β-catenin and Wnt) in freshwater sponge archeocyte cells might indicate a possible conserved role for Wnt signaling in stem cell maintenance, self-renewal, and/or differentiation [30]. Our finding that *EmSFRP* is expressed in a subpopulation of cells that look like the cells expressing other Wnt pathay genes would support this assertion, however, further studies are needed to determine if the subpopulation of amoeboid (archeocyte-like) cells that express *EmSFRP*, *EmWnts*, and *Emβ-catenin* are indeed sponge totipotent stem cells or some differentiated, yet amoeboid state of the archeocyte. Finally, given that the antibody staining for *EmSFRP* appears to be vesicular in nature, it may be that EmSFRP is released from these amoeboid cells to signal other sponge cells to initiate morphogenetic processes. In support of this idea, Fig 5C shows an EmSFRP expressing archeocyte in direct contact with multiple choanocytes of a choanocyte chamber.

When sponges were treated with pharmacological agents LiCl or alsterpaullone, previous studies showed that inhibition of GSK3 protein, a negative regulator of Wnt signaling, led to loss of canals, reduced canals, no ostia formation, and formation of ectopic oscula [10]. The lack of canal formation and absence of a choanosome was also reported with treatment using the GSK3 inhibitor BIO [30]. Knockdown of GSK3 expression by RNAi in *E*. *muelleri* also lead to the production of additional ectopic oscula, but not inhibition of canal formation, presumably because RNAi knockdown is partial (~30%) whereas pharmacological inhibition is likely more complete [29]. Activation of the Wnt pathway after GSK3 inhibition presumably leads to defects of the canal system in this freshwater sponge. Schippers and Nichols [30] recently demonstrated that Emβcatenin is a conserved substrate of GSK3β, and while they also discovered new functions for Emβcatenin in cell adhesion, their data support conserved functions with bilaterians in Wnt signaling. It thus seems likely that inhibition of GSK3β specifically results in activation of the Wnt/β-catenin pathway.

Here we show that knockdown of SFRP expression by RNAi leads to formation of multiple oscula as was observed with knockdown of GSK3 in this species. This suggests that SFRP is acting as a Wnt antagonist in *E*. *muelleri*, perhaps by binding directly to Wnts as some SFRPs are known to do in other metazoan systems (e.g., [61]). This is supported by our finding that SFRP is also expressed in amoeboid cells with filipodia in *E*. *muelleri* as was observed for *Wnt A*, *B*, and *C* [29]. Schippers and Nichols [30] postulated that sustained activation of the Wnt/B-catenin pathway may lead to inhibition of differentiation of archeocytes to choanocytes. It may also follow that lower levels of Wnt activation that result from RNAi treatment with GSK3 and SFRP lead to changes in signaling that result in a ‘disorganized’ body plan (i.e., ectopic oscula), but not a failure to form canals and choanosome. While we detected quantifiable differences in *EmSFRP* protein levels after RNAi knockdown, the knockdown may be transient or the SFRP protein may be highly stable or have a long half-life. The finding that inhibition of positive regulators of Wnt signaling (e.g., niclosamide or quercetin) block the formation of oscula and oscular regeneration further supports role of Wnt signaling in body plan organization [29].

This study supports the hypothesis that the regulation of Wnt signaling by Pax family members may predate the divergence of sponges. We show that a freshwater sponge Secreted Frizzled Related Protein may be regulated by EmPaxB in *E*. *muelleri*, and that EmSFRP likely plays a role as a regulator of Wnt signaling in freshwater sponge morphogenesis of the aquiferous system. Future lines of research directed at critically evaluating the binding interactions of both Pax/Six network and Wnt signaling pathway components are necessary to determine if, and the degree to which, these regulatory modules are conserved throughout metazoan evolution. More targeted biochemical analysis, and in vivo determination of developmental functions, of these pathways will help to further elucidate how these metazoan gene regulatory networks function in early branching animals, and how these functions compare to roles that they play in more complex animals.

## Supporting information

S1 Fig PhyML phylogeny. PhyML tree with aLRT support values in red and aBAYES support from IQ-TREE in black. Values in brackets are conflicting nodes (i.e., nodes found in PhyML tree but not in the IQ-TREE).
(PDF)

S2 Fig IQ-TREE phylogeny. aBAYES support values are shown in red and aLRT support values black. Values in brackets are conflicting nodes between PhyML and IQ-TREE analyses.
(PDF)

S3 Fig Regional expression of EmSFRP, EmWntA, and EmPaxB. A) In situ hybridization of *EmSFRP* in Stage 5 juvenile sponge showing region between choanoderm (top left) and periphery of sponge growth (bottom right) where filipodia possessing amoeboid cells in the mesohyl between the endopinacoderm and the basal pinadoderm layers express *EmSFRP* (see black arrowhead). Inset shows entire Stage 5 sponge with EmSFRP probe. B) Sense probe control for *EmSFRP*. C) *EmWntA* expression in filipodia possessing amoeboid cells in the mesohyl between the endopinacoderm and the basal pinadoderm layers (see black arrowhead). D) *EmPaxB* expression in subset of cells at periphery of sponge growth in endopinacoderm/basal pinacoderm region (black arrowhead shows amoeboid cell with filipodia does not stain for *EmPaxB*; reprinted from [23] under a CCC license, with permission from John Wiley and Sons, original Copyright, 2013. Scales: 200μM.
(TIFF)

S4 Fig EmSFRP antibody specificity. Western Blot analysis of *E*. *muelleri* whole-cell protein lysate with EmSFRP antibody in the absence or presence of EmSFRP antigen. Lane 1: MW marker, lane 2: anti-EmSFRP, lane 3: MW marker, lane 4: anti-EmSFRP with blocking peptide. Arrowhead indicates location of EmSFRP protein.
(TIFF)

S5 Fig EmSFRP protein localization in amoeboid cells. A) Non-staining amoeboid cell with filipodia and inclusions, but not a single large nucleolus. B) Non-staining amoeboid cell with filipodia, inclusions, and a single large nucleolus. C) EmSFRP staining amoeboid cell with filipodia and inclusions, but not a single large nucleolus. Images show DNA in blue, anti-EmSFRP in green, and F-actin in red. Scales: 20 μm.
(TIFF)

S6 Fig EmSFRP protein levels post-RNAi. Western Blot analysis of whole-cell protein lysate from control sponges and sponges treated with dsRNA to *EmSFRP* detected with EmSFRP antibody. Lane 1: MW marker, lane 2: EmSFRP dsRNA treated tissue, lane 3: control tissue. Arrowhead indicates location of EmSFRP protein.
(TIFF)

S7 Fig *EmSFRP* expression is decreased in sponges treated with dsRNA to *EmPaxB*. Relative expression (a.u. = arbitrary units) levels of *EmSFRP* were normalized to Ef1α, averages (± SEM) are shown after 96 hour treatment with dsRNA directed to *EmPaxB*. A two sample t-test indicated significant differences in *EmSFRP* expression between control and sponges treated with dsRNA for *EmPaxB* (t_2_ = 5.5114, p < 0.05).
(TIFF)

S1 Table EmPaxB binding sites. (PDF)

S2 Table Putative PaxB target genes identified by FIMO. (PDF)

S1 File MEME position specific probability matrix. (TXT)

S2 File Compare FIMO scripts. (TXT)

S3 File Optparse FIMO scripts. (TXT)

S4 File FIMO genome scaffolds. (RTF)

S5 File CRD alignment. Alignment of the cysteine rich domain for *Ephydatia*, *Amphimedon*, and selected bilaterian SFRP/FRZ genes. Epmu_SFRP has the 10 cysteines, a basic (Lysine) residue following C6, but is missing a Proline 4 residues C-terminal to C9. *Amphimedon* SFRPC and FZD6 are also missing this proline as is Nematostella SFRP. Additionally, the Proline is 5 residues from C9 in *Amphimedon* FRZB. Not shown in this picture is that the proline is also missing in *Mnemiopsis* FzdA and several other sponge sequences.
(PNG)

S6 File Master alignment. Aligned Fasta file of all sequences used to build phylogenies.
(TRE)

S7 File FRZ alignment. Alignment of only the sequences that fell into the frizzled clade in the ML tree.
(TRE)

S8 File SFRP alignment. Alignment of only the sequences that fell into the SFRP clade in the ML tree.
(TRE)

S9 File IQ tree master alignment. Text tree file generated by IQ-TREE.
(TRE)
