# Supplementary figures and images for: Secreted frizzled related protein is a target of PaxB and plays a role in aquiferous system development in the freshwater sponge, Ephydatia muelleri
Source: PLoS One. 2019 Feb 22;14(2):e0212005. doi: 10.1371/journal.pone.0212005 (PMC6386478; doi:10.1371/journal.pone.0212005)

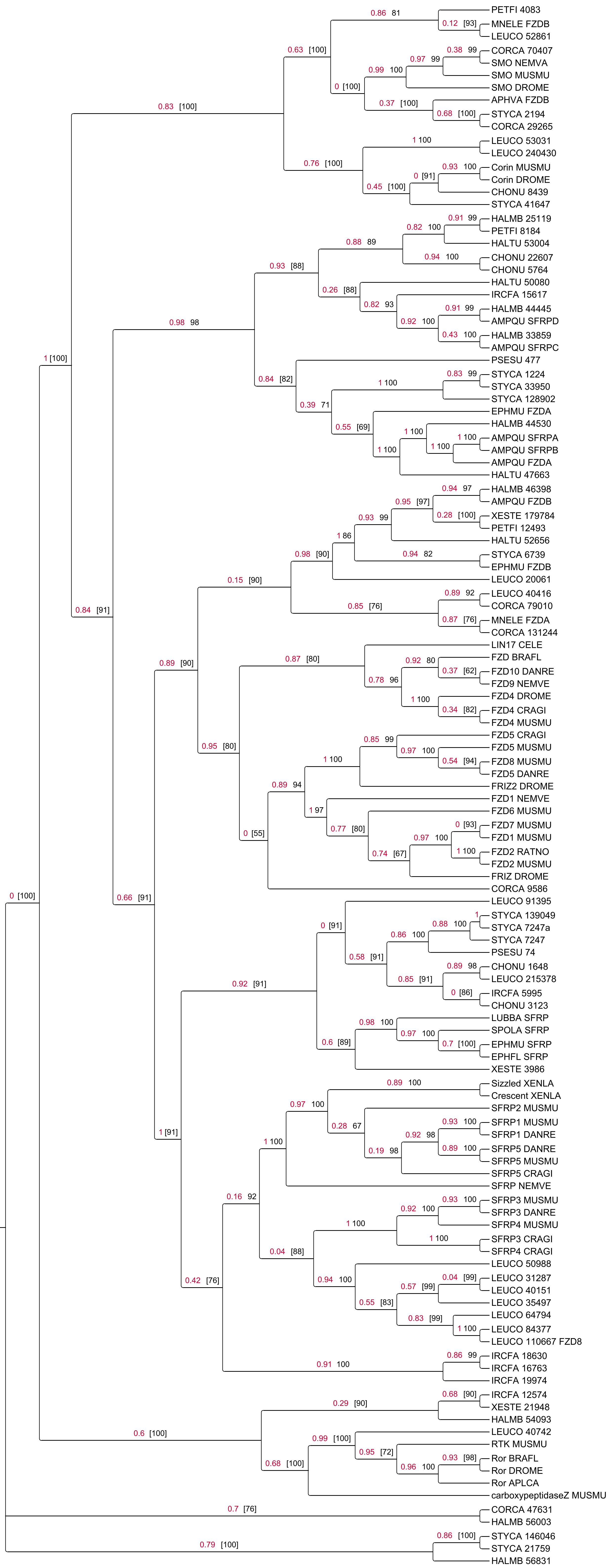

Supplement: S1 Fig — PhyML tree with aLRT support values in red and aBAYES support from IQ-TREE in black. Values in brackets are conflicting nodes (i.e., nodes found in PhyML tree but not in the IQ-TREE). (PDF) [file pone.0212005.s001.pdf]

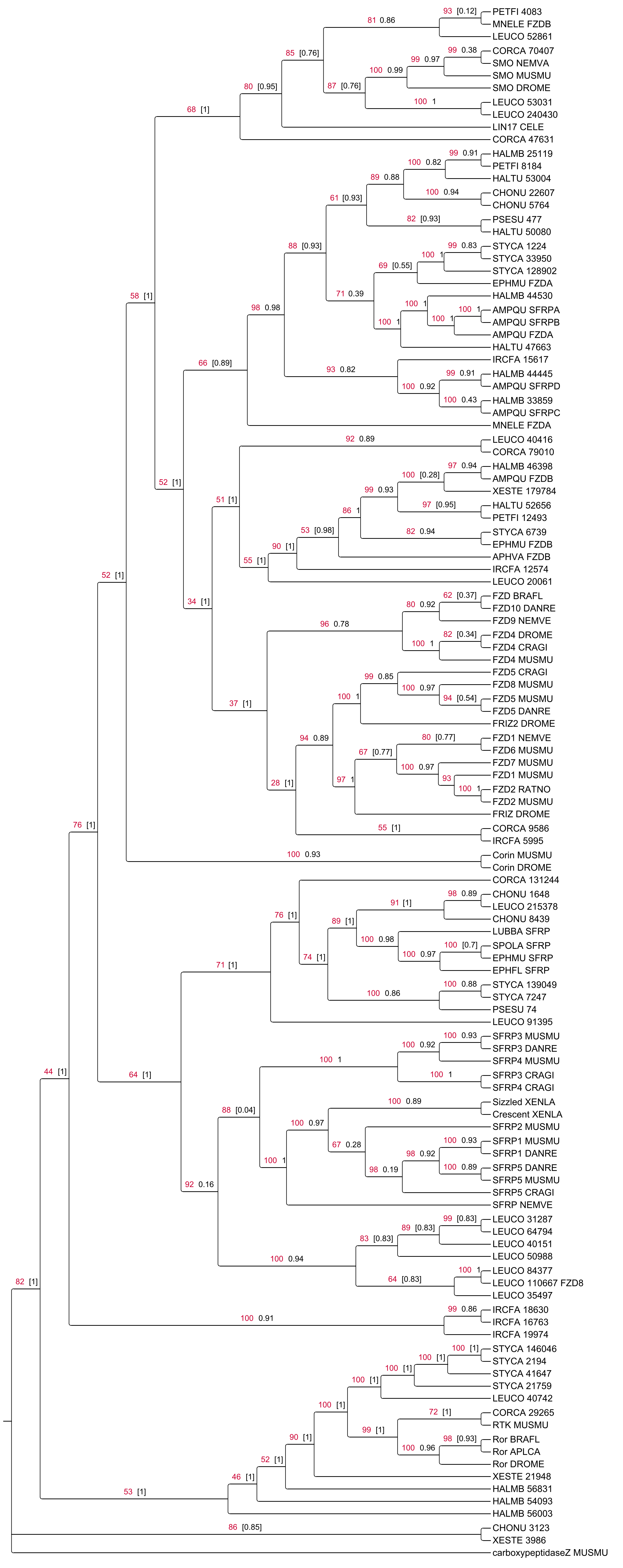

Supplement: S2 Fig — aBAYES support values are shown in red and aLRT support values black. Values in brackets are conflicting nodes between PhyML and IQ-TREE analyses. (PDF) [file pone.0212005.s002.pdf]

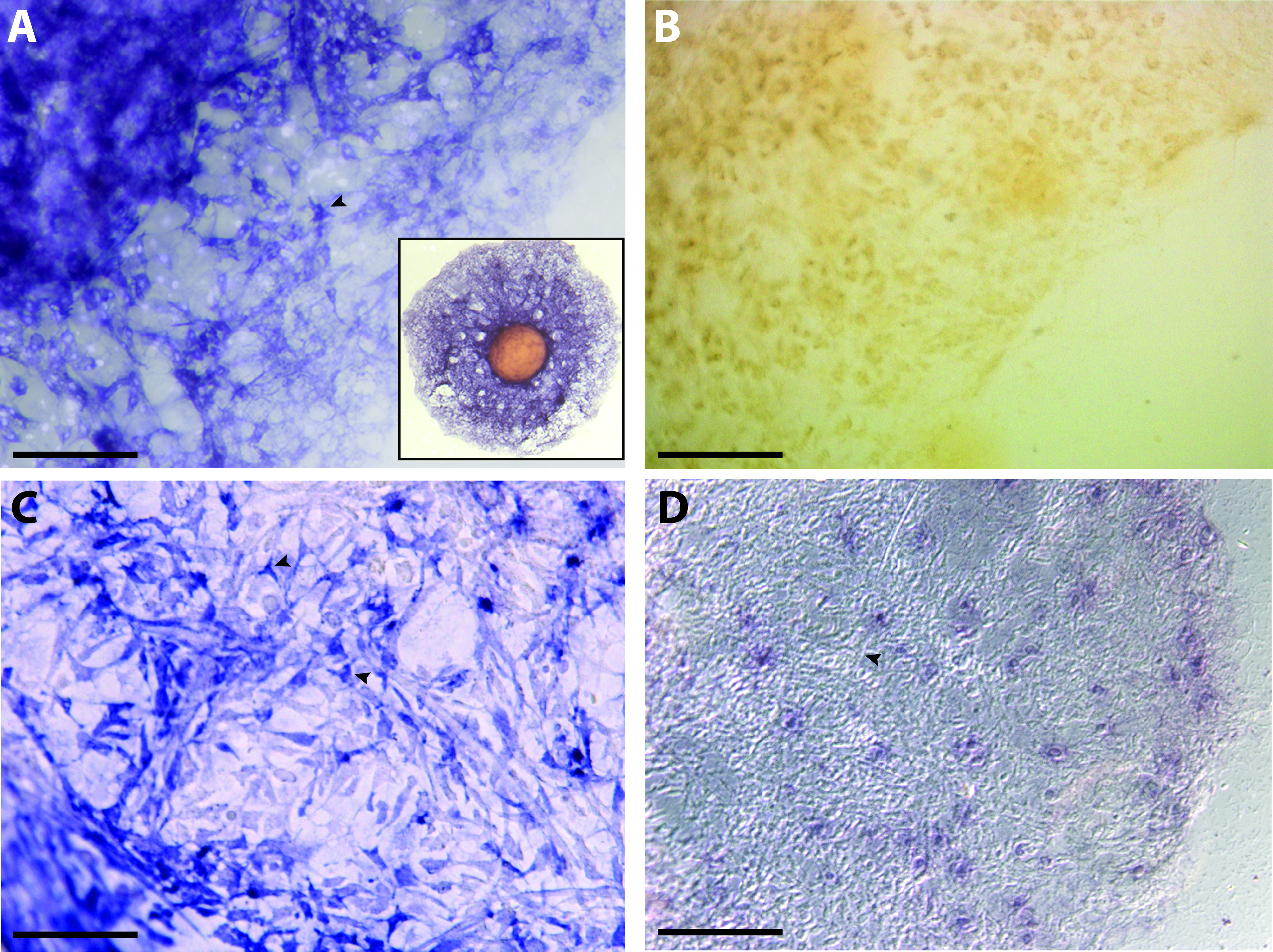

Supplement: S3 Fig — A) In situ hybridization of EmSFRP in Stage 5 juvenile sponge showing region between choanoderm (top left) and periphery of sponge growth (bottom right) where filipodia possessing amoeboid cells in the mesohyl between the endopinacoderm and the basal pinadoderm layers express EmSFRP (see black arrowhead). Inset shows entire Stage 5 sponge with EmSFRP probe. B) Sense probe control for EmSFRP. C) EmWntA expression in filipodia possessing amoeboid cells in the mesohyl between the endopinacoderm and the basal pinadoderm layers (see black arrowhead). D) EmPaxB expression in subset of cells at periphery of sponge growth in endopinacoderm/basal pinacoderm region (black arrowhead shows amoeboid cell with filipodia does not stain for EmPaxB; reprinted from [23] under a CCC license, with permission from John Wiley and Sons, original Copyright, 2013. Scales: 200μM. (TIFF) [file pone.0212005.s003.tiff]

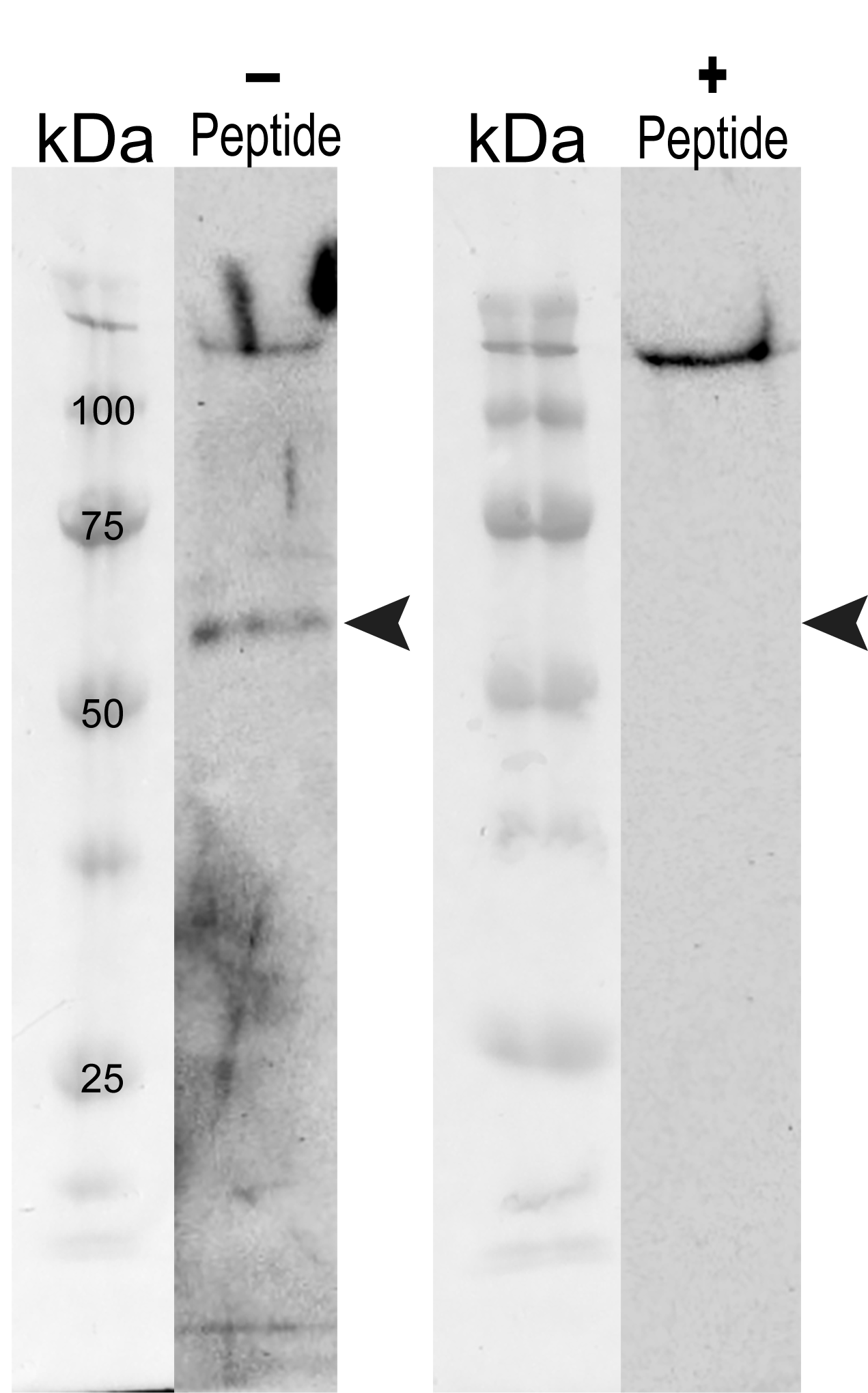

Supplement: S4 Fig — Western Blot analysis of E. muelleri whole-cell protein lysate with EmSFRP antibody in the absence or presence of EmSFRP antigen. Lane 1: MW marker, lane 2: anti-EmSFRP, lane 3: MW marker, lane 4: anti-EmSFRP with blocking peptide. Arrowhead indicates location of EmSFRP protein. (TIFF) [file pone.0212005.s004.tiff]

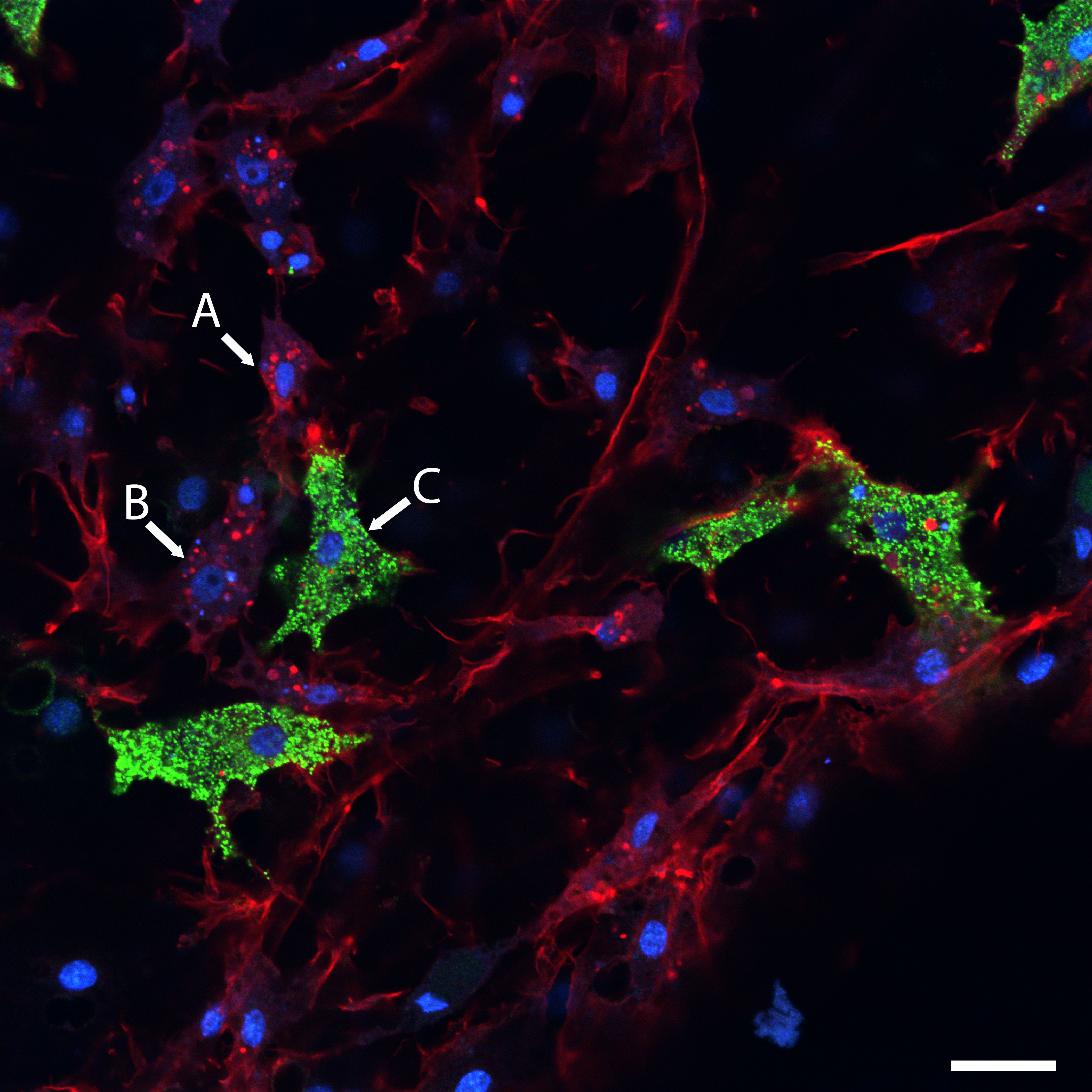

Supplement: S5 Fig — A) Non-staining amoeboid cell with filipodia and inclusions, but not a single large nucleolus. B) Non-staining amoeboid cell with filipodia, inclusions, and a single large nucleolus. C) EmSFRP staining amoeboid cell with filipodia and inclusions, but not a single large nucleolus. Images show DNA in blue, anti-EmSFRP in green, and F-actin in red. Scales: 20 μm. (TIFF) [file pone.0212005.s005.tiff]

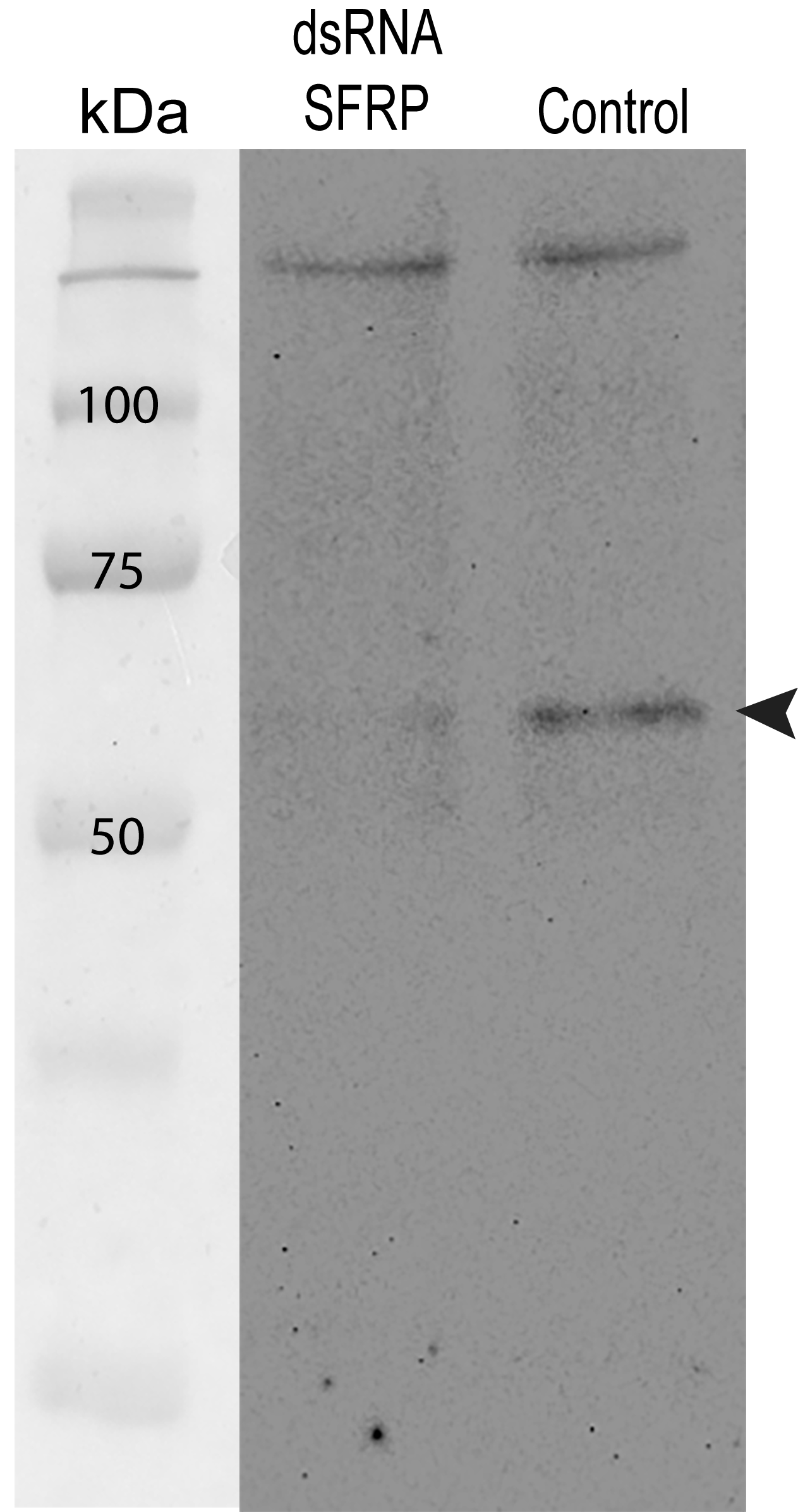

Supplement: S6 Fig — Western Blot analysis of whole-cell protein lysate from control sponges and sponges treated with dsRNA to EmSFRP detected with EmSFRP antibody. Lane 1: MW marker, lane 2: EmSFRP dsRNA treated tissue, lane 3: control tissue. Arrowhead indicates location of EmSFRP protein. (TIFF) [file pone.0212005.s006.tiff]

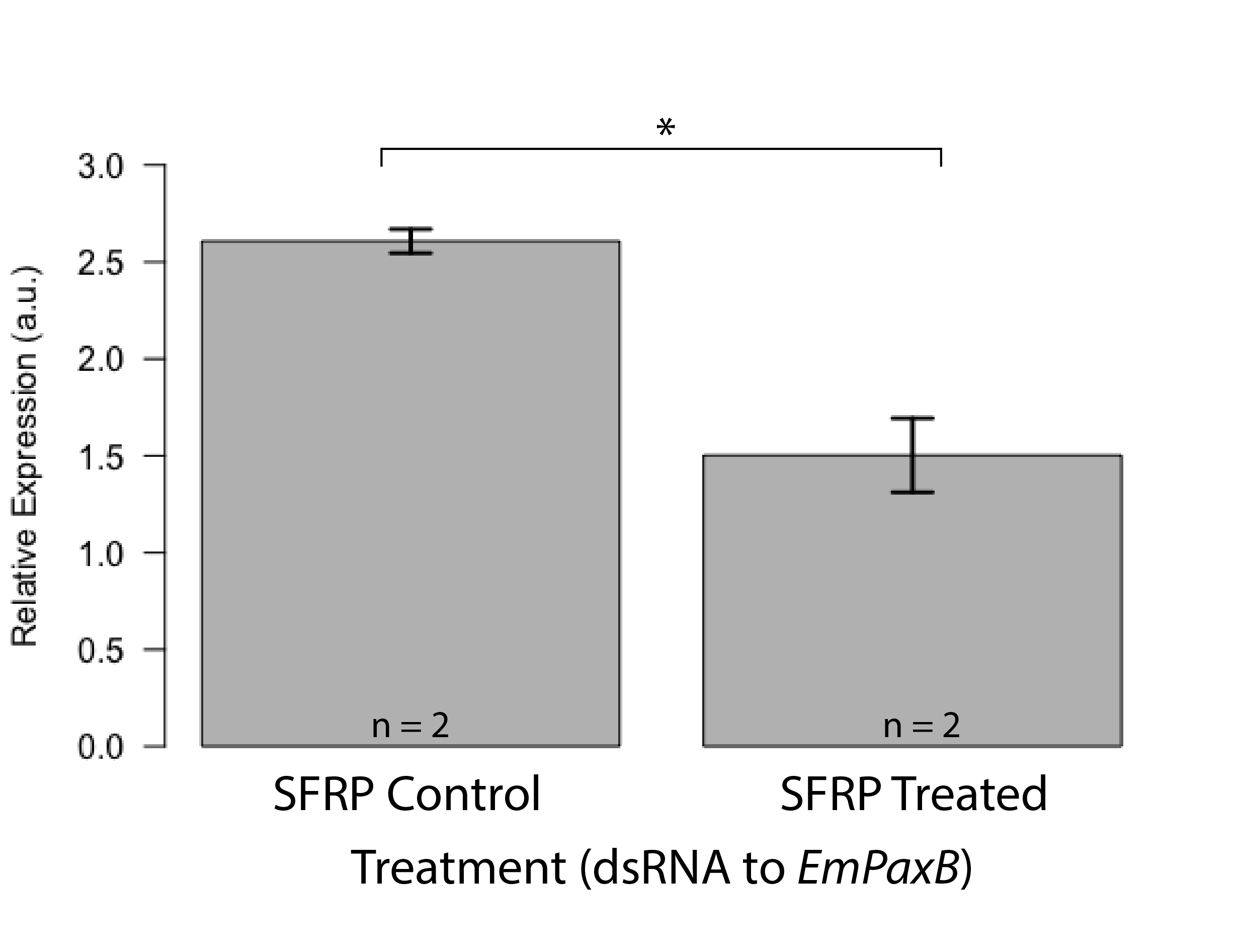

Supplement: S7 Fig — Relative expression (a.u. = arbitrary units) levels of EmSFRP were normalized to Ef1α, averages (± SEM) are shown after 96 hour treatment with dsRNA directed to EmPaxB. A two sample t-test indicated significant differences in EmSFRP expression between control and sponges treated with dsRNA for EmPaxB (t2 = 5.5114, p < 0.05). (TIFF) [file pone.0212005.s007.tiff]

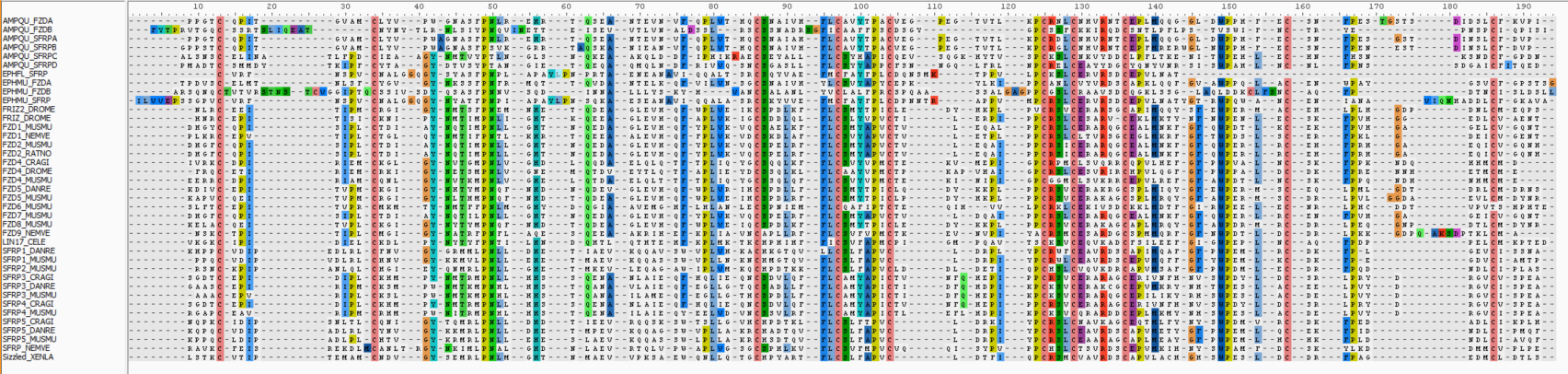

Supplement: S5 File — Alignment of the cysteine rich domain for Ephydatia, Amphimedon, and selected bilaterian SFRP/FRZ genes. Epmu_SFRP has the 10 cysteines, a basic (Lysine) residue following C6, but is missing a Proline 4 residues C-terminal to C9. Amphimedon SFRPC and FZD6 are also missing this proline as is Nematostella SFRP. Additionally, the Proline is 5 residues from C9 in Amphimedon FRZB. Not shown in this picture is that the proline is also missing in Mnemiopsis FzdA and several other sponge sequences. (PNG) [file pone.0212005.s014.png]
