# Supplementary material for: Secreted frizzled related protein is a target of PaxB and plays a role in aquiferous system development in the freshwater sponge, Ephydatia muelleri
Source: PLoS One. 2019 Feb 22;14(2):e0212005. doi: 10.1371/journal.pone.0212005 (PMC6386478; doi:10.1371/journal.pone.0212005)
Supplement: S1 Table — (PDF) [file pone.0212005.s008.pdf]

PaxB Positive Binding Sites

Site	Sequence
EmSixA	GTGAATTCGTAGGTACT
EmSixG	CAGCAGTGCTCCGAACA
EmSixK	GCGTGGGGAATCGTGCA
AqSix1	AAGCTTTGGACTGGAAA
AqSix2	AAGCCATAATGTGGTCA
AqSix3	GTGCATGCATGTATGCC

PaxB Negative Binding Sites

Site	Sequence
EmSixB	TCGCTGTGATGAATACC
EmSixC	GCGCAAGGAAGCAGGCC
EmSixD	AAGAAGCGATTGGAGAA
EmSixE	GGTCCCTCCTGTGTGA
EmSixF	AAGCTCTGGTGCATGGC
EmSixH	AACAATACAAGCTGGA
EmSixI	ACAGAGTCAGTCGTACC
EmSixJ	TAGCATACAATCGCGCATAAC
