# Supplementary material for: Secreted frizzled related protein is a target of PaxB and plays a role in aquiferous system development in the freshwater sponge, Ephydatia muelleri
Source: PLoS One. 2019 Feb 22;14(2):e0212005. doi: 10.1371/journal.pone.0212005 (PMC6386478; doi:10.1371/journal.pone.0212005)
Supplement: S2 Table — (PDF) [file pone.0212005.s009.pdf]

Amphimedon predicted peptide	Ephydatia scaffold	hit_score	hit_expect	# binding sites
gpp|Aqu1.227323|protein_O-mannosyl-transferase_2-like	scaffold4453|size16237	49.7	6.00E-05	10
gpp|Aqu1.229216|e3ubiquitin-protein_ligase_RNF5-like	scaffold8125|size4137	164	4.06E-44	9
gpp|Aqu1.203043|frizzled-8-like	scaffold6592|size4731	57	9.00E-09	8
gpp|Aqu1.204415|hypothetical_protein_LOC100633818	scaffold8125|size4137	45.4	3.00E-05	8
gpp|Aqu1.207188|RING_finger_and_transmembrane_domain-containing_protein_1-likepartial	scaffold8125|size4137	46.6	1.00E-05	8
gpp|Aqu1.209767|RING_finger_protein_nhl-1-like	scaffold8125|size4137	47	9.00E-05	8
gpp|Aqu1.219980|UDP-N-acetylglucosamine--peptide_N-acetylglucosaminyltransferase_110kDa_subunit-like	scaffold3895|size41610	53.9	9.00E-06	8
gpp|Aqu1.220903|e3ubiquitin-protein_ligase_RFWD2-like	scaffold8125|size4137	50.1	9.00E-06	8
gpp|Aqu1.224594|secreted_frizzled-related_protein_3-like	scaffold6592|size4731	54.3	2.00E-07	8
gpp|Aqu1.225381|DCRD	scaffold6592|size4731	54.7	3.00E-07	8
gpp|Aqu1.228355|frizzled-2	scaffold6592|size4731	53.9	6.00E-07	8
gpp|Aqu1.228356|frizzled-8-like	scaffold6592|size4731	57.8	6.00E-09	8
gpp|Aqu1.228399|e3ubiquitin-protein_ligase_RNF146-like	scaffold8125|size4137	48.5	1.00E-05	8
gpp|Aqu1.229216|e3ubiquitin-protein_ligase_RNF5-like	scaffold8125|size4137	80.1	7.00E-16	8
gpp|Aqu1.229844|hypothetical_protein_LOC100632604	scaffold1901|size31685	65.9	2.00E-09	8
gpp|Aqu1.201420|hypothetical_protein_LOC100635659	scaffold11303|size3085	58.9	2.00E-09	7
gpp|Aqu1.202476|sushivon_Willebrand_factor_type_AEGF_and_pentraxin_domain-containing_protein_1-like	scaffold2727|size46839	50.4	6.00E-05	7
gpp|Aqu1.206824|hypothetical_protein_LOC100635659	scaffold11303|size3085	62	2.00E-10	7

gpp|Aqu1.208112|protein_FAN-likepartial	scaffold4236|size40413	52.8	1.00E-06	7
gpp|Aqu1.214516|hypothetical_protein_LOC100634493	scaffold11303|size3085	53.1	3.00E-07	7
gpp|Aqu1.215420|lipopolysaccharide-responsive_and_beige-like_anchor_protein	scaffold4236|size40413	60.5	9.00E-16	7
gpp|Aqu1.215420|lipopolysaccharide-responsive_and_beige-like_anchor_protein	scaffold4236|size40413	42.4	9.00E-16	7
gpp|Aqu1.215420|lipopolysaccharide-responsive_and_beige-like_anchor_protein	scaffold4236|size40413	23.5	9.00E-16	7
gpp|Aqu1.215805|hypothetical_protein_LOC100635147	scaffold11303|size3085	48.5	2.00E-05	7
gpp|Aqu1.216780|hypothetical_protein_LOC100632884	scaffold11303|size3085	58.5	6.00E-09	7
gpp|Aqu1.217795|sushivon_Willebrand_factor_type_AEGF_and_pentraxin_domain-containing_protein_1-like	scaffold2727|size46839	51.2	7.00E-05	7
gpp|Aqu1.218282|neurobeachin-like_protein_2-like	scaffold4236|size40413	69.3	5.61E-45	7
gpp|Aqu1.218282|neurobeachin-like_protein_2-like	scaffold4236|size40413	68.9	5.61E-45	7
gpp|Aqu1.218282|neurobeachin-like_protein_2-like	scaffold4236|size40413	68.6	2.00E-13	7
gpp|Aqu1.218282|neurobeachin-like_protein_2-like	scaffold4236|size40413	30.4	2.00E-13	7
gpp|Aqu1.218282|neurobeachin-like_protein_2-like	scaffold4236|size40413	87.8	5.61E-45	7
gpp|Aqu1.221629|hypothetical_protein_LOC100634686	scaffold2833|size56664	58.9	4.00E-07	7
gpp|Aqu1.224505|hypothetical_protein_LOC100635147	scaffold11303|size3085	60.1	2.00E-09	7
gpp|Aqu1.225573|lysosomal-trafficking_regulator-like	scaffold4236|size40413	51.6	8.00E-14	7
gpp|Aqu1.225573|lysosomal-trafficking_regulator-like	scaffold4236|size40413	48.9	8.00E-14	7
gpp|Aqu1.226362|mental_retardation_GTPase_activating_protein_homolog_3-like	scaffold11303|size3085	43.5	6.00E-09	7

gpp|Aqu1.226362|mental_retardation_GTPase_activating_protein_homolog_3-like	scaffold11303|size3085	36.6	6.00E-09	7
gpp|Aqu1.226386|WD_repeat_and_FYVE_domain-containing_protein_3-like	scaffold4236|size40413	51.2	2.00E-14	7
gpp|Aqu1.226386|WD_repeat_and_FYVE_domain-containing_protein_3-like	scaffold4236|size40413	50.8	2.00E-14	7
gpp|Aqu1.226386|WD_repeat_and_FYVE_domain-containing_protein_3-like	scaffold4236|size40413	50.8	9.00E-05	7
gpp|Aqu1.226536|TBC1domain_family_member_15-like	scaffold1288|size38923	43.9	2.00E-06	7
gpp|Aqu1.226536|TBC1domain_family_member_15-like	scaffold1288|size38923	55.8	2.00E-06	7
gpp|Aqu1.227427|sushivon_Willebrand_factor_type_AEGF_and_pentraxin_domain-containing_protein_1-like	scaffold2727|size46839	51.2	5.00E-05	7
gpp|Aqu1.227430|sushivon_Willebrand_factor_type_AEGF_and_pentraxin_domain-containing_protein_1-like	scaffold2727|size46839	54.3	7.00E-06	7
gpp|Aqu1.227430|sushivon_Willebrand_factor_type_AEGF_and_pentraxin_domain-containing_protein_1-like	scaffold2727|size46839	51.2	6.00E-05	7
gpp|Aqu1.227430|sushivon_Willebrand_factor_type_AEGF_and_pentraxin_domain-containing_protein_1-like	scaffold2727|size46839	54.7	5.00E-06	7
gpp|Aqu1.227431|sushivon_Willebrand_factor_type_AEGF_and_pentraxin_domain-containing_protein_1-like	scaffold2727|size46839	51.2	6.00E-05	7
gpp|Aqu1.227431|sushivon_Willebrand_factor_type_AEGF_and_pentraxin_domain-containing_protein_1-like	scaffold2727|size46839	54.7	6.00E-06	7
gpp|Aqu1.227431|sushivon_Willebrand_factor_type_AEGF_and_pentraxin_domain-containing_protein_1-like	scaffold2727|size46839	54.3	7.00E-06	7
gpp|Aqu1.227432|sushivon_Willebrand_factor_type_AEGF_and_pentraxin_domain-containing_protein_1-like	scaffold2727|size46839	54.7	6.00E-06	7
gpp|Aqu1.227432|sushivon_Willebrand_factor_type_AEGF_and_pentraxin_domain-containing_protein_1-like	scaffold2727|size46839	54.3	7.00E-06	7
gpp|Aqu1.227432|sushivon_Willebrand_factor_type_AEGF_and_pentraxin_domain-containing_protein_1-like	scaffold2727|size46839	51.2	6.00E-05	7
gpp|Aqu1.228770|glucose-6-phosphate_1-dehydrogenase-like	scaffold327|size63049	103	2.00E-31	7
gpp|Aqu1.228770|glucose-6-phosphate_1-dehydrogenase-like	scaffold327|size63049	43.1	8.00E-13	7
gpp|Aqu1.228770|glucose-6-phosphate_1-dehydrogenase-like	scaffold327|size63049	56.6	2.00E-31	7

gpp|Aqu1.229238|myosin_heavy_chainstriated_muscle-like	scaffold833|size33861	53.5	9.00E-06	7
gpp|Aqu1.229238|myosin_heavy_chainstriated_muscle-like	scaffold833|size33861	56.2	2.00E-06	7
gpp|Aqu1.229238|myosin_heavy_chainstriated_muscle-like	scaffold833|size33861	53.9	7.00E-06	7
gpp|Aqu1.229392|BAI1-associated_protein_3-like	scaffold13833|size12618	50.8	2.00E-05	7
gpp|Aqu1.229461|sushivon_Willebrand_factor_type_AEGF_and_pentraxin_domain-containing_protein_1-like	scaffold2727|size46839	50.8	7.00E-05	7
gpp|Aqu1.229463|CUB_and_sushi_domain-containing_protein_1-like	scaffold2727|size46839	54.3	5.00E-06	7
gpp|Aqu1.229841|hypothetical_protein_LOC100632476	scaffold1901|size31685	84	4.00E-15	7
gpp|Aqu1.229844|hypothetical_protein_LOC100632604	scaffold1901|size31685	69.3	1.00E-10	7
gpp|Aqu1.229845|hypothetical_protein_LOC100632604	scaffold1901|size31685	50.1	9.00E-05	7
gpp|Aqu1.230206|hypothetical_protein_LOC100638242	scaffold11303|size3085	50.4	5.00E-07	7
gpp|Aqu1.230287|hypothetical_protein_LOC100631795	scaffold1901|size31685	67	5.00E-10	7
gpp|Aqu1.200487|ADP-ribosylation_factor_1-like	scaffold295|size22285	101	1.00E-33	6
gpp|Aqu1.200487|ADP-ribosylation_factor_1-like	scaffold295|size22285	34.3	1.00E-33	6
gpp|Aqu1.200717|gamma-aminobutyric_acid_type_B_receptor_subunit_2-like	scaffold6359|size8991	51.6	5.00E-09	6
gpp|Aqu1.201290|gamma-aminobutyric_acid_type_B_receptor_subunit_2-like	scaffold6359|size8991	66.2	5.00E-11	6
gpp|Aqu1.201822|acetyl-coenzyme_A_synthetase_2-likemitochondrial-like	scaffold3302|size56130	64.7	2.00E-37	6
gpp|Aqu1.201968|acetyl-coenzyme_A_synthetase-likepartial	scaffold3302|size56130	53.9	8.00E-42	6
gpp|Aqu1.202837|AP-2complex_subunit_beta-like	scaffold2684|size10648	108	6.00E-25	6

gpp|Aqu1.203327|spectrin_alpha_chainbrain-likepartial	scaffold5157|size30562	79.3	4.06E-44	6
gpp|Aqu1.203636|hypothetical_protein_LOC100641775	scaffold2684|size10648	50.4	3.00E-06	6
gpp|Aqu1.204582|gamma-aminobutyric_acid_type_B_receptor_subunit_2-like	scaffold6359|size8991	64.7	5.00E-11	6
gpp|Aqu1.204589|actin-related_protein_10-like	scaffold2005|size9171	73.6	5.00E-14	6
gpp|Aqu1.205057|hypothetical_protein_LOC100633972	scaffold3085|size20018	59.3	5.00E-08	6
gpp|Aqu1.205336|hypothetical_protein_LOC100641125	scaffold2727|size46839	58.9	5.00E-21	6
gpp|Aqu1.205336|hypothetical_protein_LOC100641125	scaffold2727|size46839	66.2	5.00E-21	6
gpp|Aqu1.207767|spectrin_alpha_chainbrain-like	scaffold5157|size30562	48.9	3.00E-13	6
gpp|Aqu1.207767|spectrin_alpha_chainbrain-like	scaffold5157|size30562	30.4	2.00E-08	6
gpp|Aqu1.207767|spectrin_alpha_chainbrain-like	scaffold5157|size30562	33.9	7.00E-07	6
gpp|Aqu1.209607|spermatogenesis-associated_protein_17-like	scaffold7123|size12914	47	2.00E-05	6
gpp|Aqu1.212330|ADP-ribosylation_factor-like_protein_8A-like	scaffold295|size22285	56.2	7.00E-09	6
gpp|Aqu1.214342|spectrin_alpha_chainbrain-like	scaffold5157|size30562	57.8	1.00E-19	6
gpp|Aqu1.214342|spectrin_alpha_chainbrain-like	scaffold5157|size30562	48.5	3.00E-13	6
gpp|Aqu1.214342|spectrin_alpha_chainbrain-like	scaffold5157|size30562	30.4	2.00E-08	6
gpp|Aqu1.214342|spectrin_alpha_chainbrain-like	scaffold5157|size30562	41.2	1.00E-07	6
gpp|Aqu1.214342|spectrin_alpha_chainbrain-like	scaffold5157|size30562	33.9	4.00E-07	6
gpp|Aqu1.214342|spectrin_alpha_chainbrain-like	scaffold5157|size30562	79.7	4.06E-44	6
gpp|Aqu1.214410|spectrin_beta_chainbrain_1-like	scaffold5157|size30562	52.8	2.00E-19	6

gpp|Aqu1.214410|spectrin_beta_chainbrain_1-like	scaffold5157|size30562	53.1	1.00E-15	6
gpp|Aqu1.214410|spectrin_beta_chainbrain_1-like	scaffold5157|size30562	33.1	1.00E-08	6
gpp|Aqu1.214410|spectrin_beta_chainbrain_1-like	scaffold5157|size30562	29.3	7.00E-05	6
gpp|Aqu1.217299|gamma-aminobutyric_acid_type_B_receptor_subunit_2-like	scaffold6359|size8991	50.8	1.00E-05	6
gpp|Aqu1.217307|gamma-aminobutyric_acid_type_B_receptor_subunit_2-like	scaffold6359|size8991	48.1	9.00E-05	6
gpp|Aqu1.217943|gamma-aminobutyric_acid_type_B_receptor_subunit_2-like	scaffold6359|size8991	62.8	3.00E-11	6
gpp|Aqu1.217944|gamma-aminobutyric_acid_type_B_receptor_subunit_2-like	scaffold6359|size8991	63.5	1.00E-10	6
gpp|Aqu1.217945|gamma-aminobutyric_acid_type_B_receptor_subunit_2-like	scaffold6359|size8991	67.4	2.00E-11	6
gpp|Aqu1.217946|gamma-aminobutyric_acid_type_B_receptor_subunit_2-like	scaffold6359|size8991	64.7	1.00E-13	6
gpp|Aqu1.218262|eukaryotic_peptide_chain_release_factor_GTP-binding_subunit_ERF3A-like	scaffold3161|size58911	50.4	2.00E-07	6
gpp|Aqu1.218262|eukaryotic_peptide_chain_release_factor_GTP-binding_subunit_ERF3A-like	scaffold3161|size58911	72.8	2.00E-11	6
gpp|Aqu1.218262|eukaryotic_peptide_chain_release_factor_GTP-binding_subunit_ERF3A-like	scaffold3161|size58911	28.9	2.00E-07	6
gpp|Aqu1.218761|hypothetical_protein_LOC100639376	scaffold5606|size10925	66.2	4.00E-11	6
gpp|Aqu1.218969|mitochondrial_carnitine-acylcarnitine_carrier_protein_CACL-like	scaffold3085|size20018	63.9	5.00E-11	6
gpp|Aqu1.219666|hypothetical_protein_LOC100641129	scaffold599|size18658	56.2	2.00E-15	6
gpp|Aqu1.219666|hypothetical_protein_LOC100641129	scaffold599|size18658	48.1	2.00E-15	6
gpp|Aqu1.220904|hypothetical_protein_LOC100633808	scaffold612|size28535	59.7	7.00E-08	6
gpp|Aqu1.220905|tyrosine-protein_kinase_CSK-like	scaffold612|size28535	49.7	9.00E-05	6

gpp|Aqu1.221373|tyrosine-protein_kinase_CSK-like	scaffold612|size28535	51.6	2.00E-05	6
gpp|Aqu1.221503|sushivon_Willebrand_factor_type_AEGF_and_pentraxin_domain-containing_protein_1-like	scaffold2727|size46839	57.8	7.00E-07	6
gpp|Aqu1.221527|hypothetical_protein_LOC100641125	scaffold2727|size46839	66.2	2.00E-20	6
gpp|Aqu1.221527|hypothetical_protein_LOC100641125	scaffold2727|size46839	56.6	2.00E-20	6
gpp|Aqu1.221528|hypothetical_protein_LOC100641125	scaffold2727|size46839	67.8	4.00E-22	6
gpp|Aqu1.221528|hypothetical_protein_LOC100641125	scaffold2727|size46839	60.8	4.00E-22	6
gpp|Aqu1.221529|hypothetical_protein_LOC100641125	scaffold2727|size46839	70.9	7.00E-22	6
gpp|Aqu1.221529|hypothetical_protein_LOC100641125	scaffold2727|size46839	57	7.00E-22	6
gpp|Aqu1.222007|gamma-aminobutyric_acid_type_B_receptor_subunit_1-like	scaffold6359|size8991	68.6	6.00E-11	6
gpp|Aqu1.223204|ADP-ribosylation_factor-like_protein_1-like	scaffold295|size22285	79	2.00E-22	6
gpp|Aqu1.223204|ADP-ribosylation_factor-like_protein_1-like	scaffold295|size22285	28.5	2.00E-22	6
gpp|Aqu1.223933|hypothetical_protein_LOC100633972	scaffold3085|size20018	59.3	1.00E-07	6
gpp|Aqu1.223991|ADP-ribosylation_factor-like	scaffold295|size22285	80.5	4.00E-21	6
gpp|Aqu1.223991|ADP-ribosylation_factor-like	scaffold295|size22285	26.2	4.00E-21	6
gpp|Aqu1.224189|kinesin-like_protein_KIF18A-like	scaffold3534|size8813	49.7	3.00E-07	6
gpp|Aqu1.224429|actin-related_protein_10-like	scaffold2005|size9171	74.3	5.00E-14	6
gpp|Aqu1.224835|ADP-ribosylation_factor-like_protein_8A-like	scaffold295|size22285	56.6	7.00E-09	6
gpp|Aqu1.225061|elongation_factor_1-alpha-like	scaffold3161|size58911	69.7	1.00E-10	6
gpp|Aqu1.225061|elongation_factor_1-alpha-like	scaffold3161|size58911	59.7	1.00E-07	6

gpp|Aqu1.225062|elongation_factor_1-alpha-like	scaffold3161|size58911	60.1	2.00E-13	6
gpp|Aqu1.225062|elongation_factor_1-alpha-like	scaffold3161|size58911	39.3	2.00E-13	6
gpp|Aqu1.225062|elongation_factor_1-alpha-like	scaffold3161|size58911	70.1	7.00E-11	6
gpp|Aqu1.225335|thyroid_adenoma-associated_protein_homolog	scaffold2684|size10648	72.8	1.00E-12	6
gpp|Aqu1.225394|putative_pre-mRNA-splicing_factor_ATP-dependent_RNA_helicase_DHX15-like	scaffold6359|size8991	65.1	2.00E-11	6
gpp|Aqu1.225409|ADP-ribosylation_factor-like_protein_3-like	scaffold295|size22285	27.7	2.00E-10	6
gpp|Aqu1.225409|ADP-ribosylation_factor-like_protein_3-like	scaffold295|size22285	60.8	2.00E-10	6
gpp|Aqu1.225827|mitogen-activated_protein_kinase-binding_protein_1-like	scaffold2396|size8385	54.3	1.00E-06	6
gpp|Aqu1.225849|gamma-aminobutyric_acid_type_B_receptor_subunit_2-like	scaffold6359|size8991	63.5	2.00E-09	6
gpp|Aqu1.226105|gamma-aminobutyric_acid_type_B_receptor_subunit_2-like	scaffold6359|size8991	45.4	3.00E-06	6
gpp|Aqu1.226105|gamma-aminobutyric_acid_type_B_receptor_subunit_2-like	scaffold6359|size8991	26.6	3.00E-06	6
gpp|Aqu1.226106|gamma-aminobutyric_acid_type_B_receptor_subunit_2-like	scaffold6359|size8991	45.4	1.00E-07	6
gpp|Aqu1.226362|mental_retardation_GTPase_activating_protein_homolog_3-like	scaffold11303|size3085	57.8	2.00E-08	6
gpp|Aqu1.226448|ADP-ribosylation_factor_6-like	scaffold295|size22285	83.2	2.00E-29	6
gpp|Aqu1.226448|ADP-ribosylation_factor_6-like	scaffold295|size22285	22.3	2.00E-29	6
gpp|Aqu1.226448|ADP-ribosylation_factor_6-like	scaffold295|size22285	38.5	2.00E-29	6
gpp|Aqu1.226536|TBC1domain_family_member_15-like	scaffold1288|size38923	31.6	2.00E-06	6
gpp|Aqu1.227108|2,3-bisphosphoglycerate-independent_phosphoglycerate_mutase-like	scaffold1476|size29941	63.2	7.00E-09	6

gpp|Aqu1.227293|ADP-ribosylation_factor-like_protein_2-like	scaffold295|size22285	72	1.00E-12	6
gpp|Aqu1.227960|hypothetical_protein_LOC100638160	scaffold3161|size58911	48.5	7.00E-19	6
gpp|Aqu1.227960|hypothetical_protein_LOC100638160	scaffold3161|size58911	84.3	3.00E-15	6
gpp|Aqu1.227960|hypothetical_protein_LOC100638160	scaffold3161|size58911	69.7	7.00E-19	6
gpp|Aqu1.228407|ADP-ribosylation_factor-like_protein_5B-like	scaffold295|size22285	58.5	1.00E-11	6
gpp|Aqu1.228407|ADP-ribosylation_factor-like_protein_5B-like	scaffold295|size22285	33.9	1.00E-11	6
gpp|Aqu1.228445|ADP-ribosylation_factor_1-like	scaffold295|size22285	102	2.00E-37	6
gpp|Aqu1.228445|ADP-ribosylation_factor_1-like	scaffold295|size22285	45.4	2.00E-37	6
gpp|Aqu1.228447|ADP-ribosylation_factor-like	scaffold295|size22285	79.7	3.00E-21	6
gpp|Aqu1.228491|ADP-ribosylation_factor_1-like	scaffold295|size22285	102	5.00E-38	6
gpp|Aqu1.228491|ADP-ribosylation_factor_1-like	scaffold295|size22285	46.2	5.00E-38	6
gpp|Aqu1.228491|ADP-ribosylation_factor_1-like	scaffold295|size22285	23.1	5.00E-38	6
gpp|Aqu1.228492|ADP-ribosylation_factor_1-like	scaffold295|size22285	100	4.00E-30	6
gpp|Aqu1.228492|ADP-ribosylation_factor_1-like	scaffold295|size22285	48.9	2.00E-05	6
gpp|Aqu1.228492|ADP-ribosylation_factor_1-like	scaffold295|size22285	23.9	4.00E-30	6
gpp|Aqu1.228770|glucose-6-phosphate_1-dehydrogenase-like	scaffold327|size63049	54.7	8.00E-13	6
gpp|Aqu1.228807|dolichyl-P-Man:Man(7)GlcNAc(2)-PP-dolichyl-alpha-1,6-mannosyltransferase-like	scaffold1837|size9476	59.7	1.00E-11	6
gpp|Aqu1.228807|dolichyl-P-Man:Man(7)GlcNAc(2)-PP-dolichyl-alpha-1,6-mannosyltransferase-like	scaffold1837|size9476	31.6	1.00E-11	6
gpp|Aqu1.228814|ADP-ribosylation_factor_4-like	scaffold295|size22285	93.2	3.00E-34	6

gpp|Aqu1.228814|ADP-ribosylation_factor_4-like	scaffold295|size22285	40.8	3.00E-34	6
gpp|Aqu1.228814|ADP-ribosylation_factor_4-like	scaffold295|size22285	24.3	3.00E-34	6
