# Supplementary material for: Secreted frizzled related protein is a target of PaxB and plays a role in aquiferous system development in the freshwater sponge, Ephydatia muelleri
Source: PLoS One. 2019 Feb 22;14(2):e0212005. doi: 10.1371/journal.pone.0212005 (PMC6386478; doi:10.1371/journal.pone.0212005)
Supplement: S4 File — (RTF) [file pone.0212005.s013.rtf]

>scaffold4453|size16237TGGCTTTAGTATAGCACCAGCGGCGCAATATTGCTCTGTTGCCCGTGAAAGAGTGTACTCTGCTCTTGTTGTGTGAACCTCTTGCGACATAGTGGGTGTCTGTGATGGTGCCTGTGATGGTGTATGTGATGGTGTATGTGATGGTGTATGTGATGGTGCCTGTGATGGTGTATGTGATGGTGTATGTGAGTGCTGTGGGATACGTGAGTACGTGACTTAAGATATCAGGTTTCATTGCACGCTCGCATACATTTGCAAATATCGTTCCTATAGATTGTATTGGTTGTCCTGGCAACATGTCAAAGGTCATGGTTAAGTACACAGCACGCAACCTTTGCTTTAAAGGAGTCATGCTAAGTGTACATCCAGATGCTCTTCCTCATAAGGGCTGATCGCCATGTTGCTAGGAATGTGAATAAAGTGGCATTGGTGTGGAACAGCGGTGTGAATTGTTAGTGTTCTCTGCACTGTGAGGTTTCCACTAGGGCAGTATCCAAATGCAGTTAGAAAAGCTGAGTAGAGGGGGGGAAGGAANCAGCCTTATGCATCTCATCNTNNATTCNANNNGNGNTGCAATAGGTGCAAACACAAGAGGCTAACAATTGTATTGAATTGCATGTACTCACATACTTCCTATAATGAAGCCTTTTCTCTGTGACATCAATCATGAATGCCAAGGTCGTACAGGCTATTTGCATTAATCCCTNNANCNCTCAGGGATGCATTGAACCCCTGGGATGGATTGAACCCCCAGGGATGCATTGAACCCCTGGGATGGATTGAACCCCTNGGGTCACCAGGGATGCATTGNNNNNNNNNNNNNNNNNNNNNNNNNNNNNNNNNNNNNNNNNNNNNNNNNNNNNNNNNNNNNNNNNNNNNNNNNNNNNNNNNNNNNNNNNNNNNNNNNNNNNNNNNNNNNNNNNNNNNNNNNNNNNNNNNNNNNNNNNNNNNNNNNNNNNNNNNNNNNNNNNNNNNNNNNNNNNNNNNNNNNNNNNNNNNNNNNNNNNNNNNNNNNNNNNNNNNNNNNNNNNNNNNNNNNNNNNNNNNNNNNNNNNNNNNNNNNNNNNNNNNNNNNNNNNNNNNNNNNNNNNNNNNNNNNNNNNNNNNNNNNNNNNNNNNNNNNNNNNNNNNNNNNNNNNNNNNNNNNNNNNNNNNNNNNNNNNNNNNNNNNNNNNNNNNNNNNNNNNNNNNNNNNNNNNNNNNNNNNNNNNNNNNNNNNNNNNNNNNNNNNNNNNNNNNNNNNNNNNNNNNNNNNNNNNNNNNNNNNNNNNNNNNNNNNNNNNNNNNNNNNNNNNNNNNNNNNNNNNNNNNNNNNNNNNNNNNNNNNNNNNNNNNNNNNNNNNNNNNNNNNNNNNNNNNNNNNNNNNNNNNNNNNNNNNNNNNNNNNNNNNNNNNNNNNNNNNNNNNNNNNNNNNNNNNNNNNNNNNNNNNNNNNNNNNNNNNNNNNNNNNNNNNNNNNNNNNNNNNNNNNNNNNNNNNNNNNNNNNNNNNNNNNNNNNNNNNNNNNNNNNNNNNNNNNNNNNNNNNNNNNNNNNNNNNNNNNNNNNNNNNNNNNNNNNNNNNNNNNNNNNNNNNNNNNNNNNNNNNNNNNNNNNNNNNNNNNNNNNNNNNNNNNNNNNNNNNNNNNNNNNNNNNNNNNNNNNNNNNNNNNNNNNNNNNNNNNNNNNNNNNNNNNNNNNNNNNNNNNNNNNNNNNNNNNNNNNNNNNNNNNNNNNNNNNNNNNNNNNNNNNNNNNNNNNNNNNNNNNNNNNNNNNNNNNNNNNNNNNNNNNNNNNNNNNNNNNNNNNNNNNNNNNNNNNNNNNNNNNNNNNNNNNNNNNNNNNNNNNNNNNNNNNNNNNNNNNNNNNNNNNNNNNNNNNNNNNNNNNNNNNNNNNNNNNNNNNNNNNNNNNNNNNNNNNNNNNNNNNNNNNNNNNNNNNNNNNNNNNNNNNNNNNNNNNNNNNNNNNNNNNNNNNNNNNNNNNNNNNNNNNNNNNNNNNNNNNNNNNNNNNNNNNNNNNNNNNNNNNNNNNNNNNNNNNNNNNNNNNNNNNNNNNNNNNNNNNNNNNNNNNNNNNNNNNNNNNNNNNNNNNNNNNNNNNNNNNNNNNNNNNNNNNNNNNNNNNNNNNNNNNNNNNNNNNNNNNNNNNNNNNNNNNNNNNNNNNNNNNNNNNNNNNNNNNNNNNNNNNNNNNNNNNNNNNNNNNNNNNNNNNNNNNNNNNNNNNNNNNNNNNNNNNNNNNNNNNNNNNNNNNNNNNNNNNNNNNNNNNNNNNNNNNNNNNNNNNNNNNNNNNNNNNNNNNNNNNNNNNNNNNNNNNNNNNNNNNNNNNNNNNNNNNNNNNNNNNNNNNNNNNNNNNNNNNNNNNNNNNNNNNNNNNNNNNNNNNNNNNNNNNNNNNNNNNNNNNNNNNNNNNNNNNNNNNNNNNNNNNNNNNNNNNNNNNNNNNNNNNNNNNNNNNNNNNNNNNNNNNNNNNNNNNNNNNNNNNNNNNNNNNNNNNNNNNNNNNNNNNNNNNNNNNNNNNNNNNNNNNNNNNNNNNNNNNNNNNNNNNNNNNNNNNNNNNNNNNNNNNNNNNNNNNNNNNNNNNNNNNNNNNNNNNNNNNNNNNNNNNNNNNNNNNNNNNNNNNNNNNNNNNNNNNNNNNNNNNNNNNNNNNNNNNNNNNNNNNNNNNNNNNNNNNNNNNNNNNNNNNNNNNNNNNNNNNNNNNNNNNNNNNNNNNNNNNNNNNNNNNNNNNNNNNNNNNNNNNNNNNNNNNNNNNNNNNNNNNNNNNNNNNNNNNNNNNNNNNNNNNNNNNNNNNNNNNNNNNNNNNNNNNNNNNNNNNNNNNNNNNNNNNNNNNNNNNNNNNNNNNNNNNNNNNNNNNNNNNNNNNNNNNNNNNNNNNNNNNNNNNNNNNNNNNNNNNNNNNNNNNNNNNNNNNNNNNNNNNNNNNNNNNNNNNNNNNNNNNNNNNNNNNNNNNNNNNNNNNNNNNNNNNNNNNNNNNNNNNNNNNNNNNNNNNNNNNNNNNNNNNNNNNNNNNNNNNNNNNNNNNNNNNNNNNNNNNNNNNNNNNNNNNNNNNNNNNNNNNNNNNNNNNNNNNNNNNNNNNNNNNNNNNNNNNNNNNNNNNNNNNNNNNNNNNNNNNNNNNNNNNNNNNNNNNNNNNNNNNNNNNNNNNNNNNNNNNNNNNNNNNNNNNNNNNNNNNNNNNNNNNNNNNNNNNNNNNNNNNNNNNNNNNNNNNNNNNNNNNNNNNNNNNNNNNNNNNNNNNNNNNNNNNNNNNNNNNNNNNNNNNNNNNNNNNNNNNNNNNNNNNNNNNNNNNNNNNNNNNNNNNNNNNNNNNNNNNNNNNNNNNNNNNNNNNNNNNNNNNNNNNNNNNNNNNNNNNNNNNNNNNNNNNNNNNNNNNNNNNNNNNNNNNNNNNNNNNNNNNNNNNNNNNNNNNNNNNNNNNNNNNNNNNNNNNNNNNNNNNNNNNNNNNNNNNNNNNNNNNNNNNNNNNNNNNNNNNNNNNNNNNNNNNNNNNNNNNNNNNNNNNNNNNNNNNNNNNNNNNNNNNNNNNNNNNNNNNNNNNNNNNNNNNNNNNNNNNNNNNNNNNNNNNNNNNNNNNNNNNNNNNNNNNNNNNNNNNNNNNNNNNNNNNNNNNNNNNNNNNNNNNNNNNNNNNNNNNNNNNNNNNNNNNNNNNNNNNNNNNNNNNNNNNNNNNNNNNNNNNNNNNNNNNNNNNNNNNNNNNNNNNNNNNNNNNNNNNNNNNNNNNNNNNNNNNNNNNNNNNNNNNNNNNNNNNNNNNNNNNNNNNNNNNNNNNNNNNNNNNNNNNNNNNNNNNNNNNNNNNNNNNNNNNNNNNNNNNNNNNNNNNNNNNNNNNNNNNNNNNNNNNNNNNNNNNNNNNNNNNNNNNNNNNNNNNNNNNNNNNNNNNNNNNNNNNNNNNGTATGTGTGTGTANNNTGTATNNNNNNNNNNNNNNNNNNNCGNATGTGTGTGAGTGTTATTGTTTACATTTAGTGGTGGCCATCTGATAATGTGCTCCAGGTGGTCAACTACAGTAATGGTGATCATCATCATGCAATGGTACATCAGTGGTCTAATAGTCATCTAATTACATACTGGTAGTGGTCACCTGATGAACATTGGCTGTGACTTTTCAAATGCTTGTTGGCAGTATTATAATTCCATTAATATTTCAGAACAGTTTCTTTTTTAGTGCAAGGCTTTTAGATCAAAGCCTGTGCTGGCCATATTCAGGATGGGTGCGGAAGGTGCAATTTGCATGCCTGCATGGGGTGGAGGGTGGGCTGGATAATATGATACTTCTCTGATCAAGAGCATGGAATGGAATTATGTATGCACAGAACACTTGCACGCTCATATCTGCCTTGTCAATTGTTTCGGATTCTTGGTGTCAAGTATCACAATTAGCCGTTCTGAATATATTAATACAATACATTATCAATAACCCTTACATCCAGCTACATGTGCGGTTTATGGGTTTGCACACTTCACTGGGCATTGCACCATGCACCAGGTAGAACCAATGGCACGATATTGTGCAGCTCTCCACCAGCACATTGCATGACAGTGAAATCACCTCTGTATGTTTTTGTCAGCACCTGTTTGCTGCATATGTAGTACAAGCAAATGCTATATACGGGCATGCATTGAGTTGGGTGTGTCCTTCATCAGGTTGGTACTCTGTTTTTAAGTATCAATCCGTCCAATGGAGCCTCCAGCTGTTAGCCCACACACACTCATTCACTGCTCTACAGGGTTACTATTTGATGTGGTGAGGTCGAGTGTAGGGATGATGTGGTGAGGTCCAGTGTAGGGATGATGTGGTGAGTTCTAATCGAAGAGCATGTGCTTTTGCTATGAAATTGTTTGAGATGACTGTACATGTATGTGTCTTGAGGGAAGTGTGTTGATGAGGTAGGCAGGTGCTGCTCTCATTATGGATGTGTTTTCATTCGACTAGGGAGAGCTATTTATAGCGTGCTATTCAGAAGTACCATATATACAGGGACAAGTTATTTGCTAGAGTAGACACACTTTGTCAACTTGCTGATGTAGGAAATGTGTTTTCAGATGTAGCTTTGCGTAGATACAGTGTGTGAATGATAGGGTGTGCTTCCACATGTACTTTATGAGTTATGTGTGGGCGTATGAATGTGCCTGTGCGCCTGTGTGTGTGTGTTTATCACAGCTTACAATAGTAAGTTCCACGTGGCCCTTTGTAATGAAGTCATCATGAGCATACTGTAAGTGTATGTGTGGTGTTTGTTTGTAGGCTTGTGTCGGTCTGCCTACTAGAGTGGGAGGGGGAGGAGGAAGAGACCACATGTGTATTGTTCTTTCTCTCCTCTTTCAAATAAAGCTCTATTATTTTTCTAACCACAACATTGGCTAGTGGTCTGGCCTACCAAATGGGCACAAAGCATTTGTCTCTCTGTAATGTGTTTCCCCGTCATACGCCATTTAAATACTTGATCATCAACAATTGCCTACAGCAAGCACGTACAAAGAGTCTGATGAGCACTTGGCATTGTGTCGGCGCTTGCTAGCAGAGATATGTGTCGCATTTCGCTTGGCATCGTTGGGCTGCCCATCCTGTCTTCTGTCCAGTGGGAGACTGCCCCCATGGCAACACATTAACACACTGAGTTAGTCTGCACTGCGCTCCAGGAATGGTGTTAGGCCACAGTGCGTGACATTAGGCCACAGTGCACATGACATTGGGCCACAGTGCACATGACATTGGGCCACAGTGCACATGACATTNNNNNACAGTGCACATGACATTAGGCCACAGTGCACATGACATTAGGCCACAGTGCACATGACATTAGGCNACAGTGTATAAGGTTGTGTCAGCAGAGAATTTTACTCTGGTTGCATCCATATATGTTCACACTGTGGAGCCAGCTGACAAGATTACAGATTGATTACATCATTTGTTATTAATGATAGGATATGCAAAATGACTTAGCATGTTTACCACAAGGCTGGTCTTCCTGTTCGCTCCAGGACACACAGAAACCTCCACAGTCACACTCAGAAGGCACCCATCACGGAATCCCACTACCAGGTCTCGTGCTATGGNCNGGTATGGTGGAGCTGTCATTGGGCTGGTATGGTGGAGCTGTCATTGGGCTGGTATGGTGACCCGCATGTTGTTCTCCACCCATGTTGCTTTAATTCAGATATGTCACCAAACTCTGGTTGAGTTATAATGCCTTGTGTACCCCCCTCCCCCACACACACATACACACACACACACATATACACACACACACACACACACATGCACACCCACACACACATGCACACCCACACACCATGCACACACACATCTAGGATGGTGTAGGTGATACCAACGATTATTGGCAGGTCGAAGTGGACGGCGGCAGTCCTGGGGACCAGATCAAGACGGTGACCACAGTTCTCAAGCTGAGGCATGTGAACGTCGGATGCTACCTCCACTCCCACGGCACCCAGCTCCCCAAGTGGTATGTGCCCCTCCACGTAGCCCCACTGCTGGGGGGGTATATGCCCCTCCACGTAGCCCCACTGCTGGGGGGTATGTGCCCCTCCACGTAGCCCCACTGCTGGGGGGGTATATGCCCCTCCATGTAGCCCCACTGCTGGGGGGTATGTGCCCCTCCACGTAGCCCCACTGCTGGGGGNNNNNNNNNNNNNNNNNNNNNNNNNNNNNNNNNNNNNNNNNNNNNNNNNNNNNNNNNNNNNNNNNNNNNNNNNNNNNNNNNNNNNNNNNNNNNNNNNNNNNNNNNNNNNNNNNNNNNNNNNNNNNNNNNNNNNNNNNNNNNNNNNNNNNNNNNNNNNNNNNNNNNNNNNNNNNNNNNNNNNNNNNNNNNNNNNNNNNNNNNNNNNNNNNNNNNNNNNNNNNNNNNNNNNNNNNNNNNNNNNNNNNNNNNNNNNNNNNNNNNNNNNNNNNNNNNNNNNNNNNNNNNNNNNNNNNNNNNNNNNNNNNNNNNNNNNNNNNNNNNNNNNNNNNNNNNNNNNNNNNNNNNNNNNNNNNNNNNNNNNNNNNNNNNNNNNNNNNNNNNNNNNNNNNNNNNNNNNNNNNNNNNNNNNNNNNNNNNNNNNNNNNNNNNNNNNNNNNNNNNNNNNNNNNNNNNNNNNNNNNNNNNNNNNNNNNNNNNNNNNNNNNNNNNNNNNNNNNNNNNNNNNNNNNNNNNNNNNNNNNNNNNNNNNNNNNNNNNNNNNNNNNNNNNNNNNNNNNNNNNNNNNNNNNNNNNNNNNNNNNNNNNNNNNNNNNNNNNNNNNNNNNNNNNNNNNNNNNNNNNNNNNNNNNNNNNNNNNNNNNNNNNNNNNNNNNNNNNNNNNNNNNNNNNNNNNNNNNNNNNNNNNNNNNNNNNNNNNNNNNNNNNNNNNNNNNNNNNNNNNNNNNNNNNNNNNNNNNNNNNNNNNNNNNNNNNNNNNNNNNNNNNNNNNNNNNNNNNNNNNNNNNNNNNNNNNNNNNNNNNNNNNNNNNNNNNNNNNNNNNNNNNNNNNNNNNNNNNNNNNNNNNNNNNNNNNNNNNNNNNNNNNNNNNNNNNNNNNNNNNNNNNNNNNNNNNNNNNNNNNNNNNNNNNNNNNNNNNNNNNNNNNNNNNNNNNNNNNNNNNNNNNNNNNNNNNNNNNNNNNNNNNNNNNNNNNNNNNNNNNNNNNNNNNNNNNNNNNNNNNNNNNNNNNNNNNNNNNNNNNNNNNNNNNNNNNNNNNNNNNNNNNNNNNNNNNNNNNNNNNNNNNNNNNNNNNNNNNNNNNNNNNNNNNNNNNNNNNNNNNNNNNNNNNNNNNNNNNNNNNNNNNNNNNNNNNNNNNNNNNNNNNNNNNNNNNNNNNNNNNNNNNNNNNNNNNNNNNNNNNNNNNNNNNNNNNNNNNNNNNNNNNNNNNNNNNNNNNNNNNNNNNNNNNNNNNNNNNNNNNNNNNNNNNNNNNNNNNNNNNNNNNNNNNNNNNNNNNNNNNNNNNNNNNNNNNNNNNNNNNNNNNNNNNNNNNNNNNNNNNNNNNNNNNNNNNNNNNNNNNNNNNNNNNNNNNNNNNNNNNNNNNNNNNNNNNNNNNNNNNNNNNNNNNNNNNNNNNNNNNNNNNNNNNNNNNNNNNNNNNNNNNNNNNNNNNNNNNNNNNNNNNNNNNNNNNNNNNNNNNNNNNNNNNNNNNNNNNNNNNNNNNNNNNNNNNNNNNNNNNNNNNNNNNNNNNNNNNNNNNNNNNNNNNNNNNNNNNNNNNNNNNNNNNNNNNNNNNNNNNNNNNNNNNNNNNNNNNNNNNNNNNNNNNNNNNNNNNNNNNNNNNNNNNNNNNNNNNNNNNNNNNNNNNNNNNNNNNNNNNNNNNNNNNNNNNNNNNNNNNNNNNNNNNNNNNNNNNNNNNNNNNNNNNNNNNNNNNNNNNNNNNNNNNNNNNNNNNNNNNNNNNNNNNNNNNNNNNNNNNNNNNNNNNNNNNNNNNNNNNNNNNNNNNNNNNNNNNNNNNNNNNNNNNNNNNNNNNNNNNNNNNNNNNNNNNNNNNNNNNNNNNNNNNNNNNNNNNNNNNNNNNNNNNNNNNNNNNNNNNNNNNNNNNNNNNNNNNNNNNNNNNNNNNNNNNNNNNNNNNNNNNNNNNNNNNNNNNNNNNNNNNNNNNNNNNNNNNNNNNNNNNNNNNNNNNNNNNNNNNNNNNNNNNNNNNNNNNNNNNNNNNNNNNNNNNNNNNNNNNNNNNNNNNNNNNNNNNNNNNNNNNNNNNNNNNNNNNNNNNNNNNNNNNNNNNNNNNNNNNNNNNNNNNNNNNNNNNNNNNNNNNNNNNNNNNNNNNNNNNNNNNNNNNNNNNNNNNNNNNNNNNNNNNNNNNNNNNNNNNNNNNNNNNNNNNNNNNNNNNNNNNNNNNNNNNNNNNNNNNNNNNNNNNNNNNNNNNNNNNNNNNNNNNNNNNNNNNNNNNNNNNNNNNNNNNNNNNNNNNNNNNNNNNNNNNNNNNNNNNNNNNNNNNNNNNNNNNNNNNNNNNNNNNNNNNNNNNNNNNNNNNNNNNNNNNNNNNNNNNNNNNNNNNNNNNNNNNNNNNNNNNNNNNNNNNNNNNNNNNNNNNNNNNNNNNNNNNNNNNNNNNNNNNNNNNNNNNNNNNNNNNNNNNNNNNNNNNNNNNNNNNNNNNNNNNNNNNNNNNNNNNNNNNNNNNNNNNNNNNNNNNNNNNNNNNNNNNNNNNNNNNNNNNNNNNNNNNNNNNNNNNNNNNNNNNNNNNNNNNNNNNNNNNNNNNNNNNNNNNNNNNNNNNNNNNNNNNNNNNNNNNNNNNNNNNNNNNNNNNNNNNNNNNNNNNNNNNNNNNNNNNNNNNNNNNNNNNNNNNNNNNNNNNNNNNNNNNNNNNNNNNNNNNNNNNNNNNNNNNNNNNNNNNNNNNNNNNNNNNNNNNNNNNNNNNNNNNNNNNNNNNNNNNNNNNNNNNNNNNNNNNNNNNNNNNNNNNNNNNNNNNNNNNNNNNNNNNNNNNNNNNNNNNNNNNNNNNNNNNNNNNNNNNNNNNNNNNNNNNNNNNNNNNNNNNNNNNNNNNNNNNNNNNNNNNNNNNNNNNNNNNNNNNNNNNNNNNNNNNNNNNNNNNNNNNNNNNNNNNNNNNNNNNNNNNNNNNNNNNNNNNNNNNNNNNNNNNNNNNNNNNNNNNNNNNNNNNNNNNNNNNNNNNNNNNNNNNNNNNNNNNNNNNNNNNNNNNNNNNNNNNNNNNNNNNNNNNNNNNNNNNNNNNNNNNNNNNNNNNNNNNNNNNNNNNNNNNNNNNNNNNNNNNNNNNNNNNNNNNNNNNNNNNNNNNNNNNNNNNNGTTCAGTGAGTGTTCAGCCTTTCTCCATAATACACGTNNANNTATAAGCAAAGGTACTTTGACATTACTGTGAACCATGGACCATTCGCTATCTCAAATGCATGTCTGTTCACTGCTGTTGAGAGGATGGTTTTTGCCACTGTGCTACTGAAGTAAACATGTTAGTGTAGTTTTTATGTTTTGTTGAATGCATTTGCAGATATCTTGCTACACTGTATAGGGTGAGATGTTTGGGATTCAGGAGGCATTATATGGAAATGGAAATGACAAACTTTGGTGGCTTTGTCTTTTTGTGCCTTGAAATCGTTTTTTAATATGCGATGTTGAGGTACATTGGCTAGGGGCAGNGAGTGTTGGGTTTGCCATAGGCCCTTCTGTGGCTGTCTGTTATGCATTGCTAGGCATTGGACACGGCACAATGGCAGAGGTCCAAGTGACAATAATATTTTAAATCAAAGCTGCTTTTTATGAATGCATGCAAGCCAAAAACTAGCTGAATTCCTCGCAGGGAGTGTTGGTTCAGTGAGTAGACACAGCCTTTGCTGACCAGAAGATAGTCTCATCTGGGAGTGTACCAACATTGTGATGGCAGGGCCTGGCTCTATGACAGAACTGTGTGTTGTCATATGTATTTAAAAANTATTTTTTTTTAATGCATATGAGATTGCTCACACACACACAGACACAAACACATACACACATGCCCAAATGCACACACCATGGGCACATGTGCAAACACATACCACACACACACATACATGTAGACACACACATGTGCGCATTGTGCGTACATGTGACTACTCCATGCACGCACACACATGCGCACACTCATGAACACACACACACACACATACACATACACACACACATGCACACACAATTTATTTCTGGTCTTCCTTNGTCCTGNCACTCCTGTCTTCTTGTTGGGTAACTTGTCAGGCATCATTTCACTGTTCNAGGCTGCTGTACGTATATGTAAAGAGGCAGTAGAGTGGGTGTGGTCCTCCCTGATGAGTACACACTGTACTGGTGTCATGTAGTCGTGGAGAGATATGTGGGACAGTGAGCTGGCTGTCAGGGAGTGGAGTTGGGTGTGTGAGATGAATACTAGCAGAGAGATTTGTATACTTACAAATATGTTATTTCAATCTATAGGTGTGGCTGTTTTGTTTTCATTTTGTGAAACATACATTAGATGTGGATGCAAGCATTTCTAATATAAACACGTGATTCACTTAATTTTTTCAGAGACAAATCTAGTCCTTTTTCCCTCCCTACCTACTCAAGCTCTTCTTGGTACATGTAATGGAGTACAAATGGATAGGTATTTTGAAAGCCTTTATGTCGAGGGAAAGGTGGTTCATAATTAATGTGCTACCTGATGGAATGTCTGATTTTTGATGAACCAGTCATTTGGAAATTCATTTTGGCACAGTGCAGGTGTCTCGATGCAGGCACTGAGGGGGTGGGATATGGCCATCCCTTCACCAATAATTGAGGCCACAGTAACCGGGAGCAGTAAGAAAGAGGAATGCAAGAGAGCACTCTATTGGCCATACGAGTTACTGAATAAAATAGGACCCAGGTAGCTTAAAACTACAGACTAACCTGCCTAAAGTTGTCACCCAAGGGTCAGAATTGTTTGCCCATATTATGGAGGTGACTGGCTATAGATGGTTGTGTAAATGCATTCATAGTGGGGTTGCATAAATCAGCATCTTCATCCAGAACTGGCCACAATGGGAGAGTGAACGTCATATCCAGTTGACCGTAAAGCAACTGTACTGTGTGTTAGACTGCACAGTATCTCTATTCAAAAATAAAGCATGAATTACAGCAATCGTTGGTATAAGTATTTGAATGATCAAGTTGCATACATGGGTACATTTTCTCTGAGAGGAGGGAAGGTGGAGATATGTTAGAGTAGATAAGTGATGCCATATATTCACGATGCCTTCTCATTTAATGAAAGCCCACACTTTGTTAATTGATTCCCAATTTATCAAACCATATGCATTGTGGTTTGTGCAATTTCTGAGCAGTCTGCAAACTAGGCTATGCAGGCATTATTAAAAGCCGGACATTGATCAAGGCAAATCATTTCACTTCCAGTGTGTTTAATGTTTAATGTTTAATGTCTATGTGCTGCAATCTGGTGTGTGTGTGTGTGTGTGTGTGTGTGTGTGTGTNNNNNNNNNNNNNNNNNNNNNNNNNNNNNNNNNNNNNNNNNNNNNNNNNNNNNNNNNNNNNNNNNNNNNNNNNNNNNNNNNNNNNNNNNNNNNNNNNNNNNNNNNNNNNNNNNNNNNNNNNNNNNNNNNNNNNNNNNNNNNNNNNNNNNNNNNNNNNNNNNNNNNNNNNNNNNNNNNNNNNNNNNNNNNNNNNNNNNNNNNNNNNNNNNNNNNNNNNNNNNNNNNNNNNNNNNNNNNNNNNNNNNNNNNNNNNNNNNNNNNNNNNNNNNNNNNNNNNNNNNNNNNNNNNNNNNNNNNNNNNNNNNNNNNNNNNNNNNNNNNNNNNNNNNNNNNNNNNNNNNNNNNNNNNNNNNNNNNNNNNNNNNNNNNNNNNNNNNNNNNNNNNNNNNNNNNNNNNNNNNNNNNNNNNNNNNNNNNNNNNNNNNNNNNNNNNNNNNNNNNNNNNNNNNNNNNNNNNNNNNNNNNNNNNNNNNNNNNNNNNNNNNNNNNNNNNNNNNNNNNNNNNNNNNNNNNNNNNNNNNNNNNNNNNNNNNNNNNNNNNNNNNNNNNNNNNNNNNNNNNNNNNNNNNNNNNNNNNNNNNNNNNNNNNNNNNNNNNNNNNNNNNNNNNNNNNNNNNNNNNNNNNNNNNNNNNNNNNNNNNNNNNNNNNNNNNNNNNNNNNNNNNNNNNNNNNNNNNNNNNNNNNNNNNNNNNNNNNNNNNNNNNNNNNNNNNNNNNNNNNNNNNNNNNNNNNNNNNNNNNNNNNNNNNNNNNNNNNNNNNNNNNNNNNNNNNNNNNNNNNNNNNNNNNNNNNNNNNNNNNNNNNNNNNNNNNTGTACCCATTTTGTTACTGTGCACCAGAACTGAAATCCAATAGCACATGTATCTTTGTTAGCTCTGGGCTGAAATAGGTCAGGTCTAGTTCATTCTACACACTTCTAACCAGTTTGCCAAAATAATTTCTGTAGACACCCGGGTGTACAAATGTACCAGGAGACATTAAATGCTGTTTATTGTGGTGTTTCCATTGGTCACCACTGGTCTGCACAAGGCTGGTCTGCCCATGTACATGTATATCAAACACAGTATTCATGACATAAATGAATCTCACACACATGTACTTACACATATTATGCATTATTTTCACTGTCCTTACCAGTACCTTCTAGACCACACCTCCTCATTCCCCTCCCCCTTGCATCCTGAAGCCACACCCCTCACCTGTTGTATTTCTTGTTGCTGCCCTGCCCACCCTAGGTGACAGCCTACACACACAAGGATGACAATAACAAGTGGCTGGTCAAGAGAGCCAAGTGTGAGTCCAGCACTGCACCCCACATGTGCGTCTGTGTACACCATGTGCGTACACCACTAGTGTACACGTGCGTACACCACTAGTGTACACGTGCGTACACGTGCGTACACCACTAGTGTACACGTGCGTACACCANTAGTGTACACGTGCGTACACCACTAGTGTACACGTGCGTACACNNNNNNNNNNNNNNNNNNNNNNNNNNNNNNGTACACGTGCGTACACCACTAGTGTACACGTGCGTACACCACTAGTGTGCATGTGCTTATGCCTCAAGTGTAAAACGTATGTGTACCTGTTTGTGTAGACTGGTGCACGCAGCACAAGTATTGAGGCACAGTGTGGACCAGTTAAGTATGTACTGTACTATGTTTTCACCAAGTGTACGGACATGTGCATGTGTGGGTGGTAGCGCTTGATTGCACTGTTAGAAGTGTACTAGTACCTGTGTATGGATGTTCCTGTATGCACAATGGTTGTGTAATGAAGCAGCTTACAAAGTGTCTCCTCTTTTGGTGATGACTTTCAGCTTCCAACAGTACTGAGGGAGAGGTGGAATTTATCAAGCATGGTGACTGGGTAGTACTGGAACACATCACGTGAGTTGTGTTGTGTGTTGTGTGTGTNTGTGTGTTGTGTGTTGTGTNTGTTGTGTGTGTTTGCATGCATGTGTGTGTGTGCATGTGnTGTGTGATGTGTGCCTGTGTGTTGTGTGTTAGGCTGTGGTGGTGTGTTGTGCAGGATGTTTACAAATGAGCTCAACACTTGTAAAGAATTATAACATCACATTAAAAAAAATTGGTTAATTGTATATTTGTAGTACGCGTATCTGAATTGTACCTATTTAGGCAAATCATATTGCGGAAGGGAAATTCAAGTTATGCTTAAGATGAANAAAATTTAGCATGAATTAATAAAACGATTATAATCCACTAGAAACGATATGCATNATTAAATTACTACAGTTTTTAAACCTGATATTTGAACCAGCTGTTGAGGTTAGCTAATAAAAATTGACCAGGATTTTCTTAACTTATTAAAGACTTATAAACTTTGTTTACGCTCATTTGTAAACATCTTGTGTAATGAGAGGGGTGAGTACTGTACTTGTGTGCCATTGGTGACCTGGATCTTAGAGTTGTGTGTGGGATGCATTTGTACATTTGCATGCATTGTATATGACTCTTTGGTGTGCATGTGCTGCATTGAACGGTGTGCATTGAATGGTGTCTCTTGTGCTGCATTGAATGGTGTCTCTTGTGCTGCATTGAATGGTGTCTCTTGTGCTGCATTGAACGGTGTCTCTTGTGCTGCATTGAACGGTGTGCATTGAACGATGTCTCTTGTGCCTCTGCAGATGTGTATCACACAGCACCTCTTAGCCCGGGTTCTGTGTCTCATCATCACCCCTGTGGTTGCTCTNTGGCTCCATCTTCGTGTTCCACTTCGTGGTGCTCAGTCACAGGTGAGGGTGATGGCAGGGGTGGAGTTGAAGGTGTGGAGTTGGGAGGGACAAGGGCTGGAATCACAGTGTTGCTGTAATTGGAGATGTATTGTCACTGTGTTGATTGAGATGGTCAGGGGTTACCTTCAGAGCTGCGAGGCAAGAGGTGCCTACCAGTGGGGTATCTGGTAGCGCAAATCAAATTAGCTTGCTNGAGGGACTCTGGCCAAGGCAGTAGAATGGTGCACCTTTGCATTGTCCATGTCACCTGACTGTCCATTGCAGTGGAAATGGAGATGGATTTTTCAGTTCTGCGTTTCAGTCTACCTTGGAAGGAAACGAGTTGCATGACCTGCAGGTGCCAGAATGTAAGTCTCTTAGGTGGTGAGGGGTGGGNGNGNGATGTGTNTNTGGGGAGGTGGGGAGATGTGTCTATGGGGAGGTGGAGATGTGTCTATGGGGAGGTGGGGAGATGTGTCTATGGGGAGGTGGGGAGATGTGTCTATGGGGAGGTGGGGAGATGTGTCTATGAGGAGGTGGGGGAGATGTGTCTATGGGGAGGTGAGGGAGATGTGTCTATGGGGAGGTGAGGGAGATGTGTCTATGNGGAGGTGGGGAGATGTGTCNTGGGGAGNNGAGGGAGATGTGTCTATGACACTGTACGTATGCCTGCTTGCAACTACTGATGTACAAGTGTTGCAGTGTTGTAGTGTTGCATGAGCAAATTGAATCACTCATACATGTACTTTGAAGGAAATGTGTTACAAAAATTATCTTCATTCATGCACTTCCAGTTTTAAATTTACCTATTTTGTGCTAATAACGTTAGCAGGACATTGGCTTGTGGTTGAATTCTGTGATGTGGGATATCTACAGTTTCCTGTTCATTTTACAGGACCATATAATGTACATAGGGGTGAATAGTACTGTTTCGCTGGAGTCTGGATTGATTTTAACAATNGTAGTTCACCAATGCCACTGAGTGGACATCTTAATNAGTGGGCATGTTAGTTAACTATCTACCCGTGCANGGACATCTGCTGTTCCCTATGAGCAAAGGTGAAAGTTGCGTGTNTTGTGTAGGTGCACAGCTCCCTCTACAATTGGATGCAGCACCATGCGGTGTCCTGTTGTGCAGTCTGACTATGTTGCCTTGTGTTTAGTGGGTAACTAGTCTTACTCTCATCTCAGATGTGGCCTATGGTTCAACCATCACTCTCAAGAACCATCGTGGAGGAGGGGGGCTTCTCCACTCCCACCATCACCTGTACCCCGAGGGCATGGGGAAATATCAGCAGCAGCAGGTTGGCCTCTGTGTGTGCGTGTATGTGTGTGTGTGTGTGTGTGTGTGTTGTGTGTGTNNNGTGTGTGTGCATGTGTGTGTGTGTGTTGTGTGTGTGTGTGTGTGATGTGCATGTACAAGCTGATTGTAGGCCTTTGAATAGAAGCTGCACTGTGGTGCATGTGTGTTTCACATGGCAATAGTGGATTATTGTTTTGTCGTTTTGTACTCTTCTATTGTACATGTACTGTATGCATGGGTTTGTACAATATGCACACTTCATATGCAGTAAGCATATTTAACACAGTAATTCGGCACA>scaffold8125|size4137CCACTACGACGATCGAGAAAAGCGTTACTGTTGCAACAAAGCATCGCATACATGCTGCAACACTTCTCTCTAATGCCATTGTTTGACNAGAATATGTTTTACTACATTCGAAGTGAGCGGTACTGTATATATCAATACATTACGTAATCGTCTGCATTGTGGTCACTACGTGTCGGGTATGGTGGACATCCCAATAACCACAACAGGCGGGCAGTGTACCTCAAACCCACAATGCAACCGCAGCTAGTGCAAGTGGCTTGGAATGGCAACAGAAATATTAAAACAGCCAGAGCGCCGCACATGTCGTCCAAAATAAACAAAAATACGCAGCCCACATACATATGCACACTGTCATACAATATTCAATTTTTAAANATTTGTTAGATTAAGCAAAGTGCATTGGAACAACGTTAGCTTCATCCAGAACTGAGAAGACCATAAACACAACGTAGAGATAGATGAGGGACACACCATACCACCTGAAGGTCAGGAAGAAAAGAAACATCACAATGTTAGTTGCAACTGAGATTGCAGTAAAACCTTCTCTGCTTTTCAGTTTACTAGAAAATAGCAGAAATCAGCTATTGTGGCAACCTGAACATTCAGATCCTGCCCACAACAAAAAAAATGGTCCTCACAGCATGAATGTATAGACATACCAATGAACAGCTGGGTGAGATAATAGACAATCCACTATTTATTATATTTATTGAGGCTGGCTGGCTGGCTCCACTAGGTGATAAACATTGTGAGGTTCCTTTTATGACAATCTACCACCACAGTGATAAATCACAACCANNTGTGATTTCTGCAANACAGGAAAGCACAACTGCTTGTATAAAGTCGCATGGGGTGTAGTTCATTTGTCCCAGCAATCTGGTGAATGATATATGTGCATGCCATAGCCATTGTTGAGGAAGCTCAATGAAGACAAGTCTCACCAAGCCATGGCTTTTGTGCACTGAATGTCATCTTGCGTGTGCATGTGTTCATGACGTACCTTGGAGGGTTAAACCTGGACACCACAAATACAATGAGTGTGGAGACCAATGAAATTGCCAGAAAGATCCACGATAACTTTACTTCATTAAAAGATGATTGATGAATGTCGAACAAAAATGTGTGACCACTTTTTGTAGCAAATGTGACCTTTGGAGAGAGAAAACAATTATATGACACAATTATATGACACAGACTGAACTGCTTCAGCCATCAGTGTTTACACTACTATGCTCTGCATGTCCTATTTGTATTGTAACTACTACCTTTATAATATTGCCAAACTAATTCTGACCTGGCTGGTCACTATATGCAGGTTAGGCTGTACATATATTATGGTATAGATCAAGGAAAATGTCCTACAAATCCTAAACAGTCACAGACAGCTAGAGCCTGGCAGAAGACTCCCTGATACAGTGGCAACAGCTGCAGCACACAACAGCTGGAATGGGCTAGGAAAGAATGCTAATCGAATTACTTTACCACCACACAATGCTGCTGAAAGATTTCTTAAAGCCTACATATGACTGTATCAGTTCTCAGTACACATACATATTTATCAGTGGCCAGTGATTGATGGTCAATGAAAAATGGTGGTGATGATGTGCATCTGGTAGTGTTTAGGCGGTATGCTTTGGTGGTGAGAGCCCATCCTACATAGAGCCCATCCTATCACTAATATCAACTATCAAAGCAGTTTATAGCTCAAATAGTAGTCATCAACTCCCATTTCTTGACCTTACCTCAGCACGCATATGGACACACCTAGGCACACTCCTAGGCATGCATATGGACACNCCTAGGCANGCATAGATGGACACACCTAGGCACACCCCTAGGCATGCATATGGACACTCCTAGGCACACTCCTAGGCATGCATATGGACACGCCTAGGCACACTCCTAGGCATGCATATGGACACTCCTAGGCATGCATATGGACACTCCTAGGCATGCATATGGGCATGCATATGGACACTCCTAGGCATGCAnCAGCCATATCCAGTGCACGTACAAGGGCATTAATTGGAGTCCGCGCACAAGTTGCGCTTAACAATAACAGCAATATGTACAAATTTGTTGCATTACCGGCTGGAGCCCCTGAGCTGGTACGTAGCAGCCAGAAGGACCGCTTCTATGCACATTATCTGAATGGTCTGCTCTCAGACACTGCTCGACAGCTCTTGCCTCTCCGCCACTGGATCCAGTATCAGAGAGAATTCCAGCTACTAGCTGAGCTAGCATACTACGGGCTAACTACTGTGCATGGAAACCAAACCCTTGGCGANGAATACTGCAACATTGTACAAGTAGCATCCAGTGCGGCTCCAACTATCGCTAGAAGAACGGGCGCGGTTCTGGTTCAGGCATTAGCACCCTATGTATTCGAGAAGCTATTGGAACATATCCACTGGAAACTGACCAATCGNGCATTCTCTCAACGTTTGAGCGACGAGGCGTATGAACGCTTAACGAAAGTAGTTGAATTCTGTGAGGAGACAGTCTCAACACTAAGGAAGCTGCATTTGGCGATATTCTACTTACAAGGCACATTTCACCAGTTTGGTAAGCGGTTGACGAGCATTCGATACATTTTGTTGCGATACAGTGGAGATGGCACAGAACCTCTCATCGATGAATGTATACAAAACATTAGGATGGTTAGTACTACTCCAAATCGTTGTGGGACTGGGCAAGCAGTTATGGAAGAAAGGATTCCAACGTTCACAGACCCGTGGGCATATTCAGGATGATTGTAGATTGCTGTTTGCTCGCAGAGATCGTACACACACTGACATTAAATGTCCCCTTTGTTTAGAACACTGCACTTCTCCTACAACCACATTGTGTGGTCACATATTCTGTTGGCAGTGTGTGGCAGAATGGACCTCTGAAAAGATGGAGTGTCCGTTGTGTCGAAGGGCAACACTGCCCCAAGAACTGGTTTCATTGCAGCATTTTGCAAATCAGTAATGTAATAGCACAAACCAATTTCATTTTGTGGTATGCTGTCATTAAATTTGCAATGAAGTCCAATTGAAAACTATCATCAGTGGTTAGAAAGCATATGTTGTGTACTGCAACAAAGCCATGTGGGATTTTAGTACACTGTTATTTTAATTTTAAGGGGACTTGCTAAAAATTACTTCCCATGTAACTGTTAACGTGTATAAATATTGACACCTATATGAACGTTAACTAAATGCAGCAAAAAGCTATTTTTAATGGCAGTGCTATGTTTCGTTTTAACACATGCGCATCTAGCTTAGTAATCTGTCATCCTGGCTTAGTAATCTGTCATCCTGGCTTAAGATTCACACCTAAGCTATTAGTTGAGGTGATGGCATATGGGGTCAGAGGATATTTCAGAGAGGTGTGTATGCACTTATATTTTTAATGCACTTATATTTTAATGCACTTATATTTTTAATGCACTTATATTTTAATGCACATTTATTTGCACAACAATCACATATAGTGTAATTTACGATTCATCAGTATGTGGAATTATTCAAGAGTTGCTGGGAGATTCTTTATGAGCAACACTTGTCAGTTTCAAGCAAATCTTGGAGATAACTGATAGCATAGTCCACCTCATTCTCGGCACAGCACAGCACAAGGCCTGGTCGTGGACCTCCATGGAGCCTTGCTGATATCAGATACACATCACCAAGGTTCAAGGTGTAGGCTGTGTGCACTTCGACTAGACCCTCAGATGGCAGCTTTCCCTCTACATCCACAAGGTAGTGTGATGCACTGTGTGGTGTGTGAGGCAGGCATTGATCACCAGGGAGCTTCAAAGACTTGGGAATATACATTCCATCGTTTTTTATCACTCTAATAATCCGCATGGAATGAGTGAATTGTCCGTCCTCCGAAGTAGACAGAGCTACCTCGCCTGTGAACACTGAAAGCAAACGCGATGATACGTACAAAAACAAAACAGCATTGGAATCCGCCCCTTTGTGGTATCTTAAGGTCNGCATACACGGGGCGACTTCCTGCCGACTTGTCGCATGCAANNNNNNNNNNNNNNNNNNNNNNNNNNNNNNNNNNNNTGTTTAGTTTACTGATGGTTTGCGGAACATTTGTCCATATTAAAGGCCGGCATACACAGGGCGACTTCCTGCCGATTTG>scaffold6592|size4731ATGCTAATACAAATCTAATCAAGCCCGCATATTATAATCAGACCGATTTTTAGTGCGTAGTAAACCTGTTTACAATTCAATTAAAAATAAAAGCAAGATGACCAGTCTTAGCTATGTATGGGCCATTTCTGGATATGAACAACTGTTTTCCAGAACCTGTGGCATATCAAGGGACCAACTACAACAACAACAAAATAACTACTCGAAGATGATATATTTATTGATATTTATCATCACAGGACCATTCTATAAGTCGGCAAAACGACTTACAGAACGGTTCTGTGATGAAAAATATGAATAAAAGTATCTCGAGTACAACAGACAGTTCTGTCTGTTGTACTCGAGGTGCCNTAGGTTNTTTCTTGATAGCACAACTATCTCACAACTAACGGGTATCGTATTAAATGCCGCATTACCATCCAATAGTTGGAGGCCTTAAAACTGNAACCATCGATTAGTATACAANTCATTTGTAATAAATAAACTTACGTTACTAGCAACCTAAAAATTCATACCATGTAGTAGAAGAAACAGTTCCACAAGGGATATCGGTCACGACTCGTCCCTTTTTTTTTTTCAGACGCAATAAACGACGTGCGCATGAACTCCTGACAGCAAACGTTTTAAGTGATTATAAGGCATATATTCATAATTCAGCACTAACTCACCAAGGAACAATCACAGCACTAGCTACTATAGTGTAAAGCTTCCCCCGACACTTTTTACGAAGCGTGATTCTACATTGCGGATCGTCAGCGTGGAATGCTACGGCTCACGTGATTTGTTTCCAACCCTACAATAAAGGTATGGGGGGAGGCAAAAATGTTAAACGGTGTGTCTTGATCACTGCAAGAATTTCAAAGGAGACTCATTTTCCTACGACTGACTGGCGTTTGATTTGTAGCGTAGGGCACATGTACACTGGATAGACATCAAATTTGGATTTCGTAGAGTTATAGATCCTTTTCATGTGTCATGTGACCGCTCTGTAATCTAACATACGTCAGTGTCACACAATATAAATTAATACACGTCCGCTATGTTGGTGCANANCNTTTNCGGANNTNTCCGAGTGACGAAAAGTACTATCGATATGCTTGCGTCACTTACTATTTGATTTCTTAGAAAGCATTTAGTTCGCACCTCTCTGTAAAGGAACAATCAAAATTGTACACGTCACGAGTCGTGTTGAGTTTAGGGCCTCTGTTTGAGGCATGGACCTCTTCTGGCGTTGATGGTATGTCTGTAATTAAATTTAATATGTAAGAGAATGCAAAGATATGAAACATTCCTCGGAGCGCGCGTGACACGACGTGATCGCTGCAGTGTGAGTCGGTAAACAGAAAAATGCTGTGCCAACGTCACAAATTCCACTGGCACTCCCTCTGATTAGATAAAGTCTAAAATTCACGCCGTCAATAGAACTATTACAATTGGAGCAAGAATCCTAATTGCACCATGAATGGGATCTAGATCGGACTCGTCACACGTCAACCTAATAGACAGCCAAGAGCCTCTCGAATAGCATTTCAATGCCGACTGTTTCAACTCTGCCAGTCTGCCAGCTTTNNNNNTAGTAGGTNCNNTNCNNNTANNNAATGTATGCGTACNGCAGCCATGTACTATGTCTGAATTAATACTCTAAAATCTATCGACCACAAAAAATGATATCGCTGATACACCCGTCCTCACTAAACGTTCTACTACAGGTTATCCATACTGCAGATAGACATAAGGAGCAGCTTTGCATTGGCAAGCTAGGGCGTGCAGTGTTATGTAACATTTCATAACTTACAGTGAACAAAAACACACGCATCCTTACAGAGCTATTTACTTGCATATGAGTGATTCGCACTTCAAAATGTATATTGTAGCAATTTGGCTGTTGAGTCAACATTTAATGAGACCCTTCGATGCCACTGAAAGGCAGCTGTTATAGCATTAATCGTTTCCTCGGGAGGGTAAAAACAGTTTCGATATCCTTTTTGAAAGGGGACTATTTGCACACAGCACACGTAAACAAAGATCGTATAGGCAAAGGAGCCGTAACAAAGTCGAACTTCTATATATTACATCATATAGGGGGACGTATGTACGTATACATTAGGTGAATACCGCATGAAAACTGTCTATACACGTTCCATCCACATGCATGCAGCAAATGGAATTGGTGTTACTAAACACAAGAAATATCTATCATTCCGCGGCGCCATGGCAACTATAGGGTTTGTGTGCGCGTATAAATTGAGGCGCGAAGTCAGACTGACCACAAGTAGACGCAGCAAATCAAAAATTTAGGATAACAGCTCTTGCGTTGCATTACGTCAAAATGGGACGTCTATCAGTCATAGCATTTCTGGCAGCCGCAGCTAGAGTGATTTTGTCCGCACCAACACCTCCCCAGATACTGGTCGTGGAACCATCCAGTGGCCCGGTGTGCGTTCGCTTCAACAGTCCCGTGTGCAANGCTCTAGGGGGACAAGGGTACAATTACGCGACGTTCCCGAACCCCATAGCTCCGGCCTATCTCCCCAATTCCCAGAAAGCCGAGAGCGAAGCCAATGCGGTGATCCAGCAGGCCCTGGCCAGCCGATGTTCTAAGTACGTTGTCGAATTTATGTGCTTCGCCTACTTCCCGCTGTGCGATCCGAACAACACGAGGGCACCCCCCGTGATGCCGTGCAGGTCACTATGCGAGCGGGTTCGGAGCGATTGCGAACCTGTGCTCAATGCCACGTACGGAACGCGTTGGCCCCAATGGGCGAACTGCGAAAANANNGACAAANGNCGTAACCCAGAACCACGCCGATGACCTGTGCTTTGGAAAGGCTGTGGCCGACACTATTCTTACGGAAGCGACGACGACGCAGCCTGCTACCACTGCCACGACAACGCAGCCTGCTACCACTGCCACGACGGCGGCACATCAGAGTGCGCCACAGCGTCCACTGTGCAGCGCCTGCAATCCCGTCCAGGTCACCCCCGAAGCGTTCAAGCTTAGAAATTACACATTTGGTAAGAAGAACATTGCATTTTGCACTGTGCAAGCGTTCACACTTTACTGACTCTCTTTTTCGTGCCACTACAGCTGCAAAGGTGCAAATCAAGTCCAAGTCAACAGCTGATAAGGTCATCACATACGGGGCGGAAGTCTTGCAATACTCACCGCTGCAACAGCAGTGCAGTTCGAACTCGTCTGTTCGATTACCTCCCAAAACGGTGATCATTTCGATGACGGCTAATGGCCAATGTGCGCCTTGCTTTGTCTTGAAACCTGGACAGATCTATCTGGTTGGCGGGAGCACTAGTCAGCAAGACAAAAGTGGCGTATGGTTGGTGCCAAANNNNGCAGNCNTNNNNNNNCCTTGGANNAATCAATACACCCAAAAATGGGTGCAAATGACTGCGAAGAGTGACAAGTGCCAGCTCGACCCCGTTCACGTCTGAGACTCGCTTCGCACACGTCAGTCTATACCCGTTCCAAAAACTATCACAGGTCACTTCATATGCGTTGTGTGAATGGGTGAGAGCGGGAAGATTTTGAAAACCGGCGTTGTACACAACCACATGCACTTTGCACTTTTCCCCTCCACTTTCTTGTTTTGTTGTTGCATGACTTGCAATTTTATGAGATTTATTCAGATTATGTCTTTGTAATTATGTTTATTATGACGTGGAAAGCAATACAGGGTAATTATATCGTGAGCATGATGCTTCAAATAAGCTTGTTCGTGCAAGACTATGTGTAGTACTTGTACGCAGTAATTTAAGCTGCATGGTATATATTGACGTGTCAGAAATGACTGCAAGTATGGCGTTTACTTATGCATGACTAAGGTATCACATGCCAGGGCACTTGAACGTGCCATTTTTAACACGCCAGGTGACTCGACGTGCTTACATTAGCACGAGACATGCGGCTAGGGTCGATCAGCGCTCTCTGATGATCTCATCTTCAGTCTCGTCTTCCGGATTCACGTGCGAGATTTATCACCACACTTGGTGGCAATCGACATATGAGATCGTTTCTGCGAAAAGGGACCTAGTGCACTTGTTTTTTTTTTAGCTATATTCACATAAATAAAAATCTAAAGGATACAAAACAAGAGTCTATTGTGATACAATATAAATTAACACGCAATAGAAATGTAACGCTTGCGTTGAAATATATGGCTAAAAAATAATGCTAATAAAATTTAGTGCCTTACAACTCCATGACAGATAGTCCGACGTAAGAAGAGCTGTGTAGAGGACTTCAGAATATANAAATTGATATATGGGATCGTTTCCGCGAAAAGGGACCTAACGCATGTTAACGCAAGATTTCAAAATTTTTCGTATTCTAGAAGTCTGAAATGTCCTCTACACATTGCTTCTTACACACTGAAAATCGGAGTTTCTATCATGAAGTTGTAAGGCGCGGAATTTTTCATTAGTCGTACATTTCAGTTACGTTTCTATTTGCGCGTACTGGAACTTTATAATGNACGCGTTACGTCCCTTTTCGCGGAAACGATTCCCAATTGAAATCTTGAAAAAACATATTCTANGCCCATTTTCGGGGAAACGATCCTATATGTATTATTCCAAGGATTACAAACTATCCCTCAAACGCATGTACGAATGTCTGCATGTACGCATATCTGCATGTCTGCATGTTTGCATGTCTGCATGTCTGCATGTCTGCATGTTTGCATGTTAAAATGGCGATTACAGTGTGTGTTAAAGCTG>scaffold8125|size4137CCACTACGACGATCGAGAAAAGCGTTACTGTTGCAACAAAGCATCGCATACATGCTGCAACACTTCTCTCTAATGCCATTGTTTGACNAGAATATGTTTTACTACATTCGAAGTGAGCGGTACTGTATATATCAATACATTACGTAATCGTCTGCATTGTGGTCACTACGTGTCGGGTATGGTGGACATCCCAATAACCACAACAGGCGGGCAGTGTACCTCAAACCCACAATGCAACCGCAGCTAGTGCAAGTGGCTTGGAATGGCAACAGAAATATTAAAACAGCCAGAGCGCCGCACATGTCGTCCAAAATAAACAAAAATACGCAGCCCACATACATATGCACACTGTCATACAATATTCAATTTTTAAANATTTGTTAGATTAAGCAAAGTGCATTGGAACAACGTTAGCTTCATCCAGAACTGAGAAGACCATAAACACAACGTAGAGATAGATGAGGGACACACCATACCACCTGAAGGTCAGGAAGAAAAGAAACATCACAATGTTAGTTGCAACTGAGATTGCAGTAAAACCTTCTCTGCTTTTCAGTTTACTAGAAAATAGCAGAAATCAGCTATTGTGGCAACCTGAACATTCAGATCCTGCCCACAACAAAAAAAATGGTCCTCACAGCATGAATGTATAGACATACCAATGAACAGCTGGGTGAGATAATAGACAATCCACTATTTATTATATTTATTGAGGCTGGCTGGCTGGCTCCACTAGGTGATAAACATTGTGAGGTTCCTTTTATGACAATCTACCACCACAGTGATAAATCACAACCANNTGTGATTTCTGCAANACAGGAAAGCACAACTGCTTGTATAAAGTCGCATGGGGTGTAGTTCATTTGTCCCAGCAATCTGGTGAATGATATATGTGCATGCCATAGCCATTGTTGAGGAAGCTCAATGAAGACAAGTCTCACCAAGCCATGGCTTTTGTGCACTGAATGTCATCTTGCGTGTGCATGTGTTCATGACGTACCTTGGAGGGTTAAACCTGGACACCACAAATACAATGAGTGTGGAGACCAATGAAATTGCCAGAAAGATCCACGATAACTTTACTTCATTAAAAGATGATTGATGAATGTCGAACAAAAATGTGTGACCACTTTTTGTAGCAAATGTGACCTTTGGAGAGAGAAAACAATTATATGACACAATTATATGACACAGACTGAACTGCTTCAGCCATCAGTGTTTACACTACTATGCTCTGCATGTCCTATTTGTATTGTAACTACTACCTTTATAATATTGCCAAACTAATTCTGACCTGGCTGGTCACTATATGCAGGTTAGGCTGTACATATATTATGGTATAGATCAAGGAAAATGTCCTACAAATCCTAAACAGTCACAGACAGCTAGAGCCTGGCAGAAGACTCCCTGATACAGTGGCAACAGCTGCAGCACACAACAGCTGGAATGGGCTAGGAAAGAATGCTAATCGAATTACTTTACCACCACACAATGCTGCTGAAAGATTTCTTAAAGCCTACATATGACTGTATCAGTTCTCAGTACACATACATATTTATCAGTGGCCAGTGATTGATGGTCAATGAAAAATGGTGGTGATGATGTGCATCTGGTAGTGTTTAGGCGGTATGCTTTGGTGGTGAGAGCCCATCCTACATAGAGCCCATCCTATCACTAATATCAACTATCAAAGCAGTTTATAGCTCAAATAGTAGTCATCAACTCCCATTTCTTGACCTTACCTCAGCACGCATATGGACACACCTAGGCACACTCCTAGGCATGCATATGGACACNCCTAGGCANGCATAGATGGACACACCTAGGCACACCCCTAGGCATGCATATGGACACTCCTAGGCACACTCCTAGGCATGCATATGGACACGCCTAGGCACACTCCTAGGCATGCATATGGACACTCCTAGGCATGCATATGGACACTCCTAGGCATGCATATGGGCATGCATATGGACACTCCTAGGCATGCAnCAGCCATATCCAGTGCACGTACAAGGGCATTAATTGGAGTCCGCGCACAAGTTGCGCTTAACAATAACAGCAATATGTACAAATTTGTTGCATTACCGGCTGGAGCCCCTGAGCTGGTACGTAGCAGCCAGAAGGACCGCTTCTATGCACATTATCTGAATGGTCTGCTCTCAGACACTGCTCGACAGCTCTTGCCTCTCCGCCACTGGATCCAGTATCAGAGAGAATTCCAGCTACTAGCTGAGCTAGCATACTACGGGCTAACTACTGTGCATGGAAACCAAACCCTTGGCGANGAATACTGCAACATTGTACAAGTAGCATCCAGTGCGGCTCCAACTATCGCTAGAAGAACGGGCGCGGTTCTGGTTCAGGCATTAGCACCCTATGTATTCGAGAAGCTATTGGAACATATCCACTGGAAACTGACCAATCGNGCATTCTCTCAACGTTTGAGCGACGAGGCGTATGAACGCTTAACGAAAGTAGTTGAATTCTGTGAGGAGACAGTCTCAACACTAAGGAAGCTGCATTTGGCGATATTCTACTTACAAGGCACATTTCACCAGTTTGGTAAGCGGTTGACGAGCATTCGATACATTTTGTTGCGATACAGTGGAGATGGCACAGAACCTCTCATCGATGAATGTATACAAAACATTAGGATGGTTAGTACTACTCCAAATCGTTGTGGGACTGGGCAAGCAGTTATGGAAGAAAGGATTCCAACGTTCACAGACCCGTGGGCATATTCAGGATGATTGTAGATTGCTGTTTGCTCGCAGAGATCGTACACACACTGACATTAAATGTCCCCTTTGTTTAGAACACTGCACTTCTCCTACAACCACATTGTGTGGTCACATATTCTGTTGGCAGTGTGTGGCAGAATGGACCTCTGAAAAGATGGAGTGTCCGTTGTGTCGAAGGGCAACACTGCCCCAAGAACTGGTTTCATTGCAGCATTTTGCAAATCAGTAATGTAATAGCACAAACCAATTTCATTTTGTGGTATGCTGTCATTAAATTTGCAATGAAGTCCAATTGAAAACTATCATCAGTGGTTAGAAAGCATATGTTGTGTACTGCAACAAAGCCATGTGGGATTTTAGTACACTGTTATTTTAATTTTAAGGGGACTTGCTAAAAATTACTTCCCATGTAACTGTTAACGTGTATAAATATTGACACCTATATGAACGTTAACTAAATGCAGCAAAAAGCTATTTTTAATGGCAGTGCTATGTTTCGTTTTAACACATGCGCATCTAGCTTAGTAATCTGTCATCCTGGCTTAGTAATCTGTCATCCTGGCTTAAGATTCACACCTAAGCTATTAGTTGAGGTGATGGCATATGGGGTCAGAGGATATTTCAGAGAGGTGTGTATGCACTTATATTTTTAATGCACTTATATTTTAATGCACTTATATTTTTAATGCACTTATATTTTAATGCACATTTATTTGCACAACAATCACATATAGTGTAATTTACGATTCATCAGTATGTGGAATTATTCAAGAGTTGCTGGGAGATTCTTTATGAGCAACACTTGTCAGTTTCAAGCAAATCTTGGAGATAACTGATAGCATAGTCCACCTCATTCTCGGCACAGCACAGCACAAGGCCTGGTCGTGGACCTCCATGGAGCCTTGCTGATATCAGATACACATCACCAAGGTTCAAGGTGTAGGCTGTGTGCACTTCGACTAGACCCTCAGATGGCAGCTTTCCCTCTACATCCACAAGGTAGTGTGATGCACTGTGTGGTGTGTGAGGCAGGCATTGATCACCAGGGAGCTTCAAAGACTTGGGAATATACATTCCATCGTTTTTTATCACTCTAATAATCCGCATGGAATGAGTGAATTGTCCGTCCTCCGAAGTAGACAGAGCTACCTCGCCTGTGAACACTGAAAGCAAACGCGATGATACGTACAAAAACAAAACAGCATTGGAATCCGCCCCTTTGTGGTATCTTAAGGTCNGCATACACGGGGCGACTTCCTGCCGACTTGTCGCATGCAANNNNNNNNNNNNNNNNNNNNNNNNNNNNNNNNNNNNTGTTTAGTTTACTGATGGTTTGCGGAACATTTGTCCATATTAAAGGCCGGCATACACAGGGCGACTTCCTGCCGATTTG>scaffold3895|size41610GAGGGAGCTTAGTTTCTGTNNNNNTCACTTCGTTCAACCAACAGAAACGTGTATTTGCCATCAGGCTTTAAAAGCCCAAGTCAAGTTGTCCTATATGACTTGTATGGACGTTTAATTTGAAGAACCTTAAAGGACTTTTCTTCAATTTNACCTGAGANGCTTGGAGGNNAGCTGATTAGTGATTGAAATGGGATTTCACCAGCAGAGTCTGCTATTGCCTCTTTGAGGTATTGGAGCACTGGTGACATGAATAGCATTGGAGAAAGCACAGAAAGAGTGGATAGAGACTAATGTCAATCCAAGACCACTATACCATACATGTAGTTGAACTTATTTAATTGCCATAGGTGTTAGTTCAATGGCTGCACTTTCCTCTAAACGGTGGAGGATATCCATAAAATACTTTAGGGAAGAATCACAAGGAGAAATGAAGCAACCTTGATGGGACAGCTTACAGAGCTTGCACAGTGGCAAAGCAGCAAGATGGACAGATGCAGGTCACACATTTGGTTAAAGTAAAAAGACGGAGAAGAGAATAAGNTCTNCTNTTAGGGATTAATAATTTATTCCATGATATTGAAAAAGGACACACAAGGCTTACAATGAGCCAAATACATTACAGTTCTTAAGTATATTGATATAGTGGATGCNNTGACAACATTATTCGATAGGACATTCTCTGATGGGACAACTCTCTCCACAAATGGATGTAATTTTGTGCTGCATTNNAAACTCCTTATTTTGTCCAACAGTCAAAGATAATATTCTGACTTCACATGTCAAGTATGCTGGCATCTAGGATGGCGATTTAGACACTAGGAAGACTGACTCAACATGCTAAGCCTTGAGTTCCTTCAGTTTCTGTGTACCATTTACGATCTGTGTTTATTGTATCAATTATACTACAATCTGATCCATTATGATGTCAGATGATAAAGTAGGCCAAACTCCGAGATCTCAAATTGTGGTCAAAAGATATTGTAATACATTTAGGGGATGCACTTCTAAGTTGCCAAACGTCACTCCAATTGCAAACATTTTCGAGTGCACTGTCCATTATTGTGCAGTCCGTATACGTGGAATGCCTAAAGAATAGCATAGAAAATACGATACAGAAGGGATGGGCGGGGAAAGAACAATNNNNNNNNNNNNNNNNNNNNNNNNNNNNNNNNNNNNNNNNNNNNNNNNNNNNNNNNNNNNNNNNNNNNNNNNNNNNNNNNNNNNNNNNNNNNNNNNNNNNNNNNNNNNNNNNNNNNNNNNNNNNNNNNNNNNNNNNNNNNNNNNNNNNNNNNNNNNNNNNNNNNNNNNNNNNNNNNNNNNNNNNNNNNNNNNNNNNNNNNNNNNNNNNNNNNNNNNNNNNNNNNNNNNNNNNNNNNNNNNNNNNNNNNNNNNNNNNNNNNNNNNNNNNNNNNNNNNNNNNNNNNNNNNNNNNNNNNNNNNNNNNNNNNNNNNNNNNNNNNNNNNNNNNNNNNNNNNNNNNNNNNNNNNNNNNNNNNNNNNNNNNNNNNNNNNNNNNNNNNNNNNNNNNNNNNNNNNNNNNNNNNNNNNNNNNNNNNNNNNNNNNNNNNNNNNNNNNNNNNNNNNNNNNNNNNNNNNNNNNNNNNNNNNNNNNNNNNNNNNNNNNNNNNNNNNNNNNNNNNNNNNNNNNNNNNNNNNNNNNNNNNNNNNNNNNNNNNNNNNNNNNNNNNNNNNNNNNNNNNNNNNNNNNNNNNNNNNNNNNNNNNNNNNNNNNNNNNNNNNNNNNNNNNNNNNNNNNNNNNNNNNNNNNNNNNNNNNNNNNNNNNNNNNNNNNNNNNNNNNNNNNNNNNNNNNNNNNNNNNNNNNNNNNNNNNNNNNNNNNNNNNNNNNNNNNNNNNNNNNNNNNNNNNNNNNNNNNNNNNNNNNNNNNNNNNNNNNNNNNNNNNNNNNNNNNNNNNNNNNNNNNNNNNNNNNNNNNNNNNNNNNNNNNNNNNNNNNNNNNNNNNNNNNNNNNNNNNNNNNNNNNNNNNNNNNNNNNNNNNNNNNNNNNNNNNNNNNNNNNNNNNNNNNNNNNNNNNNNNNNNNNNNNNNNNNNNNNNNNNNNNNNNNNNNNNNNNNNNNNNNNNNNNNNNNNNNNNNNNNNNNNNNNNNNNNNNNNNNNNNNNNNNNNNNNNNNNNNNNNNNNNNNNNNNNNNNNNNNNNNNNNNNNNNNNNNNNNNNNNNNNNNNNNNNNNNNNNNNNNNNNNNNNNNNNNNNNNNNNNNNNNNNNNNNNNNNNNNNNNNNNNNNNNNNNNNNNNNNNNNNNNNNNNNNNNNNNNNNNNNNNNNNNNNNNNNNNNNNNNNNNNNNNNNNNNNNNNNNNNNNNNNNNNNNNNNNNNNNNNNNNNNNNNNNNNNNNNNNNNNNNNNNNNNNNNNNNNNNNNNNNNNNNNNNNNNNNNNNNNNNNNNNNNNNNNNNNNNNNNNNNNNNNNNNNNNNNNNNNNNNNNNNNNNNNNNNNNNNNNNNNNNNNNNNNNNNNNNNNNNNNNNNNNNNNNNNNNNNNNNNNNNNNNNNNNNNNNNNNNNNNNNNNNNNNNNNNNNNNNNNNNNNNNNNNNNNNNNNNNNNNNNNNNNNNNNNNNNNNNNNNNNNNNNNNNNNNNNNNNNNNNNNNNNNNNNNNNNNNNNNNNNNNNNNNNNNNNNNNNNNNNNNNNNNNNNNNNNNNNNNNNNNNNNNNNNNNNNNNNNNNNNNNNNNNNNNNNNNNNNNNNNNNNNNNNNNNNNNNNNNNNNNNNNNNNNNNNNNNNNNNNNNNNNNNNNNNNNNNNNNNNNNNNNNNNNNNNNNNNNNNNNNNNNNNNNNNNNNNNNNNNNNNNNNNNNNNNNNNNNNNNNNNNNNNNNNNNNNNNNNNNNNNNNNNNNNNNNNNNNNNNNNNNNNNNNNNNNNNNNNNNNNNNNNNNNNNNNNNNNNNNNNNNNNNNNNNNNNNNNNNNNNNNNNNNNNNNNNNNNNNNNNNNNNNNNNNNNNNNNNNNNNNNNNNNNNNNNNNNNNNNNNNNNNNNNNNNNNNNNNNNNNNNNNNNNNNNNNNNNNNNNNNNNNNNNNNNNNNNNNNNNNNNNNNNNNNNNNNNNNNNNNNNNNNNNNNNNNNNNNNNNNNNNNNNNNNNNNNNNNNNNNNNNNNNNNNNNNNNNNNNNNNNNNNNNNNNNNNNNNNNNNNNNNNNNNNNNNNNNNNNNNNNNNNNNNNNNNNNNNNNNNNNNNNNNNNNNNNNNNNNNNNNNNNNNNNNNNNNNNNNNNNNNNNNNNNNNNNNNNNNNNNNNNNNNNNNNNNNNNNNNNNNNNNNNNNNNNNNNNNNNNNNNNNNNNNNNNNNNNNNNNNNNNNNNNNNNNNNNNNNNNNNNNNNNNNNNNNNNNNNNNNNNNNNNNNNNNNNNNNNNNNNNNNNNNNNNNNNNNNNNNNNNNNNNNNNNNNNNNNNNNNNNNNNNNNNNNNNNNNNNNNNNNNNNNNNNNNNNNNNNNNNNNNNNNNNNNNNNNNNNNNNNNNNNNNNNNNNNNNNNNNNNNNNNNNNNNNNNNNNNNNNNNNNNNNNNNNNNNNNNNNNNNNNNNNNNNNNNNNNNNNNNNNNNNNNNNNNNNNNNNNNNNNNNNNNNNNNNNNNNNNNNNNNNNNNNNNNNNNNNNNNNNNNNNNNNNNNNNNNNNNNNNNNNNNNNNNNNNNNNNNNNNNNNNNNNNNNNNNNNNNNNNNNNNNNNNNNNNNNNNNNNNNNNNNNNNNNNNNNNNNNNNNNNNNNNNNNNNNNNNNNNNNNNNNNNNNNNNNNNNNNNNNNNNNNNNNNNNNNNNNNNNNNNNNNNNNNNNNNNNNNNNNNNNNNNNNNNNNNNNNNNNNNNNNNNNNNNNNNNNNNNNNNNNNNNNNNNNNNNNNNNNNNNNNNCTTCCCCAGAGACATTTGACACGAATATAAAGTATGCATATTTATCAAAAATGGAGACTTTGTGCGTTAATGGATATTGAGTAATAAGGTGGCTATAGAGAGATTGAGTGAACAATGTTTCCTAACCTCACGTCTAGTATTTTGTNNNAGTCATGTAGACGCACCTGAGTGTTAAAGTTGAGTCCGGAATGACNAAACCCAATACCACANTCACACGAGGCCAGCAGAAATCCTTGTCCAGAACTTCCCCAGAGACATGCCAACAGCTTTTGACATCTGCGTAACCTCACCACTTAATATACNTCCTTTTTTAGAAGCAGGGGGTTTGCACAGGTGTGCAGCTCAAGCTGGAAAGGCCAGGTAGCACTTAGCTAATGAAGACAGTACANNTGTNNGNACCTGGGNTCGGATTGTGTTCCACTGGTGGCTGAAACATATGAAGCTTGGGGACCTGAAGCCATTCTCTCCTTCCAGCTGGATTTGACGCTTGCCATCAGGCTTTAAAAGCCCAAGTCTATTGTCCTATATGACTTGTATGGACGCTNNTTAANNTGAACNAACCTTAAAGNACTTTNCTTCAATTTTACCTGAGAGGNTTGGAGGGGAGCTGATTAGTGATTGAAATGGTAGCAGAGTCTGTTTTGTCTCTTTGAGGTGTGGGAAGCCTTAGGAGCACTGGTGACATCAATAACATTGGAGAAAGCANTGGANGAGTGGATAGAGACTGATGTCAATCCAAGACCACCATGCCGTAGTGGGAGTTGAACTTGTTTATTTGTCAAAGGTGTTAGTTCAATGGTTGCACATTCCTCTAAACNGTGGAGGATATCCATAAAATACTTCAGGGAAGAATGACAAGGAGAAATGGAGCAACCTCGATGGGACAGCTTACAGAGCTTGCACAGTGGCAAAGCAGCAAGATGGACACCTGAGGGTCACACAGCTGAAGTAAAAGACAGAGAAGAGAACAAGCCTGCTTTCTTAGGGATTAATAATTTATTCCATGATGGAAAAAGGACACACAAGNCTTGGCAGAGGTAAGCTGCCATGCTCTTACAGTGAGNCTAAAACATTACAGTTCTTAAGTACATTGATATAGTGGATGCCATGACAACATTATTCGGTAGNACANTCTACAATGGGACAACTCTCTCCACAAATGGATGTAATTTTGTATTGCATCCAAAACATNCAAANCCCNTTATTTTGTCAAACAGTCAAAGATAATATTTTGACTCTNCTTACTTGAATAGTGATTGAAAAGGGATGTCTATCAGATTATAGTATATTTGATTAAAATAACCACATATCATAGATGGTATAACAAAACAAGGTTTAGAACGTTGAGTCGGTCTTTATAGTGTCTAAATCGCCATCCTAGATGCCAGCATACTTGACATATGAAGTATCGTTGTAACCATTATGATGTCAGATGATAAAGTAGGCCTAACTCAGAGATCTTGAATTGTGGTCAAAATACATTTAGGGTATACAATTCTAAGATGCCTAAGGTCACTCCAATTGCAATCATTTTCGAGTGCGCTGTCCACTGTTGTGCGCAGTCCGTACATGACGAATAATTGCATAGGAACGCCATACAGAAGGGATGGTACTAGAAAGATAGTTTATGCATAGTACAAAAAACAAAAAGATGAAAAACATACCCTTGAGGTACTCCTGATATAAATGGTAGCAGTGAGGACATAGCTTCATTCATGCCCTTTGAGTTTGATAGATAGAAATGAAATAATTCACCACTTTGTCTACGATAGGCATATGACTTTATGAGCAGATCCACAATCAAAAGCTTGGAATGCATGTCTCAACCAATTACTGACATTTTAGTCCATTGGAGTGGAGCTTGTGGGCCTCAGCTGAAAGGGCTGCTGCACCAGAAAGTTGACTCACCCAAGCTGCCAGGGCAGAGTTGTGATGTCATGGTAATGTCATCTAATTGTTTAATAATTATAATGTAAGTTGTATATACACGATAAGGAACCAGGCAATTGGTGTTGAATGAATGCATGTAATGNAATCTCTGTTTCGATATTCTGAAGTACAATATAGGCATTCGATGTAAATGAGACGTTGAAGCTCCAGGTGCATATGTAGTGTTCCACTGTTTCAGCAAGAGGGAGGGGGAGAGCCAATGTAACGAAGCACTGTGATTTAGACCTCTCACTATGTATCCATCTATCATCACCACTAATTCAAACTGATGTCGATATTTCACTTGTTAGCCCACTGCTGTTCTTTTTATGCATAATTGGATGGGATCGCGCGCCCGGACCTAAAAGGACACCATTCATTAGTTCTCCCAAANACAACCAATAGTAGCAATATAACTATACAGCATGCTCATCTTTTGATCATGGTGTAGAACGATTGGAGGCATGACCTATCACGGCGCGACAGTGTACCGTATGCAGCCTTGTATNNNNNNNNNNNNNNNNNNNNNNNNNNNNNNNNNNNNNNNNNNNNNNNNNNNNNNNNNNNNNNNNNNNNNNNNNNNNNNNNNNNNNNNNNNNNNNNNNNNNNNNNNNNNNNNNNNNNNNNNNNNNNNNNNNNNNNNNNNNNNNNNNNNNNNNNNNNNNNNNNNNNNNNNNNNNNNNNNNNNNNNNNNNNNNNNNNNNNNNNNNNNNNNNNNNNNNNNNNNNNNNNNNNNNNNNNNNNNNNNNNNNNNNNNNNNNNNNNNNNNNNNNNNNNNNNNNNNNNNNNNNNNNNNNNNNNNNNNNNNNNNNNNNNNNNNNNNNNNNNNNNNNNNNNNNNNNNNNNNNNNNNNNNNNNNNNNNNNNNNNNNNNNNNNNNNNNNNNNNNNNNNNNNNNNNNNNNNNNNNNNNNNNNNNNNNNNNNNNNNNNNNNNNNNNNNNNNNNNNNNNNNNNNNNNNNNNNNNNNNNNNNNNNNNNNNNNNNNNNNNNNNNNNNNNNNNNNNNNNNNNNNNNNNNNNNNNNNNNNNNNNNNNNNNNNNNNNNNNNNNNNNNNNNNNNNNNNNNNNNNNNNNNNNNNNNNNNNNNNNNNNNNNNNNNNNNNNNNNNNNNNNNNNNNNNNNNNNNNNNNNNNNNNNNNNNNNNNNNNNNNNNNNNNNNNNNNNNNNNNNNNNNNNNNNNNNNNNNNNNNNNNNNNNNNNNNNNNNNNNNNNNNNNNNNNNNNNNNNNNNNNNNNNNNNNNNNNNNNNNNNNNNNNNNNNNNNNNNNNNNNNNNNNNNNNNNNNNNNNNNNNNNNNNNNNNNNNNNNNNNNNNNNNNNNNNNNNNNNNNNNNNNNNNNNNNNNNNNNNNNNNNNNNNNNNNNNNNNNNNNNAGGGATGGTGATTCGTCTACTAATATAGAATTTAGCACATGTTCTGGCCTTGTTACCAAATATCCAGATGGACGCCTAGACCAAGCTCACTGCTGTTATGGCTTCAAGTCACAATGTCACTCTCAATGCAGTGATTCATCGCCAATAATAGATAGCATCTCCTATGCCACCACTAATTACATAAAGCTTCACGTTAAACAACCTCTAGTCATAATACAATTTGAACAAATTCGGGCAGTAAGTTCCTAAACAGCGCCCTTCCATAACCGCTATCATTAGGCTTTTAATTCTGACAAAGCTATACATGTAATGCAATAAACCTCATCTTATCTGATCATATCCAAAACCAGAGGAGGAGNANGAGACTGATTTTATTTTTTTCCTCCTGGGTTCGAGTGTGGAAACAAGTAGTGAGCGCTATGCTCTGTTGTCNGAGCTTTCTAAGCGTCGGCAGTTTCACTACATTGTGTAGGTATTCGCCAGCTCTATCCAAATACGCTCGCTAAACTCCAATTGAGTCTACGATGTATCTGTGGTAGCGGAATCTTCTGTATTTTGTTAATGTACTCACTTAACCAGTACATTAACAAAATACAGTTTGGTGGTAATGGTTCTTGCAAGGACTCTAGCTTGAGGTCTGCACTCTGCTAGTTTGGAGCTAATCTGAGTGCATGAGGTCCGTGCGCTCCGTATTCAAGTTCAAATAGCTTCTTCAACCCATGCATGCCCGCCTTCAGGGTGCAGAAGCGGATCCACACACAAAATTATGACGACACACTGTTTTCTTTTTAAAGAATAAAACTGAGTGAAACAATGTAGATGTAGGATTTTTATGCACGTATTGAGTAAAAGTAAGGGGGAAAGAAAGATTACGGATATTATGCCAATTATGAATTTATAAATATTACAGACCTACATCAATTTTAGCAAATATCGTATATATGATTATTGTTATACCATCTTTGGATTATTGTTTCTTGAGCTTACTAAATTTAGATGTGTAATATTTATAAATCCAAATTTCAGTTATCCTTATTTTTACTCAATTGGTGCATACAAATCCTACAATATGTGAGGTTAGGAAATGTTTCTCACTCAGTCACAGGCTATAACTTCTCTGTAGTGTTTCAGTGCACCTTAATTTCTCAATATCCATTCATATGTGTGCTCTNNNNNNNNNNNNNNNNNNNNNNNNNNNNNNNNNNNNNNNNNNNNNNNNNNNNNNNNNNNNNNNNNNNNNNNNNNNNNNNNNNNNNNNNNNNNNNNNNNNNNNNNNNNNNNNNNNNNNNNNNNNNNNNNNNNNNNNNNNNNNNNNNNNNNNNNNNNNNNNNNNNNNNNNNNNNNNNNNNNNNNNNNNNNNNNNNNNNNNNNNNNNNNNNNNNNNNNNNNNNNNNNNNNNNNNNNNNNNNNNNNNNNNNNNNNNNNNNNNNNNNNNNNNNNNNNNNNNNNNNNNNNNNNNNNNNNNNNNNNNNNNNNNNNNNNNNNNNNNNNNNNNNNNNNNNNNNNNNNNNNNNNNNNNNNNNNNNNNNNNNNNNNNNNNNNNNNNNNNNNNNNNNNNNNNNNNNNNNNNNNNNNNNNNNNNNNNNNNNNNNNNNNNNNNNNNNNNNNNNNNNNNNNNNNNNNNNNNNNNNNNNNNNNNNNNNNNNNNNNNNNNNNNNNNNNNNNNNNNNNNNNNNNNNNNNNNNNNNNNNNNNNNNNNNNNNNNNNNNNNNNNNNNNNNNNNNNNNNNNNNNNNNNNNNNNNNNNNNNNNNNNNNNNNNNNNNNNNNNNNNNNNNNNNNNNNNNNNNNNNNNNNNNNNNNNNNNNNNNNNNNNNNNNNNNNNNNNNNNNNNNNNNNNNNNNNNNNNNNNNNNNNNNNNNNNNNNNNNNNNNNNNNNNNNNNNNNNNNNNNNNNNNNNNNNNNNNNNNNNNNNNNNNNNNNNNNNNNNNNNNNNNNNNNNNNNNNNNNNNNNNNNNNNNNNNNNNNNNNNNNNNNNNNNNNNNNNNNNNNNNNNNNNNNNNNNNNNNNNNNNNNNNNNNNNNNNNNNNNNNNNNNNNNNNNNNNNNNNNNNNNNNNNNNNNNNNNNNNNNNNNNNNNNNNNNNNNNNNNNNNNNNNNNNNNNNNNNNNNNNNNNNNNNNNNNNNNNNNNNNNNNNNNNNNNNNNNNNNNNNNNNNNNNNNNNNNNNNNNNNNNNNNNNNNNNNNNNNNNNNNNNNNNNNNNNNNNNNNNNNNNNNNNNNNNNNNNNNNNNNNNNNNNNNNNNNNNNNNNNNNNNNNNNNNNNNNNNNNNNNNNNNNNNNNNNNNNNNNNNNNNNNNNNNNNNNNNNNNNNNNNNNNNNNNNNNNNNNNNNNNNNNNNNNNNNNNNNNNNNNNNNNNNNNNNNNNNNNNNNNNNNNNNNNNNNNNNNNNNNNNNNNNNNNNNNNNNNNNNNNNNNNNNNNNNNNNNNNNNNNNNNNNNNNNNNNNNNNNNNNNNNNNNNNNNNNNNNNNNNNNNNNNNNNNNNNNNNNNNNNNNNNNNNNNNNNNNNNNNNNNNNNNNNNNNNNNNNNNNNNNNNNNNNNNNNNNNNNNNNNNNNNNNNNNNNNNNNNNNNNNNNNNNNNNNNNNNNNNNNNNNNNNNNNNNNNNNNNNNNNNNNNNNNNNNNNNNNNNNNNNNNNNNNNNNNNNNNNNNNNNNNNNNNNNNNNNNNNNNNNNNNNNNNNNNNNNNNNNNNNNNNNNNNNNNNNNNNNNNNNNNNNNNNNNNNNNNNNNNNNNNNNNNNNNNNNNNNNNNNNNNNNNNNNNNNNNNNNNNNNNNNNNNNNNNNNNNNNNNNNNNNNNNNNNNNNNNNNNNNNNNNNNNNNNNNNNNNNNNNNNNNNNNNNNNNNNNNNNNNNNNNNNNNNNNNNNNNNNNNNNNNNNNNNNNNNNNNNNNNNNNNNNNNNNNNNNNNNNNNNNNNNNNNNNNNNNNNNNNNNNNNNNNNNNNNNNNNNNNNNNNNNNNNNNNNNNNNNNNNNNNNNNNNNNNNNNNNNNNNNNNNNNACCTCAACACCTCACACACACACACACACACACACACACACACACACACACACACCTCCAAAGCTTTCTTGACACAAGCTGCCGCCACCTCTTCTGATCTGCAGCTGATCCAGAAGACCCCACCTCTGAAATAATNGTAGAAGGAGTGAATGAAAGTTTTCACAGGATCTGTAAGCTCTGTGCCTTCATAGCCCAGAAGGATCTGTTTTGACAAAACAAAACACCTGAATGAAGACATACTCACCCATCTCGTAACATACTTGAACATTGCTAGGCTGTGATGTAGTTTGAATTAGTCCTTGCTCCAGTTTCTCCACTACAGCTACCTGGGGAAAGACACACCTTGGTCCCAGAGATGCAGCAAGCAATGCTTCCTCAGTGGTCTTCGTTCTATGGAGGTAAGTCTCCCTCCCAATCAGCTGGAACCCTCCCCAGTGTGTGTACTGGGAGTACTTCCTGAAGCACCTCACACTCAGAGTGGCCTTCTGGAGGGCATGGGTGGTGCCCAGGCCATCCACCAGGTACTGGTAGAAGAACTGCATGAACATGCCTGCACTCTCGTCCGGGATCCTCCACAGAGTGGTGAGGACGGACTGGGCACCAGCAAGGAGGAAGGCCCTTCCCATCCCAATGATGCCGTCCGCACGCACCATTCCACGACCGCTGTCACAGCTGCTCAGAACGACCAAGGCCGCTTTGATGTTCATCTGTTCCACCTCACTTGGTAGAAGAAGGATGTCTGCAGATTTCGATGGCTTTCCAGTTGATGAATCAGGACCTGCAAAGGCTAAGAAACCTGATATGGCACTACCATGTGTGGCAATGTGGATCACCTGAGCAGAGCTTAGCTTTGTTACCACGGCAACCTTAGTGGCCTGCTCATGCAGGAGTGGTTGCACTTTAAGAAGCTGGGAGATCCACTTGGCCTCATCAGTGGCATGTGGAAGTCTTCCCAGGCTCCATTCCTCATTGTTGTGTTCAAAGATGGGGATGTGAGGGTTACCAACCACACAGGCTGTCCTACTATCGTACATCACTGCCCTGGTGGGGTGGGCTTCATTTAAAATACCCATGACCAACAGGGAGGGCATCATCTGAATGCTGAAAGTGCTACCAAAGGTATTGTTGAAATCCCCCTCAGGATGGAGGGCAACAAATGGAATAAGGCTGGTGTAGCTATCTGGAATTAAAATCAGGTTTTTCAAGTCTTGCAGTTTGCATTTTCCAAGCATGTCAAGGATTGGAAGGACAACTACATCAAATAGCACTGTTAGTGGAGAGGTCTGGGAACTAGACACATCCTGGAATAGCTCCAGATCNTTGCCTACCACAGCCTCCTGCACACTCTGGCGGACATATTGGTCAAACGTCTTGCCTCCAAACTTCTCTTTCTCCAGCTTCTCCACCTCAACTACTCTGCAATCTATTTGGACATGACCATATATGGGAATAATCAGCCACACTATCAGCTTGGCACCTGTGTAGGATAAGTACACAACTGGATGCTCCTGGGATTTCACAATGTATCGAATCTGCTCCAGGGAGAGGGGCGGGGTCAGGTGGTTTACACCTCCCCCCATCTTCTCCAGCAGTCTCTCCCCTAGGGTCCTAGCTCTGCACTGCTCAGCAACCACAAGCGCTTCATCCATCTGCTGCAGACCAATCAGACAGTCCTGCAGCTTGTGGAAGGTACGGGAGCTGNTCTCAATCAGAGACACAGAGAGAGACAGTCCTTGCATGGACCCTTTGGTGCTCTGCAACACCTCATTGCACAGCTCAATAGCCTGTGTCAGGTCACTAGCTCCCAGCCTGTACAGCTCTGTAGGTGTAAATCCAGGACTGGGTACCATGGTGCCTGGATTGCACAAGACTTGTAGGTTNCTGCTAATCCCACTGGTTGTGCCATTCTGTCCTGACCTCAATTGCTCCGCCCACTCAAGGTAGGCACACCCCCTGTTGTGATTTGCTGTGGACAGCACTTGCCTGTCTTTGGAGAGTCTGAAGACTTCTGAATACTGCTCAACAGCTCTCTCGTACTTCTTGCTCAACATCAAGAGGTTGCCTAGGTTACCTCGTGCCCTTGCTTGACCAGCCACATCATTTCCATATATGGCCTGATCCAGCGCCAAGTTGAAGTTCTCCTCTGCCCCCTTGAAGTCCTCCAAGCTCTGGTAGGCTGTCCCGAGGTTGTTGTAGGCTCTGCTGATGGCCTGAGGGGTGGACTCATGCTCTATGGTGAGAGCCAATGCCTTCTTGAGGTGATGGAGGGCTGCATCCTTCTGGTGCAGACCCAGGTGCGCATTCCCCATGTTCCCATGGGCCCAGCCGAGGGAGATGAGGTCATTGAGCTCCTCCCCTAGCTTGACTGCCTCTTTGTACTCTTGCAGTGCCCCAGTGTAGTCCCCCATGGACTGGAGGAGGTTCCCCAAGCTGTTGTGGACAGGGAGCTTGCTCTTGATGTCTGGTGCATGGTGGATGGCATTTCTGAATGCCTCCACTGCTTGGAGCATCTGAGAGGTCTGCTTTAGAGCTGCCCCTTGCTTAGCATAGAGTGCTGAAACACTGGGCATTACCAGTTTGAAGAGGGTACTTGGGTTATTGGTTCCGTAGTTGTGTATGGCCTCAAGATAGTGATAAGCTGCATTGCTGTAGTTGGATTTAATGGTGGAGATGTCTCCTAGGTAGACATGGGCCAGAGAGATGTCTGCAGGTCTGGCCAGAGTGAGGGCCAGGCTCCTGTACTCCATGAAGTGTTTTGTGGCTTCATGGTGGTCGGCCATCTTGTAGTGGGCGAGACCCAGGCCAAAGTGTCTGAACAGCAAGGCTTGGCAGTGTTGAGTGGCACAGGTGTGCTGGGTGATCTGCTCCAACAGAGGGATCATGTGACTGTAGTCCTCAGAACACAAACACTCCGTGGCTGAACTGAATATGGCTTTGCTTTCTTCACAAAGAATGACATCAACTGTTACAGTGGATGAAGGAATATGAATTCTTGGAGGTGTGGTGATCAGTGCTATTGGNGCAATGGTTGCAGGAGTATTTCTTGCAGCACGACTCGATTTGAGCAAAGGTTTGTGTATTAGCTTTGCAGTTGGTTTGAGNCATGTCTAGAATGTTGACTTGANNAAGGGGTCTTTGTGACATCGGGCATTGGGCATCAGTTCTTGGAGCTTGGCTTCGTCCACCCCCTCTGCCCGTGACCAGGTCANCCGCANCACCTTTGAACTCTCTGACAACATTGAGGGTAGTGTGTACCATCTCTGTGCTGGAAGGGGAGGGGTCTTCTGCCTCACATGCACATTCANAGTCCTTCTTTTGTGAACTTTGTCTTTGCAGCCCTTTCTTGTACCCACGAACAACAGCATCTTGAACTTTTACACAAGCAAGTGGAGCTGTTGGAGGATTACACAGTACTGAATTGTTGAGAGCAGCTGTACATGTACAGGTCATGGAAGCATTGCTCCAAGATAGGCATGACACATGATCTCCTTCAGGGTTCAGATTAGATGAGCATTCACCACTCTCATGTCCCAATTTACAAATTCTATTTGGCATGTCCTCTCTTGTCANCAACATTAAAATAAGCATCATTCATTGGGTGAGATGACTCCATTTGTGGTATACAGATGTTGCACGTCTTTTGCAGTGGAATGCTATTTGTTTCNAATGGGTTTTTTTGCATAGGAGGGACCACAGCTAGGGCTGAGTCAGTGGTCATCAGTGCAGTGGTTGGAGCAATGGTTGGAGCAATGGTTGGAGCAATGGTTGGAGCAATGGTTGGAGCAATGGTCTGGAGCAATGGTTGGAGCAATGGTTGGAGCAATGGTTGGAGCAATGNCTGGAGCAATGGTTGGAGCAATGGTTGATACATTTCCTGGAACATTGCCTGGTGTATAAATCTTTATTAAAAGTGAAATAAATATACTTTGANCTGCNGGCCCTTGTGGTTCAGAATTAACAGTTANACCANCATAANCTACACCAGCATAAGCTACACCAGCAACCACATCCTTGAACACACAAACCACTATGAACCAAACAGTATGAATAATCTCTTCCCTGGGGGATAAGGGATCTGTCCTCCACAGTGATGAGAGATCAGCCCCCTGGTGTCCAATAGCCAAATATTGCAAACCTGGCTGTTGGTAATATTTTTTATGTCTCAAAATGTAGGAATGTGCAGCCATTGCTCCAGTGCAGATGGTCCAGAGTGTAAGTAGTACACTGCTCCAGTACAGTTCAATGCACAGAGCACACAGTTCAATGTACAGAGCCCAAAGCAGTATATAGATGTCTTTAGTTGTATGTGTAACAAGAGATTTNGTGCTGTGAGGTTTGAAATGCATGTATTCATATGTTACCGCAGCTTTGGAGAAAGGTAGTGCATAGTTGATGTTTTTAAGTGTCATTGAAACATTGAAGTAGCTGTTTGTTAGTATCATTGCATCATTGGAGAAGATGTCTTTAAGTGTCATTGCATAATTTGAGTACATATTTGTTTTCATGAGTGTCATTGCATCATTGGGGTAGACGTTTTTAAGTTTCATTGCATCATTGGAGAAGATGNTTCTTTCTGTATTGGTAGAGAGGTCTTTCAGTAATTTAAAAATTATTTCATTTTTAAGTGTCATTGCATCATTGATGTTATTGAGAGTTGGATTTGAAACAGAGAGTACACCTGTGCCTTTTCCCTTCAATGCCGANNNNNNNNNNNNNNNNNNNNNNNNNNNNNNNNNNNNNNNNNNNNNNNNNNNNNNNNNNNNNNNNNNNNNNNNNNNNNNNNNNNNNNNNNNNNNNNNNNNNNNNNNNNNNNNNNNNNNNNNNNNNNNNNNNNNNNNNNNNNNNNNNNNNNNNNNNNNNNNNNNNNNNNNNNNNNNNNNNNNNNNNNNNNNNNNNNNNNNNNNNNNNNNNNNNNNNNNNNNNNNNNNNNNNNNNNNNNNNNNNNNNNNNNNNNNNNNNNNNNNNNNNNNNNNNNNNNNNNNNNNNNNNNNNNNNNNNNNNNNNNNNNNNNNNNNNNNNNNNNNNNNNNNNNNNNNNNNNNNNNNNNNNNNNNNNNNNNNNNNNNNNNNNNNNNNNNNNNNNNNNNNNNNNNNNNNNNNNNNNNNNNNNNNNNNNNNNNNNNNNNNNNNNNNNNNNNNNNNNNNNNNNNNNNNNNNNNNNNNNNNNNNNNNNNNNNNNNNNNNNNNNNNNNNNNNNNNNNNNNNNNNNNNNNNNNNNNNNNNNNNNNNNNNNNNNNNNNNNNNNNNNNNNNNNNNNNNNNNNNNNNNNNNNNNNNNNNNNNNNNNNNNNNNNNNNNNNNNNNNNNNNNNNNNNNNNNNNNNNNNNNNNNNNNNNNNNNNNNNNNNNNNNNNNNNNNNNNNNNNNNNNNNNNNNNNNNNNNNNNNNNNNNNNNNNNNNNNNNNNNNNNNNNNNNNNNNNNNNNNNNNNNNNNNNNNNNNNNNNNNNNNNNNNNNNNNNNNNNNNNNNNNNNNNNNNNNNNNNNNNNNNNNNNNNNNNNNNNNNNNNNNNNNNNNNNNNNNNNNNNNNNNNNNNNNNNNNNNNNNNNNNNNNNNNNNNNNNNNNNNNNNNNNNNNNNNNNNNNNNNNNNNNNNNNNNNNNNNNNNNNNNNNNNNNNNNNNNNNNNNNNNNNNNNNNNNNNNNNNNNNNNNNNNNNNNNNNNNNNNNNNNNNNNNNNNNNNNNNNNNNNNNNNNNNNNNNNNNNNNNNNNNNNNNNNNNNNNNNNNNNNNNNNNNNNNNNNNNNNNNNNNNNNNNNNNNNNNNNNNNNNNNNNNNNNNNNNNNNNNNNNNNNNNNNNNNNNNNNNNNNNNNNNNNNNNNNNNNNNNNNNNNNNNNNNNNNNNNNNNNNNNNNNNNNNNNNNNNNNNNNNNNNNNNNNNNNNNNNNNNNNNNNNNNNNNNNNNNNNNNNNNNNNNNNNNNNNNNNNNNNNNNNNNNNNNNNNNNNNNNNNNNNNNNNNNNNNNNNNNNNNNNNNNNNNNNNNNNNNNNNNNNNNNNNNNNNNNNNNNNNNNNNNNNNNNNNNNNNNNNNNNNNNNNNNNNNNNNNNNNNNNNNNNNNNNNNNNNNNNNNNNNNNNNNNNNNNNNNNNNNNNNNNNNNNNNNNNNNNNNNNNNNNNNNNNNNNNNNNNNNNNNNNNNNNNNNNNNNNNNNNNNNNNNNNNNNNNNNNNNNNNNNNNNNNNNNNNNNNNNNNNNNNNNNNNNNNNNNNNNNNNNNNNNNNNNNNNNNNNNNNNNNNNNNNNNNNNNNNNNNNNNNNNNNNNNNNNNNNNNNNNNNNNNNNNNNNNNNNNNNNNNNNNNNNNNNNNNNNNNNNNNNNNNNNNNNNNNNNNNNNNNNNNNNNNNNNNNNNNNNNNNNNNNNNNNNNNNNNNNNNNNNNNNNNNNNNNNNNNNNNNNNNNNNNNNNNNNNNNNNNNNNNNNNNNNNNNNNNNNNNNNNNNNNNNNNNNNNNNNNNNNNNNNNNNNNNNNNNNNNNNNNNNNNNNNNNNNNNNNNNNNNNNNNNNNNNNNNNNNNNNNNNNNNNNNNNNNNNNNNNNNNNNNNNNNNNNNNNNNNNNNNNNNNNNNNNNNNNNNNNNNNNNNNNNNNNNNNNNNNNNNNNNNNNNNNNNNNNNNNNNNNNNNNNNNNNNNNNNNNNNNNNNNNNNNNNNNNNNNNNNNNNNNNNNNNNNNNNNNNNNNNNNNNNNNNNNNGTGTGTGTGTGTGTGTGTGTGTGAGAGATCAGGTTCCGTTGTTGTCCCTTGTTTTAGAGAACAGTGAACAGTGTTCTGCACCAAACCTTCCTTAGTGACAAAAACATTGTGGACTCTCCATTGAACATACATGTAGTCCTATGTTGGTGTGTATAGTTTACATAACCATTCTAAACATAACCATGCCCATATATATTGCCTTAATTCCAAATATAGCCATTGAGTTAATACATTACCTTTCTCAGTTTTCTGGTGTCATTCCATGTCCAATGATGTCTGGAAACCCTTCAATGGCAATTGTGATCACATGTGACATAGTACTAGACAAAAACACAGGATGCATGGTGCATGACTTTAAGTACATTATATCATGCTGTATTAGCAAGATTCACACATTGCATCAGCATCGTGTTGCTCAGCTGACCTTTGGTCAAGGTTGATTGCACATTTGAGATAATTTCATCAAAAAATTAATTAATATTGTGAATCAAGTCTACCTTCAAGCATTACAGCAATTATGGTGCCATTTAAAAGACTATACACACGTACACATGTACATATGCGCATAGACATGCTCATACATCATATACAACTATTATGTAGGAGATGGCAGAACAATGTTCCAATCCTGTACAAAACTAACTTTGGGTTTAAAGGAAAATTTGAGGACCTACTGGCAATACTCATGAACAAGCATTTGAGCATACTCCATACCATTCATACCATTGAAGGTCATGATATCAATGAGTGAACCATAATTGGAAGAACAGACATGCTCCCAAGTCTACCAGATCACATGTTGACAGCCACGTAAAATGCCAATTCATAAAAAATACACACTCAGTTTACAGAAGTTCTAAATGCAGTACTAATCTGAGACATACTAGTGATGACTAAAATACATCAAGCATTGCACAAGTTCTTGAATCTTTCTAGCATTAGATAGAACAGCTGGTAGCACTCTTCNNNNNNNNNNNNNNNNNNNNNNNNNNNNNNNNNNNNNNNNNNNNNNNNNNNNNNNNNNNNNNNNNNNNNNNNNNNNNNNNNNNNNNNNNNNNNNNNNNNNNNNNNNNNNNNNNNNNNNNNNNNNNNNNNNNNNNNNNNNNNNNNNNNNNNNNNNNNNNNNNNNNNNNNNNNNNNNNNNNNNNNNNNNNNNNNNNNNNNNNNNNNNNNNNNNNNNNNNNNNNNNNNNNNNNNNNNNNNNNNNNNNNNNNNNNNNNNNNNNNNNNNNNNNNNNNNNNNNNNNNNNNNNNNNNNNNNNNNNNNNNNNNNNNNNNNNNNNNNNNNNNNNNNNNNNNNNNNNNNNNNNNNNNNNNNNNNNNNNNNNNNNNNNNNNNNNNNNNNNNNNNNNNNNNNNNNNNNNNNNNNNNNNNNNNNNNNNNNNNNNNNNNNNNNNNNNNNNNNNNNNNNNNNNNNNNNNNNNNNNNNNNNNNNNNNNNNNNNNNNNNNNNNNNNNNNNNNNNNNNNNNNNNNNNNNNNNNNNNNNNNNNNNNNNNNNNNNNNNNNNNNNNNNNNNNNNNNNNNNNNNNNNNNNNNNNNNNNNNNNNNNNNNNNNNNNNNNNNNNNNNNNNNNNNNNNNNNNNNNNNNNNNNNNNNNNNNNNNNNNNNNNNNNNNNNNNNNNNNNNNNNNNNNNNNNNNNNNNNNNNNNNNNNNNNNNNNNNNNNNNNNNNNNNNNNNNNNNNNNNNNNNNNNNNNNNNNNNNNNNNNNNNNNNNNNNNNNNNNNNNNNNNNNNNNNNNNNNNNNNNNNNNNNNNNNNNNNNNNNNNNNNNNNNNNNNNNNNNNNNNNNNNNNNNNNNNNNNNNNNNNNNNNNNNNNNNNNNNNNNNNNNNNNGGGTTGCAATAATGTGAGTGCATATGTGACATAGTAACTTGTGGAACTTAGCCATTTTTATGTTCAATGTATCTGGAAATATGTTGTCCAGAACAACCAAAACAGGGTCATTGTGTGTCTTTGGTTCAGTCTTGTGAATAACCTGTCAAGATANCCTAGATTTTGATCATATCCTTAATACCCAACCACCCCCACCCCCCACCCACCAACCACACACACANANACACANACACACAACACACACATACACACATACACACACNNNNNNNNNNNNNNNNNNNNNNNNNNNNNNNNNNNNNNNNNNNNNNNNNNNNNNNNNNNNNNNNNNNNNNNNNNNNNNNNNNNNNNNNNNNNNNNNNNNNNNNNNNNNNNNNNNNNNNNNNNNNNNNNNNNNNNNNNNNNNNNNNNNNNNNNNNNNNNNNNNNNNNNNNNNNNNNNNNNNNNNNNNNNNNNNNNNNNNNNNNNNNNNNNNNNNNNNNNNNNNNNNNNNNNNNNNNNNNNNNNNNNNNNNNNNNNNNNNNNNNNNNNNNNNNNNNNNNNNNNNNNNNNNNNNNNNNNNNNNNNNNNNNNNNNNNNNNNNNNNNNNNNNNNNNNNNNNNNNNNNNNNNNNNNNNNNNNNNNNNNNNNNNNNNNNNNNNNNNNNNNNNNNNNNNNNNNNNNNNNNNNNNNNNNNNNNNNNNNNNNNNNNNNNNNNNNNNNNNNNNNNNNNNNNNNNNNNNNNNNNNNNAAAACATGGGGAGCAGAACGGGTGGATTTGTCGCTGGAATTGTATTGCAGGTGAAAAGCTGACCATAATTAATATAACGAGTGAATAGTTGGTTTGCTTGTACTCTAAAATCAAAGTTTTTCATACTGGAAAAGTTTAAAAACAAATCTGAAGCCTTGGTGCACACAAAACATTATGGCACAGCACCGAAGTTGACAACATGAACAAGCTATCTGGAATTAATTTCTCCTTGATTTTAATAATTAATACAGCTGTGCTCTGCAGTTATACATAAATCAGTTAGTGTATTTACGTCATGTGCAACAAATATACCTCAGACTCCTTTGCCTANAGCANTGCAAGCATTATAAAGTATACATCTCTGCCATCTCTTATCACCAAGTNATGTCACATTGAANCAACCTTATATTACTAACCATTGAAGTGCTGGGGGTGAGGTGTGTGTGTGTGTGTGTGTGTGTGTGTGTGTGTTTATCTTTACCCATTAAATCTATCCAGATGGGCNAGGAGCTCCTTGGATGTGTCTTCCATTGATTCCCGGGCTCCCATAAAANCCTCCTTCACAACCTGTAATAANANAACACNGATGACACAGACCTCTTTCCCCAATGATGTGAAAACTACCTTCTCCAGTCGAAAGCATAGAAGAGGCTTGAGTAGGTCCCATGGTAACCTGGTACAAATCAAACATNTAGGAACAGGCTGGAGTTGAATCTGATTCGTGGGTTACCTGACTGTACTTGTTCTTGCTGTGTCCCTGAGCACCTCGTCAAGGCACGGGGGATATTCCCGACAGCCTTCACTAATAAACACTATACGAAATGTTGTCACGTGAGTTAAGTGGCACATGGGCTGTAATTAAAGTTTATTCGTTTGTTTACCCGTTCATCCCAAACAATCTAGACACAGACCGCCTAATCACTCCCCAGTGTACACGCNATTTAAAGCAGCGCTTACAACTATGTTACAAAAGAAATGTAGTAATAAAAACAATTCACCTTCTAGAATTCCCTTTAGCCTACTGTAGTCCATAGCTGCGCCTTCCTTTGGGCTTGGCTCCTCGCCGTTTGAGGTCGGTGGTTTCTTGGTCATCAAAGTGCGCGCGTTTTTCCCTTCTTAGCCCGTTAACATCATGTTTTCGAGCGACCTCACGTGACCAAACTCCGCCTATCGGTAGATAATTATCTTTGGGAGGTGCNTTTTCGCCTTTGCTTTTTAGTAATAAAAACTCTGTTCATGCGACGCATTGTGGAATACATACATTGTCAGGTCACTACATACANTGCCAAGTNACAGGCANNGAAGTACGTACACTTCATTATCTTCATAAAAGCANATTGTATTGATGAATTTGCTTAATGTACTTCAAATCAGTAGTTTAAATAATCATCATTGTTGGCATGACTTAGCACATGCATGTGTGTGCGTGCGTGCGTGTGTGTGTGTGTGTGTGTGTGTGCnTCAAGCCCCCCATCATCACCCTCCCACATACCCAGTCAAACCCCCCATCACCCTCCCATGGGTAACACATACCCAGCCATCACCCCAGGATACTGGTCACTACAACATGTTAACCTGTGGGCTTCTTGTGGTCACTTAGAGAGTAACCTGACAAAGGGCCAGCCAACAAGTGGTGGTGGGGAACCATAATAGCGTCCAAGACAAAATACACACACACACACACACACACACGTGTCAACAACCCAAACAACGGAATGAACAGTCAACACATTATCCAGGGCAGAGAGGAGGAACCTAGCAATGACAGACTGTGTGCCATGCCAAGAGGATGTGTCGCTCAATCAGCAAACTGGAGAACAATTGGTCCACCCTATCGTCATCATGTGGATTTGAGCTATTTACAATTGTCAAATATGAAAAAGCAAACATCCTTGCTGCCACTCCATCTTCATTACTACCAGCTCCACCCACATTCCCCTCCAACTGCTCCACCCACCTTCACCTCCCACTACTCCACCCTCCTCCCTACTCTCCGTTACCTTCTTTCACAAGCTCCCGAGGTATCGGAGGCATGACCACCTGATCAGCGTCGCTCTCCCCCAACTTCTCCACCTCTAGCTTTGGCTCGGTGTGTTCAAGGAGAGACTTGAGCACACCCAGCTCCGGCAAGACAGATGCGTCTCTGGCACGTTTGGCGCCTCCCTCTCCTTCCTCCTCCCTCCCTCCCTCCCCATCTCCCTCCGCTCCCAAGTGCCCCTGGTGCTCCGATATGTTCACAGTGGAGTCCGCCTCCTGCACCACTGTCTCCTCCCTCTCCATACTGTCCCTGAGGCTGCTCGACCTCTTCCTGNTCAAAAGCACTGTCCCATCCCGCAGATCAGGGGGCTGGTCCTCCTCCCCCTCTGCCGCTAGTAAAGTCTGGGTCATCGCTCTCCAGTGGTGTCAAGGATCTCAGCTGGGACCAGAAGGTAGGAGCTGNGCCAGGTTCCCCGAGGTCGTCAACCTTATCCCCTTCGGCATAGTCGTCTTCCGGGTGGGGCTCGGAGATGGGGCCGATCTGCTCCGTGTGCTTGGGTCTGTCCTCCAGAAGCTGTGCTAGTCTGACAGTTACAATACAGTGAATGTGCAGGACTTGTGTATGTGTGTGTGTATGTGTGCGTGTGTGTGTGTGTGTATGTGTGTNTGNNNNNNNNNNNNNNNNNNNNNNNNNNNNNNNNNNNNNNNNNNNNNNNNNNNNNNNNNNNNNNNNNNNNNNNNNNNNNNNNNNNNNNNNNNNNNNNNNNNNNNNNNNNNNNNNNNNNNNNNNNNNNNNNNNNNNNNNNNNNNNNNNNNNNNNNNNNNNNNNNNNNNNNNNNNNNNNNNNNNNNNNNNNNNNNNTNNACNGCATATTACTGTNNATTTTCATGNNAATGNGATTGCCTCGTGNTNTNACTACTNANATGGTTNAGAATTCAANAANNNGNNNNNTNCTNAAATGATGAAACTGTTGGGGATAAAACAATGCCTNATCACACCCCTANCATCCACAGGTAAAAATTAANNATTTTTGTTTCTTCAATTTNCTTATTTGGTCCCTGTGNGCAGTCCAATNGNCTTGANGAAAGATNNAANNNNNNNNNNNNNNNNNNNNNNNNNNNNNNNNNNNNNNNNNNNNNNNNNNNNNNNNNNNNNNNNNNNNNNNNNNNNNNNNNNNNNNNNNNNNNNNNNNNNNNNNNNNNNNNNNNNNNNNNNNNNNNNNNNNNNNNNNNNNNNNNNNNNNNNNNNNNNNNNNNNNNNNNNNNNNNNNNNNNNNNNNNNNNNNNNNNNNNNNNNNNNNNNNNNNNNNNNNNNNNNNNNNNNNNNNNNNNNNNNNNNNNNNNNNNNNNNNNNNNNNNNNNNNNNNNNNNNNNNNNNNNNNNNNNNNNNNNNNNNNNNNNNNNNNNNNNNNNNNNNNNNNNNNNNNNNNNNNNNNNNNNNNNNNNNNNNNNNNNNNNNNNNNNNNNNNNNNNNNNNNNNNNNNNNNNNNNNNNNNNNNNNNNNNNNNNNNNNNNNNNNNNNNNNNNNNNNNNNNNNNNNNNNNNNNNNNNNNNNNNNNNNNNNNNNNNNNNNNNNNNNNNNNNNNNNNNNNNNNNNNNNNNNNNNNNNNNNNNNNNNNNNNNNNNNNNNNNNNNNNNNNNNNNNNNNNNNNNNNNNNNNNNNNNNNNNNNNNNNNNNNNNNNNNNNNNNNNNNNNNNNNNNNNNNNNNNNNNNNNNNNNNNNNNNNNNNNNNNNNNNNNNNNNNNNNNNNNNNNNNNNNNNNNNNNNNNNNNNNNNNNNNNNNNNNNNNNNNNNNNNNNNNNNNNNNNNNNNNNNNNNNNNNNNNNNNNNNNNNNNNNNNNNNNNNNNNNNNNNNNNNNNNNNNNNNNNNNNNNNNNNNNNNNNNNNNNNNNNNNNNNNNNNNNNNNNNNNNNNNNNNNNNNNNNNNNNNNNNNNNNNNNNNNNNNNNNNNNNNNNNNNNNNNNNNNNNNNNNNNNNNNNNNNNNNNNNNNNNNNNNNNNNNNNNNNNNNNNNNNNNNNNNNNNNNNNNNNNNNNNNNNNNNNNNNNNNNNNNNNNNNNNNNNNNNNNNNNNNNNNNNNNNNNNNGTGTGTGTGTGTGTGTGTGTGTGTGTGTGTGTGTGTGTGTTGAGTGCGTGTGCGTGTCCTTGTACCTGTGTTGATGCATGTGCGTCTATGTGTGTATAGCTATGTGTGGGTTGTACACACTACCTTGGACTCTTCATCCACGTGAGGTCTGGTTCTACTGTGCTCACTACCAAAAGATTCTACAACACGATAGAAAAAAAGGTTGTAGTGTGTATTTGTGTGGGTATTGTATATGCACACATATGTGTGTGTGTGTGTGTGGTGTGTAATTGTGTGTNNNNNNNNNNNNNNNNNNNNNNNNNNNNNNNNNNNNNNNNNNNNNNNNNNNNNNNNNNNNNNNNNNNNNNNNNNNNNNNNNNNNNNNNNNNNNNNNNNNNNNNNNNNNNNNNNNNNNNNNNNNNNNNNNNNNNNNNNNNNNNNNNNNNNNNNNNNNNNNNNNNNNNNNNNNNNNNNNNNNNNNNNNNNNNNNNNNNNNNNNNNNNNNNNNNNNNNNNNNNNNNNNNNNNNNNNNNNNNNNNNNNNNNNNNNNNNNNNNNNNNNNNNNNNNNNNNNNNNNNNNNNNNNNNNNNNNNNNNNNNNNNNNNNNNNNNNNNNNNNNNNNNNNNNNNNNNNNNNNNNNNNNNNNNNNNNNNNNNNNNNNNNNNNNNNNNNNNNNNNNNNNNNNNNNNNNNNNNNNNNNNNNNNNNNNNNNNNNNNNNNNNNNNNNNNNNNNNNNNNNNNNNNNNNNNNNNNNNNNNNNNNNNNNNNNNNNNNNNNNNNNNNNNNNNNNNNNNNNNNNNNNNNNNNNNNNNNNNNNNNNNNNNNNNNNNNNNNNNNNNNNNNNNNNNNNNNNNNNNNNNNNNNNNNNNNNNNNNNNNNNNNNNNNNNNNNNNNNNNNNNNNNNNNNNNNNNNNNNNNNNNNNNNNNNNNNNNNNNNNNNNNNNNNNNNNNNNNNNNNNNNNNNNNNNNNNNNNNNNNNNNNNNNNNNNNNNNNNNNNNNNNNNNNNNNNNNNNNNNNNNNNNNNNNNNNNNNNNNNNNNNNNNNNNNNNNNNNNNNNNNNNNNNNNNNNNNNNNNNNNNNNNNNNNNNNNNNNNNNNNNNNNNNNNNNNNNNNNNNNNNNNNNNNNNNNNNNNNNNNNNNNNNNNNNNNNNNNNNNNNNNNNNNNNNNNNNNNNNNNNNNNNNNNNNNNNNNNNNNNNNNNNNNNNNNNNNNNNNNNNNNNNNNNNNNNNNNNNNNNNNNNNNNNNNNNNNNNNNNNNNNNNNNNNNNNNNNNNNNNNNNNNNNNNNNNNNNNNNNNNNNNNNNNNNNNNNNNNNNNNNNNNNNNNNNNNNNNNNNNNNNNNNNNNNNNNNNNNNNNNNNNNNNNNNNNNNNNNNNNNNNNNNNNNNNNNNNNNNNNNNNNNNNNNNNNNNNNNNNNNNNNNNNNNNNNNNNNNNNNNNNNNNNNNNNNNNNNNNNNNNNNNNNNNNNNNNNNNNNNNNNNNNNNNNNNNNNNNNNNNNNNNNNNNNNNNNNNNNNNNNNNNNNNNNNNNNNNNNNNNNNNNNNNNNNNNNNNNNNNNNNNNNNNNNNNNNNNNNNNNNNNNNNNNNNNNNNNNNNNNNNNNNNNNNNNNNNNNNNNNNNNNNNNNNNNNNNNNNNNNNNNNNNNNNNNNNNNNNNNNNNNNNNNNNNNNNNNNNNNNNNNNNNNNNNNNNNNNNNNNNNNNNNNNNNNNNNNNNNNNNNNNNNNNNNNNNNNNNNNNNNNNNNNNNNNNNNNNNNNNNNNNNNNNNNNNNNNNNNNNNNNNNNNNNNNNNNNNNNNNNNNNNNNNNNNNNNNNNNNNNNNNNNNNNNNNNNNNNNNNNNNNNNNNNNNNNNNNNNNNNNNNNNNNNNNNNNNNNNNNNNNNNNNNNNNNNNNNNNNNNNNNNNNNNNNNNNNNNNNNNNNNNNNNNNNNNNNNNNNNNNNNNNNNNNNNNNNNNNNNNNNNNNNNNNNNNNNNNNNNNNNNNNNNNNNNNNNNNNNNNNNNNNNNNNNNNNNNNNNNNNNNNNNNNNNNNNNNNNNNNNNNNNNNNNNNNNNNNNNNNNNNNNNNNNNNNNNNNNNNNNNNNNNNNNNNNNNNNNNNNNNNNNNNNNNNNNNNNNNNNNNNNNNNNNNNNNNNNNNNNNNNNNNNNNNNNNNNNNNNNNNNNNNNNNNNNNNNNNNNNNNNNNNNNNNNNNNNNNNNNNNNNNNNNNNNNNNNNNNNNNNNNNNNNNNNNNNNNNNNNNNNNNNNNNNNNNNNNNNNNNNNNNNNNNNNNNNNNNNNNNNNNNNNNNNNNNNNNNNNNNNNNNNNNNNNNNNNNNNNNNNNNNNNNNNNNNNNNNNNNNNNNNNNNNNNNNNNNNNNNNNNNNNNNNNNNNNNNNNNNNNNNNNNNNNNNNNNNNNNNNNNNNNNNNNNNNNNNNNNNNNNNNNNNNNNNNCCGCGGGTCGGGGGCCAGCACCACGTGGAAGGGAGCAAGGACAGCGGGCTCTCGAGCGGGAGCAACGACTCTGCACGCCTGGTCCACAGGCAGATGCAGAAGGTGGCAGACATGCAGCAGCAGCAGCAACAACAGAGGAGCCTAAAGTACAGCCAGCAAANGNNCNTGNNTCAGGATCTTCTCAGCAGGGCCACTCCTGCCAGGCACTCGTACAGGACAGAGAGGGAGATGATGAGAAATGGCGCNCTGCAGGAAGCGAGGTCGAACTCGCAGGATATGTTGGCCACCACAGAAGCTTCCCACAGACGCAACTGCAGGGTGGAGGGGAACTANGAACTGGAGGTACNTGCCCGTGGTGAAAGGGTTGGTTGAGGGTCTTGGGGTTGTCTAACATTGATGGATTCGTGTGTGGGTCTGAGTGTGTGTGTGTGTGTGTGTGTGTGTGTGTGTGTGTNNNNNNNNTGTGTGTGTGTGTGTGTGTGTGTGTGTGTGTGTGTGTGTGAATGTATTATGGAATACATAGCATATCAGTTGGTGGTAGTGGCGTCTGGAAGGCACACACATTCAAAGACTGCAGTGCAGTCCATGTATGTAGTAGTTGCTAACCACAACCATGCGCACAGCACACACAATTCAGACACATTAGATAAGATATTTGTACAATTCAATACAAGGTTGACTNNCCTGGCTGTCATCTGATTCCACTAGGAAATCTATATTCAGAAGAATGGAACCAACCAACTTGGGATCACGCTCGAGTATGGTGCTGGCCAACTGCTCGTCTGTGATGTAAGTAATGAGGGAGGGTAAGGGTGTGTGGCTCCTGTAATTAAGTGCAGGTGTTTTCTTGTGGGGGGTGCACATGTGTTCCTGTGGTGTGTGGTGCTAATCTGGTCTTGCTTTTCCCACNNNNNNAGGTGGTTCCAGGTGGACTAGCAGCCATGGATGGAAGGATCAAGAAAGGGGACATCATACGAATGGTGAGAAGCNACACCAAACCACACCAGGACCAGCCTTTCAATACCTCCCTCCCTTGTCGTCCCTTTAACCAAATCAGTGGTGATTTGTCAGTCCACTGTAGGCTACTGAATTGTGGTTGCTGTCACATCCTTCTTTACATCCTGGTTCCCACATAGTCCCTGATCTAGTAGTGGGTGAAAAGGGAGTGGGGGAGTTGCCCATTGATTCNGCCCTGTCNTATCCACTCCCTTGGGGTTGCCATGTTGTATGTATTGTCTCACCATCAGCAACTCCCAATGAACAAGACATGCCATGGTATCCATGAGAGACGGTTATGTTATCCACTGTATTGTTGATAAATACATGATGTTAGGTGGTAGCTGTGGCTTTAGTGGGTGATTGTGCACTAACAAGCTCAGTAAATGGGTCTGTTTCACTCAACCTTGTAAAGTCTAAAAAGCGCTAGCATTCCTTGTGGCTTAACAAGGGATTGGTACACAGGAGACAGCAGCTATGCTCCATTTAGNCACNTCNNNNNNNTGTGNGCTNCGNATGTNNTTNTNTTNTNTTTNNNNNNNNNNNAGGTGCACATCACAACACGAGTCATAATTGCATTTCACACATTNCTAGACATGGGGCTTAGCTGTCAGCTTAATGAATATATGGCAGTTCACATNCTTTGTTCTTCAGTTGGCTGAGGGGTTTCCATACCACAGTATCNTGTAGAGAATGCACACACATCCCTATACTAGCTGTTCAATCCTTGGCCCCAGACCTTCAAAATATCCCCTCTGTGCAAACATTAGTATTGCCTTACTCGTCTTTAGGTGAATGGATTCTCAGTGGTAGATCCAGAAGAGGCTGTCCTCTTGTTTTTGGAGCAGGACCAAGACATCACACTCACAGTGGCAAGAAAGATGCAGGTAAGGGAGCAGACTGCCAAGAAGGCATATAGGGTGTGTGGGTGGGGGATCATGTGATGCCATTAATGTTATGGGCAGTGAGATCACATGATGCAATTAATGCTCTTGGTGTCTCTCTCTCTCTTGGTGTTTATCTCTCTCTTGGTGTCTATCTCTCTCTTGGTGTCTCTCTCTCTCTTGGTGTTTATCTCTTCTTTCTCTCTGTTTATCTCTTCTTTCTCTCATGTGTCTCTTGTAGCACCTGGCCATGTATGATGACAGAAGGAACTTCAGAAGNATCGACAGTTGTGGTTCCCTTGACTCCCACACCAGNGAATCAAGGCAGTACGTGTCGAGGAAACCCAGCAACAACAGTGAAGACGGCTACGTCCATATGAATGGCAGAGACGACCTAGCTGCTGNCCTACCTGAAAACCGTGTACGACTACTGCCCACGAGAAGGCCACAGGACCCCTTACCCCCTGCACGGAGAACATTCGATGACCCCCAACTCCCCACGAGGAGGGCATTTGANGACCCCCTCCCTCCTACNAGAAGGGACCCCCTTGCTCCCTTGTGGAGGGCACCNGATGAAACCACTCCNCNCCCAGGTCTCCAGCAACACCATCACCCCAGTGCTTTCTTCAGACCCATAGCAGACGACCATCGCCNNAGGGACCGCAGAACACCCCCTCATCTACTCACAGCAACAGTCCCGCCCTATGACGACCACTCCTCCNATGAAGCACAAGACGTCCGTGCAGCTGTCTGCCCCGCCTTCTGCTGTGCCCAATGGACACATATATGGCGAGTTGGAACCACCTCACTATGGTTACATGAGGACTGAGAGTCTAGTCTAACCCTGTCCTGTGTGTGTGTACATGTGTGTTGTGTACATGTGTGTGTGTATATTTGGTGAAGTATCATACTGTTACTCTGTGTTGTGACACAACCAATCCTGGNCACAAATCACACCCAACTAGTTATGAAAACATCCTTCAAGTGATTACATTTAAAAGAAGATAAAAACAAGACTAAGCTGAGCTAGNGGCCTCTCCCTTGTTTNGANCCCCACAAACTGGTTCATGGTGGGTACTGTACACTTTNGTTTGATGAATAAACAGTGAAAATGAGTCAAATGTAAGTACAAAGGAGGAGGAGCTAACAGAATCTATCAGTTTGTATCCATGCTGCCGCTNTCAAACACTTCTGCAAGCTTTACATCTGCGTCTGTGAAGGGAGGGAGAACAGCTTAAGGTATCNAGAAGTGGATGTACATGTGGGGGTGGGTAGGAAAAGGATGTAGGGGGAGGCCACACAGCCCAGTACTACGCCAAATCTGGCGTAACGGGTGGTGTCAAATTGCACGATTTGGTTACGGATAATCGATTGGGCTATGTGGCAAGGAATGCAACTTGGCTTGTCTCCTAAGACCACTTGATTACTGTCATTTCTATCAAGCCTATCTACGTGTGCACATGTGTGTGCACGNATGATTATTCAATCGCAATCTTCAGTTNCAACAAAGTGCCGGGACTCACAAGGGCAGGCCAAAAAGAGGAATGCGTGAAACCTACAGGAGACCAATAGGTCATTAGAGCTGGCTTTAACCTCAAGGGGAGACCTACAGGAGACCAATAGGTCATTAGAGCTGGCTTTAACCTCAAGGGGAGTGCAAATTAGACATCTCACACATCCTAATGGGTTCAGGTGTAACTAATATGNCCATACCACTGCAGCCCAGCAGGAGCAAACATACACACATTCCTGCCAGTCTTAGCCTTCCTCTCCTAACCACACCAGACTACTGAGCCCCCCTAGTGTCTCCATAGGTAACACATACCCAGTCAAGCCCCTCCCACATACCCAGTCAAGCCCCCCATCANCCTCCCACATGCCCAGTNNNNNNNNNNNNNNNNNNNNNNNNNNNNNNNNNNNNNNNNNNNNNNNNNNNNNNNNNNNNNNNNNNNNNNNNNNNNNNNNNNNNNNNNNNNNNNNNNNNNNNNNNNNNNNNNNNNNNNNNNNNNNNNNNNNNNNNNNNNNNNNNNNNNNNNNNNNNNNNNNNNNNNNNNNNNNNNNNNNNNNNNNNNNNNNNNNNNNNNNNNNNNNNNNNNNNNNNNNNNNNNNNNNNNNNNNNNNNNNNNNNNNNNNNNNNNNNNNNNNNNNNNNNNNNNNNNNNNNNNNNNNNNNNNNNNNNNNNNNNNNNNNNNNNNNNNNNNNNNNNNNNNNNNNNNNNNNNNNNNNNNNNNNNNNNNNNNNNNNNNNNNNNNNNNNNNNNNNNNNNNNNNNNNNNNNNNNNNNNNNNNNNNNNNNNNNNNNNNNNNNNNNNCTCCCACCTGCCCTCCATCACCTCCTCCCACCTGCCCTCTATCAATCTCCCACCTTCTCCCCTGCCCTCCATCACCCTTTCTACCATCTCAACAGGTGCTCTCACCTGATCACTTTGATTGCTGACTGTGCTGTCCTTCCTGCAAGTCCTGCTGACCTTTGGGTTAGGGGAATGCCTTGGCTGCGCTAGAGGAAACGAGCTGAAGCTGGTGTCCACAGAACTGATGTCACCAGCCCCCAGCGTGTCTTCAGCCACCAAGCAATTCAGGTCTGACCCCTCATCAGAGGCACCCCGCAAGTGTGACCCCTCTTTGGAGATGGTCTGCAGGTGTGGTGGGCCCTCCTGGCTGCTGACATCAACCAGCAGCTGGGAGCAAGGATTCTCCTGGCTGCTCAGCCCTAGGCCACTGTCCATCTGGGCCACCGCCTCCCCCTTCTCTGCAAGTGTCTGGCAAATCTCAGCTGCTTCGTGTCTCACTCCTCGTGGAGTCAGTGACCCAAATGGTCTGTCCGATTGTAGTTGAGAACCCTCAGTGATATCTGAAGCAATTCTCTCCAAGTTACTCTTCCCCACACTGATATTTTCAAGCTCCAATGGAACAATACGTTCATCTGTCTCTCTTTGAAGTGCACTTGTGGAAGGACCGGGGACCAGGTTCCCCAGCGTGCTATCAGAGCTCCCGAGAGACAATGACTGCTTCAAGGTCGGAGGATCTCCGCCTTCATCGTCAGAGTCAATGCGTTGTTTCCTTGCAGTGCTATTTCTCTTGACAAATGGAGTGATGTCATCATCATCGTCACTACTCACATCAGCATCATCACTTGGATCACCCTCTCCATCACTGGGGTCACCTCTATCGCCCTCCTCATCACTAGGGTCACCTCTATCACCATGGTCACCCTCTCCATCACTGAGTAAACCTTTCCCGTCGTGGGGACCGTCCTCTTTGCCGCTAGTTTCATTGTCTCTCCTATCCCTCTTCTGATCACCATCCTTCTCTTCCCCCTCCTCCTCACCAGGCCTCTCTTCCTTCTCCTTAGTGCCAGTACCATGGACTCCCTCACCATCCGATAACGACTCTACATCTTTCCAAAGTTAATGTAAAAAAATAACAACCAAACAAGTTAACAACATCTCACACCCAAGCATTTCCAGGCCAAACCTGTGGTGTCCTGGTCGGTCAGTTCGGCCTCCTCCCCCTCTCCTTCCACATCCCCGGGACCCTCCTCTCCATACAGTTTCCTCTCTTCTTCAGCTATCTTCCTCTCCTCCGCTCTCCGCTGAGACAGCTGCCTCTTGAGAGTGGCGTGCAGGTGCTGCCGCAAGGCACCTGATCGGTTCATTCTTATTAATGCACCCACAGCATGATATCACCATTGAGCTACCCATCCCTTGCCCCGCCCACTGCTCCACCTACCCGGCTTATTCTTCAGTTGCACTACAAGGTCAGGTGGAATCAGCGATTGTGACTCAATGGCCAAAGGTGGAACCAGCGTGCTTGCTTGTGGTGGTGGTGGCTTCTTCGTCTGCACCAACTGGGCAGTGAACCTTTGCTTCAACCGTTCCAGCTCTGTCAACTCCTTGGTTTCCGTGACAATGGGCACAGAGAAATCAGCTATTGCAAACGAACTAAGCTTTGGTTCAGGAAGAGATGAAACCACTGGTTGGGTAACAGTACCCTCTGGTTCATCAAGGTGCGGTTCCGGGTTCCCTGTGTTCTCCTTATCAGGTGACATTACAGCTTCACTCACACCATCTTGTGTGACCGCATGGATAGCTTTAGTGCCTTTTTCCTCCGCGTATTCCAGTGCTGAACTGCCATCAAAGGAAGACGATTTCATGACTTTCACTATGTTCTGCGTACATGGCTGCAGAGTCTTGTTGACCTTCATGAGAAAGCTATCAATTGTTCTCATCCTGGGCTGGTGCTTAGGTATGGACACTTTAGACTCTGGAGAAGATAAACAAAGGTGTTTTAACTACATAGACACACACAAAAATAAGTTGTGTGTCTGCCACTCCAGTCAAGTGCTTTTGGCGCATTACGCTTGACAACCCTCCCATGACAGCAGGTGACTGTGTGGAGGTGTGATACAGTAGGTAGTCCAAACACTATAGAATCTGATTTCAGTCATTAATATTTGAGGTGACCAAACACTCAAACATGTTTTTCAGACCACACAAGTTGTTAACAGACACTGCCCAAACAAGCCATTCATTTTATTTTGTACGTCTTGTTGAAGACAACAAATAAAGACAAGCAGCAGTCCAGCAGCTCATATTTTTAAAAGCATACTACGCTCGATTCCAGTGCTGACTCTGGAAGTTGTACTACAACCAGCTGTGCAATGGTTGGCACACAAGACAACAATAGTTCCATCCATCAGGGGAAACTCCCGTGGTGTGTCAACTGGTTGCCATATGATGAGCATGTGATTGACATTGTCACATGATGAGCATGTGATCAACATCAGGGTAGAGGAGGGATGATCCCCACCATATGTGACCTTGTCACATGATGAGCATGTGATCAACATCAGGGTAGAGGAGGGNTGATCCCCACCAGTGGTCATATATGTACTCACCTCGTAGCATCCTTTGACTTTCGCTGTGCACTTCGAACAGTTCGGACTTCTTTAGCTTCTGGCGCTGTAGTCAGCGAATAATAATGGACTATCAAGTTAACTAGCTGAACGCGTACCGTCCTTTGAGCTCTACTCTCCGCTTTTTTCCTTGACGTTGAGGCGGGCACATCCGCTTGCTCTAACCCCGAAACTTCTTCCTGAGGTGCTACGCGAACGGGTTGATTTTCGGACGTTGTTGTCTCGCAGTCGCTTCCATCACTTCCCGTGCCAAAGATGTCTGAATTCTGAAACAACTACACCAGGCATGCGTGGCGTGCCGTAACTTGGGTGAAATTTGAGAAGCAGGTACATCATGAAATAATACGGGACTACTGTTTTAGATACCTCTCCCGACACCTCGTTATTTATAAAATACTTACTGCCATAGCTCACACATTACCCGCCTTCTCGTAAATGGCGCCAGCGGACTCATTACGCATGCGTGCAACTCTACGTGCGACAATGGTAAATGAATTAAGTTTGGTATGCAAAAGCGGGTGCATGCCAATCTCGCAACAAACTCTATGTATAAGGCTCCCGCAACAATATCCGCGTTATTGACGTAGCTTAGTATGAGTGGCTACCCACTAGACTCGTTTGTCGATCCCATTGACGAAGCATTGCTGTGTCGAATCTGCCGAAGGGTCCTGAATTCGCCCCGTAAGACACCGTGCGGTCATGTCTTTTGTCTGTCTTGCTTGAGCTCTTGGATTGAGTATTACGGCNTNNGTNNNAAACCGNNGCGGCGATGTGGAGTTGATAGAACTAACCCACCCTTTCGACATTGAAGAGCGTGTTTTCGGTTTGCTGAAACGATGCAAATATTTTAGCATTGGGTGCAGAAAGCAAGGCAGGCTGGCCGATATTGAGCAGCACGAGAAGGTCTGCACGTACCAACACTGCGACAACAGGCAACAGCAACNANCACANNTNCACNACNNATCCGGAGGAGATGAAGAAGACCATTGCACCGTCGCTGGACTGCCTCTTTGGCCGCTTCGGCAAGGCAAAGAAACGCCCTTCGACGAATGCTAAGCCTAGCGACATACAAACGGTACTTCTACGGCGACGTAGTCATATTACACACGTAATTAGAATAATTAAATTGACCCCGCAGCTAACTCCCCCGATATAAATAGTGTTTAAATAACTTTTATCCAGTTTTTTCTTTCTGGCGTGTGTGCACAGNGAAGACTGATGCAAACTGAGCTGACGTTTAGGTTGAAGCGAAACAAGACGACGGAGAATCTTGGCCTTGTTCTCGTATCNGGATCCAAGGTAATGTGGACGTGGGACGAAAAATGAAAACGCGCTAATGGCTTAAGTGGTAGCGCTAACCTCTGCCAATACTTTTCGCATAGCACGCGCCGGAACTAACAGCGTGGGGAGGGGCAAGAGGGAAGGAGTTTCAAATAGNGGTTTCTCCCCTCCTAGAACTCCGATGGTAATGCCGAATACTTTCTCGTCAAAGAAGTCCACGCAGGTACTCTGGCCTCCAAGGCGGGAGTCTTTGCCGGCGATCGGATCGTGCAGGTATGAACCTTTACGTGTGCATGCAGACACGCACAATCAGTAATCAGCGGAATGAGGCGTGCGAACTGCCTCAGCTACTTTCACAGCCACCTCAACGTACCTGCAGGTCGATGGCGTTGTGTTGGGTCGAATGAACCCGGGAGAGGCGGTAGCGCGCATCAAGGACAAGACCCTGGTGACCGTGACGGTCGTGAGAGGCGAGAAACCTACGTCCCACTCCCACAAGGAACACATATATGACGAAATTCTGTACTCAGACGCGAGCCAATTCTCGCTTTCGCAACCCGAGGAAATGTTGCGTCNTNCATGNNCNTGGAGNNNNNNGAGNNACATCCCCCACGGCATAGCCCGCAGGGCCCGAGGCGCGCCCGAGNNNNNNNNNNNNNNNNNNNNNNNNNNNNNNNNNNNNNNNNNNNNNNNNNNNNNNNNNNNNNNNNNNNNNNNNNNNNNNNNNNNNNNNNNNNNNNNNNNNNNNNNNNNNNNNNNNNNNNNNNNNNNNNNNNNNNNNNNNNNNNNNNNNNNNNNNNNNNNNNNNNNNNNNNNNNNNNNNNNNNNNNNNNNNNNNNNNNNNNNNNNNNNNNNNNNNNNNNNNNNNNNNNNNNNNNNNNNNNNNNNNNNNNNNNNNNNNNNNNNNNNNNNNNNNNNNNNNNNNNNNNNNNNNNNNNNNNNNNNNNNNNNNNNNNNNNNNNNNNNNNNNNNNNNNNNNNNNNNNNNNNNNNNNNNNNNNNNNNNNNNNNNNNNNNNNNNNNNNNNNNNNNNNNNNNNNNNNNNNNNNNNNNNNNNNNNNNNNNNNNNNNNNNNNNNNNNNNNNNNNNNNNNNNNNNNNNNNNNNNNNNNNNNNNNNNNNNNNNNNNNNNNNNNNNNNNNNNNNNNNNNNNNNNNNNNNNNNNNNNNNNNNNNNNNNNNNNNNNNNNNNNNNNNNNNNNNNNNNNNNNNNNNNNNNNNNNNNNNNNNNNNNNNNNNNNNNNNNNNNNNNNNNNNNNNNNNNNNNNNNNNNNNNNNNNNNNNNNNNNNNNNNNNNNNNNNNNNNNNNNNNNNNNNNNNNNNNNNNNNNNNNNNNNNNNNNNNNNNNNNNNNNNNNNNNNNNNNNNNNNNNNNNNNNNNNNNNNNNNNNNNNNNNNNNNNNNNNNNNNNNNNNNNNNNNNNNNNNNNNNNNNNNNNNNNNNNNNNNNNNNNNNNNNNNNNNNNNNNNNNNNNNNNNNNNNNNNNNNNNNNNNNNNNNNNNNNNNNNNNNNNNNNNNNNNNNNNNNNNNNNNNNNNNNNNNNNNNNNNNNNNNNNNNNNNNNNNNNNNNNNNNNNNNNNNNNNNNNNNNNNNNNNNNNNNNNNNNNNNNNNNNNNNNNNNNNNNNNNNNNNNNNNNNNNNNNNNNNNNNNNNNNNNNNNNNNNNNNNNNNNNNNNNNNNNNNNNNNNNNNNNNNNNNNNNNNNNNNNNNNNNNNNNNNNNNNNNNNNNNNNNNNNNNNNNNNNNNNNNNNNNNNNNNNNNNNNNNNNNNNNNNNNNNNNNNNNNNNNNNNNNNNNNNNNNNNNNNNNNNNNNNNNNNNNNNNNNNNNNNNNNNNNNNNNNNNNNNNNNNNNNNNNNNNNNNNNNNNNNNNNNNNNNNNNNNNNNNNNNNNNNNNNNNNNNNNNNNNNNNNNNNNNNNNNNNNNNNNNNNNNNNNNNNNNNNNNNNNNNNNNNNNNNNNNNNNNNNNNNNNNNNNNNNNNNNNNNNNNNNNNNNNNNNNNNNNNNNNNNNNNNNNNNNNNNNNNNNNNNNNNNNNNNNNNNNNNNNNNNNNNNNNNNNNNNNNNNNNNNNNNNNNNNNNNNNNNNNNNNNNNNNNNNNNNNNNNNNNNNNNNNNNNNNNNNNNNNNNNNNNNNNNNNNNNNNNNNNNNNNNNNNNNNNNNNNNNNNNNNNNNNNNNNNNNNNNNNNNNNNNNNNNNNNNNNNNNNNNNNNNNNNNNNNNNNNNNNNNNNNNNNNNNNNNNNNNNNNNNNNNNNNNNNNNNNNNNNNNNNNNNNNNNNNNNNNNNNNNNNNNNNNNNNNNNNNNNNNNNNNNNNNNNNNNNNNNNNNNNNNNNNNNNNNNNNNNNNNNNNNNNNNNNNNNNNNNNNNNNNNNNNNNNNNNNNNNNNNNNNNNNNNNNNNNNNNNNNNNNNNNNNNNNNNNNNNNNNNNNNNNNNNNNNNNNNNNNNNNNNNNNNNNNNCACACACACACACACACACACGCACACACACACAAACCATAACAGGAGAGATGGGTTACAGAAAGGTTGCCCACCTAGTGTGTTTTCTTCATCAGGTACTGGAGGTAGCTTTGCCTNATGNCTCNGCCCCCTCACACCCACACTCCCTCTGACACTGTGTCATAATCTTTGTTTGTAGGAGTGTATGTGCAGTCTGAGGGTGTGGTGTCTGTGTGTAATTGTTAGTTACTGCTATTCTGATTATGTGATTGGCATAGTGTGCAGCTCATGTTTCATATATGTGTCAACTTGTCACTGACAAGTAAACACAGCTGTTTGTGTTCATTGAAATCATGGAAATAGTTTGCATTAATGACAGTACAGTTTCGTGAACAATGAGTACTCTTAGGTTTGAGCTATTCATTGCTTGTTGTCATTTGATACTGTATGAGGGATGATTTTGCAGTGAGTAAAGTTACTAGCAATCTTGTAGAAACAAGAGTTCTGTAACATGTCATCATGTTGGATGATCATATTTGATGGTTACTTAATGCAGTCCTAAGATGTGTGGAAGAAGCTCCCACATATATGCACGTTGACTGAACTCAAGAAAGATATCCACATTCTGCTGTAGCTAGACACAACACCACTCCCAGAGTGAAAGAGCAGGTGGGCAAATGGATCCAATGAATGAGGTGTGACATCCACATCCCACACACACACACACACACACACACACACACACCATACCACTACACAAACAACAATACCAACACACCACACTAAAAACACTACACTACAACACACACATTACACAACATTACTACAATACAACATACATGTACCACCCCACACCACCCCACACCTGCACATACCACTCAACACAGACACCACATTACCTGGCAGTGGGCTTTGGTTTTGGGAGTGAGGCTCTCCTTTTCAACAGAAGTGGCATGTCCTCATCCTCACTCTCCTCCTCTCCATCCTGCTTCTGGGGTGCTTGGCTATGTCTCAGTGCCCATCGCTCCAGGACACCCTTCCCACCCTCCTCCTCCTCCCCACCATCTCCCTGGGTGGCATATTGACTAGGAGAACCACTGGGGAAAACCCCTGAACACAAAGCCAGTATATCCGTCCCTTGAGTCCCCTCCTCCAGAGTGNNNNNNGCCATGGCAACAGAGCTGAGCAATCCACTCACTGGCTTCTTATGGATGAGAGGGAACCTGACCACACCCCTTGGTGCATCCTGTGTGGCAAAGGAACCGGAACACAGGTCTAGGAGTTCGTCAGATGGCTCCTCTAAAGCAGGGGTAGCCACTGTCTTGACCAAGGAACGAGTTGATGACCTCTTGTCCACACACAAATATCTGCAATGGAGCAAGTAGTGGACAAGTAAGTACAGTGCACCCTTCTCTCTCCCCTGNGGGTAAGTACAGTGNACCCTTCTCTCTCCCCTNGGGTAAGTACAGTGCACCCTTCTCTCTCCCCTAGGGGTAAGTACAGTGNACCCTTCTCTCTCCCCTGNGGGTAAGTACAGTGCACCCTTNTCTCTCCCCTGGGGTAATACCCTTCTCTCTCCCCTAGGGTAAGTAAAGTGCATCATTCAGGTAAGTACCATTCATCCCCTCTCCCTCCTCTCCCTTCCGCACATCTCAGAATCAGCAGGTGGGACCTACCCTTGGGCATCAAGGAACTGTGTCTGAGAGTCTTCAGCACAAGACATTTCTTTAGACCAGGCCTGGGCATTGCTGTCAGTGTCAAATGGGTCCTGTGAGGGAGTGGAAGGTNTTAATGCAGTCAAGCCCCCAAACCTTATCCATAACAGAATTACTTGTGAGCCGTCTGTAGTTTGAGCTTCAAACAATTTCTAAGTTGAGACACAAAAAGTAAAACAAGTAAGCCACATTCAAACATGACAGATTACAACTCCTTCCCTTATCTAAGCCTTCATCTCCACACGTTTGTGTTGTCAATATGAAAAGTACATTGAAACTATTGACAATGATTGCTTTGAAGTGTGGAAGTGGAAGCGCCTACATGGCATGGCTACACATAGGGTACACGGAAGGGTGTGGCTACACATAGGGTACACAGTCTTGCATTGTGCATCATGCTGTGAGGGGNGCGGGGCCTGACGGCAAGTTGGCACAACTTGGTACAGAACTATATGCTTGTAATAAACACACTGCCCCTGAAAAACTACAAGGAAGCCTGAATAAAGCTTATAAATCGTATTTTAACAGCAAGTGTTAGTAAGACATGCACAATCGGGTGTGACCAAAAATGAATTTCAGGGAGCATTCATGCAACAGTAATGTTGTCATATAAGGTTTTAGTATGACATGTAAATTAATTCCCAGCGACAGCTCCAAAAATGTTACTAATAAACTACCAAAAGATGCCGCTATTCCTTTGATAGCATTAGCATGGGCTCATCAGTAGTTTGTATATACAAATGCAGCGATGGAATTTATCCTTTAAACAAGTGATATGCTTTTACAAGATCACAATCACACCAGCTCATGTGCCACACCCCATCACTCCTGAACGTAACTCACCCTAATCCACACCCATTACTGCTTATATCTGGCCATCCCAAACATGCTGACTCCACCCATATCGCGCCCACCAATTATCTCATAATCACTAATCTGCCTTCCATCCTCTCACCTNCACCTGCCCTCCATCACCTCCTCCCACCTGCCCTCCATCNCTCTCCCACCTNCCCTCCATCACCTCCTCCCACCTGCCCTCCATCAATCTCCCACCTTCTCCCCTGCCCTCCATCACCCTTTCTACCATCTCAACAGGTGCTCTCACCTGATCACTTTGATTGCTGACTGTGCTGTCCTTCCTGCAAGTCCTGCTGACCTTTGGGTTAGGGGAATGCCTTGGCTGCGCTNGAGGAAACGAGCTGAAGCTGGTGTCCACAGAACTGATGTCACCAGCCCCCAGCGTGTCTTCAGCCACCAAGCAATTCAGGTCTGACCCCTCATCAGAGGCACCCCGCAAGTGTGACCCCTCTTTGGAGATGGTCTGCAGGTGTGGTGGGCCCTCCTGGCTGCTGACATCAACCAGCAGCTGGGAGCAAGGATTCTCCTGGCTGCTCAGCCCTAGGCCACTGTCCATCTGGGCCACCGCCTCCCCCTTCTCTGCAAGTGTCTGGCAAATCTCAGCTGCTTCGTGTCTCACTCCTCGTGGAGTCAGTGACCCAAATGGTCTGTCCGATTGTAGTTGAGAACCCTCAGTGATATCTGAAGCAATTCTCTCCAAGTTACTCTTCCCCACACTGATATTTTCAAGCTCCAATGGAACAATACGTTCATCTGTCTCTCTTTGAAGTGCACTTGTGGAAGGACCGGGGACCAGGTTCCCCAGCGTGCTATCAGAGCTCCCGAGAGACAATGACTGCTTCAAGGTCGGAGGATCTCCGCCTTCATCGTCAGAGTCAATGCGTTGTTTCCTTGCAGTGCTATTTCTCTTGACAAATGGAGTGATGTCATCATCATCGTCACTACTCACATCAGCATCATCACTTGGATCACCCTCTCCATCACTGGGGTCACCTCTATCGCCCTCCTCATCACTAGGGTCACCTCTATCACCATGGTCACCCTCTCCATCACTGAGTAAACCTTTCCCGTCGTGGGGACCGTCCTCTTTGCCGCTAGTTTCATTGTCTCTCCTATCCCTCTTCTGATCACCATCCTTCTCTTCCCCCTCCTCCTCACCAGGCCTCTCTTCCTTCTCCTTAGTGCCAGTACCATGGACTCCCTCACCATCCGATAACGACTCTACATCTTTCCAAAGTTAATGTAAAAAAATAACAACCAAACAAGTTAACAACATCTCACACCCAAGCATTTCCAGGCCAAACCTGTGGTGTCCTGGTCGGTCAGTTCGGCCTCCTCCCCCTCTCCTTCCACATCCCCGGGACCCTCCTCTCCATACAGTTTCCTCTCTTCTTCAGCTATCTTCCTCTCCTCCGCTCTCCGCTGAGACAGCTGCCTCTTGAGAGTGGCGTGCAGGTGCTGCCGCAAGGCACCTGATCGGTTCATTCTTATTAATGCACCCACAGCATGATATCACCATTGAGCTACCCATCCCTTGCCCCGCCCACTGCTCCACCTACCCGGCTTATTCTTCAGTTGCACTACAAGGTCAGGTGGAATCAGCGATTGTGACTCAATGGCCAAAGGTGGAACCAGCGTGCTTGCTTGTGGTGGTGGTGGCTTCTTCGTCTGCACCAACTGGGCAGTGAACCTTTGCTTCAACCGTTCCAGCTCTGTCAACTCCTTGGTTTCCGTGACAATGGGCACAGAGAAATCAGCTATTGCAAANGAACTAAGCTTTGGTTCAGGAAGAGATGAAACCACTGGTTGGGTAACAGTACCCTCTGGTTCATCAAGGTGCGGTTCCGGGTTCCCTGTGTTCTCCTTATCAGGTGACATTACAGCTTCACTCACACCATCTTGTGTGACCGCATGGATAGCTTTAGTGCCTTTTTCCTCCNCGTATTCCAGTGCTGAACTGCCATCAAAGGAAGACGATTTCATGACTTTCACTATGTTCTGCGTACATGGCTGCAGAGTCTTGTTGACCTTCATGAGAAAGCTATCAATTGTTCTCATCCTGGGCTGGTGCTTAGGTATGGACACTTTAGACTCTGGAGAAGATAAACAAAGGTGTTTTAACTACATAGACACACACAAAAATAAGTTGTGTGTCTGCCACTCCAGTCAAGTGCTTTTGGCGCATTACGCTTGACAACCCTCCCATGACAGCAGGTGACTGTGTGGAGGTGTGATACAGTAGGTAGTCCAAACACTATAGAATCTGATTTCAGTCATTAATATTTGAGGTGACCAAACACTCAAACATGTTTTTCAGACCACACAAGTTGTTAACAGACACTGCCCAAACAAGCCATTCATTTTATTTTGTACGTCTTGTTGAAGACAACAAATAAAGACAAGCAGCAGTCCAGCAGCTCATATTTCTAAAAGCATACTACGCTCAATTCCAGTGCTGACTCTGGACAACCAGCTGTGCAATGGTTGGCACACAAGACAACAAAGGTTCCACCCATCAGGGGCAACTCCCGTGGAGTGTCAACTGGTTACCATATGTGACCATGTGGCTGACATTGTCACATGATGAGCATGTGATCAACATCAGGGTAGAGGAGGGATGATCCCCACCATATGTGACCATGTGGCTGACATTGTCACATGATGA>scaffold6592|size4731ATGCTAATACAAATCTAATCAAGCCCGCATATTATAATCAGACCGATTTTTAGTGCGTAGTAAACCTGTTTACAATTCAATTAAAAATAAAAGCAAGATGACCAGTCTTAGCTATGTATGGGCCATTTCTGGATATGAACAACTGTTTTCCAGAACCTGTGGCATATCAAGGGACCAACTACAACAACAACAAAATAACTACTCGAAGATGATATATTTATTGATATTTATCATCACAGGACCATTCTATAAGTCGGCAAAACGACTTACAGAACGGTTCTGTGATGAAAAATATGAATAAAAGTATCTCGAGTACAACAGACAGTTCTGTCTGTTGTACTCGAGGTGCCNTAGGTTNTTTCTTGATAGCACAACTATCTCACAACTAACGGGTATCGTATTAAATGCCGCATTACCATCCAATAGTTGGAGGCCTTAAAACTGNAACCATCGATTAGTATACAANTCATTTGTAATAAATAAACTTACGTTACTAGCAACCTAAAAATTCATACCATGTAGTAGAAGAAACAGTTCCACAAGGGATATCGGTCACGACTCGTCCCTTTTTTTTTTTCAGACGCAATAAACGACGTGCGCATGAACTCCTGACAGCAAACGTTTTAAGTGATTATAAGGCATATATTCATAATTCAGCACTAACTCACCAAGGAACAATCACAGCACTAGCTACTATAGTGTAAAGCTTCCCCCGACACTTTTTACGAAGCGTGATTCTACATTGCGGATCGTCAGCGTGGAATGCTACGGCTCACGTGATTTGTTTCCAACCCTACAATAAAGGTATGGGGGGAGGCAAAAATGTTAAACGGTGTGTCTTGATCACTGCAAGAATTTCAAAGGAGACTCATTTTCCTACGACTGACTGGCGTTTGATTTGTAGCGTAGGGCACATGTACACTGGATAGACATCAAATTTGGATTTCGTAGAGTTATAGATCCTTTTCATGTGTCATGTGACCGCTCTGTAATCTAACATACGTCAGTGTCACACAATATAAATTAATACACGTCCGCTATGTTGGTGCANANCNTTTNCGGANNTNTCCGAGTGACGAAAAGTACTATCGATATGCTTGCGTCACTTACTATTTGATTTCTTAGAAAGCATTTAGTTCGCACCTCTCTGTAAAGGAACAATCAAAATTGTACACGTCACGAGTCGTGTTGAGTTTAGGGCCTCTGTTTGAGGCATGGACCTCTTCTGGCGTTGATGGTATGTCTGTAATTAAATTTAATATGTAAGAGAATGCAAAGATATGAAACATTCCTCGGAGCGCGCGTGACACGACGTGATCGCTGCAGTGTGAGTCGGTAAACAGAAAAATGCTGTGCCAACGTCACAAATTCCACTGGCACTCCCTCTGATTAGATAAAGTCTAAAATTCACGCCGTCAATAGAACTATTACAATTGGAGCAAGAATCCTAATTGCACCATGAATGGGATCTAGATCGGACTCGTCACACGTCAACCTAATAGACAGCCAAGAGCCTCTCGAATAGCATTTCAATGCCGACTGTTTCAACTCTGCCAGTCTGCCAGCTTTNNNNNTAGTAGGTNCNNTNCNNNTANNNAATGTATGCGTACNGCAGCCATGTACTATGTCTGAATTAATACTCTAAAATCTATCGACCACAAAAAATGATATCGCTGATACACCCGTCCTCACTAAACGTTCTACTACAGGTTATCCATACTGCAGATAGACATAAGGAGCAGCTTTGCATTGGCAAGCTAGGGCGTGCAGTGTTATGTAACATTTCATAACTTACAGTGAACAAAAACACACGCATCCTTACAGAGCTATTTACTTGCATATGAGTGATTCGCACTTCAAAATGTATATTGTAGCAATTTGGCTGTTGAGTCAACATTTAATGAGACCCTTCGATGCCACTGAAAGGCAGCTGTTATAGCATTAATCGTTTCCTCGGGAGGGTAAAAACAGTTTCGATATCCTTTTTGAAAGGGGACTATTTGCACACAGCACACGTAAACAAAGATCGTATAGGCAAAGGAGCCGTAACAAAGTCGAACTTCTATATATTACATCATATAGGGGGACGTATGTACGTATACATTAGGTGAATACCGCATGAAAACTGTCTATACACGTTCCATCCACATGCATGCAGCAAATGGAATTGGTGTTACTAAACACAAGAAATATCTATCATTCCGCGGCGCCATGGCAACTATAGGGTTTGTGTGCGCGTATAAATTGAGGCGCGAAGTCAGACTGACCACAAGTAGACGCAGCAAATCAAAAATTTAGGATAACAGCTCTTGCGTTGCATTACGTCAAAATGGGACGTCTATCAGTCATAGCATTTCTGGCAGCCGCAGCTAGAGTGATTTTGTCCGCACCAACACCTCCCCAGATACTGGTCGTGGAACCATCCAGTGGCCCGGTGTGCGTTCGCTTCAACAGTCCCGTGTGCAANGCTCTAGGGGGACAAGGGTACAATTACGCGACGTTCCCGAACCCCATAGCTCCGGCCTATCTCCCCAATTCCCAGAAAGCCGAGAGCGAAGCCAATGCGGTGATCCAGCAGGCCCTGGCCAGCCGATGTTCTAAGTACGTTGTCGAATTTATGTGCTTCGCCTACTTCCCGCTGTGCGATCCGAACAACACGAGGGCACCCCCCGTGATGCCGTGCAGGTCACTATGCGAGCGGGTTCGGAGCGATTGCGAACCTGTGCTCAATGCCACGTACGGAACGCGTTGGCCCCAATGGGCGAACTGCGAAAANANNGACAAANGNCGTAACCCAGAACCACGCCGATGACCTGTGCTTTGGAAAGGCTGTGGCCGACACTATTCTTACGGAAGCGACGACGACGCAGCCTGCTACCACTGCCACGACAACGCAGCCTGCTACCACTGCCACGACGGCGGCACATCAGAGTGCGCCACAGCGTCCACTGTGCAGCGCCTGCAATCCCGTCCAGGTCACCCCCGAAGCGTTCAAGCTTAGAAATTACACATTTGGTAAGAAGAACATTGCATTTTGCACTGTGCAAGCGTTCACACTTTACTGACTCTCTTTTTCGTGCCACTACAGCTGCAAAGGTGCAAATCAAGTCCAAGTCAACAGCTGATAAGGTCATCACATACGGGGCGGAAGTCTTGCAATACTCACCGCTGCAACAGCAGTGCAGTTCGAACTCGTCTGTTCGATTACCTCCCAAAACGGTGATCATTTCGATGACGGCTAATGGCCAATGTGCGCCTTGCTTTGTCTTGAAACCTGGACAGATCTATCTGGTTGGCGGGAGCACTAGTCAGCAAGACAAAAGTGGCGTATGGTTGGTGCCAAANNNNGCAGNCNTNNNNNNNCCTTGGANNAATCAATACACCCAAAAATGGGTGCAAATGACTGCGAAGAGTGACAAGTGCCAGCTCGACCCCGTTCACGTCTGAGACTCGCTTCGCACACGTCAGTCTATACCCGTTCCAAAAACTATCACAGGTCACTTCATATGCGTTGTGTGAATGGGTGAGAGCGGGAAGATTTTGAAAACCGGCGTTGTACACAACCACATGCACTTTGCACTTTTCCCCTCCACTTTCTTGTTTTGTTGTTGCATGACTTGCAATTTTATGAGATTTATTCAGATTATGTCTTTGTAATTATGTTTATTATGACGTGGAAAGCAATACAGGGTAATTATATCGTGAGCATGATGCTTCAAATAAGCTTGTTCGTGCAAGACTATGTGTAGTACTTGTACGCAGTAATTTAAGCTGCATGGTATATATTGACGTGTCAGAAATGACTGCAAGTATGGCGTTTACTTATGCATGACTAAGGTATCACATGCCAGGGCACTTGAACGTGCCATTTTTAACACGCCAGGTGACTCGACGTGCTTACATTAGCACGAGACATGCGGCTAGGGTCGATCAGCGCTCTCTGATGATCTCATCTTCAGTCTCGTCTTCCGGATTCACGTGCGAGATTTATCACCACACTTGGTGGCAATCGACATATGAGATCGTTTCTGCGAAAAGGGACCTAGTGCACTTGTTTTTTTTTTAGCTATATTCACATAAATAAAAATCTAAAGGATACAAAACAAGAGTCTATTGTGATACAATATAAATTAACACGCAATAGAAATGTAACGCTTGCGTTGAAATATATGGCTAAAAAATAATGCTAATAAAATTTAGTGCCTTACAACTCCATGACAGATAGTCCGACGTAAGAAGAGCTGTGTAGAGGACTTCAGAATATANAAATTGATATATGGGATCGTTTCCGCGAAAAGGGACCTAACGCATGTTAACGCAAGATTTCAAAATTTTTCGTATTCTAGAAGTCTGAAATGTCCTCTACACATTGCTTCTTACACACTGAAAATCGGAGTTTCTATCATGAAGTTGTAAGGCGCGGAATTTTTCATTAGTCGTACATTTCAGTTACGTTTCTATTTGCGCGTACTGGAACTTTATAATGNACGCGTTACGTCCCTTTTCGCGGAAACGATTCCCAATTGAAATCTTGAAAAAACATATTCTANGCCCATTTTCGGGGAAACGATCCTATATGTATTATTCCAAGGATTACAAACTATCCCTCAAACGCATGTACGAATGTCTGCATGTACGCATATCTGCATGTCTGCATGTTTGCATGTCTGCATGTCTGCATGTCTGCATGTTTGCATGTTAAAATGGCGATTACAGTGTGTGTTAAAGCTG>scaffold2727|size46839GTTTCATTCTGTATGCAGGGCAAAGTGGTGTAAGTGTTGGCACTGTACTGATAAAGAGCTCTCTCAGAAGTGAGACTAAGTTTTACATTGAAACAAAGCATATAAAAATTATATCCCTTTCACTTGCTACCATCTCACATGCAGATGTTGGTGAGGATGCTACGAGTGTTAAGAGTGAGAATTAGTATGCATTAACACCCTTTCATATTTTGTATCATTTGTATGCAGGCAGAGGTGGTGTGGGAGATGGTGCTTTACTGAGAAAGAGCGCCACTTGCAGAAGTGAGAGTGGGTTTTAAAGCTCCATAACTGTACATAATATATCATACAATAATAAGCATCAACATACTGAACACTGAGTATGGTCGAATGAAATTGGTACAAAGTGATGCATGTATCGGGGAAAATGATTAACATGGTTTGTGTGATTTAAGGACAATGCATATAAGCACACATGTACATCCTATACTGTTGCATCTAATAGGATAGAGTACCAAAGGATCAAGGGCATCCAATTCTTTTATGCTTTTAGGTGCCAGCCCAATTGCTCATGTAACAGATGTTCATATTGAGAACAGCAGTAGCTGTGTCATAGACCTAACTCAAGGTTGGTTAATTCAATCATNATCATGATATGAGTACTTGAGTTGTCTCTAATTGTATTAACATGGAAATATACAGACGACCCTTGTAGTGCAAATGACCCCTCCATCGGTAAGTTTTAAAGTAGCAATAAAACTGATACTGAACCAAACTGCCTCTGGCAATAACATTTCAGAATTGATCACAGTGCCAAGGCANTCCATAACAATCGGAACTGTCATAGGATGGGGCACATTTGGGGTTGTGTGCAAGGGCACGTATTTAAACCAAGAAGTTGCTGTTAAATCTGTGCCTCTGCCTGACATAAAAACTAAGGATATTACATTGAAAGAAAGAGGCACAAATCCACAGGTTAGTTGTAATCTGTTAGCTTTATGCACACAATATGTATTATATTTGTATGCATGCTGTATCTAGAACTCTTAGGCATCCAAACATTGTCCCATTAATTGGAGTCTCATTTGANNTANNTCACTCACTCACTCACCCGACTGCAACACGGTGTAACAGACGCGGATACTAGAAGGATAAAATTATTGCCACTGGTTTTCTTCTTCTCCAGTACCGCAGTANCCATTCAACGTACCACGGTTACACATTACGAGTGAATAGAGTCATGCGACGACGATTGAAGGCCGGGCGGGCGGGAGAAGAAGGAACCGAATCGTCACAAACGTCTCGCCGTGCCATGCGTGTCACATCGTGAGAGCTAAAGATAGGAAATATAAACGAATGACGTAACATCTCCTGGTCATTCCAGCGGCTGCAATTGTGGGTTACATGTATACAGCAGAGACTATGCATGACCGCAAAACCGCAATTTGTACGTAACTATCACCGTAGATAAATGTAGAAGACACATGATAAAAATCAATTTCCTTTGCAGTCACCAGGATTGGGCGCATATNNNNNNNNNNNNNNNNNNNNNNNNNNNNNNNNNNNNNNNNNNNNNNNNNNNNNNNNNNNNNNNNNNNNNNNNNNNNNNNNNNNNNNNNNNNNNNNNNNNNNNNNNNNNNNNNNNNNNNNNNNNNNNNNNNNNNNNNNNNNNNNNNNNNNNNNNNNNNNNNNNNNNNNNNNNNNNNNNNNNNNNNNNNNNNNNNNNNNNNNNNNNNNNNNNNNNNNNNNNNNNNNNNNNNNNNNNNNNNNNNNNNNNNNNNNNNNNNNNNNNNNNNNNNNNNNNNNNNNNNNNNNNNNNNNNNNNNNNNNNNNNNNNNNNNNNNNNNNNNNNNNNNNNNNNNNNNNNNNNNNNNNNNNNNNNNNNNNNNNNNNNNNNNNNNNNNNNNNNNNNNNNNNNNNNNNNNNNNNNNNNNNNNNNNNNNNNNNNNNNNNNNNNNNNNNNNNNNNNNNNNNNNNNNNNNNNNNNNNNNNNNNNNNNNNNNNNNNNNNNNNNNNNNNNNNNNNNNNNNNNNNNNNNNNNNNNNNNNNNNNNNNNNNNNNNNNNNNNNNNNNNNNNNNNNNNNNNNNNNNNNNNNNNNNNNNNNNNNNNNNNNNNNNNNNNNNNNNNNNNNNNNNNNNNNNNNNNNNNNNNNNNNNNNNNNNNNNNNNNNNNNNNNNNNNNNNNNNNNNNNNNNNNNNNNNNNNNNNNNNNNNNNNNNNNNNNNNNNNNNNNNNNNNNNNNNNNNNNNNNNNNNNNNNNNNNNNNNNNNNNNNNNNNNNNNNNNNNNNNNNNNNNNNNNNNNNNNNNNNNNNNNNNNNNNNNNNNNNNNNNNNNNNNNNNNNNNNNNNNNNNNNNNNNNNNNNNNNNNNNNNNNNNNNNNNNNNNNNNNNNNNNNNNNNNNNNNNNNNNNNNNNNNNNNNNNNNNNNNNNNNNNNNNNNNNNNNNNNNNNNNNNNNNNNNNNNNNNNNNNNNNNNNNNNNNNNNNNNNNNNNNNNNNNNNNNNNNNNNNNNNNNNNNNNNNNNNNNNNNNNNNNNNNNNNNNNNNNNNNNNNNNNNNNNNNNNNNNNNNNNNNNNNNNNNNNNNNNNNNNNNNNNNNNNNNNNNNNNNNNNNNNNNNNNNNNNNNNNNNNNNNNNNNNNNNNNNNNNNNNNNNNNNNNNNNNNNNNNNNNNNNNNNNNNNNNNNNNNNNNNNNNNNNNNNNNNNNNNNNNNNNNNNNNNNNNNNNNNNNNNNNNNNNNNNNNNNNNNNNNNNNNNNNNNNNNNNNNNNNNNNNNNNNNNNNNNNNNNNNNNNNNNNNNNNNNNNNNNNNNNNNNNNNNNNNNNNNNNNNNNNNNNNNNNNNNNNNNNNNNNNNNNNNNNNNNNNNNNNNNNNNNNNNNNNNNNNNNNNNNNNNNNNNNNNNNNNNNNNNNNNNNNNNNNNNNNNNNNNNNNNNNNNNNNNNNNNNNNNNNNNNNNNNNNNNNNNNNNNNNNNNNNNNNNNNNNNNNNNNNNNNNNNNNNNNNNNNNNNNNNNNNNNNNNNNNNNNNNNNNNNNNNNNNNNNNNNNNNNNNNNNNNNNNNNNNNNNNNNNNNNNNNNNNNNNNNNNNNNNNNNNNNNNNNNNNNNNNNNNNNNNNNNNNNNNNNNNNNNNNNNNNNNNNNNNNNNNNNNNNNNNNNNNNNNNNNNNNNNNNNNNNNNNNNNNNNNNNNNNNNNNNNNNNNNNNNNNNNNNNNNNNNNNNNNNNNNNNNNNNNNNNNNNNNNNNNNNNNNNNNNNNNNNNNNNNNNNNNNNNNNNNNNNNNNNNNNNNNNNNNNNNNNNNNNNNNNNNNNNNNNNNNNNNNNNNNNNNNNNNNNNNNNNNNNNNNNNNNNNNNNNNNNNNNNNNNNNNNNNNNNNNNNNNNNNNNNNNNNNNNNNNNNNNNNNNNNNNNNNNNNNNNNNNNNNNNNNNNNNNNNNNNNNNNNNNNNNNNNNNNNNNNNNNNNNNNNNNNNNNNNNNNNNNNNNNNNNNNNNNNNNNNNNNNNNNNNNNNNNNNNNNNNNNNNNNNNNNNNNNNNNNNNNNNNNNNNNNNNNNNNNNNNNNNNNNNNNNNNNNNNNNNNNNNNNNNNNNNNNNNNNNNNNNNNNNNNNNNNNNNNNNNNNNNNNNNNNNNNNNNNNNNNNNNNNNNNNNNNNNNNNNNNNNNNNNNNNNNNNNNNNNNNNNNNNNNNNNNNNNNNNNNNNNNNNNNNNNNNNNNNNNNNNNNNNNNNNNNNNNNNNNNNNNNNNNNNNNNNNNNNNNNNNNNNNNNNNNNNNNNNNNNNNNNNNNNNNNNNNNNNNNNNNNNNNNNNNNNNNNNNNNNNNNNNNNNNNNNNNNNNNNNNNNNNNNNNNNNNNNNNNNNNNNNNNNNNNNNNNNNNNNNNNNNNNNNNNNNNNNNNNNNNNNNNNNNNNNNNNNNNNNNNNNNNNNNNNNNNNNNNNNNNNNNNNNNNNNNNNNNNNNNNNNNNNNNNNNNNNNNNNNNNNNNNNNNNNNNNNNNNNNNNNNNNNNNNNNNNNNNNNNNNNNNNNNNNNNNNNNNNNNNNNNNNNNNNNNNNNNNNNNNNNNNNNNNNNNNNNNNNNNNNNNNNNNNNNNNNNNNNNNNNNNNNNNNNNNNNNNNNNNNNNNNNNNNNNNNNNNNNNNNNNNNNNNNNNNNNNNNNNNNNNNNNNNNNNNNNNNNNNNNNNNNNNNNNNNNNNNNNNNNNNNNNNNNNNNNNNNNNNNNNNNNNNNNNNNNNNNNNNNNNNNNNNNNNNNNNNNNNNNNNNNNNNNNNNNNNNNNNNNNNNNNNNNNNNNNNNNNNNNNNNNNNNNNNNNNNNNNNNNNNNNNNNNNNNNNNNNNNNNNNNNNNNNNNNNNNNNNNNNNNNNNNNNNNNNNNNNNNNNNNNNNNNNNNNNNNNNNNNNNNNNNNNNNNNNNNNNNNNNNNNNNNNNNNNNNNNNNNNNNNNNNNNNNNNNNNNNNNNNNNNNNNNNNNNNNNNNNNNNNNNNNNNNNNNNNNNNNNNNNNNNNNNNNNNNNNNNNNNNNNNNNNNNNNNNNNNNNNNNNNNNNNNNNNNNNNNNNNNNNNNNNNNNNNNNNNNNNNNNNNNNNNNNNNNNNNNNNNNNNNNNNNNNNNNNNNNNNNNNNNNNNNNNNNNNNNNNNNNNNNNNNNNNNNNNNNNNNNNNNNNNNNNNNNNNNNNNNNNNNNNNNNNNNNNNNNNNNNNNNNNNNNNNNNNNNNNNNNNNNNNNNNNNNNNNNNNNNNNNNNNNNNNNNNNNNNNNNNNNNNNNNNNNNNNNNNNNNNNNNNNNNNNNNNNNNNNNNNNNNNNNNNNNNNNNNNNNNNNNNNNNNNNNNNNNNNNNNNNNNNNNNNNNNNNNNNNNNNNNNNNNNNNNNNNNNNNNNNNNNNNNNNNNNNNNNNNNNNNNNNNNNNNNNNNNNNNNNNNNNNNNNNNNNNNNNNNNNNNNNNNNNNNNNNNNNNNNNNNNNNNNNNNNNNNNNNNNNNNNNNNNNNNNNNNNNNNNNNNNNNNNNNNNNNNNNNNNNNNNNNNNNNNNNNNNNNNNNNNNNNNNNNNNNNNNNNNNNNNNNNNNNNNNNNNNNNNNNNNNNNNNNNNNNNNNNNNNNNNNNNNNNNNNNNNNNNNNNNNNNNNNNNNNNNNNNNNNNNNNNNNNNNNNNNNNNNNNNNNNNNNNNNNNNNNNNNNNNNNNNNNNNNNNNNNNNNNNNNNNNNNNNNNNNNNNNNNNNNNNNNNNNNNNNNNNNNNNNNNNNNNNNNNNNNNNNNNNNNNNNNNNNNNNNNNNNNNNNNNNNNNNNNNNNNNNNNNNNNNNNNNNNNNNNNNNNNNNNNNNNNNNNNNNNNNNNNNNNNNNNNNNNNNNNNNNNNNNNNNNNNNNNNNNNNNNNNNNNNNNNNNNNNNNNNNNNNNNNNNCACACACACACACACACACACACACACACACATGTACACCACACACACAAAGCAGGTCTTAGAAATAAATCACAAAAGGGACATTGCATAATAGAACGATGCACTTGAATAGATAATGATCCTGTATCCAGTAATATACGAGGACATTGCTTGAGCTGGCCATGATTATCTTCTTAATGTGGCACTTCCCTCTGAGGAGGATGCAGCCATTTATCCACAAAGCGTTTCCCTTCTAAAGGCGATGGATGTAGCCTCACATGGGGCGCCATATTGGTACTGACAACTGAGTGTGGTGTGAGGTGTTGCAGAGTATCCATGTACACTATTGCTGATCTGACACCCCAATAGATGTTTGATGGCACGCTCACACACAGGTGTTGAAGCTTCATGTCAAAACTGGCTCACCCAGTTAAAGATGGCATGCTGATAAGTACATCAACGCTATGTTTCACCACCACCACCACCAACACCACCACCACCACCACTGATCCATGCCTCATCGCACATATGTAGTAGATGACAATTTATCAGCTATAGACAATTTTTCTTGTGCTTAGATGTTATCAGCCTACCCCTGGAGCTGTCATGTACTTGTGTGTGCCAAATTGATCTAGCAATGGATAGTACAGTGTCCTTTCTGCTATGACNCAGCACCATTNACNNATTCATCCCACACACACACACCTTCTTGGCATCAGGGACATCAGAGCTGATGACCACCCAGCTTGAAGATTACATTCAATATGGTTTGGCCGTCTCCTTGGAGAATAAGAAGCTGAATCTATGCACGCCAAATTCAAGCCACTGCACATGCAAGAAGTGTGCACTAACAAGTTGTAGCATTGCCAGTGTGTGATGAAGGAGCATCTATCAGCATTACGCCACAAAATGTGGCAGATATACCACAACCAAGCAAGACCCATATTATCCACTGTTTTGTGCGTGGTAAATAACGTATTATTCCGCCTTACCAGAAATAATCTACTATACTTGACGACGATTTGTAATAACATCTTTTCAGCAGGCCTATTAGGCTGGTTAGGCAGCTTTCACCCTCTACTAAAATAAGCAGTACTCGAGTGTTCCGTTCNGATCGAGGTACTCTGCTACTGCTAGTTCGCGGTACCGAGTTGTGACACGTAATTCCATAGCAAAAAGGCTGTATATCAATAATACAACACTGTCAGTATNNNACNTTNTAAANNNNTTGTTGAAACCAAAGAATAAGAGTCCAATACAACACAGCACACAGCGTTACAACACAACGTTTCACCTTTCATTCGTCGCTACGTAACCAACCTCCGCGCGCATGCGCGTACATTGCGCATAAATAGCTTGTTGAGGTCTCATCGTTTCGCGGACAACTTGCTTGCCTTGCGCATGGGATAAGTACTCGACTGATGGTTACATGGTGACAACCATTGAAGCCCCACCCCACNNNCNGTANNTNTAGCCTAAGGTTATGAATAAAGCNNNNNNNNNNNNNNNNNNNNNNNNNNNNNNNNNNNNNNNNNNNNNNNNNNNNNNNNNNNNNNNNNNNNNNNNNNNNNNNNNNNNNNNNNNNNNNNNNNNNNNNNNNNNNNNNNNNNNNNNNNNNNNNNNNNNNNNNNNNNNNNNNNNNNNNNNNNNNNNNNNNNNNNNNNNNNNNNNNNNNNNNNNNNNNNNNNNNNNNNNNNNNNNNNNNNNNNNNNNNNNNNNNNNNNNNNNNNNNNNNNNNNNNNNNNNNNNNNNNNNNNNNNNNNNNNNNNNNNNNNNNNNNNNNNNNNNNNNNNNNNNNNNNNNNNNNNNNNNNNNNNNNNNNNNNNNNNNNNNNNNNNNNNNNNNNNNNNNNNNNNNNNNNNNNNNNNNNNNNNNNNNNNNNNNNNNNNNNNNNNNNNNNNNNNNNNNNNNNNNNNNNNNNNNNNNNNNNNNNNNNNNNNNNNNNNNNNNNNNNNNNNNNNNNNNNNNNNNNNNNNNNNNNNNNNNNNNNNNNNNNNNNNNNNNNNNNNNNNNNNNNNNNNNNNNNNNNNNNNNNNNNNNNNNNNNNNNNNNNNNNNNNNNNNNNNNNNNNNNNNNNNNNNNNNNNNNNNNNNNNNNNNNNNNNNNNNNNNNNNNNNNNNNNNNNNNNNNNNNNNNNNNNNNNNNNNNNNNNNNNNNNNNNNNNNNNNNNNNNNNNNNNNNNNNNNNNNNNNNNNNNNNNNNNNNNNNNNNNNNNNNNNNNNNNNNNNNNNNNNNNNNNNNNNNNNNNNNNNNNNNNNNNNNNNNNNNNNNNNNNNNNNNNNNNNNNNNNNNNNNNNNNNNNNNNNNNNNNNNNNNNNNNNNNNNNNNNNNNNNNNNNNNNNNNNNNNNNNNNNNNNNNNNNNNNNNNNNNNNNNNNNNNNNNNNNNNNNNNNNNNNNNNNNNNNNNNNNNNNNNNNNNNNNNNNNNNNNNNNNNNNNNNNNNNNNNNNNNNNNNNNNNNNNNNNNNNNNNNNNNNNNNNNNNNNNNNNNNNNNNNNNNNNNNNNNNNNNNNNNNNNNNNNNNNNNNNNNNNNNNNNNNNNNNNNNNNNNNNNNNNNNNNNNNNNNNNNNNNNNNNNNNNNNNNNNNNNNNNNNNNNNNNNNNNNNNNNNNNNNNNNNNNNNNNNNNNNNNNNNNNNNNNNNNNNNNNNNNNNNNNNNNNNNNNNNNNNNNNNNNNNNNNNNNNNNNNNNNNNNNNNNNNNNNNNNNNNNNNNNNNNNNNNNNNNNNNNNNNNNNNNNNNNNNNNNNNNNNNNNNNNNNNNNNNNNNNNNNNNNNNNNNNNNNNNNNNNNNNNNNNNNNNNNNNNNNNNNNNNNNNNNNNNNNNNNNNNNNNNNNNNNNNNNNNNNNNNNNNNNNNNNNNNNNNNNNNNNNNNNNNNNNNNNNNNNNNNNNNNNNNNNNNNNNNNNNNNNNNNNNNNNNNNNNNNNNNNNNNNNNNNNNNNNNNNNNNNNNNNNNNNNNNNNNNNNNNNNNNNNNNNNNNNNNNNNNNNNNNNNNNNNNNNNNNNNNNNNNNNNNNNNNNNNNNNNNNNNNNNNNNNNNNNNNNNNNNNNNNNNNNNNNNNNNNNNNNNNNNNNNNNNNNNNNNNNNNNNNNNNNNNNNNNNNNNNNNNNNNNNNNNNNNNNNNNNNNNNNNNNNNNNNNNNNNNNNNNNNNNNNNNNNNNNNNNNNNNNNNNNNNNNNNNNNNNNNNNNNNNNNNNNNNNNNNNNNNNNNNNNNNNNNNNNNNNNNNNNNNNNNNNNNNNNNNNNNNNNNNNNNNNNNNNNNNNNNNNNNNNNNNNNNNNNNNNNNNNNNNNNNNNNNNNNNNNNNNNNNNNNNNNNNNNNNNNNNNNNNNNNNNNNNNNNNNNNNNNNNNNNNNNNNNNNNNNNNNNNNNNNNNNNNNNNNNNNNNNNNGTGCAAAGTTAATGCACATTTTCCCGCTCGATTCCGCCATACGTTGAATCGTACGGCGGTTTACCAAAATGACGCGAAACGACCGGGAAAATTACTTTGTACAGCAGTATCTCCGTAATGGATAAAGCAAAAATTACGAACAGAACAAATCAATAATTACAGGATCCCTCTACGTACATCCCGAATTTGAAACGGCTCCTAGCTACCTAAAGATGTAGAGGTTTTGAACAGCTAGGGTATCGGACCTCGTAACGTCGGCACTATGATTGTGCAAGGCACCAAAATACATTTACACATAGAGAAATGGTGTAGGACGAAGTGTTGAAAATAGGGAATCGATAGTGTTAACCGTNGGTAAGTTATAGCAATTTGAGTTTTATGTTATAATTATAAACATAAATTCACAAGGGTCGAAATCTCGTAGAACGTTCCATAATATTATATATCCATGATGCAATTTTCTCGGAACTGTCCCAAATGTTGTAATACTTAACATTACAAACTGGATCAGCAAAGCCACAGCAATTTCTCCATTATCAGCTGAGTGTAGCACTTTGACAGCCTCTACCTGTAACATCGAATAGTTTACAAATGCTGTGCTACGTCATGAATTACTTTTCACATATCTCATTGGAGAGTTGAACACTTCATATGTAAAAGTGAAAAATGCACTGTATGATGGCAACAGTACAAGTAGTGTGGCTCCTTCAAACAGACTCTGGTGCAGCTCGGATATGTGATTCCTAAACAAAATCTCTGTTTCCTCCCCTTTNNTTTTTTTTTTTTTTTNNNNNNNNNNNNNNNNNNNNNNNNNNNNNNNNNNNNNNNNNNNNNNNNNNNNNNNNNNNNNNNNNNNNNNNNNNNNNNNNNNNNNNNNNNNNNNNNNNNNNNNNNNNNNNNNNNNNNNNNNNNNNNNNNNNNNNNNNNNNNNNNNNNNNNNNNNNNNNNNNNNNNNNNNNNNNNNNNNNNNNNNNNNNNNNNNNNNNNNNNACACACACACNCACACACACNCACACACACACCCACACAAACCACATAGAAGGGTTGAGTTGTGTACCCGTGCTTTCTGAGACTTCTATTTGCTGCAGTTGCACCACCACTATGCATACCAAACAAAGTTGGGTCATACCCGAGCTTTGCTATCTTATACTCCCTTAGTCTGGAGAAACTGAGGCCACCTGCTTTTCACAAATGCTCTCCTTTCTCTGTTTGTACTATTCCCCAAAACAGCATTTCATGGGATTAATATTTTAACTTCCCCATACGACAGTACATAACCATCATGCTCACAGGGCATGTACTGACCCAGTGTGTGCAATGACTAAGAATGCACCCTCATGCTATTGGTCCCTGCACATAGATCACCATGCCTTCAGCATTGAAGGTGATATAAGACAGCTCTTTACAGGGACTCTGCCCTTGGTAGCAGGGCCTGCCCTCGGATGGCATATTAAGCTATGTTCCCATGGTCCAGGGGGTTAATACATTAGGTTTTCAACTAAAGACAGGAGTTTGATTCTCCTTAGAGTGATTATCAGCCAAAACATCATTCTCGCATGAAGGATGTTATCTACTGCAGCATATTGCTGAAGTGACCTAAATTGGCTTGTCGTATCACCATAATAGTCCCTCAGACACTTGCAGAGACAATTAGACTCTTTCTTGGTCACAAACTGGTTACTAAATGGAGCGCACACAGGCACGGCAGTCATAGCGGGTGTCACCAGGCAAATAGAAGTCCTTTTTGCGGGAGGCTCCCTCAAACAGGCAGCACAGTAGCGACGGTTCCACGGACATGATGGGAGTTTTCGTATGTGCATCTTTGTGTACTTCAGCTGCGTGTTCCATTTGCCTTATCTTTTGCTCTTTGATAAACTTGTGCCATTAAAATGACAGAGTTCTAAATAAGTGTAGAAAGTTTTCTCAGGTGGACTCTAGTGCCTACCGTGCCAGATGTTGGATGTGACGGCTCTGTGCCCAAATTTCTTGTGGACTGAGCTTGACGATGCAATCTCTTTGTTGTCAGAGCTCTCATCATCGCGGCCACCAAGCCAAGCTTCATGGCAAAGTACTTAGCCAAGTGTCTGTAATCCTGGAAGGATATCGAAACGCTACTGTATTCTAGTGGAGAGAGTTGGCCTTGAAACGTGCACCTGCTTTTACAAAGACATTGTTGCCCCATACAGCTGGGTTATCCTCATTATTGAACGATGCCTGCCAGTAAGCAAGCGAAAAGTCTTACAACTAACAAGTCCGTACTTACAACACGAGATGCCTCCTTGTCTAAGAACCGGGCTGTTTGCCCCTGTAAGGGATCTGGACTTGTCATGTTCCGCATGGAAGTACACATGTTTATAATAGGTGTTCCTGCTTGTACTGGTGTTATTATGGACCTGGTATGCATCCAACTCAGTTAGGGGCCCATGCTGGTGTACCCGATCCCAATGACAAGGAAGTTCTCGATATACTGATCCATACCTCTTGAGGTACGACTGAGTGATTGAACACCACCGTTAATTGAAGATATGAGCTTTGGCTATAGCTTCTCGGAAGTGAGTGCAGATGTACTTCAAGAGACGAGAGCAAATGGCTTTGTCTTTTTTGATGTCCTTGTTGCCATGATTTGGTAACCGAGAGATCCATTTGCAAAGTTGTCGTAAATGTAATCCATGGTTACGCCGCTAGCTCCTTGAAGAAGCTCCTAATCTACAGTTTAGAGCTTGGTGATACACCGTGCCAATCTGATNGGCTTTCAATGTCAGGCAAATTCAAAGCTACCAGACACAAGTCTTCATACTAAACTTTTGTTTCCAAAGGGATTGGTTAACATGCCTCACATTAACTGCAAGCTGGTTTTTTTTTATTACCCACGTCATTGGTGACGACAGATAATGGGCTGCGTACAATTGGGGGCTCCCTTACCTCTTCAGTGTACCCACTCTGTAGCAATTCTGCTACTGCATTTGTAACAAACTCTAGCTCTACCTGAGCAGAATGATGATTGGCCGTACATAGGATAGGTGAGAATAAAAGGGGAGAACATATCCATTCCTAAGTATTTCCAACACCCATGGCGGTGCACATAGCATCTCCTCCCAATATGAAACACAGGCACCCAAACATCACCTTAGAACCCAGTCCTGCATCTCCTTCTTGGGGAGTTGGGAATACATTTGTCCACCAGGTGACATAATCTCACCTCTCTGGTGATACATTCCCTCTATCAGTGCCCACATGCTCAACATCATTGATACTAAAGCTCTCTTTGTAGGCCTGCATGGTCTCTCCTGGGGAATCACTTCCAACTCTTCTTGCATTTGGCGCTTGCCTTCCTTGCAGGAGCTGGGCAATATTGTAGGTCATCGTAAAAAAGTTAGTAGCACTTGCATACGACACTTAGACTGAACTAAAATCCTATTAAAACCTTCACCCATGTGTTCACATCACTTGCTGTACGACCAATTACATATGGAGGAACATATTTTCTCTCCAGTTCACTGTGTTGAACTGACAGAAACATATATTTTATTGTGACCAGTGTGACTTAACGGCTTTTGTATTTTTTATGCTGTCTTTTGTACACCTGATTTGACCCTTAATCTTTATCTTCAAAGTGAACAATGATTCCTCCGAAGAAGGCTGTGCGTTTAACCCTTAGGTTCAGTAGGGTGTGTGACCTTTCATGTTTGTTTTGTGATGGATTGTAAAGCTTTGCTTCTGTTGTGATGGCAAATTGTTTGTCATAATAAATGTGCTATCTCTTAGCATGGTGGTTTACGTCTGGAAACTTACTAAAGACTGCTATTACTTATGGTGATAATTCATATCTAAAACTTAAAAAGGACCAGATATTAAATCCATGTACATGTACCATATATGTATATGGGATTGAATAATTTTTTGGAAAATTTCAATTTACCAAGAAAAATATTTGAAGGTAATTTCTTGATGGAAGTGCAACACAGTTCCTGGATATCAAATGTATGGGAGGCAGTGGTCTGATGAAGGAAGGGCTTGCATATGAGGGAAAGAGCTCTGGGGAATGCTGTGGTGGGGAAATTTTGCACATCTAATCTTCGCTTTATATGAAAATACTAAATTAACTTAATGCAAATATAGTATATGATGATAGTTTTTTTCAACTACAGAACCACTGTTGCAAGGTTGTCACAGGAACGTGACCCCTGTGTTGGGTGACGCTACCAGTGATGTGCACCACTGTGGGNNCNTCAGCAGTATTAGGGGCCCCTCCTCCCCAGGCTGTTATCCAGCAGCATGAAACAAGTGCTTGTATGTGAACTGTGTGTATATATATGTGGTGCGTATGTGTGTGTCTTCAATGTGCAAATTGTGAAGTTGAAATTTTAAACCAAACGGAAACTGGGTGAACTACATATATTGACTAAAGCAAGCAGTGATGGTGAGTACAAGATGGGAGTCACTGGGTGGCCAGGACAGTAGCAGTGCCGCACAATGCCCTTATAAGCAGAGAAGTAATTTCAACCTCTCCAACCATAGTGATGGTGCTATTCTCCATCTTGAAGAGCAGTCATACAACCAGTGTTTGAATAAAGCACTTCCTATATCTTCTCAAGTACCAGTTGCTCATTACAGAGTACAGATCAAAAGAGTACAGATCAAAATCAAGACATGCTGGGACTACTTTGGATAAGGTTACGAATAAACATTAATAACATGGTAATACATATAATTATTCCATACACTTATAGCAACAGCATTTAATGCATGACCACTGAAACTCATTGACAAGTGGTATTCACCTTGACAGTATTGCCATAGCATTAATTGGCTCTGACAATGGAAAAGTTACATAATGTTTGATGTGCATAAGAGGAAGCATTGCCCTGCTGGGGTGCAATGGACACCCCGTGGCAGATGTCTATTAATAATTGAAGTTATTGAAAATAAAATACTGGTAATTATGTCTGCTTGAATCACAGCTGGATAAATGAGTAATTAGAACGTACTACGAGATAATGACCCTAGTGGATTTATGTTTATAACCTAAAACCCAAATTGCTATAATTTACGAACGGTTAACGCTATCGACTTCCCATTTTCAACTTCGTCCTGCACCATTTCACTATGCGAATATGTATTTTGGTNNNNNNNNNNNNNNNNNNNNNNNNNNNNNNNNNNNNNNNNNNNTTGTACTTGAATAAACCCCCACATCTTTAGGTAGGAGCGTTCAAATGTCTTCAAATTCAGTGTTTACGTAGAGCAGTCCTGTCGGTATTTATTTGTTTAGTTCGTTAATTGTATCCGATCCGTTACGGAGATACCGCTGTGCAAAGTTAATGCACATTTTCCCGNNNNNNNNNNNNNNNNNNNNNNNNNNNNNNNNNNNNNNNNNNNNNNNNNNNNNNNNNNNNNNNNNNNNNNNNNNNNNNNNNNNNNNNNNNNNNNNNNNNNNNNNNNNNNNNNNNNNNNNNNNNNNNNNNNNNNNNNNNNNNNNNNNNNNNNNNNNNNNNNNNNNNNNNNNNNNNNNNNNNNNNNNNNNNNNNNNNNNNNNNNNNNNNNNNNNNNNNNNNNNNNNNNNNNNNNNNNNNNNNNNNNNNNNNNNNNNNNNNNNNNNNNNNNNNNNNNNNNNNNNNNNNNNNNNNNNNNNNNNNNNNNNNNNNNNNNNNNNNNNNNNNNNNNNNNNNNNNNNNNNNNNNNNNNNNNNNNNNNNNNNNNNNNNNNNNNNNNNNNNNNNNNNNNNNNNNNNNNNNNNNNNNNNNNNNNNNNNNNNNNNNNNNNNNNNNNNNNNNNNNNNNNNNNNNNNNNNNNNNNNNNNNNNNNNNNNNNNNNNNNNNNNNNNNNNNNNNNNNNNNNNNNNNNNNNNNNNNNNNNNNNNNNNNNNNNNNNNNNNNNNNNNNNNNNNNNNNNNNNNNNNNNNNNNNNNNNNNNNNNNNNNNNNNNNNNNNNNNNNNNNNNNNNNNNNNNNNNNNNNNNNNNNNNNNNNNNNNNNNNNNNNNNNNNNNNNNNNNNNNNNNNNNNNNNNNNNNNNNNNNNNNNNNNNNNNNNNNNNNNNNNNNNNNNNNNNNNNNNNNNNNNNNNNNNNNNNNNNNNNNNNNNNNNNNNNNNNNNNNNNNNNNNNNNNNNNNNNNNNNNNNNNNNNNNNNNNNNNNNNNNNNNNNNNNNNNNNNNNNNNNNNNNNNNNNNNNNNNNNNNNNNNNNNNNNNNNNNNNNNNNNNNNNNNNNNNNNNNNNNNNNNNNNNNNNNNNNNNNNNNNNNNNNNNNNNNNNNNNNNNNNNNNNNNNNNNNNNNNNNNNNNNNNNNNNNNNNNNNNNNNNNNNNNNNNNNNNNNNNNNNNNNNNNNNNNNNNNNNNNNNNNNNNNNNNNNNNNNNNNNNNNNNNNNNNNNNNNNNNNNNNNNNNNNNNNNNNNNNNNNNNNNNNNNNNNNNNNNNNNNNNNNNNNNNNNNNNNNNNNNNNNNNNNNNNNNNNNNNNNNNNNNNNNNNNNNNNNNNNNNNNNNNNNNNNNNNNNNNNNNNNNNNNNNNNNNNNNNNNNNNNNNNNNNNNNNNNNNNNNNNNNNNNNNNNNNNNNNNNNNNNNNNNNNNNNNNNNNNNNNNNNNNNNNNNNNNNNNNNNNNNNNNNNNNNNNNNNNNNNNNNNNNNNNNNNNNNNNNNNNNNNNNNNNNNNNNNNNNNNNNNNNNNNNNNNNNNNNNNNNNNNNNNNNNNNNNNNNNNNNNNNNNNNNNNNNNNNNNNNNNNNNNNNNNNNNNNNNNNNNNNNNNNNNNNNNNNNNNNNNNNNNNNNNNNNNNNNNNNNNNNNNNNNNNNNNNNNNNNNNNNNNNNNNNNNNNNNNNNNNNNNNNNNNNNNNNNNNNNNNNNNNNNNNNNNNNNNNNNNNNNNNNNNNNNNNNNNNNNNNNNNNNNNNNNNNNNNNNNNNNNNNNNNNNNNNNNNNNNNNNNNNNNNNNNNNNNNNNNNNNNNNNNNNNNNNNNNNNNNNNNNNNNNNNNNNNNNNNNNNNNNNNNNNNNNNNNNNNNNNNNNNNNNNNNNNNNNNNNNNNNNNNNNNNNNNNNNNNNNNNNNNNNNNNNNNNNNNNNNNNNNNNNNNNNNNNNNNNNNNNNNNNNNNNNNNNNNNNNNNNNNNNNNNNNNNNNNNNNNNNNNNNNNNNNNNNNNNNNNNNNNNNNNNNNNNNNNNNNNNNNNNNNNNNNNNNNNNNNNNNNNNNNNNNNNNNNNNNNNNNNNNNNNNNNNNNNNNNNNNNNNNNNNNNNNNNNNNNNNNNNNNNNNNNNNNNNNNNNNNNNNNNNNNNNNNNNNNNNNNNNNNNNNNNNNNNNNNNNNNNNNNNNNNNNNNNNNNNNNNNNNNNNNNNNNNNNNNNNNNNNNNNNNNNNNNNNNNNNNNNNNNNNNNNNNNNNNNNNNNNNNNNNNNNNNNNNNNNNNNNNNNNNNNNNNNNNNNNNNNNNNNNNNNNNNNNNNNNNNNNNNNNNNNNNNNNNNNNNNNNNNNNNNNNNNNNNNNNNNNNNNNNNNNNNNNNNNNNNNNNNNNNNNNNNGTGTGTGTGTGTGTGTGTGTGTGTGTCCATGTATGTGTTGTACTCGGTGTATTGTGTGTGGATGACGTTTTAACCTACATGTCCTTATTCATTTCAGGTGACATGTTTGCACCGTTCTTCACCGCAAATGATGTCTACGACACCAAATTTTATACCTACAATTTAGGTGGGCATCCATTGGAAAATGCAGTGACATGTCTTTAATGAGCACTCTCACATGTAGACCAGTGTCAGTGGAGCAAAGTGGAGGTCACTGGGTACCGGCCTGATGGCAGAAGAAGCCATTGTGCCGTTCACTATGGCAACAGCATTATCTATTTTGGTGGATACAATGCTCTGAGCAAACAACACTTTGGAGATCTCTTCATTCTCAACACAGGTATTGCACATATCACCTCACACAATACACATACAGTACTGCCCAGACACAGGTATTACGTGCAGCTCCACATACACATAAAGTACCACCCAGATGCAACTTGGCACATGCGTTTGATAATGGGTCTTTTCGTGATCGCGCGCTCGCAATACCACCATCATTCGCAACTCATCAGACACTTCCCTGAGTCGAATCTGTACATTCTGATNNNANATGTACATATTGGCAATTTTTAATAAAAATTGAAAAGCTCTCATGAGTTTACGATACATGTGTGGGTCATGTGTGTACTACACATGCAAACACGCATCCACACATGCTTGTATGGCATGCNNTGGTGCTCCGATATGTGTGGTCACACACTCTTAAAGCTGCATTCTCTATTTATATACAGAGAGCCTTCACATTACAAAGCTGCAGCCTTTTGGTGATGCCCCGTGTCCTAGACGAAGAGTGGGGTGTGCTTTGATTGGAAGTGAATTCATCATATGTGGAGGCACAAGGTAGGTAGAGGTTCGGAGAGTGGAAAGTGCAGTGTCTTTTGTTCCTTTCTTTAACCCTTCACTGACCACCATCCTTCTTTAACCCTTCACTGACCACCCTGCCCTCTTTAACCCTTCAGTAACCACCCTGCCCTCCACGGACCACCCTGCCCTTTTAACCCTTTGCAGACCACCCTGCTCTCTTTAACCCTTTGCTGACCACCATGCCCTCTTAACCCTTCACTGACCACTATTTCTTCATCAACTCTTCATTTTTCAACAGTCCAATTGAACACATGCGGGATGGCAAGAAGCAGCAGATCTTGCATGACCATAATGACATGTACATCCTCCACTTGGGTGAGTCCACCTCATCATAATAATACTACTATCTTACGGTTGTTGTCCCATTTAGTGCCCTCCTTGGAACAGTTATGCACAATAACCATTCTGAAGAGCAGGACAGACATTAAAGCACTTCCTCTGTTTTTACAAGAACGAGTGTAAGTGACTTGTGCCTGTGTGTGTGGTAGGCTCTGCCTGTCTCTGTGACTGTAATATGGTATGATGTACACTTCATACCTAGTCACAAGGCCTTCTCCTAGGACCAATTGTATGTAGGCATTAAGGACAAGGGCAACCAGTCAACAGAGTTTGTCCTTGAGCCCCCCCTCCCNNCTCATGCCCCCAGTAGCCCCTCCCTCCTCATGCCCCCAGTAGCTCCTCCCTCCTCGTGCCCTCAGTAGCCCCTCCCCCCAGTAGCCCCTCCCTCCTAATGCCCCCAGTAGCCCCTGCTCCCAGTAGCCCTTCCCTCCTCATGCCCCCAGTAGCCCTCCCTCCTCATGCCCCCAGTAATTCATCCCTCATGCCCNCAGTAGCCCCTCCCTCCTCATGCCCCCAGTAGCCCCTCCCTCCTCATGCCCCCAGTAGCTCATCCCTCATGCCCTCAGTAGCCCCTCCCTCCTCGTGCCCCCAGTAGCCCCTCCCTCCTCNNNNNNNNNNNNNNNNNNNNNNNNNNNNNNNNNNNNNNNNNNNNNNNNNNNNNNNNNNNNNNNNNNNNNNNNNNNNNNNNNNNNNNNNNNNNNNNNNNNNNNNNNNNNNNNNNNNNNNNNNNNNNNNNNNNNNNNNNNNNNNNNNNNNNNNNNNNNNNNNNNNNNNNNNNNNNNNNNNNNNNNNNNNNNNNNNNNNNNNNNNNNNNNNNNNNNNNNNNNNNNNNNNNNNNNNNNNNNNNNNNNNNNNNNNNNNNNNNNNNNNNNNNNNNNNNNNNNNNNNNNNNNNNNNNNNNNNNNNNNNNNNNNNNNNNNNNNNNNNNNNNNNNNNNNNNNNNNNNNNNNNNNNNNNNNNNNNNNNNNNNNNNNNNNNNNNNNNNNNNNNNNNNNNNNNNNNNNNNNNNNNNNNNNNNNNNNNNNNNNNNNNNNNNNNNNNNNNNNNNNNNNNNNNNNNNNNNNNNNNNNNNNNNNNNNNNNNNNNNNNNNNNNNNNNNNNNNNNNNNNNNNNNNNNNNNNNNNNNNNNNNNNNNNNNNNNNNNNNNNNNNNNNNNNNNNNNNNNNNNNNNNNNNNNNNNNNNNNNNNNNNNNNNNNNNNNNNNNNNNNNNNNNNNNNNNNNNNNNNNNNNNNNNNNNNNNNNNNNNNNNNNNNNNNNNNNNNNNNNNNNNNNNNNNNNNNNNNNNNNNNNNNNNNNNNNNNNNNNNNNNNNNNNNNNNNNNNNNNNNNNNNNNNNNNNNNNNNNNNNNNNNNNNNNNNNNNNNNNNNNNNNNNNNNNNNNNNNNNNNNNNNNNNNNNNNNNNNNNNNNNNNNNNNNNNNNNNNNNNNNNNNNNNNNNNNNNNNNNNNNNNNNNNNNNNNNNNNNNNNNNNNNNNNNNNNNNNNNNNNNNNNNNNNNNNNNNNNNNNNNNNNNNNNNNNNNNNNNNNNNNNNNNNNNNNNNNNNNNNNNNNNNNNNNNNNNNNNNNNNNNNNNNNNNNNNNNNNNNNNNNNNNNNNNNNNNNNNNNNNNNNNNNNNNNNNNNNNNNNNNNNNNNNNNNNNNNNNNNNNNNNNNNNNNNNNNNNNNNNNNNNNNNNNNNNNNNNNNNNNNNNNNNNNNNNNNNNNNNNNNNNNNNNNNNNNNNNNNNNNNNNNNNNNNNNNNNNNNNNNNNNNNNNNNNNNNNNNNNNNNNNNNNNNNNNNNNNNNNNNNNNNNNNNNNNNNNNNNNNNNNNNNNNNNNNNNNNNNNNNNNNNNNNNNNNNNNNNNNNNNNNNNNNNNNNNNNNNNNNNNNNNNNNNNNNNNNNNNNNNNNNNNNNNNNNAGGGAAGGAGAGGGGAGGGAAGGGAAGGAGAGGGGAGGGAAGGGAAGGAGAGGAGAGGTGAGGAGAGGAGAGGAGAGGGAGAGGTAGGGAAGAAAAGGGAGGCTCCTTGAAGGGGTTGGATTGCTCCCAGTCTACTCTCTCAATGTCTTATCCTTACCCCTTAGCTGTCGCATGTCCTCAGATCCCAGCACCCAAGAGCAATAGCTTCATCTGTACAGCCACCCCCCCTGCTCCCCAGTTTGGGGCAACCATCAAGTACTGCTGTGCAGAGGGTCATCAAGTGGTGGGGGGAGGAGGCTCAGACTATGTGACCTGCACAGAAGATGGAGTATGGAGTGCCTCNNNNNNNNNNNNCCAAGGTGACAGTGTGGGGTATGGTGATAGGAGTGTGCCAGATGATGGGTGTGGCTAATGTTAATTTGGTGCAGGGACTGACCAGTTTCTCTCTTGTTGGCACACAACAGAAGATGGCTCAAGAAAGTTGGGCTTACTATGTACTATTTTTCAGTGTATGTGACATTTCTGCTACAGTAAATGTGAGCATCATATTTACTATTGTGCAAGTGCCACTATATCAGACCAGTAGTAAATTCTAGTAAACCCAACTTTTCACCTAGTGAAGGTGCCAGAGGGTAGGGGGAGGGTGCAGAAGGAAGGGAGGGGAGATGCCACAGGGGGTTGGGTAGGGAGTAATGGTGTAGAAAGACATGACAACAAGAATTGTGCCAAAAACCAAATTGCACAAAGTGTTATGATAGCTTTTCACATTTATGCATAGCAACCACATGCCTGACCCCACCCGCCCCCTCCAATGGAGCACTAACTGTTAATAACTCTGGAGGAGGCATGGTTGCACTTGCTGGATCCTCCAACCTCAGTTACCATGGATACAAGTCACAGATCACATTCAGGTGCAATGCCGGCTATGTTCTGATTGGAGAATCTGTACTGACCTGTGGCCCCACAGGAAGCTGGGATCATCCAACTCCTGCTTGTCGAAGTAAGCTTTAATTAATGCTCACTAAGAGCACAGAAAAGTGTTAATTCATTGGGAATGTGGTATTATCCATTGGGATGTACAGTTAGTGTGGTGTATTGTGTCTGTGGCAGTATTACACACCAGTTCAAATGATTTATGTGTGACACATCCCACCTCCACCTTACGCACCTCCTCCTACCCCACCTCCTCCTACCCCACCACCTCCCACTTTACACACCTCCTCCTACCCCACCACCTCCCACCTTACACACCTCCTACCCCACCACCTCNCACCTTACACACCTCTTCCTACCCCACCACCTCCCACCTTACACACCTCTTCCTACCCCACAACCTCCCACCTTACACACCTCCTCCCACCTTACACACCTCCTACCCTACACTTTGCACTTTCCATGTGCACACAGTGTCGCAATGTGACTACAGGTGCCCTCCCCCTCCCATCCCGACCAATGGGAGGCTTGCAAACATCATCTACCCAGCGTCAACCAACTCTACCCTTGTGCTGGGCACAGTGGCTGTGTTTGTGTGTGACGCTGGCTTCTATCTGGCCGGAAGTGCTAGCAGGACTTGCAGGGAGAATGGCATGTGGAGCGATCAGGTGACCCGCTGCTACCCATGTAAGCTACACTGCATATAGTCTGTCTGGTCACATGACCACTATGTAACTAAGTGATGATACCACATGGTGGTCACGTGTGACTGTGGGTTGCTATCCCCGTGTTTACAATTAGGGAACATTTGACCCCTGTAAGGGTAACAAAAGGTGGTCACATGACTGTGGGTTGCTATCCAGTGTTTACAATTATGGGTCATGCCGGTCATGTGACCCATGTAAGGGGTGTACCACATGGTGGTCATATTTTATGATTTGGGGTGAGAGGATGTTGATGTGAACAGGGCCCATGATTACCACCATATAATCACTAGTTCTTAAATGAGTTAGGAAATCACTTTTATTTATTTTGCAGTTGACCAGCCGAGAAGCAAGCTCCTGTACAAGATAGGCGACATGCCTTGGCAGTCGAATGAGCAGGCTTACATGAGCTCAACAGATCGCATAGTCCTGGCGTGTCAGACACAGCTAAAGGAGACGGGACTGGTGCAGTCTATCTCAAAGCTGTCTGGACCAACCCTGGATCGAAATGTCCGTGTGGTGCAATCGGGATCACCTGACAGCAGTCTGCAGTTGTCCCTTCAACCACTCACAGCTTCCTCAGGCTTAAGTGTGGCTGGCGTGTATAAGTGCCAAGTGAGGTCAACGACAGATGGATCCATTGTGCTAATAGAACTCCTGAAGATTAAAGTTTACAGTGAGGAGAGAGAGGGGGGGAGAGGGGCTATATTTGTCTGATCCTATAATTTGGTGTNNGTCAATGTTTGTGTGTGTGTGTGTGTGTGTGTGTGTGTGTNTNTGTNTNTGTGTATGTGTGCAGGTATGTTTGTGTGTGCCTTGAATATCTGAATGAACAAAGTCAAGATGAACCCTCAGTGATAACAGCTCTGTGATCTTGTTATTTTGTATCGTGAAAGTGCCTTGCAATGATGTGTGTAAAGTGTAGTGGGCCATATCCTTGTACCACCAACCTGCTGTCTATTCCAGTACCAATAACTTCAGATGTTCATTCCTAGATGCCCATTCCAATACCACTAACTTCAGATGTTCATTCCTAGGTGCCCATTCCAATACCACTAACTTCAGATGTTCATTCTAGGTGCCCATTCCAATACCACTAACTTCAGATGTTAATTTCTAGGTGCCCATTCCAATACCAATAACTTCAGATATTTATTCCTAGATCCCCATTCCAATACCACTAACTTCAGATGTTCATTCCTAGGTGCCCATTCCAATNCCACTAACTTCAGATGTTCATTCCTAGNTGCCCATTCCAATACCACTAACTTCAGATGTTCATTCCTAGNTNCCCATTCCAATACCACTAACTTCAGATGTTCATTCCTAGGTGCCCATTATACTAACTTCAGGTGTTCATTTCTAGGTACCCATTCCAGTACCACAAACCACTCTCCACTAGTATCACTAACCATGCCATCTATTCTGATACCTAACTCCATATGCTTTTCTAGAGCCACATAACAAGCCTTGAGGGTAATGCATGTGCTCCATTTGGAAGCCCACACTCTCATCAATACATATACTTCCACTCACCATGCACCCATGCTCAGATGTGCTGTGTCCCATTCCCATGTCCCCGGCCAATGGAAGCCTCCTAACAGTGGGCACGCCCCCAGGGAGCTGCTCCCTCTACACCTGCAACGAAGGCTACTCATTGGTTGGCATGGAGACAAGCGTGTGTTATGAGAACAGGACTTGGAGCAACAAGCAACCAGTGTGCGGTAAGCCCAACTTCCACGAGACCCCACTGTGACCCCTGACTCTAAAGAATGCACGTTTGTAATTATCAAAATCAATAAGTCACAATATGCCAATCTCATATAACTTTGTCACTGCAGAGAGCACAATCAAACACAATGTGCACTTATATCACGCAAAATGGTCAAATGCTTGCTCTAAAGCTTCCCCTACAACATAAATTAATCCAGTTAGTTATGAATGTATGCCTGAGCACTTGCGCAGTTATTTATCGTCATCATCTCTCTAATCTCTTAGTTCCCACCACAGGCATATTGTGTGATCCGCCTGTTCTGACTGCTTCAGTGTTTGTCAGCCCCCCACCCTCAGTCAGTGNTCNNTANAGAGTTGGAGACGCAGTGGTGTACCGCTGCGACCCTCCCACGTACCGGATGGTCGGTGCAAGCGGTGCTGTGTGTAAAAGTGACTCAATTTGGAGCGCAGCCCCACCTATTTGTATCGCCACGCTGTGATGTAATGGCCTATGCATTCTTAATATACGATATCTTTTTATGCGTATATACGAAGANNNANNNNNNNGCTTGTGTAGTAACTAAACAATGAAAAGAAGTCGTGCCGGTAAGAGCAGTAGTAAGTCACCGCCAAAGACCAGCCTCAAACCTTCTGCCGCCGAAGTAGTGGAGAAGAAGGAAGAGAATTGCGAACGTCGCTTCTTCTGGTCATGCTACAAAGACAGTGGCTTCCCGCACCGAGTGAATCACGCAGTGGCCGCGTACTCTGACGCGTGCGGCAACAGTTACATGTACTCTGTCGGCGGTTACCATGCTGACGACGACCAGAGGACCGTGCTGCAAGCAGACTCTATTGGGGGCCCGTTTTTCAGGCGGTCGCCAATAGATATTCACTGCATGGATATCGGTAAAGAAGCGTAGCGAGAAGAATTGTGGTAGCCTTGGTACTTTTCAAGCGTGTTCACCGCTTTGCAGGGACTCGTGTTTGGATGAAGGAAGAACCGTGCGCGCGTAAGGAGAGAAGCATAGCGCCCTATGGGGAAACGGCCATACCCGGATGTCGCTATGGACACACAGTGTGCGCGTACGGAGGAAACCTGTACATGTACGGTGGTCGCAATGACGAAGACGGTTCTTTCTCCTCCATGGAATGCTACGACATTGGTGAGAGCGATAGTTTAATGTAATGAAGTAGCGGAGAGATGCCATCTTCTTGGCACTTCACTGCTCCGTATCCGACTGGCACAACGTCTTAATCAGCGTCAGTTCAGTATCGTTATTTTTTGCCTGGCCAGCGAAGCCCCTCCCCCTATACGACGCATTTTCTCATTCGTAGAACACAAGGTGTGGTTGAAGCTCCATTGTGACGGAGAGTCTCCCAGCTCACGTGATGGTCATGCCTGCACGGTCATTGGCAAGGACATGTTCATTCATGGAGGATTTGCTGCCAATGTGAGTGTGTGTATAAGTGGAAGGTGTGTATTTGTGGGTGTGGTCGAGCGGTGCTATTTGCTTTTTGGTCATACATGCTAGGAACCTGCATGTATCAGTTCTTAATAAACAACAGTGCATTTATGTTATGAGAGGAACGGAGCCTGGGGTCAAGAATTACAATTATGAGTGTGTACAGATTTGCAGTGTTGTGATGTAAACAACAATAGAAAACAATTTGCAAACGAAGGTCTTCTCCCCATATAGTTGAATGTAAATAGAAGACTTGCACTTTTTTTTAAGGCATTGTTGTGTTAATGGTTGCTATGGTTACCAACAACACTGTATAGGAAATGAAATTCAATGGCGACACCTACAAATTTGACATGATGTCACATATCTGGACCAAATTGCCCTGTCAAGTATGTTTTGTGTGTGTGTGTGTGTGTGTGTGTGTGTGNNNNNNNNNNNNNNNNNNNNNNNNNNNNNNNNNNNNNNNNNNNNNNNNNNNNNNNNNNNNNNNNNNNNNNNNNNNNNNNNNNNNNNNNNNNNNNNNNNNNNNNNNNNNNNNNNNNNNNNNNNNNNNNNNNNNNNNNNNNNNNNNNNNNNNNNNNNNNNNNNNNNNNNNNNNNNNNNNNNNNNNNNNNNNNNNNNNNNNNNNNNNNNNNNNNNNNNNNNNNNNNNNNNNNNNNNNNNNNNNNNNNNNNNNNNNNNNNNNNNNNNNNNNNNNNNNNNNNNNNNNNNNNNNNNNNNNNNNNNNNNNNNNNNNNNNNNNNNNNNNNNNNNNNNNNNNNNNNNNNNNNNNNNNNNNNNNNNNNNNNNNNNNNNNNNNNNNNNNNNNNNNNNNNNNNNNNNNNNNNNNNNNNNNNNNNNNNNNNNNNNNNNNNNNNNNNNNNNNNNNNNNNNNNNNNNNNNNNNNNNNNNNNNNNNNNNNNNNNNNNNNNNNNNNNNNNNNNNNNNNNNNNNNNNNNNNNNNNNNNNNNNNNNNNNNNNNNNNNNNNNNNNNNNNNNNNNNNNNNNNNNNNNNNNNNNNNNNNNNNNNNNNNNNNNNNNNNNNNNNNNNNNNNNNNNNNNNNNNNNNNNNNNNNNNNNNNNNNNNNNNNNNNNNNNNNNNNNNNNNNNNNNNNNNNNNNNNNNNNNNNNNNNNNNNNNNNNNNNNNNNNNNNNNNNNNNNNNNNNNNNNNNNNNNNNNNNNNNNNNNNNNNNNNNNNNNNNNNNNNNNNNNNNNNNNNNNNNNNNNNNNNNNNNNNNNNNNNNNNNNNNNNNNNNNNNNNNNNNNNNNNNNNNNNNNNNNNNNNNNNNNNNNNNNACACACACACACACACGCACACACACACACACACACACACACCACTACAGTAATCCTCTGTAGCCCTCTCAAGCCCTTCATGAATGGACGTGCCACACCGGGCCTTGTAGAGAACAGGTGCTCGACCTCAGTGACCTTTGAGTGCCACCCAGGGTACATCTTAAATGGCCCCACCTCCACCAGGTGTGCTTCAAATGGGACGTGGGACCAACCTCAGCCAACGTGCCAAGGTGGGGTCTATCCTTCAATGCCTACATAAGGTGTACCCACTACATGTGTGCGATCGTGTAGGGGTCCGGGGGAGTGGGGGGAGTGGTTTCATTAACATGTCAACAAACTATGCTGGTGTGTACAACACTTGGCCAGCAGGACAAGACAGGGAGGTGAATGATCCGCAGTAATGTGGTTGGCTCTTTGTTTGTAAACAATGTGTTTTCCAAGGCATTGGTGAAGAGTATTGGAAAGCTNCACCCATTATGATGTGATCAAAGATAAGAATGGCATCGTTTTCATTTCTTGTGTTGGTATCAGGCAGGAGACAGTGGGGAGANGAGGGAAGGAGANGGGAGGGAANGGAAGGAGAGGAAAGGTGAGGAGAGGGAGAGCTAGGGAAGAAAAGGGAGGCTCCTTGAAGGGGTTGGATTGCTCCCAGTCTACTCTCTCAATGTCTTATCCTTACCCCTTAGCTGTCGCATGTCCTCAGATCCCAGCACCCAAGAGCAATAGCTTCATCTGTACAGCCACCCCCCCTGCTCCCCAGTTTGGGGCAACCATCAAGTACTGCTGTGCAGAGGGTCATCAAGTGGTGGGGGGAGGAGGCTCAGACTATGTGACCTGCACAGAAGATGGAGTATGGAGTGCCTCTGCTCCTGTGTGCCAAGGTGACAGTGTGGGGTATGGTGATAAGTGTGGGGTATGGTGATAAGTGTGGGGTATGGTGATAGGAGTGTGCCAGATGATGGGNNNNNNNNNNNNNNNNNNNNNNNNNNNNNNNNNNNNNNNNNNNNNNNNNNNNNNNNNNNNNNNNNNNNNNNNNNNNNNNNNNNNNNNNNNNNNNNNNNNNNNNNNNNNNNNNNNNNNNNNNNNNNNNNNNNNNNNNNNNNNNNNNNNNNNNNNNNNNNNNNNNNNNNNNNNNNNNNNNNNNNNNNNNNNNNNNNNNNNNNNNNNNNNNNNNNNNNNNNNNNNNNNNNNNNNNNNNNNNNNNNNNNNNNNNNNNNNNNNNNNNNNNNNNNNNNNNNNNNNNNNNNNNNNNNNNNNNNNNNNNNNNNNNNNNNNNNNNNNNNNNNNNNNNNNNNNNNNNNNNNNNNNNNNNNNNNNNNNNNNNNNNNNNNNNNNNNNNNNNNNNNNNNNNNNNNNNNNNNNNNNNNNNNNNNNNNNNNNNNNNNNNNNNNNNNNNNNNNNNNNNNNNNNNNNNNNNNNNNNNNNNNNNNNNNNNNNNNNNNNNNNNNNNNNNNNNNNNNNNNNNNNNNNNNNNNNNNNNNNNNNNNNNNNNNNNNNNNNNNNNNNNNNNNNNNNNNNNNNNNNNNNNNNNNNNNNNNNNNNNNNNNNNNNNNNNNNNNNNNNNNNNNNNNNNNNNNNNNNNNNNNNNNNNNNNNNNNNNNNNNNNNNNNNNNNNNNNNNNNNNNNNNNNNNNNNNNNNNNNNNNNNNNNNNNNNNNNNNNNNNNNNNNNNNNNNNNNNNNNNNNNNNNNNNNNNNNNNNNNNNNNNNNNNNNNNNNNNNNNNNNNNNNNNNNNNNNNNNNNNNNNNNNNNNNNNNNNNNNNNNNNNNNNNNNNNNNNNNNNNNNNNNNNNNNNNNNNNNNNNNNNNNNNNNNNNNNNNNNNNNNNNNNNNNNNNNNNNNNNNNNNNNNNNNNNNNNNNNNNNNNNNNNNNNNNNNNNNNNNNNNNNNNNNNNNNNNNNNNNNNNNNNNNNNNNNNNNNNNNNNNNNNNNNNNNNNNNNNNNNNNNNTCCAACACAACTGCACTACAAGGAATTGCGTAGTCACTTCAAAAACTGTGTTGACACATACAGCATAACCCCATTTCNTTCCACTGTTGTAAGCCGTCGGCCACGTGTTGTACATGCAATAATTGTGCGAATCGAGCTGGAAATGNCCTTAACTTTGCACACCTGTATCTCCGTGAAGGATAAAATAAANTTTAAGGAACAGAACACATCAATAGCAACAAAACNCCTTTATCTATACACTGAATTTGATGCGATTTGAACGTTCCTACCTGAAGTTGTGGAGCTTTTGAACCCCATACAGTATCGGACCTCGTAACGGCGGTACCGTGGTTGTGCAAGACACCAAAGTACATTTTCACACATAGACAGTATGTTGTATGACGTGTTGCAAATAGGAAGCCTGTAGTGTTAACCGTTCAGAAGTTATAGGGATTCAAACGTGATGCATTTTTATAACGTTTTATAATATAATTATCCGTGATTCATTTTTCACGGAAATGGTCCCATATATATTACTATAACCAGCCACGTACCAATACATAGCTCGAGATTATTTGATCACAAAAGCGCAAATGCCGACCAATTCTGAAGCTATTGGCGCAATTTTCATTCTTTAAAAGTGTGAGTTATGACATAGTACATTGCTTCGGCAGTACATATACTAAAATTGGAACGATACAGAGAAGATTAGCATGGCCCCTGCGCAAGGATGACACGCAAATTCGTGAAGCGTTCCAAATTTTTTTTAATTTTTGTTTGTCCCTAAACTTTTCTGCCACAGCGATAATGATACAATGGCATGACACATTTTTGCTGAAACAAAAGCATGTACTGACAGTGTAGCTATTTTCCTTTGAAAATAAGATTGTCATCCATCCAACTAGTATATTTAGAATCTCCTAATCTCGTTCTTACCACAGGGATAGCGTGTCCTCCCCCACCCTTTGTTCCAAACCTCCTGATGAACCCTCCAGGGTCACTCTACTTTTGCGACACTACTGTCCAGTTTAGCTGTAGTTATGGCTACCAGATTGAAGGTGCTGTGACTAGACACCTGTNNNGGCAACGGGACATGGAGTGATGCCTTTCCCACATGCAAAGGTCAGCAGCAAACCTTTTGGCAAACACTACTTTCATGACTCTGAGAACAAAGTTGTCTTAAATATGGTTGACCATAGCTGTCTCATCCTCCCTGTTGTCTTCTTCCTCCCCTCTTTCGTTCCTCCCCTTCTTTCACTTTCTATTCCCCTCCCTCCTTTCACTCCTCCCTCTCCCTCCCATCCTTTCACTCTCATATCTCCCCTCTCTTCTCTCCCCCTCTTCCTCCTCCTCCAATTGTTCCTCAGCCGTTACATGTCCTCCATTGTCTTTGTCNAANNGANNTATNCGCACTGAACCTGGACCAATGAACATCAGCCATTTTGCATGTGACCCCTGCTTCANGTTGAATGGGCNTCCGGTCATACAGTGTACCTCGAGTGGCACATGGAATGATACAATCCCCAGATGTGATTGTAAGTTGTGTATTTGTGTGCACCTTGTATGTGTGTGTACCTGTGTGTGTATGTGTGTGCCTATGCATGTATGTGTGTGTACCTGTGTGTGTATGTGTGTGCCTATGTGTGTATGTGTGTGTGCCTGTGTTTGTGTGTATGNNNGTGTNTGTNCNNGTGTGNANCATGTAAGTTGCCAAGGACCAAATGCATGGNTGCTTCTGAATTGTTAGTATTTGNATCCTACACATGTGTTNGTCATTATGCCACACCCCTGTGGGTGTGTTGTGGTATGTGTGTAGTGGTGTGTGTGTAGTGGTGTGTGTGTAGTGGTGTGTGTGTGTGTTTAGTGGTGTGTGTGGTGGAACATTCCCTGTAAATAGCAGTTACTGAGAATACAACNTTTGCATTGACCCTCACATGCTCCCACATCACACTCCACACACTCATACNTCTATGTCCTCATACTCCATCATCACACCCTCACATCCTCCCACTCCTCTTCACACTCACACCCTCACATTGACCACTTGCATCTCCATCATCACACTCAAATTCACCATGACCCTTACNCACACCTCTCACACACACATCCCTCACACACATACACATACACCTCACACACACATGTACACACACACACACACACACACACACACGCACNNNNNNNNNNNNNNNNNNNNNNNNNNNNNNNNNNNNNNNNNNNNNNNNNNNNNNNNNNNNNNNNNNNNNNNNNNNNNNNNNNNNNNNNNNNNNNNNNNNNNNNNNNNNNNNNNNNNNNNNNNNNNNNNNNNNNNNNNNNNNNNNNNNNNNNNNNNNNNNNNNNNNNNNNNNNNNNNNNNNNNNNNNNNNNNNNNNNNNNNNNNNNNNNNNNNNNNNNNNNNNNNNNNNNNNNNNNNNNNNNNNNNNNNNNNNNNNNNNNNNNNNNNNNNNNNNNNNNNNNNNNNNNNNNNNNNNNNNNNNNNNNNNNNNNNNNNNNNNNNNNNNNNNNNNNNNNNNNNNNNNNNNNNNNNNNNNNNNNNNNNNNNNNNNNNNNNNNNNNNNNNNNNNNNNNNNNNNNNNNNNNNNNNNNNNNNNNNNNNNNNNNNNNNNNNNNNNNNNNNNNNNNNNNNNNNNNNNNNNNNNNNNNNNNNNNNNNNNNNNNNNNNNNNNNNNNNNNNNNNNNNNNNNNNNNNNNNNNNNNNNNNNNNNNNNNNNNNNNNNNNNNNNNNNNNNNNNNNNNNNNNNNNNNNNNNNNNNNNNNNNNNNNNNNNNNNNNNNNNNNNNNNNNNNNNNNNNNNNNNNNNNNNNNNNNNNNNNNNNNNNNNNNNNNNNNNNNNNNNNNNNNNNNNNNNNNNNNNNNNNNNNNNNNNNNNNNNNNNNNNNNNNNNNNNNNNNNNNNNNNNNNNNNNNNNNNNNNNNNNNNNNNNNNNNNNNNNNNNNNNNNNNNNNNNNNNNNNNNNNNNNNNNNNNNNNNNNNNNNNNNNNNNNNNNNNNNNNNNNNNNNNNNNNNNNNNNNNNNNNNNNNNNNNNNNNNNNNNNNNNNNNNNNNNNNNNNNNNNNNNNNNNNNNNNNNNNNNNNNNNNNNNNNNNNNNNNNNNNNNNNNNNNNNNNNNNNNNNNNNNNNNNNNNNNNNNNNNNNNNNNNNNNNNNNNNNNNNNNNNNNNNNNNNNNNNNNNNNNNNNNNNNNNNNNNNNNNNNNNNNNNNNNNNNNNNNNNNNNNNNNNNNNNNNNNNNNNNNNNNNNNNNNNNNNNNNNNNNNNNNNNNNNNNNNNNNNNNNNNNNNNNNNNNNNNNNNNNNNNNNNNNNNNNNNNNNNNNNNNNNNNNNNNNNNNNNNNNNNNNNNNNNNNNNNNNNNNNNNNNNNNNNNNNNNNNNNNNNNNNNNNNNNNNNNNNNNNNNNNNNNNNNNNNNNNNNNNNNNNNNNNNNNNNNNNNNNNNNNNNNNNNNNNNNNNNNNNNNNNNNNNNNNNNNNNNNNNNNNNNNNNNNNNNNNNNNNNNNNNNNNNNNNNNNNNNNNNNNNNNNNNNNNNNNNNNNNNNNNNNNNNNNNNNNNNNNNNNNNNNNNNNNNNNNNNNNNNNNNNNNNNNNNNNNNNNNNNNNNNNNNNNNNNNNNNNNNNNNNNNNNNNNNNNNNNNNNNNNNNNNNNNNNNNNNNNNNNNNNNNNNNNNNNNNNNNNNNNNNNNNNNNNNNNNNNNNNNNNNNNNNNNNNNNNNNNNNNNNNNNNNNNNNNNNNNNNNNNNNNNNNNNNNNNNNNNNNNNNNNNNNNNNNNNNNNNNNNNNNNNNNNNNNNNNNNNNNNNNNNNNNNNNNNNNNNNNNNNNNNNNNNNNNNNNNNNNNNNNNNNNNNNNNNNNNNNNNNNNNNNNNNNNNNNNNNNNNNNNNNNNNNNNNNNNNNNNNNNNNNNNNNNNNNNNNNNNNNNNNNNNNNNNNNNNNNNNNNNNNNNNNNNNNNNNNNNNNNNNNNNNNNNNNNNNNNNNNNNNNNNNNNNNNNNNNNNNNNNNNNNNNNNNNNNNNNNNNNNNNNNNNNNNNNNNNNNNNNNNNNNNNNNNNNNNNNNNNNNNNNNNNNNNNNNNNNNNNNNNNNNNNNNNNNNNNNNNNNNNNNNNNNNNNNNNNNNNNNNNNNNNNNNNNNNNNNNNNNNNNNNNNNNNNNNNNNNNNNNNNNNNNNNNNNNNNNNNNNNNNNNNNNNNNNNNNNNNNNNNNNNNNNNNNNNNNNNNNNNNNNNNNNNNNNNNNNNNNNNNNNNNNNNNNNNNNNNNNNNNNNNNNNNNNNNNNNNNNNNNNNNNNNNNNNNNNNNNNNNNNNNNNNNNNNNNNNNNNNNNNNNNNNNNNNNNNNNNNNNNNNNNNNNNNNNNNNNNNNNNNNNNNNNNNNNNNNNNNNNNNNNNNNNNNNNNNNNNNNNNNNNNNNNNNNNNNNNNNNNNNNNNNNNNNNNNNNNNNNNNNNNNNNNNNNNNNNNNNNNNNNNNNNNNNNNNNNNNNNNNNNNNNNNNNNNNNNNNNNNNNNNNNNNNNNNNNNNNNNNNNNNNNNNNNNNNNNNNNNNNNNNNNNNNNNNNNNNNNNNNNNNNNNNNNNNNNNNNNNNNNNNNNNNNNNNNNNNNNNNNNNNNNNNNNNNNNNNNNNNNNNNNNNNNNNNNNNNNNNNNNNNNNNNNNNNNNNNNNNNNNNNNNNNNNNNNNNNNNNNNNNNNNNNNNNNNNNNNNNNNNNNNNNNNNNNNNNNNNNNNNNNNNNNNNNNNCACACACACACGCACACACACACACACACACACACACCATGCATNNNNNNNCTTGGGTTTTTTTTTTGGCTCCATCTCATATACCAGTGGTGTGCTCACTTACACTTTGGCACTGTCACTGTCTCTTGTTCTATTGGATGCCTGTTAACCATGAGCATATTGAATACATGCTATCTTTGCTGTTGTGTTTTCAATGCTCACATATTTACCTATTCCAAAAAGCGGTGAAGTGCTTGCCGCTTCCAAGCCCAGAACACGGCTCAACGCAGCCCCAGTCCTGTTCCAACAGTTCCTACCTCTCAAGCTGCTTGTTCTCATGTGACGAGGGATACCGTTTGGTGGGCAATGGCCTCCTTACCTGTGCTCGGAANGGCATCTGGAGCAGCCTCCCTCCCAGATGCAATGGTGCGTCACTTTCACCTTACACAGCTGCTGCATGCCGCGTGCTCAAGCATGGCTCATACGAAGCAGGTGTCTCAAGTGATCTTCTTTGGCACCCCATGCAGTGATCAACTGTGGGCCCCCACAGCAGGCTGCTAGTCTGAAGGTGCAGTACTCGACTACTGAGTTCGGAGCATCTGCACTCTACCAGTGTGGGGAGTGCTTCAGGAGTGTGGGAGGGAACGTAACCACGGTGAAACACTGTGATGCGTCTGGGGAGTGGAGGGGCTCCTCACCCATATGCGAAGGTATGGACAGCACAACCTTCTTTAAATGGTTCACTCCCCTGTTTCCTTATGCATGAAGTTGAAGTGCTTTCATGGGAGGGCTTGCCTTGTACCACTTGACCTATGCTTTTTCCTTGTGTGTGTGTGTGCATGTGTGTGTGTATGTGTGTGTGTGTGTGTATGTTGCATGTGCAGCCGTGCAGTGTGTTCCAATTGTTGCACCCCTCAATGGGGGGGTCAGCCAGACGTCAAGCTTGTGTGGCAGCTCCGTGAGGTTCTACTGCAACGAGGGTTATCTCCTGGGTNNNNNTNNNGGNTNCATGTGTGCCTGATGAGTGGGCAATGGAGCAATCCTGTACCCATCTGCATGTGTAAGTTACACACACACACACACACACACACACACAnTGATGATGGTGATGATGGTGATGATGATGGTGATGATGGGATGACGATGATGGTGATGATGACGATGATGAACAAAGGTGATGACGGTGATGATGGTGATGTGGTAGGGTGACCATTTCTTTGCAATGATCAATGATGGAGACTTGATGATATNTGTAATCAGTAGCAAGACACCACTGCACATAATCACTGAGCGCAAAGCTCAATCATTACATAACATCCCGAGTTATTAAAATCGACATGAGAATAGCTGAATTATTTTTTTACTGATTGGCACACCCTGTCAGAAGTTAGTGGTCCTCTGTTCCTGGTTGCTTCCACCCCTCCCCTCAAATTACATCANNGTNAAATAAATAATCCATGTGAACAGAGCCTGTACTGTGTACATGAATCTCTGTATCATTGCTAGTTACCTCAAACAACCGACAGTCATCCCGTTTTCTTAACTCATCAACCAGCCAATGGGATTTGGTGCTGGCTATTGGGATTGTTGCCAGCACAACACAGCGGCAGCTGTCAAAAAGCGCCCTCACAGCCTCTACAAAGTCACTGCTGATAACCTCCATTTTGCCCACCTCGTCCACCACAACGAGGCGCCCCCCCCNNNNNNCNNNNNGGTCGATACGCATGGCAGGAAGCGCTAATTTCTCGAACGAAGCCACATCGACTTGGTATTTGCCAACACAGGCACGTCTGTGTTGTGGAGCAGACCCGTGTGCACCGGCGCTGATATGAAGTGCGTGAAAGATGGAAAAATAAATGCTAAGTTTGTTTCCTTTAAGAACCTGCTGGTATGTCAGACCTCCCTATTCTAGCCAAGGGACCTCTGTCTCCATTAAACGTCACCACATCAAAGCCCACTCGTGTTCCTCTCCCTCGTCCCTCAGACTGCCGAACCTCTTCCGTATAAAATCCTTGGAGGCGCAAGCCATGCTCGCTTTTCAAGCACACTGCAACCCTTTTAACCAAAGTTGTTTTTCCAACACCTGCATTACGAATGGGGACCGTGCATTCACGTGCAACTTAAATTTTGCTCTCTACCTGGTGGGCCTGTGACAAGAGCATGAATATGACGCGCACNTGCGACATCGTCTTATTATGCTCAAAATTACTTGTGGGTANNNNGACACGTGCAGGGCGCGCCAAATGTTCTCAATACAGTATGGTGTTTCACGCCATTATTCCAGCGAGGTTCCTGTCACTTATGGCTCACATGGTCATAAACATAATGCTGTTTTGGGGGAGAGTAAGTCGACACTCACGCGTATCAAAGTTATTGGTGTCTCTATCCAATCAGGTTAACTTCGTGATTTTCCAAACAGGAGCAAAACATTCTCTCGTGTGTTTCGATAGCAGGAGCGGACTATAGTGCAAAAGATGCACAGTAAGCATCCATGGGACTTCTCACTCTCCCCTTCCCCAACTTTAAGGGTTCCCCGAACTTTCAGGTTGCTTGCGGCATTGTCTTGTTCGTTGTTCTGCACGGTGGCGGAGCTGCTTGGTTTTCTCTCAGGACTGTCGATGTTTACCGCGCTGCCTGCTCTGTTCTGTATCCTCATGATGATGGTGACATTACCCACCACCGTGTTCCCTTTATTCTTCCTTGACGCTCTTTGTAGCAATTGCAGCCCATGTCAGTGGTACGATAACTTCGTCGCTGTTTGTGGTGGGCAGTTGGAGCTGTGATACTTATTGGTATATACTTGGCACTTGTAGGTAAGCCAAGAATGAGACTCAATATGAATAGATTTCTAGTATATCATTCTTTCTTCCAGCATTCTGCCATTCCTTGTAGAAATTGGTGTGGTTTTGTTGACAGTGAAATGTAAGGTCCTTGTATGATGTAAACTTCTGGGGATGGTGGCAACTTGCGAGTCACACCTACAGCACAGTACTACTTGTGTTGCTCCACCATAGCAACTGATGTACATGTGTTTTATGATTAACAAGACTTTTTGTGTCTGCAAATCACAAAGATACTGTAATTGCATTTTTTGGCTGGCTTAATGTATAAGTATTGTCATGGAAACTATAGTCCAGCTGTGCATATATAAGAGCTTTTGTGCATGCATATTTTTCTAGTAAAAATAGATTTAACTGTTTAAACCGGCTCTGTGCGTTCAGAGGAGACACGACTATATGCATCAAGTGCAACTGTTGGTGCTTTTCTTTGTCGCCGTTAGTGGTACAGCCTCCCTGGTTGACGACACGTGCTTGGCGGAATTGGGGCGCCAGCGACAGAACGCCTCTCTAGCGGATTCTCCGCGCCCTTGGGCAGTTTTGCTTTGGGTCACGTGTGTGTCACGTCAAGCGGGTGCTTCGGCAGCGGCAGGCGCTTTGGTGCGTAACTGTGCGGGAGTGTTGGCCAAAGACAACTGGATCGTCACCACTGCCGACTGCGTTTCTTGCCCCGGGCGCGGGGAACAAGTTTCCGTCACTGCAGACGTTGGTGCTCTTAGCAACGATATACAGCGGGACCAGTTGGCGGGAAATAAGGTGCAACGCCTTAGCGTTGACAAAGTGGCTTTTCATCCGAACTACGCTCCAGGCGGGGCTCGCGGCAATGTTGCTTTGTTGCACCTGAGCGTTCGAATAAGCGAGGCGGCCAAGTTTGTCCTTCGTCTTGCCAACTGCCGCGACAATGCATCGTTTTCTCAGCTCACAGCGCAAAGCAGCGGGTGGGTATCAGCGTTGTCGGAACATCCGCCTGGAGCGGACCGACTGGTTTTACGGGATTCGTCCGTCAGCTTGTTGCCCAAGGACACGTGCAGCAGAGCGTTGGGCCCGTAAGCNANNNNNACCTGCTGTGCTCTGTAGGGGTAGACCCTTCCCTGCCTCCANCCAGTGAATGCCACCAGGCTGGTGGATTATTATCTAAGTGTGGAACAGTGTTATCCCAAGAAGGGAAGCCCCCTGGTCGTAGTGGGAAAGCCCGGCATTGTCCCGCGTTCTGATAATCCCACGACGATCATTGGCTGCGAATGGCAACTGCTAGGCCTTCTGTCGTTGGGTGCCGAATGCAACACGACTGGTCCTAGCCTGTTCGTGAACTTATGCAGTTATGAGGCGTGGCTCGAGAATACGATCCAGACTGAAAACGGTGTGTCCTTTATAATTGCCACATGTTTGCAGGCATTGACACGTGGATCGTAGAATGAATTCACCATAACTCTCATCCCTTTATTCCCCAGCCCGCTTCGAGGTTTACTCCGCTTGCCCGTCTCCTCCCCAGGCCCCTGCTAACGGCTATCTGTGCTCTTCCAATGACCCTCTCAGCGGGATTCAATTCTGTTGCAACGATGGGTTCAGACTAGTCGGAGGTGCAATCTCACCTGTTTGTAACCTGGCCAGCAAGCGTTGGGAGCCTCTAGCACCACTCTGTGAACGTAAGGGGCTACATGGAACGACACACGCACACGCACGCACACACACACACACACACACANNNNNNNNNNNNNNNNNNNNNNNNNNNNNNNNNNNNNNNNNNNNNNNNNNNNNNNNNNNNNNNNNNNNNNNNNNNNNNNNNNNNNNNNNNNNNNNNNNNNNNNNNNNNNNNNNNNNNNNNNNNNNNNNNNNNNNNNNNNNNNNNNNNNNNNNNNNNNNNNNNNNNNNNNNNNNNNNNNNNNNNNNNNNNNNNNNNNNNNNNNNNNNNNNNNNNNNNNNNNNNNNNNNNNNNNNNNNNNNNNNNNNNNNNNNNNNNNNNNNNNNNNNNNNNNNNNNNNNNNNNNNNNNNNNNNNNNNNNNNNNNNNNNNNNNNNNNNNNNNNNNNNNNNNNNNNNNNNNNNNNNNNNNNNNNNNNNNNNNNNNNNNNNNNNNNNNNNNNNNNNNNNNNNNNNNNNNNNNNNNNNNNNNNNNNNNNNNNNNNNNNNNNNNNNNNNNNNNNNNNNNNNNNNNNNNNNNNNNNNNNNNNNNNNNNNNNNNNNNNNNNNNNNNNNNNNNNNNNNNNNNNNNNNNNNNNNNNNNNNNNNNNNNNNNNNNNNNNNNNNNNNNNNNNNNNNNNNNNNNNNNNNNNNNNNNNNNNNNNNNNNNNNNNNNNNNNNNNNNNNNNNNNNNNNNNNNNNNNNNNNNNNNNNNNNNNNNNNNNNNNNNNNNNNNNNNNNNNNNNNNNNNNNNNNNNNNNNNNNNNNNNNNNNNNNNNNNNNNNNNNNNNNNNNNNNNNNNNNNNNNNNNNNNNNNNNNNNNNNNNNNNNNNNNNNNNNNNNNNNNNNNNNNNNNNNNNNNNNNNNNNNNNNNNNNNNNNNNNNNNNNNNNNNNNNNNNNNNNNNNNNNNNNNNNNNNNNNNNNNNNNNNNNNNNNNNNNNNNNNNNNNNNNNNNNNNNNNNNNNNNNNNNNNNNNNNNNNNNNNNNNNNNNNNNNNNNNNNNNNNNNNNNNNNNNNNNNNNNNNNNNNNNNNNNNNNNNNNNNNNNNNNNNNNNNNNNNNNNNNNNNNNNNNNNNNNNNNNNNNNNNNNNNNNNNNNNNNNNNNNNNNNNNNNNNNNNNNNNNNNNNNNNNNNNNNNNNNNNNNNNNNNNNNNNNNNNNNNNNNNNNNNNNNNNNNNNNNNNNNNNNNNNNNNNNNNNNNNNNNNNNNNNNNNNNNNNNNNNNNNNNNNNNNNNNNNNNNNNNNNNNNNNNNNNNNNNNNNNNNNNNNNNNNNNNNNNNNNNNNNNNNNNNNNNNNNNNNNNNNNNNNNNNNNNNNNNNNNNNNNNNNNNNNNNNNNNNNNNNNNNNNNNNNNNNNNNNNNNNNNNNNNNNNNNNNNNNNNNNNNNNNNNNNNNNNNNNNNNNNNNNNNNNNNNNNNNNNNNNNNNNNNNNNNNNNNNNNNNNNNNNNNNNNNNNNNNNNNNNNNNNNNNNNNNNNNNNNNNNNNNNNNNNNNNNNNNNNNNNNNNNNNNNNNNNNNNNNNNNNNNNNNNNNNNNNNNNNNNNNNNNNNNNNNNNNNNNNNNNNNNNNNNNNNNNNNNNNNNNNNNNNNNNNNNNNNNNNNNNNNNNNNNNNNNNNNNNNNNNNNNNNNNNNNNNNNNNNNNNNNNNNNNNNNNNNNNNNNNNNNNNNNNNNNNNNNNNNNNNNNNNNNNNNNNNNNNNNNNNNNNNNNNNNNNNNNNNNNNNNNNNNNNNNNNNNNNNNNNNNNNNNNNNNNNNNNNNNNNNNNNNNNNNNNNNNNNNNNNNNNNNNNNNNNNNNNNNNNNNNNNNNNNNNNNNNNNNNNNNNNNNNNNNNNNNNNNNNNNNNNNNNNNNNNNNNNNNNNNNNNNNNNNNNNNNNNNNNNNNNNNNNNNNNNNNNNNNNNNNNNNNNNNNNNNNNNNNNNNNNNNNNNNNNNNNNNNNNNNNNNNNNNNNNNNNNNNNNNNNNNNNNNNNNNNNNNNNNNNNNNNNNNNNNNNNNNNNNNNNNNNNNNNNNNNNNNNNNNNNNNNNNNNNNNNNNNNNNNNNNNNNNNNNNNNNNNNNNNNNNNNNNNNNNNNNNNNNNNNNNNNNNNNNNNNNNNNNNNNNNNNNNNNNNNNNNNNNNNNNNNNNNNNNNNNNNNNNNNNNNNNNNNNNNNNNNNNNNNNNNNNNNNNNNNNNNNNNNNNNNNNNNNNNNNNNNNNNNNNNNNNNNNNNNNNNNNNNNNNNNNNNNNNNNNNNNNNNNNNNNNNNNNNNNNNNNNNNNNNNNNNNNNNNNNNNNNNNNNNNNNNNNNNNNNNNNNNNNNNNNNNNNNNNNNNNNNNNNNNNNNNNNNNNNNNNNNNNNNNNNNNNNNNNNNNNNNNNNNNNNNNNNNNNNNNNNNNNNNNACACACACACACACACACACACACACACACACACAACTTGTACTGGAGGGCTAATCTCATTGGACACTTCAATATTACCGGAAGGCTCACCATATTCTCTTGCTGTTCCTCCGTGTCCTCTTGTCCATCAGACTGTATTGGTCATAACTCCTCTTCCACAGATAATAGTGCTTGACCAGACTGCTAATTGGCTTGTCTGGCAGCTAAAGGGTTAAGGTGAAAGGCCCCGCCCCCAGTGTAAAGCATGAACAACAGGGCGACGGAGTTGTTGAGCTCAGGCAATAAGTAACAAGAATAGATGAATACCATTGCTCTGATGCGCTGGAAGTTCTTTCCATGGTAGTGAAATGCTTGCTCAAATGTGACTGTATCCTCCATATTCCATTCGTCTGGTTTAAGGGGGGGAAAGAAGGCAGGAGAGGGGAGAGAGGNNNNGGANNANNNANANNNNANNNNNANNGNANGGCNAGGGAGGGTGGGTAAGGAGAGATAGGAAGGAGATTGGGGAGGAGAGGGAAAAAGTCTATAGATGTAGCCTCCACACATGACATCATACATGTACCTTTTAAAGGGCAGAAGTTGGGAAGATCCTCCAATGCTTTGTCTATGCTGTACTTGTGCCACAGAAGCAACCCAAGGGCCTATGGGGGAACACCAGGCAAATGAAACTCCTTCCTCTCCTTTGTATTTGGCATGGACTGTCTTTGGTTATTCAATTTTGTGGGTCAAATATATTCATAGCAGCCATTCATAGTCACATGATCCACAGATAAGGTGCGCACTAATTAAAACACAACACATGTTTGGATAGTGCCTAAACACTCAAAGCCCATTGTTTAAAAACAAACTGGCAATCTAAATAAAGCACCTCTCCAGCACATTCAACGAGTGAAGGTGCTAAAGTGAAGGTGCTAAGCCAGTGACTCATGAGTATGCACCAATCAAAAGCTGCAGAGCTGAAAGTGACATAGAACATCATGTAAACCAACAAACGGGAGTATGTGTCAAGCTGGACACAGATATGAATAAATTATTACATTTCTATGAAAACATTTTCCCCTAAAAGGCACACAATGTGATGCATAAAGTTACATATCAAACACACACACACACACACACACACACACACACACACACACANNNNNNNNNNNNNNNNNNNNNNNNNNNNNNNNNNNNNNNNNNNNNNNNNNNNNNNNNNNNNNNNNNNNNNNNNNNNNNNNNNNNNNNNNNNNNNNNNNNNNNNNNNNNNNNNNNNNNNNNNNNNNNNNNNNNNNNNNNNNNNNNNNNNNNNNNNNNNNNNNNNNNNNNNNNNNNNNNNNNNNNNNNNNNNNNNNNNNNNNNNNNNNNNNNNNNNNNNNNNNNNNNNNNNNNNNNNNNNNNNNNNNNNNNNNNNNNNNNNNNNNNNNNNNNNNNNNNNNNNNNNNNNNNNNNNNNNNNNNNNNNNNNNNNNNNNNNNNNNNNNNNNNNNNNNNNNNNNNNNNNNNNNNNNNNNNNNNNNNNNNNNNNNNNNNNNNNNNNNNNNNNNNNNNNNNNNNNNNNNNNNNNNNNNNNNNNNNNNNNNNNNNNNNNNNNNNNNNNNNNNNNNNNNNNNNNNNNNNNNNNNNNNNNNNNNNNNNNNNNNNNNNNNNNNNNNNNNNNNNNNNNNNNNNNNNNNNNNNNNNNNNNNNNNNNNNNNNNNNNNNNNNNNNNNNNNNNNNNNNNNNNNNNNNNNNNNNNNNNNNNNNNNNNNNNNNNNNNNNNNNNNNNNNNNNNNNNNNNNNNNNNNNNNNNNNNNNNNNNNNNNNNNNNNNNNNNNNNNNNNNNNNNNNNNNNNNNNNNNNNNNNNNNNNNNNNNNNNNNNNNNNNNNNNNNNNNNNNNNNNNNNNNNNNNNNNNNNNNNNNNNNNNNNNNNNNNNNNNNNNNNNNNNNNNNNNNNNNNNNNNNNNNNNNNNNNNNNNNNNNNNNNNNNNNNNNNNNNNNNNNNNNNNNNNNNNNNNNNNNNNNNNNNNNNNNNNNNNNNNNNNNNNNNNNNNNNNNNNNNNNNNNNNNNNNNNNNNNNNNNNNNNNNNNNNNNNNNNNNNNNNNNNNNNNNNNNNNNNNNNNNNNNNNNNNNNNNNNNNNNNNNNNNNNNNNNNNNNNNNNNNNNNNNNNNNNNNNNNNNNNNNNNNNNNNNNNNNNNNNNNNNNNNNNNNNNNNNNNNNNNNNNNNNNNNNNNNNNNNNNNNNNNNNNNNNNNNNNNNNNNNNNNNNNNNNNNNNNNNNNNNNNNNNNNNNNNNNNNNNNNNNNNNNNNNNNNNNNNNNNNNNNNNNNNNNNNNNNNNNNNNNNNNNNNNNNNNNNNNNNNNNNNNNNNNNNNNNNNNNNNNNNNNNNNNNNNNNNNNNNNNNNNNNNNNNNNNNNNNNNNNNNNNNNNNNNNNNNNNNNNNNNNNNNNNNNNNNNNNNNNNNNNNNNNNNNNNNNNNNNNNNNNNNNNNNNNNNNNNNNNNNNNNNNNNNNNNNNNNNNNNNNNNNNNNNNNNNNNNNNNNNNNNNNNNNNNNNNNNNNNNNNNNNNNNNNNNNNNNNNNNNNNNNNNNNNNNNNNNNNNNNNNNNNNNNNNNNNNNNNNNNNNNNNNNNNNNNNNNNNNNNNNNNNNNNNNNNNNNNNNNNNNNNNNNNNNNNNNNNNNNNNNNNNNNNNNNNNNNNNNNNNNNNNNNNNNNNNNNNNNNNNNNNNNNNNNNNNNNNNNNNNNNGAGGGTGAGAGGAAGGAGGGAGGAGAGAGGGAGGGAGGAAGAGATGGGAGCAGCTGGGCAAAACAAACAAACCCATGATAAACCTTCTAATGCCCTTNCTGCATGCTTCTTTCTTCATGACATCATTCACATTACATCGTCTAAAACTAATTCCTAAGTGAAGGGGTGTGGTCACCTTGTCCTTGATATCCTGTGATTCTCTTTCCAATTGCTTCAAATAAAGCCCACCTGTGTCGTTGCACAGCTGGGTCAGTTCAGCTGGTGACAGGTGAATGCCCTGGAGAGACAGGTGTTGGTAGGGAACCATGAAGCAACTCTAAATAATGCACACACCAATGCAACATACGCCCATACCTTAGGGAGCTGTTTCCCTTTGCCGAAAAGTGGGGCAGAAGGGGGAGGTGCCCCACCCCCCTCAGAGTGGCTTGAACTTGAGTTATTACTGTCACTGTCATCATTTAGAGGCGGAAGGCCGGCACTGGGGAGGAGGAAGGTGAGGAAAGGGAAGATGGAGGAAGGGGGATGTTGGAGGGACATAAAAAGTAAACATATACATTTTGCTAAGATCAATAGTTTTTTTTTGTTGTGAGCCCAGCAACCACACATGGCAACCCCACACAACAGCTCTCTCCTAGGAAGGCATCTGTCTTATATGGTTACAGGGGTTGTTTTTACACTGATCCAGCACATCTTGCAAACACCTCCACACATTCCACTCCACACATTCCACTCCACACTTCCCAACACATCACTCCCTTCACAGATGATGTCATAAAACGGAGGTATGACACAACCTAACAAACAACATAATATCTACAGTCAATTACAGCGTTATAAATAGAGGCTTATTAATAAAATTTAAACACTAAATTGAATAGTGGTCTGGTTATACAATATGAATGCTCTCTGATGGATTATTAAATAATTATTAATAGCTATTGATAGTGGCATGCATAAAGTAAAATTGATTGGAGCAAATATTCCACAGCAATCATGACCTCCCTGCTGACTTGAGATCCCACAACTGTGACTAGCCCTCTCTCTGGTATCATGGGTGACTAGCCCTCTCTCTGGTATCATGGGTGACTAGCCCTCTCTCTGGTATCANGGGTGACTAGCCCTCTCTCTGGTATCATGGNNNNNNNNNNNNNNNNNNNNNNNNNNNNNNNNNNNNNNNNNNNNNNNNNNNNNNNNNNNNNNNNNNNNNNNNNNNNNNNNNNNNNNNNNNNNNNNNNNNNNNNNNNNNNNNNNNNNNNNNNNNNNNNNNNNNNNNNNNNNNNNNNNNNNNNNNNNNNNNNNNNNNNNNNNNNNNNNNNNNNNNNNNNNNNNNNNNNNNNNNNNNNNNNNNNNNNNNNNNNNNNNNNNNNNNNNNNNNNNNNNNNNNNNNNNNNNNNNNNNNNNNNNNNNNNNNNNNNNNNNNNNNNNNNNNNNNNNNNNNNNNNNNNNNNNNNNNNNNNNNNNNNNNNNNNNNNNNNNNNNNNNNNNNNNNNNNNNNNNNNNNNNNNNNNNNNNNNNNNNNNNNNNNNNNNNNNNNNNNNNNNNNNNCACTGAGCTTAGCCCTCACAGGTACAGAGTCAGTGTTCTCGTAGCTGGCTCCTCCCTTCTCCTTGTGGGTGGGGACTGTTTCAACATCCTGCTTAGAGATAATGTCATCACACATGACCCCTACAGGGGGCAGCCACACCCCCAGGGACAAGGCTAGATGATAGTCTGGGGATCGAGGGCTATATGGATAGTCTGGGGATGAGGGCTATATGGGGTGAGGGGCTAGATGATAGTCTGGGGAGGAGAGGCTTGATTAACGTTCTGGGGAGGAGGACCATTAAATTCCCCCAACCAATGGTACTGTGTACTGCCAGAGACAAATGTAGCAGCTACCACTGAACATGCAAACCTGGATATATAATCATCACTACTACATCACTATTACATCACTATTACATCACTATTACATCACTGCTAATACCCATTGCATGCACAGCTCAACTGAGGTGCTAGCCCTGCCCCTTACACACACACACACACACACACACACACACACACACACACANNNNNNNNNNNNNNNNNNNNNNNNNNNNNNNNNNNNNNNNNNNNNNNNNNNNNNNNNNNNNNNNNNNNNNNNNNNNNNNNNNNNNNNNNNNNNNNNNNNNNNNNNNNNNNNNNNNNNNNNNNNNNNNNNNNNNNNNNNNNNNNNNNNNNNNNNNNNNNNNNNNNNNNNNNNNNNNNNNNNNNNNNNNNNNNNNNNNNNNNNNNNNNNNNNNNNNNNNNNNNNNNNNNNNNNNNNNNNNNNNNNNNNNNNNNNNNNNNNNNNNNNNNNNNNNNNNNNNNNNNNNNNNNNNNNNNNNNNNNNNNNNNNNNNNNNNNNNNNNNNNNNNNNNNNNNNNNNNNNNNNNNNNNCACACACACACACACACNCANACACACACACACACACGCGAACACACACGAGCTAGCTATATACTTACCCTGGCATTCTTGCTGTAACGTGAGTGTTTCTGACCCTGTGCATTGAACAAAGTAAAACATCTCTAAATGCCTCTGTGCGTGGTTGTGTGCATGTGACAAGGAAAGCTCCCTTATCCTAGCTGTCAAGCCTTTCCCACCAGACAGCCACAAATACGATATGCTTGGATTCTTATTAATGCACATGTACCTATTTAGACCCTCAAATAATTGTGNTGCATAGTTTCAACGGATACACATGCATCATGTGACCTGCACATGACCCTCACATGTCTGATCTCATATTCAGCGATGATGCGAGGCAGGTTGAGCTTCCTCTTATAGTTGGTGAAGAAGTTCCTGCACTGTGCCACTGTCTTGCTCTCAAGGGCNGTTGCCATGGCTTCAAAGTCTTTGCCATGCTCATGGATTACTGAAGGAGAGGGAGGTGTGAGGAGGAAGGGAGGAGGAAGGGAAGAGGTAAGGAATGGCAACAACAGTGTAACTAACAGTTTACACAGGGCACAGGGTCTAACATGTACCTTCCAGAGCTATATGTATCTCCTCCTCTGTCCAACGCGATGGAGCTCCCACTTTCTTTGTGAACTACACAGTACAGACAGACAATTGTCACTCCATATCACACAACCTCCCTCGTANCANACAGCCTCCCTCTAGCACACAGCNTCCCTCTAGCACANANCCTCCCCCTCTAGCACANANCNTCCNTCTAGCACACANCCTCCCTCTAGCACACAGCCTCCCTCTAGCACACAACCTCCCTCTAGCACACAGCTTCCCTCTAGCACACACTCCAAGCACCCCCAGATGGCTTATCCCCTCCCTCCCCTCTCCACCACCATCAGAAGCTCCCTCCCCTCTCCACCACCATCAGAGGCTCCCTCCCCTCTCCACCACCATCAGAGGCTTCCTCCCCTCTCCACCACCATCACAGGCTCCCTCCCCAAAGAACAGTAGAGCTCACACACCTCACACACACTCATTTGATGGATAACAGTGTGCAAGTGCCCAGGCCAAACATATTAACATTCAANCCCACCTGCTCCCTGGAACACATCAATGCACACTTTATCAATTGATTGCAAACAATATAGTCACTTAACTAGCACAGATGGCAAACAATATAGTCTNCTTNANTAGCANNGATGNCAANCATTGCATAACCATACATAAGCTATAGGTGTAATACTGTTGAATTGCACACTCAGGCATCATGTGACCGTTGTCAGGGAGACATCGTACAAGTGGGTGCCCAAATAAGGTCAGTGGAGTGTAGGGTGGTATCAATGCATGTGTGGGTGTGTGTGCATGTGTGGGGTGTGTAGGTGTGTGTAGATCACATGCCCTCACATCCAGAGGGCGGAGCTGCTCAATGCCCTCAAAGCTTGAACACTTCTCCTTCAGATCAGCTAGTAGCTGTTTGTTCTTCTGTATCTGCACACAGGAGGTGGTGGAAAGATCTCATTACACAAGAATAACAGGTGTGTGCGCATGTGGAGGGAGGAGAGAGGGAGGGAGGAGAGAGGGAGGGAGNNNNNNNNNNNNNNNNNNNNNNNNNNNNNNNNNNNNNNNNNNNNNNNNNNNNNNNNNNNNNNNNNNNNNNNNNNNNNNNNNNNNNNNNNNNNNNNNNNNNNNNNNNNNNNNNNNNNNNNNNNNNNNNNNNNNNNNNNNNNNNNNNNNNNNNNNNNNNNNNNNNNNNNNNNNNNNNNNNNNNNNNNNNNNNNNNNNNNNNNNNNNNNNNNNNNNNNNNNNNNNNNNNNNNNNNNNNNNNNNNNNNNNNNNNNNNNNNNNNNNNNNNNNNNNNNNNNNNNNNNNNNNNNNNNNNNNNNNNNNNNNNNNNNNNNNNNNNNNNNNNNNNNNNNNNNNNNNNNNNNNNNNNNNNNNNNNNNNNNNNNNNNNNNNNNNNNNNNNNNNNNNNNNNNNNNNNNNNNNNNNNNNNNNNNNNNNNNNNNNNNNNNNNNNNNNNNNNNNNNNNNNNNNNNNNNNNNNNNNNNNNNNNNNNNNNNNNNNNNNNNNNNNNNNNNNNNNNNNNNNNNNNNNNNNNNNNNNNNNNNNNNNNNNNNNNNNNNNNNNNNNNNNNNNNNNNNNNNNNNNNNNNNNNNNNNNNNNNNNNNNNNNNNNNNNNNNNNNNNNNNNNNNNNNNNNNNNNNNNNNNNNNNNNNNNNNNNNNNNNNNNNNNNNNNNNNNNNNNNNNNNNNNNNNNNNNNNNNNNNNNNNNNNNNNNNNNNNNNNNNNNNNNNNNNNNNNNNNNNNNNNNNNNNNNGGATGAGATGAGGGGATGGGATGAGGGGATGAGATGAGGAGGGGATGAGATGAGGGGNTGAGATGAGGGGATGGGATGAGGGGATGGGATGAGATGATAATCTTGCTCTGTTATACATGAATACGTGAATGTGTACATTAATAAGTCCATGAAATGTTAAAGAACTAAATTCACAAAACCTAGGAAATCTAAAAGAGAGGCACTTGGTACAGGACTAGGCCTTACAGTCAGGTTAACCCTGACAGAATGGCTCTGCAGTTGGCTCTCACTAAGGCTAGGCCTAGGCAGCTGTATATTCCAAAGATGGTCTTGGTGGGTTGGTTTAGCCTGGTGGAAAGGCGACCAGCGAGCTTAGAAAAGGCCACCACTGCTGCAGCACCCCATAGGCGTAGGAAGCAGCATAAGATTGGGGGGGAGTGGCCAAGAGTTGGTGGTAAAGAAGAGAAAAGTGAAAC>scaffold4236|size40413CCACCACCACCACCACCACCACCACCACCACCACCTCCTCCAGCATGGCCGGTATTAACTGCAGAATGGCGGGCACCCTCACTTCCATCCAGCATGAGGCCGAGGTGGAGACCGGGAAGCCGGGGATGTGGGCCAAAGAGGAGACTCAATAGCTGTATGCACAATACTTGATCATTATTGTACTAAATTATCCAAGAGATGGTTATGGTGCATACGAAATGCGCAATATTATCCGTCAGTTTATGCTTGACATAGATGCATGCATGGCATTAAAGTGCCAGTGTTGGCTTTAAGAGACTGTTGGATTGATGTCTGATCGATCTGGAGAACAGGACAAAGAGACAGAATCCAAACGGACTCACAATAGTATATAAAGTGGGTAGTTCCCAATGCAGTGCTACAAGATGACGAGCAAGAGAAGAAGCGTGCGTATGAAGAGAGGGTGAGAGAAATCGAGCATGGCTCCTTTGCACCCCTCGTAGTCTCAATCTCAGGAGGTATGGGTCCGATAGCCACAACCGTGTACAAAAGAATGGCCTCGCTCATAGCTCAGTGAAAAATACAATAATCCATATATCTTTTTTGGCTAAGATGCAAGCTGAGTTTCTCCTTATTGCGATCTGCATAACACATGTGCATCAGAGGCTCGAGGTCCTCCTATCACAGACCAACAAACATGTTTAGTGAGACCATTGTCCTATCCTGCTTGGTAAACAGGATCTCAATTTGAACATTGAAATCCTATCCGTCTTTTTTCTTTTATTNNNTNCTTANNTCATTACCACGTTTCCACGCATTATGTGTATATTTTTATTTAAAAAGCGTATACTTTACGGACTTATCTGCTGTACTGATTTGATTTATTGTCAGTTCAAAGCGATAGTCTCGTACCCAGACCTGACGGACGTAAAAGCATGTTTGCACGTCAGCCGAGTCTGGGTANNNNNNNNNNGGGGGCGGGGCCTAAATATACTACGGCCTGTCCGGGCCATGTAAACTTAAAAGACGTTACGAAAGTGGAAAAAAAAACTTACGAAATAAGTAAATTAAAACATTCTCTATACGCGAAAGTTGGCTCTCCTCCTCTCTTCCTGCCACTCCTCNTACCCCCATTGTCATCTTGTAACCATTACTGCCGATACATACATGATCACTTGCACAAGATTTAGTCACTATAAACTTATTGTGTAATAACTATTGCATAAAGTAGTTAAACGTGGTTAATTTCGGGTTTCCTCCCTCATCTCAAGTCACATGTTCTCATGTTCACGCCACGAGCCAGGCAGAAAACAGCATTTTTATTCATTGTCATTATTTTTATTTTATTAATTTTAGATTANCCATTACAATAAATAAATACATACATAATCAATAAACATTTGAGCACATAATACTACAAACCTACCTACCTAGGACNCNAAGAGTCCTTATCAGTTATTGAAATGACGACGACNNAGCANNNNCNCNNACCAGGGCAATGCTTAGCCTTCCATATAGCCCAGACACTGCTGAAGACATGGGACAAGCAGTTCGAGTGGCGATCCATGTAGCGAGTCTGTCGAGGCACCTGCTGNTANGTGTAAGCTCAGCTGGGGAGTTGGCGTGACCAAAAGCCAAGCTGTGGCGTGGGGAGAAGACACAGAGGGAAACCGNNNNNNNNNNNNNNNNNNNNNNNNNNNNNNNNNNNNNNNNNNNNNNNNNNNNNNNNNNNNNNNNNNNNNNNNNNNNNNNNNNNNNNNNNNNNNNNNNNNNNNNNNNNNNNNNNNNNNNNNNNNNNNNNNNNNNNNNNNNNNNNNNNNNNNNNNNNNNNNNNNNNNNNNNNNNNNNNNNNNNNNNNNNNNNNNNNNNNNNNNNNNNNNNNNNNNNNNNNNNNNNNNNNNNNNNNNNNNNNNNNNNNNNNNNNNNNNNNNNNNNNNNNNNNNNNNNNNNNNNNNNNNNNNNNNNNNNNNNNNNNNNNNNNNNNNNNNNNNNNNNNNNNNNNNNNNNNNNNNNNNNNNNNNNNNNNNNNNNNNNNNNNNNNNNNNNNNNNNNNNNNNNNNNNNNNNNNNNNNNNNNNNNNNNNNNNNNNNNNNNNNNNNNNNNNNNNNNNNNNNNNNNNNNNNNNNNNNNNNNNNNNNNNNNNNNNNNNNNNNNNNNNNNNNNNNNNNNNNNNNNNNNNNNNNNNNNNNNNNNNNNNNNNNNNNNNNNNNNNNNNNNNNNNNNNNNNNNNNNNNNNNNNNNNNNNNNNNNNNNNNNNNNNNNNNNNNNNNNNNNNNNNNNNNNNNNNNNNNNNNNNNNNNNNNNNNNNNNNNNNNNNNNNNNNNNNNNNNNNNNNNNNNNNNNNNNNNNNNNNNNNNNNNNNNNNNNNNNNNNNNNNNNNNNNNNNNNNNNNNNNNNNNNNNNNNNNNNNNNNNNNNNNNNNNNNNNNNNNNNNNNNNNNNNNNNNNNNNNNNNNNNNNNNNNNNNNNNNNNNNNNNNNNNNNNNNNNNNNNNNNNNNNNNNNNNNNNNNNNNNNNNNNNNNNNNNNNNNNNNNNNNNNNNNNNNNNNNNNNNNNNNNNNNNNNNNNNNNNNNNNNNNNNNNNNNNNNNNNNNNNNNNNNNNNNNNNNNNNNNNNNNNNNNNNNNNNNNNNNNNNNNNNNNNNNNNNNNNNNNNNNNNNNNNNNNNNNNNNNNNNNNNNNNNNNNNNNNNNNNNNNNNNNNNNNNNNNNNNNNNNNNNNNNNNNNNNNNNNNNNNNNNNNNNNNNNNNNNNNNNNNNNNNNNNNNNNNNNNNNNNNNNNNNNNNNNNNNNNNNNNNNNNNNNNNNNNNNNNNNNNNNNNNNNNNNNNNNNNNNNNNNNNNNNNNNNNNNNNNNNNNNNNNNNNNNNNNNNNNNNNNNNNNNNNNNNNNNNNNNNNNNNNNNNNNNNNNNNNNNNNNNNNNNNNNNNNNNNNNNNNNNNNNNNNNNNNNNNNNNNNNNNNNNNNNNNNNNNNNNNNNNNNNNNNNNNNNNNNNNNNNNNNNNNNNNNNNNNNNNNNNNNNNNNNNNNNNNNNNNNNNNNNNNNNNNNNNNNNNNNNNNNNNNNNNNNNNNNNNNNNNNNNNNNNNNNNNNNNNNNNNNNNNNNNNNNNNNNNNNNNNNNNNNNNNNNNNNNNNNNNNNNNNNNNNNNNNNNNNNNNNNNNNNNNNNNNNNNNNNNNNNNNNNNNNNNNNNNNNNNNNNNNNNNNNNNNNNNNNNNNNNNNNNNNNNNNNNNNNNNNNNNNNNNNNNNNNNNNNNNNNNNNNNNNNNNNNNNNNNNNNNNNNNNNNNNNNNNNNNNNNNNNNNNNNNNNNNNNNNNNNNNNNNNNNNNNNNNNNNNNNNNNNNNNNNNNNNNNNNNNNNNNNNNNNNNNNNNNNNNNNNNNNNNNNNNNNNNNNNNNNNNNNNNNNNNNNNNNNNNNNNNNNNNNNNNNNNNNNNNNNNNNNNNNNNNNNNNNNNNNNNNNNNNNNNNNNNNNNNNNNNNNNNNNNNNNNNNNNNNNNNNNNNNNNNNNNNNNNNNNNNNNNNNNNNNNNNNNNNNNNNNNNNNNNNNNNNNNNNNNNNNNNNNNNNNNNNNNNNNNNNNNNNNNNNNNNNNNNNNNNNNNNNNNNNNNNNNNNNNNNNNNNNNNNNNNNNNNNNNNNNNNNNNNNNNNNNNNNNNNNNNNNNNNNNNNNNNNNNNNNNNNNNNNNNNNNNNNNNNNNNNNNNNNNNNNNNNNNNNNNNNNNNNNNNNNNNNNNNNNNNNNNNNNNNNNNNNNNNNNNNNNNNNNNNNNNNNNNNNNNNNNNNNNNNNNNNNNNNNNNNNNNNNNNNNNNNNNNNNNNNNNNNNNNNNNNNNNNNNNNNNNNNNNNNNNNNNNNNNNNNNNNNNNNNNNNNNNNNNNNNNNNNNNNNNNNNNNNNNNNNNNNNNNNNNNNNNNNNNNNNNNNNNNNNNNNNNNNNNNNNNNNNNNNNNNNNNNNNNNNNNNNNNNNNNNNNNNNNNNNNNNNNNNNNNNNNNNNNNNNNNNNNNNNNNNNNNNNNNNNNNNNNNNNNNNNNNNNNNNNNNNNNNNNNNNNNNNNNNNNNNNNNNNNNNNNNNNNNNNNNNNNNNNNNNNNNNNNNNNNNNNNNNNNNNNNNNNNNNNNNNNNNNNNNNNNNNNNNNNNNNNNNNNNNNNNNNNNNNNNNNNNNNNNNNNNNNNNNNNNNNNNNNNNNNNNNNNNNNNNNNNNNNNNNNNNNNNNNNNNNNNNNNNNNNNNNNNNNNNNNNNNNNNNNNNNNNNNNNNNNNNNNNNNNNNNNNNNNNNNNNNNNNNNNNNNNNNNNNNNNNNNNNNNNNNNNNNNNNNNNNNNNNNNNNNNNNNNNNNNNNNNNNNNNNNNNNNNNNNNNNNNNNNNNNNNNNNNNNNNNNNNNNNNNNNNNNNNNNNNNNNNNNNNNNNNNNNNNNNNNNNNNNNNNNNNNNNNNNNNNNNNNNNNNNNNNNNNNNNNNNNNNNNNNNNNNNNNNNNNNNNNNNNNNNNNNNNNNNNNNNNNNNNNNNNNNNNNNNNNNNNNNNNNNNNNNNNNNNNNNNNNNNNNNNNNNNNNNNNNNNNNNNNNNNNNNNNNNNNNNNNNNNNNNNNNNNNNNNNNNNNNNNNNNNNNNNNNNNNNNNNNNNNNNNNNNNNNNNNNNNNNNNNNNNNNNNNNNNNNNNNNNNNNNNNNNNNNNNNNNNNNNNNNNNNNNNNNNNNNNNNNNNNNNNNNNNNNNNNNNNNNNNNNNNNNNNNNNNNNNNNNNNNNNNNNNNNNNNNNNNNNNNNNNNNNNNNNNNNNNNNNNNNNNNNNNNNNNNNNNNNNNNNNNNNNNNNNNNNNNNNNNNNNNNNNNNNNNNNNNNNNNNNNNNNNNNNNNNNNNNNNNNNNNNNNNNNNNNNNNNNNNNNNNNNNNNNNNNNNNNNNNNNNNNNNNNNNNNNNNNNNNNNNNNNNNNNNNNNNNNNNNNNNNNNNNNNNNNNNNNNNNNNNNNNNNNNNNNNNNNNNNNNNNNNNNNNNNNNNNNNNNNNNNNNNNNNNNNNNNNNNNNNNNNNNNNNNNNNNNNNNNNNNNNNNNNNNNNNNNNNNNNNNNNNNNNNNNNNNNNNNNNNNNNNNNNNNNNNNNNNNNNNNNNNNNNNNNNNNNNNNNNNNNNNNNNNNNNNNNNNNNNNNNNNNNNNNNNNNNNNNNNNNNNNNNNNNNNNNNNNNNNNNNNNNNNNNNNNNNNNNNNNNCCAGCCAATAACAGCAAGCACACGGGNCTNGNNTNNACNNGTNTATANATNNATGGCCACAAGCTGGCCAACCCAAGGCTGCATTGGAAGCAATAGGGGCAAACACAAGAGGGTGGGGCGTATATCAAAGGGGAGGCCAGTTGCAGAATGTGAACGGGGGAATTTCGAGAGCCTAAACAGTACCATAAGGGTTAGAAGCACTGAGTATGTACAGTGTATGTGTCAACGTTGCTCCATAGATAGTGTGGCCCAAAGGTGGAAGCGGCCAGTCCATACGCAGCACACACACACNCNNNCNNNNNNACNNANAGCAGCATCAGCTGCTCTACGCCTAAACACACTCACGCGACGCTCGGTTGCAATAAGCCGAAAGGATTTCTGTCCAGCGGCAGTGATGGAGGAGGAGTGACACATTTCTTCGTCGCTTTCAACAGACGCTTTAGATAAAAACAGAAAGCCTTGCTTTTTAGTTTAGGGTTCGACTGTTTTGTATACATCGACCACGGCTCCAAGAGCTCTTTAGTTCTGCAAAGATGGGCACACTTATGACTCCATTCACTCGGTCTAACCGACCGTCAAAATATATACAACGTACACAAAACTTACTTGAAAACCTCTTCCGACATGCTGAGACCTGCCCCCTCCTCCTCTGAATGATGTCAGCTTAGGTGCCTCGTTTGCAGGCTCAAATAAATTCATTGCTAAAGTCACGTGCGCAAACTATTTTTCNTTATATAGCTCACAATTAGATCATTTGTAACGCTTCTGTTCTCAAGTAAGCAAAAAATAAAAAAAGAAAGTTCTGTACCCAGACCCAAAAGTAGTAAGGAATGCAATCAGGACCAGTGGGTGTGGCATAAATTCTTTGCCCCCTCCCTATGGAAACGATCTATAGCTGCATGGGATCATGAATCATAAGGTATTCCAAGTATGTAGCNGCGTGAATGACTCTCCTGATATCAGATCCTGCAGACAGACTGAGTTGTACTGTGATCAAGTNGCATGCTTTCATATAATGCATGACCTAATGTGGTGCTCAGCAGGTTCATAACAGAGGGGCTGTGAAGTCTGACACTTCATATCCTTAACAATGGCAAATGAAATGAAACATAGATGGTTATGATAAATTTCTGCACATACATGAATATAAAAATACCCACTAACCACTCCATGCAATAAAGAACAGAAGAGAGGGTACAAAACGAAGGAAGAAACACCTGACACTCACAACATGTCACATGACAACAGCTACGCCGGCAGCTGCCCTGCAAGTCTCCATAGAACTGACCAGATCCCAGCGTGTTGTTTACAGGGTCATACAGTTCTGCTGTTTTCAAGTACGAAGGACCATTGATCCCTCCAACAGCCACCATATGTGCACCAATCGCACATAGNCAGACTCCATCGCGAGCTGAGGTCATTTTGGCTGCTGGATACCATTTGCCACTGCGAACATCGAGAAACTCCACAGAGTCCAGCCGTGAGGTTCCATCGTTACCACCNGCAACAAAAATCTGCTGACCCAAGACTGCCACCCCGATCCCCGCACGAGGTTTCCCCAGCGGGGGCAATGTTCTCCATCGGTTGACATGAGGATCGTAGCACTCCACAACGTTGAGAAACGAGGTCCCATCGTTCCCGCCAAATGTGTACAACTTGTCACCAAAGACAGCTACTCCCGGGAAACTTCTCCGTGAGCTCATCTGAGCGACAGTGACCCAAACGTTCTTTTCAAGGTCGAACCTTTCAACCGTGTTGAGACAGGAGCGACCGTCGTAGCCCCCCGCGGCGTACATGGCGCCTCCCATGACCCCCACGGCCACGCCCGAGCGACACGTGCTCAGGGGGGCCACGTACGTCCAGAACTGAGTGGCCGGATCGAAGCACTCCACGGTGCTGAGGCGCAGAGAGCCGTCCGACCCGCCCACCGCGTACACTTTGCCGTCGAGGACCCCGACGCCCAACTGTTGCCTACGAACCATGAGGGGCGTGCCCTCGCTCCACTTGTTTTCCAGCAGCGAGAACGTCTCGACGGAGTGAGTGATGCTTTCGGACGACTCCTTCCCGCCAACCGCGTACACGACGCCCACCGTGGACTTGCGCGGAAAGAAGCACGGATTGGCCGAACGGATGTCTCTGTCGGCAGGCATTAGGTGGTAGCGTTTCGCGTCGTCAAGAAGGTCCCGACACTCGATGCTCTGCTTCACGATGGGATCACGGTCAACGTCGTTCATCAAACAGTCGACGGCGAGGAGCNNNNNNNNNACGTGCTTCAGCAGCTTTGGCAGCTGTTGCCCGCGCTCGCCCGGGTCCTGCCTGACCCAGCTCATGACAGCTGCGTACACGACCTCTTCCCTGGGTACGTTCAGGTCGTTGCTTGAGATCAGCGAGGCGATACCTTCGGCCGAACTGTGGAAGAACTCGTTGGTGTTGGTCACCTCGAGGAAGTGCCGGCGAGCAAAAGAATCGACCACCTCCGACAGCTGGGTGCAGCCATTGACTTCGGCAAAATTCTTAACACCCAGGCAGTTGCTCACGTCCAAGTGGTTTTTTAGAAATTCGCAGCACACTTCCTGGGCCTGTTTTATCTGCAGCAATGAGGCAGCCGCTAACGTGTCTTGTACATTTTCCACCGTGACTGTTAAGATCCCTGTATAGGCAAAGTCAATCAAACTGCTCAGAACATCTCCACTCATGCCCTTGATTTGAACTACGTCCTGACCGCTCTCCAAAAGTTCATGAGTAAACATGGCCAGAAAGTAGGCACTGCAGGCAGACAGGATGGCTCTGTGGGCATTTATCTCTCTTCCTTCGGCACTCAACGTCACGTCACATAGCTTTTTGTGACTTCGAAGTAAGTGTACCTTTTGAAGAACGCTCTTGCTGTAGTGAAGTGTTGCTTCGTCGGCATCGAAATGTGATGATGGAGCCGTAGAACGATGTGAGTTTTCCTGACGAGATAGAAGCATTGATGTCTCTTTTTCTGCCATTCTCTTTCATTTCATCGTGTCAAATTGACGAATTGAGTCCAAAGTATGAAAATAGATACATTACGTTTCAAATTTTAAAATTACTTGCGATGTATTTTTAGAACAGTGCCGGATGGAATTGATGCAGAAGTCACGTCTAGAGGGACAACCGAGAATCAAATCGCTTATCTCCTTGTCGCTATCGAATGGCAACACAAAAATAAACAGTTTCACGTTGGTTTCATATCACAAAAGATGTGCTTTTAGAGCAAAAAAAAAATGTTTGTGGCAGTTTCTCGTGCATGATCAGTCCCTTGGTCGTCGTCTTCTTCTTCTTCTTCCTTCGTCACGTATAACTAATTAAATTTCTTTCAGCCCTGGTGACCCATTTGAGATCAACAGATCTGATGTACGACTGTACTTAAAAAACAAACAAAAAAACAAAAAAAAACACGCATACATACGTCGCTATTGAATTTATTTGGGAACTGTATCTTACCAGTAGGCCCTAAATCTGACAACTGTAGGTGAAAGAATGCAGCCATTCCCTTTCCTTCCCTTAGTCTCGTCCCCATATTTTATTCCTTTCGCCGTAGCGGTAATGTGCTGCGGTATATTGGGTCTGGGGACGAGACACTACTACCACCACCACCACCACCACCACCACCACCACCACCNNNNNNNNNNNNNNNNNNNNNNNNNNNNNNNNNNNNNNNNNNNNNNNNNNNNNNNNNNNNNNNNNNNNNNNNNNNNNNNNNNNNNNNNNNNNNNNNNNNNNNNNNNNNNNNNNNNNNNNNNNNNNNNNNNNNNNNNNNNNNNNNNNNNNNNNNNNNNNNNNNNNNNNNNNNNNNNNNNNNNNNNNNNNNNNNNNNNNNNNNNNNNNNNNNNNNNNNNNNNNNNNNNNNNNNNNNNNNNNNNNNNNNNNNNNNNNNNNNNNNNNNNNNNNNNNNNNNNNNNNNNNNNNNNNNNNNNNNNNNNNNNNNNNNNNNNNNNNNNNNNNNNNNNNNNNNNNNNNNNNNNNNNNNNNNNNNNNNNNNNNNNNNNNNNNNNNNNNNNNNNNNNNNNNNNNNNNNNNNNNNNNNNNNNNNNNNNNNNNNNNNNNNNNNNNNNNNNNNNNNNNNNNNNNNNNNNNNNNNNNNNNNNNNNNNNNNNNNNNNNNNNNNNNNNNNNNNNNNNNNNNNNNNNNNNNNNNNNNNNNNNNNNNNNNNNNNNNNNNNNNNNNNNNNNNNNNNNNNNNNNNNNNNNNNNNNNNNNNNNNNNNNNNNNNNNNNNNNNNNNNNNNNNNNNNNNNNNNNNNNNNNNNNNNNNNNNNNNNNNNNNNNNNNNNNNNNNNNNNNNNNNNNNNNNNNNNNNNNNNNNNNNNNNNNNNNNNNNNNNNNNNNNNNNNNNNNNNNNNNNNNNNNNNNNNNNNNNNNNNNNNNNNNNNNNNNNNNNNNNNNNNNNNNNNNNNNNNNNNNNNNNNNNNNNNNNNNNNNNNNNNNNNNNNNNNNNNNNNNNNNNNNNNNNNNNNNNNNNNNNNNNNNNNNNNNNNNNNNNNNNNNNNNNNNNNNNNNNNNNNNNNNNNNNNNNNNNNNNNNNNNNNNNNNNNNNNNNNNNNNNNNNNNNNNNNNNNNNNNNNNNNNNNNNNNNNNNNNNNNNNNNNNNNNNNNNNNNNNNNNNNNNNNNNNNNNNNNNNNNNNNNNNNNNNNNNNNNNNNNNNNNNNNNNNNNNNNNNNNNNNNNNNNNNNNNNNNNNNNNNNNNNNNNNNNNNNNNNNNNNNNNNNNNNNNNNNNNNNNNNNNNNNNNNNNNNNNNNNNNNNNNNNNNNNNNNNNNNNNNNNNNNNNNNNNNNNNNNNNNNNNNNNNNNNNNNNNNNNNNNNNNNNNNNNNNNNNNNNNNNNNNNNNNNNNNNNNNNNNNNNNNNNNNNNNNNNNNNNNNNNNNNNNNNNNNNNNNNNNNNNNNNNNNNNNNNNNNNNNNNNNNNNNNNNNNNNNNNNNNNNNNNNNNNNNNNNNNNNNNNNNNNNNNNNNNNNNNNNNNNNNNNNNNNNNNNNNNNNNNNNNNNNNNNNNNNNNNNNNNNNNNNNNNNNNNNNNNNNNNNNNNNNNNNNNNNNNNNNNNNNNNNNNNNNNNNNNNNNNNNNNNNNNNNNNNNNNNNNNNNNNNNNNNNNNNNNNNNNNNNNNNNNNNNNNNNNNNNNNNNNNNNNNNNNNNNNNNNNNNNNNNNNNNNNNNNNNNNNNNNNNNNNNNNNNNNNNNNNNNNNNNNNNNNNNNNNNNNNNNNNNNNNNNNNNNNNNNNNNNNNNNNNNNNNNNNNNNNNNNNNNNNNNNNNNNNNNNNNNNNNNNNNNNNNNNNNNNNNNNNNNNNNNNNNNNNNNNNNNNNNNNNNNNNNNNNNNNNNNNNNNNNNNNNNNNNNNNNNNNNNNNNNNNNNNNNNNNNNNNNNNNNNNNNNNNNNNNNNNNNNNNNNNNNNNNNNNNNNNNNNNNNNNNNNNNNNNNNNNNNNNNNNNNNNNNNNNNNNNNNNNNNNNNNNNNNNNNNNNNNNNNNNNNNNNNNNNNNNNNNNNNNNNNNNNNNNNNNNNNNNNNNNNNNNNNNNNNNNNNNNNNNNNNNNNNNNNNNNNNNNNNNNNNNNNNNNNNNNNNNNNNNNNNNNNNNNNNNNNNNNNNNNNNNNNNNNNNNNNNNNNNNNNNNNNNNNNNNNNNNNNNNNNNNNNNNNNNNNNNNNNNNNNNNNNNNNNNNNNNNNNNNNNNNNNNNNNNNNNNNNNNNNNNNNNNNNNNNNNNNNNNNNNNNNNNNNNNNNNNNNNNNNNNNNNNNNNNNNNNNNNNNNNNNNNNNNNNNNNNNNNNNNNNNNNNNNNNNNNNNNNNNNNNNNNNNNNNNNNNNNNNNNNNNNNNNNNNNNNNNNNNNNNNNNNNNNNNNNNNNNNNNNNNNNNNNNNNNNNNNNNNNNNNNNNNNNNNNNNNNNNNNNNNNNNNNNNNNNNNNNNNNNNNNNNNNNNNNNNNNNNNNNNNNNNNNNNNNNNNNNNNNNNNNNNNNNNNNNNNNNNNNNNNNNNNNNNNNNNNNNNNNNNNNNNNNNNNNNNNNNNNNNNNNNNNNNNNNNNNNNNNNNNNNNNNNNNNNNNNNNNNNNNNNNNNNNNNNNNNNNNNNNNNNNNNNNNNNNNNNNNNNNNNNNNNNNNNNNNNNNNNNNNNNNNNNNNNNNNNNNNNNNNNNNNNNNNNNNNNNNNNNNNNNNNNNNNNNNNNNNATGTGTGTGTGTNTGTGTNTGTGTGTGTGTNTNTGTGTGTGTGAGGATATGTGTAAGAGGAGAGTATTAAATCATGAGTGCATAAAAGCATACAATGGGTTAAAACAATGAGCTGTTAACAAATCATCAAATATACCAGATCTATTTATACAGGAGTACCACAAATATAGAAATGTAAGCAAATTAAACTAGTTACCATGTTCAATAGTTCCTGGAGGACCTCCATAGTAGGCGGGGTAAGAGAAGCCATTAATTTTTTCAAAACACTGTATTTTATGTGCTGTCTGAATGATGCTTTTATAGTTGACGATCCTTTCAGTGCCACAGTGAGAGTCCTTATCAGCAGACATACAAGGGTGGTTCCCAGACCACCAAGGGGAACATTNTTTAGCTCCTTGAAGGACTCCACCAGCTTCTCAAAACAGCCCACTTCTGCAAAGAGCTCCTGTGTGGTTTGGATAACAATGGGTGCTCATGGCTTGGTCGTGTGGCTTTACCTTCAACTGGGTGGAGCCCTTGGGAGCATCCATCAATAGTGAAATAGAGTCTGGAGGAGAGAACCAAGCAATAAGGTGCAAGAAAGGAAGTGAATGATGAGGGAGGTGAACAAGGGGGAGAGGGAGGGAACATAGGGGGAGAGGGAGGGAACAAGGGGGAGAGGGAGGGAACAAGGGGGAGAGAGGGAACAAGGGGGAGAGAGGGAACAAGGGGGAGAGAGGGAACAAGGGGGAGAGGGAGGGAACAAGGGGAGAGGGAGGGAACAAGGGGGAGAGAGAGAACAAGGGAGAAAGGGAGGGAACAAGGNNNANANNNANNNACACANNNGAGAGAGAGAGGGAACAATGAGGGAGGGGAACAAGGGGGAGAGAGGAAGGAGAGCAAGGATTAAAATACTCACTGAGTAAAAGAACGAAGAATGTAGGAAGAGCTTGTTGCTGTTCAGGTTTTAATTCAGAAATGCTTTATGCCAATACAACAACCAAATATTAAGAACAAATCTTCACAAANGAAGGTGGTAGTCACCTGTATGGCAGGACACTGAACAGCTTAGATAAAGTGACTCCAGTGTCCACCTCAACCTGTTCCTATGGTAACACCACAACATAAATATATGAACATCCCTCTCTTCTCATGCCCCACCCACTTGCCTCTTTGGGCATGAAACAATGGAGGACGTGTAACACTTTAATGAACACCTTTATGGTCAGTGCCTGCNNNNNNNNNNNNNNNNNNNNNNNNNNNNNNNNNNNNNNNNNNNNNNNNNNNNNNNNNNNNNNNNNNNNNNNNNNNNNNNNNNNNNNNNNNNNNNNNNNNNNNNNNNNNNNNNNNNNNNNNNNNNNNNNNNNNNNNNNNNNNNNNNNNNNNNNNNNNNNNNNNNNNNNNNNNNNNNNNNNNNNNNNNNNNNNNNNNNNNNNNNNNNNNNNNNNNNNNNNNNNNNNNNNNNNNNNNNNNNNNNNNNNNNNNNNNNNNNNNNNNNNNNNNNNNNNNNNNNNNNNNNNNNNNNNNNNNNNNNNNNNNNNNNNNNNNNNNNNNNNNNNNNNNNNNNNNNNNNNNNNNNNNNNNNNNNNNNNNNNNNNNNNNNNNNNNNNNNNNNNNNNNNNNNNNNNNNNNNNNNNNNNNNNNNNNNNNNNNNNNNNNNNNNNNNNNNNNNNNNNNNNNNNNNNNNNNNNNNNNNNNNNNNNNNNNNNNNNNNNNNNNNNNNNNNNNNNNNNNNNNNNNNNNNNNNNNNNNNNNNNNNNNNNNNNNNNNNNNNNNNNNNNNNNNNNNNNNNNNNNNNNNNNNNNNNNNNNNNNNNNNNNNNNNNNNNNNNNNNNNNNNNNNNNNNNNNNNNNNNNNNNNNNNNNNNNNNNNNNNNNNNNNNNNNNNNNNNNNNNNNNNNNNNNNNNNNNNNNNNNNNNNNNNNNNNNNNNNNNNNNNNNNNNNNNNNNNNNNNNNNNNNNNNNNNNNNNNNNNNNNNNNNNNNNNNNNNNNNNNNNNNNNNNNNNNNNNNNNNNNNNNNNNNNNNNNNNNNNNNNNNNNNNNNNNNNNNNNNNNNNNNNNNNNNNNNNNNNNNNNNNNNNNNNNNNNNNNNNNNNNNNNNNNNNNNNNNNNNNNNNNNNNNNNNNNNNNNNNNNNNNNNNNNNNNNNNNNNNNNNNNNNNNNNNNNNNNNNNNNNNNNNNNNNNNNNNNNNNNNNNNNNNNNNNNNNNNNNNNNNNNNNNNNNNNNNNNNNNNNNNNNNNNNNNNNNNNNNNNNNNNNNNNNNNNNNNNNNNNNNNNNNNNNNNNNNNNNNNNNNNNNNNNNNNNNNNNNNNNNNNNNNNNNNNNNNNNNNNNNNNNNNNNNNNNNNNNNNNNNNNNNNNNNNNNNNNNNNNNNNNNNNNNNNNNNNNNNNNNNNNNNNNNNNNNNNNNNNNNNNNNNNNNNNNNNNNNNNNNNNNNNNNNNNNNNNNNNNNNNNNNNNNNNNNNNNNNNNNNNNNNNNNNNNNNNNNNNNNNNNNNNNNNNNNNNNNNNNNNNNNNNNNNNNNNNNNNNNNNNNNNNNNNNNNNNNNNNNNNNNNNNNNNNNNNNNNNNNNNNNNNNNNNNNNNNNNNNNNNNNNNNNNNNNNNNNNNNNNNNNNNNNNNNNNNNNNNNNNNNNNNNNNNNNNNNNNNNNNNNNNNNNNNNNNNNNNNNNNNNNNNNNNNNNNNNNNNNNNNNNNNNNNNNNNNNNNNNNNNNNNNNNNNNNNNNNNNNNNNNNNNNNNNNNNNNNNNNNNNNNNNNNNNNNNNNNNNNNNNNNNNNNNNNNNNNNNNNNNNNNNNNNNNNNNNNNNNNNNNNNNNNNNNNNNNNNNNNNNNNNNNNNNNNNNNNNNNNNNNNNNNNNNNNNNNNNNNNNNNNNNNNNNNNNNNNNNNNNNNNNNNNNNNNNNNNNNNNNNNNNNNNNNNNNNNNNNNNNNNNNNNNNNNNNNNNNNNNNNNNNNNNNNNNNNNNNNNNNNNNNNNNNNNNNNNNNNNNNNNNNNNNNNNNNNNNNNNNNNNNNNNNNNNNNNNNNNNNNNNNNNNNNNNNNNNNNNNNNNNNNNNNNNNNNNNNNNNNNNNNNNNNNNNNNNNNNNNNNNNNNNNNNNNNNNNNNNNNNNNNNNNNNNNNNNNNNNNNNNNNNNNNNNNNNNNNNNNNNNNNNNNNNNNNNNNNNNNNNNNNNNNNNNNNNNNNNNNNNNNNNNNNNNNNNNNNNNNNNNNNNNNNNNNNNNNNNNNNNNNNNNNNNNNNNNNNNNNNNNNNNNNNNNNNNGAGGGAGGAGGGAGGGTTAGAGAACAAGGTGAAGAAACAAGAGGAAGACAAGTGGTGTCAGTGAAGGCTCATTGGAGTTAAACACATATGGAGGCTCATTGGAGTTAAACACATATGGAGGCTCATTGGAGTTAAACACATATGGAGGCTCATTGGAGTTAAACACANATGGAAGCTCAGTGGGGTTAAACACATACAAAGGCTCATTGGGGTTAAAAATTACCAGTTCCAAATTCTCGCCACACTTCACTGGTCGAAGGGAAACCAAAATGTCCTTGAGCTCAGTACCCATGATCGAGTGGGTGCCCAGTCCTTCAATCAATCTCAACAAATTGTCTGCAACCCAAAGAGAGTTGTTACACCCCACAGGGGGGGTGGGGCACACAGGGNNGNGNGGCNNACTAGGGCCGGGTGGCTAACTANNNNNNNNNNNNNNNNNNNNNNNNNNNNNNNNNNNNNNNNNNNNNNNNNNNNNNNNNNNNNNNNNNNNNNNNNNNNNNNNNNNNNNNNNNNNNNNNNNNNNNNNNNNNNNNNNNNNNNNNNNNNNNNNNNNNNNNNNNNNNNNNNNNNNNNNNNNNNNNNNNNNNNNNNNNNNNNNNNNNNNNNNNNNNNNNNNNNNNNNNNNNNNNNNNNNNNNNNNNNNNNNNNNNNNNNNNNNNNNNNNNNNNNNNNNNNNNNNNNNNNNNNNNNNNNNNNNNNNNNNNNNNNNNNNNNNNNNNNNNNNNNNNNNNNNNNNNNNNNNNNNNNNNNNNNNNNNNNNNNNNNNNNNNNNNNNNNNNNNNNNNNNNNNNNNNNNNNNNNNNNNNNNNNNNNNNNNNNNNNNNNNNNNNNNNNNNNNNNNNNNNNNNNNNNNNNNNNNNNNNNNNNNNNNNNNNNNNNNNNNNNNNNNNNNNNNNNNNNNNNNNNNNNNNNNNNNNNNNNNNNNNNNNNNNNNNNNNNNNNNNNNNNNNNNNNNNNNNNNNNNNNNNNNNNNNNNNNNNNNNNNNNNNNNNNNNNNNNNNNNNNNNNNNNNNNNNNNNNNNNNNNNNNNNNNNNNNNNNNNNNNNNNNNNNNNNNNNNNNNNNNNNNNNNNNNNNNNNNNNNNNNNNNNNNNNNNNNNNNNNNNNNNNNNNNNNNNNNNNNNNNNNNNNNNNNNNNNNNNNNNNNNNNNNNNNNNNNNNNNNNNNNNNNNNNNNNNNNNNNNNNNNNNNNNNNNNNNNNNNNNNNNNNNNNNNNNNNNNNNNNNNNNNNNNNNNNNNNNNNNNNNNNNNNNNNNNNNNNNNNNNNNNNNNNNNNNNNNNNNNNNNNNNNNNNNNNNNNNNNNNNNNNNNNNNNNNNNNNNNNNNNNNNNNNNNNNNNNNNNNNNNNNNNNNNNNNNNNNNNNNNNNNNNNNNNNNNNNNNNNNNNNNNNNNNNNNNNNNNNNNNNNNNTCAAAAGCATTGCGTTCTTTCGTGGACACCCGGTATACAGCACAACAGAGTAGCATTATTAAACATTAACTATTGAATGCCACTGCTCCTTATTTGCCAAGAGGTAATGAACACAAGGACACACCCATAATGAGTAACAGCATTTCTATGACATCATGACGTCAGTAATATCAGACAAGCCCTTTAACTCAATCCTGATTGCACGTAACAGTTTTAAGGCAGAACAGCACTGCCCTTCTGTCCAGTCAAACAGCCCTGTTTCCGTAGGACACAGCACAGAGACACTTACCAGTATTCCAAAATTCACTAGCTTTGGGTCGAGGTGGCTCAATGATGGAATATGCCTCCCTCTGTCTCAAGGGCAGTCCGGCCTGCTCCAGTATGGGGAAGAACACCTCCACTCCACCCAGGGAACTCAAAACCAACTGAAATGGGGGAGGTGGCGTCCATTGGCAGGGCTATGAGNTNTCCCCACCTTTCCTCCTCNCCCTCCCTCCCTTATCGATAATGTACAGGCTTGCCTTGACGTCTCTGATCACATAGACGTCTCCATNCAGTACTGCATCCACAGTCCCNTGCACAGCATGTGACGGAGAGAGGTCCACACACATCTTATCTTTGCATGCCTGTGCACGATGGGACAAGCTAGCTACAAGAGNACCACACTCACTGTTGCCATGACTACCTTCGGATGATAGTAAAAGATCAGCTTAGTCTTCCACTCGTCTGCCTTCGTTTGGAAGATNGACAAATCATTAGGTCCTAAGAATAGCAATTATAATTGATGCCCACCACTTCCCTCGTACCCAGTCCTTACCACGANAGTGGAGAAGCTGGACAGTGGTGTGGTTGAGTGNGTCAGAGAAGAGACACACAGGNCCAATGCAGCCCCCAAAGTTGGTCACAGAGCCAAAGGACGTGATACACTCATCCAGCCGCAAGGGGCTGATGATGTCATGAGGGGTGGGGCTCTCANGGGGAGAGGTGGAGCCCTCTGCGGAGAGCTGATTGTCTGTGCTGTCATGGCTCCAGCCAATCCTGCACTCTGTAAATGTCTGCATGGAGGAAGACATGAGGGAGGAGGAAGGCATGGGGAGGAGGAAGGCATGGGAGGAGGAAGACATGGGGAGGAGGAAGACATGGGGAGGAGGAAGACATGGGGAGGAGGAAGACATGGGGAGGAGGAAGGCGTGGGGAAGAGGAAGGCATGGGGAGGAGGAAGACATGGGGGAGGAGGAAGACATGGGGAGGAGGTNNNNCANTGGGGAGGAGGAAGNCATGGGGGAGGAGGAAGACATGGGGGAGGAGGAAGACATGGGGGGAGAAGGAGAACATGGAGGGAGGAGGAACGGGGGAGGAATGCAAAGGAAGGGATGATAATGGAAGTGTGATAAACCCAGTGTCTACCACTCACATTGGCAAGTGGTACAGCTCTGATCCCATCTGTTGCGGAGATTAGCCCATCCACGAGCACCTGTATGGTGACCTTGTCGCTGTGCTTCTTGCTACTGCTGTACACCACACACACTGAATGCTGGGCACCAGGTAGAGGTAGAGCANAACATAAATACACTCCACCCCTCCCCAATGACNAAAAGAACGCGTACCCATTTCTGTGGAGTGAGTGGCTCTGTGAGAGAAACAGTCTTAAGCCCCCTCTTCTTGGAGTGAACTGCCACCACCAGGACCAGGTCTTTGGTAAAGAACGCTTCAAACCCTGTACTACCAGCATAGAAACTGAAAGTAAGAGACCATTGGGTTATGATATGTAGCCCCTCCTTCTGCTCCACCCACCTGTAAAGCATTCTTCTCATGTTCCTTTGGTCCCCATCTGCACAACTTCGATCCAAACTCACCCAGCAGTGGAAGGAGAAGGCATTTTCTTTGGGCCAGCGATGTAGCTCTGAGATGGATATTTGCTGTANAGAGGATAAGATGATAAGGATGACACTAACACAGAGATAAGATGACAGAGATGACACTAACACAGAGATAAGATAAGGATGACACTAACACAGAGATAAGATAAGGATGACACTAANNNNNNNNNNNNNNNNNNNNNNNNNNNNNNNNNNNNNNNNNNNNNNNNNNNNNNNNNNNNNNNNNNNNNNNNNNNNNNNNNNNNNNNNNNNNNNNNNNNNNNNNNNNNNNNNNNNNNNNNNNNNNNNNNNNNNNNNNNNNNNNNNNNNNNNNNNNNNNNNNNNNNNNNNNNNNNNNNNNNNNNNNNNNNNNNNNNNNNNNNNNNNNNNNNNNNNNNNNNNNNNNNNNNNNNNNNNNNNNNNNNNNNNNNNNNNNNNNNNNNNNNNNNNNNNNNNNNNNNNNNNNNNNNNNNNNNNNNNNNNNNNNNNNNNNNNNNNNNNNNNNNNNNNNNNNNNNNNNNNNNNNNNNNNNNNNNNNNNNNNNNNNNNNNNNNNNNNNNNNNNNNNNNNNNNNNNNNNNNNNNNNNNNNNNNNNNNNNNNNNNNNNNNNNNNNNNNNNNNNNNNNNNNNNNNNNNNNNNNNNNNNNNNNNNNNNNNNNNNNNNNNNNNNNNNNNNNNNNNNNNNNNNNNNNNNNNNNNNNNNNNNNNNNNNNNNNNNNNNNNNNNNNNNNNNNNNNNNNNNNNNNNNNNNNNNNNNNNNNNNNNNNNNNNNNNNNNNNNNNNNNNNNNNNNNNNNNNNNNNNNNNNNNNNNNNNNNNNNNNNNNNNNNNNNNNNNNNNNNNNNNNNNNNNNNNNNNNNNNNNNNNNNNNNNNNNNNNNNNNNNNNNNNNNNNNNNNNNNNNNNNNNNNNNNNNNNNNNNNNNNNNNNNNNNNNNNNNNNNNNNNNNNNNNNNNNNNNNNNNNNNNNNNNNNNNNNNNNNNNNNNNNNNNNNNNNNNNNNNNNNNNNNNNNNNNNNNNNNNNNNNNNNNNNNNNNNNNNNNNNNNNNNNNNNNNNNNNNNNNNNNNNNNNNNNNNNNNNNNNNNNNNNNNNNNNNNNNNNNNNNNNNNNNNNNNNNNNNNNNNNNNNNNNNNNNNNNNNNNNNNNNNNNNNNNNNNNNNNNNNNNNNNNNNNNNNNNNNNNNNNNNNNNNNNNNNNNNNNNNNNNNNNNNNNNNNNNNNNNNNNNNNNNNNNNNNNNNNNNNNNNNNNNNNNNNNNNNNNNNNNNNNNNNNNNNNNNNNNNNNNNNNNNNNNNNNNNNNNNNNNNNNNNNNNNNNNNNNNNNNNNNNNNNNNNNNNNNNNNNNNNNNNNNNNNNNNNNNNNNNNNNNNNNNNNNNNNNNNNNNNNNNNNNNNNNNNNNNNNNNNNNNNNNNNNNNNNNNNNNNNNNNNNNNNNNNNNNNNNNNNNNNNNNNNNNNNNNNNNNNNNNNNNNNNNNNNNNNNNNNNNNNNNNNNNNNNNNNNNNNNNNNNNNNNNNNNNNNNNNNNNNNNNNNNNNNNNNNNNNNNNNNNNNNNNNNNNNNNNNNNNNNNNNNNNNNNNNNNNNNNNNNNNNNNNNNNNNNNNNNNNNNNNNNNNNNNNNNNNNNNNNNNNNNNNNNNNNNNNNNNNNNNNNNNNNNNNNNNNNNNNNNNNNNNNNNNNNNNNNNNNNNNNNNNNNNNNNNNNNNNNNNNNNNNNNNNNNNNNNNNNNNNNNNNNNNNNNNNNNNNNNNNNNNNNNNNNNNNNNNNNNNNNNNNNNNNNNNNNNNNNNNNNNNNNNNNNNNNNNNNNNNNNNNNNNNNNNNNNNNNNNNNNNNNNNNNNNNNNNNNNNNNNNNNNNNNNNNNNNNNNNNNNNNNNNNNNNNNNNNNNNNNNNNNNNNNNNNNNNNNNNNNNNNNNNNNNNNNNNNNNNNNNNNNNNNNNNNNNNNNNNNNNNNNNNNNNNNNNNNNNNNNNNNNNNNNNNNNNNNNNNNNNNNNNNNNNNNNNNNNNNNNNNNNNNNNNNNNNNNNNNNNNNNNNNNNNNNNNNNNNNNNNNNNNNNNNNNNNNNNNNNNNNNNNNNNNNNNNNNNNNNNNNNNNNNNNNNNNNNNNNNNNNNNNNNNNNNNNNNNNNNNNNNNNNNNNNNNNNNNNNNNNNNNNNNNNNNNNNNNNNNNNNNNNNNNNNNNNNNNNNNNNNNNNNNNNNNNNNNNNNNNNNNNNNNNNNNNNNNNNNNNNNNNNNNNNNNNNNNNNNNNNNNNNNNNNNNNNNNNNNNNNNNNNNNNNNNNNNNNNNNNNNNNNNNNNNNNNNNNNNNNNNNNNNNNNNNNNNNNNNNNNNNNNNNNNNNNNNNNNNNNNNNNNNNNNNNNNNNNNNNNNNNNNNNNNNNNNNNNNNNNNNNNNNNNNNNNNNNNNNNNNNNNNNNNNNNNNNNNNNNNNNNNNNNNNNNNNNNNNNNNNNNNNNNNNNNNNNNNNNNNNNNNNNNNNNNNNNNNNNNNNNNNNNNNNNNNNNNNNNNNNNNNNNNNNNNNNNNNNNNNNNNNNNNNNNNNNNNNNNNNNNNNNNNNNNNNNNNNNNNNNNNNNNNNNNNNNNNNNNNNNNNNNNNNNNNNNNNNNNNNNNNNNNNNNNNNNNNNNNNNNNNNNNNNNNNNNNNNNNNNNNNNNNNNNNNNNNNNNNNNNNNNNNNNNNNNNNNNNNNNNNNNNNNNNNNNNNNNNNNNNNNNNNNNNNNNNNNNNNNNNNNNNNNNNNNNNNNNNNNNNNNNNNNNNNNNNNNNNNNNNNNNNNNNNNNNNNNNNNNNNNNNNNNNNNNNNNNNNNNNNNNNNNNNNNNNNNNNNNNNNNNNNNNNNNNNNNNNNNNNNNNNNNNNNNNNNNNNNNNNNNNNNNNNNNNNNNNNNNNNNNNNNNNNNNNNNNNNNNNNNNNNNNNNNNNNNNNNNNNNNNNNNNNNNNNNNNNNNNNNNNNCACACACACACACACACACACATACACACACACACACTTTGAAANAGTCANATANAGAGGGAGTGGTGTGTGGCACACCTTCTGAAGGAGGGCTCCAATTGTAGCTGGTCCCCCAACGACAAAGAAGACCTGCTCATTGATTGATGTCTTGGTCAACATGGAGGTCATCAGTCGAATGAAGTATGTGACTGCCTCATGTTCTGGAAGTGTGCAAATAACAGGTGTGAACTCATGCTTGAGATGGCACTGCACGTAATGTCTGGAGAAGTCTTGATCAACAGACACAACAGTGGATTTCACCTCTAACGCTTACCATTATTCCAAGGTTCAAAGTTCAATGTGGTTCTGGGCACATGATTTATATTTATGGAACATATTTCCCTCTGTNTCAAAGGCAGTCCNGCCTGCTCCAGTATGGGGAAGAACACCTCCACTCCACCCAGGGAACTCAAAACCAACTGAAATGGGGGAGGTGGTGTCAATGAGATACTACTGTCACTCCCTTCCCTCACTCCCCCTTGGGATCAACTGGCTTGCCTGAACATCTCTGGTCACATAGACTTCACCAACCAGTACGCCCTCCATGGCTCCTTCCACTGTGTGTGATGGAGAGAGGTCCACACAGATGTTGNNCTTACAGGCCTGAAGGGAAGAAGAGACCATCAAAGATGGTGCCTAGAACAGAGGGGGATTTCCCCACCTTTGGGTGGAAGAACAGCACCAGTTTACTAATGAGTGCCTCCGTGTCAGGATACTTTGGTTGGAAAAGAGATAGGTTATTGGGTCCTGGCATATGTGGAAAAAGAAACAAAACAGGTCAATAAGCAGCATGTCTNCNNNNANNGATNNANTGGTNNCNNCTCCTACTTCCCCACCCANNNNNNNNNNNNNNNNNNNNNNNNNNNNNNNNNNNNNNNNNNNNNNNNNCCTCCTACTTCCCCACCCACTGTCCACTGTAACCCCTCCTACTTCCCCACCCACTGTCCACAGTAACCCCTCCTACTTCCCCACCCACTGTCCACTGTAACCCCTCCTACTTCCCCACCCACTGTCCACTGTAACCCTCCTACTTCCCCATTACATACACACCATTTCGATACAAAAGCTGTACAGCTTGCAGTTGCAACACTTCTGAGAAGATGTAGACAGAGCTGATCCTGCCAAACAGGCTAATGGCAGGACCAAAGGCTACGGGACTGTCCGCCATTGCCTCAAGCGATTCTGAAAGATCTGCCTCACGTTGGCAAAACCCAATATTGCAATGCTTGTAATCCTGGGGAGGAGGCAGTGTGCCAAGTGAGGATGTGGGGAGGAGAGGGAGAAGGTTGAGAGGAGAGTGAGGAGGTTGAGAGGAGAGGGAGAAGGTTGAGAGGAGAGGGAGGAGGTTGAGAGGAGAGGGAGGAGGTTGAGAGGAGAGGGAGGAGGTTGAGAGGAGAGGGAGGAGGTTGAGAGGAGAGGGAGGAGGTTGAGAGGAGAGGGAGGAAATTAAGAGGAGAGGNAGGAGGTTGAGAGGAGAGGGAGGAGGTTGAGAGNAGAGGGAGGAGGNNGNGTGGTTGAAAGGATTGGGAGGAGGAAGTGCAGGGCACACCAAGTAAAAGAGAGAAATCTGCATGCATGTGACCTTTACTTACGGCAGAAAGGTCTGGTGCTCTGAGATCAGTTGAAAAGGCTTTCTGGATTTCATCGATGTACACAGCAGTCTCATTTTTCCCACCCCAGGGTTTTTTAGAAGCGCTATGCGTCACACATACTGCATGCTAATGGAAGCTCACTGCATTTGATGCTATTGCAGCATGCCAGAACATCACCAGCGATGTCTTACCCATTTCTGAGGACTGAGTGGCTCTTTCACTGAGATGGTTTGAAACCCTCTCTTCTTGGAGTGAACTGCCACCACCAGGACCAGGTCTTTGGTAAAGAACGCTTCAAACCCTGTACTACCAGCATAGAAACTGAAAGAAATAGACCATTGGGTTATGATACGTAGCCCCTCCTTCTGCTCCACCCACCTGTAAAGCATTCTTCTCATGTTCCTCTGGTCCCCATCTGCACAACTTTGATCCAAACTCACCCAGCAGTGGAAGGAGAAGNCATACTGGTTTGGTTGCACGACTTCTGGAACAGCTATATGTATATGCTGTGCATNAAGTATTAGAGAGGAGGGAGAGGAGGGAGAGGAGGGAGAGGAGGGAGAGGAGGGAGAGGGAGGGGAGAGGGGAGGAGGAGGGAGAAGGGAGAGGAGGGGGAAGGGAGAGGAGGGAGGAGGGAGAAGAGGGAGAAGAGGAAGAAGAGGGGGAGGAGGGAGAGGAGGGGGAGGAGGAGGGAGAAGGGAGAGGAGGGGGAGAGGGANNNNNNNNNNNNNNNNNNNNNNNNNNNNNNNNNNNNNNNNNNNNNNNNNNNNNNNNNNNNNNNNNNNNNNNNNNNNNNNNNNNNNNNNNNNNNNNNNNNNNNNNNNNNNNNNNNNNNNNNNNNNNNNNNNNNNNNNNNNNNNNNNNNNNNNNNNNNNNNNNNNNNNNNNNNNNNNNNNNNNNNNNNNNNNNNNNNNNNNNNNNNNNNNNNNNNNNNNNNNNNNNNNNNNNNNNNNNNNNNNNNNNNNNNNNNNNNNNNNNNNNNNNNNNNNNNNNNNNNNNNNNNNNNNNNNNNNNNNNNNNNNNNNNNNNNNNNNNNNNNNNNNNNNNNNNNNNNNNNNNNNNNNNNNNNNNNNNNNNNNNNNNNNNNNNNNNNNNNNNNNNNNNNNNNNNNNNNNNNNNNNNNNNNNNNNNNNNNNNNNNNNNNNNNNNNNNNNNNNNNNNNNNNNNNNNNNNNNNNNNNNNNNNNNNNNNNNNNNNNNNNNNNNNNNNNNNNNNNNNNNNNNNNNNNNNNNNNNNNNNNNNNNNNNNNNNNNNNNNNNNNNNNNNNNNNNNNNNNNNNNNNNNNNNNNNNNNNNNNNNNNNNNNNNNNNNNNNNNNNNNNNNNNNNNNNNNNNNNNNNNNNNNNNNNNNNNNNNNNNNNNNNNNNNNNNNNNNNNNNNNNNNNNNNNNNNNNNNNNNNNNNNNNNNNNNNNNNNNNNNNNNNNNNNNNNNNNNNNNNNNNNNNNNNNNNNNNNNNNNNNNNNNNNNNNNNNNNNNNNNNNNNNNNNNNNNNNNNNNNNNNNNNNNNNNNNNNNNNNNNNNNNNNNNNNNNNNNNAGCCGGACGAGGTCAATCTCCCGACAACAGAGCATATTCTGATGGGTGGGTGTGGTTAGTAGGCCCAGAAGGTACTGCAACATCTCATCTCTCTGAGGGAGGAGAGGGAGGGAGGAGAGGGAGAAGTACTCACCAGAATTTCATCGTTCACTGCACACAGAAACCCTAGAATAGCATCCAATTCGTCACTGAAAATGAATTCTGCAAAGTACAGATCAACAACTGTGGAGAAACAGCAACAGAATCAGTCGATTGTCCCACCATCTGGTACAATGTACACAACACACCATCACATCTGGTACACAACACACAATCACATCTGGTACACAACACATCATCACAGCTGGTACACAGCACACCATCACATCAAGGCAACACAGCTTGGCATAACTACCGGAGATGACTCACTGGACATTAAGGACTTCCTTATATGGACTGTACTCAGCTTTGGATTAATAGCAGATGATGGACTACAAGGGAGGTCATACATTAATTGGAAGACTAGTTAGTAAATGCCCTACTTGTAATACTCTCTGATGATGTCTAAAACAAACTGAACTCCAAAATCTCTCCTACAGTCGTCCAGCTTCAGACTGACTAGGGTTGACAAGTACTTGCTGTGACCTGGGAATGGAAACACAAGAATGGGACAAGAACGGCAAGCATTGGAATGTGACATAAGTGATTGTACAGGTCACTGACTCAGTTGCACAGAGTAGTCTGCTCTGCTCCATATCCTGTAGTTGAACAGCAGTGACGAATAGAGATCAGTCTGCTGCTCTGGCTGGTCTGGACTGAGCTTCTCAACAAATTTTTCGATCGCACTGTGAGCTGCCACAGTCAACAGAGAAGGAGGGACCTGGGCACACACACATATACATATACATACACACATCACACACACACACACACACACACACACANACACACAACAAATTAACTGAATACACATGTTGCTCACCTTACATAGAACATGTAGTCAGGGGCATAAGCATCATTGGGATCAGGGTCCAGCTGGGTCATCAGATCAAATGCTGTCTTCTGGTAAAGCAGAGGAAACCTGTTGTGCATCTCCAACCCTACAGGATGGGATACAGGCCTATCAGNNNNNNNNNNNNNNNNNNNNNNNNNNNNNNNNNNNNNNNNNNNNNNNNNNNNNNNNNNNNNNNNNNNNNNNNNNNNNNNNNNNNNNNNNNNNNNNNNNNNNNNNNNNNNNNNNNNNNNNNNNNNNNNNNNNNNNNNNNNNNNNNNNNNNNNNNNNNNNNNNNNNNNNNNNNNNNNNNNNNNNNNNNNNNNNNNNNNNNNNNNNNNNNNNNNNNNNNNNNNNNNNNNNNNNNNNNNNNNNNNNNNNNNNNNNNNNNNNNNNNNNNNNNNNNNNNNNNNNNNNNNNNNNNNNNNNNNNNNNNNNNNNNNNNNNNNNNNNNNNNNNNNNNNNNNNNNNNNNNNNNNNNNNNNNNNNNNNNNNNNNNNNNNNNNNNNNNNNNNNNNNNNNNNNNNNNNNNNNNNNNNNNNNNNNNNNNNNNNNNNNNNNNNNNNNNNNNNNNNNNNNNNNNNNNNNNNNNNNNNNNNNNNNNNNNNNNNNNNNNNNNNNNNNNNNNNNNNNNNNNNNNNNNNNNNNNNNNNNNNNNNNNNNNNNNNNNNNNNNNNNNNNNNNNNNNNNNNNNNNNNNNNNNNNNNNNNNNNNNNNNNNNNNNNNNNNNNNNNNNNNNNNNNNNNNNNNNNNNNNNNNNNNNNNNNNNNNNNNNNNNNNNNNNNNNNNNNNNNNNNNNNNNNNNNNNNNNNNNNNNNNNNNNNNNNNNNNNNNNNNNNNNNNNNNNNNNNNNNNNNNNNNNNNNNNNNNNNNNNNNNNNNNNNNNNNNNNNNNNNNNNNNNNNNNNNNNNNNNNNNNNNNNNNNNNNNNNNNNNNNNNNNNNNNNNNNNNNNNNNNNNNNNNNNNNNNNNNNNNNNNNNNNNNNNNNNNNNNNNNNNNNNNNNNNNNNNNNNNNNNNNNNNNNGGGACATGTGTACCATCATGTACAACATCATGGACATGTGTACCATCATGTACAACATCATGGGACATGTGTACCATCATGTACAACATCATGGGACATGTGTACCATCATGTACAACATCATGGGACATGTGTACCATCATGTACAACATACCGGGACATTTTTTCAATACAGCTCACCTGTTCTTCCATGCTCCACACTCCCCTATAAGGAACCTTCGTGTGCCACTCCATCTCTGTAAAGTTGCTGATAACTCCTGCCTGTATTGATTGTCTAATGTCTTTTGTCGAGACAGCTCCAATTCCCGCTGTGAGTAAAACTGCTGCGATACTGTTATCTGATATTTTTCAAGATCTGAGAACACTATTTCTTTTCTTTTTGCTTCTGCTTTCAAGCAGTCTTGCTTGCATTGCCTAGTATGGTCCGATAAGACCTTCAACCATTTGTAAACTACCTCAGAGTATTGCATTTGTATAGCCTGAAGCTGTGTTAGAGTGTTGAGAGATGGGAGAGGGCACTCACCCGACAACCTGCTTACCTCACCCAGCCACGTATGCCACTCCTCCCTCTTGGAATATTCAACCAGCTCGATAGTCTCATTGGTGAGATCTGGTAGATAAGAAGGCAAAGGAACCAGCTCCTTATTCCGATACAATTGAAAAAGGTGCTGAAGTAGAGTAGCCCTTGTTGTAGGAGGTTGATCTGAACACACCCAGTATAGAAAATAGGAAGCAAATAGATTAGCATGTTGGAAGAAAAAGACGAGAGGGTAAGAAGGGATAAAGGGAGCAGAACAAAGGAGAAGGAACAGACAGAATCATTCACTGACCACTGCTGAACTGGGTTGCAATGCACCTGAGCACANANACTGNNTCNNGTGCTTCCAGGAATTGAAGTTCTTGCAGCTTACAACCATACTGACAATACTGTGGTACAAAGAGGCCCATAAGAAGGAGAGGGTGAGTAAGGAGAGGGGAAAGGAAGGGGAGGAGAGACAGAATGCATAAAGAGGAGACAGAGCATAGAGTACTAGTAAAATTGTACCACACTGAGATAGCACTGAGAAAACAATATCCACTCACTAAACACCCTCTTCCTCTTACTACTACCCAATCACCTCTGCATCATCAGAGCAAGCACTGGCCAATAAGATTCGCAGCACCGACTGCATCAGGGCTGGTTCTATGGGTCCTTCCAGCACATGAGCTGCTCTCAACATATTCCAGTATTGTTCCAGCAGCTGAAAGGAAAACATTCAAGCCATTGAGGTGTTAAGTTACACTAACATACCTGCATAGAGCAGTGTTCTTTGTTGAGCACCAAGACCCCATTATGAGAGAACTTTTGAAGAATCACTTCTAAATCTGTTGAATGTGAAAGATATCAGGTGTGCAGTAGGGTGGTGTGGTGTGTGCAAGAGTAGTCAGGTGTGCAGTAGGGTGGTGTGGTGTGTGCAAGATATCAGGTGTACAGTAGGATGTGTGGAAGATGTGTACCTTCTACAACATCAAACGAGGGATAGCTTGAGATGATGAAAGCTTCAAGCAGTTGGGATAATTGAAGGATGACAGAGGATAACATGTTTTCAGGAGACCAGCTTGGAACTTGGAAAACAAGGGACCATAGCAGTGATGAACCAATGATGGCCTCCAGCTCCCATGCCTTCTGGCCCAAATCTTTTCCCAATTCCTTCTCCTCTCTCTTCAGCCCCCTTCCAGCAACCACTCTCCTTACCTGAGGAACACTCCAAGGCAACAGCATTCATCAACAGCAAGAAGATTCTAACAAGCAAAGCAACATGAATGAAATATATATAACCATCTCCAGTCCAGTATAAAAAGACATCTGGACCACCCAACAACACAACAAGACATCTGGACCACCCAATAACACAACAAGACATCTGGACTGTACCTTTGTTTGATCAGGTGGACTGGCACTAACAGTCTCTGCTCTGTTGCAAAGAGGTCCAACGCTGCCATAAACTGACCACATACCTATGTGGGAGGGGCCAAAGTGAGGACACACCCACAGACCACACCTACTTTTCTAGTTTGTCCTTCCGACTGCTCTGAGATGATGCAATGCCACAGAATGTAACCAATGGTCTTCATGACAACATCATACGTTTCCGAAGTGGCTGATTCAGCTTCTGTCTGGTTGTCCACGTCCGATTTGCAGGGGGTCAGTAGCCATAGCAACAACCTCTCCCACCCTTTGGACTCTGCCAGCTTGGCCACGTGGCTGGAGTTAAGATGGAGGATGGAGATGAAATCCTGACAAATTGTGTGTCGAATGGACATGGAGGCTGTTCGAAGCAGTCTTAGAGTGGCCAGGTAGACATCAAAGTTGGAGATGCCGCCTGAAACAGAATACGCACATTGCACATGCTGTCTGGTCGGTGTGGTTATCACATTTATGCACATTGCACACATGTTTGTGTTGCCAGCCGTACATGCTCCATTGTTTGTATTACATGTCTACGGGCATTGTATGCTGCACACAGGCACTGTACGTATACTTCACTGCACACGGCACTGTATGCTGCACATGGGCACTGTACATATACTTCACTGCACATGGGCACTGTATNCTGCACATGGGCACTGTATGCTGCACATNGGCACTGTACATATACTTCACTGCACACAGGCACTGTACGTAGCACATACCTGTTTGGTTGTCACTCTTCTTGACCATGCAGAGGTCTAGAAGGGCCAAAATGATGGACGAGGGCAGACACTGCTCATTCTGACACATGGCAGCAGCAAAGGCCCCCGAGTGGAGGGTGTTGTACACCACACCACTCTCAACGCTCCCTTGCTTTAAATTGAGCTGGGTCAGAATCTGCAAGCGTGGGGCGTGAGCACATGGTTTCACCACGCCCCCTCAACAGACCTACCCTCCACCACGCCCCCTCAACACACCCCCTCAACAGACCTACCCTCCACNACAACCCCCTCAAAAGACCTACCCTCAGAGTTGTGAGTCGCACGCCAAAGTCTTGCGTCTGAATTAAAANGAGNAAGACCACACCCTTTTCCCAGAGGGCTGTTGAGAGCATATTTTGATGNGTGGGTGTGGTTAGTAGGCCCAGAAGGAATCGAAGAACCTCATCTAACTGAATAGAGGAGTGGTTTTTTAAAGGAAGAACACACACACACATACATGCACACAACACATACACGCACGCACGCACGCANNNNNNNNNNNNNNNNNNNNNNNNNNNNNNNNNNNNNNNNNNNNNNNNNNNNNNNNNNNNNNNNNNNNNNNNNNNNNNNNNNNNNNNNNNNNNNNNNNNNNNNNNNNNNNNNNNNNNNNNNNNNNNNNNNNNNNNNNNNNNNNNNNNNNNNNNNNNNNNNNNNNNNNNNNNNNNNNNNNNNNNNNNNNNNNNNNNNNNNNNNNNNNNNNNNNNNNNNNNNNNNNNNNNNNNNNNNNNNNNNNNNNNNNNNNNNNNNNNNNNNNNNNNNNNNNNNNNNTGTGTGTGTATGTGTATGTGTATGTGTGCATGTGTGTGTGTGCATGTGGTGGTTCACAGTCATATGTTCATGCATTATCAACAGTCGAGGGCTGGTTTTCTCCATACTTTGTCTGGACCTTTTCCTCCAGGTCAGGGTTCTGTATCCCCATGGGTTTGGACAGGTCTCTGAACACCTCAGAGTTGCTCAGGTCCAATGTATCACAACTGTAGTCTCGCAGGATCCATGGAAACTAAACACGGAGCAGATACTAATTGATAATTTGCATACAGCTCAAGGATCCTCACAATAGGATATTGGTTGAGGTCATTGTAGGTCCTTCCTGATATAGTGTTGAGTTGCATGATGTAATCAAAATTTGTAATTTCACGTTTCACCCATTTCTATAACAAAATAAACCACATCATTCCAAGTGGAGATGAAGAGGGTAAGGAGATGGAGGTCATGGAAGAGGGGAAGGAGATGGAAGGTCATGGAAGAGGGTAAGGAGATGGAAGGTCATGGAAGAGAGTAAGGAGATGGAGGTCATGGAAGAGAGTAAGGAGATGGAAGGTCATGGAAGAGGGGAAGGAGATGGAAGGTCATGGAANNNNNNNNNNNNNNNNNNNNNNNNNNNNNNNNNNNNNNNNNNNNNNNNNNNNNNNNNNNNNNNNNNNNNNNNNNNNNNNNNNNNNNNNNNNNNNNNNNNNNNNNNNNNNNNNNNNNNNNNNNNNNNNNNNNNNNNNNNNNNNNNNNNNNNNNNNNNNNNNNNNNNNNNNNNNNNNNNNNNNNNNNNNNNNNNNNNNNNNNNNNNNNNNNNNNNNNNNNNNNNNNNNNNNNNNNNNNNNNNNNNNNNNNNNNNNNNNNNNNNNNNNNNNNNNNNNNNNNNNNNNNNNNNNNNNNNNNNNNNNNNNNNNNNNNNNNNNNNNNNNNNNNNNNNNNNNNNNNNNNNNNNNNNNNNNNNNNNNNNNNNNNNNNNNNNNNNNNNNNNNNNNNNNNNNNNNNNNNNNNNNNNNNNNNNNNNNNNNNNNNNNNNNNNNNNNNNNNNNNNNNNNNNNNNNNNNNNNNNNNNNNNNNNNNNNNNNNNNNNNNNNNNNNNNNNNNNNNNNNNNNNNNNNNNNNNNNNNNNNNNNNNNNNNNNNNNNNNNNNNNNNNNNNNNNNNNNNNNNNNNNNNNNNNNNNNNNNNNNNNNNNNNNNNNNNNNNNNNNNNNNNNNNNNNNNNNNNNNNNNNNNNNNNNNNNNNNNNNNNNNNNNNNNNNNNNNNNNNNNNNNNNNNNNNNNNNNNNNNNNNNNNNNNNNNNNNNNNNNNNNNNNNNNNNNNNNNNNNNNNNNNNNNNNNNNNNNNNNNNNNNNNNNNNNNNNNNNNNNNNNNNNNNNNNNNNNNNNNNNNNNNNNNNNNNNNNNNNNNNNNNNNNNNNNNNNNNNNNNNNNNNNNNNNNNNNNNNNNNNNNNNNNNNNNNNNNNNNNNNNNNNNNNNNNNNNNNNNNNNNNNNNNNNNNNNNNNNNNNNNNNNNNNNNNNNNNNNNNNNNNNNNNNNNNNNNNNNNNNNNNNNNNNNNNNNNNNNNNNNNNNNNNNNNNNNNNNNNNNNNNNNNNNNNNNNNNNNNNNNNNNNNNNNNNNNNNNNNNNNNNNNNNNNNNNNNNNNNNNNNNNNNNNNNNNNNNNNNNNNNNNNNNNNNNNNNNNNNNNNNNNNNNNNNNNNNNNNNNNNNNNNNNNNNNNNNNNNNNNNNNNNNNNNNNNNNNNNNNNNNNNNNNNNNNNNNNNNNNNNNNNNNNNNNNNNNNNNNNNNNNNNNNNNNNNNNNNNNNNNNNNNNNNNNNNNNNNNNNNNNNNNNNNNNNNNNNNNNNNNNNNNNNNNNNNNNNNNNNNNNNNNNNNNNNNNNNNNNNNNNNNNNNNNNNNNNNNNNNNNNNNNNNNNNNNNNNNNNNNNNNNNNNNNNNNNNNNNNNNNNNNNNNNNNNNNNNNNNNNNNNNNNNNNNNNNNNNNNNNNNNNNNNNNNNNNNNNNNNNNNNNNNNNNNNNNNNNNNNNNNNNNNNNNNNNNNNNNNNNNNNNNNNNNNNNNNNNNNNNNNNNNNNNNNNNNNNNNNNNNNNNNNNNNNNNNNNNNNNNNNNNNNNNNNNNNNNNNNNNNNNNNNNNNNNNNNNNNNNNNNNNNNNNNNNNNNNNNNNNNNNNNNNNNNNNNNNNNNNNNNNNNNNNNNNNNNNNNNNNNNNNNNNNNNNNNNNNNNNNNNNNNNNNNNNNNNNNNNNNNNNNNNNNNNNNNNNNNNNNNNNNNNNNNNNNNNNNNNNNNNNNNNNNNNNNNNNNNNNNNNNNNNNNNNNNNNNNNNNNNNNNNNNNNNNNNNNNNNNNNNNNNNNNNNNNNNNNNNNNNNNNNNNNNNNNNNNNNNNNNNNNNNNNNNNNNNNNNNNNNNNNNNNNNNNNNNNNNNNNNNNNNNNNNNNNNNNNNNNNNNNNNNNNNNNNNNNNNNNNNNNNNNNNNNNNNNNNNNNNNNNNNNNNNNNNNNNNNNNNNNNNNNNNNNNNNNNNNNNNNNNNNNNNNNNNNNNNNNNNNNNNNNNNNNNNNNNNNNNNNNNNNNNNNNNNNNNNNNNNNNNNNNNNNNNNNNNNNNNNNNNNNNNNNNNNNNNNNNNNNNNNNNNNNNNNNNNNNNNNNNNNNNNNNNNNNNNNNNNNNNNNNNNNNNNNNNNNNNNNNNNNNNNNNNNNNNNNNNNNNNNNNNNNNNNNNNNNNNNNNNNNNNNNNNNNNNNNNNNNNNNNNNNNNNNNNNNNNNNNNNNNNNNNNNNNNNNNNNNNNNNNNNNNNNNNNNNNNNNNNNNNNNNNNNNNNNNNNNNNNNNNNNNNNNNNNNNNNNNNNNNNNNNNNNNNNNNNNNNNNNNNNNNNNNNNNNNNNNNNNNNNNNNNNNNNNNNNNNNNNNNNNNNNNNNNNNNNNNNNNNNNNNNNNNNNNNNNNNNNNNNNNNNNNNNNNNNNNNNNNNNNNNNNNNNNNNNNNNNNNNNNNNNNNNNNNNNNNNNNNNNNNNNNNNNNNNNNNNNNNNNNNNNNNNNNNNNNNNNNNNNNNNNNNNNNNNNNNNNNNNNNNNNNNNNNNNNNNNNNNNCATACACACACCATCACACTGTACACACCATCACATGTACACACCATCACACTGTACACACCATCACACTGTACACACCATCACACTGTACACACCATCACACTGTACACACCATCACAACATACCAAGGCTTCTCGATGTTGTCTTATGAAGTCCTCAGGGGACGACGCCCACTTTGGAAGTATCACATCTCCAACAACTGTTTTGTCTTGGGTGACACCAAGGTCATAGCCTATAAAGACAAGTTAGCAGAGTGCTTAGAAATGAATGCAACCCTTTAAGTNATTAACTAGGCAAACANTGATGGNTATTNTAAGCAGTAATTACTGGATCAAAGTCACTTGAAAGTGAGACCAAATATTCAAGATGAACAACAAAAAAAAAANAAANAAAAAAANAAAAAAANAAATTTAGTTGTTAGTCCTTGAATAAATTAATTAATTAACTAGCAATTATTGAAGTTCACATTTTAATTTTTTGAGAGCTTTTTTGAAGCTTGAGGAGTTGGCTTTGTATTTCCCAAATATTTATTAAACCATTCCGCAATGTATTAATTATGAGTACTTATTGGAACAATTGACCTAACCATTTATATTCCTCAAGAAATCCGGTAGGCAGAAAAACTCTGGTGTAAGTTCTTTCACATCACTTCCTAAATAGGTGATTCTCCAAGCATTGGGTATGGAGAAGAACTGTCTATCAGGGTGATCAAACCTAGAGTATTAGCAATATAGCAGAGTGGGTAAGATTGAAGTGAAGATGGGTCAAGATACCATCCCAGCTGTATGTGGCACCTGTTCCCACACAACTCACTTTCCACTCTGTAGGTTGATGTGCAGGGATGTGAAGGGCTNCANTACGNNNNNAGTAGTGGATGACAGTTGTGGCATTGCTGTAGTGTGTNCCNTAGTGAAACTTGCCCATCACTGGATCGTCAAATTCCTCATATCTGCAAGTGCACAGTGCGCATAAGTGTTGTGTGTATAATTGACCACTGTGTCAACACCACATGACACTTTTAAATGGCTGGCGTCCATACTTTGTCCGGACCTTTTCCTCCAGGTCAGGGTTCTGTATCCCCATGGGTTTGGACAGGTCTCTGAACACCTCAGAGTTGCTCAGGTCCAATGTATCACAACTGTAGTCTTGCAGGATCCATGGAAACTAAACACGGAGCAGATACTAAATGATAATTTGCATACAGCTCAAGTATCCTCACAATAGGATATTGGTTGAGGTCATTGTAGGTCCTTCCTGATATAGTGTTGAGTTGCATGATGTAATCAAAATTTGTAATTTCACGTTTCACCCATTTCTACAGTTGTGCAGTTGAATAATGAAAATGAAAGCAGTAGTGAAGGTGGTCATGGAGGAGGTAAGGAATGAGGGAGAGGTGGTCATGGAGGAGGTAAGGGGGAGAGGTGGTCATGGAGGAGGTAAGGGGGAGAGGTGGCCATGGAGGAGGTAAGGATGTAAGGGAGGAGGTGGAGGTGGTGAGGGAGGAGAAAAATTAACACAAGGTGTACAGACAGGGTATAACGACAGTTAGTGTATTCCCAACCTGAATTAGACCTGAAGACTGCATCAGTTTGGCAGGCTCTTGTGTGCTGAAGATGTTAAGGTTTGGAGTACTCAGCGAAACAATGCAGTTGCACACCTTATTCAAAATCTGTGCAACAAGGAGAGAGATACATGCACACCACTTAACACCCAGTCTGGAGACAATGAAAACACTGTGCCTTTTTGGTGGGAAAATTAAGGAAGTAGTTTGTCTGATCCAAAAGAAACATCTCCAGTGCAGTACGGCTTGTGTTGTACCTACGAAGGTGAAGCTCCCTTATCTGGTTAAGTTTGACTGTGAAGTCAGTGAGGTACCCTGCGAAAGAAAGAGAAAATGAATAACCATTGGAGCCTTGCCTTAGGAAGAGGCACTAATAGTTCAATGTCAGATATATCTGGTCACATGTCTTAGCACAAAGTCAACTGTCATACACCTATGCTATTCTAAGTAGTGCACACTCTTGTTACAGATATGCTACTCCATGTTTACAGATATGCTACTCCAAGTGCACACNNTTGTTACAGATATGCTACTCCAAGTGCACACTTTTGTTACAGATATGCTACTACAAGTGCACACTTTTGTTACAGATATGCTACTCCAAGTGCACACTCTTGTTCCTCCCTTCTTCATTTCCTCCTCCCTATCTGTGATCACACCACCCCTTTAACTCTCCCGGTACACTCACAAGATATCGCATCCTTGTCTTGTGCATCCGACACAAAGTAGATGCATCTATTTGTAATGTCCAAGGTTCCAGAGATGACCTCTACCACCGTCACAAGCTGACACATCTCTCTTATNATCAGTTTCTCTTTCTTGCCCTTCTCCAGAGCCACAGCTGGCTCATCTCGAGCTAGCTTTTGGATCTGATCAAACAGATGTTCCTCCTCCTCCTCACTGGNGGACTTTGTAGCTGTGGCCATGGCTAGCAGAGAGGTGTCTGCAGTGCTGGACACCAGGNGAGTCTCCCCAGAGCTGTGTTGACTAGCATCGGTGTGTTTGTTATAGTTGTAGTTACGGGCCAGTTTTAGCCGCATGCGTGAAAAGTTCTCTGTTCTGTCCATCTTCCACAGGTAGAGATCAGGATCTCTGGAACAGAGGATCACGGAATCACAGGGTTATGTACATATACATTCATGTGTGCCACACAGCACCACACAGAGGTACATACATGTATGTACTGTTCCATCATGTATAAAACATGAGTACAACTTCATGTACAACATCATGGGACATGTGTCCCCATGGAACATGTGTACCCATGTACAACATCATGGAACATGTGTACCATCATGTACAACATATGTACCATCAATGTACTANATACTGGCACATAGTGGGGACATGTGTACCATCATGTACNNNNNNNNNNNNNNNNNNNNNNNNNNNNNNNNNNNNNNNNNNNNNNNNNNNNNNNNNNNNNNNNNNNNNNNNNNNNNNNNNNNNNNNNNNNNNNNNNNNNNNNNNNNNNNNNNNNNNNNNNNNNNNNNNNNNNNNNNNNNNNNNNNNNNNNNNNNNNNNNNNNNNNNNNNNNNNNNNNNNNNNNNNNNNNNNNNNNNNNNNNNNNNNNNNNNNNNNNNNNNNNNNNNNNNNNNNNNNNNNNNNNNNNNNNNNNNNNNNNNNNNNNNNNNNNNNNNNNNNNNNNNNNNNNNNNNNNNNNNNNNNNNNNNNNNNNNNNNNNNNNNNNNNNNNNNNNNNNNNNNNNNNNNNNNNNNNNNNNNNNNNNNNNNNNNNNNNNNNNNNNNNNNNNNNNNNNNNNNNNNNNNNNNNNNNNNNNNNNNNNNNNNNNNNNNNNNNNNNNNNNNNNNNNNNNNNNNNNNNNNNNNNNNNNNNNNNNNNNNNNNNNNNNNNNNNNNNNNNNNNNNNNNNNNNNNNNNNNNNNNNNNNNNNNNNNNNNNNNNNNNNNNNNNNNNNNNNNNNNNNNNNNNNNNNNNNNNNNNNNNNNNNNNNNNNNNNNNNNNNNNNNNNNNNNNNNNNNNNNNNNNNNNNNNNNNNNNNNNNNNNNNNNNNNNNNNNNNNNNNNNNNNNNNNNNNNNNNNNNNNNNNNNNNNNNNNNNNNNNNNNNNNNNNNNNNNNNNNNNNNNNNNNNNNNNNNNNNNNNNNNNNNNNNNNNNNNNNNNNNNNNNNNNNNNNNNNNNNNNNNNNNNNNNNNNNNNNNNNNNNNNNNNNNNNNNNNNNNNNNNNNNNNNNNNNNNNNNNNNNNNNNNNNNNNNNNNNNNNNNNNNNNNNNNNNNNNNNNNNNNNNNNNNNNNNNNNNNNNNNNNNNNNNNNNNNNNNNNNNNNNNNNNNNNNNNNNNNNNNNNNNNNNNNNNNNNNNNNNNNNNNNNNNNNNNNNNNNNNNNNNNNNNNNNNNNNNNNNNNNNNNNNNNNNNNNNNNNNNNNNNNNNNNNNNNNNNNNNNNNNNNNNNNNNNNNNNNNNNNNNNNNNNNNNNNNNNNNNNNNNNNNNNNNNNNNNNNNNNNNNNNNNNNNNNNNNNNNNNNNNNNNNNNNNNNNNNNNNNNNNNNNNNNNNNNNNNNNNNNNNNNNNNNNNNNNNNNNNNNNNNNNNNNNNNNNNNNNNNNNNNNNNNNNNNNNNNNNNNNNNNNNNNNNNNNNNNNNNNNNNNNNNNNNNNNNNNNNNNNNNNNNNNNNNNNNNNNNNNNNNNNNNNNNNNNNNNNNNNNNNNNNNNNNNNNNNNNNNNNNNNNNNNNNNNNNNNNNNNNNNNNNNNNNNNNNNNNNNNNNNNNNNNNNNNNNNNNNNNNNNNNNNNNNNNNNNNNNNNNNNNNNNNNNNNNNNNNNNNNNNNNNNNNNNNNNNNNNNNNNNNNNNNNNNNNNNNNNNNNNNNNNNNNNNNNNNNNNNNNNNNNNNNNNNNNNNNNNNNNNNNNNNNNNNNNNNNNNNNNNNNNNNNNNNNNNNNNNNNNNNNNNNNNNNNNNNNNNNNNNNNNNNNNNNNNNNNNNNNNNNNNNNNNNNNNNNNNNNNNNNNNNNNNNNNNNNNNNNNNNNNNNNNNNNNNNNNNNNNNNNNNNNNNNNNNNNNNNNNNNNNNNNNNNNNNNNNNNNNNNNNNNNNNNNNNNNNNNNNNNNNNNNNNNNNNNNNNNNNNNNNNNNNNNNNNNNNNNNNNNNNNNNNNNNNNNNNNNNNNNNNNNNNNNNNNNNNNNNNNNNNNNNNNNNNNNNNNNNNNNNNNNNNNNNNNNNNNNNNNNNNNNNNNNNNNNNNNNNNNNNNNNNNNNNNNNNNNNNNNNNNNNNNNNNNNNNNNNNNNNNNNNNNNNNNNNNNNNNNNNNNNNNNNNNNNNNNNNNNNNNNNNNNNNNNNNNNNNNNNNNNNNNNNNNNNNNNNNNNNNNNNNNNNNNNNNNNNNNNNNNNNNNNNNNNNNNNNNNNNNNNNNNNNNNNNNNNNNNNNNNNNNNNNNNNNNNNNNNNNNNNNNNNNNNNNNNNNNNNNNNNNNNNNNNNNNNNNNNNNNNNNNNNNNNNNNNNNNNNNNNNNNNNNNNNNNNNNNNNNNNNNNNNNNNNNNNNNNNNNNNNNNNNNNNNNNNNNNNNNNNNNNNNNNNNNNNNNNNNNNNNNNNNNNNNNNNNNNNNNNNNNNNNNNNNNNNNNNNNNNNNNNNNNNNNNNNNNNNNNNNNNNNNNNNNNNNNNNNNNNNNNNNNNNNNNNNNNNNNNNNNNNNNNNNNNNNNNNNNNNNNNNNNNNNNNNNNNNNNNNNNNNNNNNNNNNNNNNNNNNNNNNNNNNNNNNNNNNNNNNNNNNNNNNNNNNNNNNNNNNNNNNNNNNNNNNNNNNNNNNNNNNNNNNNNNNNNNNNNNNNNNNNNNNNNNNNNNNNNNNNNNNNNNNNNNNNNNNNNNNNNNNNNNNNNNNNNNNNNNNNNNNNNNNNNNNNNNNNNNNNNNNNNNNNNNNNNNNNNNNNNNNNNNNNNNNNNNNNNNNNNNNNNNNNNNNNNNNNNNNNNNNNNNNNNNNNNNNNNNNNNNNNNNNNNNNNNNNNNNNNNNNNNNNNNNNNNNNNNNNNNNNNNNNNNNNNNNNNNNNNNNNNNNNNNNNNNNNNNNNNNNNNNNNNNNNNNNNNNNNNNNNNNNNNNNNNNNNNNNNNNNNNNNNNNNNNNNNNNNNNNNNNNNNNNNNNNNNNNNNNNNNNNNNNNNNNNNNNNNNNNNNNNNNNNNNNNNNNNNNNNNNNNNNNNNNNNNNNNNNNNNNNNNNNNNNNNNNNNNNNNNNNNNNNNNNNNNNNNNNNNNNNNNNNNNNNNNNNNNNNNNNNNNNNNNNNNNNNNNNNNNNNNNNNNNNNNNNNNNNNNNNNNNNNNNNNNNNNNNNNNNNNNNNNNNNNNNNNNNNNNNNNNNNNNNNNNNNNNNNNNNNNNNNNNNNNNNNNNNNNNNNNNNNNNNNNNNNNNNNNNNNNNNNNNNNNNNNNNNNNNNNNNNNNNNNNNNNNNNNNNNNNNNNNNNNNNNNNNNNNNNNNNNNNNNNNNNNNNNNNNNNNNNNNNNNNNNNNNNNNNNNNNNNNNNNNNNNNNNNNNNNNNNNNNNNNNNNNNNNNNNNNNNNNNNNNNNNNNNNNNNNNNNNNNNNNNNNNNNNNNNNNNNNNNNNNNNNNNNNNNNNNNNNNNNNNNNNNNNNNNNNNNNNNNNNNNNNNNNNNNNNNNNNNNNNNNNNNNNNNNNNNNNNNNNNNNNNNNNNNNNNNNNNNNNNNNNNNNNNNNNNNNNNNNNNNNNNNNNNNNNNNNNNNNNNNNNNNNNNNNNNNNNNNNNNNNNNNNNNNNNNNNNNNNNNNNNNNNNNNNNNNNNNNNNNNNNNNNNNNNNNNNNNNNNNNNNNNNNNNNNNNNNNNNNNNNNNNNNNNNNNNNNNNNNNNNNNNNNNNNNNNNNNNNNNNNNNNNNNNNNNNNNNNNNNNNNNNNNNNNNNNNNNNNNNNNNNNNNNNNNNNNNNNNNNNNNNNNNNNNNNNNNNNNNNNNNNNNNNNNNNNNNNNNNNNNNNNNNNNNNNNNNNNNNNNNNNNNNNNNNNNNNNNNNNNNNNNNNNNNNNNNNNNNNNNNNNNNNNNNNNNNNNNNNNNNNNNNNNNNNNNNNNNNNNNNNNNNNNNNNNNNNNNNNNNNNNNNNNNNNNNNNNNNNNNNNNNNNNNNNNNNNNNNNNNNNNNNNNNNNNNNNNNNNNNNNNNNNNNNNNNNNNNNNNNNNNNNNNNNNNNNNNNNNNNNNNNNNNNNNNNNNNNNNNNNNNNNNNNNNNNNNNNNNNNNNNNNNNNNNNNNNNNNNNNNNNNNNNNNNNNNNNNNNNNNNNNNNNNNNNNNNNNNNNNNNNNNNNNNNNNNNNNNNNNNNNNNNNNNNNNNNNNNNNNNNNNNNNNNNNNNNNNNNNNNNNNNNNNNNNNNNNNNNNNNNNNNNNNNNNNNNNNNNNNNNNNNNNNNNNNNNNNNNNNNNNNNNNNNNNNNNNNNNNNNNNNNNNNNNNNNNNNNNNNNNNNNNNNNNNNNNNNNNNNNNNNNNNNNNNNNNNNNNNNNNNNNNNNNNNNNNNNNNNNNNNNNNNNNNNNNNNNNNNNNNNNNNNNNNNNNNNNNNNNNNNNNNNNNNNNNNNNNNNNNNNNNNNNNNNNNNNNNNNNNNNNNNNNNNNNNNNNNNNNNNNNNNNNNNNNNNNNNNNNNNNNNNNNNNNNNNNNNNNNNNNNNNNNNNNNNNNNNNNNNNNNNNNNNNNNNNNNNNNNNNNNNCCACACACACATACCCCACNCACACACACACACACACACACACACACACCACACACAAACACACACCAATGAAGCAGCTGTGGGGTTGCTCTACAAATGGAGTACCTCTACAACATTAGCCATGAGGTCATCTCTCGTTTGACCTCTTTGATCGTCCCCCTTACGCACCACCTGCATTTGCTCAAAGAGGTTCACTATGACTCTCTTGCCAGTGAAGGGAGAGGAGTAGCCTCTGTAGCTCTTCACAGGCGGGTCTCTTGTCGGATGCCTCTCGTTAAACAGACGAGTTGGTGTCTGACCAAAATTGGTTATAATACTTTCAATCTCTCGGCGGCTTTTTGGGTCTTGGATGTTGTCTAAGTTGACATTTTCTTTATAAGGGAAGTGAAAATATTAACAACATTGAAGATTAGACAATAAGGTCCCAATTATCCATGTACCTTGGTATGAGTACTTGTTAAACACATTCATAGCCTTTTCTGCCTCAGGCCCAGTCTGTTTGTACCCAAAAATGAGGTCAATCCACTCATTGAGGTGTGCTGAAACGTATTCAGANTCCTGAGGGAGGAGGGTAGAGCACAATGNACACACATGCTCCTACNNGNNATTACACTGTACACACCATCACACTGTACACACCATCACAC>scaffold4236|size40413CCACCACCACCACCACCACCACCACCACCACCACCTCCTCCAGCATGGCCGGTATTAACTGCAGAATGGCGGGCACCCTCACTTCCATCCAGCATGAGGCCGAGGTGGAGACCGGGAAGCCGGGGATGTGGGCCAAAGAGGAGACTCAATAGCTGTATGCACAATACTTGATCATTATTGTACTAAATTATCCAAGAGATGGTTATGGTGCATACGAAATGCGCAATATTATCCGTCAGTTTATGCTTGACATAGATGCATGCATGGCATTAAAGTGCCAGTGTTGGCTTTAAGAGACTGTTGGATTGATGTCTGATCGATCTGGAGAACAGGACAAAGAGACAGAATCCAAACGGACTCACAATAGTATATAAAGTGGGTAGTTCCCAATGCAGTGCTACAAGATGACGAGCAAGAGAAGAAGCGTGCGTATGAAGAGAGGGTGAGAGAAATCGAGCATGGCTCCTTTGCACCCCTCGTAGTCTCAATCTCAGGAGGTATGGGTCCGATAGCCACAACCGTGTACAAAAGAATGGCCTCGCTCATAGCTCAGTGAAAAATACAATAATCCATATATCTTTTTTGGCTAAGATGCAAGCTGAGTTTCTCCTTATTGCGATCTGCATAACACATGTGCATCAGAGGCTCGAGGTCCTCCTATCACAGACCAACAAACATGTTTAGTGAGACCATTGTCCTATCCTGCTTGGTAAACAGGATCTCAATTTGAACATTGAAATCCTATCCGTCTTTTTTCTTTTATTNNNTNCTTANNTCATTACCACGTTTCCACGCATTATGTGTATATTTTTATTTAAAAAGCGTATACTTTACGGACTTATCTGCTGTACTGATTTGATTTATTGTCAGTTCAAAGCGATAGTCTCGTACCCAGACCTGACGGACGTAAAAGCATGTTTGCACGTCAGCCGAGTCTGGGTANNNNNNNNNNGGGGGCGGGGCCTAAATATACTACGGCCTGTCCGGGCCATGTAAACTTAAAAGACGTTACGAAAGTGGAAAAAAAAACTTACGAAATAAGTAAATTAAAACATTCTCTATACGCGAAAGTTGGCTCTCCTCCTCTCTTCCTGCCACTCCTCNTACCCCCATTGTCATCTTGTAACCATTACTGCCGATACATACATGATCACTTGCACAAGATTTAGTCACTATAAACTTATTGTGTAATAACTATTGCATAAAGTAGTTAAACGTGGTTAATTTCGGGTTTCCTCCCTCATCTCAAGTCACATGTTCTCATGTTCACGCCACGAGCCAGGCAGAAAACAGCATTTTTATTCATTGTCATTATTTTTATTTTATTAATTTTAGATTANCCATTACAATAAATAAATACATACATAATCAATAAACATTTGAGCACATAATACTACAAACCTACCTACCTAGGACNCNAAGAGTCCTTATCAGTTATTGAAATGACGACGACNNAGCANNNNCNCNNACCAGGGCAATGCTTAGCCTTCCATATAGCCCAGACACTGCTGAAGACATGGGACAAGCAGTTCGAGTGGCGATCCATGTAGCGAGTCTGTCGAGGCACCTGCTGNTANGTGTAAGCTCAGCTGGGGAGTTGGCGTGACCAAAAGCCAAGCTGTGGCGTGGGGAGAAGACACAGAGGGAAACCGNNNNNNNNNNNNNNNNNNNNNNNNNNNNNNNNNNNNNNNNNNNNNNNNNNNNNNNNNNNNNNNNNNNNNNNNNNNNNNNNNNNNNNNNNNNNNNNNNNNNNNNNNNNNNNNNNNNNNNNNNNNNNNNNNNNNNNNNNNNNNNNNNNNNNNNNNNNNNNNNNNNNNNNNNNNNNNNNNNNNNNNNNNNNNNNNNNNNNNNNNNNNNNNNNNNNNNNNNNNNNNNNNNNNNNNNNNNNNNNNNNNNNNNNNNNNNNNNNNNNNNNNNNNNNNNNNNNNNNNNNNNNNNNNNNNNNNNNNNNNNNNNNNNNNNNNNNNNNNNNNNNNNNNNNNNNNNNNNNNNNNNNNNNNNNNNNNNNNNNNNNNNNNNNNNNNNNNNNNNNNNNNNNNNNNNNNNNNNNNNNNNNNNNNNNNNNNNNNNNNNNNNNNNNNNNNNNNNNNNNNNNNNNNNNNNNNNNNNNNNNNNNNNNNNNNNNNNNNNNNNNNNNNNNNNNNNNNNNNNNNNNNNNNNNNNNNNNNNNNNNNNNNNNNNNNNNNNNNNNNNNNNNNNNNNNNNNNNNNNNNNNNNNNNNNNNNNNNNNNNNNNNNNNNNNNNNNNNNNNNNNNNNNNNNNNNNNNNNNNNNNNNNNNNNNNNNNNNNNNNNNNNNNNNNNNNNNNNNNNNNNNNNNNNNNNNNNNNNNNNNNNNNNNNNNNNNNNNNNNNNNNNNNNNNNNNNNNNNNNNNNNNNNNNNNNNNNNNNNNNNNNNNNNNNNNNNNNNNNNNNNNNNNNNNNNNNNNNNNNNNNNNNNNNNNNNNNNNNNNNNNNNNNNNNNNNNNNNNNNNNNNNNNNNNNNNNNNNNNNNNNNNNNNNNNNNNNNNNNNNNNNNNNNNNNNNNNNNNNNNNNNNNNNNNNNNNNNNNNNNNNNNNNNNNNNNNNNNNNNNNNNNNNNNNNNNNNNNNNNNNNNNNNNNNNNNNNNNNNNNNNNNNNNNNNNNNNNNNNNNNNNNNNNNNNNNNNNNNNNNNNNNNNNNNNNNNNNNNNNNNNNNNNNNNNNNNNNNNNNNNNNNNNNNNNNNNNNNNNNNNNNNNNNNNNNNNNNNNNNNNNNNNNNNNNNNNNNNNNNNNNNNNNNNNNNNNNNNNNNNNNNNNNNNNNNNNNNNNNNNNNNNNNNNNNNNNNNNNNNNNNNNNNNNNNNNNNNNNNNNNNNNNNNNNNNNNNNNNNNNNNNNNNNNNNNNNNNNNNNNNNNNNNNNNNNNNNNNNNNNNNNNNNNNNNNNNNNNNNNNNNNNNNNNNNNNNNNNNNNNNNNNNNNNNNNNNNNNNNNNNNNNNNNNNNNNNNNNNNNNNNNNNNNNNNNNNNNNNNNNNNNNNNNNNNNNNNNNNNNNNNNNNNNNNNNNNNNNNNNNNNNNNNNNNNNNNNNNNNNNNNNNNNNNNNNNNNNNNNNNNNNNNNNNNNNNNNNNNNNNNNNNNNNNNNNNNNNNNNNNNNNNNNNNNNNNNNNNNNNNNNNNNNNNNNNNNNNNNNNNNNNNNNNNNNNNNNNNNNNNNNNNNNNNNNNNNNNNNNNNNNNNNNNNNNNNNNNNNNNNNNNNNNNNNNNNNNNNNNNNNNNNNNNNNNNNNNNNNNNNNNNNNNNNNNNNNNNNNNNNNNNNNNNNNNNNNNNNNNNNNNNNNNNNNNNNNNNNNNNNNNNNNNNNNNNNNNNNNNNNNNNNNNNNNNNNNNNNNNNNNNNNNNNNNNNNNNNNNNNNNNNNNNNNNNNNNNNNNNNNNNNNNNNNNNNNNNNNNNNNNNNNNNNNNNNNNNNNNNNNNNNNNNNNNNNNNNNNNNNNNNNNNNNNNNNNNNNNNNNNNNNNNNNNNNNNNNNNNNNNNNNNNNNNNNNNNNNNNNNNNNNNNNNNNNNNNNNNNNNNNNNNNNNNNNNNNNNNNNNNNNNNNNNNNNNNNNNNNNNNNNNNNNNNNNNNNNNNNNNNNNNNNNNNNNNNNNNNNNNNNNNNNNNNNNNNNNNNNNNNNNNNNNNNNNNNNNNNNNNNNNNNNNNNNNNNNNNNNNNNNNNNNNNNNNNNNNNNNNNNNNNNNNNNNNNNNNNNNNNNNNNNNNNNNNNNNNNNNNNNNNNNNNNNNNNNNNNNNNNNNNNNNNNNNNNNNNNNNNNNNNNNNNNNNNNNNNNNNNNNNNNNNNNNNNNNNNNNNNNNNNNNNNNNNNNNNNNNNNNNNNNNNNNNNNNNNNNNNNNNNNNNNNNNNNNNNNNNNNNNNNNNNNNNNNNNNNNNNNNNNNNNNNNNNNNNNNNNNNNNNNNNNNNNNNNNNNNNNNNNNNNNNNNNNNNNNNNNNNNNNNNNNNNNNNNNNNNNNNNNNNNNNNNNNNNNNNNNNNNNNNNNNNNNNNNNNNNNNNNNNNNNNNNNNNNNNNNNNNNNNNNNNNNNNNNNNNNNNNNNNNNNNNNNNNNNNNNNNNNNNNNNNNNNNNNNNNNNNNNNNNNNNNNNNNNNNNNNNNNNNNNNNNNNNNNNNNNNNNNNNNNNNNNNNNNNNNNNNNNNNNNNNNNNNNNNNNNNNNNNNNNNNNNNNNNNNNNNNNNNNNNNNNNNNNNNNNNNNNNNNNNNNNNNNNNNNNNNNNNNNNNNNNNNNNNNNNNNNNNNNNNNNNNNNNNNNNNNNNNNNNNNNNNNNNNNNNNNNNNNNNNNNNNNNNNNNNNNNNNNNNNNNNNNNNNNNNNNNNNNNNNNNNNNNNNNNNNNNNNNNNNNNNNNNNNNNNNNNNNNNNNNNNNNNNNNNNNNNNNNNNNNNNNNNNNNNNNNNNNNNNNNNNNNNNNNNNNNNNNNNNNNNNNNNNNNNNNNNNNNNNNNNNNNNNNNNNNNNNNNNNNNNNNNNNNNNNNNNNNNNNNNNNNNNNNNNNNNNNNNNNNNNNNNNNNNNNNNNNNNNNNNNNNNNNNNNNNNNNNNNNNNNNNNNNNNNNNNNNNNNNNNNNNNNNNNNNNNNNNNNNNNNNNNNNNNNNNNNNNNNNNNNNNNNNNNNNNNNNNNNNNNNNNNNNNNNNNNNNNNNNNNNNNNNNNNNNNNNNNNNNNNNNNNNNNNNNNNNNNNNNNNNNNNNNNNNNNNNNNNNNNNNNNNNNNNNNNNNNNNNNNNNNNNNNNNNNNNNNNNNNNNNNNNNNNNNNNNNNNNNNNNNNNNNNNNNNNNNNNNNNNNNNNNNNNNNNNNNNNNNNNNNNNNNNNNNNNNNNNNNNNNNNNNNNNNNNNNNNNNNNNNNNNNNNNNNNNNNNNNNNNNNNNNNNNNNNNNNNNNNNNNNNNNNNNNNNNNNNNNNNNNNNNNNNNNNNNNNNNNNNNNNNNNNNNNNNNNNNNNNNNNNNNNNNNNNNNNNNNNNNNNNNNNNNNNNNNNNNNNNNNNNNNNNNNNNNNNNNNNNNNNNNNNNNNNNNNNNNNNNNNNNNNNNNNNNNNNNNNNNNNNNNNNNNNNNNNNNNNNNNNNNNNNNNNNNNNNNNNNNNNNNNNNNNNNNNNNNNNNNNNNNNNNNNNNNNNNNNNNNNNNNNNNNNNNNNNNNNNNNNNNNNNNNNNNNNNNNNNNNNNNNNNNNNNCCAGCCAATAACAGCAAGCACACGGGNCTNGNNTNNACNNGTNTATANATNNATGGCCACAAGCTGGCCAACCCAAGGCTGCATTGGAAGCAATAGGGGCAAACACAAGAGGGTGGGGCGTATATCAAAGGGGAGGCCAGTTGCAGAATGTGAACGGGGGAATTTCGAGAGCCTAAACAGTACCATAAGGGTTAGAAGCACTGAGTATGTACAGTGTATGTGTCAACGTTGCTCCATAGATAGTGTGGCCCAAAGGTGGAAGCGGCCAGTCCATACGCAGCACACACACACNCNNNCNNNNNNACNNANAGCAGCATCAGCTGCTCTACGCCTAAACACACTCACGCGACGCTCGGTTGCAATAAGCCGAAAGGATTTCTGTCCAGCGGCAGTGATGGAGGAGGAGTGACACATTTCTTCGTCGCTTTCAACAGACGCTTTAGATAAAAACAGAAAGCCTTGCTTTTTAGTTTAGGGTTCGACTGTTTTGTATACATCGACCACGGCTCCAAGAGCTCTTTAGTTCTGCAAAGATGGGCACACTTATGACTCCATTCACTCGGTCTAACCGACCGTCAAAATATATACAACGTACACAAAACTTACTTGAAAACCTCTTCCGACATGCTGAGACCTGCCCCCTCCTCCTCTGAATGATGTCAGCTTAGGTGCCTCGTTTGCAGGCTCAAATAAATTCATTGCTAAAGTCACGTGCGCAAACTATTTTTCNTTATATAGCTCACAATTAGATCATTTGTAACGCTTCTGTTCTCAAGTAAGCAAAAAATAAAAAAAGAAAGTTCTGTACCCAGACCCAAAAGTAGTAAGGAATGCAATCAGGACCAGTGGGTGTGGCATAAATTCTTTGCCCCCTCCCTATGGAAACGATCTATAGCTGCATGGGATCATGAATCATAAGGTATTCCAAGTATGTAGCNGCGTGAATGACTCTCCTGATATCAGATCCTGCAGACAGACTGAGTTGTACTGTGATCAAGTNGCATGCTTTCATATAATGCATGACCTAATGTGGTGCTCAGCAGGTTCATAACAGAGGGGCTGTGAAGTCTGACACTTCATATCCTTAACAATGGCAAATGAAATGAAACATAGATGGTTATGATAAATTTCTGCACATACATGAATATAAAAATACCCACTAACCACTCCATGCAATAAAGAACAGAAGAGAGGGTACAAAACGAAGGAAGAAACACCTGACACTCACAACATGTCACATGACAACAGCTACGCCGGCAGCTGCCCTGCAAGTCTCCATAGAACTGACCAGATCCCAGCGTGTTGTTTACAGGGTCATACAGTTCTGCTGTTTTCAAGTACGAAGGACCATTGATCCCTCCAACAGCCACCATATGTGCACCAATCGCACATAGNCAGACTCCATCGCGAGCTGAGGTCATTTTGGCTGCTGGATACCATTTGCCACTGCGAACATCGAGAAACTCCACAGAGTCCAGCCGTGAGGTTCCATCGTTACCACCNGCAACAAAAATCTGCTGACCCAAGACTGCCACCCCGATCCCCGCACGAGGTTTCCCCAGCGGGGGCAATGTTCTCCATCGGTTGACATGAGGATCGTAGCACTCCACAACGTTGAGAAACGAGGTCCCATCGTTCCCGCCAAATGTGTACAACTTGTCACCAAAGACAGCTACTCCCGGGAAACTTCTCCGTGAGCTCATCTGAGCGACAGTGACCCAAACGTTCTTTTCAAGGTCGAACCTTTCAACCGTGTTGAGACAGGAGCGACCGTCGTAGCCCCCCGCGGCGTACATGGCGCCTCCCATGACCCCCACGGCCACGCCCGAGCGACACGTGCTCAGGGGGGCCACGTACGTCCAGAACTGAGTGGCCGGATCGAAGCACTCCACGGTGCTGAGGCGCAGAGAGCCGTCCGACCCGCCCACCGCGTACACTTTGCCGTCGAGGACCCCGACGCCCAACTGTTGCCTACGAACCATGAGGGGCGTGCCCTCGCTCCACTTGTTTTCCAGCAGCGAGAACGTCTCGACGGAGTGAGTGATGCTTTCGGACGACTCCTTCCCGCCAACCGCGTACACGACGCCCACCGTGGACTTGCGCGGAAAGAAGCACGGATTGGCCGAACGGATGTCTCTGTCGGCAGGCATTAGGTGGTAGCGTTTCGCGTCGTCAAGAAGGTCCCGACACTCGATGCTCTGCTTCACGATGGGATCACGGTCAACGTCGTTCATCAAACAGTCGACGGCGAGGAGCNNNNNNNNNACGTGCTTCAGCAGCTTTGGCAGCTGTTGCCCGCGCTCGCCCGGGTCCTGCCTGACCCAGCTCATGACAGCTGCGTACACGACCTCTTCCCTGGGTACGTTCAGGTCGTTGCTTGAGATCAGCGAGGCGATACCTTCGGCCGAACTGTGGAAGAACTCGTTGGTGTTGGTCACCTCGAGGAAGTGCCGGCGAGCAAAAGAATCGACCACCTCCGACAGCTGGGTGCAGCCATTGACTTCGGCAAAATTCTTAACACCCAGGCAGTTGCTCACGTCCAAGTGGTTTTTTAGAAATTCGCAGCACACTTCCTGGGCCTGTTTTATCTGCAGCAATGAGGCAGCCGCTAACGTGTCTTGTACATTTTCCACCGTGACTGTTAAGATCCCTGTATAGGCAAAGTCAATCAAACTGCTCAGAACATCTCCACTCATGCCCTTGATTTGAACTACGTCCTGACCGCTCTCCAAAAGTTCATGAGTAAACATGGCCAGAAAGTAGGCACTGCAGGCAGACAGGATGGCTCTGTGGGCATTTATCTCTCTTCCTTCGGCACTCAACGTCACGTCACATAGCTTTTTGTGACTTCGAAGTAAGTGTACCTTTTGAAGAACGCTCTTGCTGTAGTGAAGTGTTGCTTCGTCGGCATCGAAATGTGATGATGGAGCCGTAGAACGATGTGAGTTTTCCTGACGAGATAGAAGCATTGATGTCTCTTTTTCTGCCATTCTCTTTCATTTCATCGTGTCAAATTGACGAATTGAGTCCAAAGTATGAAAATAGATACATTACGTTTCAAATTTTAAAATTACTTGCGATGTATTTTTAGAACAGTGCCGGATGGAATTGATGCAGAAGTCACGTCTAGAGGGACAACCGAGAATCAAATCGCTTATCTCCTTGTCGCTATCGAATGGCAACACAAAAATAAACAGTTTCACGTTGGTTTCATATCACAAAAGATGTGCTTTTAGAGCAAAAAAAAAATGTTTGTGGCAGTTTCTCGTGCATGATCAGTCCCTTGGTCGTCGTCTTCTTCTTCTTCTTCCTTCGTCACGTATAACTAATTAAATTTCTTTCAGCCCTGGTGACCCATTTGAGATCAACAGATCTGATGTACGACTGTACTTAAAAAACAAACAAAAAAACAAAAAAAAACACGCATACATACGTCGCTATTGAATTTATTTGGGAACTGTATCTTACCAGTAGGCCCTAAATCTGACAACTGTAGGTGAAAGAATGCAGCCATTCCCTTTCCTTCCCTTAGTCTCGTCCCCATATTTTATTCCTTTCGCCGTAGCGGTAATGTGCTGCGGTATATTGGGTCTGGGGACGAGACACTACTACCACCACCACCACCACCACCACCACCACCACCACCNNNNNNNNNNNNNNNNNNNNNNNNNNNNNNNNNNNNNNNNNNNNNNNNNNNNNNNNNNNNNNNNNNNNNNNNNNNNNNNNNNNNNNNNNNNNNNNNNNNNNNNNNNNNNNNNNNNNNNNNNNNNNNNNNNNNNNNNNNNNNNNNNNNNNNNNNNNNNNNNNNNNNNNNNNNNNNNNNNNNNNNNNNNNNNNNNNNNNNNNNNNNNNNNNNNNNNNNNNNNNNNNNNNNNNNNNNNNNNNNNNNNNNNNNNNNNNNNNNNNNNNNNNNNNNNNNNNNNNNNNNNNNNNNNNNNNNNNNNNNNNNNNNNNNNNNNNNNNNNNNNNNNNNNNNNNNNNNNNNNNNNNNNNNNNNNNNNNNNNNNNNNNNNNNNNNNNNNNNNNNNNNNNNNNNNNNNNNNNNNNNNNNNNNNNNNNNNNNNNNNNNNNNNNNNNNNNNNNNNNNNNNNNNNNNNNNNNNNNNNNNNNNNNNNNNNNNNNNNNNNNNNNNNNNNNNNNNNNNNNNNNNNNNNNNNNNNNNNNNNNNNNNNNNNNNNNNNNNNNNNNNNNNNNNNNNNNNNNNNNNNNNNNNNNNNNNNNNNNNNNNNNNNNNNNNNNNNNNNNNNNNNNNNNNNNNNNNNNNNNNNNNNNNNNNNNNNNNNNNNNNNNNNNNNNNNNNNNNNNNNNNNNNNNNNNNNNNNNNNNNNNNNNNNNNNNNNNNNNNNNNNNNNNNNNNNNNNNNNNNNNNNNNNNNNNNNNNNNNNNNNNNNNNNNNNNNNNNNNNNNNNNNNNNNNNNNNNNNNNNNNNNNNNNNNNNNNNNNNNNNNNNNNNNNNNNNNNNNNNNNNNNNNNNNNNNNNNNNNNNNNNNNNNNNNNNNNNNNNNNNNNNNNNNNNNNNNNNNNNNNNNNNNNNNNNNNNNNNNNNNNNNNNNNNNNNNNNNNNNNNNNNNNNNNNNNNNNNNNNNNNNNNNNNNNNNNNNNNNNNNNNNNNNNNNNNNNNNNNNNNNNNNNNNNNNNNNNNNNNNNNNNNNNNNNNNNNNNNNNNNNNNNNNNNNNNNNNNNNNNNNNNNNNNNNNNNNNNNNNNNNNNNNNNNNNNNNNNNNNNNNNNNNNNNNNNNNNNNNNNNNNNNNNNNNNNNNNNNNNNNNNNNNNNNNNNNNNNNNNNNNNNNNNNNNNNNNNNNNNNNNNNNNNNNNNNNNNNNNNNNNNNNNNNNNNNNNNNNNNNNNNNNNNNNNNNNNNNNNNNNNNNNNNNNNNNNNNNNNNNNNNNNNNNNNNNNNNNNNNNNNNNNNNNNNNNNNNNNNNNNNNNNNNNNNNNNNNNNNNNNNNNNNNNNNNNNNNNNNNNNNNNNNNNNNNNNNNNNNNNNNNNNNNNNNNNNNNNNNNNNNNNNNNNNNNNNNNNNNNNNNNNNNNNNNNNNNNNNNNNNNNNNNNNNNNNNNNNNNNNNNNNNNNNNNNNNNNNNNNNNNNNNNNNNNNNNNNNNNNNNNNNNNNNNNNNNNNNNNNNNNNNNNNNNNNNNNNNNNNNNNNNNNNNNNNNNNNNNNNNNNNNNNNNNNNNNNNNNNNNNNNNNNNNNNNNNNNNNNNNNNNNNNNNNNNNNNNNNNNNNNNNNNNNNNNNNNNNNNNNNNNNNNNNNNNNNNNNNNNNNNNNNNNNNNNNNNNNNNNNNNNNNNNNNNNNNNNNNNNNNNNNNNNNNNNNNNNNNNNNNNNNNNNNNNNNNNNNNNNNNNNNNNNNNNNNNNNNNNNNNNNNNNNNNNNNNNNNNNNNNNNNNNNNNNNNNNNNNNNNNNNNNNNNNNNNNNNNNNNNNNNNNNNNNNNNNNNNNNNNNNNNNNNNNNNNNNNNNNNNNNNNNNNNNNNNNNNNNNNNNNNNNNNNNNNNNNNNNNNNNNNNNNNNNNNNNNNNNNNNNNNNNNNNNNNNNNNNNNNNNNNNNNNNNNNNNNNNNNNNNNNNNNNNNNNNNNNNNNNNNNNNNNNNNNNNNNNNNNNNNNNNNNNNNNNNNNNNNNNNNNNNNNNNNNNNNNNNNNNNNNNNNNNNNNNNNNNNNNNNNNNNNNNNNNNNNNNNNNNNNNNNNNNNNNNNNNNNNNNNNNNNNNNNNNNNNNNNNNNNNNNNNNNNNNNNNNNNNNNNNNNNNNNNNNNNNNNNNNNNNNNNNNNNNNNNNNNNNNNNNNNNNNNNNNNNNNNNNNNNNNNNNNNNNNNNNNNNNNNNNNNNNNNNNNNNNNNNNNNNNNNNNNNNNNNNNNNNNNNNNNNNNNNNNNNNNNNNNNNNNNNNNNNNNNNNNNNNNNNNNNNNNNNNNNNNNNNNNNNNNNNNNNNNNNNNNNNNNNNNNNNNNNNNNNNNNNNNNNNNNNNNNNNNNNNNNNNNNNNNNNNNNNNNNNNNNNNNNNNNNNNNNNNNNNNNNNNNNNNNNNNNNNNNNNNNNNNNNNNNNNNNNNNNNNNNNNNNNNNNNNNNNNNNNNNNNNNNNNNNNNNNNNNNNNNNNNNNNNNNNNNNNNNNNNNNNNNNNNNNNNNNNNNNNNNNNNNNNNNNNNNNNNNNNNNNNNNNNNNNNNNNNNNNNNNNNNNNNNNNNNATGTGTGTGTGTNTGTGTNTGTGTGTGTGTNTNTGTGTGTGTGAGGATATGTGTAAGAGGAGAGTATTAAATCATGAGTGCATAAAAGCATACAATGGGTTAAAACAATGAGCTGTTAACAAATCATCAAATATACCAGATCTATTTATACAGGAGTACCACAAATATAGAAATGTAAGCAAATTAAACTAGTTACCATGTTCAATAGTTCCTGGAGGACCTCCATAGTAGGCGGGGTAAGAGAAGCCATTAATTTTTTCAAAACACTGTATTTTATGTGCTGTCTGAATGATGCTTTTATAGTTGACGATCCTTTCAGTGCCACAGTGAGAGTCCTTATCAGCAGACATACAAGGGTGGTTCCCAGACCACCAAGGGGAACATTNTTTAGCTCCTTGAAGGACTCCACCAGCTTCTCAAAACAGCCCACTTCTGCAAAGAGCTCCTGTGTGGTTTGGATAACAATGGGTGCTCATGGCTTGGTCGTGTGGCTTTACCTTCAACTGGGTGGAGCCCTTGGGAGCATCCATCAATAGTGAAATAGAGTCTGGAGGAGAGAACCAAGCAATAAGGTGCAAGAAAGGAAGTGAATGATGAGGGAGGTGAACAAGGGGGAGAGGGAGGGAACATAGGGGGAGAGGGAGGGAACAAGGGGGAGAGGGAGGGAACAAGGGGGAGAGAGGGAACAAGGGGGAGAGAGGGAACAAGGGGGAGAGAGGGAACAAGGGGGAGAGGGAGGGAACAAGGGGAGAGGGAGGGAACAAGGGGGAGAGAGAGAACAAGGGAGAAAGGGAGGGAACAAGGNNNANANNNANNNACACANNNGAGAGAGAGAGGGAACAATGAGGGAGGGGAACAAGGGGGAGAGAGGAAGGAGAGCAAGGATTAAAATACTCACTGAGTAAAAGAACGAAGAATGTAGGAAGAGCTTGTTGCTGTTCAGGTTTTAATTCAGAAATGCTTTATGCCAATACAACAACCAAATATTAAGAACAAATCTTCACAAANGAAGGTGGTAGTCACCTGTATGGCAGGACACTGAACAGCTTAGATAAAGTGACTCCAGTGTCCACCTCAACCTGTTCCTATGGTAACACCACAACATAAATATATGAACATCCCTCTCTTCTCATGCCCCACCCACTTGCCTCTTTGGGCATGAAACAATGGAGGACGTGTAACACTTTAATGAACACCTTTATGGTCAGTGCCTGCNNNNNNNNNNNNNNNNNNNNNNNNNNNNNNNNNNNNNNNNNNNNNNNNNNNNNNNNNNNNNNNNNNNNNNNNNNNNNNNNNNNNNNNNNNNNNNNNNNNNNNNNNNNNNNNNNNNNNNNNNNNNNNNNNNNNNNNNNNNNNNNNNNNNNNNNNNNNNNNNNNNNNNNNNNNNNNNNNNNNNNNNNNNNNNNNNNNNNNNNNNNNNNNNNNNNNNNNNNNNNNNNNNNNNNNNNNNNNNNNNNNNNNNNNNNNNNNNNNNNNNNNNNNNNNNNNNNNNNNNNNNNNNNNNNNNNNNNNNNNNNNNNNNNNNNNNNNNNNNNNNNNNNNNNNNNNNNNNNNNNNNNNNNNNNNNNNNNNNNNNNNNNNNNNNNNNNNNNNNNNNNNNNNNNNNNNNNNNNNNNNNNNNNNNNNNNNNNNNNNNNNNNNNNNNNNNNNNNNNNNNNNNNNNNNNNNNNNNNNNNNNNNNNNNNNNNNNNNNNNNNNNNNNNNNNNNNNNNNNNNNNNNNNNNNNNNNNNNNNNNNNNNNNNNNNNNNNNNNNNNNNNNNNNNNNNNNNNNNNNNNNNNNNNNNNNNNNNNNNNNNNNNNNNNNNNNNNNNNNNNNNNNNNNNNNNNNNNNNNNNNNNNNNNNNNNNNNNNNNNNNNNNNNNNNNNNNNNNNNNNNNNNNNNNNNNNNNNNNNNNNNNNNNNNNNNNNNNNNNNNNNNNNNNNNNNNNNNNNNNNNNNNNNNNNNNNNNNNNNNNNNNNNNNNNNNNNNNNNNNNNNNNNNNNNNNNNNNNNNNNNNNNNNNNNNNNNNNNNNNNNNNNNNNNNNNNNNNNNNNNNNNNNNNNNNNNNNNNNNNNNNNNNNNNNNNNNNNNNNNNNNNNNNNNNNNNNNNNNNNNNNNNNNNNNNNNNNNNNNNNNNNNNNNNNNNNNNNNNNNNNNNNNNNNNNNNNNNNNNNNNNNNNNNNNNNNNNNNNNNNNNNNNNNNNNNNNNNNNNNNNNNNNNNNNNNNNNNNNNNNNNNNNNNNNNNNNNNNNNNNNNNNNNNNNNNNNNNNNNNNNNNNNNNNNNNNNNNNNNNNNNNNNNNNNNNNNNNNNNNNNNNNNNNNNNNNNNNNNNNNNNNNNNNNNNNNNNNNNNNNNNNNNNNNNNNNNNNNNNNNNNNNNNNNNNNNNNNNNNNNNNNNNNNNNNNNNNNNNNNNNNNNNNNNNNNNNNNNNNNNNNNNNNNNNNNNNNNNNNNNNNNNNNNNNNNNNNNNNNNNNNNNNNNNNNNNNNNNNNNNNNNNNNNNNNNNNNNNNNNNNNNNNNNNNNNNNNNNNNNNNNNNNNNNNNNNNNNNNNNNNNNNNNNNNNNNNNNNNNNNNNNNNNNNNNNNNNNNNNNNNNNNNNNNNNNNNNNNNNNNNNNNNNNNNNNNNNNNNNNNNNNNNNNNNNNNNNNNNNNNNNNNNNNNNNNNNNNNNNNNNNNNNNNNNNNNNNNNNNNNNNNNNNNNNNNNNNNNNNNNNNNNNNNNNNNNNNNNNNNNNNNNNNNNNNNNNNNNNNNNNNNNNNNNNNNNNNNNNNNNNNNNNNNNNNNNNNNNNNNNNNNNNNNNNNNNNNNNNNNNNNNNNNNNNNNNNNNNNNNNNNNNNNNNNNNNNNNNNNNNNNNNNNNNNNNNNNNNNNNNNNNNNNNNNNNNNNNNNNNNNNNNNNNNNNNNNNNNNNNNNNNNNNNNNNNNNNNNNNNNNNNNNNNNNNNNNNNNNNNNNNNNNNNNNNNNNNNNNNNNNNNNNNNNNNNNNNNNNNNNNNNNNNNNNNNNNNNNNNNNNNNNNNNNNNNNNNNNNNNNNNNNNNNNNNNNNNNNNNNNNNNNNNNNNNNNNNNNNNNNNNNNNNNNNNNNNNNNNNNNNNNNNNNNNNNNNNNNNNNNNNNNNNNNNNNNNNNNNNNNNNNNNNNNNNNNNNNNNNNNNNNNNNNNNNNNNNNNNNNNNNNNNNNNNNNNNNNNNNNNNNNNNNNNNNNNNNNNNNNNNNNNNNNNNNNNNNNNNNNNNNNNNNNNNNNNNNNNNNNNNNNNNNNGAGGGAGGAGGGAGGGTTAGAGAACAAGGTGAAGAAACAAGAGGAAGACAAGTGGTGTCAGTGAAGGCTCATTGGAGTTAAACACATATGGAGGCTCATTGGAGTTAAACACATATGGAGGCTCATTGGAGTTAAACACATATGGAGGCTCATTGGAGTTAAACACANATGGAAGCTCAGTGGGGTTAAACACATACAAAGGCTCATTGGGGTTAAAAATTACCAGTTCCAAATTCTCGCCACACTTCACTGGTCGAAGGGAAACCAAAATGTCCTTGAGCTCAGTACCCATGATCGAGTGGGTGCCCAGTCCTTCAATCAATCTCAACAAATTGTCTGCAACCCAAAGAGAGTTGTTACACCCCACAGGGGGGGTGGGGCACACAGGGNNGNGNGGCNNACTAGGGCCGGGTGGCTAACTANNNNNNNNNNNNNNNNNNNNNNNNNNNNNNNNNNNNNNNNNNNNNNNNNNNNNNNNNNNNNNNNNNNNNNNNNNNNNNNNNNNNNNNNNNNNNNNNNNNNNNNNNNNNNNNNNNNNNNNNNNNNNNNNNNNNNNNNNNNNNNNNNNNNNNNNNNNNNNNNNNNNNNNNNNNNNNNNNNNNNNNNNNNNNNNNNNNNNNNNNNNNNNNNNNNNNNNNNNNNNNNNNNNNNNNNNNNNNNNNNNNNNNNNNNNNNNNNNNNNNNNNNNNNNNNNNNNNNNNNNNNNNNNNNNNNNNNNNNNNNNNNNNNNNNNNNNNNNNNNNNNNNNNNNNNNNNNNNNNNNNNNNNNNNNNNNNNNNNNNNNNNNNNNNNNNNNNNNNNNNNNNNNNNNNNNNNNNNNNNNNNNNNNNNNNNNNNNNNNNNNNNNNNNNNNNNNNNNNNNNNNNNNNNNNNNNNNNNNNNNNNNNNNNNNNNNNNNNNNNNNNNNNNNNNNNNNNNNNNNNNNNNNNNNNNNNNNNNNNNNNNNNNNNNNNNNNNNNNNNNNNNNNNNNNNNNNNNNNNNNNNNNNNNNNNNNNNNNNNNNNNNNNNNNNNNNNNNNNNNNNNNNNNNNNNNNNNNNNNNNNNNNNNNNNNNNNNNNNNNNNNNNNNNNNNNNNNNNNNNNNNNNNNNNNNNNNNNNNNNNNNNNNNNNNNNNNNNNNNNNNNNNNNNNNNNNNNNNNNNNNNNNNNNNNNNNNNNNNNNNNNNNNNNNNNNNNNNNNNNNNNNNNNNNNNNNNNNNNNNNNNNNNNNNNNNNNNNNNNNNNNNNNNNNNNNNNNNNNNNNNNNNNNNNNNNNNNNNNNNNNNNNNNNNNNNNNNNNNNNNNNNNNNNNNNNNNNNNNNNNNNNNNNNNNNNNNNNNNNNNNNNNNNNNNNNNNNNNNNNNNNNNNNNNNNNNNNNNNNNNNNNNNNNNNNNNNNNNNNNNNNNNNNNNNNNNNNNNNNNNNNNNNNTCAAAAGCATTGCGTTCTTTCGTGGACACCCGGTATACAGCACAACAGAGTAGCATTATTAAACATTAACTATTGAATGCCACTGCTCCTTATTTGCCAAGAGGTAATGAACACAAGGACACACCCATAATGAGTAACAGCATTTCTATGACATCATGACGTCAGTAATATCAGACAAGCCCTTTAACTCAATCCTGATTGCACGTAACAGTTTTAAGGCAGAACAGCACTGCCCTTCTGTCCAGTCAAACAGCCCTGTTTCCGTAGGACACAGCACAGAGACACTTACCAGTATTCCAAAATTCACTAGCTTTGGGTCGAGGTGGCTCAATGATGGAATATGCCTCCCTCTGTCTCAAGGGCAGTCCGGCCTGCTCCAGTATGGGGAAGAACACCTCCACTCCACCCAGGGAACTCAAAACCAACTGAAATGGGGGAGGTGGCGTCCATTGGCAGGGCTATGAGNTNTCCCCACCTTTCCTCCTCNCCCTCCCTCCCTTATCGATAATGTACAGGCTTGCCTTGACGTCTCTGATCACATAGACGTCTCCATNCAGTACTGCATCCACAGTCCCNTGCACAGCATGTGACGGAGAGAGGTCCACACACATCTTATCTTTGCATGCCTGTGCACGATGGGACAAGCTAGCTACAAGAGNACCACACTCACTGTTGCCATGACTACCTTCGGATGATAGTAAAAGATCAGCTTAGTCTTCCACTCGTCTGCCTTCGTTTGGAAGATNGACAAATCATTAGGTCCTAAGAATAGCAATTATAATTGATGCCCACCACTTCCCTCGTACCCAGTCCTTACCACGANAGTGGAGAAGCTGGACAGTGGTGTGGTTGAGTGNGTCAGAGAAGAGACACACAGGNCCAATGCAGCCCCCAAAGTTGGTCACAGAGCCAAAGGACGTGATACACTCATCCAGCCGCAAGGGGCTGATGATGTCATGAGGGGTGGGGCTCTCANGGGGAGAGGTGGAGCCCTCTGCGGAGAGCTGATTGTCTGTGCTGTCATGGCTCCAGCCAATCCTGCACTCTGTAAATGTCTGCATGGAGGAAGACATGAGGGAGGAGGAAGGCATGGGGAGGAGGAAGGCATGGGAGGAGGAAGACATGGGGAGGAGGAAGACATGGGGAGGAGGAAGACATGGGGAGGAGGAAGACATGGGGAGGAGGAAGGCGTGGGGAAGAGGAAGGCATGGGGAGGAGGAAGACATGGGGGAGGAGGAAGACATGGGGAGGAGGTNNNNCANTGGGGAGGAGGAAGNCATGGGGGAGGAGGAAGACATGGGGGAGGAGGAAGACATGGGGGGAGAAGGAGAACATGGAGGGAGGAGGAACGGGGGAGGAATGCAAAGGAAGGGATGATAATGGAAGTGTGATAAACCCAGTGTCTACCACTCACATTGGCAAGTGGTACAGCTCTGATCCCATCTGTTGCGGAGATTAGCCCATCCACGAGCACCTGTATGGTGACCTTGTCGCTGTGCTTCTTGCTACTGCTGTACACCACACACACTGAATGCTGGGCACCAGGTAGAGGTAGAGCANAACATAAATACACTCCACCCCTCCCCAATGACNAAAAGAACGCGTACCCATTTCTGTGGAGTGAGTGGCTCTGTGAGAGAAACAGTCTTAAGCCCCCTCTTCTTGGAGTGAACTGCCACCACCAGGACCAGGTCTTTGGTAAAGAACGCTTCAAACCCTGTACTACCAGCATAGAAACTGAAAGTAAGAGACCATTGGGTTATGATATGTAGCCCCTCCTTCTGCTCCACCCACCTGTAAAGCATTCTTCTCATGTTCCTTTGGTCCCCATCTGCACAACTTCGATCCAAACTCACCCAGCAGTGGAAGGAGAAGGCATTTTCTTTGGGCCAGCGATGTAGCTCTGAGATGGATATTTGCTGTANAGAGGATAAGATGATAAGGATGACACTAACACAGAGATAAGATGACAGAGATGACACTAACACAGAGATAAGATAAGGATGACACTAACACAGAGATAAGATAAGGATGACACTAANNNNNNNNNNNNNNNNNNNNNNNNNNNNNNNNNNNNNNNNNNNNNNNNNNNNNNNNNNNNNNNNNNNNNNNNNNNNNNNNNNNNNNNNNNNNNNNNNNNNNNNNNNNNNNNNNNNNNNNNNNNNNNNNNNNNNNNNNNNNNNNNNNNNNNNNNNNNNNNNNNNNNNNNNNNNNNNNNNNNNNNNNNNNNNNNNNNNNNNNNNNNNNNNNNNNNNNNNNNNNNNNNNNNNNNNNNNNNNNNNNNNNNNNNNNNNNNNNNNNNNNNNNNNNNNNNNNNNNNNNNNNNNNNNNNNNNNNNNNNNNNNNNNNNNNNNNNNNNNNNNNNNNNNNNNNNNNNNNNNNNNNNNNNNNNNNNNNNNNNNNNNNNNNNNNNNNNNNNNNNNNNNNNNNNNNNNNNNNNNNNNNNNNNNNNNNNNNNNNNNNNNNNNNNNNNNNNNNNNNNNNNNNNNNNNNNNNNNNNNNNNNNNNNNNNNNNNNNNNNNNNNNNNNNNNNNNNNNNNNNNNNNNNNNNNNNNNNNNNNNNNNNNNNNNNNNNNNNNNNNNNNNNNNNNNNNNNNNNNNNNNNNNNNNNNNNNNNNNNNNNNNNNNNNNNNNNNNNNNNNNNNNNNNNNNNNNNNNNNNNNNNNNNNNNNNNNNNNNNNNNNNNNNNNNNNNNNNNNNNNNNNNNNNNNNNNNNNNNNNNNNNNNNNNNNNNNNNNNNNNNNNNNNNNNNNNNNNNNNNNNNNNNNNNNNNNNNNNNNNNNNNNNNNNNNNNNNNNNNNNNNNNNNNNNNNNNNNNNNNNNNNNNNNNNNNNNNNNNNNNNNNNNNNNNNNNNNNNNNNNNNNNNNNNNNNNNNNNNNNNNNNNNNNNNNNNNNNNNNNNNNNNNNNNNNNNNNNNNNNNNNNNNNNNNNNNNNNNNNNNNNNNNNNNNNNNNNNNNNNNNNNNNNNNNNNNNNNNNNNNNNNNNNNNNNNNNNNNNNNNNNNNNNNNNNNNNNNNNNNNNNNNNNNNNNNNNNNNNNNNNNNNNNNNNNNNNNNNNNNNNNNNNNNNNNNNNNNNNNNNNNNNNNNNNNNNNNNNNNNNNNNNNNNNNNNNNNNNNNNNNNNNNNNNNNNNNNNNNNNNNNNNNNNNNNNNNNNNNNNNNNNNNNNNNNNNNNNNNNNNNNNNNNNNNNNNNNNNNNNNNNNNNNNNNNNNNNNNNNNNNNNNNNNNNNNNNNNNNNNNNNNNNNNNNNNNNNNNNNNNNNNNNNNNNNNNNNNNNNNNNNNNNNNNNNNNNNNNNNNNNNNNNNNNNNNNNNNNNNNNNNNNNNNNNNNNNNNNNNNNNNNNNNNNNNNNNNNNNNNNNNNNNNNNNNNNNNNNNNNNNNNNNNNNNNNNNNNNNNNNNNNNNNNNNNNNNNNNNNNNNNNNNNNNNNNNNNNNNNNNNNNNNNNNNNNNNNNNNNNNNNNNNNNNNNNNNNNNNNNNNNNNNNNNNNNNNNNNNNNNNNNNNNNNNNNNNNNNNNNNNNNNNNNNNNNNNNNNNNNNNNNNNNNNNNNNNNNNNNNNNNNNNNNNNNNNNNNNNNNNNNNNNNNNNNNNNNNNNNNNNNNNNNNNNNNNNNNNNNNNNNNNNNNNNNNNNNNNNNNNNNNNNNNNNNNNNNNNNNNNNNNNNNNNNNNNNNNNNNNNNNNNNNNNNNNNNNNNNNNNNNNNNNNNNNNNNNNNNNNNNNNNNNNNNNNNNNNNNNNNNNNNNNNNNNNNNNNNNNNNNNNNNNNNNNNNNNNNNNNNNNNNNNNNNNNNNNNNNNNNNNNNNNNNNNNNNNNNNNNNNNNNNNNNNNNNNNNNNNNNNNNNNNNNNNNNNNNNNNNNNNNNNNNNNNNNNNNNNNNNNNNNNNNNNNNNNNNNNNNNNNNNNNNNNNNNNNNNNNNNNNNNNNNNNNNNNNNNNNNNNNNNNNNNNNNNNNNNNNNNNNNNNNNNNNNNNNNNNNNNNNNNNNNNNNNNNNNNNNNNNNNNNNNNNNNNNNNNNNNNNNNNNNNNNNNNNNNNNNNNNNNNNNNNNNNNNNNNNNNNNNNNNNNNNNNNNNNNNNNNNNNNNNNNNNNNNNNNNNNNNNNNNNNNNNNNNNNNNNNNNNNNNNNNNNNNNNNNNNNNNNNNNNNNNNNNNNNNNNNNNNNNNNNNNNNNNNNNNNNNNNNNNNNNNNNNNNNNNNNNNNNNNNNNNNNNNNNNNNNNNNNNNNNNNNNNNNNNNNNNNNNNNNNNNNNNNNNNNNNNNNNNNNNNNNNNNNNNNNNNNNNNNNNNNNNNNNNNNNNNNNNNNNNNNNNNNNNNNNNNNNNNNNNNNNNNNNNNNNNNNNNNNNNNNNNNNNNNNNNNNNNNNNNNNNNNNNNNNNNNNNNNNNNNNNNNNNNNNNNNNNNNNNNNNNNNNNNNNNNNNNNNNNNNNNNNNNNNNNNNNNNNNNNNNNNNNNNNNNNNNNNNNNNNNNNNNNNNNNNNNNNNNNNNNNNNNNNNNNNNNNNNNNNNNNNNNNNNNNNNNNNNNNNNNNNNNNNNNNNNNNNNNNNNNNNNNNNNNNNNNNNNNNNNNNNNNNNNNNNNNNNNNNNNNNNNNNNNNNNNNNNNNNNNNNNNNNNNNNNNNNNNNNNNNNNNNNNNNNNNNNNNNNNNNNNNNNNNNNNNNNNNNNNNNNNNNNNNNNNNNNNNNNNNNNNNNNNNNNNNNNNNNNNNNNNNNNNNNNNNNNNNNNNNNNNNNNNNNNNNNNNNNNNNNNNNNNNNNNNNNNNNNNNNNNNNNNNNNNNNNNNNNNNNNNNNNNNNNNNNNNNNNNNNCACACACACACACACACACACATACACACACACACACTTTGAAANAGTCANATANAGAGGGAGTGGTGTGTGGCACACCTTCTGAAGGAGGGCTCCAATTGTAGCTGGTCCCCCAACGACAAAGAAGACCTGCTCATTGATTGATGTCTTGGTCAACATGGAGGTCATCAGTCGAATGAAGTATGTGACTGCCTCATGTTCTGGAAGTGTGCAAATAACAGGTGTGAACTCATGCTTGAGATGGCACTGCACGTAATGTCTGGAGAAGTCTTGATCAACAGACACAACAGTGGATTTCACCTCTAACGCTTACCATTATTCCAAGGTTCAAAGTTCAATGTGGTTCTGGGCACATGATTTATATTTATGGAACATATTTCCCTCTGTNTCAAAGGCAGTCCNGCCTGCTCCAGTATGGGGAAGAACACCTCCACTCCACCCAGGGAACTCAAAACCAACTGAAATGGGGGAGGTGGTGTCAATGAGATACTACTGTCACTCCCTTCCCTCACTCCCCCTTGGGATCAACTGGCTTGCCTGAACATCTCTGGTCACATAGACTTCACCAACCAGTACGCCCTCCATGGCTCCTTCCACTGTGTGTGATGGAGAGAGGTCCACACAGATGTTGNNCTTACAGGCCTGAAGGGAAGAAGAGACCATCAAAGATGGTGCCTAGAACAGAGGGGGATTTCCCCACCTTTGGGTGGAAGAACAGCACCAGTTTACTAATGAGTGCCTCCGTGTCAGGATACTTTGGTTGGAAAAGAGATAGGTTATTGGGTCCTGGCATATGTGGAAAAAGAAACAAAACAGGTCAATAAGCAGCATGTCTNCNNNNANNGATNNANTGGTNNCNNCTCCTACTTCCCCACCCANNNNNNNNNNNNNNNNNNNNNNNNNNNNNNNNNNNNNNNNNNNNNNNNNCCTCCTACTTCCCCACCCACTGTCCACTGTAACCCCTCCTACTTCCCCACCCACTGTCCACAGTAACCCCTCCTACTTCCCCACCCACTGTCCACTGTAACCCCTCCTACTTCCCCACCCACTGTCCACTGTAACCCTCCTACTTCCCCATTACATACACACCATTTCGATACAAAAGCTGTACAGCTTGCAGTTGCAACACTTCTGAGAAGATGTAGACAGAGCTGATCCTGCCAAACAGGCTAATGGCAGGACCAAAGGCTACGGGACTGTCCGCCATTGCCTCAAGCGATTCTGAAAGATCTGCCTCACGTTGGCAAAACCCAATATTGCAATGCTTGTAATCCTGGGGAGGAGGCAGTGTGCCAAGTGAGGATGTGGGGAGGAGAGGGAGAAGGTTGAGAGGAGAGTGAGGAGGTTGAGAGGAGAGGGAGAAGGTTGAGAGGAGAGGGAGGAGGTTGAGAGGAGAGGGAGGAGGTTGAGAGGAGAGGGAGGAGGTTGAGAGGAGAGGGAGGAGGTTGAGAGGAGAGGGAGGAGGTTGAGAGGAGAGGGAGGAAATTAAGAGGAGAGGNAGGAGGTTGAGAGGAGAGGGAGGAGGTTGAGAGNAGAGGGAGGAGGNNGNGTGGTTGAAAGGATTGGGAGGAGGAAGTGCAGGGCACACCAAGTAAAAGAGAGAAATCTGCATGCATGTGACCTTTACTTACGGCAGAAAGGTCTGGTGCTCTGAGATCAGTTGAAAAGGCTTTCTGGATTTCATCGATGTACACAGCAGTCTCATTTTTCCCACCCCAGGGTTTTTTAGAAGCGCTATGCGTCACACATACTGCATGCTAATGGAAGCTCACTGCATTTGATGCTATTGCAGCATGCCAGAACATCACCAGCGATGTCTTACCCATTTCTGAGGACTGAGTGGCTCTTTCACTGAGATGGTTTGAAACCCTCTCTTCTTGGAGTGAACTGCCACCACCAGGACCAGGTCTTTGGTAAAGAACGCTTCAAACCCTGTACTACCAGCATAGAAACTGAAAGAAATAGACCATTGGGTTATGATACGTAGCCCCTCCTTCTGCTCCACCCACCTGTAAAGCATTCTTCTCATGTTCCTCTGGTCCCCATCTGCACAACTTTGATCCAAACTCACCCAGCAGTGGAAGGAGAAGNCATACTGGTTTGGTTGCACGACTTCTGGAACAGCTATATGTATATGCTGTGCATNAAGTATTAGAGAGGAGGGAGAGGAGGGAGAGGAGGGAGAGGAGGGAGAGGAGGGAGAGGGAGGGGAGAGGGGAGGAGGAGGGAGAAGGGAGAGGAGGGGGAAGGGAGAGGAGGGAGGAGGGAGAAGAGGGAGAAGAGGAAGAAGAGGGGGAGGAGGGAGAGGAGGGGGAGGAGGAGGGAGAAGGGAGAGGAGGGGGAGAGGGANNNNNNNNNNNNNNNNNNNNNNNNNNNNNNNNNNNNNNNNNNNNNNNNNNNNNNNNNNNNNNNNNNNNNNNNNNNNNNNNNNNNNNNNNNNNNNNNNNNNNNNNNNNNNNNNNNNNNNNNNNNNNNNNNNNNNNNNNNNNNNNNNNNNNNNNNNNNNNNNNNNNNNNNNNNNNNNNNNNNNNNNNNNNNNNNNNNNNNNNNNNNNNNNNNNNNNNNNNNNNNNNNNNNNNNNNNNNNNNNNNNNNNNNNNNNNNNNNNNNNNNNNNNNNNNNNNNNNNNNNNNNNNNNNNNNNNNNNNNNNNNNNNNNNNNNNNNNNNNNNNNNNNNNNNNNNNNNNNNNNNNNNNNNNNNNNNNNNNNNNNNNNNNNNNNNNNNNNNNNNNNNNNNNNNNNNNNNNNNNNNNNNNNNNNNNNNNNNNNNNNNNNNNNNNNNNNNNNNNNNNNNNNNNNNNNNNNNNNNNNNNNNNNNNNNNNNNNNNNNNNNNNNNNNNNNNNNNNNNNNNNNNNNNNNNNNNNNNNNNNNNNNNNNNNNNNNNNNNNNNNNNNNNNNNNNNNNNNNNNNNNNNNNNNNNNNNNNNNNNNNNNNNNNNNNNNNNNNNNNNNNNNNNNNNNNNNNNNNNNNNNNNNNNNNNNNNNNNNNNNNNNNNNNNNNNNNNNNNNNNNNNNNNNNNNNNNNNNNNNNNNNNNNNNNNNNNNNNNNNNNNNNNNNNNNNNNNNNNNNNNNNNNNNNNNNNNNNNNNNNNNNNNNNNNNNNNNNNNNNNNNNNNNNNNNNNNNNNNNNNNNNNNNNNNNNNNNNNNNNNNNNNNNNNNNNNNNNNAGCCGGACGAGGTCAATCTCCCGACAACAGAGCATATTCTGATGGGTGGGTGTGGTTAGTAGGCCCAGAAGGTACTGCAACATCTCATCTCTCTGAGGGAGGAGAGGGAGGGAGGAGAGGGAGAAGTACTCACCAGAATTTCATCGTTCACTGCACACAGAAACCCTAGAATAGCATCCAATTCGTCACTGAAAATGAATTCTGCAAAGTACAGATCAACAACTGTGGAGAAACAGCAACAGAATCAGTCGATTGTCCCACCATCTGGTACAATGTACACAACACACCATCACATCTGGTACACAACACACAATCACATCTGGTACACAACACATCATCACAGCTGGTACACAGCACACCATCACATCAAGGCAACACAGCTTGGCATAACTACCGGAGATGACTCACTGGACATTAAGGACTTCCTTATATGGACTGTACTCAGCTTTGGATTAATAGCAGATGATGGACTACAAGGGAGGTCATACATTAATTGGAAGACTAGTTAGTAAATGCCCTACTTGTAATACTCTCTGATGATGTCTAAAACAAACTGAACTCCAAAATCTCTCCTACAGTCGTCCAGCTTCAGACTGACTAGGGTTGACAAGTACTTGCTGTGACCTGGGAATGGAAACACAAGAATGGGACAAGAACGGCAAGCATTGGAATGTGACATAAGTGATTGTACAGGTCACTGACTCAGTTGCACAGAGTAGTCTGCTCTGCTCCATATCCTGTAGTTGAACAGCAGTGACGAATAGAGATCAGTCTGCTGCTCTGGCTGGTCTGGACTGAGCTTCTCAACAAATTTTTCGATCGCACTGTGAGCTGCCACAGTCAACAGAGAAGGAGGGACCTGGGCACACACACATATACATATACATACACACATCACACACACACACACACACACACACACANACACACAACAAATTAACTGAATACACATGTTGCTCACCTTACATAGAACATGTAGTCAGGGGCATAAGCATCATTGGGATCAGGGTCCAGCTGGGTCATCAGATCAAATGCTGTCTTCTGGTAAAGCAGAGGAAACCTGTTGTGCATCTCCAACCCTACAGGATGGGATACAGGCCTATCAGNNNNNNNNNNNNNNNNNNNNNNNNNNNNNNNNNNNNNNNNNNNNNNNNNNNNNNNNNNNNNNNNNNNNNNNNNNNNNNNNNNNNNNNNNNNNNNNNNNNNNNNNNNNNNNNNNNNNNNNNNNNNNNNNNNNNNNNNNNNNNNNNNNNNNNNNNNNNNNNNNNNNNNNNNNNNNNNNNNNNNNNNNNNNNNNNNNNNNNNNNNNNNNNNNNNNNNNNNNNNNNNNNNNNNNNNNNNNNNNNNNNNNNNNNNNNNNNNNNNNNNNNNNNNNNNNNNNNNNNNNNNNNNNNNNNNNNNNNNNNNNNNNNNNNNNNNNNNNNNNNNNNNNNNNNNNNNNNNNNNNNNNNNNNNNNNNNNNNNNNNNNNNNNNNNNNNNNNNNNNNNNNNNNNNNNNNNNNNNNNNNNNNNNNNNNNNNNNNNNNNNNNNNNNNNNNNNNNNNNNNNNNNNNNNNNNNNNNNNNNNNNNNNNNNNNNNNNNNNNNNNNNNNNNNNNNNNNNNNNNNNNNNNNNNNNNNNNNNNNNNNNNNNNNNNNNNNNNNNNNNNNNNNNNNNNNNNNNNNNNNNNNNNNNNNNNNNNNNNNNNNNNNNNNNNNNNNNNNNNNNNNNNNNNNNNNNNNNNNNNNNNNNNNNNNNNNNNNNNNNNNNNNNNNNNNNNNNNNNNNNNNNNNNNNNNNNNNNNNNNNNNNNNNNNNNNNNNNNNNNNNNNNNNNNNNNNNNNNNNNNNNNNNNNNNNNNNNNNNNNNNNNNNNNNNNNNNNNNNNNNNNNNNNNNNNNNNNNNNNNNNNNNNNNNNNNNNNNNNNNNNNNNNNNNNNNNNNNNNNNNNNNNNNNNNNNNNNNNNNNNNNNNNNNNNNNNNNNNNNNNNNNNNNNNNNNNNNNNNNNNNNNNNNNGGGACATGTGTACCATCATGTACAACATCATGGACATGTGTACCATCATGTACAACATCATGGGACATGTGTACCATCATGTACAACATCATGGGACATGTGTACCATCATGTACAACATCATGGGACATGTGTACCATCATGTACAACATACCGGGACATTTTTTCAATACAGCTCACCTGTTCTTCCATGCTCCACACTCCCCTATAAGGAACCTTCGTGTGCCACTCCATCTCTGTAAAGTTGCTGATAACTCCTGCCTGTATTGATTGTCTAATGTCTTTTGTCGAGACAGCTCCAATTCCCGCTGTGAGTAAAACTGCTGCGATACTGTTATCTGATATTTTTCAAGATCTGAGAACACTATTTCTTTTCTTTTTGCTTCTGCTTTCAAGCAGTCTTGCTTGCATTGCCTAGTATGGTCCGATAAGACCTTCAACCATTTGTAAACTACCTCAGAGTATTGCATTTGTATAGCCTGAAGCTGTGTTAGAGTGTTGAGAGATGGGAGAGGGCACTCACCCGACAACCTGCTTACCTCACCCAGCCACGTATGCCACTCCTCCCTCTTGGAATATTCAACCAGCTCGATAGTCTCATTGGTGAGATCTGGTAGATAAGAAGGCAAAGGAACCAGCTCCTTATTCCGATACAATTGAAAAAGGTGCTGAAGTAGAGTAGCCCTTGTTGTAGGAGGTTGATCTGAACACACCCAGTATAGAAAATAGGAAGCAAATAGATTAGCATGTTGGAAGAAAAAGACGAGAGGGTAAGAAGGGATAAAGGGAGCAGAACAAAGGAGAAGGAACAGACAGAATCATTCACTGACCACTGCTGAACTGGGTTGCAATGCACCTGAGCACANANACTGNNTCNNGTGCTTCCAGGAATTGAAGTTCTTGCAGCTTACAACCATACTGACAATACTGTGGTACAAAGAGGCCCATAAGAAGGAGAGGGTGAGTAAGGAGAGGGGAAAGGAAGGGGAGGAGAGACAGAATGCATAAAGAGGAGACAGAGCATAGAGTACTAGTAAAATTGTACCACACTGAGATAGCACTGAGAAAACAATATCCACTCACTAAACACCCTCTTCCTCTTACTACTACCCAATCACCTCTGCATCATCAGAGCAAGCACTGGCCAATAAGATTCGCAGCACCGACTGCATCAGGGCTGGTTCTATGGGTCCTTCCAGCACATGAGCTGCTCTCAACATATTCCAGTATTGTTCCAGCAGCTGAAAGGAAAACATTCAAGCCATTGAGGTGTTAAGTTACACTAACATACCTGCATAGAGCAGTGTTCTTTGTTGAGCACCAAGACCCCATTATGAGAGAACTTTTGAAGAATCACTTCTAAATCTGTTGAATGTGAAAGATATCAGGTGTGCAGTAGGGTGGTGTGGTGTGTGCAAGAGTAGTCAGGTGTGCAGTAGGGTGGTGTGGTGTGTGCAAGATATCAGGTGTACAGTAGGATGTGTGGAAGATGTGTACCTTCTACAACATCAAACGAGGGATAGCTTGAGATGATGAAAGCTTCAAGCAGTTGGGATAATTGAAGGATGACAGAGGATAACATGTTTTCAGGAGACCAGCTTGGAACTTGGAAAACAAGGGACCATAGCAGTGATGAACCAATGATGGCCTCCAGCTCCCATGCCTTCTGGCCCAAATCTTTTCCCAATTCCTTCTCCTCTCTCTTCAGCCCCCTTCCAGCAACCACTCTCCTTACCTGAGGAACACTCCAAGGCAACAGCATTCATCAACAGCAAGAAGATTCTAACAAGCAAAGCAACATGAATGAAATATATATAACCATCTCCAGTCCAGTATAAAAAGACATCTGGACCACCCAACAACACAACAAGACATCTGGACCACCCAATAACACAACAAGACATCTGGACTGTACCTTTGTTTGATCAGGTGGACTGGCACTAACAGTCTCTGCTCTGTTGCAAAGAGGTCCAACGCTGCCATAAACTGACCACATACCTATGTGGGAGGGGCCAAAGTGAGGACACACCCACAGACCACACCTACTTTTCTAGTTTGTCCTTCCGACTGCTCTGAGATGATGCAATGCCACAGAATGTAACCAATGGTCTTCATGACAACATCATACGTTTCCGAAGTGGCTGATTCAGCTTCTGTCTGGTTGTCCACGTCCGATTTGCAGGGGGTCAGTAGCCATAGCAACAACCTCTCCCACCCTTTGGACTCTGCCAGCTTGGCCACGTGGCTGGAGTTAAGATGGAGGATGGAGATGAAATCCTGACAAATTGTGTGTCGAATGGACATGGAGGCTGTTCGAAGCAGTCTTAGAGTGGCCAGGTAGACATCAAAGTTGGAGATGCCGCCTGAAACAGAATACGCACATTGCACATGCTGTCTGGTCGGTGTGGTTATCACATTTATGCACATTGCACACATGTTTGTGTTGCCAGCCGTACATGCTCCATTGTTTGTATTACATGTCTACGGGCATTGTATGCTGCACACAGGCACTGTACGTATACTTCACTGCACACGGCACTGTATGCTGCACATGGGCACTGTACATATACTTCACTGCACATGGGCACTGTATNCTGCACATGGGCACTGTATGCTGCACATNGGCACTGTACATATACTTCACTGCACACAGGCACTGTACGTAGCACATACCTGTTTGGTTGTCACTCTTCTTGACCATGCAGAGGTCTAGAAGGGCCAAAATGATGGACGAGGGCAGACACTGCTCATTCTGACACATGGCAGCAGCAAAGGCCCCCGAGTGGAGGGTGTTGTACACCACACCACTCTCAACGCTCCCTTGCTTTAAATTGAGCTGGGTCAGAATCTGCAAGCGTGGGGCGTGAGCACATGGTTTCACCACGCCCCCTCAACAGACCTACCCTCCACCACGCCCCCTCAACACACCCCCTCAACAGACCTACCCTCCACNACAACCCCCTCAAAAGACCTACCCTCAGAGTTGTGAGTCGCACGCCAAAGTCTTGCGTCTGAATTAAAANGAGNAAGACCACACCCTTTTCCCAGAGGGCTGTTGAGAGCATATTTTGATGNGTGGGTGTGGTTAGTAGGCCCAGAAGGAATCGAAGAACCTCATCTAACTGAATAGAGGAGTGGTTTTTTAAAGGAAGAACACACACACACATACATGCACACAACACATACACGCACGCACGCACGCANNNNNNNNNNNNNNNNNNNNNNNNNNNNNNNNNNNNNNNNNNNNNNNNNNNNNNNNNNNNNNNNNNNNNNNNNNNNNNNNNNNNNNNNNNNNNNNNNNNNNNNNNNNNNNNNNNNNNNNNNNNNNNNNNNNNNNNNNNNNNNNNNNNNNNNNNNNNNNNNNNNNNNNNNNNNNNNNNNNNNNNNNNNNNNNNNNNNNNNNNNNNNNNNNNNNNNNNNNNNNNNNNNNNNNNNNNNNNNNNNNNNNNNNNNNNNNNNNNNNNNNNNNNNNNNNNNNNNTGTGTGTGTATGTGTATGTGTATGTGTGCATGTGTGTGTGTGCATGTGGTGGTTCACAGTCATATGTTCATGCATTATCAACAGTCGAGGGCTGGTTTTCTCCATACTTTGTCTGGACCTTTTCCTCCAGGTCAGGGTTCTGTATCCCCATGGGTTTGGACAGGTCTCTGAACACCTCAGAGTTGCTCAGGTCCAATGTATCACAACTGTAGTCTCGCAGGATCCATGGAAACTAAACACGGAGCAGATACTAATTGATAATTTGCATACAGCTCAAGGATCCTCACAATAGGATATTGGTTGAGGTCATTGTAGGTCCTTCCTGATATAGTGTTGAGTTGCATGATGTAATCAAAATTTGTAATTTCACGTTTCACCCATTTCTATAACAAAATAAACCACATCATTCCAAGTGGAGATGAAGAGGGTAAGGAGATGGAGGTCATGGAAGAGGGGAAGGAGATGGAAGGTCATGGAAGAGGGTAAGGAGATGGAAGGTCATGGAAGAGAGTAAGGAGATGGAGGTCATGGAAGAGAGTAAGGAGATGGAAGGTCATGGAAGAGGGGAAGGAGATGGAAGGTCATGGAANNNNNNNNNNNNNNNNNNNNNNNNNNNNNNNNNNNNNNNNNNNNNNNNNNNNNNNNNNNNNNNNNNNNNNNNNNNNNNNNNNNNNNNNNNNNNNNNNNNNNNNNNNNNNNNNNNNNNNNNNNNNNNNNNNNNNNNNNNNNNNNNNNNNNNNNNNNNNNNNNNNNNNNNNNNNNNNNNNNNNNNNNNNNNNNNNNNNNNNNNNNNNNNNNNNNNNNNNNNNNNNNNNNNNNNNNNNNNNNNNNNNNNNNNNNNNNNNNNNNNNNNNNNNNNNNNNNNNNNNNNNNNNNNNNNNNNNNNNNNNNNNNNNNNNNNNNNNNNNNNNNNNNNNNNNNNNNNNNNNNNNNNNNNNNNNNNNNNNNNNNNNNNNNNNNNNNNNNNNNNNNNNNNNNNNNNNNNNNNNNNNNNNNNNNNNNNNNNNNNNNNNNNNNNNNNNNNNNNNNNNNNNNNNNNNNNNNNNNNNNNNNNNNNNNNNNNNNNNNNNNNNNNNNNNNNNNNNNNNNNNNNNNNNNNNNNNNNNNNNNNNNNNNNNNNNNNNNNNNNNNNNNNNNNNNNNNNNNNNNNNNNNNNNNNNNNNNNNNNNNNNNNNNNNNNNNNNNNNNNNNNNNNNNNNNNNNNNNNNNNNNNNNNNNNNNNNNNNNNNNNNNNNNNNNNNNNNNNNNNNNNNNNNNNNNNNNNNNNNNNNNNNNNNNNNNNNNNNNNNNNNNNNNNNNNNNNNNNNNNNNNNNNNNNNNNNNNNNNNNNNNNNNNNNNNNNNNNNNNNNNNNNNNNNNNNNNNNNNNNNNNNNNNNNNNNNNNNNNNNNNNNNNNNNNNNNNNNNNNNNNNNNNNNNNNNNNNNNNNNNNNNNNNNNNNNNNNNNNNNNNNNNNNNNNNNNNNNNNNNNNNNNNNNNNNNNNNNNNNNNNNNNNNNNNNNNNNNNNNNNNNNNNNNNNNNNNNNNNNNNNNNNNNNNNNNNNNNNNNNNNNNNNNNNNNNNNNNNNNNNNNNNNNNNNNNNNNNNNNNNNNNNNNNNNNNNNNNNNNNNNNNNNNNNNNNNNNNNNNNNNNNNNNNNNNNNNNNNNNNNNNNNNNNNNNNNNNNNNNNNNNNNNNNNNNNNNNNNNNNNNNNNNNNNNNNNNNNNNNNNNNNNNNNNNNNNNNNNNNNNNNNNNNNNNNNNNNNNNNNNNNNNNNNNNNNNNNNNNNNNNNNNNNNNNNNNNNNNNNNNNNNNNNNNNNNNNNNNNNNNNNNNNNNNNNNNNNNNNNNNNNNNNNNNNNNNNNNNNNNNNNNNNNNNNNNNNNNNNNNNNNNNNNNNNNNNNNNNNNNNNNNNNNNNNNNNNNNNNNNNNNNNNNNNNNNNNNNNNNNNNNNNNNNNNNNNNNNNNNNNNNNNNNNNNNNNNNNNNNNNNNNNNNNNNNNNNNNNNNNNNNNNNNNNNNNNNNNNNNNNNNNNNNNNNNNNNNNNNNNNNNNNNNNNNNNNNNNNNNNNNNNNNNNNNNNNNNNNNNNNNNNNNNNNNNNNNNNNNNNNNNNNNNNNNNNNNNNNNNNNNNNNNNNNNNNNNNNNNNNNNNNNNNNNNNNNNNNNNNNNNNNNNNNNNNNNNNNNNNNNNNNNNNNNNNNNNNNNNNNNNNNNNNNNNNNNNNNNNNNNNNNNNNNNNNNNNNNNNNNNNNNNNNNNNNNNNNNNNNNNNNNNNNNNNNNNNNNNNNNNNNNNNNNNNNNNNNNNNNNNNNNNNNNNNNNNNNNNNNNNNNNNNNNNNNNNNNNNNNNNNNNNNNNNNNNNNNNNNNNNNNNNNNNNNNNNNNNNNNNNNNNNNNNNNNNNNNNNNNNNNNNNNNNNNNNNNNNNNNNNNNNNNNNNNNNNNNNNNNNNNNNNNNNNNNNNNNNNNNNNNNNNNNNNNNNNNNNNNNNNNNNNNNNNNNNNNNNNNNNNNNNNNNNNNNNNNNNNNNNNNNNNNNNNNNNNNNNNNNNNNNNNNNNNNNNNNNNNNNNNNNNNNNNNNNNNNNNNNNNNNNNNNNNNNNNNNNNNNNNNNNNNNNNNNNNNNNNNNNNNNNNNNNNNNNNNNNNNNNNNNNNNNNNNNNNNNNNNNNNNNNNNNNNNNNNNNNNNNNNNNNNNNNNNNNNNNNNNNNNNNNNNNNNNNNNNNNNNNNNNNNNNNNNNNNNNNNNNNNNNNNNNNNNNNNNNNNNNNNNNNNNNNNNNNNNNNNNNNNNNNNNNNNNNNNNNNNNNNNNNNNNNNNNNNNNNNNNNNNNNNNNNNNNNNNNNNNNNNNNNNNNNNNNNNNNNNNNNNNNNNNNNNNNNNNNNNNNNNNNNNNNNNNNNNNNNNNNNNNNNNNNNNNNNNNNNNNNNNNNNNNNNNNNNNNNNNNNNNNNNNNNNNNNNNNNNNNNNNNNNNNNNNNNNNNNNNNNNNNNNNNNNNNNNNNNNNNNNNNNNNNNNNNNNNNNNNNNNNNNNNNNNNNNNNNNNNNNNNNNNNNNNNNNNNNNNNNNNNNNNNNNNNNNNNNNNNNNNNNNNCATACACACACCATCACACTGTACACACCATCACATGTACACACCATCACACTGTACACACCATCACACTGTACACACCATCACACTGTACACACCATCACACTGTACACACCATCACAACATACCAAGGCTTCTCGATGTTGTCTTATGAAGTCCTCAGGGGACGACGCCCACTTTGGAAGTATCACATCTCCAACAACTGTTTTGTCTTGGGTGACACCAAGGTCATAGCCTATAAAGACAAGTTAGCAGAGTGCTTAGAAATGAATGCAACCCTTTAAGTNATTAACTAGGCAAACANTGATGGNTATTNTAAGCAGTAATTACTGGATCAAAGTCACTTGAAAGTGAGACCAAATATTCAAGATGAACAACAAAAAAAAAANAAANAAAAAAANAAAAAAANAAATTTAGTTGTTAGTCCTTGAATAAATTAATTAATTAACTAGCAATTATTGAAGTTCACATTTTAATTTTTTGAGAGCTTTTTTGAAGCTTGAGGAGTTGGCTTTGTATTTCCCAAATATTTATTAAACCATTCCGCAATGTATTAATTATGAGTACTTATTGGAACAATTGACCTAACCATTTATATTCCTCAAGAAATCCGGTAGGCAGAAAAACTCTGGTGTAAGTTCTTTCACATCACTTCCTAAATAGGTGATTCTCCAAGCATTGGGTATGGAGAAGAACTGTCTATCAGGGTGATCAAACCTAGAGTATTAGCAATATAGCAGAGTGGGTAAGATTGAAGTGAAGATGGGTCAAGATACCATCCCAGCTGTATGTGGCACCTGTTCCCACACAACTCACTTTCCACTCTGTAGGTTGATGTGCAGGGATGTGAAGGGCTNCANTACGNNNNNAGTAGTGGATGACAGTTGTGGCATTGCTGTAGTGTGTNCCNTAGTGAAACTTGCCCATCACTGGATCGTCAAATTCCTCATATCTGCAAGTGCACAGTGCGCATAAGTGTTGTGTGTATAATTGACCACTGTGTCAACACCACATGACACTTTTAAATGGCTGGCGTCCATACTTTGTCCGGACCTTTTCCTCCAGGTCAGGGTTCTGTATCCCCATGGGTTTGGACAGGTCTCTGAACACCTCAGAGTTGCTCAGGTCCAATGTATCACAACTGTAGTCTTGCAGGATCCATGGAAACTAAACACGGAGCAGATACTAAATGATAATTTGCATACAGCTCAAGTATCCTCACAATAGGATATTGGTTGAGGTCATTGTAGGTCCTTCCTGATATAGTGTTGAGTTGCATGATGTAATCAAAATTTGTAATTTCACGTTTCACCCATTTCTACAGTTGTGCAGTTGAATAATGAAAATGAAAGCAGTAGTGAAGGTGGTCATGGAGGAGGTAAGGAATGAGGGAGAGGTGGTCATGGAGGAGGTAAGGGGGAGAGGTGGTCATGGAGGAGGTAAGGGGGAGAGGTGGCCATGGAGGAGGTAAGGATGTAAGGGAGGAGGTGGAGGTGGTGAGGGAGGAGAAAAATTAACACAAGGTGTACAGACAGGGTATAACGACAGTTAGTGTATTCCCAACCTGAATTAGACCTGAAGACTGCATCAGTTTGGCAGGCTCTTGTGTGCTGAAGATGTTAAGGTTTGGAGTACTCAGCGAAACAATGCAGTTGCACACCTTATTCAAAATCTGTGCAACAAGGAGAGAGATACATGCACACCACTTAACACCCAGTCTGGAGACAATGAAAACACTGTGCCTTTTTGGTGGGAAAATTAAGGAAGTAGTTTGTCTGATCCAAAAGAAACATCTCCAGTGCAGTACGGCTTGTGTTGTACCTACGAAGGTGAAGCTCCCTTATCTGGTTAAGTTTGACTGTGAAGTCAGTGAGGTACCCTGCGAAAGAAAGAGAAAATGAATAACCATTGGAGCCTTGCCTTAGGAAGAGGCACTAATAGTTCAATGTCAGATATATCTGGTCACATGTCTTAGCACAAAGTCAACTGTCATACACCTATGCTATTCTAAGTAGTGCACACTCTTGTTACAGATATGCTACTCCATGTTTACAGATATGCTACTCCAAGTGCACACNNTTGTTACAGATATGCTACTCCAAGTGCACACTTTTGTTACAGATATGCTACTACAAGTGCACACTTTTGTTACAGATATGCTACTCCAAGTGCACACTCTTGTTCCTCCCTTCTTCATTTCCTCCTCCCTATCTGTGATCACACCACCCCTTTAACTCTCCCGGTACACTCACAAGATATCGCATCCTTGTCTTGTGCATCCGACACAAAGTAGATGCATCTATTTGTAATGTCCAAGGTTCCAGAGATGACCTCTACCACCGTCACAAGCTGACACATCTCTCTTATNATCAGTTTCTCTTTCTTGCCCTTCTCCAGAGCCACAGCTGGCTCATCTCGAGCTAGCTTTTGGATCTGATCAAACAGATGTTCCTCCTCCTCCTCACTGGNGGACTTTGTAGCTGTGGCCATGGCTAGCAGAGAGGTGTCTGCAGTGCTGGACACCAGGNGAGTCTCCCCAGAGCTGTGTTGACTAGCATCGGTGTGTTTGTTATAGTTGTAGTTACGGGCCAGTTTTAGCCGCATGCGTGAAAAGTTCTCTGTTCTGTCCATCTTCCACAGGTAGAGATCAGGATCTCTGGAACAGAGGATCACGGAATCACAGGGTTATGTACATATACATTCATGTGTGCCACACAGCACCACACAGAGGTACATACATGTATGTACTGTTCCATCATGTATAAAACATGAGTACAACTTCATGTACAACATCATGGGACATGTGTCCCCATGGAACATGTGTACCCATGTACAACATCATGGAACATGTGTACCATCATGTACAACATATGTACCATCAATGTACTANATACTGGCACATAGTGGGGACATGTGTACCATCATGTACNNNNNNNNNNNNNNNNNNNNNNNNNNNNNNNNNNNNNNNNNNNNNNNNNNNNNNNNNNNNNNNNNNNNNNNNNNNNNNNNNNNNNNNNNNNNNNNNNNNNNNNNNNNNNNNNNNNNNNNNNNNNNNNNNNNNNNNNNNNNNNNNNNNNNNNNNNNNNNNNNNNNNNNNNNNNNNNNNNNNNNNNNNNNNNNNNNNNNNNNNNNNNNNNNNNNNNNNNNNNNNNNNNNNNNNNNNNNNNNNNNNNNNNNNNNNNNNNNNNNNNNNNNNNNNNNNNNNNNNNNNNNNNNNNNNNNNNNNNNNNNNNNNNNNNNNNNNNNNNNNNNNNNNNNNNNNNNNNNNNNNNNNNNNNNNNNNNNNNNNNNNNNNNNNNNNNNNNNNNNNNNNNNNNNNNNNNNNNNNNNNNNNNNNNNNNNNNNNNNNNNNNNNNNNNNNNNNNNNNNNNNNNNNNNNNNNNNNNNNNNNNNNNNNNNNNNNNNNNNNNNNNNNNNNNNNNNNNNNNNNNNNNNNNNNNNNNNNNNNNNNNNNNNNNNNNNNNNNNNNNNNNNNNNNNNNNNNNNNNNNNNNNNNNNNNNNNNNNNNNNNNNNNNNNNNNNNNNNNNNNNNNNNNNNNNNNNNNNNNNNNNNNNNNNNNNNNNNNNNNNNNNNNNNNNNNNNNNNNNNNNNNNNNNNNNNNNNNNNNNNNNNNNNNNNNNNNNNNNNNNNNNNNNNNNNNNNNNNNNNNNNNNNNNNNNNNNNNNNNNNNNNNNNNNNNNNNNNNNNNNNNNNNNNNNNNNNNNNNNNNNNNNNNNNNNNNNNNNNNNNNNNNNNNNNNNNNNNNNNNNNNNNNNNNNNNNNNNNNNNNNNNNNNNNNNNNNNNNNNNNNNNNNNNNNNNNNNNNNNNNNNNNNNNNNNNNNNNNNNNNNNNNNNNNNNNNNNNNNNNNNNNNNNNNNNNNNNNNNNNNNNNNNNNNNNNNNNNNNNNNNNNNNNNNNNNNNNNNNNNNNNNNNNNNNNNNNNNNNNNNNNNNNNNNNNNNNNNNNNNNNNNNNNNNNNNNNNNNNNNNNNNNNNNNNNNNNNNNNNNNNNNNNNNNNNNNNNNNNNNNNNNNNNNNNNNNNNNNNNNNNNNNNNNNNNNNNNNNNNNNNNNNNNNNNNNNNNNNNNNNNNNNNNNNNNNNNNNNNNNNNNNNNNNNNNNNNNNNNNNNNNNNNNNNNNNNNNNNNNNNNNNNNNNNNNNNNNNNNNNNNNNNNNNNNNNNNNNNNNNNNNNNNNNNNNNNNNNNNNNNNNNNNNNNNNNNNNNNNNNNNNNNNNNNNNNNNNNNNNNNNNNNNNNNNNNNNNNNNNNNNNNNNNNNNNNNNNNNNNNNNNNNNNNNNNNNNNNNNNNNNNNNNNNNNNNNNNNNNNNNNNNNNNNNNNNNNNNNNNNNNNNNNNNNNNNNNNNNNNNNNNNNNNNNNNNNNNNNNNNNNNNNNNNNNNNNNNNNNNNNNNNNNNNNNNNNNNNNNNNNNNNNNNNNNNNNNNNNNNNNNNNNNNNNNNNNNNNNNNNNNNNNNNNNNNNNNNNNNNNNNNNNNNNNNNNNNNNNNNNNNNNNNNNNNNNNNNNNNNNNNNNNNNNNNNNNNNNNNNNNNNNNNNNNNNNNNNNNNNNNNNNNNNNNNNNNNNNNNNNNNNNNNNNNNNNNNNNNNNNNNNNNNNNNNNNNNNNNNNNNNNNNNNNNNNNNNNNNNNNNNNNNNNNNNNNNNNNNNNNNNNNNNNNNNNNNNNNNNNNNNNNNNNNNNNNNNNNNNNNNNNNNNNNNNNNNNNNNNNNNNNNNNNNNNNNNNNNNNNNNNNNNNNNNNNNNNNNNNNNNNNNNNNNNNNNNNNNNNNNNNNNNNNNNNNNNNNNNNNNNNNNNNNNNNNNNNNNNNNNNNNNNNNNNNNNNNNNNNNNNNNNNNNNNNNNNNNNNNNNNNNNNNNNNNNNNNNNNNNNNNNNNNNNNNNNNNNNNNNNNNNNNNNNNNNNNNNNNNNNNNNNNNNNNNNNNNNNNNNNNNNNNNNNNNNNNNNNNNNNNNNNNNNNNNNNNNNNNNNNNNNNNNNNNNNNNNNNNNNNNNNNNNNNNNNNNNNNNNNNNNNNNNNNNNNNNNNNNNNNNNNNNNNNNNNNNNNNNNNNNNNNNNNNNNNNNNNNNNNNNNNNNNNNNNNNNNNNNNNNNNNNNNNNNNNNNNNNNNNNNNNNNNNNNNNNNNNNNNNNNNNNNNNNNNNNNNNNNNNNNNNNNNNNNNNNNNNNNNNNNNNNNNNNNNNNNNNNNNNNNNNNNNNNNNNNNNNNNNNNNNNNNNNNNNNNNNNNNNNNNNNNNNNNNNNNNNNNNNNNNNNNNNNNNNNNNNNNNNNNNNNNNNNNNNNNNNNNNNNNNNNNNNNNNNNNNNNNNNNNNNNNNNNNNNNNNNNNNNNNNNNNNNNNNNNNNNNNNNNNNNNNNNNNNNNNNNNNNNNNNNNNNNNNNNNNNNNNNNNNNNNNNNNNNNNNNNNNNNNNNNNNNNNNNNNNNNNNNNNNNNNNNNNNNNNNNNNNNNNNNNNNNNNNNNNNNNNNNNNNNNNNNNNNNNNNNNNNNNNNNNNNNNNNNNNNNNNNNNNNNNNNNNNNNNNNNNNNNNNNNNNNNNNNNNNNNNNNNNNNNNNNNNNNNNNNNNNNNNNNNNNNNNNNNNNNNNNNNNNNNNNNNNNNNNNNNNNNNNNNNNNNNNNNNNNNNNNNNNNNNNNNNNNNNNNNNNNNNNNNNNNNNNNNNNNNNNNNNNNNNNNNNNNNNNNNNNNNNNNNNNNNNNNNNNNNNNNNNNNNNNNNNNNNNNNNNNNNNNNNNNNNNNNNNNNNNNNNNNNNNNNNNNNNNNNNNNNNNNNNNNNNNNNNNNNNNNNNNNNNNNNNNNNNNNNNNNNNNNNNNNNNNNNNNNNNNNNNNNNNNNNNNNNNNNNNNNNNNNNNNNNNNNNNNNNNNNNNNNNNNNNNNNNNNNNNNNNNNNNNNNNNNNNNNNNNNNNNNNNNNNNNNNNNNNNNNNNNNNNNNNNNNNNNNNNNNNNNNNNNNNNNNNNNNNNNNNNNNNNNNNNNNNNNNNNNNNNNNNNNNNNNNNNNNNNNNNNNNNNNNNNNNNNNNNNNNNNNNNNNNNNNNNNNNNNNNNNNNNNNNNNNNNNNNNNNNNNNNNNNNNNNNNNNNNNNNNNNNNNNNNNNNNNNNNNNNNNNNNNNNNNNNNNNNNNNNNNNNNNNNNNNNNNNNNNNNNNNNNNNNNNNNNNNNNNNNNNNNNNNNNNNNNNNNNNNNNNNNNNNNNNNNNNNNNNNNNNNNNNNNNNNNNNNNNNNNNNNNNNNNNNNNNNNNNNNNNNNNNNNNNNNNNNNNNNNNNNNNNNNNNNNNNNNNNNNNNNNNNNNNNNNNNNNNNNNNNNNNNNNNNNNNNNNNNNNNNNNNNNNNNNNNNNNNNNNNNNNNNNNNNNNNNNNNNNNNNNNNNNNNNNNNNNNNNNNNNNNNNNNNNNNNNNNNNNNNNNNNNNNNNNNNNNNNNNNNNNNNNNNNNNNNNNNNNNNNNNNNNNNNNNNNNNNNNNNNNNNNNNNNNNNNNNNNNNNNNNNNNNNNNNNNNNNNNNNNNNNNNNNNNNNNNNNNNNNNNNNNNNNNNNNNNNNNNNNNNNNNNNNNNNNNNNNNNNNNNNNNNNNNNNNNNNNNNNNNNNNNNNNNNNNNNNNNNNNNNNNNNNNNNNNNNNNNNNNNNNNNNNNNNNNNNNNNNNNNNNNNNNNNNNNNNNNNNNNNNNNNNNNNNNNNNNNNNNNNNNNNNNNNNNNNNNNNNNNNNNNNNNNNNNNNNNNNNNNNNNNNNNNNNNNNNNNNNNNNNNNNNNNNNNNNNNNNNNNNNNNNNNNNNNNNNNNNNNNNNNNNNNNNNNNNNNNNNNNNNNNNNNNNNNNNNNNNNNNNNNNNNNNNNNNNNNNNNNNNNNNNNNNNNNNNNNNNNNNNNNNNNNNNNNNNNNNNNNNNNNNNNNNNNNNNNNNNNNNNNNNNNNNNNNNNNNNNNNNNNNNNNNNNNNNNNNNNNNNNNNNNNNNNNNNNNNNNNNNNNNNNNNNNNNNNNNNNNNNNNNNNNNNNNNNNNNNNNNNNNNNNNNNNNNNNNNNNNNNNNNNNNNNNNNNNNNNNNNNNNNNNNNNNNNNNNNNNNNNNNNNNNNNNNNNNNNNNNNNNNNNNNNNNNNNNNNNNNNNNNNNNNNNNNNNNNNNNNNNNNNNNNNNNNNNNNNNNNNNNNNNNNNNNNNNNNNNNNNNNNNNNNNNNNNNNNNNNNNNNNNNNNNNNNNNNNNNNNNNNNNNNNNNNNNNNNNNNNNNNNNNNNCCACACACACATACCCCACNCACACACACACACACACACACACACACACCACACACAAACACACACCAATGAAGCAGCTGTGGGGTTGCTCTACAAATGGAGTACCTCTACAACATTAGCCATGAGGTCATCTCTCGTTTGACCTCTTTGATCGTCCCCCTTACGCACCACCTGCATTTGCTCAAAGAGGTTCACTATGACTCTCTTGCCAGTGAAGGGAGAGGAGTAGCCTCTGTAGCTCTTCACAGGCGGGTCTCTTGTCGGATGCCTCTCGTTAAACAGACGAGTTGGTGTCTGACCAAAATTGGTTATAATACTTTCAATCTCTCGGCGGCTTTTTGGGTCTTGGATGTTGTCTAAGTTGACATTTTCTTTATAAGGGAAGTGAAAATATTAACAACATTGAAGATTAGACAATAAGGTCCCAATTATCCATGTACCTTGGTATGAGTACTTGTTAAACACATTCATAGCCTTTTCTGCCTCAGGCCCAGTCTGTTTGTACCCAAAAATGAGGTCAATCCACTCATTGAGGTGTGCTGAAACGTATTCAGANTCCTGAGGGAGGAGGGTAGAGCACAATGNACACACATGCTCCTACNNGNNATTACACTGTACACACCATCACACTGTACACACCATCACAC>scaffold4236|size40413CCACCACCACCACCACCACCACCACCACCACCACCTCCTCCAGCATGGCCGGTATTAACTGCAGAATGGCGGGCACCCTCACTTCCATCCAGCATGAGGCCGAGGTGGAGACCGGGAAGCCGGGGATGTGGGCCAAAGAGGAGACTCAATAGCTGTATGCACAATACTTGATCATTATTGTACTAAATTATCCAAGAGATGGTTATGGTGCATACGAAATGCGCAATATTATCCGTCAGTTTATGCTTGACATAGATGCATGCATGGCATTAAAGTGCCAGTGTTGGCTTTAAGAGACTGTTGGATTGATGTCTGATCGATCTGGAGAACAGGACAAAGAGACAGAATCCAAACGGACTCACAATAGTATATAAAGTGGGTAGTTCCCAATGCAGTGCTACAAGATGACGAGCAAGAGAAGAAGCGTGCGTATGAAGAGAGGGTGAGAGAAATCGAGCATGGCTCCTTTGCACCCCTCGTAGTCTCAATCTCAGGAGGTATGGGTCCGATAGCCACAACCGTGTACAAAAGAATGGCCTCGCTCATAGCTCAGTGAAAAATACAATAATCCATATATCTTTTTTGGCTAAGATGCAAGCTGAGTTTCTCCTTATTGCGATCTGCATAACACATGTGCATCAGAGGCTCGAGGTCCTCCTATCACAGACCAACAAACATGTTTAGTGAGACCATTGTCCTATCCTGCTTGGTAAACAGGATCTCAATTTGAACATTGAAATCCTATCCGTCTTTTTTCTTTTATTNNNTNCTTANNTCATTACCACGTTTCCACGCATTATGTGTATATTTTTATTTAAAAAGCGTATACTTTACGGACTTATCTGCTGTACTGATTTGATTTATTGTCAGTTCAAAGCGATAGTCTCGTACCCAGACCTGACGGACGTAAAAGCATGTTTGCACGTCAGCCGAGTCTGGGTANNNNNNNNNNGGGGGCGGGGCCTAAATATACTACGGCCTGTCCGGGCCATGTAAACTTAAAAGACGTTACGAAAGTGGAAAAAAAAACTTACGAAATAAGTAAATTAAAACATTCTCTATACGCGAAAGTTGGCTCTCCTCCTCTCTTCCTGCCACTCCTCNTACCCCCATTGTCATCTTGTAACCATTACTGCCGATACATACATGATCACTTGCACAAGATTTAGTCACTATAAACTTATTGTGTAATAACTATTGCATAAAGTAGTTAAACGTGGTTAATTTCGGGTTTCCTCCCTCATCTCAAGTCACATGTTCTCATGTTCACGCCACGAGCCAGGCAGAAAACAGCATTTTTATTCATTGTCATTATTTTTATTTTATTAATTTTAGATTANCCATTACAATAAATAAATACATACATAATCAATAAACATTTGAGCACATAATACTACAAACCTACCTACCTAGGACNCNAAGAGTCCTTATCAGTTATTGAAATGACGACGACNNAGCANNNNCNCNNACCAGGGCAATGCTTAGCCTTCCATATAGCCCAGACACTGCTGAAGACATGGGACAAGCAGTTCGAGTGGCGATCCATGTAGCGAGTCTGTCGAGGCACCTGCTGNTANGTGTAAGCTCAGCTGGGGAGTTGGCGTGACCAAAAGCCAAGCTGTGGCGTGGGGAGAAGACACAGAGGGAAACCGNNNNNNNNNNNNNNNNNNNNNNNNNNNNNNNNNNNNNNNNNNNNNNNNNNNNNNNNNNNNNNNNNNNNNNNNNNNNNNNNNNNNNNNNNNNNNNNNNNNNNNNNNNNNNNNNNNNNNNNNNNNNNNNNNNNNNNNNNNNNNNNNNNNNNNNNNNNNNNNNNNNNNNNNNNNNNNNNNNNNNNNNNNNNNNNNNNNNNNNNNNNNNNNNNNNNNNNNNNNNNNNNNNNNNNNNNNNNNNNNNNNNNNNNNNNNNNNNNNNNNNNNNNNNNNNNNNNNNNNNNNNNNNNNNNNNNNNNNNNNNNNNNNNNNNNNNNNNNNNNNNNNNNNNNNNNNNNNNNNNNNNNNNNNNNNNNNNNNNNNNNNNNNNNNNNNNNNNNNNNNNNNNNNNNNNNNNNNNNNNNNNNNNNNNNNNNNNNNNNNNNNNNNNNNNNNNNNNNNNNNNNNNNNNNNNNNNNNNNNNNNNNNNNNNNNNNNNNNNNNNNNNNNNNNNNNNNNNNNNNNNNNNNNNNNNNNNNNNNNNNNNNNNNNNNNNNNNNNNNNNNNNNNNNNNNNNNNNNNNNNNNNNNNNNNNNNNNNNNNNNNNNNNNNNNNNNNNNNNNNNNNNNNNNNNNNNNNNNNNNNNNNNNNNNNNNNNNNNNNNNNNNNNNNNNNNNNNNNNNNNNNNNNNNNNNNNNNNNNNNNNNNNNNNNNNNNNNNNNNNNNNNNNNNNNNNNNNNNNNNNNNNNNNNNNNNNNNNNNNNNNNNNNNNNNNNNNNNNNNNNNNNNNNNNNNNNNNNNNNNNNNNNNNNNNNNNNNNNNNNNNNNNNNNNNNNNNNNNNNNNNNNNNNNNNNNNNNNNNNNNNNNNNNNNNNNNNNNNNNNNNNNNNNNNNNNNNNNNNNNNNNNNNNNNNNNNNNNNNNNNNNNNNNNNNNNNNNNNNNNNNNNNNNNNNNNNNNNNNNNNNNNNNNNNNNNNNNNNNNNNNNNNNNNNNNNNNNNNNNNNNNNNNNNNNNNNNNNNNNNNNNNNNNNNNNNNNNNNNNNNNNNNNNNNNNNNNNNNNNNNNNNNNNNNNNNNNNNNNNNNNNNNNNNNNNNNNNNNNNNNNNNNNNNNNNNNNNNNNNNNNNNNNNNNNNNNNNNNNNNNNNNNNNNNNNNNNNNNNNNNNNNNNNNNNNNNNNNNNNNNNNNNNNNNNNNNNNNNNNNNNNNNNNNNNNNNNNNNNNNNNNNNNNNNNNNNNNNNNNNNNNNNNNNNNNNNNNNNNNNNNNNNNNNNNNNNNNNNNNNNNNNNNNNNNNNNNNNNNNNNNNNNNNNNNNNNNNNNNNNNNNNNNNNNNNNNNNNNNNNNNNNNNNNNNNNNNNNNNNNNNNNNNNNNNNNNNNNNNNNNNNNNNNNNNNNNNNNNNNNNNNNNNNNNNNNNNNNNNNNNNNNNNNNNNNNNNNNNNNNNNNNNNNNNNNNNNNNNNNNNNNNNNNNNNNNNNNNNNNNNNNNNNNNNNNNNNNNNNNNNNNNNNNNNNNNNNNNNNNNNNNNNNNNNNNNNNNNNNNNNNNNNNNNNNNNNNNNNNNNNNNNNNNNNNNNNNNNNNNNNNNNNNNNNNNNNNNNNNNNNNNNNNNNNNNNNNNNNNNNNNNNNNNNNNNNNNNNNNNNNNNNNNNNNNNNNNNNNNNNNNNNNNNNNNNNNNNNNNNNNNNNNNNNNNNNNNNNNNNNNNNNNNNNNNNNNNNNNNNNNNNNNNNNNNNNNNNNNNNNNNNNNNNNNNNNNNNNNNNNNNNNNNNNNNNNNNNNNNNNNNNNNNNNNNNNNNNNNNNNNNNNNNNNNNNNNNNNNNNNNNNNNNNNNNNNNNNNNNNNNNNNNNNNNNNNNNNNNNNNNNNNNNNNNNNNNNNNNNNNNNNNNNNNNNNNNNNNNNNNNNNNNNNNNNNNNNNNNNNNNNNNNNNNNNNNNNNNNNNNNNNNNNNNNNNNNNNNNNNNNNNNNNNNNNNNNNNNNNNNNNNNNNNNNNNNNNNNNNNNNNNNNNNNNNNNNNNNNNNNNNNNNNNNNNNNNNNNNNNNNNNNNNNNNNNNNNNNNNNNNNNNNNNNNNNNNNNNNNNNNNNNNNNNNNNNNNNNNNNNNNNNNNNNNNNNNNNNNNNNNNNNNNNNNNNNNNNNNNNNNNNNNNNNNNNNNNNNNNNNNNNNNNNNNNNNNNNNNNNNNNNNNNNNNNNNNNNNNNNNNNNNNNNNNNNNNNNNNNNNNNNNNNNNNNNNNNNNNNNNNNNNNNNNNNNNNNNNNNNNNNNNNNNNNNNNNNNNNNNNNNNNNNNNNNNNNNNNNNNNNNNNNNNNNNNNNNNNNNNNNNNNNNNNNNNNNNNNNNNNNNNNNNNNNNNNNNNNNNNNNNNNNNNNNNNNNNNNNNNNNNNNNNNNNNNNNNNNNNNNNNNNNNNNNNNNNNNNNNNNNNNNNNNNNNNNNNNNNNNNNNNNNNNNNNNNNNNNNNNNNNNNNNNNNNNNNNNNNNNNNNNNNNNNNNNNNNNNNNNNNNNNNNNNNNNNNNNNNNNNNNNNNNNNNNNNNNNNNNNNNNNNNNNNNNNNNNNNNNNNNNNNNNNNNNNNNNNNNNNNNNNNNNNNNNNNNNNNNNNNNNNNNNNNNNNNNNNNNNNNNNNNNNNNNNNNNNNNNNNNNNNNNNNNNNNNNNNNNNNNNNNNNNNNNNNNNNNNNNNNNNNNNNNNNNNNNNNNNNNNNNNNNNNNNNNNNNNNNNNNNNNNNNNNNNNNNNNNNNNNNNNNNNNNNNNNNNNNNNNNNNNNNNNNNNNNNNNNNNNNNNNNNNNNNNNNNNNNNNNNNNNNNNNNNNNNNNNNNNNNNNNNNNNNNNNNNNNNNNNNNNNNNNNNNNNNNNNNNNNNNNNNNNNNNNNNNNNNNNNNNNNNNNNNNNNNNNNNNNNNNNNNNNNNNNNNNNNNNNNNNNNNNNNNNNNNNNNNNNNNNNNNNNNNNNNNNNNNNNNNNNNNNNNNNNNNNNNNNNNNNNNNNNNNNNNNNNNNNNNNNNNNNNNNNNNNNNNNNNNNNNNNNNNNNNNNNNNNNNNNNNNNNNNNNNNNNNNNNNNNNNNNNNNNNNNNNNNNNNNNNNNNNNNNNNNNNNNNNNNNNNNNNNNNNNNNNNNNNNNNNNNNNNNNNNNNNNNNNNNNNNNNNNNNNNNNNNNNNNNNNNNNNNNNNNNNNNNNNNNNNNNNNNNNNNNNNNNNNNNNNNNNNNNNNNNNNNNNNNNNNNNNNNNNNNNNNNNNNNNNNNNNNNNNNNNNNNNNNNNNNNNNNNNNNNNNNNNNNNNNNNNNNNNNNNNNNNNNNNNNNNNNNNNNNNNNNNNNNNNNNNNNNNNNNNNNNNNNNNNNNNNNNNNNNNNNNNNNNNNNNNNNNNNNNNNNNNNNNNNNNNNNNNNNNNNNNNNNNNNNNNNNNNNNNNNNNNNNNNNNNNNNNNNNNNNNNNNNNNNNNNNNNNNNNNNNNNNNNNNNNNNNNNNNNNNNNNNNNNNNNNNNNNNNNNNNNNNNNNNNNNNNNNNNNNNNNNNNNNNNNNNNNNNNNNNNNNNNNNNNNNNNNNNNNNNCCAGCCAATAACAGCAAGCACACGGGNCTNGNNTNNACNNGTNTATANATNNATGGCCACAAGCTGGCCAACCCAAGGCTGCATTGGAAGCAATAGGGGCAAACACAAGAGGGTGGGGCGTATATCAAAGGGGAGGCCAGTTGCAGAATGTGAACGGGGGAATTTCGAGAGCCTAAACAGTACCATAAGGGTTAGAAGCACTGAGTATGTACAGTGTATGTGTCAACGTTGCTCCATAGATAGTGTGGCCCAAAGGTGGAAGCGGCCAGTCCATACGCAGCACACACACACNCNNNCNNNNNNACNNANAGCAGCATCAGCTGCTCTACGCCTAAACACACTCACGCGACGCTCGGTTGCAATAAGCCGAAAGGATTTCTGTCCAGCGGCAGTGATGGAGGAGGAGTGACACATTTCTTCGTCGCTTTCAACAGACGCTTTAGATAAAAACAGAAAGCCTTGCTTTTTAGTTTAGGGTTCGACTGTTTTGTATACATCGACCACGGCTCCAAGAGCTCTTTAGTTCTGCAAAGATGGGCACACTTATGACTCCATTCACTCGGTCTAACCGACCGTCAAAATATATACAACGTACACAAAACTTACTTGAAAACCTCTTCCGACATGCTGAGACCTGCCCCCTCCTCCTCTGAATGATGTCAGCTTAGGTGCCTCGTTTGCAGGCTCAAATAAATTCATTGCTAAAGTCACGTGCGCAAACTATTTTTCNTTATATAGCTCACAATTAGATCATTTGTAACGCTTCTGTTCTCAAGTAAGCAAAAAATAAAAAAAGAAAGTTCTGTACCCAGACCCAAAAGTAGTAAGGAATGCAATCAGGACCAGTGGGTGTGGCATAAATTCTTTGCCCCCTCCCTATGGAAACGATCTATAGCTGCATGGGATCATGAATCATAAGGTATTCCAAGTATGTAGCNGCGTGAATGACTCTCCTGATATCAGATCCTGCAGACAGACTGAGTTGTACTGTGATCAAGTNGCATGCTTTCATATAATGCATGACCTAATGTGGTGCTCAGCAGGTTCATAACAGAGGGGCTGTGAAGTCTGACACTTCATATCCTTAACAATGGCAAATGAAATGAAACATAGATGGTTATGATAAATTTCTGCACATACATGAATATAAAAATACCCACTAACCACTCCATGCAATAAAGAACAGAAGAGAGGGTACAAAACGAAGGAAGAAACACCTGACACTCACAACATGTCACATGACAACAGCTACGCCGGCAGCTGCCCTGCAAGTCTCCATAGAACTGACCAGATCCCAGCGTGTTGTTTACAGGGTCATACAGTTCTGCTGTTTTCAAGTACGAAGGACCATTGATCCCTCCAACAGCCACCATATGTGCACCAATCGCACATAGNCAGACTCCATCGCGAGCTGAGGTCATTTTGGCTGCTGGATACCATTTGCCACTGCGAACATCGAGAAACTCCACAGAGTCCAGCCGTGAGGTTCCATCGTTACCACCNGCAACAAAAATCTGCTGACCCAAGACTGCCACCCCGATCCCCGCACGAGGTTTCCCCAGCGGGGGCAATGTTCTCCATCGGTTGACATGAGGATCGTAGCACTCCACAACGTTGAGAAACGAGGTCCCATCGTTCCCGCCAAATGTGTACAACTTGTCACCAAAGACAGCTACTCCCGGGAAACTTCTCCGTGAGCTCATCTGAGCGACAGTGACCCAAACGTTCTTTTCAAGGTCGAACCTTTCAACCGTGTTGAGACAGGAGCGACCGTCGTAGCCCCCCGCGGCGTACATGGCGCCTCCCATGACCCCCACGGCCACGCCCGAGCGACACGTGCTCAGGGGGGCCACGTACGTCCAGAACTGAGTGGCCGGATCGAAGCACTCCACGGTGCTGAGGCGCAGAGAGCCGTCCGACCCGCCCACCGCGTACACTTTGCCGTCGAGGACCCCGACGCCCAACTGTTGCCTACGAACCATGAGGGGCGTGCCCTCGCTCCACTTGTTTTCCAGCAGCGAGAACGTCTCGACGGAGTGAGTGATGCTTTCGGACGACTCCTTCCCGCCAACCGCGTACACGACGCCCACCGTGGACTTGCGCGGAAAGAAGCACGGATTGGCCGAACGGATGTCTCTGTCGGCAGGCATTAGGTGGTAGCGTTTCGCGTCGTCAAGAAGGTCCCGACACTCGATGCTCTGCTTCACGATGGGATCACGGTCAACGTCGTTCATCAAACAGTCGACGGCGAGGAGCNNNNNNNNNACGTGCTTCAGCAGCTTTGGCAGCTGTTGCCCGCGCTCGCCCGGGTCCTGCCTGACCCAGCTCATGACAGCTGCGTACACGACCTCTTCCCTGGGTACGTTCAGGTCGTTGCTTGAGATCAGCGAGGCGATACCTTCGGCCGAACTGTGGAAGAACTCGTTGGTGTTGGTCACCTCGAGGAAGTGCCGGCGAGCAAAAGAATCGACCACCTCCGACAGCTGGGTGCAGCCATTGACTTCGGCAAAATTCTTAACACCCAGGCAGTTGCTCACGTCCAAGTGGTTTTTTAGAAATTCGCAGCACACTTCCTGGGCCTGTTTTATCTGCAGCAATGAGGCAGCCGCTAACGTGTCTTGTACATTTTCCACCGTGACTGTTAAGATCCCTGTATAGGCAAAGTCAATCAAACTGCTCAGAACATCTCCACTCATGCCCTTGATTTGAACTACGTCCTGACCGCTCTCCAAAAGTTCATGAGTAAACATGGCCAGAAAGTAGGCACTGCAGGCAGACAGGATGGCTCTGTGGGCATTTATCTCTCTTCCTTCGGCACTCAACGTCACGTCACATAGCTTTTTGTGACTTCGAAGTAAGTGTACCTTTTGAAGAACGCTCTTGCTGTAGTGAAGTGTTGCTTCGTCGGCATCGAAATGTGATGATGGAGCCGTAGAACGATGTGAGTTTTCCTGACGAGATAGAAGCATTGATGTCTCTTTTTCTGCCATTCTCTTTCATTTCATCGTGTCAAATTGACGAATTGAGTCCAAAGTATGAAAATAGATACATTACGTTTCAAATTTTAAAATTACTTGCGATGTATTTTTAGAACAGTGCCGGATGGAATTGATGCAGAAGTCACGTCTAGAGGGACAACCGAGAATCAAATCGCTTATCTCCTTGTCGCTATCGAATGGCAACACAAAAATAAACAGTTTCACGTTGGTTTCATATCACAAAAGATGTGCTTTTAGAGCAAAAAAAAAATGTTTGTGGCAGTTTCTCGTGCATGATCAGTCCCTTGGTCGTCGTCTTCTTCTTCTTCTTCCTTCGTCACGTATAACTAATTAAATTTCTTTCAGCCCTGGTGACCCATTTGAGATCAACAGATCTGATGTACGACTGTACTTAAAAAACAAACAAAAAAACAAAAAAAAACACGCATACATACGTCGCTATTGAATTTATTTGGGAACTGTATCTTACCAGTAGGCCCTAAATCTGACAACTGTAGGTGAAAGAATGCAGCCATTCCCTTTCCTTCCCTTAGTCTCGTCCCCATATTTTATTCCTTTCGCCGTAGCGGTAATGTGCTGCGGTATATTGGGTCTGGGGACGAGACACTACTACCACCACCACCACCACCACCACCACCACCACCACCNNNNNNNNNNNNNNNNNNNNNNNNNNNNNNNNNNNNNNNNNNNNNNNNNNNNNNNNNNNNNNNNNNNNNNNNNNNNNNNNNNNNNNNNNNNNNNNNNNNNNNNNNNNNNNNNNNNNNNNNNNNNNNNNNNNNNNNNNNNNNNNNNNNNNNNNNNNNNNNNNNNNNNNNNNNNNNNNNNNNNNNNNNNNNNNNNNNNNNNNNNNNNNNNNNNNNNNNNNNNNNNNNNNNNNNNNNNNNNNNNNNNNNNNNNNNNNNNNNNNNNNNNNNNNNNNNNNNNNNNNNNNNNNNNNNNNNNNNNNNNNNNNNNNNNNNNNNNNNNNNNNNNNNNNNNNNNNNNNNNNNNNNNNNNNNNNNNNNNNNNNNNNNNNNNNNNNNNNNNNNNNNNNNNNNNNNNNNNNNNNNNNNNNNNNNNNNNNNNNNNNNNNNNNNNNNNNNNNNNNNNNNNNNNNNNNNNNNNNNNNNNNNNNNNNNNNNNNNNNNNNNNNNNNNNNNNNNNNNNNNNNNNNNNNNNNNNNNNNNNNNNNNNNNNNNNNNNNNNNNNNNNNNNNNNNNNNNNNNNNNNNNNNNNNNNNNNNNNNNNNNNNNNNNNNNNNNNNNNNNNNNNNNNNNNNNNNNNNNNNNNNNNNNNNNNNNNNNNNNNNNNNNNNNNNNNNNNNNNNNNNNNNNNNNNNNNNNNNNNNNNNNNNNNNNNNNNNNNNNNNNNNNNNNNNNNNNNNNNNNNNNNNNNNNNNNNNNNNNNNNNNNNNNNNNNNNNNNNNNNNNNNNNNNNNNNNNNNNNNNNNNNNNNNNNNNNNNNNNNNNNNNNNNNNNNNNNNNNNNNNNNNNNNNNNNNNNNNNNNNNNNNNNNNNNNNNNNNNNNNNNNNNNNNNNNNNNNNNNNNNNNNNNNNNNNNNNNNNNNNNNNNNNNNNNNNNNNNNNNNNNNNNNNNNNNNNNNNNNNNNNNNNNNNNNNNNNNNNNNNNNNNNNNNNNNNNNNNNNNNNNNNNNNNNNNNNNNNNNNNNNNNNNNNNNNNNNNNNNNNNNNNNNNNNNNNNNNNNNNNNNNNNNNNNNNNNNNNNNNNNNNNNNNNNNNNNNNNNNNNNNNNNNNNNNNNNNNNNNNNNNNNNNNNNNNNNNNNNNNNNNNNNNNNNNNNNNNNNNNNNNNNNNNNNNNNNNNNNNNNNNNNNNNNNNNNNNNNNNNNNNNNNNNNNNNNNNNNNNNNNNNNNNNNNNNNNNNNNNNNNNNNNNNNNNNNNNNNNNNNNNNNNNNNNNNNNNNNNNNNNNNNNNNNNNNNNNNNNNNNNNNNNNNNNNNNNNNNNNNNNNNNNNNNNNNNNNNNNNNNNNNNNNNNNNNNNNNNNNNNNNNNNNNNNNNNNNNNNNNNNNNNNNNNNNNNNNNNNNNNNNNNNNNNNNNNNNNNNNNNNNNNNNNNNNNNNNNNNNNNNNNNNNNNNNNNNNNNNNNNNNNNNNNNNNNNNNNNNNNNNNNNNNNNNNNNNNNNNNNNNNNNNNNNNNNNNNNNNNNNNNNNNNNNNNNNNNNNNNNNNNNNNNNNNNNNNNNNNNNNNNNNNNNNNNNNNNNNNNNNNNNNNNNNNNNNNNNNNNNNNNNNNNNNNNNNNNNNNNNNNNNNNNNNNNNNNNNNNNNNNNNNNNNNNNNNNNNNNNNNNNNNNNNNNNNNNNNNNNNNNNNNNNNNNNNNNNNNNNNNNNNNNNNNNNNNNNNNNNNNNNNNNNNNNNNNNNNNNNNNNNNNNNNNNNNNNNNNNNNNNNNNNNNNNNNNNNNNNNNNNNNNNNNNNNNNNNNNNNNNNNNNNNNNNNNNNNNNNNNNNNNNNNNNNNNNNNNNNNNNNNNNNNNNNNNNNNNNNNNNNNNNNNNNNNNNNNNNNNNNNNNNNNNNNNNNNNNNNNNNNNNNNNNNNNNNNNNNNNNNNNNNNNNNNNNNNNNNNNNNNNNNNNNNNNNNNNNNNNNNNNNNNNNNNNNNNNNNNNNNNNNNNNNNNNNNNNNNNNNNNNNNNNNNNNNNNNNNNNNNNNNNNNNNNNNNNNNNNNNNNNNNNNNNNNNNNNNNNNNNNNNNNNNNNNNNNNNNNNNNNNNNNNNNNNNNNNNNNNNNNNNNNNNNNNNNNNNNNNNNNNNNNNNNNNNNNNNNNNNNNNNNNNNNNNNNNNNNNNNNNNNNNNNNNNNNNNNNNNNNNNNNNNNNNNNNNNNNNNNNNNNNNNNNNNNNNNNNNNNNNNNNNNNNNNNNNNNNNNNNNNNNNNNNNNNNNNNNNNNNNNNNNNNNNNNNNNNNNNNNNNNNNNNNNNNNNNNNNNNNNNNNNNNNNNNNNNNNNNNNNNNNNNNNNNNNNNNNNNNNNNNNNNNNNNNNNNNNNNNNNNNNNNNNNNNNNNNNNNNNNNNNNNNNNNNNNNNNNNNNNNNNNNNNNNNNNNNNNNNNNNNNNNNNNNNNNNNNNNNNNNNNNNNNNNNNNNNNNNNNNNNNNNNNNNNNNNNNNNNNNNNNNNNNNNNNNNNNNNNNNNNNNNNNNNNNNNNNNNNNNNNNNNNNNNNNNNNNNNNNNNNNNNNNNNNNNNNNNNNNNNATGTGTGTGTGTNTGTGTNTGTGTGTGTGTNTNTGTGTGTGTGAGGATATGTGTAAGAGGAGAGTATTAAATCATGAGTGCATAAAAGCATACAATGGGTTAAAACAATGAGCTGTTAACAAATCATCAAATATACCAGATCTATTTATACAGGAGTACCACAAATATAGAAATGTAAGCAAATTAAACTAGTTACCATGTTCAATAGTTCCTGGAGGACCTCCATAGTAGGCGGGGTAAGAGAAGCCATTAATTTTTTCAAAACACTGTATTTTATGTGCTGTCTGAATGATGCTTTTATAGTTGACGATCCTTTCAGTGCCACAGTGAGAGTCCTTATCAGCAGACATACAAGGGTGGTTCCCAGACCACCAAGGGGAACATTNTTTAGCTCCTTGAAGGACTCCACCAGCTTCTCAAAACAGCCCACTTCTGCAAAGAGCTCCTGTGTGGTTTGGATAACAATGGGTGCTCATGGCTTGGTCGTGTGGCTTTACCTTCAACTGGGTGGAGCCCTTGGGAGCATCCATCAATAGTGAAATAGAGTCTGGAGGAGAGAACCAAGCAATAAGGTGCAAGAAAGGAAGTGAATGATGAGGGAGGTGAACAAGGGGGAGAGGGAGGGAACATAGGGGGAGAGGGAGGGAACAAGGGGGAGAGGGAGGGAACAAGGGGGAGAGAGGGAACAAGGGGGAGAGAGGGAACAAGGGGGAGAGAGGGAACAAGGGGGAGAGGGAGGGAACAAGGGGAGAGGGAGGGAACAAGGGGGAGAGAGAGAACAAGGGAGAAAGGGAGGGAACAAGGNNNANANNNANNNACACANNNGAGAGAGAGAGGGAACAATGAGGGAGGGGAACAAGGGGGAGAGAGGAAGGAGAGCAAGGATTAAAATACTCACTGAGTAAAAGAACGAAGAATGTAGGAAGAGCTTGTTGCTGTTCAGGTTTTAATTCAGAAATGCTTTATGCCAATACAACAACCAAATATTAAGAACAAATCTTCACAAANGAAGGTGGTAGTCACCTGTATGGCAGGACACTGAACAGCTTAGATAAAGTGACTCCAGTGTCCACCTCAACCTGTTCCTATGGTAACACCACAACATAAATATATGAACATCCCTCTCTTCTCATGCCCCACCCACTTGCCTCTTTGGGCATGAAACAATGGAGGACGTGTAACACTTTAATGAACACCTTTATGGTCAGTGCCTGCNNNNNNNNNNNNNNNNNNNNNNNNNNNNNNNNNNNNNNNNNNNNNNNNNNNNNNNNNNNNNNNNNNNNNNNNNNNNNNNNNNNNNNNNNNNNNNNNNNNNNNNNNNNNNNNNNNNNNNNNNNNNNNNNNNNNNNNNNNNNNNNNNNNNNNNNNNNNNNNNNNNNNNNNNNNNNNNNNNNNNNNNNNNNNNNNNNNNNNNNNNNNNNNNNNNNNNNNNNNNNNNNNNNNNNNNNNNNNNNNNNNNNNNNNNNNNNNNNNNNNNNNNNNNNNNNNNNNNNNNNNNNNNNNNNNNNNNNNNNNNNNNNNNNNNNNNNNNNNNNNNNNNNNNNNNNNNNNNNNNNNNNNNNNNNNNNNNNNNNNNNNNNNNNNNNNNNNNNNNNNNNNNNNNNNNNNNNNNNNNNNNNNNNNNNNNNNNNNNNNNNNNNNNNNNNNNNNNNNNNNNNNNNNNNNNNNNNNNNNNNNNNNNNNNNNNNNNNNNNNNNNNNNNNNNNNNNNNNNNNNNNNNNNNNNNNNNNNNNNNNNNNNNNNNNNNNNNNNNNNNNNNNNNNNNNNNNNNNNNNNNNNNNNNNNNNNNNNNNNNNNNNNNNNNNNNNNNNNNNNNNNNNNNNNNNNNNNNNNNNNNNNNNNNNNNNNNNNNNNNNNNNNNNNNNNNNNNNNNNNNNNNNNNNNNNNNNNNNNNNNNNNNNNNNNNNNNNNNNNNNNNNNNNNNNNNNNNNNNNNNNNNNNNNNNNNNNNNNNNNNNNNNNNNNNNNNNNNNNNNNNNNNNNNNNNNNNNNNNNNNNNNNNNNNNNNNNNNNNNNNNNNNNNNNNNNNNNNNNNNNNNNNNNNNNNNNNNNNNNNNNNNNNNNNNNNNNNNNNNNNNNNNNNNNNNNNNNNNNNNNNNNNNNNNNNNNNNNNNNNNNNNNNNNNNNNNNNNNNNNNNNNNNNNNNNNNNNNNNNNNNNNNNNNNNNNNNNNNNNNNNNNNNNNNNNNNNNNNNNNNNNNNNNNNNNNNNNNNNNNNNNNNNNNNNNNNNNNNNNNNNNNNNNNNNNNNNNNNNNNNNNNNNNNNNNNNNNNNNNNNNNNNNNNNNNNNNNNNNNNNNNNNNNNNNNNNNNNNNNNNNNNNNNNNNNNNNNNNNNNNNNNNNNNNNNNNNNNNNNNNNNNNNNNNNNNNNNNNNNNNNNNNNNNNNNNNNNNNNNNNNNNNNNNNNNNNNNNNNNNNNNNNNNNNNNNNNNNNNNNNNNNNNNNNNNNNNNNNNNNNNNNNNNNNNNNNNNNNNNNNNNNNNNNNNNNNNNNNNNNNNNNNNNNNNNNNNNNNNNNNNNNNNNNNNNNNNNNNNNNNNNNNNNNNNNNNNNNNNNNNNNNNNNNNNNNNNNNNNNNNNNNNNNNNNNNNNNNNNNNNNNNNNNNNNNNNNNNNNNNNNNNNNNNNNNNNNNNNNNNNNNNNNNNNNNNNNNNNNNNNNNNNNNNNNNNNNNNNNNNNNNNNNNNNNNNNNNNNNNNNNNNNNNNNNNNNNNNNNNNNNNNNNNNNNNNNNNNNNNNNNNNNNNNNNNNNNNNNNNNNNNNNNNNNNNNNNNNNNNNNNNNNNNNNNNNNNNNNNNNNNNNNNNNNNNNNNNNNNNNNNNNNNNNNNNNNNNNNNNNNNNNNNNNNNNNNNNNNNNNNNNNNNNNNNNNNNNNNNNNNNNNNNNNNNNNNNNNNNNNNNNNNNNNNNNNNNNNNNNNNNNNNNNNNNNNNNNNNNNNNNNNNNNNNNNNNNNNNNNNNNNNNNNNNNNNNNNNNNNNNNNNNNNNNNNNNNNNNNNNNNNNNNNNNNNNNNNNNNNNNNNNNNNNNNNNNNNNNNNNNNNNNNNNNNNNNNNNNNNNNNNNNNNNNNNNNNNNNNNNNNNNNNNNNNNNNNNNNNNNNNNNNNNNNNNNNNNNNNNNNNNNNNNNNNNNNNNNNNNNNNNNNNNNNNNNNNNNNNNNNNNNNNNNNNNNNNNNNNNNNNNNNNNNNNNNNNNNNNNNNNNNNNNNNNNNNNNNNNNNNNNNNNNNNNNNNNNNNNNNNNNNNNNNGAGGGAGGAGGGAGGGTTAGAGAACAAGGTGAAGAAACAAGAGGAAGACAAGTGGTGTCAGTGAAGGCTCATTGGAGTTAAACACATATGGAGGCTCATTGGAGTTAAACACATATGGAGGCTCATTGGAGTTAAACACATATGGAGGCTCATTGGAGTTAAACACANATGGAAGCTCAGTGGGGTTAAACACATACAAAGGCTCATTGGGGTTAAAAATTACCAGTTCCAAATTCTCGCCACACTTCACTGGTCGAAGGGAAACCAAAATGTCCTTGAGCTCAGTACCCATGATCGAGTGGGTGCCCAGTCCTTCAATCAATCTCAACAAATTGTCTGCAACCCAAAGAGAGTTGTTACACCCCACAGGGGGGGTGGGGCACACAGGGNNGNGNGGCNNACTAGGGCCGGGTGGCTAACTANNNNNNNNNNNNNNNNNNNNNNNNNNNNNNNNNNNNNNNNNNNNNNNNNNNNNNNNNNNNNNNNNNNNNNNNNNNNNNNNNNNNNNNNNNNNNNNNNNNNNNNNNNNNNNNNNNNNNNNNNNNNNNNNNNNNNNNNNNNNNNNNNNNNNNNNNNNNNNNNNNNNNNNNNNNNNNNNNNNNNNNNNNNNNNNNNNNNNNNNNNNNNNNNNNNNNNNNNNNNNNNNNNNNNNNNNNNNNNNNNNNNNNNNNNNNNNNNNNNNNNNNNNNNNNNNNNNNNNNNNNNNNNNNNNNNNNNNNNNNNNNNNNNNNNNNNNNNNNNNNNNNNNNNNNNNNNNNNNNNNNNNNNNNNNNNNNNNNNNNNNNNNNNNNNNNNNNNNNNNNNNNNNNNNNNNNNNNNNNNNNNNNNNNNNNNNNNNNNNNNNNNNNNNNNNNNNNNNNNNNNNNNNNNNNNNNNNNNNNNNNNNNNNNNNNNNNNNNNNNNNNNNNNNNNNNNNNNNNNNNNNNNNNNNNNNNNNNNNNNNNNNNNNNNNNNNNNNNNNNNNNNNNNNNNNNNNNNNNNNNNNNNNNNNNNNNNNNNNNNNNNNNNNNNNNNNNNNNNNNNNNNNNNNNNNNNNNNNNNNNNNNNNNNNNNNNNNNNNNNNNNNNNNNNNNNNNNNNNNNNNNNNNNNNNNNNNNNNNNNNNNNNNNNNNNNNNNNNNNNNNNNNNNNNNNNNNNNNNNNNNNNNNNNNNNNNNNNNNNNNNNNNNNNNNNNNNNNNNNNNNNNNNNNNNNNNNNNNNNNNNNNNNNNNNNNNNNNNNNNNNNNNNNNNNNNNNNNNNNNNNNNNNNNNNNNNNNNNNNNNNNNNNNNNNNNNNNNNNNNNNNNNNNNNNNNNNNNNNNNNNNNNNNNNNNNNNNNNNNNNNNNNNNNNNNNNNNNNNNNNNNNNNNNNNNNNNNNNNNNNNNNNNNNNNNNNNNNNNNNNNNNNNNNNNNNNNNNNNNNNNNNNNNTCAAAAGCATTGCGTTCTTTCGTGGACACCCGGTATACAGCACAACAGAGTAGCATTATTAAACATTAACTATTGAATGCCACTGCTCCTTATTTGCCAAGAGGTAATGAACACAAGGACACACCCATAATGAGTAACAGCATTTCTATGACATCATGACGTCAGTAATATCAGACAAGCCCTTTAACTCAATCCTGATTGCACGTAACAGTTTTAAGGCAGAACAGCACTGCCCTTCTGTCCAGTCAAACAGCCCTGTTTCCGTAGGACACAGCACAGAGACACTTACCAGTATTCCAAAATTCACTAGCTTTGGGTCGAGGTGGCTCAATGATGGAATATGCCTCCCTCTGTCTCAAGGGCAGTCCGGCCTGCTCCAGTATGGGGAAGAACACCTCCACTCCACCCAGGGAACTCAAAACCAACTGAAATGGGGGAGGTGGCGTCCATTGGCAGGGCTATGAGNTNTCCCCACCTTTCCTCCTCNCCCTCCCTCCCTTATCGATAATGTACAGGCTTGCCTTGACGTCTCTGATCACATAGACGTCTCCATNCAGTACTGCATCCACAGTCCCNTGCACAGCATGTGACGGAGAGAGGTCCACACACATCTTATCTTTGCATGCCTGTGCACGATGGGACAAGCTAGCTACAAGAGNACCACACTCACTGTTGCCATGACTACCTTCGGATGATAGTAAAAGATCAGCTTAGTCTTCCACTCGTCTGCCTTCGTTTGGAAGATNGACAAATCATTAGGTCCTAAGAATAGCAATTATAATTGATGCCCACCACTTCCCTCGTACCCAGTCCTTACCACGANAGTGGAGAAGCTGGACAGTGGTGTGGTTGAGTGNGTCAGAGAAGAGACACACAGGNCCAATGCAGCCCCCAAAGTTGGTCACAGAGCCAAAGGACGTGATACACTCATCCAGCCGCAAGGGGCTGATGATGTCATGAGGGGTGGGGCTCTCANGGGGAGAGGTGGAGCCCTCTGCGGAGAGCTGATTGTCTGTGCTGTCATGGCTCCAGCCAATCCTGCACTCTGTAAATGTCTGCATGGAGGAAGACATGAGGGAGGAGGAAGGCATGGGGAGGAGGAAGGCATGGGAGGAGGAAGACATGGGGAGGAGGAAGACATGGGGAGGAGGAAGACATGGGGAGGAGGAAGACATGGGGAGGAGGAAGGCGTGGGGAAGAGGAAGGCATGGGGAGGAGGAAGACATGGGGGAGGAGGAAGACATGGGGAGGAGGTNNNNCANTGGGGAGGAGGAAGNCATGGGGGAGGAGGAAGACATGGGGGAGGAGGAAGACATGGGGGGAGAAGGAGAACATGGAGGGAGGAGGAACGGGGGAGGAATGCAAAGGAAGGGATGATAATGGAAGTGTGATAAACCCAGTGTCTACCACTCACATTGGCAAGTGGTACAGCTCTGATCCCATCTGTTGCGGAGATTAGCCCATCCACGAGCACCTGTATGGTGACCTTGTCGCTGTGCTTCTTGCTACTGCTGTACACCACACACACTGAATGCTGGGCACCAGGTAGAGGTAGAGCANAACATAAATACACTCCACCCCTCCCCAATGACNAAAAGAACGCGTACCCATTTCTGTGGAGTGAGTGGCTCTGTGAGAGAAACAGTCTTAAGCCCCCTCTTCTTGGAGTGAACTGCCACCACCAGGACCAGGTCTTTGGTAAAGAACGCTTCAAACCCTGTACTACCAGCATAGAAACTGAAAGTAAGAGACCATTGGGTTATGATATGTAGCCCCTCCTTCTGCTCCACCCACCTGTAAAGCATTCTTCTCATGTTCCTTTGGTCCCCATCTGCACAACTTCGATCCAAACTCACCCAGCAGTGGAAGGAGAAGGCATTTTCTTTGGGCCAGCGATGTAGCTCTGAGATGGATATTTGCTGTANAGAGGATAAGATGATAAGGATGACACTAACACAGAGATAAGATGACAGAGATGACACTAACACAGAGATAAGATAAGGATGACACTAACACAGAGATAAGATAAGGATGACACTAANNNNNNNNNNNNNNNNNNNNNNNNNNNNNNNNNNNNNNNNNNNNNNNNNNNNNNNNNNNNNNNNNNNNNNNNNNNNNNNNNNNNNNNNNNNNNNNNNNNNNNNNNNNNNNNNNNNNNNNNNNNNNNNNNNNNNNNNNNNNNNNNNNNNNNNNNNNNNNNNNNNNNNNNNNNNNNNNNNNNNNNNNNNNNNNNNNNNNNNNNNNNNNNNNNNNNNNNNNNNNNNNNNNNNNNNNNNNNNNNNNNNNNNNNNNNNNNNNNNNNNNNNNNNNNNNNNNNNNNNNNNNNNNNNNNNNNNNNNNNNNNNNNNNNNNNNNNNNNNNNNNNNNNNNNNNNNNNNNNNNNNNNNNNNNNNNNNNNNNNNNNNNNNNNNNNNNNNNNNNNNNNNNNNNNNNNNNNNNNNNNNNNNNNNNNNNNNNNNNNNNNNNNNNNNNNNNNNNNNNNNNNNNNNNNNNNNNNNNNNNNNNNNNNNNNNNNNNNNNNNNNNNNNNNNNNNNNNNNNNNNNNNNNNNNNNNNNNNNNNNNNNNNNNNNNNNNNNNNNNNNNNNNNNNNNNNNNNNNNNNNNNNNNNNNNNNNNNNNNNNNNNNNNNNNNNNNNNNNNNNNNNNNNNNNNNNNNNNNNNNNNNNNNNNNNNNNNNNNNNNNNNNNNNNNNNNNNNNNNNNNNNNNNNNNNNNNNNNNNNNNNNNNNNNNNNNNNNNNNNNNNNNNNNNNNNNNNNNNNNNNNNNNNNNNNNNNNNNNNNNNNNNNNNNNNNNNNNNNNNNNNNNNNNNNNNNNNNNNNNNNNNNNNNNNNNNNNNNNNNNNNNNNNNNNNNNNNNNNNNNNNNNNNNNNNNNNNNNNNNNNNNNNNNNNNNNNNNNNNNNNNNNNNNNNNNNNNNNNNNNNNNNNNNNNNNNNNNNNNNNNNNNNNNNNNNNNNNNNNNNNNNNNNNNNNNNNNNNNNNNNNNNNNNNNNNNNNNNNNNNNNNNNNNNNNNNNNNNNNNNNNNNNNNNNNNNNNNNNNNNNNNNNNNNNNNNNNNNNNNNNNNNNNNNNNNNNNNNNNNNNNNNNNNNNNNNNNNNNNNNNNNNNNNNNNNNNNNNNNNNNNNNNNNNNNNNNNNNNNNNNNNNNNNNNNNNNNNNNNNNNNNNNNNNNNNNNNNNNNNNNNNNNNNNNNNNNNNNNNNNNNNNNNNNNNNNNNNNNNNNNNNNNNNNNNNNNNNNNNNNNNNNNNNNNNNNNNNNNNNNNNNNNNNNNNNNNNNNNNNNNNNNNNNNNNNNNNNNNNNNNNNNNNNNNNNNNNNNNNNNNNNNNNNNNNNNNNNNNNNNNNNNNNNNNNNNNNNNNNNNNNNNNNNNNNNNNNNNNNNNNNNNNNNNNNNNNNNNNNNNNNNNNNNNNNNNNNNNNNNNNNNNNNNNNNNNNNNNNNNNNNNNNNNNNNNNNNNNNNNNNNNNNNNNNNNNNNNNNNNNNNNNNNNNNNNNNNNNNNNNNNNNNNNNNNNNNNNNNNNNNNNNNNNNNNNNNNNNNNNNNNNNNNNNNNNNNNNNNNNNNNNNNNNNNNNNNNNNNNNNNNNNNNNNNNNNNNNNNNNNNNNNNNNNNNNNNNNNNNNNNNNNNNNNNNNNNNNNNNNNNNNNNNNNNNNNNNNNNNNNNNNNNNNNNNNNNNNNNNNNNNNNNNNNNNNNNNNNNNNNNNNNNNNNNNNNNNNNNNNNNNNNNNNNNNNNNNNNNNNNNNNNNNNNNNNNNNNNNNNNNNNNNNNNNNNNNNNNNNNNNNNNNNNNNNNNNNNNNNNNNNNNNNNNNNNNNNNNNNNNNNNNNNNNNNNNNNNNNNNNNNNNNNNNNNNNNNNNNNNNNNNNNNNNNNNNNNNNNNNNNNNNNNNNNNNNNNNNNNNNNNNNNNNNNNNNNNNNNNNNNNNNNNNNNNNNNNNNNNNNNNNNNNNNNNNNNNNNNNNNNNNNNNNNNNNNNNNNNNNNNNNNNNNNNNNNNNNNNNNNNNNNNNNNNNNNNNNNNNNNNNNNNNNNNNNNNNNNNNNNNNNNNNNNNNNNNNNNNNNNNNNNNNNNNNNNNNNNNNNNNNNNNNNNNNNNNNNNNNNNNNNNNNNNNNNNNNNNNNNNNNNNNNNNNNNNNNNNNNNNNNNNNNNNNNNNNNNNNNNNNNNNNNNNNNNNNNNNNNNNNNNNNNNNNNNNNNNNNNNNNNNNNNNNNNNNNNNNNNNNNNNNNNNNNNNNNNNNNNNNNNNNNNNNNNNNNNNNNNNNNNNNNNNNNNNNNNNNNNNNNNNNNNNNNNNNNNNNNNNNNNNNNNNNNNNNNNNNNNNNNNNNNNNNNNNNNNNNNNNNNNNNNNNNNNNNNNNNNNNNNNNNNNNNNNNNNNNNNNNNNNNNNNNNNNNNNNNNNNNNNNNNNNNNNNNNNNNNNNNNNNNNNNNNNNNNNNNNNNNNNNNNNNNNNNNNNNNNNNNNNNNNNNNNNNNNNNNNNNNNNNNNNNNNNNNNNNNNNNNNNNNNNNNNNNNNNNNNNNNNNNNNNNNNNNNNNNNNNNNNNNNNNNNNNNNNNNNNNNNNNNNNNNNNNNNNNNNNNNNNNNNNNNNNNNNNNNNNNNNNNNNNNNNNNNNNNNNNNNNNNNNNNNNNNNNNNNNNNNNNNNNNNNNNNNNNNNNNNNNNNNNNNNNNNNNNNNNNNNNNNNNNNNNNNNNNNNNNNNNNNNNNNNNNNNNNNNNNNNNNNNNNNNNNNNNNNNNNNNNNNNNNNNNNNNNNNNNNNNNNNNNNNNNNNNNNNNNNNNNNNNNNNNNNNNNNNNNNNNNNNNNNNNNNCACACACACACACACACACACATACACACACACACACTTTGAAANAGTCANATANAGAGGGAGTGGTGTGTGGCACACCTTCTGAAGGAGGGCTCCAATTGTAGCTGGTCCCCCAACGACAAAGAAGACCTGCTCATTGATTGATGTCTTGGTCAACATGGAGGTCATCAGTCGAATGAAGTATGTGACTGCCTCATGTTCTGGAAGTGTGCAAATAACAGGTGTGAACTCATGCTTGAGATGGCACTGCACGTAATGTCTGGAGAAGTCTTGATCAACAGACACAACAGTGGATTTCACCTCTAACGCTTACCATTATTCCAAGGTTCAAAGTTCAATGTGGTTCTGGGCACATGATTTATATTTATGGAACATATTTCCCTCTGTNTCAAAGGCAGTCCNGCCTGCTCCAGTATGGGGAAGAACACCTCCACTCCACCCAGGGAACTCAAAACCAACTGAAATGGGGGAGGTGGTGTCAATGAGATACTACTGTCACTCCCTTCCCTCACTCCCCCTTGGGATCAACTGGCTTGCCTGAACATCTCTGGTCACATAGACTTCACCAACCAGTACGCCCTCCATGGCTCCTTCCACTGTGTGTGATGGAGAGAGGTCCACACAGATGTTGNNCTTACAGGCCTGAAGGGAAGAAGAGACCATCAAAGATGGTGCCTAGAACAGAGGGGGATTTCCCCACCTTTGGGTGGAAGAACAGCACCAGTTTACTAATGAGTGCCTCCGTGTCAGGATACTTTGGTTGGAAAAGAGATAGGTTATTGGGTCCTGGCATATGTGGAAAAAGAAACAAAACAGGTCAATAAGCAGCATGTCTNCNNNNANNGATNNANTGGTNNCNNCTCCTACTTCCCCACCCANNNNNNNNNNNNNNNNNNNNNNNNNNNNNNNNNNNNNNNNNNNNNNNNNCCTCCTACTTCCCCACCCACTGTCCACTGTAACCCCTCCTACTTCCCCACCCACTGTCCACAGTAACCCCTCCTACTTCCCCACCCACTGTCCACTGTAACCCCTCCTACTTCCCCACCCACTGTCCACTGTAACCCTCCTACTTCCCCATTACATACACACCATTTCGATACAAAAGCTGTACAGCTTGCAGTTGCAACACTTCTGAGAAGATGTAGACAGAGCTGATCCTGCCAAACAGGCTAATGGCAGGACCAAAGGCTACGGGACTGTCCGCCATTGCCTCAAGCGATTCTGAAAGATCTGCCTCACGTTGGCAAAACCCAATATTGCAATGCTTGTAATCCTGGGGAGGAGGCAGTGTGCCAAGTGAGGATGTGGGGAGGAGAGGGAGAAGGTTGAGAGGAGAGTGAGGAGGTTGAGAGGAGAGGGAGAAGGTTGAGAGGAGAGGGAGGAGGTTGAGAGGAGAGGGAGGAGGTTGAGAGGAGAGGGAGGAGGTTGAGAGGAGAGGGAGGAGGTTGAGAGGAGAGGGAGGAGGTTGAGAGGAGAGGGAGGAAATTAAGAGGAGAGGNAGGAGGTTGAGAGGAGAGGGAGGAGGTTGAGAGNAGAGGGAGGAGGNNGNGTGGTTGAAAGGATTGGGAGGAGGAAGTGCAGGGCACACCAAGTAAAAGAGAGAAATCTGCATGCATGTGACCTTTACTTACGGCAGAAAGGTCTGGTGCTCTGAGATCAGTTGAAAAGGCTTTCTGGATTTCATCGATGTACACAGCAGTCTCATTTTTCCCACCCCAGGGTTTTTTAGAAGCGCTATGCGTCACACATACTGCATGCTAATGGAAGCTCACTGCATTTGATGCTATTGCAGCATGCCAGAACATCACCAGCGATGTCTTACCCATTTCTGAGGACTGAGTGGCTCTTTCACTGAGATGGTTTGAAACCCTCTCTTCTTGGAGTGAACTGCCACCACCAGGACCAGGTCTTTGGTAAAGAACGCTTCAAACCCTGTACTACCAGCATAGAAACTGAAAGAAATAGACCATTGGGTTATGATACGTAGCCCCTCCTTCTGCTCCACCCACCTGTAAAGCATTCTTCTCATGTTCCTCTGGTCCCCATCTGCACAACTTTGATCCAAACTCACCCAGCAGTGGAAGGAGAAGNCATACTGGTTTGGTTGCACGACTTCTGGAACAGCTATATGTATATGCTGTGCATNAAGTATTAGAGAGGAGGGAGAGGAGGGAGAGGAGGGAGAGGAGGGAGAGGAGGGAGAGGGAGGGGAGAGGGGAGGAGGAGGGAGAAGGGAGAGGAGGGGGAAGGGAGAGGAGGGAGGAGGGAGAAGAGGGAGAAGAGGAAGAAGAGGGGGAGGAGGGAGAGGAGGGGGAGGAGGAGGGAGAAGGGAGAGGAGGGGGAGAGGGANNNNNNNNNNNNNNNNNNNNNNNNNNNNNNNNNNNNNNNNNNNNNNNNNNNNNNNNNNNNNNNNNNNNNNNNNNNNNNNNNNNNNNNNNNNNNNNNNNNNNNNNNNNNNNNNNNNNNNNNNNNNNNNNNNNNNNNNNNNNNNNNNNNNNNNNNNNNNNNNNNNNNNNNNNNNNNNNNNNNNNNNNNNNNNNNNNNNNNNNNNNNNNNNNNNNNNNNNNNNNNNNNNNNNNNNNNNNNNNNNNNNNNNNNNNNNNNNNNNNNNNNNNNNNNNNNNNNNNNNNNNNNNNNNNNNNNNNNNNNNNNNNNNNNNNNNNNNNNNNNNNNNNNNNNNNNNNNNNNNNNNNNNNNNNNNNNNNNNNNNNNNNNNNNNNNNNNNNNNNNNNNNNNNNNNNNNNNNNNNNNNNNNNNNNNNNNNNNNNNNNNNNNNNNNNNNNNNNNNNNNNNNNNNNNNNNNNNNNNNNNNNNNNNNNNNNNNNNNNNNNNNNNNNNNNNNNNNNNNNNNNNNNNNNNNNNNNNNNNNNNNNNNNNNNNNNNNNNNNNNNNNNNNNNNNNNNNNNNNNNNNNNNNNNNNNNNNNNNNNNNNNNNNNNNNNNNNNNNNNNNNNNNNNNNNNNNNNNNNNNNNNNNNNNNNNNNNNNNNNNNNNNNNNNNNNNNNNNNNNNNNNNNNNNNNNNNNNNNNNNNNNNNNNNNNNNNNNNNNNNNNNNNNNNNNNNNNNNNNNNNNNNNNNNNNNNNNNNNNNNNNNNNNNNNNNNNNNNNNNNNNNNNNNNNNNNNNNNNNNNNNNNNNNNNNNNNNNNNNNNNNNNNNNNNNNNNNNNAGCCGGACGAGGTCAATCTCCCGACAACAGAGCATATTCTGATGGGTGGGTGTGGTTAGTAGGCCCAGAAGGTACTGCAACATCTCATCTCTCTGAGGGAGGAGAGGGAGGGAGGAGAGGGAGAAGTACTCACCAGAATTTCATCGTTCACTGCACACAGAAACCCTAGAATAGCATCCAATTCGTCACTGAAAATGAATTCTGCAAAGTACAGATCAACAACTGTGGAGAAACAGCAACAGAATCAGTCGATTGTCCCACCATCTGGTACAATGTACACAACACACCATCACATCTGGTACACAACACACAATCACATCTGGTACACAACACATCATCACAGCTGGTACACAGCACACCATCACATCAAGGCAACACAGCTTGGCATAACTACCGGAGATGACTCACTGGACATTAAGGACTTCCTTATATGGACTGTACTCAGCTTTGGATTAATAGCAGATGATGGACTACAAGGGAGGTCATACATTAATTGGAAGACTAGTTAGTAAATGCCCTACTTGTAATACTCTCTGATGATGTCTAAAACAAACTGAACTCCAAAATCTCTCCTACAGTCGTCCAGCTTCAGACTGACTAGGGTTGACAAGTACTTGCTGTGACCTGGGAATGGAAACACAAGAATGGGACAAGAACGGCAAGCATTGGAATGTGACATAAGTGATTGTACAGGTCACTGACTCAGTTGCACAGAGTAGTCTGCTCTGCTCCATATCCTGTAGTTGAACAGCAGTGACGAATAGAGATCAGTCTGCTGCTCTGGCTGGTCTGGACTGAGCTTCTCAACAAATTTTTCGATCGCACTGTGAGCTGCCACAGTCAACAGAGAAGGAGGGACCTGGGCACACACACATATACATATACATACACACATCACACACACACACACACACACACACACANACACACAACAAATTAACTGAATACACATGTTGCTCACCTTACATAGAACATGTAGTCAGGGGCATAAGCATCATTGGGATCAGGGTCCAGCTGGGTCATCAGATCAAATGCTGTCTTCTGGTAAAGCAGAGGAAACCTGTTGTGCATCTCCAACCCTACAGGATGGGATACAGGCCTATCAGNNNNNNNNNNNNNNNNNNNNNNNNNNNNNNNNNNNNNNNNNNNNNNNNNNNNNNNNNNNNNNNNNNNNNNNNNNNNNNNNNNNNNNNNNNNNNNNNNNNNNNNNNNNNNNNNNNNNNNNNNNNNNNNNNNNNNNNNNNNNNNNNNNNNNNNNNNNNNNNNNNNNNNNNNNNNNNNNNNNNNNNNNNNNNNNNNNNNNNNNNNNNNNNNNNNNNNNNNNNNNNNNNNNNNNNNNNNNNNNNNNNNNNNNNNNNNNNNNNNNNNNNNNNNNNNNNNNNNNNNNNNNNNNNNNNNNNNNNNNNNNNNNNNNNNNNNNNNNNNNNNNNNNNNNNNNNNNNNNNNNNNNNNNNNNNNNNNNNNNNNNNNNNNNNNNNNNNNNNNNNNNNNNNNNNNNNNNNNNNNNNNNNNNNNNNNNNNNNNNNNNNNNNNNNNNNNNNNNNNNNNNNNNNNNNNNNNNNNNNNNNNNNNNNNNNNNNNNNNNNNNNNNNNNNNNNNNNNNNNNNNNNNNNNNNNNNNNNNNNNNNNNNNNNNNNNNNNNNNNNNNNNNNNNNNNNNNNNNNNNNNNNNNNNNNNNNNNNNNNNNNNNNNNNNNNNNNNNNNNNNNNNNNNNNNNNNNNNNNNNNNNNNNNNNNNNNNNNNNNNNNNNNNNNNNNNNNNNNNNNNNNNNNNNNNNNNNNNNNNNNNNNNNNNNNNNNNNNNNNNNNNNNNNNNNNNNNNNNNNNNNNNNNNNNNNNNNNNNNNNNNNNNNNNNNNNNNNNNNNNNNNNNNNNNNNNNNNNNNNNNNNNNNNNNNNNNNNNNNNNNNNNNNNNNNNNNNNNNNNNNNNNNNNNNNNNNNNNNNNNNNNNNNNNNNNNNNNNNNNNNNNNNNNNNNNNNNNNNNNNNNNNNNNNGGGACATGTGTACCATCATGTACAACATCATGGACATGTGTACCATCATGTACAACATCATGGGACATGTGTACCATCATGTACAACATCATGGGACATGTGTACCATCATGTACAACATCATGGGACATGTGTACCATCATGTACAACATACCGGGACATTTTTTCAATACAGCTCACCTGTTCTTCCATGCTCCACACTCCCCTATAAGGAACCTTCGTGTGCCACTCCATCTCTGTAAAGTTGCTGATAACTCCTGCCTGTATTGATTGTCTAATGTCTTTTGTCGAGACAGCTCCAATTCCCGCTGTGAGTAAAACTGCTGCGATACTGTTATCTGATATTTTTCAAGATCTGAGAACACTATTTCTTTTCTTTTTGCTTCTGCTTTCAAGCAGTCTTGCTTGCATTGCCTAGTATGGTCCGATAAGACCTTCAACCATTTGTAAACTACCTCAGAGTATTGCATTTGTATAGCCTGAAGCTGTGTTAGAGTGTTGAGAGATGGGAGAGGGCACTCACCCGACAACCTGCTTACCTCACCCAGCCACGTATGCCACTCCTCCCTCTTGGAATATTCAACCAGCTCGATAGTCTCATTGGTGAGATCTGGTAGATAAGAAGGCAAAGGAACCAGCTCCTTATTCCGATACAATTGAAAAAGGTGCTGAAGTAGAGTAGCCCTTGTTGTAGGAGGTTGATCTGAACACACCCAGTATAGAAAATAGGAAGCAAATAGATTAGCATGTTGGAAGAAAAAGACGAGAGGGTAAGAAGGGATAAAGGGAGCAGAACAAAGGAGAAGGAACAGACAGAATCATTCACTGACCACTGCTGAACTGGGTTGCAATGCACCTGAGCACANANACTGNNTCNNGTGCTTCCAGGAATTGAAGTTCTTGCAGCTTACAACCATACTGACAATACTGTGGTACAAAGAGGCCCATAAGAAGGAGAGGGTGAGTAAGGAGAGGGGAAAGGAAGGGGAGGAGAGACAGAATGCATAAAGAGGAGACAGAGCATAGAGTACTAGTAAAATTGTACCACACTGAGATAGCACTGAGAAAACAATATCCACTCACTAAACACCCTCTTCCTCTTACTACTACCCAATCACCTCTGCATCATCAGAGCAAGCACTGGCCAATAAGATTCGCAGCACCGACTGCATCAGGGCTGGTTCTATGGGTCCTTCCAGCACATGAGCTGCTCTCAACATATTCCAGTATTGTTCCAGCAGCTGAAAGGAAAACATTCAAGCCATTGAGGTGTTAAGTTACACTAACATACCTGCATAGAGCAGTGTTCTTTGTTGAGCACCAAGACCCCATTATGAGAGAACTTTTGAAGAATCACTTCTAAATCTGTTGAATGTGAAAGATATCAGGTGTGCAGTAGGGTGGTGTGGTGTGTGCAAGAGTAGTCAGGTGTGCAGTAGGGTGGTGTGGTGTGTGCAAGATATCAGGTGTACAGTAGGATGTGTGGAAGATGTGTACCTTCTACAACATCAAACGAGGGATAGCTTGAGATGATGAAAGCTTCAAGCAGTTGGGATAATTGAAGGATGACAGAGGATAACATGTTTTCAGGAGACCAGCTTGGAACTTGGAAAACAAGGGACCATAGCAGTGATGAACCAATGATGGCCTCCAGCTCCCATGCCTTCTGGCCCAAATCTTTTCCCAATTCCTTCTCCTCTCTCTTCAGCCCCCTTCCAGCAACCACTCTCCTTACCTGAGGAACACTCCAAGGCAACAGCATTCATCAACAGCAAGAAGATTCTAACAAGCAAAGCAACATGAATGAAATATATATAACCATCTCCAGTCCAGTATAAAAAGACATCTGGACCACCCAACAACACAACAAGACATCTGGACCACCCAATAACACAACAAGACATCTGGACTGTACCTTTGTTTGATCAGGTGGACTGGCACTAACAGTCTCTGCTCTGTTGCAAAGAGGTCCAACGCTGCCATAAACTGACCACATACCTATGTGGGAGGGGCCAAAGTGAGGACACACCCACAGACCACACCTACTTTTCTAGTTTGTCCTTCCGACTGCTCTGAGATGATGCAATGCCACAGAATGTAACCAATGGTCTTCATGACAACATCATACGTTTCCGAAGTGGCTGATTCAGCTTCTGTCTGGTTGTCCACGTCCGATTTGCAGGGGGTCAGTAGCCATAGCAACAACCTCTCCCACCCTTTGGACTCTGCCAGCTTGGCCACGTGGCTGGAGTTAAGATGGAGGATGGAGATGAAATCCTGACAAATTGTGTGTCGAATGGACATGGAGGCTGTTCGAAGCAGTCTTAGAGTGGCCAGGTAGACATCAAAGTTGGAGATGCCGCCTGAAACAGAATACGCACATTGCACATGCTGTCTGGTCGGTGTGGTTATCACATTTATGCACATTGCACACATGTTTGTGTTGCCAGCCGTACATGCTCCATTGTTTGTATTACATGTCTACGGGCATTGTATGCTGCACACAGGCACTGTACGTATACTTCACTGCACACGGCACTGTATGCTGCACATGGGCACTGTACATATACTTCACTGCACATGGGCACTGTATNCTGCACATGGGCACTGTATGCTGCACATNGGCACTGTACATATACTTCACTGCACACAGGCACTGTACGTAGCACATACCTGTTTGGTTGTCACTCTTCTTGACCATGCAGAGGTCTAGAAGGGCCAAAATGATGGACGAGGGCAGACACTGCTCATTCTGACACATGGCAGCAGCAAAGGCCCCCGAGTGGAGGGTGTTGTACACCACACCACTCTCAACGCTCCCTTGCTTTAAATTGAGCTGGGTCAGAATCTGCAAGCGTGGGGCGTGAGCACATGGTTTCACCACGCCCCCTCAACAGACCTACCCTCCACCACGCCCCCTCAACACACCCCCTCAACAGACCTACCCTCCACNACAACCCCCTCAAAAGACCTACCCTCAGAGTTGTGAGTCGCACGCCAAAGTCTTGCGTCTGAATTAAAANGAGNAAGACCACACCCTTTTCCCAGAGGGCTGTTGAGAGCATATTTTGATGNGTGGGTGTGGTTAGTAGGCCCAGAAGGAATCGAAGAACCTCATCTAACTGAATAGAGGAGTGGTTTTTTAAAGGAAGAACACACACACACATACATGCACACAACACATACACGCACGCACGCACGCANNNNNNNNNNNNNNNNNNNNNNNNNNNNNNNNNNNNNNNNNNNNNNNNNNNNNNNNNNNNNNNNNNNNNNNNNNNNNNNNNNNNNNNNNNNNNNNNNNNNNNNNNNNNNNNNNNNNNNNNNNNNNNNNNNNNNNNNNNNNNNNNNNNNNNNNNNNNNNNNNNNNNNNNNNNNNNNNNNNNNNNNNNNNNNNNNNNNNNNNNNNNNNNNNNNNNNNNNNNNNNNNNNNNNNNNNNNNNNNNNNNNNNNNNNNNNNNNNNNNNNNNNNNNNNNNNNNNNTGTGTGTGTATGTGTATGTGTATGTGTGCATGTGTGTGTGTGCATGTGGTGGTTCACAGTCATATGTTCATGCATTATCAACAGTCGAGGGCTGGTTTTCTCCATACTTTGTCTGGACCTTTTCCTCCAGGTCAGGGTTCTGTATCCCCATGGGTTTGGACAGGTCTCTGAACACCTCAGAGTTGCTCAGGTCCAATGTATCACAACTGTAGTCTCGCAGGATCCATGGAAACTAAACACGGAGCAGATACTAATTGATAATTTGCATACAGCTCAAGGATCCTCACAATAGGATATTGGTTGAGGTCATTGTAGGTCCTTCCTGATATAGTGTTGAGTTGCATGATGTAATCAAAATTTGTAATTTCACGTTTCACCCATTTCTATAACAAAATAAACCACATCATTCCAAGTGGAGATGAAGAGGGTAAGGAGATGGAGGTCATGGAAGAGGGGAAGGAGATGGAAGGTCATGGAAGAGGGTAAGGAGATGGAAGGTCATGGAAGAGAGTAAGGAGATGGAGGTCATGGAAGAGAGTAAGGAGATGGAAGGTCATGGAAGAGGGGAAGGAGATGGAAGGTCATGGAANNNNNNNNNNNNNNNNNNNNNNNNNNNNNNNNNNNNNNNNNNNNNNNNNNNNNNNNNNNNNNNNNNNNNNNNNNNNNNNNNNNNNNNNNNNNNNNNNNNNNNNNNNNNNNNNNNNNNNNNNNNNNNNNNNNNNNNNNNNNNNNNNNNNNNNNNNNNNNNNNNNNNNNNNNNNNNNNNNNNNNNNNNNNNNNNNNNNNNNNNNNNNNNNNNNNNNNNNNNNNNNNNNNNNNNNNNNNNNNNNNNNNNNNNNNNNNNNNNNNNNNNNNNNNNNNNNNNNNNNNNNNNNNNNNNNNNNNNNNNNNNNNNNNNNNNNNNNNNNNNNNNNNNNNNNNNNNNNNNNNNNNNNNNNNNNNNNNNNNNNNNNNNNNNNNNNNNNNNNNNNNNNNNNNNNNNNNNNNNNNNNNNNNNNNNNNNNNNNNNNNNNNNNNNNNNNNNNNNNNNNNNNNNNNNNNNNNNNNNNNNNNNNNNNNNNNNNNNNNNNNNNNNNNNNNNNNNNNNNNNNNNNNNNNNNNNNNNNNNNNNNNNNNNNNNNNNNNNNNNNNNNNNNNNNNNNNNNNNNNNNNNNNNNNNNNNNNNNNNNNNNNNNNNNNNNNNNNNNNNNNNNNNNNNNNNNNNNNNNNNNNNNNNNNNNNNNNNNNNNNNNNNNNNNNNNNNNNNNNNNNNNNNNNNNNNNNNNNNNNNNNNNNNNNNNNNNNNNNNNNNNNNNNNNNNNNNNNNNNNNNNNNNNNNNNNNNNNNNNNNNNNNNNNNNNNNNNNNNNNNNNNNNNNNNNNNNNNNNNNNNNNNNNNNNNNNNNNNNNNNNNNNNNNNNNNNNNNNNNNNNNNNNNNNNNNNNNNNNNNNNNNNNNNNNNNNNNNNNNNNNNNNNNNNNNNNNNNNNNNNNNNNNNNNNNNNNNNNNNNNNNNNNNNNNNNNNNNNNNNNNNNNNNNNNNNNNNNNNNNNNNNNNNNNNNNNNNNNNNNNNNNNNNNNNNNNNNNNNNNNNNNNNNNNNNNNNNNNNNNNNNNNNNNNNNNNNNNNNNNNNNNNNNNNNNNNNNNNNNNNNNNNNNNNNNNNNNNNNNNNNNNNNNNNNNNNNNNNNNNNNNNNNNNNNNNNNNNNNNNNNNNNNNNNNNNNNNNNNNNNNNNNNNNNNNNNNNNNNNNNNNNNNNNNNNNNNNNNNNNNNNNNNNNNNNNNNNNNNNNNNNNNNNNNNNNNNNNNNNNNNNNNNNNNNNNNNNNNNNNNNNNNNNNNNNNNNNNNNNNNNNNNNNNNNNNNNNNNNNNNNNNNNNNNNNNNNNNNNNNNNNNNNNNNNNNNNNNNNNNNNNNNNNNNNNNNNNNNNNNNNNNNNNNNNNNNNNNNNNNNNNNNNNNNNNNNNNNNNNNNNNNNNNNNNNNNNNNNNNNNNNNNNNNNNNNNNNNNNNNNNNNNNNNNNNNNNNNNNNNNNNNNNNNNNNNNNNNNNNNNNNNNNNNNNNNNNNNNNNNNNNNNNNNNNNNNNNNNNNNNNNNNNNNNNNNNNNNNNNNNNNNNNNNNNNNNNNNNNNNNNNNNNNNNNNNNNNNNNNNNNNNNNNNNNNNNNNNNNNNNNNNNNNNNNNNNNNNNNNNNNNNNNNNNNNNNNNNNNNNNNNNNNNNNNNNNNNNNNNNNNNNNNNNNNNNNNNNNNNNNNNNNNNNNNNNNNNNNNNNNNNNNNNNNNNNNNNNNNNNNNNNNNNNNNNNNNNNNNNNNNNNNNNNNNNNNNNNNNNNNNNNNNNNNNNNNNNNNNNNNNNNNNNNNNNNNNNNNNNNNNNNNNNNNNNNNNNNNNNNNNNNNNNNNNNNNNNNNNNNNNNNNNNNNNNNNNNNNNNNNNNNNNNNNNNNNNNNNNNNNNNNNNNNNNNNNNNNNNNNNNNNNNNNNNNNNNNNNNNNNNNNNNNNNNNNNNNNNNNNNNNNNNNNNNNNNNNNNNNNNNNNNNNNNNNNNNNNNNNNNNNNNNNNNNNNNNNNNNNNNNNNNNNNNNNNNNNNNNNNNNNNNNNNNNNNNNNNNNNNNNNNNNNNNNNNNNNNNNNNNNNNNNNNNNNNNNNNNNNNNNNNNNNNNNNNNNNNNNNNNNNNNNNNNNNNNNNNNNNNNNNNNNNNNNNNNNNNNNNNNNNNNNNNNNNNNNNNNNNNNNNNNNNNNNNNNNNNNNNNNNNNNNNNNNNNNNNNNNNNNNNNNNNNNNNNNNNNNNNNNNNNNNNNNNNNNNNNNNNNNNNNNNNNNNNNNNNNNNNNNNNNNNNNNNNNNNNNNNNNNNNNNNNNNNNNNNNNNNNNNNNNNNNNNNNNNNNNNNNNNNNNNNNNNNNNNNNNNNNNNNNNNNNNNNNNNNNNNNNNNNNNNNNNNNNNNNNNNNNNNNNNNNNNNNNNNNNNNNNNNNNNNNNNNNNNNNNNNNNNNNNNNNNNNNNNNNNNNNNNNNNNNNNNNNNNNNNNNNNNNNNNNNNNNNNNNNNNNNNNNNNNNNNNNNNNNNNNNNNNNNNNNNNNNNNNNNNNNNNNNCATACACACACCATCACACTGTACACACCATCACATGTACACACCATCACACTGTACACACCATCACACTGTACACACCATCACACTGTACACACCATCACACTGTACACACCATCACAACATACCAAGGCTTCTCGATGTTGTCTTATGAAGTCCTCAGGGGACGACGCCCACTTTGGAAGTATCACATCTCCAACAACTGTTTTGTCTTGGGTGACACCAAGGTCATAGCCTATAAAGACAAGTTAGCAGAGTGCTTAGAAATGAATGCAACCCTTTAAGTNATTAACTAGGCAAACANTGATGGNTATTNTAAGCAGTAATTACTGGATCAAAGTCACTTGAAAGTGAGACCAAATATTCAAGATGAACAACAAAAAAAAAANAAANAAAAAAANAAAAAAANAAATTTAGTTGTTAGTCCTTGAATAAATTAATTAATTAACTAGCAATTATTGAAGTTCACATTTTAATTTTTTGAGAGCTTTTTTGAAGCTTGAGGAGTTGGCTTTGTATTTCCCAAATATTTATTAAACCATTCCGCAATGTATTAATTATGAGTACTTATTGGAACAATTGACCTAACCATTTATATTCCTCAAGAAATCCGGTAGGCAGAAAAACTCTGGTGTAAGTTCTTTCACATCACTTCCTAAATAGGTGATTCTCCAAGCATTGGGTATGGAGAAGAACTGTCTATCAGGGTGATCAAACCTAGAGTATTAGCAATATAGCAGAGTGGGTAAGATTGAAGTGAAGATGGGTCAAGATACCATCCCAGCTGTATGTGGCACCTGTTCCCACACAACTCACTTTCCACTCTGTAGGTTGATGTGCAGGGATGTGAAGGGCTNCANTACGNNNNNAGTAGTGGATGACAGTTGTGGCATTGCTGTAGTGTGTNCCNTAGTGAAACTTGCCCATCACTGGATCGTCAAATTCCTCATATCTGCAAGTGCACAGTGCGCATAAGTGTTGTGTGTATAATTGACCACTGTGTCAACACCACATGACACTTTTAAATGGCTGGCGTCCATACTTTGTCCGGACCTTTTCCTCCAGGTCAGGGTTCTGTATCCCCATGGGTTTGGACAGGTCTCTGAACACCTCAGAGTTGCTCAGGTCCAATGTATCACAACTGTAGTCTTGCAGGATCCATGGAAACTAAACACGGAGCAGATACTAAATGATAATTTGCATACAGCTCAAGTATCCTCACAATAGGATATTGGTTGAGGTCATTGTAGGTCCTTCCTGATATAGTGTTGAGTTGCATGATGTAATCAAAATTTGTAATTTCACGTTTCACCCATTTCTACAGTTGTGCAGTTGAATAATGAAAATGAAAGCAGTAGTGAAGGTGGTCATGGAGGAGGTAAGGAATGAGGGAGAGGTGGTCATGGAGGAGGTAAGGGGGAGAGGTGGTCATGGAGGAGGTAAGGGGGAGAGGTGGCCATGGAGGAGGTAAGGATGTAAGGGAGGAGGTGGAGGTGGTGAGGGAGGAGAAAAATTAACACAAGGTGTACAGACAGGGTATAACGACAGTTAGTGTATTCCCAACCTGAATTAGACCTGAAGACTGCATCAGTTTGGCAGGCTCTTGTGTGCTGAAGATGTTAAGGTTTGGAGTACTCAGCGAAACAATGCAGTTGCACACCTTATTCAAAATCTGTGCAACAAGGAGAGAGATACATGCACACCACTTAACACCCAGTCTGGAGACAATGAAAACACTGTGCCTTTTTGGTGGGAAAATTAAGGAAGTAGTTTGTCTGATCCAAAAGAAACATCTCCAGTGCAGTACGGCTTGTGTTGTACCTACGAAGGTGAAGCTCCCTTATCTGGTTAAGTTTGACTGTGAAGTCAGTGAGGTACCCTGCGAAAGAAAGAGAAAATGAATAACCATTGGAGCCTTGCCTTAGGAAGAGGCACTAATAGTTCAATGTCAGATATATCTGGTCACATGTCTTAGCACAAAGTCAACTGTCATACACCTATGCTATTCTAAGTAGTGCACACTCTTGTTACAGATATGCTACTCCATGTTTACAGATATGCTACTCCAAGTGCACACNNTTGTTACAGATATGCTACTCCAAGTGCACACTTTTGTTACAGATATGCTACTACAAGTGCACACTTTTGTTACAGATATGCTACTCCAAGTGCACACTCTTGTTCCTCCCTTCTTCATTTCCTCCTCCCTATCTGTGATCACACCACCCCTTTAACTCTCCCGGTACACTCACAAGATATCGCATCCTTGTCTTGTGCATCCGACACAAAGTAGATGCATCTATTTGTAATGTCCAAGGTTCCAGAGATGACCTCTACCACCGTCACAAGCTGACACATCTCTCTTATNATCAGTTTCTCTTTCTTGCCCTTCTCCAGAGCCACAGCTGGCTCATCTCGAGCTAGCTTTTGGATCTGATCAAACAGATGTTCCTCCTCCTCCTCACTGGNGGACTTTGTAGCTGTGGCCATGGCTAGCAGAGAGGTGTCTGCAGTGCTGGACACCAGGNGAGTCTCCCCAGAGCTGTGTTGACTAGCATCGGTGTGTTTGTTATAGTTGTAGTTACGGGCCAGTTTTAGCCGCATGCGTGAAAAGTTCTCTGTTCTGTCCATCTTCCACAGGTAGAGATCAGGATCTCTGGAACAGAGGATCACGGAATCACAGGGTTATGTACATATACATTCATGTGTGCCACACAGCACCACACAGAGGTACATACATGTATGTACTGTTCCATCATGTATAAAACATGAGTACAACTTCATGTACAACATCATGGGACATGTGTCCCCATGGAACATGTGTACCCATGTACAACATCATGGAACATGTGTACCATCATGTACAACATATGTACCATCAATGTACTANATACTGGCACATAGTGGGGACATGTGTACCATCATGTACNNNNNNNNNNNNNNNNNNNNNNNNNNNNNNNNNNNNNNNNNNNNNNNNNNNNNNNNNNNNNNNNNNNNNNNNNNNNNNNNNNNNNNNNNNNNNNNNNNNNNNNNNNNNNNNNNNNNNNNNNNNNNNNNNNNNNNNNNNNNNNNNNNNNNNNNNNNNNNNNNNNNNNNNNNNNNNNNNNNNNNNNNNNNNNNNNNNNNNNNNNNNNNNNNNNNNNNNNNNNNNNNNNNNNNNNNNNNNNNNNNNNNNNNNNNNNNNNNNNNNNNNNNNNNNNNNNNNNNNNNNNNNNNNNNNNNNNNNNNNNNNNNNNNNNNNNNNNNNNNNNNNNNNNNNNNNNNNNNNNNNNNNNNNNNNNNNNNNNNNNNNNNNNNNNNNNNNNNNNNNNNNNNNNNNNNNNNNNNNNNNNNNNNNNNNNNNNNNNNNNNNNNNNNNNNNNNNNNNNNNNNNNNNNNNNNNNNNNNNNNNNNNNNNNNNNNNNNNNNNNNNNNNNNNNNNNNNNNNNNNNNNNNNNNNNNNNNNNNNNNNNNNNNNNNNNNNNNNNNNNNNNNNNNNNNNNNNNNNNNNNNNNNNNNNNNNNNNNNNNNNNNNNNNNNNNNNNNNNNNNNNNNNNNNNNNNNNNNNNNNNNNNNNNNNNNNNNNNNNNNNNNNNNNNNNNNNNNNNNNNNNNNNNNNNNNNNNNNNNNNNNNNNNNNNNNNNNNNNNNNNNNNNNNNNNNNNNNNNNNNNNNNNNNNNNNNNNNNNNNNNNNNNNNNNNNNNNNNNNNNNNNNNNNNNNNNNNNNNNNNNNNNNNNNNNNNNNNNNNNNNNNNNNNNNNNNNNNNNNNNNNNNNNNNNNNNNNNNNNNNNNNNNNNNNNNNNNNNNNNNNNNNNNNNNNNNNNNNNNNNNNNNNNNNNNNNNNNNNNNNNNNNNNNNNNNNNNNNNNNNNNNNNNNNNNNNNNNNNNNNNNNNNNNNNNNNNNNNNNNNNNNNNNNNNNNNNNNNNNNNNNNNNNNNNNNNNNNNNNNNNNNNNNNNNNNNNNNNNNNNNNNNNNNNNNNNNNNNNNNNNNNNNNNNNNNNNNNNNNNNNNNNNNNNNNNNNNNNNNNNNNNNNNNNNNNNNNNNNNNNNNNNNNNNNNNNNNNNNNNNNNNNNNNNNNNNNNNNNNNNNNNNNNNNNNNNNNNNNNNNNNNNNNNNNNNNNNNNNNNNNNNNNNNNNNNNNNNNNNNNNNNNNNNNNNNNNNNNNNNNNNNNNNNNNNNNNNNNNNNNNNNNNNNNNNNNNNNNNNNNNNNNNNNNNNNNNNNNNNNNNNNNNNNNNNNNNNNNNNNNNNNNNNNNNNNNNNNNNNNNNNNNNNNNNNNNNNNNNNNNNNNNNNNNNNNNNNNNNNNNNNNNNNNNNNNNNNNNNNNNNNNNNNNNNNNNNNNNNNNNNNNNNNNNNNNNNNNNNNNNNNNNNNNNNNNNNNNNNNNNNNNNNNNNNNNNNNNNNNNNNNNNNNNNNNNNNNNNNNNNNNNNNNNNNNNNNNNNNNNNNNNNNNNNNNNNNNNNNNNNNNNNNNNNNNNNNNNNNNNNNNNNNNNNNNNNNNNNNNNNNNNNNNNNNNNNNNNNNNNNNNNNNNNNNNNNNNNNNNNNNNNNNNNNNNNNNNNNNNNNNNNNNNNNNNNNNNNNNNNNNNNNNNNNNNNNNNNNNNNNNNNNNNNNNNNNNNNNNNNNNNNNNNNNNNNNNNNNNNNNNNNNNNNNNNNNNNNNNNNNNNNNNNNNNNNNNNNNNNNNNNNNNNNNNNNNNNNNNNNNNNNNNNNNNNNNNNNNNNNNNNNNNNNNNNNNNNNNNNNNNNNNNNNNNNNNNNNNNNNNNNNNNNNNNNNNNNNNNNNNNNNNNNNNNNNNNNNNNNNNNNNNNNNNNNNNNNNNNNNNNNNNNNNNNNNNNNNNNNNNNNNNNNNNNNNNNNNNNNNNNNNNNNNNNNNNNNNNNNNNNNNNNNNNNNNNNNNNNNNNNNNNNNNNNNNNNNNNNNNNNNNNNNNNNNNNNNNNNNNNNNNNNNNNNNNNNNNNNNNNNNNNNNNNNNNNNNNNNNNNNNNNNNNNNNNNNNNNNNNNNNNNNNNNNNNNNNNNNNNNNNNNNNNNNNNNNNNNNNNNNNNNNNNNNNNNNNNNNNNNNNNNNNNNNNNNNNNNNNNNNNNNNNNNNNNNNNNNNNNNNNNNNNNNNNNNNNNNNNNNNNNNNNNNNNNNNNNNNNNNNNNNNNNNNNNNNNNNNNNNNNNNNNNNNNNNNNNNNNNNNNNNNNNNNNNNNNNNNNNNNNNNNNNNNNNNNNNNNNNNNNNNNNNNNNNNNNNNNNNNNNNNNNNNNNNNNNNNNNNNNNNNNNNNNNNNNNNNNNNNNNNNNNNNNNNNNNNNNNNNNNNNNNNNNNNNNNNNNNNNNNNNNNNNNNNNNNNNNNNNNNNNNNNNNNNNNNNNNNNNNNNNNNNNNNNNNNNNNNNNNNNNNNNNNNNNNNNNNNNNNNNNNNNNNNNNNNNNNNNNNNNNNNNNNNNNNNNNNNNNNNNNNNNNNNNNNNNNNNNNNNNNNNNNNNNNNNNNNNNNNNNNNNNNNNNNNNNNNNNNNNNNNNNNNNNNNNNNNNNNNNNNNNNNNNNNNNNNNNNNNNNNNNNNNNNNNNNNNNNNNNNNNNNNNNNNNNNNNNNNNNNNNNNNNNNNNNNNNNNNNNNNNNNNNNNNNNNNNNNNNNNNNNNNNNNNNNNNNNNNNNNNNNNNNNNNNNNNNNNNNNNNNNNNNNNNNNNNNNNNNNNNNNNNNNNNNNNNNNNNNNNNNNNNNNNNNNNNNNNNNNNNNNNNNNNNNNNNNNNNNNNNNNNNNNNNNNNNNNNNNNNNNNNNNNNNNNNNNNNNNNNNNNNNNNNNNNNNNNNNNNNNNNNNNNNNNNNNNNNNNNNNNNNNNNNNNNNNNNNNNNNNNNNNNNNNNNNNNNNNNNNNNNNNNNNNNNNNNNNNNNNNNNNNNNNNNNNNNNNNNNNNNNNNNNNNNNNNNNNNNNNNNNNNNNNNNNNNNNNNNNNNNNNNNNNNNNNNNNNNNNNNNNNNNNNNNNNNNNNNNNNNNNNNNNNNNNNNNNNNNNNNNNNNNNNNNNNNNNNNNNNNNNNNNNNNNNNNNNNNNNNNNNNNNNNNNNNNNNNNNNNNNNNNNNNNNNNNNNNNNNNNNNNNNNNNNNNNNNNNNNNNNNNNNNNNNNNNNNNNNNNNNNNNNNNNNNNNNNNNNNNNNNNNNNNNNNNNNNNNNNNNNNNNNNNNNNNNNNNNNNNNNNNNNNNNNNNNNNNNNNNNNNNNNNNNNNNNNNNNNNNNNNNNNNNNNNNNNNNNNNNNNNNNNNNNNNNNNNNNNNNNNNNNNNNNNNNNNNNNNNNNNNNNNNNNNNNNNNNNNNNNNNNNNNNNNNNNNNNNNNNNNNNNNNNNNNNNNNNNNNNNNNNNNNNNNNNNNNNNNNNNNNNNNNNNNNNNNNNNNNNNNNNNNNNNNNNNNNNNNNNNNNNNNNNNNNNNNNNNNNNNNNNNNNNNNNNNNNNNNNNNNNNNNNNNNNNNNNNNNNNNNNNNNNNNNNNNNNNNNNNNNNNNNNNNNNNNNNNNNNNNNNNNNNNNNNNNNNNNNNNNNNNNNNNNNNNNNNNNNNNNNNNNNNNNNNNNNNNNNNNNNNNNNNNNNNNNNNNNNNNNNNNNNNNNNNNNNNNNNNNNNNNNNNNNNNNNNNNNNNNNNNNNNNNNNNNNNNNNNNNNNNNNNNNNNNNNNNNNNNNNNNNNNNNNNNNNNNNNNNNNNNNNNNNNNNNNNNNNNNNNNNNNNNNNNNNNNNNNNNNNNNNNNNNNNNNNNNNNNNNNNNNNNNNNNNNNNNNNNNNNNNNNNNNNNNNNNNNNNNNNNNNNNNNNNNNNNNNNNNNNNNNNNNNNNNNNNNNNNNNNNNNNNNNNNNNNNNNNNNNNNNNNNNNNNNNNNNNNNNNNNNNNNNNNNNNNNNNNNNNNNNNNNNNNNNNNNNNNNNNNNNNNNNNNNNNNNNNNNNNNNNNNNNNNNNNNNNNNNNNNNNNNNNNNNNNNNNNNNNNNNNNNNNNNNNNNNNNNNNNNNNNNNNNNNNNNNNNNNNNNNNNNNNNNNNNNNNNNNNNNNNNNNNNNNNNNNNNNNNNNNNNNNNNNNNNNNNNNNNNNNNNNNNNNNNNNNNNNNNNNNNNNNNNNNNNNNNNNNNNNNNNNNNNNNNNNNNNNNNNNNNNNNNNNNNNNNNNNNNNNNNNNNNNNNNNNNNNNNNNNNNNNNNNNNNNNNNNNNNNNNNNNNNNNNNNNNNNNNNNNNNNNNNNNNNNNNNNNNNNNNNNNNNNNNNNNNCCACACACACATACCCCACNCACACACACACACACACACACACACACACCACACACAAACACACACCAATGAAGCAGCTGTGGGGTTGCTCTACAAATGGAGTACCTCTACAACATTAGCCATGAGGTCATCTCTCGTTTGACCTCTTTGATCGTCCCCCTTACGCACCACCTGCATTTGCTCAAAGAGGTTCACTATGACTCTCTTGCCAGTGAAGGGAGAGGAGTAGCCTCTGTAGCTCTTCACAGGCGGGTCTCTTGTCGGATGCCTCTCGTTAAACAGACGAGTTGGTGTCTGACCAAAATTGGTTATAATACTTTCAATCTCTCGGCGGCTTTTTGGGTCTTGGATGTTGTCTAAGTTGACATTTTCTTTATAAGGGAAGTGAAAATATTAACAACATTGAAGATTAGACAATAAGGTCCCAATTATCCATGTACCTTGGTATGAGTACTTGTTAAACACATTCATAGCCTTTTCTGCCTCAGGCCCAGTCTGTTTGTACCCAAAAATGAGGTCAATCCACTCATTGAGGTGTGCTGAAACGTATTCAGANTCCTGAGGGAGGAGGGTAGAGCACAATGNACACACATGCTCCTACNNGNNATTACACTGTACACACCATCACACTGTACACACCATCACAC>scaffold4236|size40413CCACCACCACCACCACCACCACCACCACCACCACCTCCTCCAGCATGGCCGGTATTAACTGCAGAATGGCGGGCACCCTCACTTCCATCCAGCATGAGGCCGAGGTGGAGACCGGGAAGCCGGGGATGTGGGCCAAAGAGGAGACTCAATAGCTGTATGCACAATACTTGATCATTATTGTACTAAATTATCCAAGAGATGGTTATGGTGCATACGAAATGCGCAATATTATCCGTCAGTTTATGCTTGACATAGATGCATGCATGGCATTAAAGTGCCAGTGTTGGCTTTAAGAGACTGTTGGATTGATGTCTGATCGATCTGGAGAACAGGACAAAGAGACAGAATCCAAACGGACTCACAATAGTATATAAAGTGGGTAGTTCCCAATGCAGTGCTACAAGATGACGAGCAAGAGAAGAAGCGTGCGTATGAAGAGAGGGTGAGAGAAATCGAGCATGGCTCCTTTGCACCCCTCGTAGTCTCAATCTCAGGAGGTATGGGTCCGATAGCCACAACCGTGTACAAAAGAATGGCCTCGCTCATAGCTCAGTGAAAAATACAATAATCCATATATCTTTTTTGGCTAAGATGCAAGCTGAGTTTCTCCTTATTGCGATCTGCATAACACATGTGCATCAGAGGCTCGAGGTCCTCCTATCACAGACCAACAAACATGTTTAGTGAGACCATTGTCCTATCCTGCTTGGTAAACAGGATCTCAATTTGAACATTGAAATCCTATCCGTCTTTTTTCTTTTATTNNNTNCTTANNTCATTACCACGTTTCCACGCATTATGTGTATATTTTTATTTAAAAAGCGTATACTTTACGGACTTATCTGCTGTACTGATTTGATTTATTGTCAGTTCAAAGCGATAGTCTCGTACCCAGACCTGACGGACGTAAAAGCATGTTTGCACGTCAGCCGAGTCTGGGTANNNNNNNNNNGGGGGCGGGGCCTAAATATACTACGGCCTGTCCGGGCCATGTAAACTTAAAAGACGTTACGAAAGTGGAAAAAAAAACTTACGAAATAAGTAAATTAAAACATTCTCTATACGCGAAAGTTGGCTCTCCTCCTCTCTTCCTGCCACTCCTCNTACCCCCATTGTCATCTTGTAACCATTACTGCCGATACATACATGATCACTTGCACAAGATTTAGTCACTATAAACTTATTGTGTAATAACTATTGCATAAAGTAGTTAAACGTGGTTAATTTCGGGTTTCCTCCCTCATCTCAAGTCACATGTTCTCATGTTCACGCCACGAGCCAGGCAGAAAACAGCATTTTTATTCATTGTCATTATTTTTATTTTATTAATTTTAGATTANCCATTACAATAAATAAATACATACATAATCAATAAACATTTGAGCACATAATACTACAAACCTACCTACCTAGGACNCNAAGAGTCCTTATCAGTTATTGAAATGACGACGACNNAGCANNNNCNCNNACCAGGGCAATGCTTAGCCTTCCATATAGCCCAGACACTGCTGAAGACATGGGACAAGCAGTTCGAGTGGCGATCCATGTAGCGAGTCTGTCGAGGCACCTGCTGNTANGTGTAAGCTCAGCTGGGGAGTTGGCGTGACCAAAAGCCAAGCTGTGGCGTGGGGAGAAGACACAGAGGGAAACCGNNNNNNNNNNNNNNNNNNNNNNNNNNNNNNNNNNNNNNNNNNNNNNNNNNNNNNNNNNNNNNNNNNNNNNNNNNNNNNNNNNNNNNNNNNNNNNNNNNNNNNNNNNNNNNNNNNNNNNNNNNNNNNNNNNNNNNNNNNNNNNNNNNNNNNNNNNNNNNNNNNNNNNNNNNNNNNNNNNNNNNNNNNNNNNNNNNNNNNNNNNNNNNNNNNNNNNNNNNNNNNNNNNNNNNNNNNNNNNNNNNNNNNNNNNNNNNNNNNNNNNNNNNNNNNNNNNNNNNNNNNNNNNNNNNNNNNNNNNNNNNNNNNNNNNNNNNNNNNNNNNNNNNNNNNNNNNNNNNNNNNNNNNNNNNNNNNNNNNNNNNNNNNNNNNNNNNNNNNNNNNNNNNNNNNNNNNNNNNNNNNNNNNNNNNNNNNNNNNNNNNNNNNNNNNNNNNNNNNNNNNNNNNNNNNNNNNNNNNNNNNNNNNNNNNNNNNNNNNNNNNNNNNNNNNNNNNNNNNNNNNNNNNNNNNNNNNNNNNNNNNNNNNNNNNNNNNNNNNNNNNNNNNNNNNNNNNNNNNNNNNNNNNNNNNNNNNNNNNNNNNNNNNNNNNNNNNNNNNNNNNNNNNNNNNNNNNNNNNNNNNNNNNNNNNNNNNNNNNNNNNNNNNNNNNNNNNNNNNNNNNNNNNNNNNNNNNNNNNNNNNNNNNNNNNNNNNNNNNNNNNNNNNNNNNNNNNNNNNNNNNNNNNNNNNNNNNNNNNNNNNNNNNNNNNNNNNNNNNNNNNNNNNNNNNNNNNNNNNNNNNNNNNNNNNNNNNNNNNNNNNNNNNNNNNNNNNNNNNNNNNNNNNNNNNNNNNNNNNNNNNNNNNNNNNNNNNNNNNNNNNNNNNNNNNNNNNNNNNNNNNNNNNNNNNNNNNNNNNNNNNNNNNNNNNNNNNNNNNNNNNNNNNNNNNNNNNNNNNNNNNNNNNNNNNNNNNNNNNNNNNNNNNNNNNNNNNNNNNNNNNNNNNNNNNNNNNNNNNNNNNNNNNNNNNNNNNNNNNNNNNNNNNNNNNNNNNNNNNNNNNNNNNNNNNNNNNNNNNNNNNNNNNNNNNNNNNNNNNNNNNNNNNNNNNNNNNNNNNNNNNNNNNNNNNNNNNNNNNNNNNNNNNNNNNNNNNNNNNNNNNNNNNNNNNNNNNNNNNNNNNNNNNNNNNNNNNNNNNNNNNNNNNNNNNNNNNNNNNNNNNNNNNNNNNNNNNNNNNNNNNNNNNNNNNNNNNNNNNNNNNNNNNNNNNNNNNNNNNNNNNNNNNNNNNNNNNNNNNNNNNNNNNNNNNNNNNNNNNNNNNNNNNNNNNNNNNNNNNNNNNNNNNNNNNNNNNNNNNNNNNNNNNNNNNNNNNNNNNNNNNNNNNNNNNNNNNNNNNNNNNNNNNNNNNNNNNNNNNNNNNNNNNNNNNNNNNNNNNNNNNNNNNNNNNNNNNNNNNNNNNNNNNNNNNNNNNNNNNNNNNNNNNNNNNNNNNNNNNNNNNNNNNNNNNNNNNNNNNNNNNNNNNNNNNNNNNNNNNNNNNNNNNNNNNNNNNNNNNNNNNNNNNNNNNNNNNNNNNNNNNNNNNNNNNNNNNNNNNNNNNNNNNNNNNNNNNNNNNNNNNNNNNNNNNNNNNNNNNNNNNNNNNNNNNNNNNNNNNNNNNNNNNNNNNNNNNNNNNNNNNNNNNNNNNNNNNNNNNNNNNNNNNNNNNNNNNNNNNNNNNNNNNNNNNNNNNNNNNNNNNNNNNNNNNNNNNNNNNNNNNNNNNNNNNNNNNNNNNNNNNNNNNNNNNNNNNNNNNNNNNNNNNNNNNNNNNNNNNNNNNNNNNNNNNNNNNNNNNNNNNNNNNNNNNNNNNNNNNNNNNNNNNNNNNNNNNNNNNNNNNNNNNNNNNNNNNNNNNNNNNNNNNNNNNNNNNNNNNNNNNNNNNNNNNNNNNNNNNNNNNNNNNNNNNNNNNNNNNNNNNNNNNNNNNNNNNNNNNNNNNNNNNNNNNNNNNNNNNNNNNNNNNNNNNNNNNNNNNNNNNNNNNNNNNNNNNNNNNNNNNNNNNNNNNNNNNNNNNNNNNNNNNNNNNNNNNNNNNNNNNNNNNNNNNNNNNNNNNNNNNNNNNNNNNNNNNNNNNNNNNNNNNNNNNNNNNNNNNNNNNNNNNNNNNNNNNNNNNNNNNNNNNNNNNNNNNNNNNNNNNNNNNNNNNNNNNNNNNNNNNNNNNNNNNNNNNNNNNNNNNNNNNNNNNNNNNNNNNNNNNNNNNNNNNNNNNNNNNNNNNNNNNNNNNNNNNNNNNNNNNNNNNNNNNNNNNNNNNNNNNNNNNNNNNNNNNNNNNNNNNNNNNNNNNNNNNNNNNNNNNNNNNNNNNNNNNNNNNNNNNNNNNNNNNNNNNNNNNNNNNNNNNNNNNNNNNNNNNNNNNNNNNNNNNNNNNNNNNNNNNNNNNNNNNNNNNNNNNNNNNNNNNNNNNNNNNNNNNNNNNNNNNNNNNNNNNNNNNNNNNNNNNNNNNNNNNNNNNNNNNNNNNNNNNNNNNNNNNNNNNNNNNNNNNNNNNNNNNNNNNNNNNNNNNNNNNNNNNNNNNNNNNNNNNNNNNNNNNNNNNNNNNNNNNNNNNNNNNNNNNNNNNNNNNNNNNNNNNNNNNNNNNNNNNNNNNNNNNNNNNNNNNNNNNNNNNNNNNNNNNNNNNNNNNNNNNNNNNNNNNNNNNNNNNNNNNNNNNNNNNNNNNNNNNNNNNNNNNNNNNNNNNNNNNNNNNNNNNNNNNNNNNNNNNNNNNNNNNNNNNNNNNNNNNNNNNNNNNNNNNNNNNNNNNNNNNNNNNNNNNNNNNNNNNNNNNNNNNNNNNNNNNNNNNNNNNNNNNNNNNNNNNNNNNNNNNNNNNNNNNNNNNNNNNNNNNNNNNNNNNNNNNNNNNNNNNNNNNNNNNNNNNNNNNNNNNNNNNNNNNNNNNNNNNNNNNNNNNNNNNNNNNNNNNNNNNNNNNNNNNNNNNNNNNNNNNNNNNNNNNNNNNNNNNNNNNNNNNNNNNNNNNNNNNNNNNNNNNNNNNNNNNNNNNNNNNNNNNNNNNNNNNNNNNNNNNNNNNNNNNNNNNNNNNNNNNNNNNNNNNNNNNNNNNNNNNNNNNNNNNNNNNNNNNNNNNNNNNNNNNNNNNNNNNNNNNNNNNNNNNNNNNNNNNNNNNNNNNNNNNNNNNNNNNNNNNNNNNNNNNNNNNNNNNNNNNNNNNNNNNNNNNNNNNNNNNNNNNNNNNNNNNNNNNNNNNNNNNNNNNNNNNNNNNNNNNNNNNNNNNNNNNNNNNNNNNNNNNNNNNNNNNNNNNNNNNNNNNNNNNNNNNNNNNNNNNNNNNNNNNNNNNNNNNNNNNNNNNNNNNNNNNNNNNNNNNNNNNNNNNNNNNNNNNNNNNNNNNNNNNNNNNNNNNNNNNNNNNNNNNNNNNNNNNNNNNNNNNNNNNNNNNNNNNNNNNNNNNNNNNNNNNNNNNNNNNNNNNNNNNNNNNNNNNNNNNNNNNNNNNNNNNNNNNNNNNNNNNNNNNNNNNNNNNNNNNNNNNNNNNNNNNNNNNNNNNNNNNNNNNNNNNNNNNNCCAGCCAATAACAGCAAGCACACGGGNCTNGNNTNNACNNGTNTATANATNNATGGCCACAAGCTGGCCAACCCAAGGCTGCATTGGAAGCAATAGGGGCAAACACAAGAGGGTGGGGCGTATATCAAAGGGGAGGCCAGTTGCAGAATGTGAACGGGGGAATTTCGAGAGCCTAAACAGTACCATAAGGGTTAGAAGCACTGAGTATGTACAGTGTATGTGTCAACGTTGCTCCATAGATAGTGTGGCCCAAAGGTGGAAGCGGCCAGTCCATACGCAGCACACACACACNCNNNCNNNNNNACNNANAGCAGCATCAGCTGCTCTACGCCTAAACACACTCACGCGACGCTCGGTTGCAATAAGCCGAAAGGATTTCTGTCCAGCGGCAGTGATGGAGGAGGAGTGACACATTTCTTCGTCGCTTTCAACAGACGCTTTAGATAAAAACAGAAAGCCTTGCTTTTTAGTTTAGGGTTCGACTGTTTTGTATACATCGACCACGGCTCCAAGAGCTCTTTAGTTCTGCAAAGATGGGCACACTTATGACTCCATTCACTCGGTCTAACCGACCGTCAAAATATATACAACGTACACAAAACTTACTTGAAAACCTCTTCCGACATGCTGAGACCTGCCCCCTCCTCCTCTGAATGATGTCAGCTTAGGTGCCTCGTTTGCAGGCTCAAATAAATTCATTGCTAAAGTCACGTGCGCAAACTATTTTTCNTTATATAGCTCACAATTAGATCATTTGTAACGCTTCTGTTCTCAAGTAAGCAAAAAATAAAAAAAGAAAGTTCTGTACCCAGACCCAAAAGTAGTAAGGAATGCAATCAGGACCAGTGGGTGTGGCATAAATTCTTTGCCCCCTCCCTATGGAAACGATCTATAGCTGCATGGGATCATGAATCATAAGGTATTCCAAGTATGTAGCNGCGTGAATGACTCTCCTGATATCAGATCCTGCAGACAGACTGAGTTGTACTGTGATCAAGTNGCATGCTTTCATATAATGCATGACCTAATGTGGTGCTCAGCAGGTTCATAACAGAGGGGCTGTGAAGTCTGACACTTCATATCCTTAACAATGGCAAATGAAATGAAACATAGATGGTTATGATAAATTTCTGCACATACATGAATATAAAAATACCCACTAACCACTCCATGCAATAAAGAACAGAAGAGAGGGTACAAAACGAAGGAAGAAACACCTGACACTCACAACATGTCACATGACAACAGCTACGCCGGCAGCTGCCCTGCAAGTCTCCATAGAACTGACCAGATCCCAGCGTGTTGTTTACAGGGTCATACAGTTCTGCTGTTTTCAAGTACGAAGGACCATTGATCCCTCCAACAGCCACCATATGTGCACCAATCGCACATAGNCAGACTCCATCGCGAGCTGAGGTCATTTTGGCTGCTGGATACCATTTGCCACTGCGAACATCGAGAAACTCCACAGAGTCCAGCCGTGAGGTTCCATCGTTACCACCNGCAACAAAAATCTGCTGACCCAAGACTGCCACCCCGATCCCCGCACGAGGTTTCCCCAGCGGGGGCAATGTTCTCCATCGGTTGACATGAGGATCGTAGCACTCCACAACGTTGAGAAACGAGGTCCCATCGTTCCCGCCAAATGTGTACAACTTGTCACCAAAGACAGCTACTCCCGGGAAACTTCTCCGTGAGCTCATCTGAGCGACAGTGACCCAAACGTTCTTTTCAAGGTCGAACCTTTCAACCGTGTTGAGACAGGAGCGACCGTCGTAGCCCCCCGCGGCGTACATGGCGCCTCCCATGACCCCCACGGCCACGCCCGAGCGACACGTGCTCAGGGGGGCCACGTACGTCCAGAACTGAGTGGCCGGATCGAAGCACTCCACGGTGCTGAGGCGCAGAGAGCCGTCCGACCCGCCCACCGCGTACACTTTGCCGTCGAGGACCCCGACGCCCAACTGTTGCCTACGAACCATGAGGGGCGTGCCCTCGCTCCACTTGTTTTCCAGCAGCGAGAACGTCTCGACGGAGTGAGTGATGCTTTCGGACGACTCCTTCCCGCCAACCGCGTACACGACGCCCACCGTGGACTTGCGCGGAAAGAAGCACGGATTGGCCGAACGGATGTCTCTGTCGGCAGGCATTAGGTGGTAGCGTTTCGCGTCGTCAAGAAGGTCCCGACACTCGATGCTCTGCTTCACGATGGGATCACGGTCAACGTCGTTCATCAAACAGTCGACGGCGAGGAGCNNNNNNNNNACGTGCTTCAGCAGCTTTGGCAGCTGTTGCCCGCGCTCGCCCGGGTCCTGCCTGACCCAGCTCATGACAGCTGCGTACACGACCTCTTCCCTGGGTACGTTCAGGTCGTTGCTTGAGATCAGCGAGGCGATACCTTCGGCCGAACTGTGGAAGAACTCGTTGGTGTTGGTCACCTCGAGGAAGTGCCGGCGAGCAAAAGAATCGACCACCTCCGACAGCTGGGTGCAGCCATTGACTTCGGCAAAATTCTTAACACCCAGGCAGTTGCTCACGTCCAAGTGGTTTTTTAGAAATTCGCAGCACACTTCCTGGGCCTGTTTTATCTGCAGCAATGAGGCAGCCGCTAACGTGTCTTGTACATTTTCCACCGTGACTGTTAAGATCCCTGTATAGGCAAAGTCAATCAAACTGCTCAGAACATCTCCACTCATGCCCTTGATTTGAACTACGTCCTGACCGCTCTCCAAAAGTTCATGAGTAAACATGGCCAGAAAGTAGGCACTGCAGGCAGACAGGATGGCTCTGTGGGCATTTATCTCTCTTCCTTCGGCACTCAACGTCACGTCACATAGCTTTTTGTGACTTCGAAGTAAGTGTACCTTTTGAAGAACGCTCTTGCTGTAGTGAAGTGTTGCTTCGTCGGCATCGAAATGTGATGATGGAGCCGTAGAACGATGTGAGTTTTCCTGACGAGATAGAAGCATTGATGTCTCTTTTTCTGCCATTCTCTTTCATTTCATCGTGTCAAATTGACGAATTGAGTCCAAAGTATGAAAATAGATACATTACGTTTCAAATTTTAAAATTACTTGCGATGTATTTTTAGAACAGTGCCGGATGGAATTGATGCAGAAGTCACGTCTAGAGGGACAACCGAGAATCAAATCGCTTATCTCCTTGTCGCTATCGAATGGCAACACAAAAATAAACAGTTTCACGTTGGTTTCATATCACAAAAGATGTGCTTTTAGAGCAAAAAAAAAATGTTTGTGGCAGTTTCTCGTGCATGATCAGTCCCTTGGTCGTCGTCTTCTTCTTCTTCTTCCTTCGTCACGTATAACTAATTAAATTTCTTTCAGCCCTGGTGACCCATTTGAGATCAACAGATCTGATGTACGACTGTACTTAAAAAACAAACAAAAAAACAAAAAAAAACACGCATACATACGTCGCTATTGAATTTATTTGGGAACTGTATCTTACCAGTAGGCCCTAAATCTGACAACTGTAGGTGAAAGAATGCAGCCATTCCCTTTCCTTCCCTTAGTCTCGTCCCCATATTTTATTCCTTTCGCCGTAGCGGTAATGTGCTGCGGTATATTGGGTCTGGGGACGAGACACTACTACCACCACCACCACCACCACCACCACCACCACCACCNNNNNNNNNNNNNNNNNNNNNNNNNNNNNNNNNNNNNNNNNNNNNNNNNNNNNNNNNNNNNNNNNNNNNNNNNNNNNNNNNNNNNNNNNNNNNNNNNNNNNNNNNNNNNNNNNNNNNNNNNNNNNNNNNNNNNNNNNNNNNNNNNNNNNNNNNNNNNNNNNNNNNNNNNNNNNNNNNNNNNNNNNNNNNNNNNNNNNNNNNNNNNNNNNNNNNNNNNNNNNNNNNNNNNNNNNNNNNNNNNNNNNNNNNNNNNNNNNNNNNNNNNNNNNNNNNNNNNNNNNNNNNNNNNNNNNNNNNNNNNNNNNNNNNNNNNNNNNNNNNNNNNNNNNNNNNNNNNNNNNNNNNNNNNNNNNNNNNNNNNNNNNNNNNNNNNNNNNNNNNNNNNNNNNNNNNNNNNNNNNNNNNNNNNNNNNNNNNNNNNNNNNNNNNNNNNNNNNNNNNNNNNNNNNNNNNNNNNNNNNNNNNNNNNNNNNNNNNNNNNNNNNNNNNNNNNNNNNNNNNNNNNNNNNNNNNNNNNNNNNNNNNNNNNNNNNNNNNNNNNNNNNNNNNNNNNNNNNNNNNNNNNNNNNNNNNNNNNNNNNNNNNNNNNNNNNNNNNNNNNNNNNNNNNNNNNNNNNNNNNNNNNNNNNNNNNNNNNNNNNNNNNNNNNNNNNNNNNNNNNNNNNNNNNNNNNNNNNNNNNNNNNNNNNNNNNNNNNNNNNNNNNNNNNNNNNNNNNNNNNNNNNNNNNNNNNNNNNNNNNNNNNNNNNNNNNNNNNNNNNNNNNNNNNNNNNNNNNNNNNNNNNNNNNNNNNNNNNNNNNNNNNNNNNNNNNNNNNNNNNNNNNNNNNNNNNNNNNNNNNNNNNNNNNNNNNNNNNNNNNNNNNNNNNNNNNNNNNNNNNNNNNNNNNNNNNNNNNNNNNNNNNNNNNNNNNNNNNNNNNNNNNNNNNNNNNNNNNNNNNNNNNNNNNNNNNNNNNNNNNNNNNNNNNNNNNNNNNNNNNNNNNNNNNNNNNNNNNNNNNNNNNNNNNNNNNNNNNNNNNNNNNNNNNNNNNNNNNNNNNNNNNNNNNNNNNNNNNNNNNNNNNNNNNNNNNNNNNNNNNNNNNNNNNNNNNNNNNNNNNNNNNNNNNNNNNNNNNNNNNNNNNNNNNNNNNNNNNNNNNNNNNNNNNNNNNNNNNNNNNNNNNNNNNNNNNNNNNNNNNNNNNNNNNNNNNNNNNNNNNNNNNNNNNNNNNNNNNNNNNNNNNNNNNNNNNNNNNNNNNNNNNNNNNNNNNNNNNNNNNNNNNNNNNNNNNNNNNNNNNNNNNNNNNNNNNNNNNNNNNNNNNNNNNNNNNNNNNNNNNNNNNNNNNNNNNNNNNNNNNNNNNNNNNNNNNNNNNNNNNNNNNNNNNNNNNNNNNNNNNNNNNNNNNNNNNNNNNNNNNNNNNNNNNNNNNNNNNNNNNNNNNNNNNNNNNNNNNNNNNNNNNNNNNNNNNNNNNNNNNNNNNNNNNNNNNNNNNNNNNNNNNNNNNNNNNNNNNNNNNNNNNNNNNNNNNNNNNNNNNNNNNNNNNNNNNNNNNNNNNNNNNNNNNNNNNNNNNNNNNNNNNNNNNNNNNNNNNNNNNNNNNNNNNNNNNNNNNNNNNNNNNNNNNNNNNNNNNNNNNNNNNNNNNNNNNNNNNNNNNNNNNNNNNNNNNNNNNNNNNNNNNNNNNNNNNNNNNNNNNNNNNNNNNNNNNNNNNNNNNNNNNNNNNNNNNNNNNNNNNNNNNNNNNNNNNNNNNNNNNNNNNNNNNNNNNNNNNNNNNNNNNNNNNNNNNNNNNNNNNNNNNNNNNNNNNNNNNNNNNNNNNNNNNNNNNNNNNNNNNNNNNNNNNNNNNNNNNNNNNNNNNNNNNNNNNNNNNNNNNNNNNNNNNNNNNNNNNNNNNNNNNNNNNNNNNNNNNNNNNNNNNNNNNNNNNNNNNNNNNNNNNNNNNNNNNNNNNNNNNNNNNNNNNNNNNNNNNNNNNNNNNNNNNNNNNNNNNNNNNNNNNNNNNNNNNNNNNNNNNNNNNNNNNNNNNNNNNNNNNNNNNNNNNNNNNNNNNNNNNNNNNNNNNNNNNNNNNNNNNNNNNNNNNNNNNNNNNNNNNNNNNNNNNNNNNNNNNNNNNNNNNNNNNNNNNNNNNNNNNNNNNNNNNNNNNNNNNNNNNNNNNNNNNNNNNNNNNNNNNNNNNNNNNNNNNNNNNNNNNNNNNNNNNNNNNNNNNNNNNNNNNNNNNNNNNNNNNNNNNNNNNNNNNNNNNNNNNNNNNNNNNNNNNNNNNNNNNNNNNNNNNNNNNNNNNNNNNNNNNNNNNNNNNNNNNNNNNNNNNNNNNNNNNNNNNNNNNNNNNNNNNNNNNNNNNNNNNNNNNNNNNNNNNNNNNNNNNNNNNNNNNNNNNNNNNNNNNNNNNNNNNNNNNNNNNNNNNNNNNNNNNNNNNNNNNNNNNNNNNNNNNNNNNNNNNNNNNNNNNNNNNNNNNNNNNNNNNNNNNNNNNNNNNNNNNNNNNNNNNNNNNNNNNNNNNNNNNNNNNNNNNNNNNNNNNNNNNNNNNNNNNNNNNNNNNNNNNNNNNNATGTGTGTGTGTNTGTGTNTGTGTGTGTGTNTNTGTGTGTGTGAGGATATGTGTAAGAGGAGAGTATTAAATCATGAGTGCATAAAAGCATACAATGGGTTAAAACAATGAGCTGTTAACAAATCATCAAATATACCAGATCTATTTATACAGGAGTACCACAAATATAGAAATGTAAGCAAATTAAACTAGTTACCATGTTCAATAGTTCCTGGAGGACCTCCATAGTAGGCGGGGTAAGAGAAGCCATTAATTTTTTCAAAACACTGTATTTTATGTGCTGTCTGAATGATGCTTTTATAGTTGACGATCCTTTCAGTGCCACAGTGAGAGTCCTTATCAGCAGACATACAAGGGTGGTTCCCAGACCACCAAGGGGAACATTNTTTAGCTCCTTGAAGGACTCCACCAGCTTCTCAAAACAGCCCACTTCTGCAAAGAGCTCCTGTGTGGTTTGGATAACAATGGGTGCTCATGGCTTGGTCGTGTGGCTTTACCTTCAACTGGGTGGAGCCCTTGGGAGCATCCATCAATAGTGAAATAGAGTCTGGAGGAGAGAACCAAGCAATAAGGTGCAAGAAAGGAAGTGAATGATGAGGGAGGTGAACAAGGGGGAGAGGGAGGGAACATAGGGGGAGAGGGAGGGAACAAGGGGGAGAGGGAGGGAACAAGGGGGAGAGAGGGAACAAGGGGGAGAGAGGGAACAAGGGGGAGAGAGGGAACAAGGGGGAGAGGGAGGGAACAAGGGGAGAGGGAGGGAACAAGGGGGAGAGAGAGAACAAGGGAGAAAGGGAGGGAACAAGGNNNANANNNANNNACACANNNGAGAGAGAGAGGGAACAATGAGGGAGGGGAACAAGGGGGAGAGAGGAAGGAGAGCAAGGATTAAAATACTCACTGAGTAAAAGAACGAAGAATGTAGGAAGAGCTTGTTGCTGTTCAGGTTTTAATTCAGAAATGCTTTATGCCAATACAACAACCAAATATTAAGAACAAATCTTCACAAANGAAGGTGGTAGTCACCTGTATGGCAGGACACTGAACAGCTTAGATAAAGTGACTCCAGTGTCCACCTCAACCTGTTCCTATGGTAACACCACAACATAAATATATGAACATCCCTCTCTTCTCATGCCCCACCCACTTGCCTCTTTGGGCATGAAACAATGGAGGACGTGTAACACTTTAATGAACACCTTTATGGTCAGTGCCTGCNNNNNNNNNNNNNNNNNNNNNNNNNNNNNNNNNNNNNNNNNNNNNNNNNNNNNNNNNNNNNNNNNNNNNNNNNNNNNNNNNNNNNNNNNNNNNNNNNNNNNNNNNNNNNNNNNNNNNNNNNNNNNNNNNNNNNNNNNNNNNNNNNNNNNNNNNNNNNNNNNNNNNNNNNNNNNNNNNNNNNNNNNNNNNNNNNNNNNNNNNNNNNNNNNNNNNNNNNNNNNNNNNNNNNNNNNNNNNNNNNNNNNNNNNNNNNNNNNNNNNNNNNNNNNNNNNNNNNNNNNNNNNNNNNNNNNNNNNNNNNNNNNNNNNNNNNNNNNNNNNNNNNNNNNNNNNNNNNNNNNNNNNNNNNNNNNNNNNNNNNNNNNNNNNNNNNNNNNNNNNNNNNNNNNNNNNNNNNNNNNNNNNNNNNNNNNNNNNNNNNNNNNNNNNNNNNNNNNNNNNNNNNNNNNNNNNNNNNNNNNNNNNNNNNNNNNNNNNNNNNNNNNNNNNNNNNNNNNNNNNNNNNNNNNNNNNNNNNNNNNNNNNNNNNNNNNNNNNNNNNNNNNNNNNNNNNNNNNNNNNNNNNNNNNNNNNNNNNNNNNNNNNNNNNNNNNNNNNNNNNNNNNNNNNNNNNNNNNNNNNNNNNNNNNNNNNNNNNNNNNNNNNNNNNNNNNNNNNNNNNNNNNNNNNNNNNNNNNNNNNNNNNNNNNNNNNNNNNNNNNNNNNNNNNNNNNNNNNNNNNNNNNNNNNNNNNNNNNNNNNNNNNNNNNNNNNNNNNNNNNNNNNNNNNNNNNNNNNNNNNNNNNNNNNNNNNNNNNNNNNNNNNNNNNNNNNNNNNNNNNNNNNNNNNNNNNNNNNNNNNNNNNNNNNNNNNNNNNNNNNNNNNNNNNNNNNNNNNNNNNNNNNNNNNNNNNNNNNNNNNNNNNNNNNNNNNNNNNNNNNNNNNNNNNNNNNNNNNNNNNNNNNNNNNNNNNNNNNNNNNNNNNNNNNNNNNNNNNNNNNNNNNNNNNNNNNNNNNNNNNNNNNNNNNNNNNNNNNNNNNNNNNNNNNNNNNNNNNNNNNNNNNNNNNNNNNNNNNNNNNNNNNNNNNNNNNNNNNNNNNNNNNNNNNNNNNNNNNNNNNNNNNNNNNNNNNNNNNNNNNNNNNNNNNNNNNNNNNNNNNNNNNNNNNNNNNNNNNNNNNNNNNNNNNNNNNNNNNNNNNNNNNNNNNNNNNNNNNNNNNNNNNNNNNNNNNNNNNNNNNNNNNNNNNNNNNNNNNNNNNNNNNNNNNNNNNNNNNNNNNNNNNNNNNNNNNNNNNNNNNNNNNNNNNNNNNNNNNNNNNNNNNNNNNNNNNNNNNNNNNNNNNNNNNNNNNNNNNNNNNNNNNNNNNNNNNNNNNNNNNNNNNNNNNNNNNNNNNNNNNNNNNNNNNNNNNNNNNNNNNNNNNNNNNNNNNNNNNNNNNNNNNNNNNNNNNNNNNNNNNNNNNNNNNNNNNNNNNNNNNNNNNNNNNNNNNNNNNNNNNNNNNNNNNNNNNNNNNNNNNNNNNNNNNNNNNNNNNNNNNNNNNNNNNNNNNNNNNNNNNNNNNNNNNNNNNNNNNNNNNNNNNNNNNNNNNNNNNNNNNNNNNNNNNNNNNNNNNNNNNNNNNNNNNNNNNNNNNNNNNNNNNNNNNNNNNNNNNNNNNNNNNNNNNNNNNNNNNNNNNNNNNNNNNNNNNNNNNNNNNNNNNNNNNNNNNNNNNNNNNNNNNNNNNNNNNNNNNNNNNNNNNNNNNNNNNNNNNNNNNNNNNNNNNNNNNNNNNNNNNNNNNNNNNNNNNNNNNNNNNNNNNNNNNNNNNNNNNNNNNNNNNNNNNNNNNNNNNNNNNNNNNNNNNNNNNNNNNNNNNNNNNNNNNNNNNNNNNNNNNNNNNNNNNNNNNNNNNNNNNNNNNNNNNNNNNNNNNNNNNNNNNNNNNNNNNNNNNNNNNNNNNNNNNNNNNNNNNNNNNNNNNNNNNNNNNNNNNNNNNNNNNNNNNNNNNNNNNNNNNNNNNNNNNNNNNNNNNNNNNNNNNNNNNNNNNNNNNNNNNNNNNNNNGAGGGAGGAGGGAGGGTTAGAGAACAAGGTGAAGAAACAAGAGGAAGACAAGTGGTGTCAGTGAAGGCTCATTGGAGTTAAACACATATGGAGGCTCATTGGAGTTAAACACATATGGAGGCTCATTGGAGTTAAACACATATGGAGGCTCATTGGAGTTAAACACANATGGAAGCTCAGTGGGGTTAAACACATACAAAGGCTCATTGGGGTTAAAAATTACCAGTTCCAAATTCTCGCCACACTTCACTGGTCGAAGGGAAACCAAAATGTCCTTGAGCTCAGTACCCATGATCGAGTGGGTGCCCAGTCCTTCAATCAATCTCAACAAATTGTCTGCAACCCAAAGAGAGTTGTTACACCCCACAGGGGGGGTGGGGCACACAGGGNNGNGNGGCNNACTAGGGCCGGGTGGCTAACTANNNNNNNNNNNNNNNNNNNNNNNNNNNNNNNNNNNNNNNNNNNNNNNNNNNNNNNNNNNNNNNNNNNNNNNNNNNNNNNNNNNNNNNNNNNNNNNNNNNNNNNNNNNNNNNNNNNNNNNNNNNNNNNNNNNNNNNNNNNNNNNNNNNNNNNNNNNNNNNNNNNNNNNNNNNNNNNNNNNNNNNNNNNNNNNNNNNNNNNNNNNNNNNNNNNNNNNNNNNNNNNNNNNNNNNNNNNNNNNNNNNNNNNNNNNNNNNNNNNNNNNNNNNNNNNNNNNNNNNNNNNNNNNNNNNNNNNNNNNNNNNNNNNNNNNNNNNNNNNNNNNNNNNNNNNNNNNNNNNNNNNNNNNNNNNNNNNNNNNNNNNNNNNNNNNNNNNNNNNNNNNNNNNNNNNNNNNNNNNNNNNNNNNNNNNNNNNNNNNNNNNNNNNNNNNNNNNNNNNNNNNNNNNNNNNNNNNNNNNNNNNNNNNNNNNNNNNNNNNNNNNNNNNNNNNNNNNNNNNNNNNNNNNNNNNNNNNNNNNNNNNNNNNNNNNNNNNNNNNNNNNNNNNNNNNNNNNNNNNNNNNNNNNNNNNNNNNNNNNNNNNNNNNNNNNNNNNNNNNNNNNNNNNNNNNNNNNNNNNNNNNNNNNNNNNNNNNNNNNNNNNNNNNNNNNNNNNNNNNNNNNNNNNNNNNNNNNNNNNNNNNNNNNNNNNNNNNNNNNNNNNNNNNNNNNNNNNNNNNNNNNNNNNNNNNNNNNNNNNNNNNNNNNNNNNNNNNNNNNNNNNNNNNNNNNNNNNNNNNNNNNNNNNNNNNNNNNNNNNNNNNNNNNNNNNNNNNNNNNNNNNNNNNNNNNNNNNNNNNNNNNNNNNNNNNNNNNNNNNNNNNNNNNNNNNNNNNNNNNNNNNNNNNNNNNNNNNNNNNNNNNNNNNNNNNNNNNNNNNNNNNNNNNNNNNNNNNNNNNNNNNNNNNNNNNNNNNNNNNNNNNNNNNNNNNNNNNNNNNNNNNNNNNTCAAAAGCATTGCGTTCTTTCGTGGACACCCGGTATACAGCACAACAGAGTAGCATTATTAAACATTAACTATTGAATGCCACTGCTCCTTATTTGCCAAGAGGTAATGAACACAAGGACACACCCATAATGAGTAACAGCATTTCTATGACATCATGACGTCAGTAATATCAGACAAGCCCTTTAACTCAATCCTGATTGCACGTAACAGTTTTAAGGCAGAACAGCACTGCCCTTCTGTCCAGTCAAACAGCCCTGTTTCCGTAGGACACAGCACAGAGACACTTACCAGTATTCCAAAATTCACTAGCTTTGGGTCGAGGTGGCTCAATGATGGAATATGCCTCCCTCTGTCTCAAGGGCAGTCCGGCCTGCTCCAGTATGGGGAAGAACACCTCCACTCCACCCAGGGAACTCAAAACCAACTGAAATGGGGGAGGTGGCGTCCATTGGCAGGGCTATGAGNTNTCCCCACCTTTCCTCCTCNCCCTCCCTCCCTTATCGATAATGTACAGGCTTGCCTTGACGTCTCTGATCACATAGACGTCTCCATNCAGTACTGCATCCACAGTCCCNTGCACAGCATGTGACGGAGAGAGGTCCACACACATCTTATCTTTGCATGCCTGTGCACGATGGGACAAGCTAGCTACAAGAGNACCACACTCACTGTTGCCATGACTACCTTCGGATGATAGTAAAAGATCAGCTTAGTCTTCCACTCGTCTGCCTTCGTTTGGAAGATNGACAAATCATTAGGTCCTAAGAATAGCAATTATAATTGATGCCCACCACTTCCCTCGTACCCAGTCCTTACCACGANAGTGGAGAAGCTGGACAGTGGTGTGGTTGAGTGNGTCAGAGAAGAGACACACAGGNCCAATGCAGCCCCCAAAGTTGGTCACAGAGCCAAAGGACGTGATACACTCATCCAGCCGCAAGGGGCTGATGATGTCATGAGGGGTGGGGCTCTCANGGGGAGAGGTGGAGCCCTCTGCGGAGAGCTGATTGTCTGTGCTGTCATGGCTCCAGCCAATCCTGCACTCTGTAAATGTCTGCATGGAGGAAGACATGAGGGAGGAGGAAGGCATGGGGAGGAGGAAGGCATGGGAGGAGGAAGACATGGGGAGGAGGAAGACATGGGGAGGAGGAAGACATGGGGAGGAGGAAGACATGGGGAGGAGGAAGGCGTGGGGAAGAGGAAGGCATGGGGAGGAGGAAGACATGGGGGAGGAGGAAGACATGGGGAGGAGGTNNNNCANTGGGGAGGAGGAAGNCATGGGGGAGGAGGAAGACATGGGGGAGGAGGAAGACATGGGGGGAGAAGGAGAACATGGAGGGAGGAGGAACGGGGGAGGAATGCAAAGGAAGGGATGATAATGGAAGTGTGATAAACCCAGTGTCTACCACTCACATTGGCAAGTGGTACAGCTCTGATCCCATCTGTTGCGGAGATTAGCCCATCCACGAGCACCTGTATGGTGACCTTGTCGCTGTGCTTCTTGCTACTGCTGTACACCACACACACTGAATGCTGGGCACCAGGTAGAGGTAGAGCANAACATAAATACACTCCACCCCTCCCCAATGACNAAAAGAACGCGTACCCATTTCTGTGGAGTGAGTGGCTCTGTGAGAGAAACAGTCTTAAGCCCCCTCTTCTTGGAGTGAACTGCCACCACCAGGACCAGGTCTTTGGTAAAGAACGCTTCAAACCCTGTACTACCAGCATAGAAACTGAAAGTAAGAGACCATTGGGTTATGATATGTAGCCCCTCCTTCTGCTCCACCCACCTGTAAAGCATTCTTCTCATGTTCCTTTGGTCCCCATCTGCACAACTTCGATCCAAACTCACCCAGCAGTGGAAGGAGAAGGCATTTTCTTTGGGCCAGCGATGTAGCTCTGAGATGGATATTTGCTGTANAGAGGATAAGATGATAAGGATGACACTAACACAGAGATAAGATGACAGAGATGACACTAACACAGAGATAAGATAAGGATGACACTAACACAGAGATAAGATAAGGATGACACTAANNNNNNNNNNNNNNNNNNNNNNNNNNNNNNNNNNNNNNNNNNNNNNNNNNNNNNNNNNNNNNNNNNNNNNNNNNNNNNNNNNNNNNNNNNNNNNNNNNNNNNNNNNNNNNNNNNNNNNNNNNNNNNNNNNNNNNNNNNNNNNNNNNNNNNNNNNNNNNNNNNNNNNNNNNNNNNNNNNNNNNNNNNNNNNNNNNNNNNNNNNNNNNNNNNNNNNNNNNNNNNNNNNNNNNNNNNNNNNNNNNNNNNNNNNNNNNNNNNNNNNNNNNNNNNNNNNNNNNNNNNNNNNNNNNNNNNNNNNNNNNNNNNNNNNNNNNNNNNNNNNNNNNNNNNNNNNNNNNNNNNNNNNNNNNNNNNNNNNNNNNNNNNNNNNNNNNNNNNNNNNNNNNNNNNNNNNNNNNNNNNNNNNNNNNNNNNNNNNNNNNNNNNNNNNNNNNNNNNNNNNNNNNNNNNNNNNNNNNNNNNNNNNNNNNNNNNNNNNNNNNNNNNNNNNNNNNNNNNNNNNNNNNNNNNNNNNNNNNNNNNNNNNNNNNNNNNNNNNNNNNNNNNNNNNNNNNNNNNNNNNNNNNNNNNNNNNNNNNNNNNNNNNNNNNNNNNNNNNNNNNNNNNNNNNNNNNNNNNNNNNNNNNNNNNNNNNNNNNNNNNNNNNNNNNNNNNNNNNNNNNNNNNNNNNNNNNNNNNNNNNNNNNNNNNNNNNNNNNNNNNNNNNNNNNNNNNNNNNNNNNNNNNNNNNNNNNNNNNNNNNNNNNNNNNNNNNNNNNNNNNNNNNNNNNNNNNNNNNNNNNNNNNNNNNNNNNNNNNNNNNNNNNNNNNNNNNNNNNNNNNNNNNNNNNNNNNNNNNNNNNNNNNNNNNNNNNNNNNNNNNNNNNNNNNNNNNNNNNNNNNNNNNNNNNNNNNNNNNNNNNNNNNNNNNNNNNNNNNNNNNNNNNNNNNNNNNNNNNNNNNNNNNNNNNNNNNNNNNNNNNNNNNNNNNNNNNNNNNNNNNNNNNNNNNNNNNNNNNNNNNNNNNNNNNNNNNNNNNNNNNNNNNNNNNNNNNNNNNNNNNNNNNNNNNNNNNNNNNNNNNNNNNNNNNNNNNNNNNNNNNNNNNNNNNNNNNNNNNNNNNNNNNNNNNNNNNNNNNNNNNNNNNNNNNNNNNNNNNNNNNNNNNNNNNNNNNNNNNNNNNNNNNNNNNNNNNNNNNNNNNNNNNNNNNNNNNNNNNNNNNNNNNNNNNNNNNNNNNNNNNNNNNNNNNNNNNNNNNNNNNNNNNNNNNNNNNNNNNNNNNNNNNNNNNNNNNNNNNNNNNNNNNNNNNNNNNNNNNNNNNNNNNNNNNNNNNNNNNNNNNNNNNNNNNNNNNNNNNNNNNNNNNNNNNNNNNNNNNNNNNNNNNNNNNNNNNNNNNNNNNNNNNNNNNNNNNNNNNNNNNNNNNNNNNNNNNNNNNNNNNNNNNNNNNNNNNNNNNNNNNNNNNNNNNNNNNNNNNNNNNNNNNNNNNNNNNNNNNNNNNNNNNNNNNNNNNNNNNNNNNNNNNNNNNNNNNNNNNNNNNNNNNNNNNNNNNNNNNNNNNNNNNNNNNNNNNNNNNNNNNNNNNNNNNNNNNNNNNNNNNNNNNNNNNNNNNNNNNNNNNNNNNNNNNNNNNNNNNNNNNNNNNNNNNNNNNNNNNNNNNNNNNNNNNNNNNNNNNNNNNNNNNNNNNNNNNNNNNNNNNNNNNNNNNNNNNNNNNNNNNNNNNNNNNNNNNNNNNNNNNNNNNNNNNNNNNNNNNNNNNNNNNNNNNNNNNNNNNNNNNNNNNNNNNNNNNNNNNNNNNNNNNNNNNNNNNNNNNNNNNNNNNNNNNNNNNNNNNNNNNNNNNNNNNNNNNNNNNNNNNNNNNNNNNNNNNNNNNNNNNNNNNNNNNNNNNNNNNNNNNNNNNNNNNNNNNNNNNNNNNNNNNNNNNNNNNNNNNNNNNNNNNNNNNNNNNNNNNNNNNNNNNNNNNNNNNNNNNNNNNNNNNNNNNNNNNNNNNNNNNNNNNNNNNNNNNNNNNNNNNNNNNNNNNNNNNNNNNNNNNNNNNNNNNNNNNNNNNNNNNNNNNNNNNNNNNNNNNNNNNNNNNNNNNNNNNNNNNNNNNNNNNNNNNNNNNNNNNNNNNNNNNNNNNNNNNNNNNNNNNNNNNNNNNNNNNNNNNNNNNNNNNNNNNNNNNNNNNNNNNNNNNNNNNNNNNNNNNNNNNNNNNNNNNNNNNNNNNNNNNNNNNNNNNNNNNNNNNNNNNNNNNNNNNNNNNNNNNNNNNNNNNNNNNNNNNNNNNNNNNNNNNNNNNNNNNNNNNNNNNNNNNNNNNNNNNNNNNNNNNNNNNNNNNNNNNNNNNNNNNNNNNNNNNNNNNNNNNNNNNNNNNNNNNNNNNNNNNNNNNNNNNNNNNNNNNNNNNNNNNNNNNNNNNNNNNNNNNNNNNNNNNNNNNNNNNNNNNNNNNNNNNNNNNNNNNNNNNNNNNNNNNNNNNNNNNNNNNNNNNNNNNNNNNNNNNNNNNNNNNNNNNNNNNNNNNNNNNNNNNNNNNNNNNNNNNNNNNNNNNNNNNNNNNNNNNNNNNNNNNNNNNNNNNNNNNNNNNNNNNNNNNNNNNNNNNNNNNNNNNNNNNNNNNNNNNNNNNNNNNNNNNNNNNNNNNNNNNNNNNNNNNNNNNNNNNNNNNNNNNNNNNNNNNNNNNNNNNNNNNNNNNNNNNNNNNNNNNNNNNNNNNNNNNNNNNNNNNNNNNNNNNNNNNNNNNNNNNNNNNNNNNNNNNNNNNNNNNNNNNNNNNNNNNNNNNNNNNNNNNNNNNNNNCACACACACACACACACACACATACACACACACACACTTTGAAANAGTCANATANAGAGGGAGTGGTGTGTGGCACACCTTCTGAAGGAGGGCTCCAATTGTAGCTGGTCCCCCAACGACAAAGAAGACCTGCTCATTGATTGATGTCTTGGTCAACATGGAGGTCATCAGTCGAATGAAGTATGTGACTGCCTCATGTTCTGGAAGTGTGCAAATAACAGGTGTGAACTCATGCTTGAGATGGCACTGCACGTAATGTCTGGAGAAGTCTTGATCAACAGACACAACAGTGGATTTCACCTCTAACGCTTACCATTATTCCAAGGTTCAAAGTTCAATGTGGTTCTGGGCACATGATTTATATTTATGGAACATATTTCCCTCTGTNTCAAAGGCAGTCCNGCCTGCTCCAGTATGGGGAAGAACACCTCCACTCCACCCAGGGAACTCAAAACCAACTGAAATGGGGGAGGTGGTGTCAATGAGATACTACTGTCACTCCCTTCCCTCACTCCCCCTTGGGATCAACTGGCTTGCCTGAACATCTCTGGTCACATAGACTTCACCAACCAGTACGCCCTCCATGGCTCCTTCCACTGTGTGTGATGGAGAGAGGTCCACACAGATGTTGNNCTTACAGGCCTGAAGGGAAGAAGAGACCATCAAAGATGGTGCCTAGAACAGAGGGGGATTTCCCCACCTTTGGGTGGAAGAACAGCACCAGTTTACTAATGAGTGCCTCCGTGTCAGGATACTTTGGTTGGAAAAGAGATAGGTTATTGGGTCCTGGCATATGTGGAAAAAGAAACAAAACAGGTCAATAAGCAGCATGTCTNCNNNNANNGATNNANTGGTNNCNNCTCCTACTTCCCCACCCANNNNNNNNNNNNNNNNNNNNNNNNNNNNNNNNNNNNNNNNNNNNNNNNNCCTCCTACTTCCCCACCCACTGTCCACTGTAACCCCTCCTACTTCCCCACCCACTGTCCACAGTAACCCCTCCTACTTCCCCACCCACTGTCCACTGTAACCCCTCCTACTTCCCCACCCACTGTCCACTGTAACCCTCCTACTTCCCCATTACATACACACCATTTCGATACAAAAGCTGTACAGCTTGCAGTTGCAACACTTCTGAGAAGATGTAGACAGAGCTGATCCTGCCAAACAGGCTAATGGCAGGACCAAAGGCTACGGGACTGTCCGCCATTGCCTCAAGCGATTCTGAAAGATCTGCCTCACGTTGGCAAAACCCAATATTGCAATGCTTGTAATCCTGGGGAGGAGGCAGTGTGCCAAGTGAGGATGTGGGGAGGAGAGGGAGAAGGTTGAGAGGAGAGTGAGGAGGTTGAGAGGAGAGGGAGAAGGTTGAGAGGAGAGGGAGGAGGTTGAGAGGAGAGGGAGGAGGTTGAGAGGAGAGGGAGGAGGTTGAGAGGAGAGGGAGGAGGTTGAGAGGAGAGGGAGGAGGTTGAGAGGAGAGGGAGGAAATTAAGAGGAGAGGNAGGAGGTTGAGAGGAGAGGGAGGAGGTTGAGAGNAGAGGGAGGAGGNNGNGTGGTTGAAAGGATTGGGAGGAGGAAGTGCAGGGCACACCAAGTAAAAGAGAGAAATCTGCATGCATGTGACCTTTACTTACGGCAGAAAGGTCTGGTGCTCTGAGATCAGTTGAAAAGGCTTTCTGGATTTCATCGATGTACACAGCAGTCTCATTTTTCCCACCCCAGGGTTTTTTAGAAGCGCTATGCGTCACACATACTGCATGCTAATGGAAGCTCACTGCATTTGATGCTATTGCAGCATGCCAGAACATCACCAGCGATGTCTTACCCATTTCTGAGGACTGAGTGGCTCTTTCACTGAGATGGTTTGAAACCCTCTCTTCTTGGAGTGAACTGCCACCACCAGGACCAGGTCTTTGGTAAAGAACGCTTCAAACCCTGTACTACCAGCATAGAAACTGAAAGAAATAGACCATTGGGTTATGATACGTAGCCCCTCCTTCTGCTCCACCCACCTGTAAAGCATTCTTCTCATGTTCCTCTGGTCCCCATCTGCACAACTTTGATCCAAACTCACCCAGCAGTGGAAGGAGAAGNCATACTGGTTTGGTTGCACGACTTCTGGAACAGCTATATGTATATGCTGTGCATNAAGTATTAGAGAGGAGGGAGAGGAGGGAGAGGAGGGAGAGGAGGGAGAGGAGGGAGAGGGAGGGGAGAGGGGAGGAGGAGGGAGAAGGGAGAGGAGGGGGAAGGGAGAGGAGGGAGGAGGGAGAAGAGGGAGAAGAGGAAGAAGAGGGGGAGGAGGGAGAGGAGGGGGAGGAGGAGGGAGAAGGGAGAGGAGGGGGAGAGGGANNNNNNNNNNNNNNNNNNNNNNNNNNNNNNNNNNNNNNNNNNNNNNNNNNNNNNNNNNNNNNNNNNNNNNNNNNNNNNNNNNNNNNNNNNNNNNNNNNNNNNNNNNNNNNNNNNNNNNNNNNNNNNNNNNNNNNNNNNNNNNNNNNNNNNNNNNNNNNNNNNNNNNNNNNNNNNNNNNNNNNNNNNNNNNNNNNNNNNNNNNNNNNNNNNNNNNNNNNNNNNNNNNNNNNNNNNNNNNNNNNNNNNNNNNNNNNNNNNNNNNNNNNNNNNNNNNNNNNNNNNNNNNNNNNNNNNNNNNNNNNNNNNNNNNNNNNNNNNNNNNNNNNNNNNNNNNNNNNNNNNNNNNNNNNNNNNNNNNNNNNNNNNNNNNNNNNNNNNNNNNNNNNNNNNNNNNNNNNNNNNNNNNNNNNNNNNNNNNNNNNNNNNNNNNNNNNNNNNNNNNNNNNNNNNNNNNNNNNNNNNNNNNNNNNNNNNNNNNNNNNNNNNNNNNNNNNNNNNNNNNNNNNNNNNNNNNNNNNNNNNNNNNNNNNNNNNNNNNNNNNNNNNNNNNNNNNNNNNNNNNNNNNNNNNNNNNNNNNNNNNNNNNNNNNNNNNNNNNNNNNNNNNNNNNNNNNNNNNNNNNNNNNNNNNNNNNNNNNNNNNNNNNNNNNNNNNNNNNNNNNNNNNNNNNNNNNNNNNNNNNNNNNNNNNNNNNNNNNNNNNNNNNNNNNNNNNNNNNNNNNNNNNNNNNNNNNNNNNNNNNNNNNNNNNNNNNNNNNNNNNNNNNNNNNNNNNNNNNNNNNNNNNNNNNNNNNNNNNNNNNNNNNNNNNNNNNNNNAGCCGGACGAGGTCAATCTCCCGACAACAGAGCATATTCTGATGGGTGGGTGTGGTTAGTAGGCCCAGAAGGTACTGCAACATCTCATCTCTCTGAGGGAGGAGAGGGAGGGAGGAGAGGGAGAAGTACTCACCAGAATTTCATCGTTCACTGCACACAGAAACCCTAGAATAGCATCCAATTCGTCACTGAAAATGAATTCTGCAAAGTACAGATCAACAACTGTGGAGAAACAGCAACAGAATCAGTCGATTGTCCCACCATCTGGTACAATGTACACAACACACCATCACATCTGGTACACAACACACAATCACATCTGGTACACAACACATCATCACAGCTGGTACACAGCACACCATCACATCAAGGCAACACAGCTTGGCATAACTACCGGAGATGACTCACTGGACATTAAGGACTTCCTTATATGGACTGTACTCAGCTTTGGATTAATAGCAGATGATGGACTACAAGGGAGGTCATACATTAATTGGAAGACTAGTTAGTAAATGCCCTACTTGTAATACTCTCTGATGATGTCTAAAACAAACTGAACTCCAAAATCTCTCCTACAGTCGTCCAGCTTCAGACTGACTAGGGTTGACAAGTACTTGCTGTGACCTGGGAATGGAAACACAAGAATGGGACAAGAACGGCAAGCATTGGAATGTGACATAAGTGATTGTACAGGTCACTGACTCAGTTGCACAGAGTAGTCTGCTCTGCTCCATATCCTGTAGTTGAACAGCAGTGACGAATAGAGATCAGTCTGCTGCTCTGGCTGGTCTGGACTGAGCTTCTCAACAAATTTTTCGATCGCACTGTGAGCTGCCACAGTCAACAGAGAAGGAGGGACCTGGGCACACACACATATACATATACATACACACATCACACACACACACACACACACACACACANACACACAACAAATTAACTGAATACACATGTTGCTCACCTTACATAGAACATGTAGTCAGGGGCATAAGCATCATTGGGATCAGGGTCCAGCTGGGTCATCAGATCAAATGCTGTCTTCTGGTAAAGCAGAGGAAACCTGTTGTGCATCTCCAACCCTACAGGATGGGATACAGGCCTATCAGNNNNNNNNNNNNNNNNNNNNNNNNNNNNNNNNNNNNNNNNNNNNNNNNNNNNNNNNNNNNNNNNNNNNNNNNNNNNNNNNNNNNNNNNNNNNNNNNNNNNNNNNNNNNNNNNNNNNNNNNNNNNNNNNNNNNNNNNNNNNNNNNNNNNNNNNNNNNNNNNNNNNNNNNNNNNNNNNNNNNNNNNNNNNNNNNNNNNNNNNNNNNNNNNNNNNNNNNNNNNNNNNNNNNNNNNNNNNNNNNNNNNNNNNNNNNNNNNNNNNNNNNNNNNNNNNNNNNNNNNNNNNNNNNNNNNNNNNNNNNNNNNNNNNNNNNNNNNNNNNNNNNNNNNNNNNNNNNNNNNNNNNNNNNNNNNNNNNNNNNNNNNNNNNNNNNNNNNNNNNNNNNNNNNNNNNNNNNNNNNNNNNNNNNNNNNNNNNNNNNNNNNNNNNNNNNNNNNNNNNNNNNNNNNNNNNNNNNNNNNNNNNNNNNNNNNNNNNNNNNNNNNNNNNNNNNNNNNNNNNNNNNNNNNNNNNNNNNNNNNNNNNNNNNNNNNNNNNNNNNNNNNNNNNNNNNNNNNNNNNNNNNNNNNNNNNNNNNNNNNNNNNNNNNNNNNNNNNNNNNNNNNNNNNNNNNNNNNNNNNNNNNNNNNNNNNNNNNNNNNNNNNNNNNNNNNNNNNNNNNNNNNNNNNNNNNNNNNNNNNNNNNNNNNNNNNNNNNNNNNNNNNNNNNNNNNNNNNNNNNNNNNNNNNNNNNNNNNNNNNNNNNNNNNNNNNNNNNNNNNNNNNNNNNNNNNNNNNNNNNNNNNNNNNNNNNNNNNNNNNNNNNNNNNNNNNNNNNNNNNNNNNNNNNNNNNNNNNNNNNNNNNNNNNNNNNNNNNNNNNNNNNNNNNNNNNNNNNNNNNNNNNNNNNGGGACATGTGTACCATCATGTACAACATCATGGACATGTGTACCATCATGTACAACATCATGGGACATGTGTACCATCATGTACAACATCATGGGACATGTGTACCATCATGTACAACATCATGGGACATGTGTACCATCATGTACAACATACCGGGACATTTTTTCAATACAGCTCACCTGTTCTTCCATGCTCCACACTCCCCTATAAGGAACCTTCGTGTGCCACTCCATCTCTGTAAAGTTGCTGATAACTCCTGCCTGTATTGATTGTCTAATGTCTTTTGTCGAGACAGCTCCAATTCCCGCTGTGAGTAAAACTGCTGCGATACTGTTATCTGATATTTTTCAAGATCTGAGAACACTATTTCTTTTCTTTTTGCTTCTGCTTTCAAGCAGTCTTGCTTGCATTGCCTAGTATGGTCCGATAAGACCTTCAACCATTTGTAAACTACCTCAGAGTATTGCATTTGTATAGCCTGAAGCTGTGTTAGAGTGTTGAGAGATGGGAGAGGGCACTCACCCGACAACCTGCTTACCTCACCCAGCCACGTATGCCACTCCTCCCTCTTGGAATATTCAACCAGCTCGATAGTCTCATTGGTGAGATCTGGTAGATAAGAAGGCAAAGGAACCAGCTCCTTATTCCGATACAATTGAAAAAGGTGCTGAAGTAGAGTAGCCCTTGTTGTAGGAGGTTGATCTGAACACACCCAGTATAGAAAATAGGAAGCAAATAGATTAGCATGTTGGAAGAAAAAGACGAGAGGGTAAGAAGGGATAAAGGGAGCAGAACAAAGGAGAAGGAACAGACAGAATCATTCACTGACCACTGCTGAACTGGGTTGCAATGCACCTGAGCACANANACTGNNTCNNGTGCTTCCAGGAATTGAAGTTCTTGCAGCTTACAACCATACTGACAATACTGTGGTACAAAGAGGCCCATAAGAAGGAGAGGGTGAGTAAGGAGAGGGGAAAGGAAGGGGAGGAGAGACAGAATGCATAAAGAGGAGACAGAGCATAGAGTACTAGTAAAATTGTACCACACTGAGATAGCACTGAGAAAACAATATCCACTCACTAAACACCCTCTTCCTCTTACTACTACCCAATCACCTCTGCATCATCAGAGCAAGCACTGGCCAATAAGATTCGCAGCACCGACTGCATCAGGGCTGGTTCTATGGGTCCTTCCAGCACATGAGCTGCTCTCAACATATTCCAGTATTGTTCCAGCAGCTGAAAGGAAAACATTCAAGCCATTGAGGTGTTAAGTTACACTAACATACCTGCATAGAGCAGTGTTCTTTGTTGAGCACCAAGACCCCATTATGAGAGAACTTTTGAAGAATCACTTCTAAATCTGTTGAATGTGAAAGATATCAGGTGTGCAGTAGGGTGGTGTGGTGTGTGCAAGAGTAGTCAGGTGTGCAGTAGGGTGGTGTGGTGTGTGCAAGATATCAGGTGTACAGTAGGATGTGTGGAAGATGTGTACCTTCTACAACATCAAACGAGGGATAGCTTGAGATGATGAAAGCTTCAAGCAGTTGGGATAATTGAAGGATGACAGAGGATAACATGTTTTCAGGAGACCAGCTTGGAACTTGGAAAACAAGGGACCATAGCAGTGATGAACCAATGATGGCCTCCAGCTCCCATGCCTTCTGGCCCAAATCTTTTCCCAATTCCTTCTCCTCTCTCTTCAGCCCCCTTCCAGCAACCACTCTCCTTACCTGAGGAACACTCCAAGGCAACAGCATTCATCAACAGCAAGAAGATTCTAACAAGCAAAGCAACATGAATGAAATATATATAACCATCTCCAGTCCAGTATAAAAAGACATCTGGACCACCCAACAACACAACAAGACATCTGGACCACCCAATAACACAACAAGACATCTGGACTGTACCTTTGTTTGATCAGGTGGACTGGCACTAACAGTCTCTGCTCTGTTGCAAAGAGGTCCAACGCTGCCATAAACTGACCACATACCTATGTGGGAGGGGCCAAAGTGAGGACACACCCACAGACCACACCTACTTTTCTAGTTTGTCCTTCCGACTGCTCTGAGATGATGCAATGCCACAGAATGTAACCAATGGTCTTCATGACAACATCATACGTTTCCGAAGTGGCTGATTCAGCTTCTGTCTGGTTGTCCACGTCCGATTTGCAGGGGGTCAGTAGCCATAGCAACAACCTCTCCCACCCTTTGGACTCTGCCAGCTTGGCCACGTGGCTGGAGTTAAGATGGAGGATGGAGATGAAATCCTGACAAATTGTGTGTCGAATGGACATGGAGGCTGTTCGAAGCAGTCTTAGAGTGGCCAGGTAGACATCAAAGTTGGAGATGCCGCCTGAAACAGAATACGCACATTGCACATGCTGTCTGGTCGGTGTGGTTATCACATTTATGCACATTGCACACATGTTTGTGTTGCCAGCCGTACATGCTCCATTGTTTGTATTACATGTCTACGGGCATTGTATGCTGCACACAGGCACTGTACGTATACTTCACTGCACACGGCACTGTATGCTGCACATGGGCACTGTACATATACTTCACTGCACATGGGCACTGTATNCTGCACATGGGCACTGTATGCTGCACATNGGCACTGTACATATACTTCACTGCACACAGGCACTGTACGTAGCACATACCTGTTTGGTTGTCACTCTTCTTGACCATGCAGAGGTCTAGAAGGGCCAAAATGATGGACGAGGGCAGACACTGCTCATTCTGACACATGGCAGCAGCAAAGGCCCCCGAGTGGAGGGTGTTGTACACCACACCACTCTCAACGCTCCCTTGCTTTAAATTGAGCTGGGTCAGAATCTGCAAGCGTGGGGCGTGAGCACATGGTTTCACCACGCCCCCTCAACAGACCTACCCTCCACCACGCCCCCTCAACACACCCCCTCAACAGACCTACCCTCCACNACAACCCCCTCAAAAGACCTACCCTCAGAGTTGTGAGTCGCACGCCAAAGTCTTGCGTCTGAATTAAAANGAGNAAGACCACACCCTTTTCCCAGAGGGCTGTTGAGAGCATATTTTGATGNGTGGGTGTGGTTAGTAGGCCCAGAAGGAATCGAAGAACCTCATCTAACTGAATAGAGGAGTGGTTTTTTAAAGGAAGAACACACACACACATACATGCACACAACACATACACGCACGCACGCACGCANNNNNNNNNNNNNNNNNNNNNNNNNNNNNNNNNNNNNNNNNNNNNNNNNNNNNNNNNNNNNNNNNNNNNNNNNNNNNNNNNNNNNNNNNNNNNNNNNNNNNNNNNNNNNNNNNNNNNNNNNNNNNNNNNNNNNNNNNNNNNNNNNNNNNNNNNNNNNNNNNNNNNNNNNNNNNNNNNNNNNNNNNNNNNNNNNNNNNNNNNNNNNNNNNNNNNNNNNNNNNNNNNNNNNNNNNNNNNNNNNNNNNNNNNNNNNNNNNNNNNNNNNNNNNNNNNNNNNTGTGTGTGTATGTGTATGTGTATGTGTGCATGTGTGTGTGTGCATGTGGTGGTTCACAGTCATATGTTCATGCATTATCAACAGTCGAGGGCTGGTTTTCTCCATACTTTGTCTGGACCTTTTCCTCCAGGTCAGGGTTCTGTATCCCCATGGGTTTGGACAGGTCTCTGAACACCTCAGAGTTGCTCAGGTCCAATGTATCACAACTGTAGTCTCGCAGGATCCATGGAAACTAAACACGGAGCAGATACTAATTGATAATTTGCATACAGCTCAAGGATCCTCACAATAGGATATTGGTTGAGGTCATTGTAGGTCCTTCCTGATATAGTGTTGAGTTGCATGATGTAATCAAAATTTGTAATTTCACGTTTCACCCATTTCTATAACAAAATAAACCACATCATTCCAAGTGGAGATGAAGAGGGTAAGGAGATGGAGGTCATGGAAGAGGGGAAGGAGATGGAAGGTCATGGAAGAGGGTAAGGAGATGGAAGGTCATGGAAGAGAGTAAGGAGATGGAGGTCATGGAAGAGAGTAAGGAGATGGAAGGTCATGGAAGAGGGGAAGGAGATGGAAGGTCATGGAANNNNNNNNNNNNNNNNNNNNNNNNNNNNNNNNNNNNNNNNNNNNNNNNNNNNNNNNNNNNNNNNNNNNNNNNNNNNNNNNNNNNNNNNNNNNNNNNNNNNNNNNNNNNNNNNNNNNNNNNNNNNNNNNNNNNNNNNNNNNNNNNNNNNNNNNNNNNNNNNNNNNNNNNNNNNNNNNNNNNNNNNNNNNNNNNNNNNNNNNNNNNNNNNNNNNNNNNNNNNNNNNNNNNNNNNNNNNNNNNNNNNNNNNNNNNNNNNNNNNNNNNNNNNNNNNNNNNNNNNNNNNNNNNNNNNNNNNNNNNNNNNNNNNNNNNNNNNNNNNNNNNNNNNNNNNNNNNNNNNNNNNNNNNNNNNNNNNNNNNNNNNNNNNNNNNNNNNNNNNNNNNNNNNNNNNNNNNNNNNNNNNNNNNNNNNNNNNNNNNNNNNNNNNNNNNNNNNNNNNNNNNNNNNNNNNNNNNNNNNNNNNNNNNNNNNNNNNNNNNNNNNNNNNNNNNNNNNNNNNNNNNNNNNNNNNNNNNNNNNNNNNNNNNNNNNNNNNNNNNNNNNNNNNNNNNNNNNNNNNNNNNNNNNNNNNNNNNNNNNNNNNNNNNNNNNNNNNNNNNNNNNNNNNNNNNNNNNNNNNNNNNNNNNNNNNNNNNNNNNNNNNNNNNNNNNNNNNNNNNNNNNNNNNNNNNNNNNNNNNNNNNNNNNNNNNNNNNNNNNNNNNNNNNNNNNNNNNNNNNNNNNNNNNNNNNNNNNNNNNNNNNNNNNNNNNNNNNNNNNNNNNNNNNNNNNNNNNNNNNNNNNNNNNNNNNNNNNNNNNNNNNNNNNNNNNNNNNNNNNNNNNNNNNNNNNNNNNNNNNNNNNNNNNNNNNNNNNNNNNNNNNNNNNNNNNNNNNNNNNNNNNNNNNNNNNNNNNNNNNNNNNNNNNNNNNNNNNNNNNNNNNNNNNNNNNNNNNNNNNNNNNNNNNNNNNNNNNNNNNNNNNNNNNNNNNNNNNNNNNNNNNNNNNNNNNNNNNNNNNNNNNNNNNNNNNNNNNNNNNNNNNNNNNNNNNNNNNNNNNNNNNNNNNNNNNNNNNNNNNNNNNNNNNNNNNNNNNNNNNNNNNNNNNNNNNNNNNNNNNNNNNNNNNNNNNNNNNNNNNNNNNNNNNNNNNNNNNNNNNNNNNNNNNNNNNNNNNNNNNNNNNNNNNNNNNNNNNNNNNNNNNNNNNNNNNNNNNNNNNNNNNNNNNNNNNNNNNNNNNNNNNNNNNNNNNNNNNNNNNNNNNNNNNNNNNNNNNNNNNNNNNNNNNNNNNNNNNNNNNNNNNNNNNNNNNNNNNNNNNNNNNNNNNNNNNNNNNNNNNNNNNNNNNNNNNNNNNNNNNNNNNNNNNNNNNNNNNNNNNNNNNNNNNNNNNNNNNNNNNNNNNNNNNNNNNNNNNNNNNNNNNNNNNNNNNNNNNNNNNNNNNNNNNNNNNNNNNNNNNNNNNNNNNNNNNNNNNNNNNNNNNNNNNNNNNNNNNNNNNNNNNNNNNNNNNNNNNNNNNNNNNNNNNNNNNNNNNNNNNNNNNNNNNNNNNNNNNNNNNNNNNNNNNNNNNNNNNNNNNNNNNNNNNNNNNNNNNNNNNNNNNNNNNNNNNNNNNNNNNNNNNNNNNNNNNNNNNNNNNNNNNNNNNNNNNNNNNNNNNNNNNNNNNNNNNNNNNNNNNNNNNNNNNNNNNNNNNNNNNNNNNNNNNNNNNNNNNNNNNNNNNNNNNNNNNNNNNNNNNNNNNNNNNNNNNNNNNNNNNNNNNNNNNNNNNNNNNNNNNNNNNNNNNNNNNNNNNNNNNNNNNNNNNNNNNNNNNNNNNNNNNNNNNNNNNNNNNNNNNNNNNNNNNNNNNNNNNNNNNNNNNNNNNNNNNNNNNNNNNNNNNNNNNNNNNNNNNNNNNNNNNNNNNNNNNNNNNNNNNNNNNNNNNNNNNNNNNNNNNNNNNNNNNNNNNNNNNNNNNNNNNNNNNNNNNNNNNNNNNNNNNNNNNNNNNNNNNNNNNNNNNNNNNNNNNNNNNNNNNNNNNNNNNNNNNNNNNNNNNNNNNNNNNNNNNNNNNNNNNNNNNNNNNNNNNNNNNNNNNNNNNNNNNNNNNNNNNNNNNNNNNNNNNNNNNNNNNNNNNNNNNNNNNNNNNNNNNNNNNNNNNNNNNNNNNNNNNNNNNNNNNNNNNNNNNNNNNNNNNNNNNNNNNNNNNNNNNNNNNNNNNNNNNNNNNNNNNNNNNNNNNNNNNNNNNNNNNNNNNNNNNNNNNNNNNNNNNNNNNNNNNNNNNNNNNNNNNNNNNNNNNNNNNNNNNNNNNNNNNNNNNNNNNNNNNNNNNNNNNNNNNNNNNNNNNNNNNNNNNNNNNNNNNNNNNNNNNNNNNNNNNNNNNNNNNNNNNNNNNNNNNNNNNNNNNNNNNNNNNNNNNNNNNNNNNNNNNNNNNNNNNNNNNNNNNNNNNNNNNNNNNNNNNNNNNNNNNNNNNNNNNNNNNNNNNNNNNNNNNNNNNNNNNNNNNNNNNNNNNNCATACACACACCATCACACTGTACACACCATCACATGTACACACCATCACACTGTACACACCATCACACTGTACACACCATCACACTGTACACACCATCACACTGTACACACCATCACAACATACCAAGGCTTCTCGATGTTGTCTTATGAAGTCCTCAGGGGACGACGCCCACTTTGGAAGTATCACATCTCCAACAACTGTTTTGTCTTGGGTGACACCAAGGTCATAGCCTATAAAGACAAGTTAGCAGAGTGCTTAGAAATGAATGCAACCCTTTAAGTNATTAACTAGGCAAACANTGATGGNTATTNTAAGCAGTAATTACTGGATCAAAGTCACTTGAAAGTGAGACCAAATATTCAAGATGAACAACAAAAAAAAAANAAANAAAAAAANAAAAAAANAAATTTAGTTGTTAGTCCTTGAATAAATTAATTAATTAACTAGCAATTATTGAAGTTCACATTTTAATTTTTTGAGAGCTTTTTTGAAGCTTGAGGAGTTGGCTTTGTATTTCCCAAATATTTATTAAACCATTCCGCAATGTATTAATTATGAGTACTTATTGGAACAATTGACCTAACCATTTATATTCCTCAAGAAATCCGGTAGGCAGAAAAACTCTGGTGTAAGTTCTTTCACATCACTTCCTAAATAGGTGATTCTCCAAGCATTGGGTATGGAGAAGAACTGTCTATCAGGGTGATCAAACCTAGAGTATTAGCAATATAGCAGAGTGGGTAAGATTGAAGTGAAGATGGGTCAAGATACCATCCCAGCTGTATGTGGCACCTGTTCCCACACAACTCACTTTCCACTCTGTAGGTTGATGTGCAGGGATGTGAAGGGCTNCANTACGNNNNNAGTAGTGGATGACAGTTGTGGCATTGCTGTAGTGTGTNCCNTAGTGAAACTTGCCCATCACTGGATCGTCAAATTCCTCATATCTGCAAGTGCACAGTGCGCATAAGTGTTGTGTGTATAATTGACCACTGTGTCAACACCACATGACACTTTTAAATGGCTGGCGTCCATACTTTGTCCGGACCTTTTCCTCCAGGTCAGGGTTCTGTATCCCCATGGGTTTGGACAGGTCTCTGAACACCTCAGAGTTGCTCAGGTCCAATGTATCACAACTGTAGTCTTGCAGGATCCATGGAAACTAAACACGGAGCAGATACTAAATGATAATTTGCATACAGCTCAAGTATCCTCACAATAGGATATTGGTTGAGGTCATTGTAGGTCCTTCCTGATATAGTGTTGAGTTGCATGATGTAATCAAAATTTGTAATTTCACGTTTCACCCATTTCTACAGTTGTGCAGTTGAATAATGAAAATGAAAGCAGTAGTGAAGGTGGTCATGGAGGAGGTAAGGAATGAGGGAGAGGTGGTCATGGAGGAGGTAAGGGGGAGAGGTGGTCATGGAGGAGGTAAGGGGGAGAGGTGGCCATGGAGGAGGTAAGGATGTAAGGGAGGAGGTGGAGGTGGTGAGGGAGGAGAAAAATTAACACAAGGTGTACAGACAGGGTATAACGACAGTTAGTGTATTCCCAACCTGAATTAGACCTGAAGACTGCATCAGTTTGGCAGGCTCTTGTGTGCTGAAGATGTTAAGGTTTGGAGTACTCAGCGAAACAATGCAGTTGCACACCTTATTCAAAATCTGTGCAACAAGGAGAGAGATACATGCACACCACTTAACACCCAGTCTGGAGACAATGAAAACACTGTGCCTTTTTGGTGGGAAAATTAAGGAAGTAGTTTGTCTGATCCAAAAGAAACATCTCCAGTGCAGTACGGCTTGTGTTGTACCTACGAAGGTGAAGCTCCCTTATCTGGTTAAGTTTGACTGTGAAGTCAGTGAGGTACCCTGCGAAAGAAAGAGAAAATGAATAACCATTGGAGCCTTGCCTTAGGAAGAGGCACTAATAGTTCAATGTCAGATATATCTGGTCACATGTCTTAGCACAAAGTCAACTGTCATACACCTATGCTATTCTAAGTAGTGCACACTCTTGTTACAGATATGCTACTCCATGTTTACAGATATGCTACTCCAAGTGCACACNNTTGTTACAGATATGCTACTCCAAGTGCACACTTTTGTTACAGATATGCTACTACAAGTGCACACTTTTGTTACAGATATGCTACTCCAAGTGCACACTCTTGTTCCTCCCTTCTTCATTTCCTCCTCCCTATCTGTGATCACACCACCCCTTTAACTCTCCCGGTACACTCACAAGATATCGCATCCTTGTCTTGTGCATCCGACACAAAGTAGATGCATCTATTTGTAATGTCCAAGGTTCCAGAGATGACCTCTACCACCGTCACAAGCTGACACATCTCTCTTATNATCAGTTTCTCTTTCTTGCCCTTCTCCAGAGCCACAGCTGGCTCATCTCGAGCTAGCTTTTGGATCTGATCAAACAGATGTTCCTCCTCCTCCTCACTGGNGGACTTTGTAGCTGTGGCCATGGCTAGCAGAGAGGTGTCTGCAGTGCTGGACACCAGGNGAGTCTCCCCAGAGCTGTGTTGACTAGCATCGGTGTGTTTGTTATAGTTGTAGTTACGGGCCAGTTTTAGCCGCATGCGTGAAAAGTTCTCTGTTCTGTCCATCTTCCACAGGTAGAGATCAGGATCTCTGGAACAGAGGATCACGGAATCACAGGGTTATGTACATATACATTCATGTGTGCCACACAGCACCACACAGAGGTACATACATGTATGTACTGTTCCATCATGTATAAAACATGAGTACAACTTCATGTACAACATCATGGGACATGTGTCCCCATGGAACATGTGTACCCATGTACAACATCATGGAACATGTGTACCATCATGTACAACATATGTACCATCAATGTACTANATACTGGCACATAGTGGGGACATGTGTACCATCATGTACNNNNNNNNNNNNNNNNNNNNNNNNNNNNNNNNNNNNNNNNNNNNNNNNNNNNNNNNNNNNNNNNNNNNNNNNNNNNNNNNNNNNNNNNNNNNNNNNNNNNNNNNNNNNNNNNNNNNNNNNNNNNNNNNNNNNNNNNNNNNNNNNNNNNNNNNNNNNNNNNNNNNNNNNNNNNNNNNNNNNNNNNNNNNNNNNNNNNNNNNNNNNNNNNNNNNNNNNNNNNNNNNNNNNNNNNNNNNNNNNNNNNNNNNNNNNNNNNNNNNNNNNNNNNNNNNNNNNNNNNNNNNNNNNNNNNNNNNNNNNNNNNNNNNNNNNNNNNNNNNNNNNNNNNNNNNNNNNNNNNNNNNNNNNNNNNNNNNNNNNNNNNNNNNNNNNNNNNNNNNNNNNNNNNNNNNNNNNNNNNNNNNNNNNNNNNNNNNNNNNNNNNNNNNNNNNNNNNNNNNNNNNNNNNNNNNNNNNNNNNNNNNNNNNNNNNNNNNNNNNNNNNNNNNNNNNNNNNNNNNNNNNNNNNNNNNNNNNNNNNNNNNNNNNNNNNNNNNNNNNNNNNNNNNNNNNNNNNNNNNNNNNNNNNNNNNNNNNNNNNNNNNNNNNNNNNNNNNNNNNNNNNNNNNNNNNNNNNNNNNNNNNNNNNNNNNNNNNNNNNNNNNNNNNNNNNNNNNNNNNNNNNNNNNNNNNNNNNNNNNNNNNNNNNNNNNNNNNNNNNNNNNNNNNNNNNNNNNNNNNNNNNNNNNNNNNNNNNNNNNNNNNNNNNNNNNNNNNNNNNNNNNNNNNNNNNNNNNNNNNNNNNNNNNNNNNNNNNNNNNNNNNNNNNNNNNNNNNNNNNNNNNNNNNNNNNNNNNNNNNNNNNNNNNNNNNNNNNNNNNNNNNNNNNNNNNNNNNNNNNNNNNNNNNNNNNNNNNNNNNNNNNNNNNNNNNNNNNNNNNNNNNNNNNNNNNNNNNNNNNNNNNNNNNNNNNNNNNNNNNNNNNNNNNNNNNNNNNNNNNNNNNNNNNNNNNNNNNNNNNNNNNNNNNNNNNNNNNNNNNNNNNNNNNNNNNNNNNNNNNNNNNNNNNNNNNNNNNNNNNNNNNNNNNNNNNNNNNNNNNNNNNNNNNNNNNNNNNNNNNNNNNNNNNNNNNNNNNNNNNNNNNNNNNNNNNNNNNNNNNNNNNNNNNNNNNNNNNNNNNNNNNNNNNNNNNNNNNNNNNNNNNNNNNNNNNNNNNNNNNNNNNNNNNNNNNNNNNNNNNNNNNNNNNNNNNNNNNNNNNNNNNNNNNNNNNNNNNNNNNNNNNNNNNNNNNNNNNNNNNNNNNNNNNNNNNNNNNNNNNNNNNNNNNNNNNNNNNNNNNNNNNNNNNNNNNNNNNNNNNNNNNNNNNNNNNNNNNNNNNNNNNNNNNNNNNNNNNNNNNNNNNNNNNNNNNNNNNNNNNNNNNNNNNNNNNNNNNNNNNNNNNNNNNNNNNNNNNNNNNNNNNNNNNNNNNNNNNNNNNNNNNNNNNNNNNNNNNNNNNNNNNNNNNNNNNNNNNNNNNNNNNNNNNNNNNNNNNNNNNNNNNNNNNNNNNNNNNNNNNNNNNNNNNNNNNNNNNNNNNNNNNNNNNNNNNNNNNNNNNNNNNNNNNNNNNNNNNNNNNNNNNNNNNNNNNNNNNNNNNNNNNNNNNNNNNNNNNNNNNNNNNNNNNNNNNNNNNNNNNNNNNNNNNNNNNNNNNNNNNNNNNNNNNNNNNNNNNNNNNNNNNNNNNNNNNNNNNNNNNNNNNNNNNNNNNNNNNNNNNNNNNNNNNNNNNNNNNNNNNNNNNNNNNNNNNNNNNNNNNNNNNNNNNNNNNNNNNNNNNNNNNNNNNNNNNNNNNNNNNNNNNNNNNNNNNNNNNNNNNNNNNNNNNNNNNNNNNNNNNNNNNNNNNNNNNNNNNNNNNNNNNNNNNNNNNNNNNNNNNNNNNNNNNNNNNNNNNNNNNNNNNNNNNNNNNNNNNNNNNNNNNNNNNNNNNNNNNNNNNNNNNNNNNNNNNNNNNNNNNNNNNNNNNNNNNNNNNNNNNNNNNNNNNNNNNNNNNNNNNNNNNNNNNNNNNNNNNNNNNNNNNNNNNNNNNNNNNNNNNNNNNNNNNNNNNNNNNNNNNNNNNNNNNNNNNNNNNNNNNNNNNNNNNNNNNNNNNNNNNNNNNNNNNNNNNNNNNNNNNNNNNNNNNNNNNNNNNNNNNNNNNNNNNNNNNNNNNNNNNNNNNNNNNNNNNNNNNNNNNNNNNNNNNNNNNNNNNNNNNNNNNNNNNNNNNNNNNNNNNNNNNNNNNNNNNNNNNNNNNNNNNNNNNNNNNNNNNNNNNNNNNNNNNNNNNNNNNNNNNNNNNNNNNNNNNNNNNNNNNNNNNNNNNNNNNNNNNNNNNNNNNNNNNNNNNNNNNNNNNNNNNNNNNNNNNNNNNNNNNNNNNNNNNNNNNNNNNNNNNNNNNNNNNNNNNNNNNNNNNNNNNNNNNNNNNNNNNNNNNNNNNNNNNNNNNNNNNNNNNNNNNNNNNNNNNNNNNNNNNNNNNNNNNNNNNNNNNNNNNNNNNNNNNNNNNNNNNNNNNNNNNNNNNNNNNNNNNNNNNNNNNNNNNNNNNNNNNNNNNNNNNNNNNNNNNNNNNNNNNNNNNNNNNNNNNNNNNNNNNNNNNNNNNNNNNNNNNNNNNNNNNNNNNNNNNNNNNNNNNNNNNNNNNNNNNNNNNNNNNNNNNNNNNNNNNNNNNNNNNNNNNNNNNNNNNNNNNNNNNNNNNNNNNNNNNNNNNNNNNNNNNNNNNNNNNNNNNNNNNNNNNNNNNNNNNNNNNNNNNNNNNNNNNNNNNNNNNNNNNNNNNNNNNNNNNNNNNNNNNNNNNNNNNNNNNNNNNNNNNNNNNNNNNNNNNNNNNNNNNNNNNNNNNNNNNNNNNNNNNNNNNNNNNNNNNNNNNNNNNNNNNNNNNNNNNNNNNNNNNNNNNNNNNNNNNNNNNNNNNNNNNNNNNNNNNNNNNNNNNNNNNNNNNNNNNNNNNNNNNNNNNNNNNNNNNNNNNNNNNNNNNNNNNNNNNNNNNNNNNNNNNNNNNNNNNNNNNNNNNNNNNNNNNNNNNNNNNNNNNNNNNNNNNNNNNNNNNNNNNNNNNNNNNNNNNNNNNNNNNNNNNNNNNNNNNNNNNNNNNNNNNNNNNNNNNNNNNNNNNNNNNNNNNNNNNNNNNNNNNNNNNNNNNNNNNNNNNNNNNNNNNNNNNNNNNNNNNNNNNNNNNNNNNNNNNNNNNNNNNNNNNNNNNNNNNNNNNNNNNNNNNNNNNNNNNNNNNNNNNNNNNNNNNNNNNNNNNNNNNNNNNNNNNNNNNNNNNNNNNNNNNNNNNNNNNNNNNNNNNNNNNNNNNNNNNNNNNNNNNNNNNNNNNNNNNNNNNNNNNNNNNNNNNNNNNNNNNNNNNNNNNNNNNNNNNNNNNNNNNNNNNNNNNNNNNNNNNNNNNNNNNNNNNNNNNNNNNNNNNNNNNNNNNNNNNNNNNNNNNNNNNNNNNNNNNNNNNNNNNNNNNNNNNNNNNNNNNNNNNNNNNNNNNNNNNNNNNNNNNNNNNNNNNNNNNNNNNNNNNNNNNNNNNNNNNNNNNNNNNNNNNNNNNNNNNNNNNNNNNNNNNNNNNNNNNNNNNNNNNNNNNNNNNNNNNNNNNNNNNNNNNNNNNNNNNNNNNNNNNNNNNNNNNNNNNNNNNNNNNNNNNNNNNNNNNNNNNNNNNNNNNNNNNNNNNNNNNNNNNNNNNNNNNNNNNNNNNNNNNNNNNNNNNNNNNNNNNNNNNNNNNNNNNNNNNNNNNNNNNNNNNNNNNNNNNNNNNNNNNNNNNNNNNNNNNNNNNNNNNNNNNNNNNNNNNNNNNNNNNNNNNNNNNNNNNNNNNNNNNNNNNNNNNNNNNNNNNNNNNNNNNNNNNNNNNNNNNNNNNNNNNNNNNNNNNNNNNNNNNNNNNNNNNNNNNNNNNNNNNNNNNNNNNNNNNNNNNNNNNNNNNNNNNNNNNNNNNNNNNNNNNNNNNNNNNNNNNNNNNNNNNNNNNNNNNNNNNNNNNNNNNNNNNNNNNNNNNNNNNNNNNNNNNNNNNNNNNNNNNNNNNNNNNNNNNNNNNNNNNNNNNNNNNNNNNNNNNNNNNNNNNNNNNNNNNNNNNNNNNNNNNNNNNNNNNNNNNNNNNNNNNNNNNNNNNNNNNNNNNNNNNNNNNNNNNNNNNNNNNNNNNNNNNNNNNNNNNNNNNNNNNNNNNNNNNNNNNNNNNNNNNNNNNNNNNNNNNNNNNNNNNNNNNNNNNNNNNNNNNNNNNNNNNNNNNNNNNNNNNNNNNNNNNNNNNNNNNNNNNNNNNNNNNNNNNNNNNNNNNNNNNNNNNNNNNNNNNNNCCACACACACATACCCCACNCACACACACACACACACACACACACACACCACACACAAACACACACCAATGAAGCAGCTGTGGGGTTGCTCTACAAATGGAGTACCTCTACAACATTAGCCATGAGGTCATCTCTCGTTTGACCTCTTTGATCGTCCCCCTTACGCACCACCTGCATTTGCTCAAAGAGGTTCACTATGACTCTCTTGCCAGTGAAGGGAGAGGAGTAGCCTCTGTAGCTCTTCACAGGCGGGTCTCTTGTCGGATGCCTCTCGTTAAACAGACGAGTTGGTGTCTGACCAAAATTGGTTATAATACTTTCAATCTCTCGGCGGCTTTTTGGGTCTTGGATGTTGTCTAAGTTGACATTTTCTTTATAAGGGAAGTGAAAATATTAACAACATTGAAGATTAGACAATAAGGTCCCAATTATCCATGTACCTTGGTATGAGTACTTGTTAAACACATTCATAGCCTTTTCTGCCTCAGGCCCAGTCTGTTTGTACCCAAAAATGAGGTCAATCCACTCATTGAGGTGTGCTGAAACGTATTCAGANTCCTGAGGGAGGAGGGTAGAGCACAATGNACACACATGCTCCTACNNGNNATTACACTGTACACACCATCACACTGTACACACCATCACAC>scaffold11303|size3085CATGCATTACATCATCATGCATTACATCATCATGCATTACATCATCATGCANTACAACATAGTGCTCAGTCAACTGCTTCCCTACAAATCCCATCTTTGACCCTCCCACTCTCCCTCACTCTGCTCTACTGCTTGAACTCCTCCCCAGCCCTTACCTTAAAAGGGTACTCCTCGAACATATGTGCATTAGGGGCAGGGATTGTCTCTATGGAAACAGATCCCATTATCGTTTCTTTCACATATGCAACTGGGTCAAACGGTTCCTGAGCAGCTGAATTGGTCTGGGACAGCAATCCCTGCTGCTTCCCATGTAGCAGTACATCTTGCATCTGAACAAAACTCTTCAGTCCTGTGTTCATGACAGTCACCATGTTCTCCACCAGTGTCTGGAGAGAACACAGAGTAGAGGGCAGCTGTGTGACAAATACTGAGCTCTGCAGGGTGTTGAGGCTGTTCACAGCTACATCGTACTCCAGACGAGCCTGAATGGGGAGGAGGAACGTGAGGTCATGATGAGTACAGGTCAGGATGGGTACATGAGGTCATGATGGGTACATGAGGTCACGACGGGTACATGAGGTCATGATGGGTACATGAGGTCATGATGGGTACATGACGGGTACATGAGGTCATGATGGGTACATGAGGTCATGATGGGTACATGAGGTCATGACGGGTACATGGTCTGCACCTCTTGGGCTTTCTTGCACATAACAGCTTCTCTCTCGTACAGCTTATTACCAGGCTGCATTGACCAGTTGTCTCCAAACTCTTNGGTCATAAGAGATGATTCCCAGTCTGCTGAACAGCTGGTGAGTGCATATCATGTAACCATGGCAACTACATGTAGCATCATACCTGTAATAGCGCTCTCTTGCTCTGCTTAGCTGCAAAACAGAGGTAAAGTGGTCTTTAATTAGCTGGCGACCATCATGGGATATCTGAAAATGAGAAGCAATATGCATCACTGTGCCATCTTCTCCCCCACCCTCCCCCTCCTCCCTCTTCACCTCCTCCCACTCCACCTCCTCCCCCTACCTCTCCGTCCACCCTCCCCCTCCTCCCTCTCCCTCCCTCACTCCTCTCACCATTCTAAATCTCTTCTCAAGATCCACTAGCATGTACACTTGCAAGGGTTGAAGCGCTTGGAGTTCCAGGTTTGATGATACGTTGGAGCGATGTAAGGCTTCGGCCTGGATCTGCTCTTTGAGCGAGCTCCACTGANNCNNNNATCGCACTGATCGGAAGTGTGGTCAGTGCGGTAGTGCTTCGGTGTGAGCGTGCGTACATGCCAAACTTACCCATGAAACCTCATCTTATTGAATTGGGCCTCTTCCGATATGGATAAGGACAATTTCTGAAGCGCCTTGATGTTGTCTCGATCCACGGCTATCCTCTTCTTCAATAGACTGACGATCCCCTACGCGATGAATAAAGTCCAGGTGACACCGCACTGGAGTAAGGTGAGCCTCTATTCTGTACCTTGGCATTTTTGATCAGGTCTTCGACACGGGATACAACTGTTTCGAAGTATTCCTGAACATTAGAGATATAATTCTCACTGAACTTTTAAGCTCAATTTCCCATCCGTCATACCGCTAGCAAAAGACGTCCCTCGTCATTCGAATGGCTCNNNNNNNTGCNGNNCATGCTCATCTGGTCTGTCCTTCGCCACGCCCCTATCAATAAAGTAGGTCACGTGATAATAGAATAGAATAATGTACTAGGTTTCGTTCCCATCCCCTATTTGGAGACAGGCAACTGAACCCTAGTTACCGCATGGAACTGCAATGTGGCTTATCCTTCTATATCCGTACACTCACTGTGAGTTGATATTTCGGTCANTTCGTATATCCGTACACTCACTGTGAGTTGATATTTCGGTCAGGCGCCCTCCCTTCGACGACTAAAACAGTATAAGTTTCANATTTANATACGCGATAAGAGAGGGCTGAGACTAAATGCGCTTTATTTTTGAGTTGTCCGAGTCACACAGAAATCCACGAGGCCAACTTAAAAAATCTCGTTTCGAAATGTGTACTGGAAGAGATGGTTGTATGTGTTTCCCCTTAGNGNNTAGGGTGTTCTGGGGCATATTTTCTTTGCNTCGTACCAAAGCTTTGAATAAGGCAGATTATTTACATAGCGGAGTGGTAGTCCGCTCACAATGTATTAAATACTGTCACTTGTATATGCATATATGAAATAAAATCCACGAGTACACNTTAGTGTACTTTATTCATTATGTTTTCTTTTTCTGCATGCTTGGTTTTTCTGGCCTCATAGACTATAGTCAGGGACAGTGTTCTTGCATGATGCACTTCAGCCTCTGCAGTTTGTTCANCATACTGCCATATGTCCTGTACACTTTATTGAAGTGGGCATGTATTGCTTCAGTGCCCTGTTCTCCCATTAGCCCAAATCCTGCACTCATCCATTCACTGATGTGCATTTCTAAGATATGCATTTNAACTGTNCTCTTTGTTCCAGATGGTCAAAGTAACTGAAGAATTCTCGTATGGATTCTGCTGTAACAAANACAACCAGATGTATTAGTATTGGCAATGCATGTTCTGATCATACCAAGTGCACTCCGGACTGAGAAAACAATGAAAAGGCTCTCTTTTATTTCTCGCAAGTAGCAGTTGCTTCCTCAACCATTTGGGGTGAGTGCTTTTGTGCAATGTCAATGAGGGATGTGCAACAGTAGGTTATATTTCAGTTTATATTTATTGTTTGATTTTACCTGCAATAACTTGTTTACGTGGTTGCCAATGAATTCTCCACTGTGATACTGTTGGCATTGTACATTTAGAGACGCGAGAGCTGTTTCCACTACCTTCACNAAGGGTCCCTCAATCACTTCAAACCCTTTTTGCAGCCTCAGATTGTCATCAGAATCCTTTGTTCCTACATGCAATAGGTATGTGCCAATCAGACCCGTCACTAGATTTGAGAGTAAGGAGGTTAATAAATAGGTGCAGGTATATTGTCGTATAGGAAAATGGGTCTAAGGCAAGTTATGTAACTCTCACAACAGGACCCCCCCCNCATCCTACCAAATACTGTACCGTAAAGTACCGCG>scaffold4236|size40413CCACCACCACCACCACCACCACCACCACCACCACCTCCTCCAGCATGGCCGGTATTAACTGCAGAATGGCGGGCACCCTCACTTCCATCCAGCATGAGGCCGAGGTGGAGACCGGGAAGCCGGGGATGTGGGCCAAAGAGGAGACTCAATAGCTGTATGCACAATACTTGATCATTATTGTACTAAATTATCCAAGAGATGGTTATGGTGCATACGAAATGCGCAATATTATCCGTCAGTTTATGCTTGACATAGATGCATGCATGGCATTAAAGTGCCAGTGTTGGCTTTAAGAGACTGTTGGATTGATGTCTGATCGATCTGGAGAACAGGACAAAGAGACAGAATCCAAACGGACTCACAATAGTATATAAAGTGGGTAGTTCCCAATGCAGTGCTACAAGATGACGAGCAAGAGAAGAAGCGTGCGTATGAAGAGAGGGTGAGAGAAATCGAGCATGGCTCCTTTGCACCCCTCGTAGTCTCAATCTCAGGAGGTATGGGTCCGATAGCCACAACCGTGTACAAAAGAATGGCCTCGCTCATAGCTCAGTGAAAAATACAATAATCCATATATCTTTTTTGGCTAAGATGCAAGCTGAGTTTCTCCTTATTGCGATCTGCATAACACATGTGCATCAGAGGCTCGAGGTCCTCCTATCACAGACCAACAAACATGTTTAGTGAGACCATTGTCCTATCCTGCTTGGTAAACAGGATCTCAATTTGAACATTGAAATCCTATCCGTCTTTTTTCTTTTATTNNNTNCTTANNTCATTACCACGTTTCCACGCATTATGTGTATATTTTTATTTAAAAAGCGTATACTTTACGGACTTATCTGCTGTACTGATTTGATTTATTGTCAGTTCAAAGCGATAGTCTCGTACCCAGACCTGACGGACGTAAAAGCATGTTTGCACGTCAGCCGAGTCTGGGTANNNNNNNNNNGGGGGCGGGGCCTAAATATACTACGGCCTGTCCGGGCCATGTAAACTTAAAAGACGTTACGAAAGTGGAAAAAAAAACTTACGAAATAAGTAAATTAAAACATTCTCTATACGCGAAAGTTGGCTCTCCTCCTCTCTTCCTGCCACTCCTCNTACCCCCATTGTCATCTTGTAACCATTACTGCCGATACATACATGATCACTTGCACAAGATTTAGTCACTATAAACTTATTGTGTAATAACTATTGCATAAAGTAGTTAAACGTGGTTAATTTCGGGTTTCCTCCCTCATCTCAAGTCACATGTTCTCATGTTCACGCCACGAGCCAGGCAGAAAACAGCATTTTTATTCATTGTCATTATTTTTATTTTATTAATTTTAGATTANCCATTACAATAAATAAATACATACATAATCAATAAACATTTGAGCACATAATACTACAAACCTACCTACCTAGGACNCNAAGAGTCCTTATCAGTTATTGAAATGACGACGACNNAGCANNNNCNCNNACCAGGGCAATGCTTAGCCTTCCATATAGCCCAGACACTGCTGAAGACATGGGACAAGCAGTTCGAGTGGCGATCCATGTAGCGAGTCTGTCGAGGCACCTGCTGNTANGTGTAAGCTCAGCTGGGGAGTTGGCGTGACCAAAAGCCAAGCTGTGGCGTGGGGAGAAGACACAGAGGGAAACCGNNNNNNNNNNNNNNNNNNNNNNNNNNNNNNNNNNNNNNNNNNNNNNNNNNNNNNNNNNNNNNNNNNNNNNNNNNNNNNNNNNNNNNNNNNNNNNNNNNNNNNNNNNNNNNNNNNNNNNNNNNNNNNNNNNNNNNNNNNNNNNNNNNNNNNNNNNNNNNNNNNNNNNNNNNNNNNNNNNNNNNNNNNNNNNNNNNNNNNNNNNNNNNNNNNNNNNNNNNNNNNNNNNNNNNNNNNNNNNNNNNNNNNNNNNNNNNNNNNNNNNNNNNNNNNNNNNNNNNNNNNNNNNNNNNNNNNNNNNNNNNNNNNNNNNNNNNNNNNNNNNNNNNNNNNNNNNNNNNNNNNNNNNNNNNNNNNNNNNNNNNNNNNNNNNNNNNNNNNNNNNNNNNNNNNNNNNNNNNNNNNNNNNNNNNNNNNNNNNNNNNNNNNNNNNNNNNNNNNNNNNNNNNNNNNNNNNNNNNNNNNNNNNNNNNNNNNNNNNNNNNNNNNNNNNNNNNNNNNNNNNNNNNNNNNNNNNNNNNNNNNNNNNNNNNNNNNNNNNNNNNNNNNNNNNNNNNNNNNNNNNNNNNNNNNNNNNNNNNNNNNNNNNNNNNNNNNNNNNNNNNNNNNNNNNNNNNNNNNNNNNNNNNNNNNNNNNNNNNNNNNNNNNNNNNNNNNNNNNNNNNNNNNNNNNNNNNNNNNNNNNNNNNNNNNNNNNNNNNNNNNNNNNNNNNNNNNNNNNNNNNNNNNNNNNNNNNNNNNNNNNNNNNNNNNNNNNNNNNNNNNNNNNNNNNNNNNNNNNNNNNNNNNNNNNNNNNNNNNNNNNNNNNNNNNNNNNNNNNNNNNNNNNNNNNNNNNNNNNNNNNNNNNNNNNNNNNNNNNNNNNNNNNNNNNNNNNNNNNNNNNNNNNNNNNNNNNNNNNNNNNNNNNNNNNNNNNNNNNNNNNNNNNNNNNNNNNNNNNNNNNNNNNNNNNNNNNNNNNNNNNNNNNNNNNNNNNNNNNNNNNNNNNNNNNNNNNNNNNNNNNNNNNNNNNNNNNNNNNNNNNNNNNNNNNNNNNNNNNNNNNNNNNNNNNNNNNNNNNNNNNNNNNNNNNNNNNNNNNNNNNNNNNNNNNNNNNNNNNNNNNNNNNNNNNNNNNNNNNNNNNNNNNNNNNNNNNNNNNNNNNNNNNNNNNNNNNNNNNNNNNNNNNNNNNNNNNNNNNNNNNNNNNNNNNNNNNNNNNNNNNNNNNNNNNNNNNNNNNNNNNNNNNNNNNNNNNNNNNNNNNNNNNNNNNNNNNNNNNNNNNNNNNNNNNNNNNNNNNNNNNNNNNNNNNNNNNNNNNNNNNNNNNNNNNNNNNNNNNNNNNNNNNNNNNNNNNNNNNNNNNNNNNNNNNNNNNNNNNNNNNNNNNNNNNNNNNNNNNNNNNNNNNNNNNNNNNNNNNNNNNNNNNNNNNNNNNNNNNNNNNNNNNNNNNNNNNNNNNNNNNNNNNNNNNNNNNNNNNNNNNNNNNNNNNNNNNNNNNNNNNNNNNNNNNNNNNNNNNNNNNNNNNNNNNNNNNNNNNNNNNNNNNNNNNNNNNNNNNNNNNNNNNNNNNNNNNNNNNNNNNNNNNNNNNNNNNNNNNNNNNNNNNNNNNNNNNNNNNNNNNNNNNNNNNNNNNNNNNNNNNNNNNNNNNNNNNNNNNNNNNNNNNNNNNNNNNNNNNNNNNNNNNNNNNNNNNNNNNNNNNNNNNNNNNNNNNNNNNNNNNNNNNNNNNNNNNNNNNNNNNNNNNNNNNNNNNNNNNNNNNNNNNNNNNNNNNNNNNNNNNNNNNNNNNNNNNNNNNNNNNNNNNNNNNNNNNNNNNNNNNNNNNNNNNNNNNNNNNNNNNNNNNNNNNNNNNNNNNNNNNNNNNNNNNNNNNNNNNNNNNNNNNNNNNNNNNNNNNNNNNNNNNNNNNNNNNNNNNNNNNNNNNNNNNNNNNNNNNNNNNNNNNNNNNNNNNNNNNNNNNNNNNNNNNNNNNNNNNNNNNNNNNNNNNNNNNNNNNNNNNNNNNNNNNNNNNNNNNNNNNNNNNNNNNNNNNNNNNNNNNNNNNNNNNNNNNNNNNNNNNNNNNNNNNNNNNNNNNNNNNNNNNNNNNNNNNNNNNNNNNNNNNNNNNNNNNNNNNNNNNNNNNNNNNNNNNNNNNNNNNNNNNNNNNNNNNNNNNNNNNNNNNNNNNNNNNNNNNNNNNNNNNNNNNNNNNNNNNNNNNNNNNNNNNNNNNNNNNNNNNNNNNNNNNNNNNNNNNNNNNNNNNNNNNNNNNNNNNNNNNNNNNNNNNNNNNNNNNNNNNNNNNNNNNNNNNNNNNNNNNNNNNNNNNNNNNNNNNNNNNNNNNNNNNNNNNNNNNNNNNNNNNNNNNNNNNNNNNNNNNNNNNNNNNNNNNNNNNNNNNNNNNNNNNNNNNNNNNNNNNNNNNNNNNNNNNNNNNNNNNNNNNNNNNNNNNNNNNNNNNNNNNNNNNNNNNNNNNNNNNNNNNNNNNNNNNNNNNNNNNNNNNNNNNNNNNNNNNNNNNNNNNNNNNNNNNNNNNNNNNNNNNNNNNNNNNNNNNNNNNNNNNNNNNNNNNNNNNNNNNNNNNNNNNNNNNNNNNNNNNNNNNNNNNNNNNNNNNNNNNNNNNNNNNNNNNNNNNNNNNNNNNNNNNNNNNNNNNNNNNNNNNNNNNNNNNNNNNNNNNNNNNNNNNNNNNNNNNNNNNNNNNNNNNNNNNNNNNNNNNNNNNNNNNNNNNNNNNNNNNNNNNNNNNNNNNNNNNNNNNNNNNNNNNNNNNNNNNNNNNNNNNNNNNNNNNNNNNNNNNNNNNNNNNNNNNNNNNNNNNNNNNNNNNNNNNNNNNNNNNNNNNNNNNNNNNNNNNNNNNNNNNNNNNNNNNNNNNNNNNNNNNNNNNNNNNNNNNNNNNNNNNNNNNNNNNNNNNNNNNNNNNNNNNNNNNNNNNNNNNNNNNNNNNNNNNNNNNNNNNNNNNNNNNNNNNNNNNNNNNNNNNNNNNNNNNNNNNNNNNNNNNNNNNNNNNNNNNNNNNNNNNNNNNNNNNNNNNNNNNNNNNNNNNNNNNNNNNNNNNNNNNNNNNNNNNNNNNNNNNNNNNNNNNNNNNNNNNNNNNNNNNNNNNNNNNNNNNNNNNNNNNNNNNNNNNNNNNNNNNNNNNNNNNNNNNNNNNNNNNNNNNNNNNNNNNNNNNNNNNNNNNNNNNNNNNNNNNNNNNNNNNNNNNNNNNNNNNNNNNNNNNNNNNNNNNNNNNNNNNNNNNNNNNNNNNNNNNNNNNNNNNNNNNNNNNNNNNNNNNNNNNNNNNNNNNNNNNNNNNNNNNNNNNNNNNNNNNNNNNNNNNNNNNNNNNNNNNNNNNNNNNNNNNNNNNNNNNNNNNNNNNNNNNNNNNNNNNNNNNNNNNNNNNNNNNNNNNNNNNNNNNNNNNNNNNNNNNNNNNNNNNNNNNNNNNNNNNNNNNNNNNNNNNNNNNNNNNNNNNNNNNNNNNNNNNNNNNNNNNNNNNNNNNNNNNNNNNNNNNNNNNNNNNNNNNNNNNNNNNNNNNNNNNNNNNNNNNNNNNNNNNNNNNNNNNNNNNNNNNNNNNNNNNNCCAGCCAATAACAGCAAGCACACGGGNCTNGNNTNNACNNGTNTATANATNNATGGCCACAAGCTGGCCAACCCAAGGCTGCATTGGAAGCAATAGGGGCAAACACAAGAGGGTGGGGCGTATATCAAAGGGGAGGCCAGTTGCAGAATGTGAACGGGGGAATTTCGAGAGCCTAAACAGTACCATAAGGGTTAGAAGCACTGAGTATGTACAGTGTATGTGTCAACGTTGCTCCATAGATAGTGTGGCCCAAAGGTGGAAGCGGCCAGTCCATACGCAGCACACACACACNCNNNCNNNNNNACNNANAGCAGCATCAGCTGCTCTACGCCTAAACACACTCACGCGACGCTCGGTTGCAATAAGCCGAAAGGATTTCTGTCCAGCGGCAGTGATGGAGGAGGAGTGACACATTTCTTCGTCGCTTTCAACAGACGCTTTAGATAAAAACAGAAAGCCTTGCTTTTTAGTTTAGGGTTCGACTGTTTTGTATACATCGACCACGGCTCCAAGAGCTCTTTAGTTCTGCAAAGATGGGCACACTTATGACTCCATTCACTCGGTCTAACCGACCGTCAAAATATATACAACGTACACAAAACTTACTTGAAAACCTCTTCCGACATGCTGAGACCTGCCCCCTCCTCCTCTGAATGATGTCAGCTTAGGTGCCTCGTTTGCAGGCTCAAATAAATTCATTGCTAAAGTCACGTGCGCAAACTATTTTTCNTTATATAGCTCACAATTAGATCATTTGTAACGCTTCTGTTCTCAAGTAAGCAAAAAATAAAAAAAGAAAGTTCTGTACCCAGACCCAAAAGTAGTAAGGAATGCAATCAGGACCAGTGGGTGTGGCATAAATTCTTTGCCCCCTCCCTATGGAAACGATCTATAGCTGCATGGGATCATGAATCATAAGGTATTCCAAGTATGTAGCNGCGTGAATGACTCTCCTGATATCAGATCCTGCAGACAGACTGAGTTGTACTGTGATCAAGTNGCATGCTTTCATATAATGCATGACCTAATGTGGTGCTCAGCAGGTTCATAACAGAGGGGCTGTGAAGTCTGACACTTCATATCCTTAACAATGGCAAATGAAATGAAACATAGATGGTTATGATAAATTTCTGCACATACATGAATATAAAAATACCCACTAACCACTCCATGCAATAAAGAACAGAAGAGAGGGTACAAAACGAAGGAAGAAACACCTGACACTCACAACATGTCACATGACAACAGCTACGCCGGCAGCTGCCCTGCAAGTCTCCATAGAACTGACCAGATCCCAGCGTGTTGTTTACAGGGTCATACAGTTCTGCTGTTTTCAAGTACGAAGGACCATTGATCCCTCCAACAGCCACCATATGTGCACCAATCGCACATAGNCAGACTCCATCGCGAGCTGAGGTCATTTTGGCTGCTGGATACCATTTGCCACTGCGAACATCGAGAAACTCCACAGAGTCCAGCCGTGAGGTTCCATCGTTACCACCNGCAACAAAAATCTGCTGACCCAAGACTGCCACCCCGATCCCCGCACGAGGTTTCCCCAGCGGGGGCAATGTTCTCCATCGGTTGACATGAGGATCGTAGCACTCCACAACGTTGAGAAACGAGGTCCCATCGTTCCCGCCAAATGTGTACAACTTGTCACCAAAGACAGCTACTCCCGGGAAACTTCTCCGTGAGCTCATCTGAGCGACAGTGACCCAAACGTTCTTTTCAAGGTCGAACCTTTCAACCGTGTTGAGACAGGAGCGACCGTCGTAGCCCCCCGCGGCGTACATGGCGCCTCCCATGACCCCCACGGCCACGCCCGAGCGACACGTGCTCAGGGGGGCCACGTACGTCCAGAACTGAGTGGCCGGATCGAAGCACTCCACGGTGCTGAGGCGCAGAGAGCCGTCCGACCCGCCCACCGCGTACACTTTGCCGTCGAGGACCCCGACGCCCAACTGTTGCCTACGAACCATGAGGGGCGTGCCCTCGCTCCACTTGTTTTCCAGCAGCGAGAACGTCTCGACGGAGTGAGTGATGCTTTCGGACGACTCCTTCCCGCCAACCGCGTACACGACGCCCACCGTGGACTTGCGCGGAAAGAAGCACGGATTGGCCGAACGGATGTCTCTGTCGGCAGGCATTAGGTGGTAGCGTTTCGCGTCGTCAAGAAGGTCCCGACACTCGATGCTCTGCTTCACGATGGGATCACGGTCAACGTCGTTCATCAAACAGTCGACGGCGAGGAGCNNNNNNNNNACGTGCTTCAGCAGCTTTGGCAGCTGTTGCCCGCGCTCGCCCGGGTCCTGCCTGACCCAGCTCATGACAGCTGCGTACACGACCTCTTCCCTGGGTACGTTCAGGTCGTTGCTTGAGATCAGCGAGGCGATACCTTCGGCCGAACTGTGGAAGAACTCGTTGGTGTTGGTCACCTCGAGGAAGTGCCGGCGAGCAAAAGAATCGACCACCTCCGACAGCTGGGTGCAGCCATTGACTTCGGCAAAATTCTTAACACCCAGGCAGTTGCTCACGTCCAAGTGGTTTTTTAGAAATTCGCAGCACACTTCCTGGGCCTGTTTTATCTGCAGCAATGAGGCAGCCGCTAACGTGTCTTGTACATTTTCCACCGTGACTGTTAAGATCCCTGTATAGGCAAAGTCAATCAAACTGCTCAGAACATCTCCACTCATGCCCTTGATTTGAACTACGTCCTGACCGCTCTCCAAAAGTTCATGAGTAAACATGGCCAGAAAGTAGGCACTGCAGGCAGACAGGATGGCTCTGTGGGCATTTATCTCTCTTCCTTCGGCACTCAACGTCACGTCACATAGCTTTTTGTGACTTCGAAGTAAGTGTACCTTTTGAAGAACGCTCTTGCTGTAGTGAAGTGTTGCTTCGTCGGCATCGAAATGTGATGATGGAGCCGTAGAACGATGTGAGTTTTCCTGACGAGATAGAAGCATTGATGTCTCTTTTTCTGCCATTCTCTTTCATTTCATCGTGTCAAATTGACGAATTGAGTCCAAAGTATGAAAATAGATACATTACGTTTCAAATTTTAAAATTACTTGCGATGTATTTTTAGAACAGTGCCGGATGGAATTGATGCAGAAGTCACGTCTAGAGGGACAACCGAGAATCAAATCGCTTATCTCCTTGTCGCTATCGAATGGCAACACAAAAATAAACAGTTTCACGTTGGTTTCATATCACAAAAGATGTGCTTTTAGAGCAAAAAAAAAATGTTTGTGGCAGTTTCTCGTGCATGATCAGTCCCTTGGTCGTCGTCTTCTTCTTCTTCTTCCTTCGTCACGTATAACTAATTAAATTTCTTTCAGCCCTGGTGACCCATTTGAGATCAACAGATCTGATGTACGACTGTACTTAAAAAACAAACAAAAAAACAAAAAAAAACACGCATACATACGTCGCTATTGAATTTATTTGGGAACTGTATCTTACCAGTAGGCCCTAAATCTGACAACTGTAGGTGAAAGAATGCAGCCATTCCCTTTCCTTCCCTTAGTCTCGTCCCCATATTTTATTCCTTTCGCCGTAGCGGTAATGTGCTGCGGTATATTGGGTCTGGGGACGAGACACTACTACCACCACCACCACCACCACCACCACCACCACCACCNNNNNNNNNNNNNNNNNNNNNNNNNNNNNNNNNNNNNNNNNNNNNNNNNNNNNNNNNNNNNNNNNNNNNNNNNNNNNNNNNNNNNNNNNNNNNNNNNNNNNNNNNNNNNNNNNNNNNNNNNNNNNNNNNNNNNNNNNNNNNNNNNNNNNNNNNNNNNNNNNNNNNNNNNNNNNNNNNNNNNNNNNNNNNNNNNNNNNNNNNNNNNNNNNNNNNNNNNNNNNNNNNNNNNNNNNNNNNNNNNNNNNNNNNNNNNNNNNNNNNNNNNNNNNNNNNNNNNNNNNNNNNNNNNNNNNNNNNNNNNNNNNNNNNNNNNNNNNNNNNNNNNNNNNNNNNNNNNNNNNNNNNNNNNNNNNNNNNNNNNNNNNNNNNNNNNNNNNNNNNNNNNNNNNNNNNNNNNNNNNNNNNNNNNNNNNNNNNNNNNNNNNNNNNNNNNNNNNNNNNNNNNNNNNNNNNNNNNNNNNNNNNNNNNNNNNNNNNNNNNNNNNNNNNNNNNNNNNNNNNNNNNNNNNNNNNNNNNNNNNNNNNNNNNNNNNNNNNNNNNNNNNNNNNNNNNNNNNNNNNNNNNNNNNNNNNNNNNNNNNNNNNNNNNNNNNNNNNNNNNNNNNNNNNNNNNNNNNNNNNNNNNNNNNNNNNNNNNNNNNNNNNNNNNNNNNNNNNNNNNNNNNNNNNNNNNNNNNNNNNNNNNNNNNNNNNNNNNNNNNNNNNNNNNNNNNNNNNNNNNNNNNNNNNNNNNNNNNNNNNNNNNNNNNNNNNNNNNNNNNNNNNNNNNNNNNNNNNNNNNNNNNNNNNNNNNNNNNNNNNNNNNNNNNNNNNNNNNNNNNNNNNNNNNNNNNNNNNNNNNNNNNNNNNNNNNNNNNNNNNNNNNNNNNNNNNNNNNNNNNNNNNNNNNNNNNNNNNNNNNNNNNNNNNNNNNNNNNNNNNNNNNNNNNNNNNNNNNNNNNNNNNNNNNNNNNNNNNNNNNNNNNNNNNNNNNNNNNNNNNNNNNNNNNNNNNNNNNNNNNNNNNNNNNNNNNNNNNNNNNNNNNNNNNNNNNNNNNNNNNNNNNNNNNNNNNNNNNNNNNNNNNNNNNNNNNNNNNNNNNNNNNNNNNNNNNNNNNNNNNNNNNNNNNNNNNNNNNNNNNNNNNNNNNNNNNNNNNNNNNNNNNNNNNNNNNNNNNNNNNNNNNNNNNNNNNNNNNNNNNNNNNNNNNNNNNNNNNNNNNNNNNNNNNNNNNNNNNNNNNNNNNNNNNNNNNNNNNNNNNNNNNNNNNNNNNNNNNNNNNNNNNNNNNNNNNNNNNNNNNNNNNNNNNNNNNNNNNNNNNNNNNNNNNNNNNNNNNNNNNNNNNNNNNNNNNNNNNNNNNNNNNNNNNNNNNNNNNNNNNNNNNNNNNNNNNNNNNNNNNNNNNNNNNNNNNNNNNNNNNNNNNNNNNNNNNNNNNNNNNNNNNNNNNNNNNNNNNNNNNNNNNNNNNNNNNNNNNNNNNNNNNNNNNNNNNNNNNNNNNNNNNNNNNNNNNNNNNNNNNNNNNNNNNNNNNNNNNNNNNNNNNNNNNNNNNNNNNNNNNNNNNNNNNNNNNNNNNNNNNNNNNNNNNNNNNNNNNNNNNNNNNNNNNNNNNNNNNNNNNNNNNNNNNNNNNNNNNNNNNNNNNNNNNNNNNNNNNNNNNNNNNNNNNNNNNNNNNNNNNNNNNNNNNNNNNNNNNNNNNNNNNNNNNNNNNNNNNNNNNNNNNNNNNNNNNNNNNNNNNNNNNNNNNNNNNNNNNNNNNNNNNNNNNNNNNNNNNNNNNNNNNNNNNNNNNNNNNNNNNNNNNNNNNNNNNNNNNNNNNNNNNNNNNNNNNNNNNNNNNNNNNNNNNNNNNNNNNNNNNNNNNNNNNNNNNNNNNNNNNNNNNNNNNNNNNNNNNNNNNNNNNNNNNNNNNNNNNNNNNNNNNNNNNNNNNNNNNNNNNNNNNNNNNNNNNNNNNNNNNNNNNNNNNNNNNNNNNNNNNNNNNNNNNNNNNNNNNNNNNNNNNNNNNNNNNNNNNNNNNNNNNNNNNNNNNNNNNNNNNNNNNNNNNNNNNNNNNNNNNNNNNNNNNNNNNNNNNNNNNNNNNNNNNNNNNNNNNNNNNNNNNNNNNNNNNNNNNNNNNNNNNNNNNNNNNNNNNNNNNNNNNNNNNNNNNNNNNNNNNNNNNNNNNNNNNNNNNNNNNNNNNNNNNNNNNNNNNNNNNNNNNNNNNNNNNNNNNNNNNNNNNNNNNNNNNNNNNNNNNNNNNNNNNNNNNNNNNNNNNNNNNNNNNNNNNNNNNNNNNNNNNNNNNNNNNNNNNNNNNNNNNNNNNNNNNNNNNNNNNNNNNNNNNNNNNNNNNNNNNNNNNNNNNNNNNNNNNNNNNNNNNNNNNNNNNNNNNNNNNNNNNNNNNNNNNNNNNNNNNNNNNNNNNNNNNNNNNNNNNNNNNNNNNNNNNNNNNNNNNNNNNNNNNNNNNNNNNNNNNNNNNNNNNNNNNNNNNNNNNNNNNNNNNNNNNNNNNNNNNNNNNNNNNNNNNNNNNNNNNNNNNNNNNNNNNNNNNNNNNNNNNNNNNNNNNNNNNNNNNNATGTGTGTGTGTNTGTGTNTGTGTGTGTGTNTNTGTGTGTGTGAGGATATGTGTAAGAGGAGAGTATTAAATCATGAGTGCATAAAAGCATACAATGGGTTAAAACAATGAGCTGTTAACAAATCATCAAATATACCAGATCTATTTATACAGGAGTACCACAAATATAGAAATGTAAGCAAATTAAACTAGTTACCATGTTCAATAGTTCCTGGAGGACCTCCATAGTAGGCGGGGTAAGAGAAGCCATTAATTTTTTCAAAACACTGTATTTTATGTGCTGTCTGAATGATGCTTTTATAGTTGACGATCCTTTCAGTGCCACAGTGAGAGTCCTTATCAGCAGACATACAAGGGTGGTTCCCAGACCACCAAGGGGAACATTNTTTAGCTCCTTGAAGGACTCCACCAGCTTCTCAAAACAGCCCACTTCTGCAAAGAGCTCCTGTGTGGTTTGGATAACAATGGGTGCTCATGGCTTGGTCGTGTGGCTTTACCTTCAACTGGGTGGAGCCCTTGGGAGCATCCATCAATAGTGAAATAGAGTCTGGAGGAGAGAACCAAGCAATAAGGTGCAAGAAAGGAAGTGAATGATGAGGGAGGTGAACAAGGGGGAGAGGGAGGGAACATAGGGGGAGAGGGAGGGAACAAGGGGGAGAGGGAGGGAACAAGGGGGAGAGAGGGAACAAGGGGGAGAGAGGGAACAAGGGGGAGAGAGGGAACAAGGGGGAGAGGGAGGGAACAAGGGGAGAGGGAGGGAACAAGGGGGAGAGAGAGAACAAGGGAGAAAGGGAGGGAACAAGGNNNANANNNANNNACACANNNGAGAGAGAGAGGGAACAATGAGGGAGGGGAACAAGGGGGAGAGAGGAAGGAGAGCAAGGATTAAAATACTCACTGAGTAAAAGAACGAAGAATGTAGGAAGAGCTTGTTGCTGTTCAGGTTTTAATTCAGAAATGCTTTATGCCAATACAACAACCAAATATTAAGAACAAATCTTCACAAANGAAGGTGGTAGTCACCTGTATGGCAGGACACTGAACAGCTTAGATAAAGTGACTCCAGTGTCCACCTCAACCTGTTCCTATGGTAACACCACAACATAAATATATGAACATCCCTCTCTTCTCATGCCCCACCCACTTGCCTCTTTGGGCATGAAACAATGGAGGACGTGTAACACTTTAATGAACACCTTTATGGTCAGTGCCTGCNNNNNNNNNNNNNNNNNNNNNNNNNNNNNNNNNNNNNNNNNNNNNNNNNNNNNNNNNNNNNNNNNNNNNNNNNNNNNNNNNNNNNNNNNNNNNNNNNNNNNNNNNNNNNNNNNNNNNNNNNNNNNNNNNNNNNNNNNNNNNNNNNNNNNNNNNNNNNNNNNNNNNNNNNNNNNNNNNNNNNNNNNNNNNNNNNNNNNNNNNNNNNNNNNNNNNNNNNNNNNNNNNNNNNNNNNNNNNNNNNNNNNNNNNNNNNNNNNNNNNNNNNNNNNNNNNNNNNNNNNNNNNNNNNNNNNNNNNNNNNNNNNNNNNNNNNNNNNNNNNNNNNNNNNNNNNNNNNNNNNNNNNNNNNNNNNNNNNNNNNNNNNNNNNNNNNNNNNNNNNNNNNNNNNNNNNNNNNNNNNNNNNNNNNNNNNNNNNNNNNNNNNNNNNNNNNNNNNNNNNNNNNNNNNNNNNNNNNNNNNNNNNNNNNNNNNNNNNNNNNNNNNNNNNNNNNNNNNNNNNNNNNNNNNNNNNNNNNNNNNNNNNNNNNNNNNNNNNNNNNNNNNNNNNNNNNNNNNNNNNNNNNNNNNNNNNNNNNNNNNNNNNNNNNNNNNNNNNNNNNNNNNNNNNNNNNNNNNNNNNNNNNNNNNNNNNNNNNNNNNNNNNNNNNNNNNNNNNNNNNNNNNNNNNNNNNNNNNNNNNNNNNNNNNNNNNNNNNNNNNNNNNNNNNNNNNNNNNNNNNNNNNNNNNNNNNNNNNNNNNNNNNNNNNNNNNNNNNNNNNNNNNNNNNNNNNNNNNNNNNNNNNNNNNNNNNNNNNNNNNNNNNNNNNNNNNNNNNNNNNNNNNNNNNNNNNNNNNNNNNNNNNNNNNNNNNNNNNNNNNNNNNNNNNNNNNNNNNNNNNNNNNNNNNNNNNNNNNNNNNNNNNNNNNNNNNNNNNNNNNNNNNNNNNNNNNNNNNNNNNNNNNNNNNNNNNNNNNNNNNNNNNNNNNNNNNNNNNNNNNNNNNNNNNNNNNNNNNNNNNNNNNNNNNNNNNNNNNNNNNNNNNNNNNNNNNNNNNNNNNNNNNNNNNNNNNNNNNNNNNNNNNNNNNNNNNNNNNNNNNNNNNNNNNNNNNNNNNNNNNNNNNNNNNNNNNNNNNNNNNNNNNNNNNNNNNNNNNNNNNNNNNNNNNNNNNNNNNNNNNNNNNNNNNNNNNNNNNNNNNNNNNNNNNNNNNNNNNNNNNNNNNNNNNNNNNNNNNNNNNNNNNNNNNNNNNNNNNNNNNNNNNNNNNNNNNNNNNNNNNNNNNNNNNNNNNNNNNNNNNNNNNNNNNNNNNNNNNNNNNNNNNNNNNNNNNNNNNNNNNNNNNNNNNNNNNNNNNNNNNNNNNNNNNNNNNNNNNNNNNNNNNNNNNNNNNNNNNNNNNNNNNNNNNNNNNNNNNNNNNNNNNNNNNNNNNNNNNNNNNNNNNNNNNNNNNNNNNNNNNNNNNNNNNNNNNNNNNNNNNNNNNNNNNNNNNNNNNNNNNNNNNNNNNNNNNNNNNNNNNNNNNNNNNNNNNNNNNNNNNNNNNNNNNNNNNNNNNNNNNNNNNNNNNNNNNNNNNNNNNNNNNNNNNNNNNNNNNNNNNNNNNNNNNNNNNNNNNNNNNNNNNNNNNNNNNNNNNNNNNNNNNNNNNNNNNNNNNNNNNNNNNNNNNNNNNNNNNNNNNNNNNNNNNNNNNNNNNNNNNNNNNNNNNNNNNNNNNNNNNNNNNNNNNNNNNNNNNNNNNNNNNNNNNNNNNNNNNNNNNNNNNNNNNNNNNNNNNNNNNNNNNNNNNNNNNNNNNNNNNNNNNNNNNNNNNNNNNNNNNNNNNNNNNNNNNNNNNNNNNNNNNNNNNNNNNNNNNNNNNNNNNNNNNNNNNNNNNNNNNNNNNNNNNNNNNNNNNNNNNNNNNNNNNNNNNNNNNNNNNNNNNNNNNNNNNNNNNNNNNNNNNNNNNNNNNNNNNNNNNNNNNNNNNNNNNNNNNNNNNNNNNNNNNNNNNNNNNNNNNNNNNNNNNNNNNNNNNNNNNNNNNNNNNNNNNNNNNNNNNNNNNNNNGAGGGAGGAGGGAGGGTTAGAGAACAAGGTGAAGAAACAAGAGGAAGACAAGTGGTGTCAGTGAAGGCTCATTGGAGTTAAACACATATGGAGGCTCATTGGAGTTAAACACATATGGAGGCTCATTGGAGTTAAACACATATGGAGGCTCATTGGAGTTAAACACANATGGAAGCTCAGTGGGGTTAAACACATACAAAGGCTCATTGGGGTTAAAAATTACCAGTTCCAAATTCTCGCCACACTTCACTGGTCGAAGGGAAACCAAAATGTCCTTGAGCTCAGTACCCATGATCGAGTGGGTGCCCAGTCCTTCAATCAATCTCAACAAATTGTCTGCAACCCAAAGAGAGTTGTTACACCCCACAGGGGGGGTGGGGCACACAGGGNNGNGNGGCNNACTAGGGCCGGGTGGCTAACTANNNNNNNNNNNNNNNNNNNNNNNNNNNNNNNNNNNNNNNNNNNNNNNNNNNNNNNNNNNNNNNNNNNNNNNNNNNNNNNNNNNNNNNNNNNNNNNNNNNNNNNNNNNNNNNNNNNNNNNNNNNNNNNNNNNNNNNNNNNNNNNNNNNNNNNNNNNNNNNNNNNNNNNNNNNNNNNNNNNNNNNNNNNNNNNNNNNNNNNNNNNNNNNNNNNNNNNNNNNNNNNNNNNNNNNNNNNNNNNNNNNNNNNNNNNNNNNNNNNNNNNNNNNNNNNNNNNNNNNNNNNNNNNNNNNNNNNNNNNNNNNNNNNNNNNNNNNNNNNNNNNNNNNNNNNNNNNNNNNNNNNNNNNNNNNNNNNNNNNNNNNNNNNNNNNNNNNNNNNNNNNNNNNNNNNNNNNNNNNNNNNNNNNNNNNNNNNNNNNNNNNNNNNNNNNNNNNNNNNNNNNNNNNNNNNNNNNNNNNNNNNNNNNNNNNNNNNNNNNNNNNNNNNNNNNNNNNNNNNNNNNNNNNNNNNNNNNNNNNNNNNNNNNNNNNNNNNNNNNNNNNNNNNNNNNNNNNNNNNNNNNNNNNNNNNNNNNNNNNNNNNNNNNNNNNNNNNNNNNNNNNNNNNNNNNNNNNNNNNNNNNNNNNNNNNNNNNNNNNNNNNNNNNNNNNNNNNNNNNNNNNNNNNNNNNNNNNNNNNNNNNNNNNNNNNNNNNNNNNNNNNNNNNNNNNNNNNNNNNNNNNNNNNNNNNNNNNNNNNNNNNNNNNNNNNNNNNNNNNNNNNNNNNNNNNNNNNNNNNNNNNNNNNNNNNNNNNNNNNNNNNNNNNNNNNNNNNNNNNNNNNNNNNNNNNNNNNNNNNNNNNNNNNNNNNNNNNNNNNNNNNNNNNNNNNNNNNNNNNNNNNNNNNNNNNNNNNNNNNNNNNNNNNNNNNNNNNNNNNNNNNNNNNNNNNNNNNNNNNNNNNNNNNNNNNNNNNNNNNNNNNNNNNNNNNNNNNNNNNNNNNNNNNTCAAAAGCATTGCGTTCTTTCGTGGACACCCGGTATACAGCACAACAGAGTAGCATTATTAAACATTAACTATTGAATGCCACTGCTCCTTATTTGCCAAGAGGTAATGAACACAAGGACACACCCATAATGAGTAACAGCATTTCTATGACATCATGACGTCAGTAATATCAGACAAGCCCTTTAACTCAATCCTGATTGCACGTAACAGTTTTAAGGCAGAACAGCACTGCCCTTCTGTCCAGTCAAACAGCCCTGTTTCCGTAGGACACAGCACAGAGACACTTACCAGTATTCCAAAATTCACTAGCTTTGGGTCGAGGTGGCTCAATGATGGAATATGCCTCCCTCTGTCTCAAGGGCAGTCCGGCCTGCTCCAGTATGGGGAAGAACACCTCCACTCCACCCAGGGAACTCAAAACCAACTGAAATGGGGGAGGTGGCGTCCATTGGCAGGGCTATGAGNTNTCCCCACCTTTCCTCCTCNCCCTCCCTCCCTTATCGATAATGTACAGGCTTGCCTTGACGTCTCTGATCACATAGACGTCTCCATNCAGTACTGCATCCACAGTCCCNTGCACAGCATGTGACGGAGAGAGGTCCACACACATCTTATCTTTGCATGCCTGTGCACGATGGGACAAGCTAGCTACAAGAGNACCACACTCACTGTTGCCATGACTACCTTCGGATGATAGTAAAAGATCAGCTTAGTCTTCCACTCGTCTGCCTTCGTTTGGAAGATNGACAAATCATTAGGTCCTAAGAATAGCAATTATAATTGATGCCCACCACTTCCCTCGTACCCAGTCCTTACCACGANAGTGGAGAAGCTGGACAGTGGTGTGGTTGAGTGNGTCAGAGAAGAGACACACAGGNCCAATGCAGCCCCCAAAGTTGGTCACAGAGCCAAAGGACGTGATACACTCATCCAGCCGCAAGGGGCTGATGATGTCATGAGGGGTGGGGCTCTCANGGGGAGAGGTGGAGCCCTCTGCGGAGAGCTGATTGTCTGTGCTGTCATGGCTCCAGCCAATCCTGCACTCTGTAAATGTCTGCATGGAGGAAGACATGAGGGAGGAGGAAGGCATGGGGAGGAGGAAGGCATGGGAGGAGGAAGACATGGGGAGGAGGAAGACATGGGGAGGAGGAAGACATGGGGAGGAGGAAGACATGGGGAGGAGGAAGGCGTGGGGAAGAGGAAGGCATGGGGAGGAGGAAGACATGGGGGAGGAGGAAGACATGGGGAGGAGGTNNNNCANTGGGGAGGAGGAAGNCATGGGGGAGGAGGAAGACATGGGGGAGGAGGAAGACATGGGGGGAGAAGGAGAACATGGAGGGAGGAGGAACGGGGGAGGAATGCAAAGGAAGGGATGATAATGGAAGTGTGATAAACCCAGTGTCTACCACTCACATTGGCAAGTGGTACAGCTCTGATCCCATCTGTTGCGGAGATTAGCCCATCCACGAGCACCTGTATGGTGACCTTGTCGCTGTGCTTCTTGCTACTGCTGTACACCACACACACTGAATGCTGGGCACCAGGTAGAGGTAGAGCANAACATAAATACACTCCACCCCTCCCCAATGACNAAAAGAACGCGTACCCATTTCTGTGGAGTGAGTGGCTCTGTGAGAGAAACAGTCTTAAGCCCCCTCTTCTTGGAGTGAACTGCCACCACCAGGACCAGGTCTTTGGTAAAGAACGCTTCAAACCCTGTACTACCAGCATAGAAACTGAAAGTAAGAGACCATTGGGTTATGATATGTAGCCCCTCCTTCTGCTCCACCCACCTGTAAAGCATTCTTCTCATGTTCCTTTGGTCCCCATCTGCACAACTTCGATCCAAACTCACCCAGCAGTGGAAGGAGAAGGCATTTTCTTTGGGCCAGCGATGTAGCTCTGAGATGGATATTTGCTGTANAGAGGATAAGATGATAAGGATGACACTAACACAGAGATAAGATGACAGAGATGACACTAACACAGAGATAAGATAAGGATGACACTAACACAGAGATAAGATAAGGATGACACTAANNNNNNNNNNNNNNNNNNNNNNNNNNNNNNNNNNNNNNNNNNNNNNNNNNNNNNNNNNNNNNNNNNNNNNNNNNNNNNNNNNNNNNNNNNNNNNNNNNNNNNNNNNNNNNNNNNNNNNNNNNNNNNNNNNNNNNNNNNNNNNNNNNNNNNNNNNNNNNNNNNNNNNNNNNNNNNNNNNNNNNNNNNNNNNNNNNNNNNNNNNNNNNNNNNNNNNNNNNNNNNNNNNNNNNNNNNNNNNNNNNNNNNNNNNNNNNNNNNNNNNNNNNNNNNNNNNNNNNNNNNNNNNNNNNNNNNNNNNNNNNNNNNNNNNNNNNNNNNNNNNNNNNNNNNNNNNNNNNNNNNNNNNNNNNNNNNNNNNNNNNNNNNNNNNNNNNNNNNNNNNNNNNNNNNNNNNNNNNNNNNNNNNNNNNNNNNNNNNNNNNNNNNNNNNNNNNNNNNNNNNNNNNNNNNNNNNNNNNNNNNNNNNNNNNNNNNNNNNNNNNNNNNNNNNNNNNNNNNNNNNNNNNNNNNNNNNNNNNNNNNNNNNNNNNNNNNNNNNNNNNNNNNNNNNNNNNNNNNNNNNNNNNNNNNNNNNNNNNNNNNNNNNNNNNNNNNNNNNNNNNNNNNNNNNNNNNNNNNNNNNNNNNNNNNNNNNNNNNNNNNNNNNNNNNNNNNNNNNNNNNNNNNNNNNNNNNNNNNNNNNNNNNNNNNNNNNNNNNNNNNNNNNNNNNNNNNNNNNNNNNNNNNNNNNNNNNNNNNNNNNNNNNNNNNNNNNNNNNNNNNNNNNNNNNNNNNNNNNNNNNNNNNNNNNNNNNNNNNNNNNNNNNNNNNNNNNNNNNNNNNNNNNNNNNNNNNNNNNNNNNNNNNNNNNNNNNNNNNNNNNNNNNNNNNNNNNNNNNNNNNNNNNNNNNNNNNNNNNNNNNNNNNNNNNNNNNNNNNNNNNNNNNNNNNNNNNNNNNNNNNNNNNNNNNNNNNNNNNNNNNNNNNNNNNNNNNNNNNNNNNNNNNNNNNNNNNNNNNNNNNNNNNNNNNNNNNNNNNNNNNNNNNNNNNNNNNNNNNNNNNNNNNNNNNNNNNNNNNNNNNNNNNNNNNNNNNNNNNNNNNNNNNNNNNNNNNNNNNNNNNNNNNNNNNNNNNNNNNNNNNNNNNNNNNNNNNNNNNNNNNNNNNNNNNNNNNNNNNNNNNNNNNNNNNNNNNNNNNNNNNNNNNNNNNNNNNNNNNNNNNNNNNNNNNNNNNNNNNNNNNNNNNNNNNNNNNNNNNNNNNNNNNNNNNNNNNNNNNNNNNNNNNNNNNNNNNNNNNNNNNNNNNNNNNNNNNNNNNNNNNNNNNNNNNNNNNNNNNNNNNNNNNNNNNNNNNNNNNNNNNNNNNNNNNNNNNNNNNNNNNNNNNNNNNNNNNNNNNNNNNNNNNNNNNNNNNNNNNNNNNNNNNNNNNNNNNNNNNNNNNNNNNNNNNNNNNNNNNNNNNNNNNNNNNNNNNNNNNNNNNNNNNNNNNNNNNNNNNNNNNNNNNNNNNNNNNNNNNNNNNNNNNNNNNNNNNNNNNNNNNNNNNNNNNNNNNNNNNNNNNNNNNNNNNNNNNNNNNNNNNNNNNNNNNNNNNNNNNNNNNNNNNNNNNNNNNNNNNNNNNNNNNNNNNNNNNNNNNNNNNNNNNNNNNNNNNNNNNNNNNNNNNNNNNNNNNNNNNNNNNNNNNNNNNNNNNNNNNNNNNNNNNNNNNNNNNNNNNNNNNNNNNNNNNNNNNNNNNNNNNNNNNNNNNNNNNNNNNNNNNNNNNNNNNNNNNNNNNNNNNNNNNNNNNNNNNNNNNNNNNNNNNNNNNNNNNNNNNNNNNNNNNNNNNNNNNNNNNNNNNNNNNNNNNNNNNNNNNNNNNNNNNNNNNNNNNNNNNNNNNNNNNNNNNNNNNNNNNNNNNNNNNNNNNNNNNNNNNNNNNNNNNNNNNNNNNNNNNNNNNNNNNNNNNNNNNNNNNNNNNNNNNNNNNNNNNNNNNNNNNNNNNNNNNNNNNNNNNNNNNNNNNNNNNNNNNNNNNNNNNNNNNNNNNNNNNNNNNNNNNNNNNNNNNNNNNNNNNNNNNNNNNNNNNNNNNNNNNNNNNNNNNNNNNNNNNNNNNNNNNNNNNNNNNNNNNNNNNNNNNNNNNNNNNNNNNNNNNNNNNNNNNNNNNNNNNNNNNNNNNNNNNNNNNNNNNNNNNNNNNNNNNNNNNNNNNNNNNNNNNNNNNNNNNNNNNNNNNNNNNNNNNNNNNNNNNNNNNNNNNNNNNNNNNNNNNNNNNNNNNNNNNNNNNNNNNNNNNNNNNNNNNNNNNNNNNNNNNNNNNNNNNNNNNNNNNNNNNNNNNNNNNNNNNNNNNNNNNNNNNNNNNNNNNNNNNNNNNNNNNNNNNNNNNNNNNNNNNNNNNNNNNNNNNNNNNNNNNNNNNNNNNNNNNNNNNNNNNNNNNNNNNNNNNNNNNNNNNNNNNNNNNNNNNNNNNNNNNNNNNNNNNNNNNNNNNNNNNNNNNNNNNNNNNNNNNNNNNNNNNNNNNNNNNNNNNNNNNNNNNNNNNNNNNNNNNNNNNNNNNNNNNNNNNNNNNNNNNNNNNNNNNNNNNNNNNNNNNNNNNNNNNNNNNNNNNNNNNNNNNNNNNNNNNNNNNNNNNNNNNNNNNNNNNNNNNNNNNNNNNNNNNNNNNNNNNNNNNNNNNNNNNNNNNNNNNNNNNNNNNNNNNNNNNNNNNNNNNNNNNNNNNNNNNNNNNNNNNNNNNNNNNNNNNNNNNNNNNNNNNNNNNNNNNNNNNNNNNNNNNNNNNNNNNNNNNNNNNNNNNNNNNNNNCACACACACACACACACACACATACACACACACACACTTTGAAANAGTCANATANAGAGGGAGTGGTGTGTGGCACACCTTCTGAAGGAGGGCTCCAATTGTAGCTGGTCCCCCAACGACAAAGAAGACCTGCTCATTGATTGATGTCTTGGTCAACATGGAGGTCATCAGTCGAATGAAGTATGTGACTGCCTCATGTTCTGGAAGTGTGCAAATAACAGGTGTGAACTCATGCTTGAGATGGCACTGCACGTAATGTCTGGAGAAGTCTTGATCAACAGACACAACAGTGGATTTCACCTCTAACGCTTACCATTATTCCAAGGTTCAAAGTTCAATGTGGTTCTGGGCACATGATTTATATTTATGGAACATATTTCCCTCTGTNTCAAAGGCAGTCCNGCCTGCTCCAGTATGGGGAAGAACACCTCCACTCCACCCAGGGAACTCAAAACCAACTGAAATGGGGGAGGTGGTGTCAATGAGATACTACTGTCACTCCCTTCCCTCACTCCCCCTTGGGATCAACTGGCTTGCCTGAACATCTCTGGTCACATAGACTTCACCAACCAGTACGCCCTCCATGGCTCCTTCCACTGTGTGTGATGGAGAGAGGTCCACACAGATGTTGNNCTTACAGGCCTGAAGGGAAGAAGAGACCATCAAAGATGGTGCCTAGAACAGAGGGGGATTTCCCCACCTTTGGGTGGAAGAACAGCACCAGTTTACTAATGAGTGCCTCCGTGTCAGGATACTTTGGTTGGAAAAGAGATAGGTTATTGGGTCCTGGCATATGTGGAAAAAGAAACAAAACAGGTCAATAAGCAGCATGTCTNCNNNNANNGATNNANTGGTNNCNNCTCCTACTTCCCCACCCANNNNNNNNNNNNNNNNNNNNNNNNNNNNNNNNNNNNNNNNNNNNNNNNNCCTCCTACTTCCCCACCCACTGTCCACTGTAACCCCTCCTACTTCCCCACCCACTGTCCACAGTAACCCCTCCTACTTCCCCACCCACTGTCCACTGTAACCCCTCCTACTTCCCCACCCACTGTCCACTGTAACCCTCCTACTTCCCCATTACATACACACCATTTCGATACAAAAGCTGTACAGCTTGCAGTTGCAACACTTCTGAGAAGATGTAGACAGAGCTGATCCTGCCAAACAGGCTAATGGCAGGACCAAAGGCTACGGGACTGTCCGCCATTGCCTCAAGCGATTCTGAAAGATCTGCCTCACGTTGGCAAAACCCAATATTGCAATGCTTGTAATCCTGGGGAGGAGGCAGTGTGCCAAGTGAGGATGTGGGGAGGAGAGGGAGAAGGTTGAGAGGAGAGTGAGGAGGTTGAGAGGAGAGGGAGAAGGTTGAGAGGAGAGGGAGGAGGTTGAGAGGAGAGGGAGGAGGTTGAGAGGAGAGGGAGGAGGTTGAGAGGAGAGGGAGGAGGTTGAGAGGAGAGGGAGGAGGTTGAGAGGAGAGGGAGGAAATTAAGAGGAGAGGNAGGAGGTTGAGAGGAGAGGGAGGAGGTTGAGAGNAGAGGGAGGAGGNNGNGTGGTTGAAAGGATTGGGAGGAGGAAGTGCAGGGCACACCAAGTAAAAGAGAGAAATCTGCATGCATGTGACCTTTACTTACGGCAGAAAGGTCTGGTGCTCTGAGATCAGTTGAAAAGGCTTTCTGGATTTCATCGATGTACACAGCAGTCTCATTTTTCCCACCCCAGGGTTTTTTAGAAGCGCTATGCGTCACACATACTGCATGCTAATGGAAGCTCACTGCATTTGATGCTATTGCAGCATGCCAGAACATCACCAGCGATGTCTTACCCATTTCTGAGGACTGAGTGGCTCTTTCACTGAGATGGTTTGAAACCCTCTCTTCTTGGAGTGAACTGCCACCACCAGGACCAGGTCTTTGGTAAAGAACGCTTCAAACCCTGTACTACCAGCATAGAAACTGAAAGAAATAGACCATTGGGTTATGATACGTAGCCCCTCCTTCTGCTCCACCCACCTGTAAAGCATTCTTCTCATGTTCCTCTGGTCCCCATCTGCACAACTTTGATCCAAACTCACCCAGCAGTGGAAGGAGAAGNCATACTGGTTTGGTTGCACGACTTCTGGAACAGCTATATGTATATGCTGTGCATNAAGTATTAGAGAGGAGGGAGAGGAGGGAGAGGAGGGAGAGGAGGGAGAGGAGGGAGAGGGAGGGGAGAGGGGAGGAGGAGGGAGAAGGGAGAGGAGGGGGAAGGGAGAGGAGGGAGGAGGGAGAAGAGGGAGAAGAGGAAGAAGAGGGGGAGGAGGGAGAGGAGGGGGAGGAGGAGGGAGAAGGGAGAGGAGGGGGAGAGGGANNNNNNNNNNNNNNNNNNNNNNNNNNNNNNNNNNNNNNNNNNNNNNNNNNNNNNNNNNNNNNNNNNNNNNNNNNNNNNNNNNNNNNNNNNNNNNNNNNNNNNNNNNNNNNNNNNNNNNNNNNNNNNNNNNNNNNNNNNNNNNNNNNNNNNNNNNNNNNNNNNNNNNNNNNNNNNNNNNNNNNNNNNNNNNNNNNNNNNNNNNNNNNNNNNNNNNNNNNNNNNNNNNNNNNNNNNNNNNNNNNNNNNNNNNNNNNNNNNNNNNNNNNNNNNNNNNNNNNNNNNNNNNNNNNNNNNNNNNNNNNNNNNNNNNNNNNNNNNNNNNNNNNNNNNNNNNNNNNNNNNNNNNNNNNNNNNNNNNNNNNNNNNNNNNNNNNNNNNNNNNNNNNNNNNNNNNNNNNNNNNNNNNNNNNNNNNNNNNNNNNNNNNNNNNNNNNNNNNNNNNNNNNNNNNNNNNNNNNNNNNNNNNNNNNNNNNNNNNNNNNNNNNNNNNNNNNNNNNNNNNNNNNNNNNNNNNNNNNNNNNNNNNNNNNNNNNNNNNNNNNNNNNNNNNNNNNNNNNNNNNNNNNNNNNNNNNNNNNNNNNNNNNNNNNNNNNNNNNNNNNNNNNNNNNNNNNNNNNNNNNNNNNNNNNNNNNNNNNNNNNNNNNNNNNNNNNNNNNNNNNNNNNNNNNNNNNNNNNNNNNNNNNNNNNNNNNNNNNNNNNNNNNNNNNNNNNNNNNNNNNNNNNNNNNNNNNNNNNNNNNNNNNNNNNNNNNNNNNNNNNNNNNNNNNNNNNNNNNNNNNNNNNNNNNNNNNNNNNNNNNNNNNNNNNNNNNNNNAGCCGGACGAGGTCAATCTCCCGACAACAGAGCATATTCTGATGGGTGGGTGTGGTTAGTAGGCCCAGAAGGTACTGCAACATCTCATCTCTCTGAGGGAGGAGAGGGAGGGAGGAGAGGGAGAAGTACTCACCAGAATTTCATCGTTCACTGCACACAGAAACCCTAGAATAGCATCCAATTCGTCACTGAAAATGAATTCTGCAAAGTACAGATCAACAACTGTGGAGAAACAGCAACAGAATCAGTCGATTGTCCCACCATCTGGTACAATGTACACAACACACCATCACATCTGGTACACAACACACAATCACATCTGGTACACAACACATCATCACAGCTGGTACACAGCACACCATCACATCAAGGCAACACAGCTTGGCATAACTACCGGAGATGACTCACTGGACATTAAGGACTTCCTTATATGGACTGTACTCAGCTTTGGATTAATAGCAGATGATGGACTACAAGGGAGGTCATACATTAATTGGAAGACTAGTTAGTAAATGCCCTACTTGTAATACTCTCTGATGATGTCTAAAACAAACTGAACTCCAAAATCTCTCCTACAGTCGTCCAGCTTCAGACTGACTAGGGTTGACAAGTACTTGCTGTGACCTGGGAATGGAAACACAAGAATGGGACAAGAACGGCAAGCATTGGAATGTGACATAAGTGATTGTACAGGTCACTGACTCAGTTGCACAGAGTAGTCTGCTCTGCTCCATATCCTGTAGTTGAACAGCAGTGACGAATAGAGATCAGTCTGCTGCTCTGGCTGGTCTGGACTGAGCTTCTCAACAAATTTTTCGATCGCACTGTGAGCTGCCACAGTCAACAGAGAAGGAGGGACCTGGGCACACACACATATACATATACATACACACATCACACACACACACACACACACACACACANACACACAACAAATTAACTGAATACACATGTTGCTCACCTTACATAGAACATGTAGTCAGGGGCATAAGCATCATTGGGATCAGGGTCCAGCTGGGTCATCAGATCAAATGCTGTCTTCTGGTAAAGCAGAGGAAACCTGTTGTGCATCTCCAACCCTACAGGATGGGATACAGGCCTATCAGNNNNNNNNNNNNNNNNNNNNNNNNNNNNNNNNNNNNNNNNNNNNNNNNNNNNNNNNNNNNNNNNNNNNNNNNNNNNNNNNNNNNNNNNNNNNNNNNNNNNNNNNNNNNNNNNNNNNNNNNNNNNNNNNNNNNNNNNNNNNNNNNNNNNNNNNNNNNNNNNNNNNNNNNNNNNNNNNNNNNNNNNNNNNNNNNNNNNNNNNNNNNNNNNNNNNNNNNNNNNNNNNNNNNNNNNNNNNNNNNNNNNNNNNNNNNNNNNNNNNNNNNNNNNNNNNNNNNNNNNNNNNNNNNNNNNNNNNNNNNNNNNNNNNNNNNNNNNNNNNNNNNNNNNNNNNNNNNNNNNNNNNNNNNNNNNNNNNNNNNNNNNNNNNNNNNNNNNNNNNNNNNNNNNNNNNNNNNNNNNNNNNNNNNNNNNNNNNNNNNNNNNNNNNNNNNNNNNNNNNNNNNNNNNNNNNNNNNNNNNNNNNNNNNNNNNNNNNNNNNNNNNNNNNNNNNNNNNNNNNNNNNNNNNNNNNNNNNNNNNNNNNNNNNNNNNNNNNNNNNNNNNNNNNNNNNNNNNNNNNNNNNNNNNNNNNNNNNNNNNNNNNNNNNNNNNNNNNNNNNNNNNNNNNNNNNNNNNNNNNNNNNNNNNNNNNNNNNNNNNNNNNNNNNNNNNNNNNNNNNNNNNNNNNNNNNNNNNNNNNNNNNNNNNNNNNNNNNNNNNNNNNNNNNNNNNNNNNNNNNNNNNNNNNNNNNNNNNNNNNNNNNNNNNNNNNNNNNNNNNNNNNNNNNNNNNNNNNNNNNNNNNNNNNNNNNNNNNNNNNNNNNNNNNNNNNNNNNNNNNNNNNNNNNNNNNNNNNNNNNNNNNNNNNNNNNNNNNNNNNNNNNNNNNNNNNNNNNNNNNNNNNNNNNGGGACATGTGTACCATCATGTACAACATCATGGACATGTGTACCATCATGTACAACATCATGGGACATGTGTACCATCATGTACAACATCATGGGACATGTGTACCATCATGTACAACATCATGGGACATGTGTACCATCATGTACAACATACCGGGACATTTTTTCAATACAGCTCACCTGTTCTTCCATGCTCCACACTCCCCTATAAGGAACCTTCGTGTGCCACTCCATCTCTGTAAAGTTGCTGATAACTCCTGCCTGTATTGATTGTCTAATGTCTTTTGTCGAGACAGCTCCAATTCCCGCTGTGAGTAAAACTGCTGCGATACTGTTATCTGATATTTTTCAAGATCTGAGAACACTATTTCTTTTCTTTTTGCTTCTGCTTTCAAGCAGTCTTGCTTGCATTGCCTAGTATGGTCCGATAAGACCTTCAACCATTTGTAAACTACCTCAGAGTATTGCATTTGTATAGCCTGAAGCTGTGTTAGAGTGTTGAGAGATGGGAGAGGGCACTCACCCGACAACCTGCTTACCTCACCCAGCCACGTATGCCACTCCTCCCTCTTGGAATATTCAACCAGCTCGATAGTCTCATTGGTGAGATCTGGTAGATAAGAAGGCAAAGGAACCAGCTCCTTATTCCGATACAATTGAAAAAGGTGCTGAAGTAGAGTAGCCCTTGTTGTAGGAGGTTGATCTGAACACACCCAGTATAGAAAATAGGAAGCAAATAGATTAGCATGTTGGAAGAAAAAGACGAGAGGGTAAGAAGGGATAAAGGGAGCAGAACAAAGGAGAAGGAACAGACAGAATCATTCACTGACCACTGCTGAACTGGGTTGCAATGCACCTGAGCACANANACTGNNTCNNGTGCTTCCAGGAATTGAAGTTCTTGCAGCTTACAACCATACTGACAATACTGTGGTACAAAGAGGCCCATAAGAAGGAGAGGGTGAGTAAGGAGAGGGGAAAGGAAGGGGAGGAGAGACAGAATGCATAAAGAGGAGACAGAGCATAGAGTACTAGTAAAATTGTACCACACTGAGATAGCACTGAGAAAACAATATCCACTCACTAAACACCCTCTTCCTCTTACTACTACCCAATCACCTCTGCATCATCAGAGCAAGCACTGGCCAATAAGATTCGCAGCACCGACTGCATCAGGGCTGGTTCTATGGGTCCTTCCAGCACATGAGCTGCTCTCAACATATTCCAGTATTGTTCCAGCAGCTGAAAGGAAAACATTCAAGCCATTGAGGTGTTAAGTTACACTAACATACCTGCATAGAGCAGTGTTCTTTGTTGAGCACCAAGACCCCATTATGAGAGAACTTTTGAAGAATCACTTCTAAATCTGTTGAATGTGAAAGATATCAGGTGTGCAGTAGGGTGGTGTGGTGTGTGCAAGAGTAGTCAGGTGTGCAGTAGGGTGGTGTGGTGTGTGCAAGATATCAGGTGTACAGTAGGATGTGTGGAAGATGTGTACCTTCTACAACATCAAACGAGGGATAGCTTGAGATGATGAAAGCTTCAAGCAGTTGGGATAATTGAAGGATGACAGAGGATAACATGTTTTCAGGAGACCAGCTTGGAACTTGGAAAACAAGGGACCATAGCAGTGATGAACCAATGATGGCCTCCAGCTCCCATGCCTTCTGGCCCAAATCTTTTCCCAATTCCTTCTCCTCTCTCTTCAGCCCCCTTCCAGCAACCACTCTCCTTACCTGAGGAACACTCCAAGGCAACAGCATTCATCAACAGCAAGAAGATTCTAACAAGCAAAGCAACATGAATGAAATATATATAACCATCTCCAGTCCAGTATAAAAAGACATCTGGACCACCCAACAACACAACAAGACATCTGGACCACCCAATAACACAACAAGACATCTGGACTGTACCTTTGTTTGATCAGGTGGACTGGCACTAACAGTCTCTGCTCTGTTGCAAAGAGGTCCAACGCTGCCATAAACTGACCACATACCTATGTGGGAGGGGCCAAAGTGAGGACACACCCACAGACCACACCTACTTTTCTAGTTTGTCCTTCCGACTGCTCTGAGATGATGCAATGCCACAGAATGTAACCAATGGTCTTCATGACAACATCATACGTTTCCGAAGTGGCTGATTCAGCTTCTGTCTGGTTGTCCACGTCCGATTTGCAGGGGGTCAGTAGCCATAGCAACAACCTCTCCCACCCTTTGGACTCTGCCAGCTTGGCCACGTGGCTGGAGTTAAGATGGAGGATGGAGATGAAATCCTGACAAATTGTGTGTCGAATGGACATGGAGGCTGTTCGAAGCAGTCTTAGAGTGGCCAGGTAGACATCAAAGTTGGAGATGCCGCCTGAAACAGAATACGCACATTGCACATGCTGTCTGGTCGGTGTGGTTATCACATTTATGCACATTGCACACATGTTTGTGTTGCCAGCCGTACATGCTCCATTGTTTGTATTACATGTCTACGGGCATTGTATGCTGCACACAGGCACTGTACGTATACTTCACTGCACACGGCACTGTATGCTGCACATGGGCACTGTACATATACTTCACTGCACATGGGCACTGTATNCTGCACATGGGCACTGTATGCTGCACATNGGCACTGTACATATACTTCACTGCACACAGGCACTGTACGTAGCACATACCTGTTTGGTTGTCACTCTTCTTGACCATGCAGAGGTCTAGAAGGGCCAAAATGATGGACGAGGGCAGACACTGCTCATTCTGACACATGGCAGCAGCAAAGGCCCCCGAGTGGAGGGTGTTGTACACCACACCACTCTCAACGCTCCCTTGCTTTAAATTGAGCTGGGTCAGAATCTGCAAGCGTGGGGCGTGAGCACATGGTTTCACCACGCCCCCTCAACAGACCTACCCTCCACCACGCCCCCTCAACACACCCCCTCAACAGACCTACCCTCCACNACAACCCCCTCAAAAGACCTACCCTCAGAGTTGTGAGTCGCACGCCAAAGTCTTGCGTCTGAATTAAAANGAGNAAGACCACACCCTTTTCCCAGAGGGCTGTTGAGAGCATATTTTGATGNGTGGGTGTGGTTAGTAGGCCCAGAAGGAATCGAAGAACCTCATCTAACTGAATAGAGGAGTGGTTTTTTAAAGGAAGAACACACACACACATACATGCACACAACACATACACGCACGCACGCACGCANNNNNNNNNNNNNNNNNNNNNNNNNNNNNNNNNNNNNNNNNNNNNNNNNNNNNNNNNNNNNNNNNNNNNNNNNNNNNNNNNNNNNNNNNNNNNNNNNNNNNNNNNNNNNNNNNNNNNNNNNNNNNNNNNNNNNNNNNNNNNNNNNNNNNNNNNNNNNNNNNNNNNNNNNNNNNNNNNNNNNNNNNNNNNNNNNNNNNNNNNNNNNNNNNNNNNNNNNNNNNNNNNNNNNNNNNNNNNNNNNNNNNNNNNNNNNNNNNNNNNNNNNNNNNNNNNNNNNTGTGTGTGTATGTGTATGTGTATGTGTGCATGTGTGTGTGTGCATGTGGTGGTTCACAGTCATATGTTCATGCATTATCAACAGTCGAGGGCTGGTTTTCTCCATACTTTGTCTGGACCTTTTCCTCCAGGTCAGGGTTCTGTATCCCCATGGGTTTGGACAGGTCTCTGAACACCTCAGAGTTGCTCAGGTCCAATGTATCACAACTGTAGTCTCGCAGGATCCATGGAAACTAAACACGGAGCAGATACTAATTGATAATTTGCATACAGCTCAAGGATCCTCACAATAGGATATTGGTTGAGGTCATTGTAGGTCCTTCCTGATATAGTGTTGAGTTGCATGATGTAATCAAAATTTGTAATTTCACGTTTCACCCATTTCTATAACAAAATAAACCACATCATTCCAAGTGGAGATGAAGAGGGTAAGGAGATGGAGGTCATGGAAGAGGGGAAGGAGATGGAAGGTCATGGAAGAGGGTAAGGAGATGGAAGGTCATGGAAGAGAGTAAGGAGATGGAGGTCATGGAAGAGAGTAAGGAGATGGAAGGTCATGGAAGAGGGGAAGGAGATGGAAGGTCATGGAANNNNNNNNNNNNNNNNNNNNNNNNNNNNNNNNNNNNNNNNNNNNNNNNNNNNNNNNNNNNNNNNNNNNNNNNNNNNNNNNNNNNNNNNNNNNNNNNNNNNNNNNNNNNNNNNNNNNNNNNNNNNNNNNNNNNNNNNNNNNNNNNNNNNNNNNNNNNNNNNNNNNNNNNNNNNNNNNNNNNNNNNNNNNNNNNNNNNNNNNNNNNNNNNNNNNNNNNNNNNNNNNNNNNNNNNNNNNNNNNNNNNNNNNNNNNNNNNNNNNNNNNNNNNNNNNNNNNNNNNNNNNNNNNNNNNNNNNNNNNNNNNNNNNNNNNNNNNNNNNNNNNNNNNNNNNNNNNNNNNNNNNNNNNNNNNNNNNNNNNNNNNNNNNNNNNNNNNNNNNNNNNNNNNNNNNNNNNNNNNNNNNNNNNNNNNNNNNNNNNNNNNNNNNNNNNNNNNNNNNNNNNNNNNNNNNNNNNNNNNNNNNNNNNNNNNNNNNNNNNNNNNNNNNNNNNNNNNNNNNNNNNNNNNNNNNNNNNNNNNNNNNNNNNNNNNNNNNNNNNNNNNNNNNNNNNNNNNNNNNNNNNNNNNNNNNNNNNNNNNNNNNNNNNNNNNNNNNNNNNNNNNNNNNNNNNNNNNNNNNNNNNNNNNNNNNNNNNNNNNNNNNNNNNNNNNNNNNNNNNNNNNNNNNNNNNNNNNNNNNNNNNNNNNNNNNNNNNNNNNNNNNNNNNNNNNNNNNNNNNNNNNNNNNNNNNNNNNNNNNNNNNNNNNNNNNNNNNNNNNNNNNNNNNNNNNNNNNNNNNNNNNNNNNNNNNNNNNNNNNNNNNNNNNNNNNNNNNNNNNNNNNNNNNNNNNNNNNNNNNNNNNNNNNNNNNNNNNNNNNNNNNNNNNNNNNNNNNNNNNNNNNNNNNNNNNNNNNNNNNNNNNNNNNNNNNNNNNNNNNNNNNNNNNNNNNNNNNNNNNNNNNNNNNNNNNNNNNNNNNNNNNNNNNNNNNNNNNNNNNNNNNNNNNNNNNNNNNNNNNNNNNNNNNNNNNNNNNNNNNNNNNNNNNNNNNNNNNNNNNNNNNNNNNNNNNNNNNNNNNNNNNNNNNNNNNNNNNNNNNNNNNNNNNNNNNNNNNNNNNNNNNNNNNNNNNNNNNNNNNNNNNNNNNNNNNNNNNNNNNNNNNNNNNNNNNNNNNNNNNNNNNNNNNNNNNNNNNNNNNNNNNNNNNNNNNNNNNNNNNNNNNNNNNNNNNNNNNNNNNNNNNNNNNNNNNNNNNNNNNNNNNNNNNNNNNNNNNNNNNNNNNNNNNNNNNNNNNNNNNNNNNNNNNNNNNNNNNNNNNNNNNNNNNNNNNNNNNNNNNNNNNNNNNNNNNNNNNNNNNNNNNNNNNNNNNNNNNNNNNNNNNNNNNNNNNNNNNNNNNNNNNNNNNNNNNNNNNNNNNNNNNNNNNNNNNNNNNNNNNNNNNNNNNNNNNNNNNNNNNNNNNNNNNNNNNNNNNNNNNNNNNNNNNNNNNNNNNNNNNNNNNNNNNNNNNNNNNNNNNNNNNNNNNNNNNNNNNNNNNNNNNNNNNNNNNNNNNNNNNNNNNNNNNNNNNNNNNNNNNNNNNNNNNNNNNNNNNNNNNNNNNNNNNNNNNNNNNNNNNNNNNNNNNNNNNNNNNNNNNNNNNNNNNNNNNNNNNNNNNNNNNNNNNNNNNNNNNNNNNNNNNNNNNNNNNNNNNNNNNNNNNNNNNNNNNNNNNNNNNNNNNNNNNNNNNNNNNNNNNNNNNNNNNNNNNNNNNNNNNNNNNNNNNNNNNNNNNNNNNNNNNNNNNNNNNNNNNNNNNNNNNNNNNNNNNNNNNNNNNNNNNNNNNNNNNNNNNNNNNNNNNNNNNNNNNNNNNNNNNNNNNNNNNNNNNNNNNNNNNNNNNNNNNNNNNNNNNNNNNNNNNNNNNNNNNNNNNNNNNNNNNNNNNNNNNNNNNNNNNNNNNNNNNNNNNNNNNNNNNNNNNNNNNNNNNNNNNNNNNNNNNNNNNNNNNNNNNNNNNNNNNNNNNNNNNNNNNNNNNNNNNNNNNNNNNNNNNNNNNNNNNNNNNNNNNNNNNNNNNNNNNNNNNNNNNNNNNNNNNNNNNNNNNNNNNNNNNNNNNNNNNNNNNNNNNNNNNNNNNNNNNNNNNNNNNNNNNNNNNNNNNNNNNNNNNNNNNNNNNNNNNNNNNNNNNNNNNNNNNNNNNNNNNNNNNNNNNNNNNNNNNNNNNNNNNNNNNNNNNNNNNNNNNNNNNNNNNNNNNNNNNNNNNNNNNNNNNNNNNNNNNNNNNNNNNNNNNNNNNNNNNNNNNNNNNNNNNNNNNNNNNNNNNNNNNNNNNNNNNNNNNNNNNNNNNNNNNNNNNNNNNNNNNNNNNNNNNNNNNNNNNNNNNNNNNNNNNNNNNNNNNNNNNNNNNNNNNNNNNNNNNNNNNNNNNNNNNNNNNNNNNNNNNNNNNNNNNNNNNNNNNNNNNNNNNNNNNNNNNNNNNNNNNNNNNNNNNNNNNNNNNNNNNNNNNNNNNNNNNNCATACACACACCATCACACTGTACACACCATCACATGTACACACCATCACACTGTACACACCATCACACTGTACACACCATCACACTGTACACACCATCACACTGTACACACCATCACAACATACCAAGGCTTCTCGATGTTGTCTTATGAAGTCCTCAGGGGACGACGCCCACTTTGGAAGTATCACATCTCCAACAACTGTTTTGTCTTGGGTGACACCAAGGTCATAGCCTATAAAGACAAGTTAGCAGAGTGCTTAGAAATGAATGCAACCCTTTAAGTNATTAACTAGGCAAACANTGATGGNTATTNTAAGCAGTAATTACTGGATCAAAGTCACTTGAAAGTGAGACCAAATATTCAAGATGAACAACAAAAAAAAAANAAANAAAAAAANAAAAAAANAAATTTAGTTGTTAGTCCTTGAATAAATTAATTAATTAACTAGCAATTATTGAAGTTCACATTTTAATTTTTTGAGAGCTTTTTTGAAGCTTGAGGAGTTGGCTTTGTATTTCCCAAATATTTATTAAACCATTCCGCAATGTATTAATTATGAGTACTTATTGGAACAATTGACCTAACCATTTATATTCCTCAAGAAATCCGGTAGGCAGAAAAACTCTGGTGTAAGTTCTTTCACATCACTTCCTAAATAGGTGATTCTCCAAGCATTGGGTATGGAGAAGAACTGTCTATCAGGGTGATCAAACCTAGAGTATTAGCAATATAGCAGAGTGGGTAAGATTGAAGTGAAGATGGGTCAAGATACCATCCCAGCTGTATGTGGCACCTGTTCCCACACAACTCACTTTCCACTCTGTAGGTTGATGTGCAGGGATGTGAAGGGCTNCANTACGNNNNNAGTAGTGGATGACAGTTGTGGCATTGCTGTAGTGTGTNCCNTAGTGAAACTTGCCCATCACTGGATCGTCAAATTCCTCATATCTGCAAGTGCACAGTGCGCATAAGTGTTGTGTGTATAATTGACCACTGTGTCAACACCACATGACACTTTTAAATGGCTGGCGTCCATACTTTGTCCGGACCTTTTCCTCCAGGTCAGGGTTCTGTATCCCCATGGGTTTGGACAGGTCTCTGAACACCTCAGAGTTGCTCAGGTCCAATGTATCACAACTGTAGTCTTGCAGGATCCATGGAAACTAAACACGGAGCAGATACTAAATGATAATTTGCATACAGCTCAAGTATCCTCACAATAGGATATTGGTTGAGGTCATTGTAGGTCCTTCCTGATATAGTGTTGAGTTGCATGATGTAATCAAAATTTGTAATTTCACGTTTCACCCATTTCTACAGTTGTGCAGTTGAATAATGAAAATGAAAGCAGTAGTGAAGGTGGTCATGGAGGAGGTAAGGAATGAGGGAGAGGTGGTCATGGAGGAGGTAAGGGGGAGAGGTGGTCATGGAGGAGGTAAGGGGGAGAGGTGGCCATGGAGGAGGTAAGGATGTAAGGGAGGAGGTGGAGGTGGTGAGGGAGGAGAAAAATTAACACAAGGTGTACAGACAGGGTATAACGACAGTTAGTGTATTCCCAACCTGAATTAGACCTGAAGACTGCATCAGTTTGGCAGGCTCTTGTGTGCTGAAGATGTTAAGGTTTGGAGTACTCAGCGAAACAATGCAGTTGCACACCTTATTCAAAATCTGTGCAACAAGGAGAGAGATACATGCACACCACTTAACACCCAGTCTGGAGACAATGAAAACACTGTGCCTTTTTGGTGGGAAAATTAAGGAAGTAGTTTGTCTGATCCAAAAGAAACATCTCCAGTGCAGTACGGCTTGTGTTGTACCTACGAAGGTGAAGCTCCCTTATCTGGTTAAGTTTGACTGTGAAGTCAGTGAGGTACCCTGCGAAAGAAAGAGAAAATGAATAACCATTGGAGCCTTGCCTTAGGAAGAGGCACTAATAGTTCAATGTCAGATATATCTGGTCACATGTCTTAGCACAAAGTCAACTGTCATACACCTATGCTATTCTAAGTAGTGCACACTCTTGTTACAGATATGCTACTCCATGTTTACAGATATGCTACTCCAAGTGCACACNNTTGTTACAGATATGCTACTCCAAGTGCACACTTTTGTTACAGATATGCTACTACAAGTGCACACTTTTGTTACAGATATGCTACTCCAAGTGCACACTCTTGTTCCTCCCTTCTTCATTTCCTCCTCCCTATCTGTGATCACACCACCCCTTTAACTCTCCCGGTACACTCACAAGATATCGCATCCTTGTCTTGTGCATCCGACACAAAGTAGATGCATCTATTTGTAATGTCCAAGGTTCCAGAGATGACCTCTACCACCGTCACAAGCTGACACATCTCTCTTATNATCAGTTTCTCTTTCTTGCCCTTCTCCAGAGCCACAGCTGGCTCATCTCGAGCTAGCTTTTGGATCTGATCAAACAGATGTTCCTCCTCCTCCTCACTGGNGGACTTTGTAGCTGTGGCCATGGCTAGCAGAGAGGTGTCTGCAGTGCTGGACACCAGGNGAGTCTCCCCAGAGCTGTGTTGACTAGCATCGGTGTGTTTGTTATAGTTGTAGTTACGGGCCAGTTTTAGCCGCATGCGTGAAAAGTTCTCTGTTCTGTCCATCTTCCACAGGTAGAGATCAGGATCTCTGGAACAGAGGATCACGGAATCACAGGGTTATGTACATATACATTCATGTGTGCCACACAGCACCACACAGAGGTACATACATGTATGTACTGTTCCATCATGTATAAAACATGAGTACAACTTCATGTACAACATCATGGGACATGTGTCCCCATGGAACATGTGTACCCATGTACAACATCATGGAACATGTGTACCATCATGTACAACATATGTACCATCAATGTACTANATACTGGCACATAGTGGGGACATGTGTACCATCATGTACNNNNNNNNNNNNNNNNNNNNNNNNNNNNNNNNNNNNNNNNNNNNNNNNNNNNNNNNNNNNNNNNNNNNNNNNNNNNNNNNNNNNNNNNNNNNNNNNNNNNNNNNNNNNNNNNNNNNNNNNNNNNNNNNNNNNNNNNNNNNNNNNNNNNNNNNNNNNNNNNNNNNNNNNNNNNNNNNNNNNNNNNNNNNNNNNNNNNNNNNNNNNNNNNNNNNNNNNNNNNNNNNNNNNNNNNNNNNNNNNNNNNNNNNNNNNNNNNNNNNNNNNNNNNNNNNNNNNNNNNNNNNNNNNNNNNNNNNNNNNNNNNNNNNNNNNNNNNNNNNNNNNNNNNNNNNNNNNNNNNNNNNNNNNNNNNNNNNNNNNNNNNNNNNNNNNNNNNNNNNNNNNNNNNNNNNNNNNNNNNNNNNNNNNNNNNNNNNNNNNNNNNNNNNNNNNNNNNNNNNNNNNNNNNNNNNNNNNNNNNNNNNNNNNNNNNNNNNNNNNNNNNNNNNNNNNNNNNNNNNNNNNNNNNNNNNNNNNNNNNNNNNNNNNNNNNNNNNNNNNNNNNNNNNNNNNNNNNNNNNNNNNNNNNNNNNNNNNNNNNNNNNNNNNNNNNNNNNNNNNNNNNNNNNNNNNNNNNNNNNNNNNNNNNNNNNNNNNNNNNNNNNNNNNNNNNNNNNNNNNNNNNNNNNNNNNNNNNNNNNNNNNNNNNNNNNNNNNNNNNNNNNNNNNNNNNNNNNNNNNNNNNNNNNNNNNNNNNNNNNNNNNNNNNNNNNNNNNNNNNNNNNNNNNNNNNNNNNNNNNNNNNNNNNNNNNNNNNNNNNNNNNNNNNNNNNNNNNNNNNNNNNNNNNNNNNNNNNNNNNNNNNNNNNNNNNNNNNNNNNNNNNNNNNNNNNNNNNNNNNNNNNNNNNNNNNNNNNNNNNNNNNNNNNNNNNNNNNNNNNNNNNNNNNNNNNNNNNNNNNNNNNNNNNNNNNNNNNNNNNNNNNNNNNNNNNNNNNNNNNNNNNNNNNNNNNNNNNNNNNNNNNNNNNNNNNNNNNNNNNNNNNNNNNNNNNNNNNNNNNNNNNNNNNNNNNNNNNNNNNNNNNNNNNNNNNNNNNNNNNNNNNNNNNNNNNNNNNNNNNNNNNNNNNNNNNNNNNNNNNNNNNNNNNNNNNNNNNNNNNNNNNNNNNNNNNNNNNNNNNNNNNNNNNNNNNNNNNNNNNNNNNNNNNNNNNNNNNNNNNNNNNNNNNNNNNNNNNNNNNNNNNNNNNNNNNNNNNNNNNNNNNNNNNNNNNNNNNNNNNNNNNNNNNNNNNNNNNNNNNNNNNNNNNNNNNNNNNNNNNNNNNNNNNNNNNNNNNNNNNNNNNNNNNNNNNNNNNNNNNNNNNNNNNNNNNNNNNNNNNNNNNNNNNNNNNNNNNNNNNNNNNNNNNNNNNNNNNNNNNNNNNNNNNNNNNNNNNNNNNNNNNNNNNNNNNNNNNNNNNNNNNNNNNNNNNNNNNNNNNNNNNNNNNNNNNNNNNNNNNNNNNNNNNNNNNNNNNNNNNNNNNNNNNNNNNNNNNNNNNNNNNNNNNNNNNNNNNNNNNNNNNNNNNNNNNNNNNNNNNNNNNNNNNNNNNNNNNNNNNNNNNNNNNNNNNNNNNNNNNNNNNNNNNNNNNNNNNNNNNNNNNNNNNNNNNNNNNNNNNNNNNNNNNNNNNNNNNNNNNNNNNNNNNNNNNNNNNNNNNNNNNNNNNNNNNNNNNNNNNNNNNNNNNNNNNNNNNNNNNNNNNNNNNNNNNNNNNNNNNNNNNNNNNNNNNNNNNNNNNNNNNNNNNNNNNNNNNNNNNNNNNNNNNNNNNNNNNNNNNNNNNNNNNNNNNNNNNNNNNNNNNNNNNNNNNNNNNNNNNNNNNNNNNNNNNNNNNNNNNNNNNNNNNNNNNNNNNNNNNNNNNNNNNNNNNNNNNNNNNNNNNNNNNNNNNNNNNNNNNNNNNNNNNNNNNNNNNNNNNNNNNNNNNNNNNNNNNNNNNNNNNNNNNNNNNNNNNNNNNNNNNNNNNNNNNNNNNNNNNNNNNNNNNNNNNNNNNNNNNNNNNNNNNNNNNNNNNNNNNNNNNNNNNNNNNNNNNNNNNNNNNNNNNNNNNNNNNNNNNNNNNNNNNNNNNNNNNNNNNNNNNNNNNNNNNNNNNNNNNNNNNNNNNNNNNNNNNNNNNNNNNNNNNNNNNNNNNNNNNNNNNNNNNNNNNNNNNNNNNNNNNNNNNNNNNNNNNNNNNNNNNNNNNNNNNNNNNNNNNNNNNNNNNNNNNNNNNNNNNNNNNNNNNNNNNNNNNNNNNNNNNNNNNNNNNNNNNNNNNNNNNNNNNNNNNNNNNNNNNNNNNNNNNNNNNNNNNNNNNNNNNNNNNNNNNNNNNNNNNNNNNNNNNNNNNNNNNNNNNNNNNNNNNNNNNNNNNNNNNNNNNNNNNNNNNNNNNNNNNNNNNNNNNNNNNNNNNNNNNNNNNNNNNNNNNNNNNNNNNNNNNNNNNNNNNNNNNNNNNNNNNNNNNNNNNNNNNNNNNNNNNNNNNNNNNNNNNNNNNNNNNNNNNNNNNNNNNNNNNNNNNNNNNNNNNNNNNNNNNNNNNNNNNNNNNNNNNNNNNNNNNNNNNNNNNNNNNNNNNNNNNNNNNNNNNNNNNNNNNNNNNNNNNNNNNNNNNNNNNNNNNNNNNNNNNNNNNNNNNNNNNNNNNNNNNNNNNNNNNNNNNNNNNNNNNNNNNNNNNNNNNNNNNNNNNNNNNNNNNNNNNNNNNNNNNNNNNNNNNNNNNNNNNNNNNNNNNNNNNNNNNNNNNNNNNNNNNNNNNNNNNNNNNNNNNNNNNNNNNNNNNNNNNNNNNNNNNNNNNNNNNNNNNNNNNNNNNNNNNNNNNNNNNNNNNNNNNNNNNNNNNNNNNNNNNNNNNNNNNNNNNNNNNNNNNNNNNNNNNNNNNNNNNNNNNNNNNNNNNNNNNNNNNNNNNNNNNNNNNNNNNNNNNNNNNNNNNNNNNNNNNNNNNNNNNNNNNNNNNNNNNNNNNNNNNNNNNNNNNNNNNNNNNNNNNNNNNNNNNNNNNNNNNNNNNNNNNNNNNNNNNNNNNNNNNNNNNNNNNNNNNNNNNNNNNNNNNNNNNNNNNNNNNNNNNNNNNNNNNNNNNNNNNNNNNNNNNNNNNNNNNNNNNNNNNNNNNNNNNNNNNNNNNNNNNNNNNNNNNNNNNNNNNNNNNNNNNNNNNNNNNNNNNNNNNNNNNNNNNNNNNNNNNNNNNNNNNNNNNNNNNNNNNNNNNNNNNNNNNNNNNNNNNNNNNNNNNNNNNNNNNNNNNNNNNNNNNNNNNNNNNNNNNNNNNNNNNNNNNNNNNNNNNNNNNNNNNNNNNNNNNNNNNNNNNNNNNNNNNNNNNNNNNNNNNNNNNNNNNNNNNNNNNNNNNNNNNNNNNNNNNNNNNNNNNNNNNNNNNNNNNNNNNNNNNNNNNNNNNNNNNNNNNNNNNNNNNNNNNNNNNNNNNNNNNNNNNNNNNNNNNNNNNNNNNNNNNNNNNNNNNNNNNNNNNNNNNNNNNNNNNNNNNNNNNNNNNNNNNNNNNNNNNNNNNNNNNNNNNNNNNNNNNNNNNNNNNNNNNNNNNNNNNNNNNNNNNNNNNNNNNNNNNNNNNNNNNNNNNNNNNNNNNNNNNNNNNNNNNNNNNNNNNNNNNNNNNNNNNNNNNNNNNNNNNNNNNNNNNNNNNNNNNNNNNNNNNNNNNNNNNNNNNNNNNNNNNNNNNNNNNNNNNNNNNNNNNNNNNNNNNNNNNNNNNNNNNNNNNNNNNNNNNNNNNNNNNNNNNNNNNNNNNNNNNNNNNNNNNNNNNNNNNNNNNNNNNNNNNNNNNNNNNNNNNNNNNNNNNNNNNNNNNNNNNNNNNNNNNNNNNNNNNNNNNNNNNNNNNNNNNNNNNNNNNNNNNNNNNNNNNNNNNNNNNNNNNNNNNNNNNNNNNNNNNNNNNNNNNNNNNNNNNNNNNNNNNNNNNNNNNNNNNNNNNNNNNNNNNNNNNNNNNNNNNNNNNNNNNNNNNNNNNNNNNNNNNNNNNNNNNNNNNNNNNNNNNNNNNNNNNNNNNNNNNNNNNNNNNNNNNNNNNNNNNNNNNNNNNNNNNNNNNNNNNNNNNNNNNNNNNNNNNNNNNNNNNNNNNNNNNNNNNNNNNNNNNNNNNNNNNNNNNNNNNNNNNNNNNNNNNNNNNNNNNNNNNNNNNNNNNNNNNNNNNNNNNNNNNNNNNNNNNNNNNNNNNNNNNNNNNNNNNNNNNNNNNNNNNNNNNNNNNNNNNNNNNNNNNNNNNNNNNNNNNNNNNNNNNNNNNNNNNNNNNNNNNNNNNNNNNNNNNNNNNNNNNNNNNNNNNNNNNNNNNNNNNNNCCACACACACATACCCCACNCACACACACACACACACACACACACACACCACACACAAACACACACCAATGAAGCAGCTGTGGGGTTGCTCTACAAATGGAGTACCTCTACAACATTAGCCATGAGGTCATCTCTCGTTTGACCTCTTTGATCGTCCCCCTTACGCACCACCTGCATTTGCTCAAAGAGGTTCACTATGACTCTCTTGCCAGTGAAGGGAGAGGAGTAGCCTCTGTAGCTCTTCACAGGCGGGTCTCTTGTCGGATGCCTCTCGTTAAACAGACGAGTTGGTGTCTGACCAAAATTGGTTATAATACTTTCAATCTCTCGGCGGCTTTTTGGGTCTTGGATGTTGTCTAAGTTGACATTTTCTTTATAAGGGAAGTGAAAATATTAACAACATTGAAGATTAGACAATAAGGTCCCAATTATCCATGTACCTTGGTATGAGTACTTGTTAAACACATTCATAGCCTTTTCTGCCTCAGGCCCAGTCTGTTTGTACCCAAAAATGAGGTCAATCCACTCATTGAGGTGTGCTGAAACGTATTCAGANTCCTGAGGGAGGAGGGTAGAGCACAATGNACACACATGCTCCTACNNGNNATTACACTGTACACACCATCACACTGTACACACCATCACAC>scaffold1288|size38923CACACCAGAGNGNNNNTGTGTNACAGNGNGCCCATGTTCCTATGTTCACATGTACCCATGTTCCAATGTTCACACCTGCCCATATTCCTATGTTCACACGTACCCATGTTCCAATGTTCGCACCTGCCCATGTTCCTATGTTCACATGTGCCCATGCTCCTATGTCCTCATGTGCCCATGTTCCTCCTATATCCTGAGTCTTCATATCAAAACCTGCTTTAATATCAACAGNCCCTAAGATGGTTTCTGCCTTGTACTAGATAACTTATTTCTTTAAGCCTTTGAGTTCTTGAATATGTGGGACAATGTAATCCAACAAAGGAGTATTATTGTGAATAAGAAAGAATTGGACAATGTTCAAGATGATGAACTGTATTAGCTAACATGCAACTNTAAATGCCTTGAGAATGGAGTCTTGTTACCATGGCAGTTGTTACCATGGCAGTTGTTCTTTTCTATACCTTAGCTCTGGCAGACAAGAAAGAGCTCAAGTGCTTGGAAATATTGACATGTGCTCAGAATATNATCACTGATATGCAAAAGGCATTGTCAAGACGAGACTAGAGCTATGGCAGTGCATATTAACTTGTACCATTATTTTATATTTCATTTTTAATTGCACCAATACCATGTAGGCCTCCTGTGAGCATGCCTGCACACATCTCCCCTCCCTTTACCCAACACGTAACACAGATGAACCTTGCACCACACATGGTACAGAGTAGAGACTGCATGACCCCTCCTCCCATGACCCCTCCTTCCATGGTCCCTTCTCCCATGACCCCTCCTTCCATGGTCCCTCCTCCCATGACCCCTCCTCCCATGACCCCTCCTCCCATGGTCCCTGCTTCCCTTCATTCTATTACTGCACACAGCATCTGTTGGCAGACTTTGGCTCAAAGTGGATGAGGGTGTTGTAAAAGGTCATAATATGATGGTATGTGAAGGTCATGTGAGGGTACCTGAAGGTCATGTGAGGGTACATGAAGGTCATGTGAAAGTACATAAAGGTCATGTGGATATATGTGTCAGAAATGATGTGCGAGATAAAATACATTTATACTGTCAAATTTGATGCTATGCTGTCAGCAGTGTACTTGGGGTAACCTAACTCTTTCAGCATGTCACACTCCTCCCATATCTGCTCCAAGATGGCCGGTGGTATGCTAGTACGCCATGTATCCAAGGACTCCGTGTTGACCGGCTTCTTCACCTGATTGGTCGAAGGCTCCATTCTAAATAGTGGTATGATGCATGGAGGCAATGAAAATATTTAAATACCCGGATTGCCCCTTTGGGGCACCCGGGAGGGANNNNNNNNNNNNNNNNNNNNNNNNNNNNNNNNNNNNNNNNNNNNNNNNNNNNNNNNNNNNNNNNNNNNNNNNNNNNNNNNNNNNNNNNNNNNNNNNNNNNNNNNNNNNNNNNNNNNNNNNNNNNNNNNNNNNNNNNNNNNNNNNNNNNNNNNNNNNNNNNNNNNNNNNNNNNNNNNNNNNNNNNNNNNNNNNNNNNNNNNNNNNNNNNNNNNNNNNNNNNNNNNNNNNNNNNNNNNNNNNNNNNNNNNNNNNNNNNNNNNNNNNNNNNNNNNNNNNNNNNNNNNNNNNNNNNNNNNNNNNNNNNNNNNNNNNNNNNNNNNNNNNNNNNNNNNNNNNNNNNNNNNNNNNNNNNNNNNNNNNNNNNNNNNNNNNNNNNNNNNNNNNNNNNNNNNNNNNNNNNNNNNNNNNNNNNNNNNNNNNNNNNNNNNNNNNNNNNNNNNNNNNNNNNNNNNNNNNNNNNNNNNNNNNNNNNNNNNNNNNNNNNNNNNNNNNNNNNNNNNNNNNNNNNNNNNNNNNNNNNNNNNNNNNNNNNNNNNNNNNNNNNNNNNNNNNNNNNNNNNNNNNNNNNNNNNNNNNNNNNNNNNNNNNNNNNNNNNNNNNNNNNNNNNNNNNNNNNNNNNNNNNNNNNNNNNNNNNNNNNNNNNNNNNNNNNNNNNNNNNNNNNNNNNNNNNNNNNNNNNNNNNNNNNNNNNNNNNNNNNNNNNNNNNNNNNNNNNNNNNNNNNNNNNNNNNNNNNNNNNNNNNNNNNNNNNNNNNNNNNNNNNNNNNNNNNNNNNNNNNNNNNNNNNNNNNNNNNNNNNNNNNNNNNNNNNNNNNNNNNNNNNNNNNNNNNNNNNNNNNNNNNNNNNNNNNNNNNNNNNNNNNNNNNNNNNNNNNNNNNNNNNNNNNNNNNNNNNNNNCACACACACACACACACACACACACTGTTCAGCTACGTCTGGGCTCACATGAGTGATGGGTACTCCCAGGGGATGTGTGACCTTCTGGCACCTCTGCTTGTGGTGTTTGACGATGGTGAGGCATTACAGGTGGGAGGGGCTGGTGAGGCATTACAGGTGGGAGGGGCTGGTGAGGCTTGGTTGGGGCAGTGGCTGAAGGTTGTGTCCGGTGAGGTGTCCGGGGGATTGAGCATTACAGGTGGGAGGGGCACACACGTAATCTCATACACATGTATGAGTATGTGTGTATGTGACGTGTATGTTTGTATGCATGTATGCACATGTGCATGTATCAAGATTTGTACATGCCACACTGTGTCTCTCTCTGCCTTCTCTCAGAGTCACTTGCATACAGCTGTTACTTGAAGCTGATGGCGACACAAAGAGAATTGTTTCCACCTGAAGTTGGTATGAACACACGAATGACCAANAGGGAGGGGGGGAGGTAAGAGGTGAGGGAGAGGTAGGAGGGGAGGGGGAGAAGGTAGGAGGAGAGTGGGAGGTAGGAGGTGAGAGGGAGGGGGAGGTAAGAGGTGAGGGGGAGGTAGGAGGGGAGCAGGAGGTAGGAGAGAGGGAGTGGGAGGAGGTGAGGAAGCGAGGTAGGTGAGNGAGGTAGGGGCAGGTGGGAGGAGAGAGGTGATGGAAGGGAGGTAGGTGAGAGAGGGAGGGGCAGGTGGGAGGAGAGAGGTGATGGAAGGGAGGNAGTATAAATGTGATTGATGTGATTTGATCAAGGCTGGTAGTGTTGTGTCCTCGCAACATTCTGATGCGTAATTTTGATTACTCTTCATTGCCCCCACCCTCCCCCTCCTCACCCCTCCCATCCTCTCAGGTGACAGATACAGATTTCTTTCAATACCTTCAGGAAAAGCCAATGGGTGACAGTCTGTTTTATTGCTACCGTTGGTTTCTGGTGGGCTTTAAAAGAGGTACAGAGGGTCATTTGTGTGTGTGTGTCTATGTGTATGTGGAGAGAGGATTGTGCTGGGGATGAGGAGGGTTAATGTGATCAATTAGCCAGAACAGGACGTTAATATGTTTATAGAATGATGCATGCTTGTTTTTTGCTTGTGGTTTCATGTTCACAAATCAAGTTTATTTTGTTGACAAAAACTACACATCGTTGCTTAGGCTTGGTGGGTGGGTGTGGTCATCAGTGATTGTACACTTGATTACTTACGTAGTTTGGGAGGAGTGATCATGTGATATATGCATCCTGGTCGTCAGAGTTCGAATATGAACATGTGTTTCGTCTCTGGGAGGCCACATGGGCTGCAAAAGTTCTGGTCTCTGTCCATTTTGAGGAGTTCTTTGCTTTGGCCATCATAAACCAATACAAGTAAGGCACCTACCTCCCCTCTCGTCCCTCTCCTTTAACCCTTTGCCTTCCGTCCCAAGGAATGCCATCATGGATGCACACATGGACCCCAGTGATATTGTCAATCTGTTCACTTGTAAGATGTTCGATGGATGTGTGTTCCTTGCTCCTCTGCTCCCACGTTCTCATGTTCCTTTGCTCTCACNNNNNNNNNNNNNNNNNNNNNNNNNNNNNNNNNNNNNNNNNNNNNNNNNNNNNNNNNNNNNNNNNNNNNNNNNNNNNNNNNNNNNNNNNNNNNNNNNNNNNNNNNNNNNNNNNNNNNNNNNNNNNNNNNNNNNNNNNNNNNNNNNNNNNNNNNNNNNNNNNNNNNNNNNNNNNNNNNNNNNNNNNNNNNNNNNNNNNNNNNNNNNNNNNNNNNNNNNNNNNNNNNNNNNNNNNNNNNNNNNNNNNNNNNNNNNNNNNNNNNNNNNNNNNNNNNNNNNNNNNNNNNNNNNNNNNNNNNNNNNNNNNNNNNNNNNNNNNNNNNNNNNNNNNNNNNNNNNNNNNNNNNNNNNNNNNNNNNNNNNNNNNNNNNNNNNNNNNNNNNNNNNNNNNNNNNNNNNNNNNNNNNNNNNNNNNNNNNNNNNNNNNNNNNNNNNNNNNNNNNNNNNNNNNNNNNNNNNNNNNNNNNNNNNNNNNNNNNNNNNNNNNNNNNNNNNNNNNNNNNNNNNNNNNNNNNNNNNNNNNNNNNNNNNNNNNNNNNNNNNNNNNNNNNNNNNNNNNNNNNNNNNNNNNNNNNNNNNNNNNNNNNNNNNNNNNNNNNNNNNNNNNNNNNNNNNNNNNNNNNNNNNNNNNNNNNNNNNNNNNNNNNNNNNNNNNNNNNNNNNNNNNNNNNNNNNNNNNNNNNNNNNNNNNNNNNNNNNNNNNNNNNNNNNNNNNNNNNNNNNNNNNNNNNNNNNNNNNNNNNNNNNNNNNNNNNNNNNNNNNNNNNNNNNNNNNNNNNNNNNNNNNNNNNNNNNNNNNNNNNNNNNNNNNNNNNNNNNNNNNNNNNNNNNNNNNNNNNNNNNNNNNNNNNNNNNNNNNNNNNNNNNNNNNNNNNNNNNNNNNNNNNNNNNNNNNNNNNNNNNNNNNNNNNNNNNNNNNNNNNNNNNNNNNNNNNNNNNNNNNNNNNNNNNNNNNNNNNNNNNNNNNNNNNNNNNNNNNNNNNNNNNNNNNNNNNNNNNNNNNNNNNNNNNNNNNNNNNNNNNNNNNNNNNNNNNNNNNNNNNNNNNNNNNNNNNNNNNNNNNNNNNNNNNNNNNNNNNNNNNNNNNNNNNNNNNNNNNNNNNNNNNNNNNNNNNNNNNNNNNNNNNNNNNNNNNNNNNNNNNNNNNNNNNNNNCACACACATACATGCACACACACACACATACATGCACACTGAACATGCACACATCCACACACATACATGCACACATCCACACACATACATGCACACACACACACTGAACATGCACACATCCACACACATGCATGCACANTGAACATGCACACATCCACATCCACTCACTCGCATATGCATGCTAACAGCACACTCACAACATGCTCACAAACGTGCACACGCACACTCACAAATGCACACCAACACACGTACACACACTCACACACTCACACCTCACCATAGAGGATTCTCGCATAAAAAGCACACTCCATCACAAACCNCTCCCTGTAGGTGTGGCCATACCTCCTAGGCCTCTACCATCCGAGCAACACTCCAGACCAATGCTCCACCATTCGCAGGCAGTGTGTCGCTGACTACAGCCGGCTTATGGACGAGTGGAGACCCCTCAATGAACTTTACACCCACCAGCAACTGCAGAGGCACACAGACTCTGCCAAGGCGTGGAGGAGGGTGCTCAGTACTCCGTCTGTCGTCTCCGCCCCCAAATCTGCCCAAGGGGAGACCTCGGGGTTGCCGGGGGCAACCGAGGAGGTCGCGGAAGGATCAGGCGGCGACACGGGGGCACGGGAAGTGGGAGGAGCCATGCCGAACGGTCCTTGTTTGCAGGAGGCGGGGTATTCGGGCATCGCCCCCCTCGGGAGGGTCCCGTCTACCGATTCTGCTGCTCCCGCCCCCAAGATGGAGGTGGGGTCGGGAAACGTTCCAGCTGCCTTGCTTGGGAGTGTTGACGAGATACAGATCCACGTAGAGGAGTGTAGCCACACAACAGCGGAGGCCTTCTACTCGTACTGCGATGCTGAAGTGAGGGCCCCAGCACCTGCTGAGGATCTCTCCACCTCAATCTCCACCTACGAAACTAAGGAGTCAGCTGCTGAGCCTGGTGTGAAGAAGGGGGAGGAGGAGGAGGAAGAGGAGGAGGGGGAGATCATTGTACCTGTGGACCCAAAGGCCCAGGCGTTTATGGATGAACTGTACAAGATTGACAAGGACATTCCGAGATGCGATCGTGAGTACTGGTGAGTAGGTTATCTTTGCTGTGGTATGGTGTTGATGGTCGTGGGTTGGACGTGCTGTCAGACTAACTTCTACTTCCATATTCTCCTTCACTCTCCCCTGTCTTTTCTCCTTCCTCCCTCACTCCCTCTCCTCCCCCCCCCCCCTCTCCCCCCGATTNNNNNNNNNNNNNNNNNNNNNNNNNNNNNNNNNNNNNNNNNNNNNNNNNNNNNNNNNNNNNNNNNNNNNNNNNNNNNNNNNNNNNNNNNNNNNNNNNNNNNNNNNNNNNNNNNNNNNNNNNNNNNNNNNNNNNNNNNNNNNNNNNNNNNNNNNNNNNNNNNNNNNNNNNNNNNNNNNNNNNNNNNNNNNNNNNNNNNNNNNNNNNNNNNNNNNNNNNNNNNNNNNNNNNNNNNNNNNNNNNNNNNNNNNNNNNNNNNNNNNNNNNNNNNNNNNNNNNNNNNNNNNNNNNNNNNNNNNNNNNNNNNNNNNNNNNNNNNNNNNNNNNNNNNNNNNNNNNNNNNNNNNNNNNNNNNNNNNNNNNNNNNNNNNNNNNNNNNNNNNNNNNNNNNNNNNNNNNNNNNNNNNNNNNNNNNNNNNNNNNNNNNNNNNNNNNNNNNNNNNNNNNNNNNNNNNNNNNNNNNNNNNNNNNNNNNNNNNNNNNNNNNNNNNNNNNNNNNNNNNNNNNNNNNNNNNNNNNNNNNNNNNNNNNNNNNNNNNNNNNNNNNNNNNNNNNNNNNNNNNNNNNNNNNNNNNNNNNNNNNNNNNNNNNNNNNNNNNNNNNNNNNNNNNNNNNNNNNNNNNNNNNNNNNNNNNNNNNNNNNNNNNNNNNNNNNNNNNNNNNNNNNNNNNNNNNNNNNNNNNNNNNNNNNNNNNNNNNNNNNNNNNNNNNNGGGGGGGGGGGGGGGGGGGGGGGGATATGGGACCCATTCTACTCCTGATATGGGAATGGTACCCTGAAGACCATATTTGTGTGGGTATAATATTTTCGTGGCTGTTTGATATATGGAAGCTATTGTCTAATGTGTGACATGGGCATGTATGTCTCTATTCCGACATGCAGTTTACCCAAATCCCAGTACTGTTCAGTCATGTGATCAATAGCCATGGTACCAGCCCATGTAATCAATAAGATTAGTGTAGCGTTACTGATAAGTGCATATAAATGCCTTTGCCTTTTGTGCACTAAAACATTCGCAACGTTAATTTGTGGCCACAAATGTTTCTGTTCTGGTATTCCCCCTCCCATTCCATCCCCTCCTCTCATCCTCTCATCCTTACCATCCCTCCTTCTCACCCTCCCTCCTCCTCACCCCTCCCTCCTCCTCACCTTCCTTCCTCCTCACCCCTCCCTCCTCCTCATCCCTTCCTCCTCCTCACCCTCCCTTCCTCTCCACCCCTCCCTCCTCCCCATCCTCCTCCCATTCCTCCTCCCATCCCTCTTCCTCCCTCGTAGGGGCTGCCCCCTCTTCGCCCTTCACANTNNNNNNNNNCAGGACCCTCCCTATTGGGCGTGGCTCCTCCTCCCCAGATGATGTGCAGGACCCCAGTGCTGTCGTCAGCAAGCCTGGCTCTCTGGCAAGGTGTGTGAAGCATGTGTTGTAAAGGCATCTGAACCAGCTCTGCTTACCATTTGNCTACATGTGGTCAGTNGNNGGNNCCTGGTATGGGTATCGGCCCAACTTATGCTTCCACATGAGGATCTGGTGGTGGTGGTGTATATGTGTGTCAGTAGTTGCCATGGAGAAAAGACAATAGCTACACTCTTATCACAATATGGGCACAGTAACTATTTGAAGAGAAGAAAAAAGTCTGGGCACCAGATTGGGTCAGGAGCTAAGCCTGGGGACCAATTTGGGATGGGAACTGTATTCTGGTGACCTTATATAAGGGACCAATTTGAGATGGGGTGCAAGTCCTGGGGACTGGGCATAACATCTACTCCTTGATGGGTTTTGTATTACAATCAATACATGTAAATGTTAATTGACAATAATCATAATTAAACCCCTACCCAGGGGAGTTGTGGCACATATGTCTACACGAGAGCGCATCACAGTCACATAAAAGCGTTACATGCTCCATTGGGTGTTACTACGTACTGTGGAGCAATAATTCATTAACCAGGAGTGGACGATTTTTAATTGGCTGCGCCAGTTGATAAACATGTGGCACCTCATGCCAGTTAATAAACATGTAGCTATGAACATCTCAGCAGCAGTGATGTGGAGGTGATTATTTAACAGGTGGTTTTCATATCATAGGAAGGACTGGGAGAAATTTCAAACCAAGGTTTCCAGACAGCTGCTAGCAAGATACTTCTATGGCTGTAAGTATGTTGCTTAGAATGTCAACCATCATCTTCCTCCATCTCCTTCTCCCCACTCCATCTCCTCCCCATTCATCCTTCCACCATTCTTCCTCCTCCCCATTCCTCCCCATTTCTCCTCAATCCCATTCCTCCTCCCCATTCCTGCACCAAGGGTGGTCATACTGTCGTCACGTGCACAACATGAAGAAGAGGTTGTCTCANATGATCAGCACTAATGACGTGGTAACCGTGGAGACACATGATGGGCTTGATGAAGCCACGTGGAAGCAGTTCCAACAAGATCAGCAGGTTTGNNNTGGGNNTCANNNNGATACGTGTATCCTTTGCACAGGATACATGTATCTGCATGCGTACATATATTTGCCCTTAACCCTTTCCTCCTCCCTCAAATGNNCAGGGCACACAATGGAGAGACCTTCTGAAGGCCGTCTACTTGGGAGGCGTGGCCAGCAGCCTTAGACCAAAGGTACAGTTCCTGGAGTTGTTTTGTAGAAGCCACTTTGGTTGGCTGGTATTTCCTTCCAGTGGTCTGTGGTCAACGAAGCAGTAGAGATGTNCACACACCTTGCAATTGCACGCGCCCACACACAACTGTGCAATTACATGTGTGCACACATGCACACTAACACATCCACACACACACATGCACACTCACACACACACTGAACATGCACACATCCACACACATACANNNNNNNNNNNNNNNNNNNNNNNNNNNNNNNNNNNNNNNNNNNNNNNNNNNNNNNNNNNNNNNNNNNNNNNNNNNNNNNNNNNNNNNNNNNNNNNNNNNNNNNNNNNNNNNNNNNNNNNNNNNNNNNNNNNNNNNNNNNNNNNNNNNNNNNNNNNNNNNNNNNNNNNNNNNNNNNNNNNNNNNNNNNNNNNNNNNNNNNNNNNNNNNNNNNNNNNNNNNNNNNNNNNNNNNNNNNNNNNNNNNNNNNNNNNNNNNNNNNNNNNNNNNNNNNNNNNNNNNNNNNNNNNNNNNNNNNNNNNNNNNNNNNNNNNNNNNNNNNNNNNNNNNNNNNNNNNNNNNNNNNNNNNNNNNNNNNNNNNNNNNNNNNNNNNNNNNNNNNNNNNNNNNNNNNNNNNNNNNNNNNNNNNNNNNNNNNNNNNNNNNNNNNNNNNNNNNNNNNNNNNNNNNNNNNNNNNNNNNNNNNNNNNNNNNNNNNNNNNNNNNNNNNNNNNNNNNNNNNNNNNNNNNNNNNNNNNNNNNNNNNNNNNNNNNNNNNNNNNNNNNNNNNNNNNNNNNNNNNNNNNNNNNNNNNNNNNNNNNNNNNNNNNNNNNNNNNNNNNNNNNNNNNNNNNNNNNNNNNNNNNNNNNNNNNNNNNNNNNNNNNNNNNNNNNNNNNNNNNNNNNNNNNNNNNNNNNNNNNNNNNNNNNNNNNNNNNNNNNNNNNNNNNNNNNNNNNNNNNNNNNNNNNNNNNNNNNNNNNNNNNNNNNNNNNNNNNNNNNNNNNNNNNNNNNNNNNNNNNNNNNNNNNNNNNNNNNNNNNNNNNNNNNNNNNNNNNNNNNNNNNGTTGGAATAATGTTCCTTTGGGGCAATGTGGGCCTTATGTGTCAGTGACCTCTACAGAGGCTACCAAGGGGTTAATGTTGATAAGTGGTGCCCTCTAATTTAAGCTTTACGCTGTNNNCAAAGCTTTCATAAGGACCCTCAACCTTCAGGATAAAGATAGAAATCGTCCAATGTATTTGCATTNAATCTTGGCTGCAAGTAGCAAGGACAAGTGATGGACGGGGGACATGTATGTACGCATTTATGGTCAACTAAACTTTTGAGCAGAGTAAAAGAATGTTTTGGGGCTAACCAACNCACCTCTGTTTATAGCCAAGAGGCTAATGTTCCCCTCCCCTCCCCCTCCCCCTTTTTAGAAGCCGAAGCATAGTATACCAAGTGTGCGAGCAGAGGAAGTGTAATGATTTCATCCACTTCTTAGTATCTGTGTTCTTTCTCTTTTTAGTTTCCTTTGGGAACATGTGATGTGTATTGATGTGGACAAAGAAGTTGCCCACCTTCACTGCCACTGCTCTTCTGGTGAGTGTCTCCTCTCTNTTGANTTCGCACACACACACACACACACACACACACACNCACANATNNNNNNNNNNNNNNNNNNNNNNNNNNNNNNNNNNNNNNNNNNNNNNNNNNNNNNNNNNNNNNNNNNNNNNNNNNNNNNNNNNNNNNNNNNNNNNNNNNNNNNNNNNNNNNNNNNNNNNNNNNNNNNNNNNNNNNNNNNNNNNNNNNNNNNNNNNNNNNNNNNNNNNNNNNNNNNNNNNNNNNNNNNNNNNNNNNNNNNNNNNNNNNNNNNNNNNNNNNNNNNNNNNNNNNNNNNNNNNNNNNNNNNNNNNNNNNNNNNNNNNNNNNNNNNNNNNNNNNNNNNNNNNNNNNNNNNNNNNNNNNNNNNNNNNNNNNNNNNNNNNNNNNNNNNNNNNNNNNNNNNNNNNNNNNNNNNNNNNNNNNNNNNNNNNNNNNNNNNNNNNNNNNNNNNNNNNNNNNNNNNNNNNNNNNNNNNNNNNNNNNNNNNNNNNNNNNNNNNNNNNNNNNNNNNNNNNNNNNNNNNNNNNNNNNNNNNNNNNNNNNNNNNNNNNNNNNNNNNNNNNNNNNNNNNNNNNNNNNNNNNNNNNNNNNNNNNNNNNNNNNNNNNNNNNNNNNNNNNNNNNNNNNNNNNNNNNNNNNNNNNNNNNNNNNNNNNNNNNNNNNNNNNNNNNNNNNNNNNNNNNNNNNNNNNNNNNNNNNNNNNNNNNNNNNNNNNNNNNNNNNNNNNNNNNNNNNNNNNNNNNNNNNNNNNNNNNNNNNNNNNNNNNNNNNNNNNNNNNNNNNNNNNNNNNNNNNNNNNNNNNNNNNNNNNNNNNNNNNNNNNNNNNNNNNNNNNNNNNNNNNNNNNNNNNNNNNNNNNNNNNNNNNNNNNNNNNNNNNNNNNNNNNNNNNNNNNNNNNNNNNNNNNNNNNNNNNNNNNNNNNNNNNNNNNNNNNNNNNNNNNNNNNNNNNNNNNNNNNNNNNNNNNNNNNNNNNNNNNNNNNNNNNNNNNNNNNNNNNNNNNNNNNNNNNNNNNNNNNNNNNNNNNNNNNNNNNNNNNNNNNNNNNNNNNNNNNNNNNNNNNNNNNNNNNNNNNNNNNNNNNNNNNNNNNNNNNNNNNNNNNNNNNNNNNNNNNNNNNNNNNNNNNNNNNNNNNNNNNNNNNNNNNNNNNNNNNNNNNNNNNNNNNNNNNNNNNNNNNNNNNNNNNNNNNNNNNNNNNNNNNNNNNNNNNNNNNNNNNNNNNNNNNNNNNNNNNNNNNNNNNNNNNNNNNNNNNNNNNNNNNNNNNNNNNNNNNNNNNNNNNNNNNNNNNNNNNNNNNNNNNNNNNNNNNNNNNNNNNNNNNNNNNNNNNNNNNNNNNNNNNNNNNNNNNNNNNNNNNNNNNNNNNNNNNNNNNNNNNNNNNNNNNNNNNNNNNNNNNNNNNNNNNNNNNNNNNNNNNNNNNNNNNNNNNNNNNNNNNNNNNNNNNNNNNNNNNNNNNNNNNNNNNNNNNNNNNNNNNNNNNNNNNNNNNNNNNNNNNNNNNNNNNNNNNNNNNNNNNNNNNNNNNNNNNNNNNNNNNNNNNNNNNNNNNNNNNNNNNNNNNNNNNNNNNNNNNNNNNNNNNNNNNNNNNNNNNNNNNNNNNNNNNNNNNNNNNNNNNNNNNNNNNNNNNNNNNNNNNNNNNNNNNNNNNNNNNNNNNNNNNNNNNNNNNNNNNNNNNNNNNNNNNNNNNNNNNNNNNNNNNNNNNNNNNNNNNNNNNNNNNNNNNNNNNNNNNNNNNNNNNNNNNNNNNNNNNNNNNNNNNNNNNNNNNNNNNNNNNNNNNNNNNNNNNNNNNNNNNNNNNNNNNNNNNNNNNNNNNNNNNNNNNNNNNNNNNNNNNNNNNNNNNNNNNNNNNNNNNNNNNNNNNNNNNNNNNNNNNNNNNNNNNNNNNNNNNNNNNNNCCCCCCTTTTCCCTTTTTTCCCTCCTCCTTCACCTCCTCCTTCACCTCCTCCTTCCCCTCCTNCTCCTCCTCCTTCCCCTCCTCCTTCCCCTCCTCCTTCTCCTCCTCTTTTCCCCTCCTCCTTCCCCCCTCCTCCCTCCTCCTCCCTTACCCCATTGTGCANNNNNACANGAGGGTGTGCCCACGGAGGAGGCCTGGGGCCACGTTCAGTCCTTGTACCAGAGTCCTGGAAGCTCTGTGGTGTTTGGAAAGAACAATGTCTACATCCAGCCAGTAAGCCTGCTCACAATCTTTGAGTGCCTCTCCGAATTTATATGAAATATTCACACATCCACACACACATCACACATACATATACACACATACATGCATGCACATGTACACACCTAATATATAACATGCACATATTTACACACACANANACACACACACACACACACCACACATCCATACAACCACACATGCATACAATACCACACATCCACACACACCACACATTCACACACACACACACACCACATACACACACTCACATTTCACCTTAGCCTGGCTTTATTGCCCCATTGCCAGGATACCTGAGCTTACACCAGGTGCATGCTTCCATCCTTCTGAAATGGCTACCCAACTCTGTTCTGGAAAACAACAAAGCCAATAAAAGGTGACAAAGGTAGTGGGAGGTGGGCGTCATTTAGAAGGAGTGAGATTCAGGGTTTCAGTGATAGTGCAGTTTATTTTAGTCAATGTCTTACAGTTGGGCATTAGGTTTTTTTTTTGGGNNNNNNNNNNNNNNNNNNNNNNNNNNNNNNNNNNNNNNNNNNNNNNNNNNNNNNNNNNNNNNNNNNNNNNNNNNNNNNNNNNNNNNNNNNNNNNNNNNNNNNNNNNNNNNNNNNNNNNNNNNNNNNNNNNNNNNNNNNNNNNNNNNNNNNNNNNNNNNNNNNNNNNNNNNNNNNNNNNNNNNNNNNNNNNNNNNNNNNNNNNNNNNNNNNNNNNNNNNNNNNNNNNNNNNNNNNNNNNNNNNNNNNNNNNNNNNNNNNNNNNNNNNNNNNNNNNNNNNNNNNNNNNNNNNNNNNNNNNNNNNNNNNNNNNNNNNNNNNNNNNNNNNNNNNNNNNNNNNNNNNNNNNNNNNNNNNNNNNNNNNNNNNNNNNNNNNNNNNNNNNNNNNNNNNNNNNNNNNNNNNNNNNNNNNNNNNNNNNNNNNNNNNNNNNNNNNNNNNNNNNNNNNNNNNNNNNNNNNNNNNNNNNNNNNNNNNNNNNNNNNNNNNNNNNNNNNNNNNNNNNNNNNNNNNNNNNNNNNNNNNNNNNNNNNNNNNNNNNNNNNNNNNNNNNNNNNNNNNNNNNNNNNNNNNNNNNNNNNNNNNNNNNNNNNNNNNNNNNNNNNNNNNNNNNNNNNNNNNNNNNNNNNNNNNNNNNNNNNNNNNNNNNNNNNNNNNNNNNNNNNNNNNNNNNNNNNNNNNNNNNNNNNNNNNNNNNNNNNNNNNNNNNNNNNNNNNNNNNNNNNNNNNNNNNNNNNNNNNNNNNNNNNNNNNNNNNNNNNNNNNNNNNNNNNNNNNNNNNNNNNNNNNNNNNNNNNNNNNNNNNNNNNNNNNNNNNNNNNNNNNNNNNNNNNNNNNNNNNNNNNNNNNNNNNNNNNNNNNNNNNNNNNNNNNNNNNNNNNNNNNNNNNNNNNNNNNNNNNNNNNNNNNNNNNNNNNNNNNNNNNNNNNNNNNNNNNNNNNNNNNNNNNNNNNNNNNNNNNNNNNNNNNNNNNNNNNNNNNNNNNNNNNNNNNNNNNNNNNNNNNNNNNNNNNNNNNNNNNNNNNNNNNNNNNNNNNNNNNNNNNNNNNNNNNNNNNNNNNNNNNNNNNNNNNNNNNNNNNNNNNNNNNNNNNNNNNNNNNNNNNNNNNNNNNNNNNNNNNNNNNNNNNNNNNNNNNNNNNNNNNNNNNNNNNNNNNNNNNNNNNNNNNNNNNNNNNNNNNNNNNNNNNNNNNNNNNNNNNNNNNNNNNNNNNNNNNNNNNNNNNNNNNNNNNNNNNNNNNNNNNNNNNNNNNNNNNNNNNNNNNNNNNNNNNNNNNNNNNNNNNNNNNNNNNNNNNNNNNNNNNNNNNNNNNNNNNNNNNNNNNNNNNNNNNNNNNNNNNNNNNNNNNNNNNNNNNNNNNNNNNNNNNNNNNNNNNNNNNNNNNNNNNNNNNNNNNNNNNNNNNNNNNNNNNNNNNNNNNNNNNNNNNNNNNNNNNNNNNNNNNNNNNNNNNNNNNNNNNNNNNNNNNNNNNNNNNNNNNNNNNNNNNNNNNNNNNNNNNNNNNNNNNNNNNNNNNNNNNNNNNNNNNNNNNNNNNNNNNNNNNNNNNNNNNNNNNNNNNNNNNNNNNNNNNNNNNNNNNNNNNNNNNNNNNNNNNNNNNNNNNNNNNNNNNNNNNNNNNNNNNNNNNNNNNNNNNNNNNNNNNNNNNNNNNNNNNNNNNNNNNNNNNNNNNNNNNNNNNNNNNNNNNNNNNNNNNNNNNNNNNNNNNNNNNNNNNNNNNNNNNNNNNNNNNNNNNNNNNNNNNNNNNNNNNNNNNNNNNNNNNNNNNNNNNNNNNNNNNNNNNNNNNNNNNNNNNNNNNNNNNNNNNNNNNNNNNNNNNNNNNNNNNNNNNNNNNNNNNNNNNNNNNNNNNNNNNNNNNNNNNNNNNNNNNNNNNNNNNNNNNNNNNNNNNNNNNNNNNNNNNNNNNNNNNNNNNNNNNNNNNNNNNNNNNNNNNNNNNNNNNNNNNNNNNNNNNNNNNNNNNNNNNNNNNNNNNNNNNNNNNNNNNNNNNNNNNNNNNNNNNNNNNNNNNNNNNNNNNNNNNNNNNNNNNNNNNNNNNNNNNNNNNNNNNNNNNNNNNNNNNNNNNNNNNNNNNNNNNNNNNNNNNNNNNNNNNNNNNNNNNNNNNNNNNNNNNNNNNNNNNNNNNNNNNNNNNNNNNNNNNGTGTGTGTGTGTGTGTGTGTGCGTGTGTGTGTGTGTACNTNTNTCNCNNNNGTTGGATGTACAACTGACACAATGTTCTACGCTAGATGGACCTTGCTCCCTTCGGTTCAGTCGGCTCAAATCCATAGACAGCTCATTCATATCACCTTCTCCCATGGAGATTGTCAAGCGTCACACACCGTAAGTCGGTGTGGTGAGTGTGTGTGCATCTGTGCGTGTGTTTTAGTGTGCATAGCATGTAATGCACACACAGATGCACNCACACATGCACNCACNCACANNTGCACACANNCACATGTGCACACATGCACACACATGTGTGCATGTGCTGCAGGACTTTGCTGGTCATGTTCATCACTTTGTATGCATTCTTNTTCTTCAGTGCCATGGCAGCCATCTATGTGAGTCCATCCATGTCGTCTCAGTCTGTGTATTCCTCCTCCCTTTCTCTAACCTCTCCTCTTCCTTCTCCTTCTCCCTCCTCCCTTTCTTTACCCTCTCCTCTTCCCTCTCCTCTTCCCTCTCCTNTCCTCTNCCCTCTCCTCTTCCCTCTCCTCCTCCTCCNNNNNNNNNNNNNNTCCTCCCTTTCTCTACCCTCTCCTCTTCCCTCTCCTCCTCCTCCTCCTCCCTTTNTCTACCCTCTTCCTCCTCTTTTTTACGATGGTTCATTTCAGCATTTGATAGTANCTGTGTGTTTATGGCTTCTTCCAGCGTGTAGATTTCAAATAAGGCCCCAAGTGTCATCTGTTGTGACACATCACACAATACACCTCATGAGTGTTCAAGCTGCTGTGTACTCACACTTCTGCACTGTTGTCTTTAACTGTTGCAACTCATCCCACCCCTTGTTTCTTTTGCACACTGCCTTTTGTTTTTCTTGCATCTGCACTCCGCTGGCCATGCAGGATGCTTTGTTGTAATATTCCTTGTGACTTGACAGAATATTGATCTCAATACCACATTCCATCTCCTTCTCCTCCCCTCCCCTCTTCCTACTCACCTCNTCCTTCCCTCCTCCTCCTTCCCCTCCTCCTCCTTCCCCTCCTCCTNCNCNNNNNNNCCTCCTTCCCCTCCTCCTCCTTCCCCTCCTCCTCCTTCCCCTCCTCCTNNNNNNNNNNNNNNNNNNNNNNNNNNNNNNNNNNNNNNNNNNNNNNNNNNNNNNNNNNNNNNNNNNNNNNNNNNNNNNNNNNNNNNNNNNNNNNNNNNNNNNNNNNNNNNNNNNNNNNNNNNNNNNNNNNNNNNNNNNNNNNNNNNNNNNNNNNNNNNNNNNNNNNNNNNNNNNNNNNNNNNNNNNNNNNNNNNNNNNNNNNNNNNNNNNNNNNNNNNNNNNNNNNNNNNNNNNNNNNNNNNNNNNNNNNNNNNNNNNNNNNNNNNNNNNNNNNNNNNNNNNNNNNNNNNNNNNNNNNNNNNNNNNNNNNNNNNNNNNNNNNNNNNNNNNNNNNNNNNNNNNNNNNNNNNNNNNNNNNNNNNNNNNNNNNNNNNNNNNNNNNNNNNNNNNNNNNNNNNNNNNNNNNNNNNNNNNNNNNNNNNNNNNNNNNNNNNNNNNNNNNNNNNNNNNNNNNNNNNNNNNNNNNNNNNNNNNNNNNNNNNNNNNNNNNNNNNNNNNNNNNNNNNNNNNNNNNNNNNNNNNNNNNNNNNNNNNNNNNNNNNNNNNNNNNNNNNNNNNNNNNNNNNNNNNNNNNNNNNNNNNNNNNNNNNNNNNNNNNNNNNNNNNNNNNNNNNNNNNNNNNNNNNNNNNNNNNNNNNNNNNNNNNNNNNNNNNNNNNNNNNNNNNNNNNNNNNNNNNNNNNNNNNNNNNNNNNNNNNNNNNNNNNNNNNNNNNNNNNNNNNNNNNNNNNNNNNNNNNNNNNNNNNNNNNNNNNNNNNNNNNNNNNNNNNNNNNNNNNNNNNNNNNNNNNNNNNNNNNNNNNNNNNNNNNNNNNNNNNNNNNNNNNNNNNNNNNNNNNNNNNNNNNNNNNNNNNNNNNNNNNNNNNNNNNNNNNNNNNNNNNNNNNNNNNNNNNNNNNNNNNNNNNNNNNNNNNNNNNNNNNNNNNNNNNNNNNNNNNNNNNNNNNNNNNNNNNNNNNNNNNNNNNNNNNNNNNNNNNNNNNNNNNNNNNNNNNNNNNNNNNNNNNNNNNNNNNNNNNNNNNNNNNNNNNNNNNNNNNNNNNNNNNNNNNNNNNNNNNNNNNNNNNNNNNNNNNNNNNNNNNNNNNNNNNNNNNNNNNNNNNNNNNNNNNNNNNNNNNNNNNNNNNNNNNNNNNNNNNNNNNNNNNNNNNNNNNNNNNNNNNNNNNNNNNNNNNNNNNNNNNNNNNNNNNNNNNNNNNNNNNNNNNNNNNNNNNNNNNNNNNNNNNNNNNNNNNNNNNNNNNNNNNNNNNNNNNNNNNNNNNNNNNNNNNNNNNNNNNNNNNNNNNNNNNNNNNNNNNNNNNNNNNNNNNNNNNNNNNNNNNNNNNNNNNNNNNNNNNNNNNNNNNNNNNNNNNNNNNNNNNNNNNNNNNNNNNNNNNNNNNNNNNNNNNNNNNNNNNNNNNNNNNNNNNNNNNNNNNNNNNNNNNNNNNNNNNNNNNNNNNNNNNNNNNNNNNNNNNNNNNNNNNNNNNNNNNNNNNNNNNNNNNNNNNNNNNNNNNNNNNNNNNNNNNNNNNNNNNNNNNNNNNNNNNNNNNNNNNNNNNNNNNNNNNNNNNNNNNNNNNNNNNNNNNNNNNNNNNNNNNNNNNNNNNNNNNNNNNNNNNNNNNNNNNNNNNNNNNNNNNNNNNNNNNNNNNNNNNNNNNNNNNNNNNNNNNNNNNNNNNNNNNNNNNNNNNNNNNNNNNNNNNNNNNNNNNNNNNNNNNNNNNNNNNNNNNNNNNNNNNNNNNNNNNNNNNNNNNNNNNNNNNNNNNNNNNNNNNNNNNNNNNNNNNNNNNNNNNNNNNNNNNNNNNNNNNNNNNNNNNNNNNNNNNNNNNNNNNNNNNNNNNNNNNNNNNNNNNNNNNNNNNNNNNNNNNNNNNNNNNNNNNNNNNNNNNNNNNNNNNNNNNNNNNNNNNNNNNNNNNNNNNNNNNNNNNNNNNNNNNNNNNNNNNNNNNNNNNNNNNNNNNNNNNNNNNNNNNNNNNNNNNNNNNNNNNNNNNNNNNNNNNNNNNNNNNNNNNNNNNNNNNNNNNNNNNNNNNNNNNNNNNNNNNNNNNNNNNNNNNNNNNNNNNNNNNNNNNNNNNNNNNNNNNNNNNNNNNNNNNNNNNNNNNNNNNNNNNNNNNNNNNNNNNNNNNNNNNNNNNNNNNNNNNNNNNNNNNNNNNNNNNNNNNNNNNNNNNNNNNNNNNNNNNNNNNNNNNNNNNNNNNNNNNNNNNNNNNNNNNNNNNNNNNNNNNNNNNNNNNNNNNNNNNNNNNNNNNNNNNNNNNNNNNNNNNNNNNNNNNNNNNNNNNNNNNNNNNNNNNNNNNNNNNNNNNNNNNNNNNNNNNNNNNNNNNNNNNNNNNNNNNNNNNNNNNNNNNNNNNNNNNNNNNNNNNNNNNNNNNNNNNNNNNNNNNNNNNNNNNNNNNNNNNNNNNNNNNNNNNNNNNNNNNNNNNNNNNNNNNNNNNNNNNNNNNNNNNNNNNNNNNNNNNNNNNNNNNNNNNNNNNNNNNNNNNNNNNNNNNNNNNNNNNNNNNNNNNNNNNNNNNNNNNNNNNNNNNNNNNNNNNNNNNNNNNNNNNNNNNNNNNNNNNNNNNNNNNNNNNNNNNNNNNNNNNNNNNNNNNNNNNNNNNNNNNNNNNNNNNNNNNNNNNNNNNNNNNNNNNNNNNNNNNNNNNNNNNNNNNNNNNNNNNNNNNNNNNNNNNNNNNNNNNNNNNNNNNNNNNNNNNNNNNNNNNNNNNNNNNNNNNNNNNNNNNNNNNNNNNNNNNNNNNNNNNNNNNNNNNNNNNNNNNNNNNNNNNNNNNNNNNNNNNNNNNNNNNNNNNNNNNNNNNNNNNNNNNNNNNNNNNNNNNNNNNNNNNNNNNNNNNNNNNNNNNNNNNNNNNNNNNNNNNNNNNNNNNNNNNNNNNNNNNNNNNNNNNNNNNNNNNNNNNNNNNNNNNNNNNNNNNNNNNNNNNNNNNNNNNNNNNNNNNNNNNNNNNNNNNNNNNNNNNNNNNNNNNNNNNNNNNNNNNNNNNNNNNNNNNNNNNNNNNNNNNNNNNNNNNNNNNNNNNNNNNNNNNNNNNNNNNNNNNNNNNNNNNNNNNNNNNNNNNNNNNNNNNNNNNNNNNNNNNNNNNNNNNNNNNNNNNNNNNNNNNNNNNNNNNNNNNNNNNNNNNNNNNNNNNNNNNNNNNNNNNNNNNNNNNNNNNNNNNNNNNNNNNNNNNNNNNNNNNNNNNNNNNNNNNNNNNNNNNNNNNNNNNNNNNNNNNNNNNNNNNNNNNNNNNNNNNNNNNNNNNNNNNNNNNNNNNNNNNNNNNNNNNNNNNNNNNNNNNNNNNNNNNNNNNNNNNNNNNNNNNNNNNNNNNNNNNNNNNNNNNNNNNNNNNNNNNNNNNNNNNNNNNNNNNNNNNNNNNNNNNNNNNNNNNNNNNNNNNNNNNNNNNNNNNNNNNNNNNNNNNNNNNNNNNNNNNNNNNNNNNNNNNNNNNNNNNNNNNNNNNNNNNNNNNNNNNNNNNNNNNNNNNNNNNNNNNNNNNNNNNNNNNNNNNNNNNNNNNNNNNNNNNNNNNNNNNNNNNNNNNNNNNNNNNNNGACGAGGCGAGAGCGATCGACGCCCCTGCGATCGGATTCCCTGCGACATCGAGAACAAACCCTTTAACCCCTCTGTGTACTTCGCGCATGAATGCGTACATGGCGTCCTTGTTGGCATTCCATATGCCCTCCAGTTGGGACGCCGGTGGGTATTTGGTGCAGCCTTGCTCCACCGTTATCTCGTAGCAGTTGCTGTGCAAGTAGTTGTAGTCCTGCATCCCTCCGCTCACGCTGTACCACGCTGCGCCGTTGGTGATTCCCTCCTTGAAGCCCGCCGTATCCAACGGGCAAGGGAAGCCGCGCGACATGGTNNCGTGNGCNTACGAGTAGGCGCGGGCCAGTTGCACGAAGATGCTGTCGTCTTCCGACGGAGTGTACAGGGAAAGCCCGTTCTGGCTATCGTCGTAAGGGTAGTTGGCCACCAGGGCGCCATTGTGGAAGTTAGCCGAGAGGACGAAAGGNTAGGTCTCGAGCCAGCGCATGACCGCCTCGGTTTCTGGTTCCACCTTACCCGCCGTCACGTCAAAGCGGTCCGGAAAGTTACGGTTGAGGTCTATGCCGTGAGCGTTGCTGCGTCCCAAAAGGTAGGTGTCATCCGTCTCGGTCGCGATTCGGTACCCGTCTGGGTTCATGGTGGGCAGGATGTGGATGCGGGTGGTGTCCACGAGCTTCGTCACGTCGCTGTCCACGCCGTAGCGGTCACACAAGTCGCGCATGAAGTGGAGCAGGGTCTCCCGCCCTGTTACTTCGTTCCCATGCATGTTCCCGACGTACTTGAACTCCGGCTCTCCAGGCTCGTGAACGCCAGGGTTGTCACTCATCTCAATCACAAAGAGGTCGACATTTTGGACAGACTTCCCGATGGAATAAATTCGAGCGATGGACGGACAGTCGTTGGCCACTTCCAACATGGTGTTGTACAGGGCGTCATTATCGTGGTGGACAAAGTCGACGGCGTGGGTCTTTCGAAAGGCGGGAGCAGTATTCGAGACAGTCAAGGCCGCGAAAAGTATCCACGCCACTTCGAATAGGCGCTTCGGAAGCGTCATCATATCGGGGCCTGGAAACGGATAGAATGAGGAAATAAGGGGACGATTCAATGGTTAATTCACATTTAACAATACAGCTCTAATTATAACTATGTAGCCGGATGTCAAATTGTATTTCGTCGCGCGCTCTGAGCTCTGTATGTATGATTTTCCCAAAAGTAAATTATGGAGCGTACTTCTTGTGAATCGCTTGTCAATCGATGCAAACAAGAGGTAAGATATATTGTTTTTGCAAGATTTAGTATACTGTATGTATGTTTCGTGCTTGTGCGTCCGCTTTCATGTAATGCATGGTGGAAGAAAGTGTCCTTTGTCTCTTCATCTTCCTTACCCCCTCCTCCCTCCTCGTCTTCCTTTTATTCCTTCTTTCCTTGTGTCCTTCGTTCTCCAGTCCAAACAAATCCTGGAAGCCGCATCAGCCTCAAAGATCCACTGGAGTCACCCAAAGTTTACTACCTTCTGCGGTGAGTGGACCGTCGCACGTGCGTTGCATGTGCACCGGCTAATGACGTGCCTATTCGTACCTGCGTACGGGNNGTGTNGCGTTTGCGCATTGGCTGTACNTACGTGCTTACCTCGGGCATTTGTGGTTATGTGGTATTTATGAATATAAAACTTTGTCTTCGCTGCTCCGAATTCACTGTCACACAGTCATGACACTGTTGTATTTTGTAATGGNACCATTGTTGTTGCAACACAATCTGGTGCAGTGTCTCCATGCAGCTTTGGTGGAGGAGATTCTCTCGTACGGGACAAAGAAGAAGTACCAGGTGTCCCGTTCCAAGCCACTCCCACCTTCCGGGAAGATCCTGCATGACCTTGGCAAGAAGGTGAAGGAAGCTGAGGAGGTGTATGGCCTCAGTTTTGCTCAGCACACCAATGCTGGGCGTCCGGACTATGCGTGCATGCAGGGCAGTGCCATCACAGAGAGGAGGATTGTTTGGGTGAGGGTTGCCATGGTGGAGGGAAAGTTGAAGACTATCGTGGAAGCCATGGCAACCAATGCGAGGTGAATAATAAATTGATGGTGGTAGGGNNGGAGGCAGGTGNGAGGGACTATGGAGGGAGGCAGGTGGGAGGGACTATGGAGGGAGGCAGGTGTGAGGGACTATGGAGGGACCATGGAGGGAGGCAGGNNNNNNNNNNNNNNNNNNNNNNNNNNNNNNNNNNNNNNNNNNNNNNNNNNNNNNNNNNNNNNNNNNNNNNNNNNNNNNNNNNNNNNNNNNNNNNNNNNNNNNNNNNNNNNNNNNNNNNNNNNNNNNNNNNNNNNNNNNNNNNNNNNNNNNNNNNNNNNNNNNNNNNNNNNNNNNNNNNNNNNNNNNNNNNNNNNNNNNNNNNNNNNNNNNNNNNNNNNNNNNNNNNNNNNNNNNNNNNNNNNNNNNNNNNNNNNNNNNNNNNNNNNNNNNNNNNNNNNNNNNNNNNNNNNNNNNNNNNNNNNNNNNNNNNNNNNNNNNNNNNNNNNNNNNNNNNNNNNNNNNNNNNNNNNNNNNNNNNNNNNNNNNNNNNNNNNNNNNNNNNNNNNNNNNNNNNNNNNNNNNNNNNNNNNNNNNNNNNNNNNNNNNNNNNNNNNNNNNNNNNNNNNNNNNNNNNNNNNNNNNNNNNNNNNNNNNNNNNNNNNNNNNNNNNNNNNNNNNNNNNNNNNNNNNNNNNNNNNNNNNNNNNNNNNNNNNNNNNNNNNNNNNNNNNNNNNNNNNNNNNNNNNNNNNNNNNNNNNNNNNNNNNNNNNNNNNNNNNNNNNNNNNNNNNNNNNNNNNNNNNNNNNNNNNNNNNNNNNNNNNNNNNNNNNNNNNNNNNNNNNNNNNNNNNNNNNNNNNNNNNNNNNNNNNNNNNNNNNNNNNNNNNNNNNNNNNNNNNNNNNNNNNNNNNNNNNNNNNNNNNNNNNNNNNNNNNNNNNNNNNNNNNNNNNNNNNNNNNNNNNNNNNNNNNNNNNNNNNNNNNNNNNNNNNNNNNNNNNNNNNNNNNNNNNNNNNNNNNNNNNNNNNNNNNNNNNNNNNNNNNNNNNNNNNNNNNNNNNNNNNNNNNNNNNNNNNNNNNNNNNNNNNNNNNNNNNNNNNNNNNNNNNNNNNNNNNNNNNNNNNNNNNNNNNNNNNNNNNNNNNNNNNNNNNNNNNNNNNNNNNNNNNNNNNNNNNNNNNNNNNNNNNNNNNNNNNNNNNNNNNNNNNNNNNNNNNNNNNNNNNNNNNNNNNNNNNNNNNNNNNNNNNNNNNNNNNNNNNNNNNNNNNNNNNNNNNNNNNNNNNNNNNNNNNNNNNNNNNNNNNNNNNNNNNNNNNNNNNNNNNNNNNNNNNNNNNNNNNNNNNNNNNNNNNNNNNNNNNNNNNNNNNNNNNNNNNNNNNNNNNNNNNNNNNNNNNNNNNNNNNNNNNNNNNNNNNNNNNNNNNNNNNNNNNNNNNNNNNNNNNNNNNNNNNNNNNNNNNNNNNNNNNNNNNNNNNNNNNNNNNNNNNNNNNNNNNNNNNNNNNNNNNNNNNNNNNNNNNNNNNNNNNNNNNNNNNNNNNNNNNNNNNNNNNNNNNNNNNNNNNNNNNNNNNNNNNNNNNNNNNNNNNNNNNNNNNNNNNNNNNNNNNNNNNNNNNNNNNNNNNNNNNNNNNNNNNNNNNNNNNNNNNNNNNNNNNNNNNNNNNNNNNNNNNNNNNNNNNNNNNNNNNNNNNNNNNNNNNNNNNNNNNNNNNNNNNNNNNNNNNNNNNNNNNNNNNNNNNNNNNNNNNNNNNNNNNNNNNNNNNNNNNNNNNNNNNNNNNNNNNNNNNNNNNNNNNNNNNNNNNNNNNNNNNNNNNNNNNNNNNNNNNNNNNNNNNNNNNNNNNNNNNNNNNNNNNNNNNNNNNNNNNNNNNNNNNNNNNNNNNNNNNNNNNNNNNNNNNNNNNNNNNNNNNNNNNNNNNNNNNNNNNNNNNNNNNNNNNNNNNNNNNNNNNNNNNNNNNNNNNNNNNNNNNNNNNNNNNNNNNNNNNNNNNNNNNNNNNNNNNNNNNNNNNNNNNNNNNNNNNNNNNNNNNNNNNNNNNNNNNNNNNNNNNNNNNNNNNNNNNNNNNNNNNNNNNNNNNNNNNNNNNNNNNNNNNNNNNNNNNNNNNNNNNNNNNNNNNNNNNNNNNNNNNNNNNNNNNNNNNNNNNNNNNNNNNNNNNNNNNNNNNNNNNNNNNNNNNNNNNNNNNNNNNNNNNNNNNNNNNNNNNNNNNNNNNNNNNNNNNNNNNNNNNNNNNNNNNNNNNNNNNNNNNNNNNNNNNNNNNNNNNNNNNNNNNNNNNNNNNNNNNNNNNNNNNNNNNNNNNNNNNNNNNNNNNNNNNNNNNNNNNNNNNNNNNNNNNNNNNNNNNNNNNNNNNNNNNNNNNNNNNNNNNNNNNNNNNNNNNNNNNNNNNNNNNNNNNNNNNNNNNNNNNNNNNNNNNNNNNNNNNNNNNNNNNNNNNNNNNNNNNNNNNNNNNNNNNNNNNNNNNNNNNNNNNNNNNNNNNNNNNNNNNNNNNNNNNNNNNNNNNNNNNNNNNNNNNNNNNNNNNNNNNNNNNNNNNNNNNNNNNNNNNNNNNNNNNNNNNNNNNNNNNNNNNNNNNNNNNNNNNNNNNNNNNNNNNNNNNNNNNNNNNNNNNNNNNNNNNNNNNNNNNNNNNNNNNNNNNNNNNNNNNNNNNNNNNNNNNNNNNNNNNNNNNNNNNNNNNNNNNNNNNNNNNNNNNNNNNNNNNNNNNNNNNNNNNNNNNNNNNNNNNNNNNNNNNNNNNNNNNNNNNNNNNNNNNNNNNNNNNNNNNNNNNNNNNNNNNNNNNNNNNNNNNNNNNNNNNNNNNNNNNNNNNNNNNNNNNNNNNNNNNNNNNNNNNNNNNNNNNNNNNNNNNNNNNNNNNNNNNNNNNNNNNNNNNNNNNNNNNNNNNNNNNNNNNNNNNNNNNNNNNNNNNNNNNNNNNNNNNNNNNNNNNNNNNNNNNNNNNNNNNNNNNNNNNNNNNNNNNNNNNNNNNNNNNNNNNNNNNNNNNNNNNNNNNNNNNNNNNNNNNNNNNNNNNNNNNNNNNNNNNNNNNNNNNNNNNNNNNNNNNNNNNNNNNNNNNNNNNNNNNNNNNNNNNNNNNNNNNNNNNNNNNNNNNNNNNNNNNNNNNNNNNNNNNNNNNNNNNNNNNNNNNNNNNNNNNNNNNNNNNNNNNNNNNNNNNNNNNNNNNNNNNNNNNNNNNNNNNNNNNNNNNNNNNNNNNNNNNNNNNNNNNNNNNNNNNNNNNNNNNNNNNNNNNNNNNNNNNNNNNNNNNNNNNNNNNNNNNNNNNNNNNNNNNNNNNNNNNNNNNNNNNNNNNNNNNNNNNNNNNNNNNNNNNNNNNNNNNNNNNNNNNNNNNNNNNNNNNNNNNNNNNNNNNNNNNNNNNNNNNNNNNNNNNNNNNNNNNNNNNNNNNNNNNNNNNNNNNNNNNNNNNNNNNNNNNNNNNNNNNNNNNNNNNNNNNNNNNNNNNNNNNNNNNNNNNNNNNNNNNNNNNNNNNNNNNNNNNNNNNNNNNNNNNNNNNNNNNNNNNNNNNNNNNNNNNNNNNNNNNNNNNAGGTGGGACCTCGCCAAGTACGCCACCAACCTGATCGGCCAGGAGAACGCCAACTTCACGGCTTCGACTGGGTGGCAGCAGAAGTTCCTCCAGCGGCACAACCTTCAGCTCACTCCCAAGGCCTCTGGGGACAGAGTGCCCCTCGTGGAAGACCAGCAGAGCATCCCTACCCCTGTGCAGACAGAGGAAGTGATCACACACGAGAGTGTGGCCTTCGTGGAAAAACCCTACTCGGAGGAGATGGAGGAGCAGTTGATCGAGTGGGTGCGCAACAAGGTAGCCGAGTGCGGAAGCATCGACGTGCAGGCGCTGTGCCAGTACGTTGAAGACCTCACCGCGACCTCCAATCCCTCCTTTGTCGCCACCCTGGGGTGGGCCTTCCGCTTCCTGCACCGCAACAAGCTCCTGCTGGACCCGAAACCCCACGCCCAGAACCTGGACGGAGCCTCGTCCTCCCCAGCAGGGACACCGGGCTCGACGAGGAGACGCCCCCTCGGGAGCCCAGAGTACTCGACCCCGAAGAAGCTGCGCACCAAACTAGAGCAGGCGATGACCATGTCCCCGAGCACTGGTAACCTGTGCGAAGCCCTCCTGGCTCTGTCCANGAGAGCGTGCAGGCAGTGATGGAGGTGGTGGAGAATGCCATGCAGCAGGCCCAGGCGGCCGCAGCAGCTGCTGCAGCCGCCCAGCAGACCCAGCCGATGGAGGAGGAAGGGGTGGTGGACCTTCAGTCGAACACCTCCACCTACTTTGGGAAGCCGGCGAGAGAGTTCACCCCAGAGGAGAAGGAGGAGGTGGTGAGGTACGCCAACGCCACCACCCTGCAGAAGGCAGCGTCTCGGTACGGAGTGGCTGCCCCGACCGTGTGGCGCTGGAGGGTGNNNCNAGAAACTCCACCAGCCCAAGTACACCGCCATGCAGAAGAAGTACATCATCAAGTTTGCCGAGAACAACTCCCTGAAGGAGGCGGGGCAGCGATTCGGGATCACGACCAAGACGATCCAGAACTGGAGGAAGGCCCTCCACGCAGAGGGGCTGNNNNGNGACGAAGGTGCCAACGCATCCCAGGAGCTCCAGGAAGGAATGGACGCAGCCGACATCTCGGGCGGGTCCGACATGGTCAACTACGACGGACAGAACTTCCAGTTCATCGTCGACGGCGGTGAGGTGGTCGACGGCAACACGCGCTGCGCAGGGGAGCAGGGAGGGGAAGGGAGCGTCAGGGCGGACTCTGTGCCGCTGGAGGTGACCAACGAAGTGGACATCGAGAACGTCGGGATGGAATACGACATTGTTTCGTCGGAGGGTCACTCGGCCAAGCCCCGCTGCACCCAGCAGGAGAAGATGCAGATACTACAGTACGCCCTCGACCACTCCATCAAGGAGGCATCTCAAAAGTATGGCATCAGTCCCGGCACCTTGTACTACTGGAAGAAGAACCTTAACCCCTCAGTGACGGGTTCAGGATCGGCCAGCGCGGGAGAGCAGAGCCCCTNNNNNNNCAGCGATGGCGGAGGAGGGGGGGGGACCTACTCCCTGATGACGGAAGGGAGTAGCGCTGGGAGTGGTGGGGGGAACGTGACGTTGCAGTTCTCCGGGGGGCAGCCTGGGGACGGGAGCGCAATCCAGGTGTTGCCCGCCCTAGTGACGTCAGATGCAGTTGTGCTCCCCATATCTGAGGCGCTGTTAGCCAGTGCCGGGACCACTGCGGAAACGGTTCAGGCGCTCTCCCAGACGCTGGCCAGCATGTCGCCCGAGGCCCTCCAGAACCTCCCTGCCGACTTCAATCTGTTGCAGGCCGTCACTAATTTGCTTAGCAATGTGGACACTGCTAATGAGAAGCATAAGTTGAGCTTCTACAAACTCGCGGCTGCTGTCAGTAGGGAACTGGCTTCTCCCACGGATGTTCTGATTCCGTTTAGTGACCAACCCACCTCGGATGCTGAACAAACGGTGCTCACGGAAGCGCACTCAGCCGTGCTCGAGCAGCAGGTAGTGACCAGTGAGGAGGTGGTGTCCACCAATGCTGTGGAAGGGGTGAANNCTTCAGAAGTGGCCAGTCAAGATGTCCAATCAGCAGTGAATATGTGTCATACACAGACAGATTCCACAACTTCTAAAGAACCGTGACTTTATTCTTCATCTTTTTACATCAACCCTTTTTTTTGTGCTAAAACTTTCAAATTCCATACGTGTTTTTTTCCATTATAGTGGTTGTGTACAGACAATTAAAAGTCTATTAATCATGACTATTAACTGCCACTCCCTTTGGCATCACCACTGTTATTAAAGTTATGTTATCACATGACTTAAACTAATCCTACAAAAAATTATATGTACAAGTATGTTGAGAAAAAAAAAAAAGGGAAAGAACACAATGTACAAACAAGCTACGCATGTGTGATTTAAGACTTGCTTGTGGCAATACCATTGGCTGAGTAGATTACATCTTCGGTATCTGACTCCAGGTTGACAGGAAGCTCAGCTGGCGGNTCATCATTATAGGCAGGGGCTTGGACCAGGGGCAGTGAGACTGGGGGGTTGACCATCTTGTAGTTGAAGGCAGGCTTTCTTTCAGTTTGCTCCTCGAGGGGGAGGGGGGCAAAACCTTGCCGCGTGTGCTTGGACTTGATGAGGTGGCAGGTGATGATGACAGAGAAGGTCACTATGGCTCCCAGGAGAAACAGGATCACAACTAGTATCACACCCACTATCACCATGGGAACGACCTGGCTGCCCTTTGAATGTAAAGACGTACCGCTGGCTTTGCTATTGTCACTTGCTGTGGCTGCATCAGAAAGTGCCGTAGTGTGTCCCGCAGCAACGGTTACCATGGTTCCTGTTGACTCTGTTACTGTGGAGGCCGTCCCTGCAGAAGCGTCAGTTGAACTCTGAGCGACCACCATTTTATCCGAATCCAAGGTGAAATTGACGGTGACAGCGGGGCCGTTGCTCACGACTACCGTTTGCTGCCGCTTCACGAACCCCGGGGCCGAAGCACTGATGGTGTAGGTACCGGGGGCGAGGAGCCGCCAAAAATCCCCGTCCTTCGCCGCCCTGACGGGGCGTCCAGACGAnCAGTCACAACAGAAGAAGATCAAAGGACGCTACATAGTTACCTTGTCGTCCGAATCGAGTGACGAGGTGTTGGATCGCACGTTGAGTATTATGAAGTACGCCACGAGCGCAAGTCAACAGCGACTTCGCGCCGATCACATCACCGTCTTCAGGCGAGCAGGGAAGGGTTTTACCGGCACACTGAGCAGCAAGATGGTGGAATTGGTAAGTCCAGGGCCCTCACAACAGGTTGTTTGTTTCTCTTTTCTACGTTTTGATAATTACAATATCCCATTAAGGATACATCACGCTTATATGAACTTTCATAGAGAAGTTTATTTAAAAATAAATACTTACCACATTTTAATATATATAACACATACACACAACACAACAACGACAACAACAACAACAACAAACTTGTGTGTAAATGCATTTCCGTACATTTAATTCAGGAGTAATCAAAAACTTGCAAGTCCAATCGCATACATATACGAGTGTATCATTGCAGCAAGATCATCACATGTATGCACACACACACACACACACACACACACACACANNNNNNNNNNNNNNNNNNNNNNNNNNNNNNNNNNNNNNNNNNNNNNNNNNNNNNNNNNNNNNNNNNNNNNNNNNNNNNNNNNNNNNNNNNNNNNNNNNNNNNNNNNNNNNNNNNNNNNNNNNNNNNNNNNNNNNNNNNNNNNNNNNNNNNNNNNNNNNNNNNNNNNNNNNNNNNNNNNNNNNNNNNNNNNNNNNNNNNNNNNNNNNNNNNNNNNNNNNNNNNNNNNNNNNNNNNNNNNNNNNNNNNNNNNNNNNNNNNNNNNNNNNNNNNNNNNNNNNNNNNNNNNNNNNNNNNNNNNNNNNNNNNNNNNNNNNNNNNNNNNNNNNNNNNNNNNNNNNNNNNNNNNNNNNNNNNNNNNNNNNNNNNNNNNNNNNNNNNNNNNNNNNNNNNNNNNNNNNNNNNNNNNNNNNNNNNNNNNNNNNNNNNNNNNNNNNNNNNNNNNNNNNNNNNNNNNNNNNNNNNNNNNNNNNNNNNNNNNNNNNNNNNNNNNNNNNNNNNNNNNNNNNNNNNNNNNNNNNNNNNNNNNNNNNNNNNNNNNNNNNNNNNNNNNNNNNNNNNNNNNNNNNNNNNNNNNNNNNNNNNNNNNNNNNNNNNNNNNNNNNNNNNNNNNNNNNNNNNNNNNNNNNNNNNNNNNNNNNNNNNNNNNNNNNNNNNNNNNNNNNNNNNNNNNNNNNNNNNNNNNNNNNNNNNNNNNNNNNNNNNNNNNNNNNNNNNNNNNNNNNNNNNNNNNNNNNNNNNNNNNNNNNNNNNNNNNNNNNNNNNNNNNNNNNNNNNNNNNNNNNNNNNNNNNNNNNNNNNNNNNNNNNNNNNNNNNNNNNNNNNANGCATGGACACATGANGGGNANAGAGGACANGCATGGACNGNNNNNNNNNNNNNNNNNNNNNNNNNNNNNNNNNNNNNNNNNNNNNNNNNNNNNNNNNNNNNNNNNNNNNNNNNNNNNNNNNNNNNNNNNNNNNNNNNNNNNNNNNNNNNNNNNNNNNNNNNNNNNNNNNNNNNNNNNNNNCATAAGNAGTGTGCACACAAACAACAACATCCACATAAGTAGTACGNNNNNNNNNNNNNNNNNNNNNNNNNNNNNNNNNNNNNNNNNNNNNNNNNNNNNNNNNNNNNNNNNNNNNNNNNNNNNNNNNNNNNNNNNNNNNNNNNNNNNNNNNNNNNNNNNNNNNNNNNNNNNNNNNNNNNNNNNNNNNNNNNNNNNNNNNNNNNNNNNNNNNNNNNNNNNNNNNNNNNNNNNNNNNNNNNNNNNNNNNNNNNNNNNNNNNNNNNNNNNNNNNNNNNNNNNGTGTGTGAATATCAACACTTCCGTCCGATGTTCCATAGGNNNNNNNNNNNNNNNACACACACACACACACACACACACACACACACACACACATGATGTCTGTACATTTCTCCTCCTCCCTCCCTTCACAGCTCCACAAACATCCACTGGTGACATCTATTGAGGAAGACGTTGCAATCCGAAAATCCTACTCTAGTTTCAGCAGCGAACCTTCTTTGGCCAATGGAATCTCAGGCACTCTCAGTGTCCAGTCCGAGACCGAGACATCCAGCGCAAACTCCGATACCAAGCCTGGTACCGAGACATCCAACGCGAACTCCAATGCCAAGCCTGCTACTAGCAGCCCCGCGGTACCCTGGAACCTGGACCGTCTCGATCAGCACGCAGCCACTCCCCTCGACGGCCAGTACAATCCCGAGGCGACGGGCAAAGGCGTGGACGTATTCATCGTGGACACGGGAATCCGCTACACGCACGAGGATTTGGGCGGGAGGGCCAAATACGCGGGCCTCGACGTCATTGACCAGCTGACTGGGAGCACCCTCAAAGGGCTAGACTGCCAAGGGCACGGGTCCCACGTGGCCGGGGTTGTTGGCGGGAACACGTACGGTGTCGCCAAAGGGGCCAATCTGTACAGCCTCCGCGGACTGGACTGCACTGGTACCGGCGCTGTGAGCGGTGTCGTTACCGCTCTGGAATATATTGCGAACTATCCATCCAGCAACCCCAAAGTGATCTCTCTTTCTCTCGGCGTAGACGCNTNNGNNGNNTAGTGGATAATGGACTGCGAGAGGTGGTCAACAAGCACGGCATCACGGCAGTTGCCGCCGCCGGGAATAACGGTCTCGATTCGTGCAACTTCAGTCCTGCGTCTTCCGGGGTCACTATAGACGTCGGTGCGACCGATAATGGGGACGAGGCAGCGTCCTTTTCCAACACGGGGGCTTGNCTCGATGTTTTCGCGCCCGGCAAGATGATCGACAGCGTTGGGTTCGGGTGCGACACGTGCACCAAGGTCATGTCTGGNACCTCGATGGCCTGCCCTCACGTGACCGGCTACGTTGCCGTGCTGTTGGAACTGCAGCCCACACTGACTCCCACCCAAGTGCTGGAGAACGTTGTGGCTAAGTCAACCAAGGATGCTGTTCGACTCAACTACGTGTCAGACAAAAATACTGCTGCCCGAACTCCTAATCGCCTGCTTTACGTTCCATCTCTTGGCGCCGCNTTCTACTTAATAGAACTAATCATTTTTGGGTGTTTACTAATAATGTCAAAATTGTAATGTAGTAATGTAGGATTATTATGTCCCCGTAAGATGTAAAAGTGTGAGCTCGGAATGCGGAGGTGTAAAGTGCGGTTACACTTTGGAATGCGACGTTTTGTAGTCACGTGAGATTGTTAAGTGACGCATGCGCGTAGTGCGACATTTCTTTCTTTCTAGGTTGTGTTGANNNNNNNNNNGCTGGCTCCTTTTCAGTGGAACAGCGAAAAATAATTTCTTTCTTCTGCAAGACGCTTGTATATCTTTCTACCAGACTAAAAAGCCGTTTGGTCGCAGCTTTCCAACGCACTACAACAGCCGCAAGTGGACACACCGGTCCCAATTGCTGGGCCCAAACTGGTGAGCCGCCATCTTGTTTCCTGTGTGTGATGCAGCTGCCCGTTGTATGCTTGTGTGATACCTTAGGGGGTTGAGCTGGCTCCGATTCTGGTATATAGCGTGCTTTTGAGCGACAGATGCGATCCAAATCGCTAAACACGAGTGGTTGCTGCGTTCAGCAGCTTTTGTAATAAATCGCGCGTTCTGTATTTGTGTGGCTANGCTCCCAAATAGATACACAGCCGGGATGCCGTATTGACTTTTATATTAGATATCAATGCCAGTTAATTTAAAATAAGCAATGGTGTGTGCGTCGTGCTGCAAGCAGGAATGGATGTGGTTTTAGTCACCGCATGGCGGGTTGTGAGCCTTTGAATGTTTGGCTCTATCTAAAATCAATAAATAAATGAGTATGTGTCGTTATTCTTCCTTCATAGCTTCTGAAGCTTATAATTTCCCCGATTGAAGCTGTGGAAAATGCATCGCGGAGTGATTCTAATGGACTCCAGATTCCAGCGAAGGCAAAGGGCGGAAGGCGGAGCCCCAACGGAGGAGTCCGGTTCGGAAGCGAGGAGATTGGGGACAAACCTCCTGGAGGAAAACGTCCCTGAGTCCGTCANNGAANNNNTNCCTCTCGGACGCCGCCCACCACCACACGGCCCACGAGGGGGCCACCCCTGCCGTCTCCGGCATGGCCGTTAGCGGCGGCGGGGGCGCTGGGAACGGCGTGGAGATCGAAGGAGAGGTGGTGGAGGACCTGTCCATGAACCATTCGCAGGTGCAGCAGGAGGGTGACGGGGGAGGAGGAGGAGCGGGGCGCGGTGACGAGGAAGGAGGCGCAGGGGGCTACACTGGCCTCATTGTGGTTGACGACTGTGAACCAACAGATGAGGGGGAGAGCCTCAACATGGTGGGGTCACCGGCCCCAGCCGACGTCATTGGCTTTGCTGAAAAGTACTCGGTCGAGACGGCGGCGAAGAGGTTCAATGTCCCCGTGGCGACGATCCAGAGTTGGGCCAAGTTGCCAGACGAGATCCAGCAGCAGCAGCAGCAGCAGCCAAAGTTCAACTCCCCAGGCCAGGGGAGGAAGATATCGTACTCCAAGGCGACGGACAGTCTGATCGCCGACCACATCAGGGAGCTTCTGGCGAAGGGGGAGAGGGTGACCCTTCAATACCTGTGCAGCTATGCCAAGGCCCACATCCACCAGGAGAACCCCCAGTTCAACGCCTCNCAGGGTTGGGCCCAGCGGTTCCTCCTCCGACACGACATCGACCTCGGGGACCACTGCAAGAAGACCTCCGGCTCGAAGCAGGACGTGAGAAGCTCGGAACGGGGACGACCACTCTCCTACTCGACCGAGACNGATCAAAACATTGCGGAGTGGGTGAGGACAAAGCAGCTGGAGGGGACACTAGTGACCAACTCTGAACTCAGGAAGTACGCCAAGACCCTCATACTCAAGGAGAACCCAAACTTCACGGGTTCGGCCAGCTGGGCCCAGAACTTCCTCCTCAGGCACAAGCTGAGCCTTCACAACCAGTCCTCCGGGGGGNNNNNNNNNNNNNNNNNNNNNNNNNNNNNNNNNNNNNNNNNNNNNNNNNNNNNNNNNNNNNNNNNNNNNNNNNNNNNNNNNNNNNNNNNNNNNNNNNNNNNNNNNNNNNNNNNNNNNNNNNNNNNNNNNNNNNNNNNNNNNNNNNNNNNNNNNNNNNNNNNNNNNNNNNNNNNNNNNNNNNNNNNNNNNNNNNNNNNNNNNNNNNNNNNNNNNNNNNNNNNNNNNNNNNNNNNNNNNNNNNNNNNNNNNNNNNNNNNNNNNNNNNNNNNNNNNNNNNNNNNNNNNNNNNNNNNNNNNNNNNNNNNNNNNNNNNNNNNNNNNNNNNNNNNNNNNNNNNNNNNNNNNNNNNNNNNNNNNNNNNNNNNNNNNNNNNNNNNNNNNNNNNNNNNNNNNNNNNNNNNNNNNNNNNNNNNNNNNNNNNNNNNNNNNNNNNNNNNNNNNNNNNNNNNNNNNNNNNNNNNNNNNNNNNNNNNNNNNNNNNNNNNNNNNNNNNNNNNNNNNNNNNNNNNNNNNNNNNNNNNNNNNNNNNNNNNNNNNNNNNNNNNNNNNNNNNNNNNNNNNNNNNNNNNNNNNNNNNNNNNNNNNNNNNNNNNNNNNNNNNNNNNNNNNNNNNNNNNNNNNNNNNNNNNNNNNNNNNNNNNNNNNNNNNNNNNNNNNNNNNNNNNNNNNNNNNNNNNNNNNNNNNNNNNNNNNNNNNNNNNNNNNNNNNNNNNNNNNNNNNNNNNNNNNNNNNNNNNNNNNNNNNNNNNNNNNNNNNNNNNNNNNNNNNNNNNNNNNNNNNNNNNNNNNNNNNNNNNNNNNNNNNNNNNNNNNNNNNNNNNNNNNNNNNNNNNNNNNNNNNNNNNNNNNNNNNNNNNNNNNNNNNNNNNNNNNNNNNNNNNNNNNNNNNNNNNNNNNNNNNNNNNNNNNNNNNNNNNNNNNNNNNNNNNNNNNNNNNNNNNNNNNNNNNNNNNNNNNNNNNNNNNNNNNNNNNNNNNNNNNNNNNNNNNNNNNNNNNNNNNNNNNNNNNNNNNNNNNNNNNNNNNNNNNNNNNNNNNNNNNNNNNNNNNNNNNNNNNNNNNNNNNNNNNNNNNNNNNNNNNNNNNNNNNNNNNNNNNNNNNNNNNNNNNNNNNNNNNNNNNNNNNNNNNNNNNNNNNNNNNNNNNNNNNNNNNNNNNNNNNNNNNNNNNNNNNNNNNNNNNNNNNNNNNNNNNNNNNNNNNNNNNNNNNNNNNNNNNNNNNNNNNNNNNNNNNNNNNNNNNNNNNNNNNNNNNNNNNNNNNNNNNNNNNNNNNNNNNNNNNNNNNNNNNNNNNNNNNNNNNNNNNNNNNNNNNNNNNNNNNNNNNNNNNNNNNNNNNNNNNNNNNNNNNNNNNNNNNNNNNNNNNNNNNNNNNNNNNNNNNNNNNNNNNNNNNNNNNNNNNNNNNNNNNNNNNNNNNNNNNNNNNNNNNNNNNNNNNNNNNNNNNNNNNNNNNNNNNNNNNNNNNNNNNNNNNNNNNNNNNNNNNNNNNNNNNNNNNNNNNNNNNNNNNNNNNNNNNNNNNNNNNNNNNNNNNNNNNNNNNNNNNNNNNNNNNNNNNNNNNNNNNNNNNNNNNNNNNNNNNNNNNNNNNNNNNNNNNNNNNNNNNNNNNNNNNNNNNNNNNNNNNNNNNNNNNNNNNNNNNNNNNNNNNNNNNNNNNNNNNNNNNNNNNNNNNNNNNNNNNNNNNNNNNNNNNNNNNNNNNNNNNNNNNNNNNNNNNNNNNNNNNNNNNNNNNNNNNNNNNNNNNNNNNNNNNNNNNNNNNNNNNNNNNNNNNNNNNNNNNNNNNNNNNNNNNNNNNNNNNNNNNNNNNNNNNNNNNNNNNNNNNNNNNNNNNNNNNNNNNNNNNNNNNNNNNNNNNNNNNNNNNNNNNNNNNNNNNNNNNNNNNNNNNNNNNNNNNNNNNNNNNNNNNNNNNNNNNNNNNNNNNNNNNNNNNNNNNNNNNNNNNNNNNNNNNNNNNNNNNNNNNNNNNNNNNNNNNNNNNNNNNNNNNNNNNNNNNNNNNNNNNNNNNNNNNNNNNNNNNNNNNNNNNNNNNNNNNNNNNNNNNNNNNNNNNNNNNNNNNNNNNNNNNNNNNNNNNNNNNNNNNNNNNNNNNNNNNNNNNNNNNNNNNNNNNNNNNNNNNNNNNNNNNNNNNNNNNNNNNNNNNNNNNNNNNNNNNNNNNNNNNNNNNNNNNNNNNNNNNNNNNNNNNNNNNNNNNNNNNNNNNNNNNNNNNNNNNNNNNNNNNNNNNNNNNNNNNNNNNNNNNNNNNNNNNNNNNNNNNNNNNNNNNNNNNNNNNNNNNNNNNNNNNNNNNNNNNNNNNNNNNNNNNNNNNNNNNNNNNNNNNNNNNNNNNNNNNNNNNNNNNNNNNNNNNNNNNNNNNNNNNNNNNNNNNNNNNNNNNNNNNNNNNNNNNNNNNNNNNNNNNNNNNNNNNNNNNNNNNNNNNNNNNNNNNNNNNNNNNNNNNNNNNNNNNNNNNNNNNNNNNNNNNNNNNNNNNNNNNNNNNNNNNNNNNNNNNNNNNNNNNNNNNNNNNNNNNNNNNNNNNNNNNNNNNNNNNNNNNNNNNNNNNNNNNNNNNNNNNNNNNNNNNNNNNNNNNNNNNNNNNNNNNNNNNNNNNNNNNNNNNNNNNNNNNNNNNNNNNNNNNNNNNNNNNNNNNNNNNNNNNNNNNNNNNNNNNNNNNNNNNNNNNNNNNNNNNNNNNNNNNNNNNNNNNNNNNNNNNNNNNNNNNNNNNNNNNNNNNNNNNNNNNNNNNNNNNNNNNNNNNNNNNNNNNNNNNNNNNNNNNNNNNNNNNNNNNNNNNNNNNNNNNNNNNNNNNNNNNNNNNNNNNNNNNNNNNNNNNNNNNNNNNNNNNNNNNNNNNNNNNNNNNNNNNNNNNNNNNNNNNNNNNNNNNNNNNNNNNNNNNNNNNNNNNNNNNNNNNNNNNNNNNNNNNNNNNNNNNNNNNNNNNNNNNNNNNNNNNNNNNNNNNNNNNNNNNNNNNNNNNNNNNNNNNNNNNNNNNNNNNNNNNNNNNNNNNNNNNNNNNNNNNNNNNNNNNNNNNNNNNNNNNNNNNNNNNNNNNNNNNNNNNNNNNNNNNNNNNNNNNNNNNNNNNNNNNNNNNNNNNNNNNNNNNNNNNNNNNNNNNNNNNNNNNNNNNNNNNNNNNNNNNNNNNNNNNNNNNNNNNNNNNNNNNNNNNNNNNNNNNNNNNNNNNNNNNNNNNNNNNNNNNNNNNNNNNNNNNNNNNNNNNNNNNNNNNNNNNNNNNNNNNNNNNNNNNNNNNNNNNNNNNNNNNNNNNNNNNNNNNNNNNNNNNNNNNNNNNNNNNNNNNNNNNNNNNNNNNNNNNNNNNNNNNNNNNNNNNNNNNNNNNNNNNNNNNNNNNNNNNNNNNNNNNNNNNNNNNNNNNNNNNNNNNNNNNNNNNNNNNNNNNNNNNNNNNNNNNNNNNNNNNNNNNNNNNNNNNNNNNNNNNNNNNNNNNNNNNNNNNNNNNNNNNNNNNNNNNNNNNNNNNNNNNNNNNNNNNNNNNNNNNNNCCACCACCACTACAATACCACCACCACCACCACTACAATGCCACCACAACCACTACACCATCACTACATGTCACCACCACCACCACTACACACTCTTGTGCTATATCGTATGATGTATCTGCACTCTTAATGGCACTTGTGCTTTATTTATCTCTGAAAAAGAGCATGGTCATATTTGGGGCAACTCGCACAAAGTTTGTACAAGGCACACTAATTGTTAAGTTGTGTTCCTCATCTGATTGGTTAAGTTGTGTTCCTCATCTGATTGGTTATGATTTACACTACTTGTATCTTTTTCAGGGAGATGTTGTAGCAAAGACTGAGAAGAAAAGTTCATTTTTAAAACTCCTGCGAAGAAAGAAGGAAACCTAGACATCTTTTTTTTTGCCTTTCTTTTTAGTCACAAAGTGCTATTTATTCTACTGTGATGTCATCTTTCGTTGGAAAATCTTCCCTGATAAATATGTCCTGTGTATTAAATGTGTTGCGTGCTCGTCTTTCCGTTGTTGTACGTGTGTTTACGCGACTTATTTGCGGATGTAATGAGTATCAATTTTCACTGTGTGATGTGTGCTATCAGAGATAATATATATTTTATCTCTTGCTATCGCGGCGTTATACCAAGTTATGCATATAAAATCCAACTTCGTATTATAGGCTAGTGTATAACAAGCCGTTTTACACAGTGAATTTGTATTTTGTGCATGCAGTNAACATGATGATGANGTGNGGACCCAACTGCNTTATTATTGAGAACGGGAAAATGAGAGCGTTGTACCAGAGAGGTAGATCGTGGTTGTACAAATGAATTCCGTACTGCGTTATGTCTCCCTGGTTGTGGTTACGGAAATATAGCAATTTGCATGAAGNNNCATGCNATAANNGCACAGCATATACAAAGCATGGACAAACAACACACACTTCCATATGTGGGTGTACAAACGCGCGTAGCATAGTTGTTTAATCTTTCGTCACCGCACCGCGCGAGATAATTGTATTACTCACGTACTACAGATGTTTATTCCCGGGTTTCTAGATTGGAACTTTTCTGTTTAAGTCGCCATTCCCGGAAGCTGATTAAAAAAAAATCGAGCAAGGAAGGGCAGATGCACACCCTATTATCCGGCCTGTNNNNCGCGTCATTACTCCTGCTTGTAACTTCGCGGGCCAGTCCCATCGACGAATGGAGCGAAACCGTAGCCAAAGCCACCAGCCACGACCACTGGGAGCACCTTGTGAGAAGATCCGCCAAGAGCGCGGAAGCCGAAGGTATCGAGGACGACACTTCACACATCCAGGACGTCCAGGAGTACTACCGCAACGTTTTCGCTAGCGACAGCAACTGGGACAGCGCGACTCNNTTGCGCCTATCCTCGGCTGCGGAGGTCCACAACTTGCGCATCATGCAGTCCCTCGAAGCTTACCTACAGGCCAAAAACGACCAAGAGATGGCCAAGGTGGAGGCCGCTGACCAGTCCCTGGTGCCCGATCAGTCACAACAGAAGAAGATCAAAGGACGCTACATAGTTACCTTGTCGTCCGAATCGAGTGACGAGGTGTTGGATCGCACGTTGAGTATTATGAAGTACGCCACGAGCGCAAGTCAACAGCGACTTCGCGCCGATCACATCACCGTCTTCAGGCGAGCAGGGAAGGGTTTTACCGGCACACTGAGCAGCAAGATGGTGGAGTTGGTAAGTCCAGGGCCCTCACAACAGGTTGTTTGTTTCTCTTTTCTACGTTTTGATAATTACAATATCCCATTAAGGATACATCACGCTTATATGAACTTTCATAGAGAAGTTTATTTAAAAATAAATACTTACCACATTTTAATATATATAACACACACACACACAACACAACAACGACAACAACAACAACAACAACAAACTTGTGTGTAAATGCATTTCCGTAAATTTAATTCAGGAGTAATCAAAAACTTGCAAGTCCAATCGCATACATATACGAGTGTATCATTGCAGCAAGATCATCACATGTATACATGTATGCACACACACACACACACACACACACA>scaffold327|size63049ACTTACCTCGTGTCCGGCTTCAACACGACAACGGCAAATGCCAACAGGAGATTGAAGGTCATACCGTAGACCCAGATGTTCTCCCAGTCTTCTTTCTGTCTCTTCTCCCCGGGGCGCAGCGGCTGAGGGAGGGCGGGAGAGGCTGGAATAAATGGCAAATGCCTCATCGTATCCTTATCGCATATATGCGCACGTACATATTAGAAGCTTAAAGCGTTTATCAACAGGTCCCTGGAGGAAGAGGTGGGACTCCTCAACGACCACTACAGGAGCATCTTAGAAGAGAATGACTGGAGGACCAGACCACGGAGACGATTGTGGTACCTTTTACGACTAACAAACGGCGGGGGATCTATTTTCTACGCTTCAAAGTTGAAGCCATTTGAAGTGAAAGGTTAGTTGACAGACAGTGCTGCCAAAATATGTGTAGAGTGAAGCCTAACGTTTAGGATTCTCCTTCACGGCCTAGCTGTGCCTAAGTTATGATGATATGCCATACCCTGCATTGAAACTCAACCCCTTTCCGAATTAATGCCCATCTGTGGATTAGGTGTACACTACAACAGAACTGAGCTTCGTACCTTCTCATTGAAAAGAAACCCATTGGGTGGTTCGGGTCCGGTAGTGGGTTCATTGTGCGCGTGTGATGTCGAAGATCCCGAACGGAAAGCAGGCTTCGCTTTGGCGCCTTGCCGGTGAGCTACTCTCGTTAGTCTGGTTGAAAGCAATAATCTCAAAGCCATGGCCAATAATATTTCAACCTCATGCCCAGTCCGCGATCCCCGCTTCAGGCGGATAATTTTAATAAGTAATTTGGGGGCGGAGCTAGAGCCACGTCATGCATGCAAGCACACAGGGACATGAGATGTACGTGTCCATATCTACAACCAATTGTCCTTCAAGTGTACGCTTGATTTCAGCAACATATGACCTAAAAACGATATGTACGTTATCTCTGCTGTACGTTACAGTTCGTGTACTTGAATCACATCAGGCTATCAAATCAAGTTATCTACGGTGGTTGTTCACATGTCTACCCCAGTTGCTATGAATACTAACGAATTACGGATACAGTATTNNNNNNNNNCGTTTGTGTTTATATTAGAAGAGTTATTAATCATGAAGGGAATACTTCCAGTTGCGTCCTTGACTTGAAGCATCTAATGTAGTCATATCACGCAGTATTGATGTGATCAGTCCACATCATTGCCCTAAGGAAACTGGTAGCCATGTTTGGTAAGCAATTCATCACTCTCCTTGGGGCCATGACTGANAGAAACACCACAATTTAAGGTTGGTTACTAAATATGTCCTGCATGTACCTTCCGTATGTATACTTGTAAGGTGTGACATGCTCGTTCTCAATCTGATGAAGAAGAGGTGTAAATATCCTCCAAGCCTCCTTCAGTTCATCGCTGCAACAAGCAGAATGATATTGCTTCATGCACCAAAATNGTCTGACAACTAATACACCACACNATATGTAAAAAGGATTGCTATATATAACAGCCACCATGCCAGTAGCTCACCTCCTTACAAAGCTGGCTTGATCACCACTGAGGATGCTCANTATGACCCTCTCATATGGGTGGGGAAGCAGTAAATCCTAGCAAAATGATCAGCGGCTCAAAGAGGGTCAATTGTGCACCCTGACAACACTTGCTCACCCATCAGTACCTTGAAATGTGTCCTGTAGGTGAGATCAAGCTCACCCTGGATTGGCTCAAATGAGAATCCAGGCTGCTTCATCATTATCTTGAGATACATAGCCTCATCAGGCAGCATTCGTATTACCAGCTCATTGCGGTGGCACTTCCCTTGAAATACATCTCCTGGAATGTCATGAAACTGAACACGAATCTCTCCTCTTTGTTCACCAAGCGCTTTAATTGTTCATAGACAAATGCAAATGAAACTGTGCATGTACAATTATGCCACCACATGGATAAATAATCCCTTCAGTAAATTGCATGTTGCACACATCTCATTTACTCGTCTGTCACAACCCATTTTAAAGTCAAACTATTAACACTTATTCCCACTTCTATGCACAAACGAAAAACTAAATGGTTTTTAAAATATGGTAAATAAGTGCATGGAAGACCTTTGGTTCCTTCATTTATTTCCCCTCACTTCACAACATCTCTATCTCTATAGACCTCCTCTGCTCTCTCAACAGCCAATTCTTGCTTGCTCACACCTCCCTTGTGGCGAGCATTTGGCCTTGACAAACATCATCGTGTATCCACGGGGCACTCCTCCCACTTCACACACGATCACACACAGTACCCAACATTTTGAAGGACGCATGCATCTGATGCTAAATATTGCCTTACCCTTTCCACACTGCAGGATGAATGGAACACCATCCCAACGCTCGTTTTTAATTTGCAATACTACAGTTGTNNNANNNNNGTGCAAGAGATCCATCGGGCACAGATTCATCATCCTGGTAGCCAGCTTTGGCCCCTCCTTCAGCCTGCAGGCTCCCCACGTACTGCCCCAACACAACGTCACTTAACTTGGCTTCTGGAATGCAGCGAAGCAGTTTGACCTGCAACACAACATGCACACACAGATCATTTCTCACTGCATGCATGGAGTGTGCGAGGGAAAAAAAGGCCAATCATATCCTTTACACACTATACAGATACCTTGCATACACACACACACACACACACACACACACACACACACACNNNNNNNNNNNNNNNNNNNNNNNNNNNNNNNNNNNNNNNNNNNNNNNNNNNNNNNNNNNNNNNNNNNNNNNNNNNNNNNNNNNNNNNNNNNNNNNNNNNNNNNNNNNNNNNNNNNNNNNNNNNNNNNNNNNNNNNNNNNNNNNNNNNNNNNNNNNNNNNNNNNNNNNNNNNNNNNNNNNNNNNNNNNNNNNNNNNNNNNNNNNNNNNNNNNNNNNNNNNNNNNNNNNNNNNNNNNNNNNNNNNNNNNNNNNNNNNNNNNNNNNNNNNNNNNNNNNNCACACACACACACACACACACACACACACACACACACACACAAGCCAATAGTTTGAAATTGATGTGGTACAAATCAAAATAACTAGAAAACCTGCATGTGTCGCATAGAGTAGATTGTGCACACACCTTTTCATCCCTAATATCTTTCGCACCTGTGGTTACTGGTTTCTCCATAGCAACCAGGCAGAGCACTTGAAGTAGGTGGTTCTGTACCATATCACGAATGATGCCATATTCATCAAAATATCCTCCTCGACCTTCAGTGCCAAAGGTCTCATTGTAGGTCACGACAACGCATTTGATGCTGTTGCTGTTCCATAGGGACCGGAAGATGTAATTGGCAAACCTGGGCATTTGACATGTTTTTGATAACCACACAGTACATATAATGCTAAGACCTTGCAGAATGAAGAGAACATAAGTGATACAAGCACATATGGATATACAAACAATTGGAGAATGGAGAGGAGGGATATATTGCCTTTCAAAGAGNGNAGGGATATATTGCCTTTCAAAGAGGGAAGGGCTATCACATGTATACATCGCTGGAAGAGACATGCNNTTNTTTTNTTNTTGTTTTTTTGTTTATGACTCCAATTGGCATATAGAACTGGCAGCACTTAATCAGCACACCTGTGCCCATAAGGATCAATCAATCCACACACTTTAATAGTGTGTGAAGGGCAAAGGGGCAAAATAAATGTGACATGGATAATTGCATAGATACCATGAAAACCATTACTTCCACTTTTTGGCCTTACATGTTTTCACACCCAAAGAACCTATGAGCCTTACCTAAGGATCAGTAATGTCTGAATCATCTCATGGCTTAGGAAGTGGTCCACTCTGAAGACCTGATCCTCAGTAAACAGATTACAAATGTGACGATTAAGCTGGGCATAACTCTCCGAATCCTTTCCGAATGGTTTCTCCAGGACAATGCGGTTCCACCCACTACACAGATGGTTACATAGAAAGTGTGTCAGTGTGGCATAATCAATAACTCTTCTCTTACGTTGAACTCATTGCTGTCTCTCTCAAGCAGGATGCGACCTCTTCAAACACAGAAGAAGGAAGAGCGAGGTAGAAGAGCCTGTTGCCCACATCGTAACCATTCTCTAATTTCTTTAGCTCAGCATTCAGCTTTGCAATGGATTCCTTATCTGTGTAGCTACCAGACATGTAGCGATTGGCAGCCCAAAACTGATCCATGAGGCTCTTCTCATTTTCCTTTGGCTAGGGTATAATCAAATACCATGGAGCAACTCAAATTCATTTACCTGTATGTGCCAGTGTCTATTTGTTGCCCATGCAAATTAGAAATTGATATCTGTGCAAGTTACAATTCTGTCTACATGCAAATATGATTTCCTCCAGAATTTGACCTATGCACTTGATACCATTTAAAGAATCAGCACAAAGTCCCTAAAACAAAAACCACTTTGTGATTTGCACTGTGCCACTAACAACAAAAGGAAGAAATGGTGCATGTACCAATGAATGCCATTCTCATGCCTTCTGTCAGTGTACTTGACTGTACCCAATTGCACATCAAAGCACCCAGTTCCCCCTCCTCTCAGACCAGCGCACAGTCCAACCTTGTCTATTTGAATTTTTTTCTTCAAGTCATCTACACTCAGTTTTGACCGTGCGTAGCCGATCACTGTGGTTGCTGGTGGAAGGCGATTGCCTTTGAATAGTAACCTTTGCCATCAATGATGTGTGTGCACGTAGAGGGAGGGAATAGTGAGAATTTGAGAGATTGCGGGAGTGAAATTAAAGATAGTTAATTATAGCAGTGGTGATGCAAAGTACTTATCTGGCCTGACTCATGTGAATGTGCCTGCTTGTGCAAATCTATGCATAAAAGTTTCTCGTGCAGAAACACATGCATACAATCATAGACAAATTGACATGCGTGTACATGAGGTGTGCAAATACTAATACTGACAGTCAGCTGGTATATGCACTCACCACAGCGTTGGGTAGATCATTTTCTTTGCCAGGTCACCCTATATAAAAATCGCACAGTGGTGCAGATATATATGATCAACCGCACCAAATCCATTAATAGCCATGCATGCTTTCGTACAACATTCGGATGTAGCAGGCCCTGCACTGCATGTGTACCATACATTTGAAnCCTTTTGGCATATGTATTCATGGAAGGAGACATGGATATGTGTGCATGTGTGTGGGTGGGCAGGGGATAAAGCCTCCAGACACAACAGAGAAGTGCTGTGGTGTTACAAGTCCTGGTAGAAATCATTCGGTCTTTGTGTTTGCAGACAATGGAGGAGTGAAGCAGGAGTGATTCTGTGTGTGCATGTGTGTTACATTAGGTAGAGGAATGAAANACNNNGATATCATTGTGGGTGGAGCAAAGCAAAGGGTTAATGTGGCCCCCACCAGCTGGCCATCTTAAGCATGAAGAGGTGTGTTTGATAAAGAATGAGGTGTGGCTATATTGAACTGGTTACTCACACAGCTTGTAGTGATGTGATGGTGTGTGTATCTGTGTGAGCAGGTGTGGATGTGTGTTGGGTGTGTGAGCTGGTGTGTTTGTGTGTTGTGTGTGTGAGCTGGTGCAAAGGGTTAATGTGGCCCCCACCAGCTGGATATGAATGCCATCTTAAGCATGAAGAGGTGTGTTTGGGAAAGAATGAGGTGTGGTAATATTGAACTGGTGTTACTTCACACAGCTGTGCTGTGTGTTGCNANNTGTGTGAGCAGGTGTGGATGTGTGTTGTGTGTGTGAGCTGGTGTGTATGTATGTTGCACACATACAGTTTGGCTTTGACAGAAGCATTACACAACATGCAGCACACAAGTGTGAAGACATGCACATAAGAGCANCAGTGAGGTGTGAGGGGACAGAGTGGTGTGATACCATATGCTCCCTTTTGTCTCCGACAGGGGGGACCAGCTACACAGCTGGCACAGTGCCTTGTTTGTGGTAGTCAAGCATTGTCTGGTGGTTGGCTTTGTTGACCCATTAACTTTGGCTGCACAGATTGTGCGAGGTTGCCCAGTGGCCAGCGCAAATGCCAATTTGTACAGTGCCAATACATGTGTGTGTGCATGTACAGTGTACATGTATGTATTGTGTGTGTGTGTGTGTGTGTGCATCAATCTGTGTGCACGCGTGTATGTGTGCATGTGTGTGTGTGCATGTGTATGTGTGTGTGGTGGGTGTGAATTGCCNCCATGACACCTTGACTCTGTGTGCTGCTGCAGGTACAAGAAGGAGTGCAATGAGCTGAAGGAAGAGAACCGAAGGATCCGACAGGACCACACGGAAGCCTCGAGAGAGAAGGACAGACTCCGCTCGGAGATGGAGCAGGTTCAGAAGGAGATGAACAACCTGAGCAAGTACAAGAAGGAGTACGTGACACTGCTCGAATCCAACAGGTCCCTGGAGGAAGAGATGGGACTCCTCAACGACCACTACAAGAGCATCTTGGAGGAGAAGGAAGGACTGGAGGACCAGACCAGGGAGACGATTCAAGCCCTGAACGAGGAGAGAGAGGCCAAAGACCTTCTAGAGAGGAAGCTGAAAGACGCCTCCCTCAGACCCCAGTCCCCCAGCTGGGAGGAAGAGACGGAGTCCAGAACCGAGGGAGGTCCCCTCACCAATGGCAGATGCTCCCCTACCTTCCAATCCACTCCAAGCAAGCCCCCGGTCCCCAGTCTGCTCAACGACCTCCACGACTCCATCATGGAGCAGCTGGACCTCGAAGCCCTCAAGCGCAAGACCAAGAAGGCTGAAGAACTGGTGGGTGTGCTCCAAAAGGAAAAGCAGTCCCTGGAGGCGAGGCTGGCCGAACAGGAGGCCGAGGTGGGGCGACTCAAGGAGATNNNNNAGGCGACGAGCAGCGAGCGGGACAAGGAGTTGAAGGCCTTGACAGAGGCGGCAGCCATACGGGACGAGCTCCTGGACCAGCTCAAGGGCAAGCTGAGCGCAGTGTCCACAGAGAAGGCCAAGCGCGAGATAGACCTGGAGGAGCTCAGGGAGGAGCTGAACCGACTCAGGAAGTACACGAGCGCTGAGGTGGAGAAGACTCAGAGCGAGTGCACCCAGGAGCAGAACAAGAACGTGGAGCTGAGGGCCAGGACGACGGCCCTCGAGCAGCAGCTAGGGGAGGCTGCAGGGACGGCCCAGAAGCTGGAGGGCGCCCTCTACGCCACCCACTCCGAGCTGGCGGCCATGACGGAGGATATGAGGAGCATGCAGAAGGCCATCGTCACCCTCTGCTCCGACAGCAGGCTGAGCGGGAGGAGCCTGACATCCCGTGACATGACCCCTTCCTCCCCCGAACCAGGCACGGCGGAGGAAGGGGAGGATCCCCAGAGGGTGGGGTCTGGTGGGAGTGGTGTGGGAGGGGCTCCGAGGTTCTTGCCTCTGGAGCTGAAGCAGTCCAAGGTGACAGTTCAGGTGCACAGTGAGAGTCACACCCTCATGGCCCTGGTGCAGCTCCACGACCAGCTGCGTTCCCTGCGTCTGCCCCTCGAGCAGTTCACCCGCATCATGCTCGAGCGCTCCCTGGCACACTCGGCCAAGCATGGCACGGAGGTGACCCAGAGNGNGCNNGNNGGAGGAGGAGGGGGAGGAGGGGGGAGGAGTCCGGCAGAGGTGGAGGCCATCATCGGGAAGTGGAAGGCGAAAGTGGCGCACAAGACGGAGGAGGTGAGTAACCTCCGCGCCATCATGAAGGCCAGGCAGACAACATCGGACGTGGCGCTGAGCACCCTGAAGAGCAAGCTGGAAGGGCAGGAGAGGACCTACCAGACTGAGCTGGCCCGACTCAAGTTCCAGATCAAGTCCCTGAAGAAGGAGAGGGACGAGGGGAGCAGCCTCAAGGCCATGTACGCCCAGAGGTGCGAGGACTACATAGACGAGATGGGGCGACTCAAGAAGGAGATACATGGGCTCCAGATGGAGAACGAGGAGCTGCTCGTGTCTCTGAAGAAGACCATCCAGAAGAAGCTGGACCTGAGCACGCAGCTGGAGGAGTACCAGATCGAGAGGGAGAGGGCACTCCACATCCCCAAGCTGCTGTTGGCATCACGAGTCTAACAGACACACACACACACACAGATCAATCTCCTGTATGCCCACCACCCCTTTTTAAAACTACTACAGCATTGTATGTTTATATTCCATTGGTTTATTGAGAAATCCATCAACAAGTGATTGTTCTTTAATAAGTATAGTAGTACCATAGCATTAAAGGACAGGAGGCTTCACATACATTGTACACCACACCACACAGAGCACAAGTGTTTCTGGGAAGGTTCCATCTAGACCTGTTAGGTTATATTTGATGTCTGCAATAATGTAGATGTTCAGACATAATTCGAATGCCAGAGAACCTATATGGGGTGTGACTGCTTTCTTGCCAAGATGACCATCAAATGGTCACCTTCACACATAAAAACATGCGAGTGCGTGGATGTCATACACAACTAAATACACATACTGTATAGTACTTACATGCACACACAAAAATTACATGTACATACATACATGGCACACGCACCCCACATGTACATACNNNNNNNNNNNNNNNNNNNNNNNNNNNNNNNNNNNNNNNNNNNNNNNNNNNNNNNNNNNNNNNNNNNNNNNNNNNNNNNNNNNNNNNNNNNNNNNNNNNNNNNNNNNNNNNNNNNNNNNNNNNNNNNNNNNNNNNNNNNNNNNNNNNNNNNNNNNNNNNNNNNNNNNNNNNNNNNNNNNNNNNNNNNNNNNNNNNNNNNNNNNNNNNNNNNNNNNNNNNNNNNNNNNNNNNNNNNNNNNNNNNNNNNNNNNNNNNNNNNNNNNNNNNNNNNNNNNNNNNNNNNNNNNNNNNNNNNNNNNNNNNNNNNNNNNNNNNNNNNNNNNNNNNNNNNNNNNNNNNNNNNNNNNNNNNNNNNNNNNNNNNNNNNNNNNNNNNNNNNNNNNNNNNNNNNNNNNNNNNNNNNNNNNNNNNNNNNNNNNNNNNNNNNNNNNNNNNNNNNNNNNNNNNNNNNNNNNNNNNNNNNNNNNNNNNNNNNNNNNNNNNNNNNNNNNNNNNNNNNNNNNNNNNNNNNNNNNNNNNNNNNNNNNNNNNNNNNNNNNNNNNNNNNNNNNNNNNNNNNNNNNNNNNNNNNNNNNNNNNNNNNNNNNNNNNNNNNNNNNNNNNNNNNNNNNNNNNNNNNNNNNNNNNNNNNNNNNNNNNNNNNNNNNNNNNNNNNNNNNNNNNNNNNNNNNNNNNNNNNNNNNNNNNNNNNNNNNNNNNNNNNNNNNNNNNNNNNNNNNNNNNNNNNNNNNNNNNNNNNNNNNNNNNNNNNNNNNNNNNNNNNNNNNNNNNNNNNNNNNNNNNNNNNNNNNNNNNNNNNNNNNNNNNNNNNNNNNNNNNNNNNNNNNNNNNNNNNNNNNNNNNNNNNNNNNNNNNNNNNNNNNNNNNNNNNNNNNNNNNNNNNNNNNNNNNNNNNNNNNNNNNNNNNNNNNNNNNNNNNNNNNNNNNNNNNNNNNNNNNNNNNNNNNNNNNNNNNNNNNNNNNNNNNNNNNNNNNNNNNNNNNNNNNNNNNNNNNNNNNNNNNNNNNNNNNNNNNNNNNNNNNNNNNNNNNNNNNNNNNNNNNNNNNNNNNNNNNNNNNNNNNNNNNNNNNNNNNNNNNNNNNNNNNNNNNNNNNNNNNNNNNNNNNNNNNNNNNNNNNNNNNNNNNNNNNNNNNNNNNNNNNNNNNNNNNNNNNNNNNNNNNNNNNNNNNNNNNNNNNNNNNNNNNNNNNNNNNNNNNNNNNNNNNNNNNNNNNNNNNNNNNNNNNNNNNNNNNNNNNNNNNNNNNNNNNNNNNNNNNNNNNNNNNNNNNNNNNNNNNNNNNNNNNNNNNNNNNNNNNNNNNNNNNNNNNNNNNNNNNNNNNNNNNNNNNNNNNNNNNNNNNNNNNNNNNNNNNNNNNNNNNNNNNNNNNNNNNNNNNNNNNNNNNNNNNNNNNNNNNNNNNNNNNNNNNNNNNNNNNNNNNNNNNNNNNNNNNNNNNNNNNNNNNNNNNNNNNNNNNNNNNNNNNNNNNNNNNNNNNNNNNNNNNNNNNNNNNNNNNNNNNNNNNNNNNNNNNNNNNNNNNNNNNNNNNNNNNNNNNNNNNNNNNNNNNNNNNNNNNNNNNNNNNNNNNNNNNNNNNNNNNNNNNNNNNNNNNNNNNNNNNNNNNNNNNNNNNNNNNNNNNNNNNNNNNNNNNNNNNNNNNNNNNNNNNNNNNNNNNNNNNNNNNNNNNNNNNNNNNNNNNNNNNNNNNNNNNNNNNNNNNNNNNNNNNNNNNNNNNNNNNNNNNNNNNNNNNNNNNNNNNNNNNNNNNNNNNNNNNNNNNNNNNNNNNNNNNNNNNNNNNNNNNNNNNNNNNNNNNNNNNNNNNNNNNNNNNNNNNNNNNNNNNNNNNNNNNNNNNNNNNNNNNNNNNNNNNNNNNNNNNNNNNNNNNNNNNNNNNNNNNNNNNNNNNNNNNNNNNNNNNNCTCCTTATCTTGTGCACAAGCCCCCTCACAGGTGCTAGTTCAGCGTCCACATACTCAGAAGGCAGCTGTCTATAGAGACACATTAAAACTCCTACATGTAAAGCTTTGGTCGGAAATTACGGCACAGATCGATATGATATTGGAGGCCCCCNCAGGANGATCCNATGGTATANCTTTACGCTAACCCAAACAGGGCAATGTATTAGCACCGTACGTACGTAGCACTTAGGCACCAGCGAGAACCATGTGGATGCTTCTGCTGACATCTCAACGAGTGTGCATGCACATTGGGCGCACCCAGCCTTGTGATGTGATACATGCTGCCACCAACCCAACACCGTCGTGTGTCAAACATGCTAAGTACAAACGTGTGTTTGTTTTATTTTTTGTTTTGTTATTTTGTTTTGTTTTTTTAGATACCTGCGAGGTCTTGCTATCCTGTCAGGAACCGGAGATACTATATTAAAGGTCTCCAAACTACCTCTGCGTAAGGAGTCTTGCATCGTTAGTCCCGAGTCCTCTGCNNGNCNNAGCNNNGCACCNNNNNNNNAGCTNCAGGAAACGATTGTTTTCATCGACGACAGGGCGTGGTTCGATAACATAGATATTCATTCGCTTTTTCTTAATCACAAACAACTTTTTATTAACTCTCACATATAACGTTAAGTCTGATACTTGCGCCAAGNCTGTGTATGTAGCTAAGCAAGCTCGCTCTATTTGTTATTTTACGTACATTATTTTCATGGATTCCTCCTATAAAGCACCAGACGCGGTTCCACAATTATGAAAAAAAAATTGCATTGTGTTTTGATTGCAGAACAAGTTGAATAATATTTTTGTGATATTCGTGTAAGAATAGATTTGTTAGAATTCCCTTCAGTTAATTAGTCATGTTCTTATAAAATAAGATAACAACAAGACTGTCAAAGCCACTGAACCTGGAACTTAAACTGATTTGTAATACTGAATAACTCAACGATGCCACGGGCATAGATCTATACAATCTGGCAACCTGCAAAATTAATAAATGATTCGTTACGAATANCATTATTACATGTGTACACAGNTTAACAATGACAAGAACACTAAACAAAAATCACTCGCTACCNAAAATACCAGGCCAGTTTGATCTGGCAAGGATTGCCTGTGCATTTTGTCTCATTTAGGACTAAGCTCATGATCAGTCTGCCAAATGACTCCTTCCTCATCGCCACTTCATTGGAACTGAGCTGAGCACACAGCCCGCAGTCTCTTGCAGGTACACCTTAAGGTCTCACAGTCTTTTCTCCCAGCCACCATAAGTTTCTACCGCCAGCGAATTACACTCCCAGATTAATGATTTTTATTTCTTTATTCAATGAACTCAACACNTTGGGCTTTCATAAATGTCTAATGTGAAACCGACAGTAAATTATATGGTAATAAATAACATGCGTTCGAATCATAAGGACTGAGGCACAGATTCACTGCCTGTCAGTTCAATGGAGGTAGGAAGGTGTATAGCATGCCCATGATTGGACCGTGCTCCTCTGATGCATTGGATGGAGGAGAGTAAAAGTGAACAGAAAAATGACCAATTTAATGACTGATTTCGATTTTCACAATTTTCATATTTACTATATACATTATTATNATNATACATAATACNATANNTATATCATNACNATNNNACTANNACAANANNNATCTAANCACTGTCCAGCTGAGCCAGGATTGGTGGAGACCTGGCTTGCAAAGCTCTTGCAGTGGCACGCATAAGTTTATACATGTCTATCGCGTGGTAATAACAATTGCATCGTTGTGGCTTTGACAAGTTTCTTTCTGTGGCCAGCAGCCAATGATGCTCGTCCCCAGCTTTAGTGCTTTTCAATATAGTATCTCTAATAGTCTCGTACCCAGACCCGTTACGTAATGCTGCATANNNNNNNNNNNNNNNNNNNNNNNNNNNNNNNNNNNNNNNNNNNNNNNNNNNNNNNNNNNNNNNNNNNNNNNNNNNNNNNNNNNNNNNNNNNNNNNNNNNNNNNNNNNNNNNNNNNNNNNNNNNNNNNNNNNNNNNNNNNNNNNNNNNNNNNNNNNNNNNNNNNNNNNNNNNNNNNNNNNNNNNNNNNNNNNNNNNNNNNNNNNNNNNNNNNNNCACCTAATTATACACGGGTTCTCCTCAAAGCTGCTTTGAAAGCATCTGCCATGCCCTGGTCTCTCGTCTTTAACCATTATCTCCTCATCCTCCCCCTGCAGAATCATGGTGACTCCATTCCAAGCATCGTAAGATGACTGTTTGTGACCCATCAACCATGGTGATGGAAAAGAGGCTCCCCATGCACCTTCACCATATATATGCTTCAATCTGCTTAAGCTGCCCAACTACAGTAAGAAGTCAGTATCCAGGGGCAAGTTCAAGTATGCCGTAACTTCTGGGGCAGGGTTTGAAAGAGAAAGGAGAGGGAGGGAGGGAGGNAGGAGAGGAGAGGGAGGTAGGAGAGGAGAGGGACATGAAACCAACTACTAGAGACAAAATGACACACTTATGTAACACATTTGGAAGTATTAAAGTGTTTTACAATTATGTATAATAACATTTACATGTGCATGTAGAAATAAGTACAGATGGCCACCACATGCCCACATGCACACACAGGTACACTGTGTACATGAACGAGTAGATACACTTTGTACGTGGATATGTAAACTTGTAGCTTACATAGAAATCACCAAGAGGAATAACGAAAAATGCACAAAAAAAGAGGGGGAGGGAATAGATACATGTATTTGATATGGAGGAGGTGGTATCTGCGCCCACACTTACTTCTGAACCTGAGGAGCAGTCTGAACAGCCACAGAGCAGTGAAGCACGCACACATCTGGCCCACCAGGTGGCCTGCAGTGCTACCTGGCCTCATGCCCAGAGCAAACTGCAAGAAGCTACGTGAGAGAGGTGCAGGCCATGTCACCACCACGACACAAGTAAGTATGCCTCAAGCAAAACATCTGCAATATTGCTAATGACCTTTTGATTTGTATTTACTCTGACTGGCCCAGACCTGACATGTTTGGTTCTGTTAATTGTCTCCTTCATTCCTTCCTAAAACCACATCCATATTCGTGGTAAATAACCTAATACATATATGTTGCCACAATGGCTTCATAGTTCAAATAGCAGCAAACCCTGTAATATTTACAGATCCAAACCCATTCCAGAGGGCCCAGACCCATTCCAGAGTGCAGATCCACGCCAGGGCCCATACCCATTCCATTGCCCGGACCCGTGCTCACCCCATAATGATGATGACCACATCGGCTACATTGTAGTACTGGCGTAGGAAGGCTTTGTGGTGGAAGAGGAGGCCATAGGAGATGACCTTGAGGAAGGCTTCAGCAGCAAAGCAGAGCGAGGTAGCCGCCACCACCCACCTCTGAGCAAAGGCACATGCAACAGTGACTAGTGGGGTCACGGATGAGATGACTGACCAGCACGTGNCACCTACACCCTAATCAATGCACATCGCTGCACTGATTACATTGACATATGCATGAATGCTTGCACGAATTTAATAGTATGGATATTGTCTCTTTTTTTTTTTNNNNNNNNNNNAGGTGAGAGATAGATGTGCATGCATGCAATGGTGTCTACATTCTTTCAGTGAAACTAAACACATTATGGTGATTAAGTAAGGAAGTTAACACATGTGTGCTGTTAATTAACCATGACATGGGTGGGCTGGGGTGGAGATTGTATAACTAGGGACTTTCCCCATAGCTTGAAATCTAACAGCCCGCTAATAGCTCCGTACATACATGTACATACAACATCCCCACCTCCTCTACGGCCATCTATGGCCACACCCCTCATCTATGGCTACACCTCTCCTCTATGACCACACCCTCCTCTATGGACACACACCCTTCCCCCTCACACTTACTATCCAGGCATTGGATTGCTGGGAGTCAAGGTTGGGTTCAGGCAAGAAGAGGGCAAGAGCAATGGCATTGAGTATCACCACCAGACCCAGAAAGACTTCAAGAATCCTGTACAATGCACACCAATGATGACATCATCATCCTCAATGATGTGCCCTCACCCTCCCCCGCACACGCACTCACTGCACCTGGATTTTACAATGCGCTTTGCCCTCTTGCGAAAACTGTTCTGCTCTTCGAACAGGAACAGGGCCTTATTCCTCTGGTTGAGTTCGTTCAGCTCTCTCCTTATCTTGGTACAANNNNNNNNNNNNNNNNNNNNNNNNNNNNNNNNNNNNNNNNNNNNNNNNNNNNNNNNNNNNNNNNNNNNNNNNNNNNNNNNNNNNNNNNNNNNNNNNNNNNNNNNNNNNNNNNNNNNNNNNNNNNNNNNNNNNNNNNNNNNNNNNNNNNNNNNNNNNNNNNNNNNNNNNNNNNNNNNNNNNNNNNNNNNNNNNNNNNNNNNNNNNNNNNNNNNNNNNNNNNNNNNNNNNNNNNNNNNNNNNNNNNNNNNNNNNNNNNNNNNNNNNNNNNNNNNNNNNNNNNNNNNNNNNNNNNNNNNNNNNNNNNNNNNNNNNNNNNNNNNNNNNNNNNNNNNNNNNNNNNNNNNNNNNNNNNNNNNNNNNNNNNNNNNNNNNNNNNNNNNNNNNNNNNNNNNNNNNNNNNNNNNNNNNNNNNNNNNNNNNNNNNNNNNNNNNNNNNNNNNNNNNNNNNNNNNNNNNNNNNNNNNNNNNNNNNNNNNNNNNNNNNNNNNNNNNNNNNNNNNNNNNNNNNNNNNNNNNNNNNNNNNNNNNNNNNNNNNNNNNNNNNNNNNNNNNNNNNNNNNNNNNNNNNNNNNNNNNNNNNNNNNNNNNNNNNNNNNNNNNNNNNNNNNNNNNNNNNNNNNNNNNNNNNNNNNNNNNNNNNNNNNNNNNNNNNNNNNNNNNNNNNNNNNNNNNNNNNNNNNNNNNNNNNNNNNNNNNNNNNNNNNNNNNNNNNNNNNNNNNNNNNNNNNNNNNNNNNNNNNNNNNNNNNNNNNNNNNNNNNNNNNNNNNNNNNNNNNNNNNNNNNNNNNNNNNNNNNNNNNNNNNNNNNNNNNNNNNNNNNNNNNNNNNNNNNNNNNNNNNNNNNNNNNNNNNNNNNNNNNNNNNNNNNNNNNNNNNNNNNNNNNNNNNNNNNNNNNNNNNNNNNNNNNNNNNNNNNNNNNNNNNNNNNNNNNNNNNNNNNNNNNNNNNNNNNNNNNNNNNNNNNNNNNNNNNNNNNNNNNNNNNNNNNNNNNNNNNNNNNNNNNNNNNNNNNNNNNNNNNNNNNNNNNNNNNNNNNNNNNNNNNNNNNNNNNNNNNNNNNNNNNNNNNNNNNNNNNNNNNNNNNNNNNNNNNNNNNNNNNNNNNNNNNNNNNNNNNNNNNNNNNNNNNNNNNNNNNNNNNNNNNNNNNNNNNNNNNNNNNNNNNNNNNNNNNNNNNNNNNNNNNNNNNNNNNNNNNNNNNNNNNNNNNNNNNNNNNNNNNNNNNNNNNNNNNNNNNNNNNNNNNNNNNNNNNNNNNNNNNNNNNNNNNNNNNNNNNNNNNNNNNNNNNNNNNNNNNNNNNNNNNNNNNNNNNNNNNNNNNNNNNNNNNNNNNNNNNNNNNNNNNNNNNNNNNNNNNNNNNNNNNNNNNNNNNNNNNNNNNNNNNNNNNNNNNNNNNNNNNNNNNNNNNNNNNNNNNNNNNNNNNNNNNNNNNNNNNNNNNNNNNNNNNNNNNNNNNNNNNNNNNNNNNNNNNNNNNNNNNNNNNNNNNNNNNNNNNNNNNNNNNNNNNNNNNNNNNNNNNNNNNNNNNNNNNNNNNNNNNNNNNNNNNNNNNNNNNNNNNNNNNNNNNNNNNNNNNNNNNNNNNNNNNNNNNNNNNNNNNNNNNNNNNNNNNNNNNNNNNNNNNNNNNNNNNNNNNNNNNNNNNNNNNNNNNNNNNNNNNNNNNNNNNNNNNNNNNNNNNNNNNNNNNNNNNNNNNNNNNNNNNNNNNNNNNNNNNNNNNNNNNNNNNNNNNNNNNNNNNNNNNNNNNNNNNNNNNNNNNNNNNNNNNNNNNNNNNNNNNNNNNNNNNNNNNNNNNNNNNNNNNNNNNNNNNNNNNNNNNNNNNNNNNNNNNNNNNNNNNNNNNNNNNNNNNNNNNNNNNNNNNNNNNNNNNNNNNNNNNNNNNNNNNNNNNNNNNNNNNNNNNNNNNNNNNNNNNNNNNNNNNNNNNNNNNNNNNNCACATGTACACACACANACACACACANGTACACACACNTGNNNCCACACATGTACACACACACACATGCACACGCACACAGGTACACATAGNCACAGGCACACACACAGGCACACACAGGCACACACACACTGTACCATCAGGAGCCATGAAGTCAGCATAGCAGCCCTTATGGAGGTCACCTCTGAACACATCAAGCCCAATGAGGGCAAAGAAGAAGATGACGATTAAGCTGAACACCATGAAGTGAACCAGCGGAGTCACAGACTTGGCCAAAACCTTGAGGATAAACTGTGGACCTGTGGGATGGGGAGGAGGGGGAGGGAGAGAAGGGAAAGGGAGGGAGGTAGGAGAGGAGAGGGAGGGAGGTAGGAGAGGAGAGGGAGGGAGGTAGGANAGGAGAGGGAGGGAGGGAGGTAGGAGAGGAGAGGGAGGTAGGAGAGGAGAGGGACATGAAACCAACTACTAGAGACAAAATGACACACTTCTGTAACACATTTGGAAGTATTAAAGTGTTTTATAATTATGTATAATAACATTTACATGTGCATGTAGAAATAAGTACAGATGGCCACCACATGCCCACATGCACACACAGGTACACTGTGTACATGAACGAGTAGATACACATTGTACGTGGACATGTAAACTTGTAGCTTACATAGAAATCACCAAGAGGAATAACGAAAAGTGCACAAAAAAGAGGGGGAGGGAATGTATTTGATATGGAGGAGGTGGTATTTGCGCCCACACTTACTTCTGAACCTGAGGAGCAGTCTGAACAGCCACAGAGCAGTGAAGCACGCACACATCTGGCCCACCAGGTGGCCTGCAGTGCTNNNNNNNNNNNNNNNNNNNNNNNNNNNNNNNNNNNNNNNNNNNNNNNNNNNNNNNNNNNNNNNNNNNNNNNNNNNNNNNNNNNNNNNNNNNNNNNNNNNNNNNNNNNNNNNNNNNNNNNNNNNNNNNNNNNNNNNNNNNNNNNNNNNNNNNNNNNNNNNNNNNNNNNNNNNNNNNNNNNNNNNNNNNNNNNNNNNNNNNNNNNNNNNNNNNNNNNNNNNNNNNNNNNNNNNNNNNNNNNNNNNNNNNNNNNNNNNNNNNNNNNNNNNNNNNNNNNNNNNNNNNNNNNNNNNNNNNNNNNNNNNNNNNNNNNNNNNNNNNNNNNNNNNNNNNNNNNNNNNNNNNNNNNNNNNNNNNNNNNNNNNNNNNNNNNNNNNNNNNNNNNNNNNNNNNNNNNNNNNNNNNNNNNNNNNNNNNNNNNNNNNNNNNNNNNNNNNNNNNNNNNNNNNNNNNNNNNNNNNNNNNNNNNNNNNNNNNNNNNNNNNNNNNNNNNNNNNNNNNNNNNNNNNNNNNNNNNNNNNNNNNNNNNNNNNNNNNNNNNNNNNNNNNNNNNNNNNNNNNNNNNNNNNNNNNNNNNNNNNNNNNNNNNNNNNNNNNNNNNNNNNNNNNNNNNNNNNNNNNNNNNNNNNNNNNNNNNNNNNNNNNNNNNNNNNNNNNNNNNNNNNNNNNNNNNNNNNNNNNNNNNNNNNNNNNNNNNNNNNNNNNNNNNNNNNNNNNNNNNNNNNNNNNNNNNNNNNNNNNNNNNNNNNNNNNNNNNNNNNNNNNNNNNNNNNNNNNNNNNNNNNNNNNNNNNNNNNNNNNNNNNNNNNNNNNNNNNNNNNNNNNNNNNNNNNNNNNNNNNNNNNNNNNNNNNNNNNNNNNNNNNNNNNNNNNNNNNNNNNNNNNNNNNNNNNNNNNNNNNNNNNNNNNNNNNNNNNNNNNNNNNNNNNNNNNNNNNNNNNNNNNNNNNNNNNNNNNNNNNNNNNNNNNNNNNNNNNNNNNNNNNNNNNNNNNNNNNNNNNNNNNNNNNNNNNNNNNNNNNNNNNNNNNNNNNNNNNNNNNNNNNNNNNNNNNNNNNNNNNNNNNNNNNNNNNNNNNNNNNNNNNNNNNNNNNNNNNNNNNNNNNNNNNNNNNNNNNNNNNNNNNNNNNNNNNNNNNNNNNNNNNNNNNNNNNNNNNNNNNNNNNNNNNNNNNNNNNNNNNNNNNNNNNNNNNNNNNNNNNNNNNNNNNNNNNNNNNNNNNNNNNNNNNNNNNNNNNNNNNNNNNNNNNNNNNNNNNNNNNNNNNNNNNNNNNNNNNNNNNNNNNNNNNNNNNNNNNNNNNNNNNNNNNNNNNNNNNNNNNNNNNNNNNNNNNNNNNNNNNNNNNNNNNNNNNNNNNNNNNNNNNNNNNNNNNNNNNNNNNNNNNNNNNNNNNNNNNNNNNNNNNNNNNNNNNNNNNNNNNNNNNNNNNNNNNNNNNNNNNNNNNNNNNNNNNNNNNNNNNNNNNNNNNNNNNNNNNNNNNNNNNNNNNNNNNNNNNNNNNNNNNNNNNNNNNNNNNNNNNNNNNNNNNNNNNNNNNNNNNNNNNNNNNNNNNNNNNNNNNNNNNNNNNNNNNNNNNNNNNNNNNNNNNNNNNNNNNNNNNNNNNNNNNNNNNNNNNNNNNNNNNNNNNNNNNNNNNNNNNNNNNNNNNNNNNNNNNNNNNNNNNNNNNNNNNNNNNNNNNNNNNNNNNNNNNNNNNNNNNNNNNNNNNNNNNNNNNNNNNNNNNNNNNNNNNNNNNNNNNNNNNNNNNNNNNNNNNNNNNNNNNNNNNNNNNNNNNNNNNNNNNNNNNNNNNNNNNNNNNNNNNNNNNNNNNNNNNNNNNNNNNNNNNNNNNNNNNNNNNNNNNNNNNNNNNNNNNNNNNNNNNNNNNNNNNNNNNNNNNNNNNNNNNNNNNNNNNNNNNNNNNNNNNNNNNNNNNNNNNNNNNNNNNNNNNNNNNNNNNNNNNNNNNNNNNNNNNNNNNNNNNNNNNNNNNNNNNNNNNNNNNNNNNNNNNNNNNNNNNNNNNNNNNNNNNNNNNNNNNNNNNNNNNNNNNNNNNNNNNNNNNNNNNNNNNNNNNNNNNNNNNNNNNNNNNNNNNNNNNNNNNNNNNNNNNNNNNNNNNNNNNNNNNNNNNNNNNNNNNNNNNNNNNNNNNNNNNNNNNNNNNNNNNNNNNNNNNNNNNNNNNNNNNNNNNNNNNNNNNNNNNNNNNNNNNNNNNNNNNNNNNNNNNNNNNNNNNNNNNNNNNNNNNNNNNNNNNNNNNNNNNNNNNNNNNNNNNNNNNNNNNNNNNNNNNNNNNNNNNNNNNNNNNNNNNNNNNNNNNNNNNNNNNNNNNNNNNNNNNNNNNNNNNNNNNNNNNNNNNNNNNNNNNNNNNNNNNNNNNNNNNNNNNNNNNNNNNNNNNNNNNNNNNNNNNNNNNNNNNNNNNNNNNNNNNNNNNNNNNNNNNNNNNNNNGTGGGGGGGGGGGGGNGAGGCAACTGTAAAGTGCTGACGAGGCACACACAGGATATTCAAGTGTACTTTGCACGTGTATGTGCAATGCTTTTCAACAGTCACAGGGCACATGTATTTAACTTCCTATTACAATTAGATCATTGCTTCATTTTGTAACATTTTAAATGTTTAATTCAGTTTATGAGTTCCGACAAAGATTTTAAAAACTGACAACTGCATAATTTAGTGAAATAAAATTTGCATATAATNTTTGTTCGTATTTCATTAGAAATTCAAACCAAGTAACCAAACTTTTAAAAGCTCTCAATAATGGAAATGTTTCTTTTCACCAGTTTTGCTTACTGGTACGTTATTCATCATGGACCAGTAAGTCACGTTTCCTCCAATGCATTAGTTTGGTCACAATCTCAATGTATTTTAGAAGGCTGTGATGTACTAGTAGTACAGTGTGCACATGTATGCAGTTACCTAAAGCTGTTTGTCACAATCTATTTGCACCAGCATTATTAANATTTATAAAATCTGAAGGCTGACTCATTTTAATACTGAGCAATACAGTTTGCATTGGGTTCATTTTTACACGTACACATGCATGGCATCCGTGAGCAGCAAAGTACAATGCCATCAGATTACAGCACTCACACACACACACACACACACCACAGGATGTGCCCCTCATTCAAGGCTCATATTAACACAAGCACTCTGCACAACTGAAGATCGAGCGAACCACACACACACACACACACACACATGCACANACACANACACACATGTACACACACATACACACACATGTACACACANNNNNNNNNNNNNNNNNNNNNNNNNNNNNNNNNNNNNNNNNNNNNNNNNNNNNNNNNNNNNNNNNNNNNNNNNNNNNNNNNNNNNNNNNNNNNNNNNNNNNNNNNNNNNNNNNNNNNNNNNNNNNNNNNNNNNNNNNNNNNNNNNNNNNNNNNNNNNNNNNNNNNNNNNNNNNNNNNNNNNNNNNNNNNNNNNNNNNNNNNNNNNNNNNNNNNNNNNNNNNNNNNNNNNNNNNNNNNNNNNNNNNNNNNNNNNNNNNNNNNNNNNNNNNNNNNNNNNNNNNNNNNNNNNNNNNNNNNNNNNNNNNNNNNNNNNNNNNNNNNNNNNNNNNNNNNNNNNNNNNNNNNNNNNNNNNNNNNNNNNNNNNNNNNNNNNNNNNNNNNNNNNNNNNNNNNNNNNNNNNNNNNNNNNNNNNNNNNNNNNNNNNNNNNNNNNNNNNNNNNNNNNNNNNNNNNNNNNNNNNNNNNNNNNNNNNNNNNNNNAACACACACACACANACACACACACACACACACACACCAGATGTTCTTTCCCTTTAGCTCTATGAGAGTATTCTGCACATCGCTGGCTGCCGTTTCCATCTTTGAGGTGGGGTCAAATAGCCAACTTGGACCTGTGGAAAGATGGAGATGACTGCCTCCTGTGTTGCATCACATACTTCCCTACCAGAACACTGTAACCCAGCTTTGATCAAACATGCAGGCACTTCAACCTTATGTACTCGTGTACACCACCTACATCCAGCTGCACCTGCATGTACCCCATAAGCCCATACATGCATAGACACATAAATGTGCTGCATTTGCACACACATACATGTACACACAGTCAATGCACGTACAAACATAAATACACACCCAGACACACCCCATACCCACAAACAATTACACATCNATGAACTTACGTCTTGCTCCACTTCCATCGGTCACATNNNCAGACTCCACTGAAAAGATATAGACACATCAAATACTGCCCCCCCTCTTCCTCCCTCCCCCTCCTCCCTTCCCTCCCTTCCTCCCCCTTTCTCCCCTTCCTCTCCCTCCCATCTCCACATAACATTCATCGCTGTCTCAACCACTAATAGCCCACACTGAACCACAAACAGTTTTTCTGAGCTATGAGAAATCATATTGAAACGGAAAAAACAAAACAAAGGGTGATAAATGTATATTACTGTACATTTTGCTACACGAAATTATGTGTGCATGCATGTGTGTGTGCATGTGTGTGTGAGTGCATGTGTGTGTGTGTGTACGTTACATTTGGACAATACTGCACATGTACAGTGTAGGNTAAAGTACCTGTTACGATACAGTGTACATACATGCACATCATGTACAACACTATCCCCATGAGTCCAAGGCTGCCTGCCACCGTGTCTAGCATACTTACCCGTACCTGCTCNTAAATACACACACCCCCTTCGTGAGATCGCATCACGAATGCATCCCTGTGCATGTCTCAAAGGCTAAAACCATCCCACTGAATGATTACCACCAGTCTGACTAGCACACAACATGCAACTATGGATTAGGGGACAGGAGATGGGGAGGGGGAGGATGGGATAGGGAGGGGAGGGAAAAAGGGAGGGAGGGGAGGAAGGGTAATGAGGGCACAGAAAGGAGGAGGGGAGGGAGGGAGGAGNATATCACACATGCAAACATCACTGACTTTAAAAATCACAATAACACCCTGGTGCCATCAGGTACAGTTGGCGAATAAATAGATCACAAAAGAAACAACCTTCTCACGGTATCAAGGAATATAATGCCATAAGGAGCAGTGTGGGGACCTACGAGCTTTCCTCATCCACTGACGGTAATTCTGGATGTCCTCATCATCCCTCTGCTTCTCCTTACTCCTGCGCACCTTGCCCTGCTCCTCCTCCCTCTCACTGACACCCTGGAACACACTGGAACACGCACATCATCATAACACACACTCACACTGTACACACACANGCTCACACATGCACTTACGCATTACAAGTAAATGTGTAGAGTATGTTTATGTTTTGCATATACATATCACTGCAGGCCATCAGGTACATGTAACGAAATTATGGCAATTGCACACTTTCNCCCACACACCCACCCCTCCACTTCCTTATAATCTACCACAATCCAATTGTTACAAAACAAGTTGTACCTTATGTNTCATCAACTGAAGATACAACACATAATCTGCACTTTTACCAATTGCCATAAGTTCGTCTAAACATGGNACATGTAGATGAATGCAGTATGCTGTACGTATGCACATGGAACTGTAAACAAATCACTGAACTCTCAGTGTAACCAACTTTTGAATAGCTTTGTGTCCATCCTATTTGATAGCATCACAAAATCAGCAGACACAATTCAAAGCACAACACTGACCACACCCTCCTCTCAAAGCACAACACTGACCACACCNNNNNNNNNNNNNNNNNNNNNNNNNNNNNNNNNNNNNNNNNNNNNNNNNNNGACCACACCCTCCTCTCAANGTCCACACACACTGACCACACCCTCCTCTAGGGTCACAATTACCTGGCGACCACACCCAACAACAGATTCAGGACAAAGATGAAACCAAACAGGGCAACTGAGAGGAAGTATATCCAGACGTACGCAAAGCCTCGAATGGCTTCATACTGCAAGGGGAATGGAGAGGAGCAACGTACAAGTACGCACAAGACCACACCCCTTCCAGTGCACGCGCACACACACACACACATGCACGTATGTTCATGATCATCAAAAAAGAGCATCACCACACACACTACTTCACCAGTAAACCATTGCACATGTACCTTCCACAAGGGGAGGGGTCACCTCTCCTATGTGTTTGATTGTACAGACGAACCCGATTTATGCTAAAAATACGACTCCGAATGCATTTACGTGCACACACCACCCTCCATCTTATACCGGGTGTCCACGAAAGAACGCAATGCTTTTGAACGGGAATANNNNNNNNNNNNNNNNNNNNNNNNNNNNNNNNNNNNNNNNNNNNNNNNNNNNNNNNNNNNNNNNNNNNNNNNNNNNNNNNNNNNNNNNNNNNNNNNNNNNNNNNNNNNNNNNNNNNNNNNNNNNNNNNNNNNNNNNNNNNNNNNNNNNNNNNNNNNNNNNNNNNNNNNNNNNNNNNNNNNNNNNNNNNNNNNNNNNNNNNNNNNNNNNNNNNNNNNNNNNNNNNNNNNNNNNNNNNNNNNNNNNNNNNNNNNNNNNNNNNNNNNNNNNNNNNNNNNNNNNNNNNNNNNNNNNNNNNNNNNNNNNNNNNNNNNNNNNNNNNNNNNNNNNNNNNNNNNNNNNNNNNNNNNNNNNNNNNNNNNNNNNNNNNNNNNNNNNNNNNNNNNNNNNNNNNNNNNNNNNNNNNNNNNNNNNNNNNNNNNNNNNNNNNNNNNNNNNNNNNNNNNNNNNNNNNNNNNNNNNNNNNNNNNNNNNNNNNNNNNNNNNNNNNNNNNNNNNNNNNNNNNNNNNNNNNNNNNNNNNNNNNNNNNNNNNNNNNNNNNNNNNNNNNNNNNNNNNNNNNNNNNNNNNNNNNNNNNNNNNNNNNNNNNNNNNNNNNNNNNNNNNNNNNNNNNNNNNNNNNNNNNNNNNNNNNNNNNNNNNNNNNNNNNNNNNNNNNNNNNNNNNNNNNNNNNNNNNNNNNNNNNNNNNNNNNNNNNNNNNNNNNNNNNNNNNNNNNNNNNNNNNNNNNNNNNNNNNNNNNNNNNNNNNNNNNNNNNNNNNNNNNNNNNNNNNNNNNNNNNNNNNNNNNNNNNNNNNNNNNNNNNNNNNNNNNNNNNNNNNNNNNNNNNNNNNNNNNNNNNNNNNNNNNNNNNNNNNNNNNNNNNNNNNNNNNNNNNNNNNNNNNNNNNNNNNNNNNNNNNNNNNNNNNNNNNNNNNNNNNNNNNNNNNNNNNNNNNNNNNNNNNNNNNNNNNNNNNNNNNNNNNNNNNNNNNNNNNNNNNNNNNNNNNNNNNNNNNNNNNNNAAAGAGCAGAAAATTAAATCTTTGACCCTTCATCGCTAGTTCATCAATACTCAGCACAATGCACCCATATATGTACATGAACACANGCACATACACACACAAACACACACACAGAAAAAATGTACTGGGCTGGGGAGGAGTGGAATTTAGACCCTTACAGACGGAGAGGTCGAATCTTTGCATGTAGGTGATGNTGTCGGGTGTGTAGTGTATGGTCATGNGGAGCAGGGTGTTCAGGAGGCGCGTCCCCAGCATGGCCAGGATGAAGGGGTTGGACTGCACAATGAACTCCACATTGCTTAACACACCACGGCAGCAAGACAACAGAGAGGGGAAGGAGATGGTAGCGTATGACATCATTAGACGACTTGCCCCACCCTCACCACCCACAAAGACACGCACACAACACGTGCACTTCAGCCTCTATAAAGCAGCTAACCTACATGTATACATGNNTGNGCAACACAGAAATATATACACAATGCGCGGGGGTGTATGCAGTAGTGGGTTAACTTCACATGAGCCCACATGTGCAGTAGAAATGCAGGCAACACATACTCCATCACCCTAACACACAACATAACCCAAATACACACACACACATACACACACATATCCACACACACACATAAAAACANACACATATTGACACACACACACATGCACACACACGCATGCACNCACACACTTGCACACACACACACATGCACACACACACACANNNNNNNNNNNNNNNNNNNNNNNNNNNNNNNNNNNNNNNNNNNNNNNNNNNNNNNNNNNNNNNNNNNNNNNNNNNNNNNNNNNNNNNNNNNNNNNNNNNNNNNNNNNNNNNNNNNNNNNNNNNNNNNNNNNNNNNNNNNNNNNNNNNNNNNNNNNNNNNNNNNNNNNNNNNNNNNNNNNNNNNNNNNNNNNNNNNNNNNNNNNNNNNNNNNNNNNNNNNNNNNNNNNNNNNNNNNNNNNNNNNNNNNNNNNNNNNNNNNNNNNNNNNNNNNNNNNNNNNNNNNNNNNNNNNNNNNNNNNNNNNNNNNNNNNNNNNNNNNNNNNNNNNNNNNNNNNNNNNNNNNNNNNNNNNNNNNNNNNNNNNNNNNNNNNNNNNNNNNNNNNNNNNNNNNNNNNNNNNNNNNNNNNNNNNNNNNNNNNNNNNNNNNNNNNNNNNNNNNNNNNNNNNNNNNNNNNNNNNNNNNNNNNNNNNNNNNNNNNNNNNNNNNNNNNNNNNNNNNNNNNNNNNNNNNNNNNNNNNNNNNNNNNNNNNNNNNNNNNNNNNNNNNNNNNNNNNNNNNNNNNNNNNNNNNNNNNNNNNNNNNNNNNNNNNNNNNNNNNNNNNNNNNNNNNNNNNNNNNNNNNNNNNNNNNNNNNNNNNNNNNNNNNNNNNNNNNNNNNNNNNNNNNNNNNNNNNNNNNNNNNNNNNNNNNNNNNNNNNNNNNNNNNNNNNNNNNNNNNNNNNNNNNNNNNNNNNNNNNNNNNNNNNNNNNNNNNNNNNNNNNNNNNNNNNNNNNNNNNNNNNNNNNNNNNNNNNNNNNNNNNNNNNNNNNNNNNNNNNNNNNNNNNNNNNNNNNNNNNNNNNNNNNNNNNNNNNNNNNNNNNNNNNNNNNNNNNNNNNNNNNNNNNNNNNNNNNNNNNNNNNNNNNNNNNNNNNNNNNNNNNNNNNNNNNNNNNNNNNNNNNNNNNNNNNNNNNNNNNNNNNNNNNNNNNNNNNNNNNNNNNNNNNNNNNNNNNNNNNNNNNNNNNNNNNNNNNNNNNNNNNNNNNNNNNNNNNNNNNNNNNNNNNNNNNNNNNNNNNNNNNNNNNNNNNNNNNNNNNNNNNNNNNNNNNNNNNNNNNNNNNNNNNNNNNNNNNNNNNNNNNNNNNNNNNNNNNNNNNNNNNNNNNNNNNNNNNNNNNNNNNNNNNNNNNNNNNNNNNNNNNNNNNNNNNNNNNNNNNNNNNNNNNNNNNNNNNNNNNNNNNNNNNNNNNNNNNNNNNNNNNNNNNNNNNNNNNNNNNNNNNNNNNNNNNNNNNNNNNNNNNNNNNNNNNNNNNNNNNNNNNNNNNNNNNNNNNNNNNNNNNNNNNNNNNNNNNNNNNNNNNNNNNNNNNNNNNNNNNNNNNNNNNNNNNNNNNNNNNNNNNNNNNNNNNNNNNNNNNNNNNNNNNNNNNNNNNNNNNNNNNNNNNNNNNNNNNNNNNNNNNNNNNNNNNNNNNNNNNNNNNNNNNNNNNNNNNNNNNNNNNNNNNNNNNNNNNNNNNNNNNNNNNNNNNNNNNNNNNNNNNNNNNNNNNNNNNNNNNNNNNNNNNNNNNNNNNNNNNNNNNNNNNNNNNNNNNNNNNNNNNNNNNNNNNNNNNNNNNNNNNNNNNNNNNNNNNNNNNNNNNNNNNNNNNNNNNNNNNNNNNNNNNNNNNNNNNNNNNNNNNNNNNNNNNNNNNNNNNNNNNNNNNNNNNNNNNNNNNNNNNNNNNNNNNNNNNNNNNNNNNNNNNNNNNNNNNNNNNNNNNNNNNNNNNNNNNNNNNNNNNGGCGAAGACTAATCAACTGACGCAATACAACATTACCACACCTGTGGAATACTAAACTGAAACCGAGAGTGCAATTATCTGCATACCATACGAACACTATTCTCAGCACACAAAATGAAATATTGTTTTGGACAGGACTATAATCTAATAATAAACGTTAATTGACTGACACACCACACTGCCCATTCATGATTGTGTTCTGTAACTCTTAAGTACTGTAATTCTACTTTCTAAAAGACATCCCCCCTCCTCCCCATCCCCACCCATACATATCAGTGAAATATCCAAAATCACAAGTTGCACTACATATGATGCTAGAAGTAACCTTAGCAGTTGCGCTGGTTGCGTCTTGCCCACATCCAGATCAACTGATCCTGACTGAGCATTGCTACCTGCCCTGCTGAGGTGCTTATCCATCGCCTCCACGTGGGACGTCCTGTCTGCCCCCACCGGCTCAGACAGGGTCCGGCTGAGCGACACTGCAGACACCACGGACACACCTCCACCACCTCCACCTCCACCACCATCACCACCACTCCCGTCGTGCACTAGGGTATCCTGTGACATCATATTTTGTTCTTCTGAAGGAGGGGTGTGGTCCACCTCCNNNNNNNNNNNNNTCCGCACGTCCTCGCTGCTCGACGACAATCTGAACGACTTCATCCTATTCAGCAGGGATTTNCCTATGGCTCCGCCCACATCCCCGATGGGCGAAGACACCGACCGGTGCCACTCGGTCCGCTGACCTTCCACCATCTTGTAGGCCTCCGTGGGGATGTTGGCGTAGAACTCTATGGCCCTGCGCAGTGTGCGGCGGTGTATGTAGGGCTTGCTCGTGTTCTTGACGGCATTGATCTGCTTCTCCCTCTCCTTGCGCTCCTCCTCGCGCTGCTCGCTCCTCAGCTGGCTGTCTTCCTCCACCTTCCTCACCTCCGAGAGCTTGTCGAAGGCAACGGCAGTCACCACGCCCACCAAGACGACTGGTTGTGTTCGACGTTCAGGGAAATGTATTTGTATCTGCAATGTACCTGTACATACATGTATCGTGTTTCTGCATTCCATTACTGTGCTGTGCACTGCTATGGCATGTACCACAATCTACAGTGTACGTTTCAGAAATGTGTGGACCACACAAGCATCATGTGTCTATCAAGCTGTGCTATGAGATTGGCTGCAAGCTTCCTTACAGAAGCAAATCACAGCTTTTTACAGCATTGGCACGCATGAGAGTTAAGACATCAGTTTAATTTGTGTCTGGTGAGCTGTAAACCGTTGATCTGCACGCCTCCAAAGTGCTAAAGTGTCACTGATTCAAGGGGTTTGGTGTGTGGTTTGGTTGAGCAGCTACTGTAGCTGTGCAGCAACACTTGGTGTGCAATTAAACCTGGGCTGTTGAGCCATTATATGGTAGGTTTAGGTGAGGACGACTGTGATATGTAAAATGTGCACTACATATTCCAGATATCACAGGTTCCCCTGTGGGCACAGGTGCATAATGGAACATGATACATGCACAATGACAATCTAGAATAGCCACACAACGTGTATCACATGTATCATGGATAGAAGTAAGTGCACCACTTGTCCATGTACATAAGAGGACACGCCTCCACAGAATGTTTAGATCAACAATGGAGGCATGTTGCAGGCAGCACTGCTACATGATTCATTTGAATACCAACAAAACAAAACAAAAAGTTTGCAGAGGTGGTTTACCCAAGCATAAAATTAGGATGGTGTGGCTTTGCAATTGATTAAAGACCTATACAGTAGAACTGCGATATTACCCTGCTTAGCATTTACAAACATGCATGTGTAACGGTCATCTGAGGATACAGGATATGAACAGGACTTGCGACGTGAAGAACGAGTTAATCACGATCCCCGAGGCCAGCGGCTCAGCTCCCTCCACGTAGCGCTGCACGTAAGGTCTCACTGCGAGAGAGACCACATTGTTCCAATTCTCCGTGGTCTGCATCTGCATGTGCATGGGTACACAGCAGTGACCCCAACTCATGACAACATGCACTGTGCAGTCAAAACAATACGTAAAACAGAAACAGACTGCCAGACCATACATGCAACCACCTACAACTTCCCAAACACTTATTCACACTAGAACAGACAATAGTAAATTCACAAGCTAGCAAATTAAGCTACATGTAAAGTGGCATGGAGTACATCAAGACCTCTAAAATCAAGTTCAATTTATGCCACGTTCATTTTTAATACTATACAATGCCTGGTTAACCCTCTGGAACACTGTTGTATAAAATAATTCTTCTCGTGATCTGCTCTCCGGTTAATTAAGGAACCTCTACTGTGCTCTACTGTGTGCACAATCGACGCTGAACCAATCATGTGTCATACAAGTGTGAGCCACAGTGCTGCAAGTGGCATGAGGAGAAGGGAGCAGCCACAATAATCATTCGTATCACACATGAATACAATTCACAGATTGTTTTAAAGGCTAGTCTATTGTACATAACCACTTTATGTGGCTATGATTTACTTTTAAGATACATGTCAGTTAATAATTTGTATTATGCTGGGAAACTATAAAAAGCAAATTAAGGTCATGGAATAAGTCAAGACCTCTAATATCAAGTTCAATTTATGTCGCATTCAATTTTACTTTTTTCTCGTTAGCGCCTTTGGACTAGTGTTGTACAACATGTCTGCTTATATTCTAATTACAACNAACCTCACTTACTTTTAGCTACAGTAAGACACTCCACCACACATATATTACTTAATGACCTATACCACTACAAATCATCTAGAATTATTTTGCACATTTGGCACCACTTCCTTCTAATAAACAAATTGTGATAATGGTCCTATGAGCCATTAAGNNGTCCAACAAAACAGCTACATGTGTAGCTAGCGATTGTATTTTGATTCATTCAAAGTGGTTGTCAAGCATAGTAAGATGCGTGCCAGTGCAGACATGTCAAGACACACTACAGAAACCTGTACCTGGACTGATATAAGAAAGGCGTTGAAACAGTTATCAAAATTCGGTATCATGAATTTATCAATGTGCACCCTACAAATGAGAGAGAACATAATACAGGTAACTGGTGTGACGTCCCACCAAGTGACTTACAGCCCTCCAAACAGCCGCACACCAATGAGTGCGTAGACGAAGTCGACGATGAGTATGATGACCAGCACACTCAAGGTGAGCACAAACACATCAGACAAAATCCGTCCAATCTTCTTCACACTCTTCCAGTACCTTTGAGTAGCATTCATACATACAGCCATACACAAAAGGTAAACAACAGTAGATGTGTTTTGGCCTGGAATGAATGCTACATATTTTTGCATTTAAAAATTCTTAATGTAACTCTTTACACATCNTATTTTTATTGTCTATCTCACAAGTACACTGTAGATTGCAAGGTGCTACAAAATTTGTGCATCTGATTTCACTCTTTTTTTTTTACGTTAAAGGACAGAATTATAATTTTTAAAAGCATGACAACATCCTGACATATATGTACCAATAATTNNNNNNNNNNNNNNNNNNNNNNNNNNNNNNNNNNNNNNNNNNNNNNNNNNNNNNNNNNNNNNNNNNNNNNNNNNNNNNNNNNNNNNNNNNNNNNNNNNNNNNNNNNNNNNNNNNNNNNNNNNNNNNNNNNNNNNNNNNNNNNNNNNNNNNNNNNNNNNNNNNNNNNNNNNNNNNNNNNNNNNNNNNNNNNNNNNNNNNNNNNNNNNNNNNNNNNNNNNNNNNNNNNNNNNNNNNNNNNNNNNNNNNNNNNNNNNNNNNNNNNNNNNNNNNNNNNNNNNNNNNNNNNNNNNNNNNNNNNNNNNNNNNNNNNNNNNNNNNNNNNNNNNNNNNNNNNNNNNNNNNNNNNNNNNNNNNNNNNNNNNNNNNNNNNNNNNNNNNNNNNNNNNNNNNNNNNNNNNNNNNNNNNNNNNNNNNNNNNNNNNNNNNNNNNNNNNNNNNNNNNNNNNNNNNNNNNNNNNNNNNNNNNNNNNNNNNNNNNNNNNNNNNNNNNNNNNNNNNNNNNNNNNNNNNNNNNNNNNNNNNNNNNNNNNNNNNNNNNNNNNNNNNNNNNNNNNNNNNNNNNNNNNNNNNNNNNNNNNNNNNNNNNNNNNNNNNNNNNNNNNNNNNNNNNNNNNNNNNNNNNNNNNNNNNNNNNNNNNNNNNNNNNNNNNNNNNNNNNNNNNNNNNNNNNNNNNNNNNNNNNNNNNNNNNNNNNNNNNNNNNNNNNNNNNNNNNNNNNNNNNNNNNNNNNNNNNNNNNNNNNNNNNNNNNNNNNNNNNNNNNNNNNNNNNNNNNNNNNNNNNNNNNNNNNNNNNNNNNNNNNNNNNNNNNNNNNNNNNNNNNNNNNNNNNNNNNNNNNNNNNNNNNNNNNNNNNNNNNNNNNNNNNNNNNNNNNNNNNNNNNNNNNNNNNNNNNNNNNNNNNNNNNNNNNNNNNNNNNNNNNNNNNNNNNNNNNNNNNNNNNNNNNNNNNNNNNNNNNNNNNNNNNNNNNNNNNNNNNNNNNNNNNNNNNNNNNNNNNNNNNNNNNNNNNNNNNNNNNNNNNNNNNNNNNNNNNNNNNNNNNNNNNNNNNNNNNNNNNNNNNNNNNNNNNNNNNNNNNNNNNNNNNNNNNNNNNNNNNNNNNNNNNNNNNNNNNNNNNNNNNNNNNNNNNNNNNNNNNNNNNNNNNNNNNNNNNNNNNNNNNNNNNNNNNNNNNNNNNNNNNNNNNNNNNNNNNNNNNNNNNNNNNNNNNNNNNNNNNNNNNNNNNNNNNNNNNNNNNNNNNNNNNNNNNNNNNNNNNNNNNNNNNNNNNNNNNNNNNNNNNNNNNNNNNNNNNNNNNNNNNNNNNNNNNNNNNNNNNNNNNNNNNNNNNNNNNNNNNNNNNNNNNNNNNNNNNNNNNNNNNNNNNNNNNNNNNNNNNNNNNNNNNNNNNNNNNNNNNNNNNNNNNNNNNNNNNNNNNNNNNNNNNNNNNNNNNNNNNNNNNNNNNNNNNNNNNNNNNNNNNNNNNNNNNNNNNNNNNNNNNNNNNNNNNNNNNNNNNNNNNNNNNNNNNNNNNNNNNNNNNNNNNNNNNNNNNNNNNNNNNNNNNNNNNNNNNNNNNNNNNNNNNNNNNNNNNNNNNNNNNNNNNNNNNNNNNNNNNNNNNNNNNNNNNNNNNNNNNNNNNNNNNNNNNNNNNNNNNNNNNNNNNNNNNNNNNNNNNNNNNNNNNNNNNNNNNNNNNNNNNNNNNNNNNNNNNNNNNNNNNNNNNNNNNNNNNNNNNNNNNNNNNNNNNNNNNNNNNNNNNNNNNNNNNNNNNNNNNNNNNNNNNNNNNNNNNNNNNNNNNNNNNNNNNNNNNNNNNNNNNNNNNNNNNNNNNNNNNNNNNNNNNNNNNNNNNNNNNNNNNNNNNNNNNNNNNNNNNNNNNNNNNNNNNNNNNNNNNNNNNNNNNNNNNNNNNNNNNNNNNNNNNNNNNNNNNNNNNNNNNNNNNNNNNNNNNNNNNNNNNNNNNNNNNNNNNNNNNNNNNNNNNNNNNNNNNNNNNNNNNNNNNNNNNNNNNNNNNNNNNNNNNNNNNNNNNNNNNNNNNNNNNNNNNNNNNNNNNNNNNNNNNNNNNNNNNNNNNNNNNNNNNNNNNNNNNNNNNNNNNNNNNNNNNNNNNNNNNNNNNNNNNNNNNNNNNNNNNNNNNNNNNNNNNNNNNNNNNNNNNNNNNNNNNNNNNNNNNNNNNNNNNNNNNNNNNNNNNNNNNNNNNNNNNNNNNNNNNNNNNNNNNNNNNNNNNNNNNNNNNNNNNNNNNNNNNNNNNNNNNNNNNNNNNNNNNNNNNNNNNNNNNNNNNNNNNNNNNNNNNNNNNNNNNNNNNNNNNNNNNNNNNNNNNNNNNNNNNNNNNNNNNNNNNNNNNNNNNNNNNNNNNNNNNNNNNNNNNNNNNNNNNNNNNNNNNNNNNNNNNNNNNNNNNNNNNNNNNNNNNNNNNNNNNNNNNNNNNNNNNNNNNNNNNNNNNNNNNNNNNNNNNNNNNNNNNNNNNNNNNNNNNNNNNNNNNNNNNNNNNNNNNNNNNNNNNNNNNNNNNNNNNNNNNNNNNNNNNNNNNNNNNNNNNNNNNNNNNNNNNNNNNNNNNNN
[truncated: 163,882 more chars]
